# Genome‐wide association studies and genomics‐informed breeding for tuber dormancy in tetraploid potatoes

**DOI:** 10.1002/tpg2.70205

**Published:** 2026-02-19

**Authors:** Ao Jiao, Sanjeev Gautam, Jeewan Pandey, Douglas C. Scheuring, Jeffrey W. Koym, M. Isabel Vales

**Affiliations:** ^1^ Department of Horticultural Sciences Texas A&M University College Station Texas USA; ^2^ Texas A&M AgriLife Research and Extension Center Lubbock Texas USA

## Abstract

Upon harvest, potato (*Solanum tuberosum* L.) tubers enter a dormant state and do not sprout even under favorable conditions. Early dormancy break causes tuber spoilage and reduces postharvest quality. Common sprout control methods may compromise tuber quality, raise health and environmental concerns, and increase costs. Thus, potato varieties with long tuber dormancy are desirable to reduce tuber spoilage and lower costs for sprout prevention. This study aimed to (i) explore phenotypic variation in tuber dormancy length among advanced tetraploid potato genotypes and several public cultivars, (ii) understand the genetic basis of tuber dormancy, and (iii) obtain genomic‐estimated breeding values (GEBVs) using genomic selection. A panel of 216 tetraploid potato genotypes, mostly from the Texas A&M Potato Breeding Program, was grown in Springlake and Dalhart, TX, from 2019 to 2021, for tuber dormancy evaluation. An additional panel of 82 genotypes was evaluated in 2022. A wide range of tuber dormancy lengths was observed, and significant variations were found among genotypes, field locations, storage conditions, and market groups. Quantitative trait loci were detected on chromosomes 1, 2, 3, 5, 6, 9, and 11, each explaining up to 11.8% of phenotypic variation. In addition, GEBVs were obtained using StageWise. Potato genotypes with high GEBVs for tuber dormancy length are potential donors of genes that can extend the dormancy period. Variation in tuber dormancy among advanced tetraploid potato genotypes highlighted the potential for selection. The genomic regions identified and the GEBVs provided valuable insights into the genetic basis of potato tuber dormancy, offering guidance for breeding.

## Core Ideas

- Wide variation for potato tuber dormancy length was observed (28–176 days at 18°C and 58–214 days at 4°C).
- Tuber dormancy varied significantly among genotypes, field locations, storage conditions, and market groups.
- Quantitative trait loci (QTLs) detected for tuber dormancy on seven chromosomes explained each up to 11.8% of phenotypic variation.
- Candidate genes near QTLs were connected to sugar, hormone, and cell cycle, implying complex regulatory pathways.
- Genomic‐estimated breeding values for dormancy length can help select parents and advance genotypes in breeding programs.

## Plain Language Summary

After harvesting, potatoes become dormant and do not sprout even under ideal conditions. Premature sprouting is a major cause of potato spoilage. Common sprout‐control methods are costly and may reduce potato quality or raise health and environmental concerns. From 2019 to 2022, 298 potato lines from the Texas A&M Potato Breeding Program were grown in Springlake and Dalhart, TX, and evaluated for dormancy length. Dormancy data were then combined with the genetic information of the potato lines to gain a better understanding of the underlying genetics of potato dormancy. Regions on seven potato chromosomes were identified for the length of tuber dormancy. Further study of genes in these regions can help understand the control mechanisms of potato dormancy. The potential of these potato lines to develop new, long‐dormancy varieties was evaluated. The knowledge gained from this study can help improve management practices in potato production and storage, and aid in the development of improved potato varieties with longer dormancy.

**Abbreviations**
AIC: Akaike information criterion
BLUE: best linear unbiased estimate
CDK: cyclin‐dependent protein kinase
CDC20: cell division control 20
CIPC: chlorpropham
CIS: cold‐induced sweetening
GA: gibberellin
GEBV: genomic‐estimated breeding value
GS: genomic selection
GWAS: genome‐wide association studies
HC: hydrogen cyanamide
MAS: marker‐assisted selection
MBS: marker‐based selection
PGSC: International Potato Genome Sequencing Consortium
QQ plot: quantile–quantile plot
QTL: quantitative trait locus
SNP: single nucleotide polymorphism

## 1 INTRODUCTION

Potato (*Solanum tuberosum* L.) is the third most important human food crop worldwide after rice and wheat and the number one vegetable crop. Cultivated for their edible tubers (underground modified stems rich in nutrients), potatoes are planted in approximately 160 countries, with a global total crop production of over 383 million metric tons in 2023 (Food and Agriculture Organization of the United Nations [FAO], 2025).

Upon harvesting, potato tubers enter a state of dormancy during which tubers cannot sprout even when kept under ideal conditions (Reust, 1986). During the dormancy period, there is a temporary arrest of the cell cycle of meristematic cells at the tuber eyes at the G1 phase (M. Campbell et al., 1996). Very long tuber dormancy periods can cause delays in seed certification due to difficulties in sprouting tubers in time for winter grow‐outs, thus affecting the timely supply of seed potatoes for planting by seed growers or commercial growers. The inability to break tuber dormancy can also lead to uneven plant emergence, ultimately resulting in poor crop establishment, reduced plant densities, and lower yields (Denny, 1926; Gelles et al., 2024; Siregar et al., 2021). Conversely, when the tuber dormancy period is too short, premature tuber sprouting can occur during storage or even at harvest time, particularly under conditions of high humidity and temperature. This premature tuber sprouting can lead to the breakdown of starch into simple sugars and an increase in respiration rate, glycoalkaloid accumulation, and water loss, resulting in reduced tuber quality or even total tuber spoilage (Börnke et al., 2007).

Worldwide, various storage methods are employed to preserve potato tubers for extended periods. For example, field storage methods such as delayed harvest, clamps, and pits can be used in cooler regions or when storage costs are of concern. Other options involve the use of environmentally controlled storage facilities, which, although expensive, allow for better control of storage temperature, humidity, and gases, and can help maintain high tuber quality (Pinhero et al., 2009). In commercial potato production, tuber sprouting is generally delayed by placing tubers in cold storage or by using sprout inhibitors. However, cold storage is expensive due to the required infrastructure and energy input. In addition, low storage temperatures may lead to the breakdown of starch into reducing sugars, a process known as cold‐induced sweetening (CIS) (Sonnewald, 2001), which results in the formation of brown‐colored pigments due to the Maillard reaction at high frying temperatures, affecting the quality of potato chips and French fries (Börnke et al., 2007). Thus, for tubers intended for the processing industry, storage temperature should be set between 7°C and 10°C to reduce the risk of CIS. Lower temperatures (3°C–7°C) can be used for tubers for the fresh market, for seed, or for processing cultivars with cold‐sweetening resistance (Olsen & Kleinkopf, 2020). Resistance to CIS has been a focus in breeding efforts, and some resistant potato varieties are available on the market. Tuber dormancy can also be extended by using chemicals that act as sprout inhibitors, such as chlorpropham (CIPC), maleic hydrazide, and essential oils, or dormancy enhancers like 1,4‐dimethylnaphthalene (Olsen & Kleinkopf, 2020). The disadvantages of chemical use include higher costs and concerns regarding biosafety. The use of CIPC has been banned by the European Union since 2020 due to concerns of potential health risks associated with CIPC and its metabolite, 3‐chloroaniline (Visse‐Mansiaux et al., 2021). Thus, cultivars with long dormancy periods are desired to extend the lifespan of potato tubers, prevent spoilage, conserve energy, and reduce costs and health risks associated with the cold storage of tubers and the application of sprout inhibitors. Understanding natural variation in the duration of tuber dormancy in advanced breeding clones from breeding programs, as well as the underlying genetic basis, is of great interest in guiding the development of new potato cultivars with long dormancy periods.

The length of tuber dormancy varies among different potato genotypes, ranging from less than 1 month to over 9 months, and is influenced by growing conditions and management practices (Suttle, 2007). High temperature during the growing season is one of the major environmental factors affecting tuber dormancy since it can delay the onset of tuber dormancy, shorten the dormancy period, and sometimes even cause dormancy release prior to harvesting (heat sprouting) (Gautam, Pandey, et al., 2024; Levy & Veilleux, 2007; G. Zhang et al., 2021). Other factors that affect tuber dormancy include nitrogen application during growth (H. Zhang et al., 2022), storage temperatures (Draie & Al‐Ali, 2021; Espen et al., 1999; Murigi et al., 2021), disease such as psyllid yellow (Braidwood, 2024; Wenninger & Rashed, 2024), and herbicide drift (Hatterman‐Valenti, 2014).

Research into understanding the genetic control of potato tuber dormancy has been challenging, as most cultivated potatoes are autotetraploid (2*n* = 4*x* = 48) with tetrasomic inheritance (Muthoni et al., 2015). Previous studies aimed at understanding the genetics of potato tuber dormancy have primarily utilized diploidized *S. tuberosum* and its diploid wild relatives. Quantitative trait loci (QTLs) associated with the length of tuber dormancy were found on 10 out of the 12 chromosomes, explaining phenotypic variation ranging from 1.5% to 31% each (Bisognin et al., 2018; Freyre et al., 1994; Li et al., 2018; Sharma et al., 2021; Van den Berg et al., 1996). In a diploid F1 population from a *S. tuberosum* × *Solanum chacoense* hybrid and a *Solanum phureja* clone, six QTLs were detected to affect tuber dormancy, with one on each of chromosomes 2, 3, 4, 5, 7, and 8. The QTL on chromosome 7 showed the strongest effect on tuber dormancy, with nine markers explaining 12.4%–20.4% of the phenotypic variation (Freyre et al., 1994). In a separate QTL analysis using populations derived from reciprocal backcrosses between *S. tuberosum* and *Solanum berthaultii*, no QTLs involved in tuber dormancy were detected on chromosome 7. Instead, a QTL on chromosome 2 had the largest effect (*R* ^2^ = 31%) on tuber dormancy, which suggests that the QTL on chromosome 7 detected previously may have come from *S. chacoense* (Van den Berg et al., 1996). In the study by Bisognin et al. (2018), a diploid segregating population with *S. tuberosum*, *S. chacoense*, *S. berthaultii*, and *S. phureja* background was used. Eight QTLs (three on chromosome 5 and one on each of chromosomes 2, 3, 7, 9, and 11) were identified to affect potato tuber dormancy with the single nucleotide polymorphism (SNP) marker c2_51194 located at the QTL position of chromosome 5 explaining the largest phenotypic variance (*R* ^2^ = 16.3%) (Bisognin et al., 2018).

**Core Ideas**

- Wide variation for potato tuber dormancy length was observed (28–176 days at 18°C and 58–214 days at 4°C).
- Tuber dormancy varied significantly among genotypes, field locations, storage conditions, and market groups.
- Quantitative trait loci (QTLs) detected for tuber dormancy on seven chromosomes explained each up to 11.8% of phenotypic variation.
- Candidate genes near QTLs were connected to sugar, hormone, and cell cycle, implying complex regulatory pathways.
- Genomic‐estimated breeding values for dormancy length can help select parents and advance genotypes in breeding programs.

Most candidate genes found within the genomic regions of dormancy‐related QTLs discovered in diploid‐based studies were related to the regulation of phytohormones and carbohydrates. These findings have provided valuable insights into the location and potential functions of the genes and genomic regions involved in tuber dormancy control. However, the translation of diploid‐based genetic findings and incorporation of dormancy traits from diploid relatives into a tetraploid potato breeding remain challenging. Difficulties arise from belonging to different genetic pools, cross‐incompatibilities associated with different ploidy levels or inter‐specific barriers, infertility, as well as linkage drag bringing non‐adapted traits into cultivated tetraploid potatoes. Such limitations underscore the need for research involving elite tetraploid potato genotypes to better understand the genetics of tuber dormancy in cultivated potatoes.

In recent years, great progress has been made to better understand the genetics of cultivated potato, including advances in sequencing, development of molecular marker platforms, as well as improvements in analytical tools so that research and breeding of autopolyploid crops such as potato can benefit from the rapid advances in genomics (Endelman, 2023a; Endelman et al., 2024; Hamilton et al., 2011; Hoopes et al., 2022; Rosyara et al., 2016; The Potato Genome Sequencing Consortium, 2011).

Major approaches to understanding the genetic basis of traits include linkage mapping and association mapping. While linkage mapping requires the use of mapping populations, association mapping can be applied to a more diverse panel, allowing for the use of historical genomic recombination and variations (Kushwaha et al., 2017). One common association mapping method is genome‐wide association studies (GWAS), which identify genomic variants statistically associated with a particular trait and involve screening the entire genomes of a large number of individuals (Hutter, 2020). In tetraploid potatoes, GWAS has been used for studying traits such as tuber morphology (Pandey et al., 2022), abiotic stress tolerance (Iribar et al., 2025; R. Campbell et al., 2023; Gautam, Pandey, et al., 2024), tuber glycoalkaloid biosynthesis (Lian et al., 2025; Zhou et al., 2025), tuber nutritional quality (Gong et al., 2025; Pandey, Gautam, et al., 2023; Pandey, Thompson, et al., 2023; Rasheed et al., 2025), and disease and pest resistance (Anglin et al., 2024; Leuenberger et al., 2025; Okiro et al., 2025; Yuan et al., 2020).

The improved understanding of potato genetics and advancement of analytical tools have also enabled the utilization of genomic selection (GS) for tetraploid potato breeding. GS utilizes all the markers covering the entire genome of a phenotyped and genotyped population to predict genomic‐estimated breeding values (GEBVs) of a population that is only genotyped but not phenotyped. In potato breeding, GS has been explored for implementation in the selection of parents and advancement of breeding genotypes based on traits such as disease resistance (Enciso‐Rodriguez et al., 2018), abiotic stress response (Gautam, Pandey, et al., 2024), yield (Habyarimana et al., 2017), tuber morphology (Pandey et al., 2022), and chipping quality (Pandey, Scheuring, et al., 2023).

Considering the need to better understand phenotypic variation for tuber dormancy and its genetics in tetraploid potatoes, in the context of improving long‐term preservation of tubers and spoilage reduction, and taking advantage of statistical genomics analytical tools developed for complex polyploid species, we conducted studies with the following objectives: (i) explore phenotypic variation in tuber dormancy length among advanced potato genotypes, (ii) understand the genetic basis of tuber dormancy, and (iii) obtain GEBVs for tuber dormancy using GS.

## 2 MATERIALS AND METHODS

### 2.1 Plant materials

A panel of 216 tetraploid potato genotypes (panel one) was grown under field conditions in Springlake and Dalhart, TX, from 2019 to 2021 (Pandey et al., 2022). An additional panel (panel two) of 82 new genotypes, along with seven previously tested check genotypes, was planted in both field locations in 2022 (Table S1). Overall, panels one and two, composed of 298 unique potato genotypes, belonging to different market groups: 89 russets, 52 chip‐processing (referred to as “chippers” in this manuscript), 90 reds, 43 yellows, and 24 purples, were grown and evaluated for tuber dormancy length from 2019 to 2022. The panels included released varieties and advanced tetraploid potato genotypes from the Texas A&M Potato Breeding Program, as well as commercial reference varieties for the different market groups. Panel one (216 genotypes), included in replicated trials from 2019 to 2021 at two Texas locations, was used for GWAS analysis, while both panels were used for GS.

### 2.2 Genotyping

DNA extraction and genotyping of panel one (216 potato genotypes) were performed by Pandey et al. (2021) using the Infinium Illumina 22K V3 Potato SNP Array on the Illumina iScan (Illumina Inc.). An additional set (panel two) (82 new genotypes) was genotyped in 2022. Young leaf samples (50–80 mg) were used for DNA extraction using the DNeasy Plant Pro Kit (Qiagen). Genotyping for the newly added genotypes was conducted using the Infinium Illumina 31K V4 Potato SNP Array. Genotype calling was performed using the R package fitPoly (Voorrips et al., 2011; Zych et al., 2019), and the datasets were merged using the merge_impute function of the R package polyBreedR (Endelman, 2023b). The SNP genotype data were filtered by excluding low quality and monomorphic SNPs, as well as loci with more than 10% missing data (Pandey et al., 2021). A total of 13,970 markers were included for genomic analysis.

### 2.3 Field experiments and phenotyping

Field trials were conducted in Dalhart, TX (35°58′15″ N, 102°44′36″ W, altitude 1214 m) in 2019, 2020, and 2022, and in Springlake, TX (34°7′4.07″ N, 102°20′57.63″ W, altitude 1122 m) in 2020, 2021, and 2022. The daily minimum and maximum temperatures were obtained from the National Oceanic and Atmospheric Administration's weather stations of Littlefield (near the Springlake field location) and Dalhart Municipal Airport (near the Dalhart field location). At both locations, each genotype was planted in a 12‐hill plot with two replications using a randomized complete block design. In Dalhart, planting occurred in early May, and tubers were harvested in September (Table S2). The tuber seed pieces were planted 25 cm apart, 15 cm deep, and in rows 71 cm apart. In Springlake, planting was carried out in late March, while harvesting was done in mid‐July. Seed tubers were planted 23 cm apart with row spacing of 91 cm, and at a depth of 15 cm. At both locations, standard cultural practices for the area were used (Vales et al., 2023). Chemical and mechanical vine kill was performed two to three weeks before harvesting.

Upon harvesting from each location, four tubers (between 113 and 170 g/tuber) per replicate per genotype were placed in mesh bags and kept at room temperature (18°C, 60% relative humidity, dark) in College Station, TX. Another four tubers were placed in mesh bags and stored in milk crates (33 cm × 33 cm × 28 cm) in cold storage (4°C, 90% relative humidity, dark) in Lubbock, TX. Two replicates per genotype were included for the evaluation. Visual assessments for dormancy break were conducted weekly until the last genotype broke dormancy. Dormancy break was declared when at least three of the four tubers in a replicate were peeping, which was defined as when a sprout of at least 2 mm is seen on the tuber (Reust, 1986; Van Ittersum & Scholte, 1992). The length of tuber dormancy was calculated as the number of days from vine kill until dormancy break. A total of 11 environments were included for the assessment of tuber dormancy (Table S2). Dormancy evaluation in cold storage for tubers produced in Dalhart in 2019 continued into 2020 and was interrupted due to COVID‐19 lockdown, resulting in skewed data that were unsuitable for inclusion in the analysis.

### 2.4 Statistical analysis

The effects of genotypes, locations, years, storage conditions, and interactions were based on panel one (216 genotypes tested in replicated trials at two Texas locations from 2019 to 2021) and were assessed via analysis of variance using mixed models as *Y_ijklmn_* = *μ* + *G_i_* + *L_j_* + *S_k_* + *M_l_* + (*G* × *L*)*_ij_* + (*G* × *S*)*_jk_* + *u_m_* + *r_n_* + (*G* × *u*)*_im_* + (*M* × *u*)*_lm_* + *ε_ijklmn_*. *Y_ijklmn_* represents the dormancy observation for the *i*th genotype from the *j*th location, *k*th storage condition, belonging to the *l*th market group, grown in the *m*th year, and from the *n*th replication. *μ* is the overall mean, and *ε_ijklmn_* is the residual. Genotypes (*G_i_*), locations (*L_j_*), storage conditions (*S_k_*), and interactions were considered fixed effects, whereas years (*u_m_*), replications (*r_n_*), and their interactions were considered random. The analysis of variance was performed using JMP Pro 17 (JMP Statistical Discovery LLC). The effect of location (Dalhart vs. Springlake) on the length of tuber dormancy was determined using data from 2020. To assess the effect of the year on tuber dormancy length, data from 2019 to 2021 were used, and each location‐storage condition combination was analyzed separately. Data from 2020 and 2021 were utilized to evaluate the effect of storage conditions (room temperature vs. cold storage). The length of tuber dormancy was also compared among market groups (fixed effects) under each storage condition.

Descriptive statistics were calculated using JMP Pro 17. Best linear unbiased estimates (BLUEs) for each genotype under individual environments were estimated using the software package META‐R (Alvarado et al., 2020) as *Y_ik_* = *μ* + *R_i_* + *G_k_* + *ε_ik_*. *Y_ik_* is the trait of interest, *μ* is the mean effect, *R_i_* is the effect of the *i*th replicate, *G_k_* is the effect of the *k*th genotype, and *ε_ik_* is the error associated with the *i*th replication and the *k*th genotype. Genotype was considered a fixed effect, while all other terms were considered random effects. Normality tests were conducted using the Shapiro–Wilk *W*‐test (Shapiro & Wilk, 1965) in JMP Pro 17. Variance components were estimated using the R package StageWise (Endelman, 2023a), which employs ASReml‐R (VSN International Ltd.) to fit linear mixed models using residual maximum likelihood (REML). Broad‐sense heritability (*H* ^2^) was calculated on a plot basis from the variance components for genetic (*V*_g_) and residual (*V*_e_) variances as *V*_g_/(*V*_g_ + *V*_e_). Narrow‐sense heritability (*h* ^2^) was calculated from the variance components as *V*_a_/(*V*_a_+*V*_d_+*V*_i_+*V*_e_) using additive (*V*_a_), dominance (*V*_d_), interaction (*V*_i_), and residual (*V*_e_) variances.

### 2.5 Assessment of linkage disequilibrium and GWAS

GWAS were performed for the length of tuber dormancy with 13,970 SNPs using the R package GWASpoly Version 2 (https://github.com/jendelman/GWASpoly) based on Rosyara et al. (2016) for individual environments (year–location–storage condition combinations) and for each location–storage condition combination. Genotypic and phenotypic data of 216 genotypes (panel one) collected from 2019 to 2021 were included in the analysis. GWAS was conducted based on the model equation **y** = **Xβ** + **ZSτ** + **ZQv** + **Zu** + **ε**, where **y** is a *w* × 1 vector of observed phenotypes and *w* is the total number of observations, **X** is the incidence matrix used to model fixed effects and **β** is a *p* × 1 vector of fixed effects with *p* as the number of fixed effects, **Z** is a 2 × *n* incidence matrix that maps genotypes to the observations for a population of size *n*; the *d* × 1 vector **τ** represents SNP effect with the *n* × *d* incidence matrix **S** and the dimension *d*, **Q** is a *n* × q incidence matrix for a population of size *n*, **v** is a *q* × 1 vector of effects for *q* subpopulations, **u** is an *n* × 1 vector of polygenic effects with Var[**u**] = *σ_g_* ^2^ **K** where **K** is the kinship matrix, and **ε** is a *w* × 1 vector of residuals with Var[**ε**] = I*σ_e_* ^2^. Environments, location–storage condition combinations, and days from planting to vine kill were included as fixed effects. The *K* model (Yu et al., 2006) (a random polygenic effect) was used to control for population structure. For computational efficiency (to account for the polygenic model and the complexity of the analysis), the LOCO (leave‐one‐chromosome‐out) method (Yang et al., 2014), in which a different covariance matrix is calculated for each chromosome based on the markers from all other chromosomes, was implemented. The threshold for a genome‐wide false‐positive rate of 5% was established using the “M.eff” method, which divides the significance level by the effective number of markers while accounting for the correlation between markers (Moskvina & Schmidt, 2008). A quantile–quantile (QQ) plot was used to examine the inflation of the −log10(*p*) of the observed versus expected values under the null hypothesis. The GWASpoly package was also used to calculate the percentage of phenotypic variance explained by each significant SNP at the peak of each QTL. Manhattan plots were generated using the R package CMplot (Yin, 2024). Linkage disequilibrium (LD) was evaluated. The LD.plot function in GWASpoly was used to estimate and visualize LD for all SNPs, based on pairwise correlation coefficients calculated using allele frequencies (Hill & Robertson, 1968). The R package scam was used to fit a monotone decreasing, convex spline. The extent of LD in the panel was used to determine the appropriate window size to filter the most significant markers. The potato reference genome of *S. tuberosum* group Phureja DM1‐3 PGSC v4.03 (where PGSC is International Potato Genome Sequencing Consortium; Sharma et al., 2013) from SpudDB (http://spuddb.uga.edu/) was searched to explore putative candidate genes in a 2‐Mb flanking genomic region of each peak SNP using a custom R script (Supporting Information S1). The gene annotation data was filtered to retain only “gene” features, and the midpoint position for each gene was calculated. Absolute distance between each gene's midpoint and the peak SNPs were computed and all annotated genes with midpoints within 2 Mb from the peak SNPs were returned as candidates.

### 2.6 Genomic prediction for potato tuber dormancy

GEBVs were calculated for 292 potato genotypes (most members of panel one and two combined) that had both genotype and phenotype data via a two‐stage approach using StageWise (Endelman, 2023a). ASReml‐R (VSN International Ltd.) was used to fit the linear mixed model. GEBVs were computed with allele dosage information. In Stage 1, BLUEs of genotypic value were calculated as BLUE [*g_ij_*] = *μ_j_* + ∑*β_k_w_ik_* + a_i_ + *d_i_* + *s_ij_* + *ε_ij_*, where *g_ij_* is the genotypic value for genotype *i* in environment *j*, *μ_j_* is the fixed effect for environment *j*, *w_ik_* is the “centered” allele dosage for genotype *i* at marker *k*, *β_k_* is the fixed effect for marker *k*, a_i_ is the vector of additive values, *d_i_* is the independent and identically distributed residual genetic values used to capture nonadditive effects, *s_ij_* is the random effect that follows a multivariate normal distribution with no free variance parameters (Damesa et al., 2017), and *ε_ij_* is the model residual and it represents the genotype × year interaction.

In stage 2, genomic best linear unbiased predictors (GBLUP) were computed for a generic random vector u using the standard method as GBLUP[**u**] = û = **CPy**, where û is the predicted value of u, **C **= cov[**u**, **y**], and **P **= **V** ^−1^ − **V** ^−1^ **X**(**X**′**V** ^−1^ **X)** ^−1^ **X**′**V** ^−1^. **V** represents the variance‐covariance matrix of the response variable while **X** stands for the incidence matrix for fixed effects (Searle et al., 1992). The reliability of û_i_ is represented by the squared correlation with the true value, which equals $r_{i}^{2}=\frac{var[{\hat{u}}_{i}]}{var[u_{i}]}\;$, and var[û] = **CPC**’. The genomic additive matrix **G** is estimated as $G=\frac{WW^{T}}{\emptyset \sum p_{k}q_{k}}$, where **W** is the centered matrix of allele dosages (*n* individuals × *m* biallelic markers with frequencies *p* = 1 − *q*), and ∅ is ploidy. Different models to partition the proportion of variance excluding the environment effect were compared, and the model was selected based on a lower Akaike information criterion (AIC). Prediction reliability (*r* ^2^), which denotes the squared correlation between the true and predicted values, was computed. GEBVs were standardized as *z*‐scores using JMP Pro 17.

### 2.7 Cross‐validation

Three types of cross‐validation were performed: year‐wise cross‐validation, cross‐validation within a market group (russets), and a random‐sampling cross‐validation scheme. For the year‐wise cross‐validation, genotypes evaluated from 2019 to 2021 were considered the training population, while the newly added genotypes for evaluation in 2022 were considered the test set. Phenotype data from 2022 (which have identifiers “22” at the beginning of the environment code) were masked, and the reliabilities of phenotypic prediction were obtained using StageWise (Endelman, 2023a).

To illustrate cross‐validation within a market group, we focused on russets, as this group had the largest number of individuals, and employed both targeted and untargeted approaches. For the targeted approach, both the training population and the test set were russets (genetically close). The training population consisted of 77 russet genotypes evaluated from 2019 to 2021, whereas the test involved 12 russet genotypes newly added for dormancy evaluations in 2022. For the untargeted approach, the training population consisted of all members of panel one (216 individuals from all market groups that show wide variation for the trait of interest), and the test set comprised the 12 new russet genotypes evaluated in 2022. Reliabilities of phenotypic prediction using StageWise (Endelman, 2023a) for marker‐based selection (MBS; 2022 test set phenotypes masked) and marker‐assisted selection (MAS; both genotypes and phenotypes used for the 2022 test set) for the untargeted approach were obtained and compared with those obtained using a targeted approach (both training and test set belonging to the russet group).

To further evaluate the predictive performance of the genomic prediction model, a fivefold cross‐validation scheme was employed. The entire dataset was randomly partitioned into five equal subsets (folds). In each iteration, one fold served as the test set, while the remaining four folds were used as the training set. For each fold, phenotypic values for the test set were masked, and the training set was used to fit the genomic prediction model. The model accounted for additive genetic effects, dominance effects, and residuals. The reliability of phenotypic prediction for the test set was calculated for each fold. The process was repeated five times to ensure robustness. The mean reliability across folds was reported along with its standard deviation (SD) to capture variability in model performance.

### 2.8 Correlations between tuber dormancy and other traits

When the first panel was grown at both locations (Dalhart and Springlake, TX) from 2019 to 2020, tubers harvested and stored at room temperature were also phenotyped for many agronomical and quality traits. The phenotyping procedures and resulting data for tuber amino acid and mineral contents, tuber defects, and yield have been previously described in detail by Pandey, Thompson, et al. (2023), Pandey, Gautam, et al. (2023), and Gautam, Pandey, et al. (2024). Specific gravity was measured using the water displacement method and calculated as the ratio of the difference between the tuber's weight in air and its weight in water to its weight in air.

The correlation between tuber dormancy length and the agronomical and quality traits was evaluated, and the Spearman's rank correlation coefficient (*ρ*) and the significance probability (*p*‐value) were calculated for each pair of traits using JMP Pro 17 (JMP Statistical Discovery LLC).

## 3 RESULTS

### 3.1 Phenotypic variation for the length of tuber dormancy

The analysis of variance was performed for tuber dormancy length based on tuber dormancy data from a panel of 216 tetraploid potato genotypes grown in Dalhart and Springlake, TX, from 2019 to 2021 and evaluated both at room temperature and in cold storage. Significant differences between genotypes (*p* < 0.0001) and genotype–environment interaction (*p* < 0.0001) were found for the length of tuber dormancy (Table 1; Table S3), where different environments represent different year–location–storage condition combinations. Year was not shown to have a significant effect on tuber dormancy length (*p* = 0.5100) (Table S3).

**TABLE 1:** Mean, range (min. and max.), and broad‐sense heritability (*H* ^2^) of the tuber dormancy period length of 216 potato genotypes evaluated in two locations (Dalhart and Springlake, TX) over 3 years (2019–2021) under two storage conditions (18°C and 4°C).

Environment	Year	Location	Storage temperature	Mean	Min.	Max.	*H* ^2^
			°C	Days			
19DAL‐R	2019	Dalhart	18	80.9e	56	140	0.86
20DAL‐R	2020	Dalhart	18	86.0d	48	144	0.86
20DAL‐C	2020	Dalhart	4	146.0a	81	214	0.81
20SPR‐R	2020	Springlake	18	68.1f	33	121	0.82
20SPR‐C	2020	Springlake	4	117.6c	81	178	0.64
21SPR‐R	2021	Springlake	18	84.3d	42	168	0.83
21SPR‐C	2021	Springlake	4	127.9b	80	195	0.77

*Note*: Means not sharing a letter were significantly different at the 5% level of significance according to the Wilcoxon rank‐sum test. The numbers 19, 20, and 21 in column 1 (environment) denote years 2019, 2020, and 2021; DAL and SPR denote Dalhart and Springlake; and C and R denote cold storage and room temperature.

**FIGURE 1:**
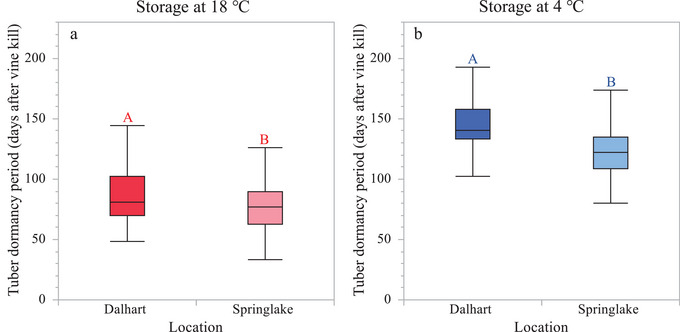
Boxplots comparing tuber dormancy lengths between locations (Dalhart and Springlake, TX) using Wilcoxon two sample test based on evaluation of 216 potato genotypes in 3 years (2020–2021) and kept under two different storage conditions (18°C and 4°C). Each box shows the interquartile range (IQR) from 25th to 75th percentile. The horizontal line within the box represents the median, and whiskers extend to the smallest and largest data points within 1.5 times the IQR from the quartiles. (a) Dormancy periods of potato tubers stored at room temperature (18°C). (b) Dormancy periods of potato tubers kept in cold storage (4°C).

Location was found to have a significant effect on tuber dormancy length (*p* = 0.0002), with tubers harvested from Dalhart having significantly longer dormancy periods than tubers grown in Springlake, both at room temperature and in cold storage (Figure 1; Table S3). At room temperature, tubers from Dalhart had a mean dormancy length of 85.0 days, with dormancy periods ranging from 48 to 144 days; while tubers from Springlake had dormancy periods between 33 and 168 days with a mean of 77.5 days (Table 2). Cold storage significantly extended the dormancy period. When placed in cold storage, tubers from Dalhart remained dormant between 81 and 214 days, with an average of 146.0 days, while tubers from Springlake had dormancy periods ranging from 80 to 195 days, with a mean of 123.7 days (Table 2). For example, in 2020, Russet Burbank (the number one russet variety used for French fries in the United States, known for having long dormancy) tubers harvested from Dalhart remained dormant for 123 days at room temperature, while tubers from Springlake sprouted within 88 days. Similarly, in cold storage, Russet Burbank tubers from Dalhart peeped 193 days after vine kill, while tubers from Springlake broke dormancy 151 days after vine kill (Table S4). Yukon Gold (a popular yellow flesh cultivar used mainly for the fresh market) displayed similar patterns in 2022, with longer dormancy observed in Dalhart tubers (85 days at room temperature and 120 days in cold storage) than those from Springlake (50 days at room temperature and 110 days in cold storage) (Table S4).

**TABLE 2:** Mean, range (min. and max.), and narrow‐sense heritability (*h* ^2^) of the tuber dormancy period length of 216 potato genotypes evaluated in two locations (Dalhart and Springlake, TX) over 3 years (2019–2021) under two storage conditions (18°C and 4°C).

Environment	Location	Storage temperature	Mean	Min.	Max.	*h* ^2^
		°C	Days			
DAL_R	Dalhart	18	85.0c	48	144	0.67
DAL_C	Dalhart	4	146.0a	81	214	0.80
SPR_R	Springlake	18	77.5d	33	168	0.46
SPR_C	Springlake	4	123.7b	80	195	0.46

*Note*: Means not sharing a letter were significantly different at the 5% level of significance according to the Wilcoxon rank‐sum test. DAL and SPR in column 1 (environment) denote Dalhart and Springlake, and C and R denote cold storage and room temperature.

**FIGURE 2:**
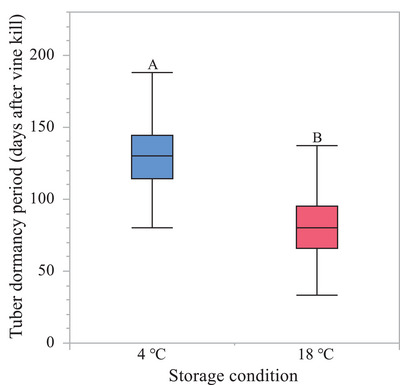
Boxplot comparing tuber dormancy periods between tubers kept under different storage conditions (18°C and 4°C) based on evaluation of 216 potato genotypes in two locations (Dalhart and Springlake, TX) over 3 years (2019–2021). Each box shows the interquartile range (IQR) from 25th to 75th percentile. The horizontal line within the box represents the median, and whiskers extend to the smallest and largest data points within 1.5 times the IQR from the quartiles.

Storage conditions had a significant effect on the length of the tuber dormancy period (*p* < 0.0001), with tubers stored in cold storage remaining dormant for significantly longer than tubers kept at room temperature (Figure 2; Table S3). Tuber dormancy length ranged from 33 to 168 days at room temperature, with a mean of 81.3 days. In cold storage, tuber dormancy periods varied from 80 to 214 days, with a mean of 130.8 days (Figure 2). In the case of the chippers variety Atlantic, tubers kept in cold storage remained dormant for more than 40 days longer than those stored at room temperature (115 days in cold storage compared to approximately 70 days at room temperature, as observed in 2020 using tubers from Springlake) (Table S4). Interactions between genotype and environment, genotype and location, genotype and storage conditions, genotype and year, and between genotype and the combination of location and storage were found to be significant (all with *p* values below 0.0001) (Table S3).

**FIGURE 3:**
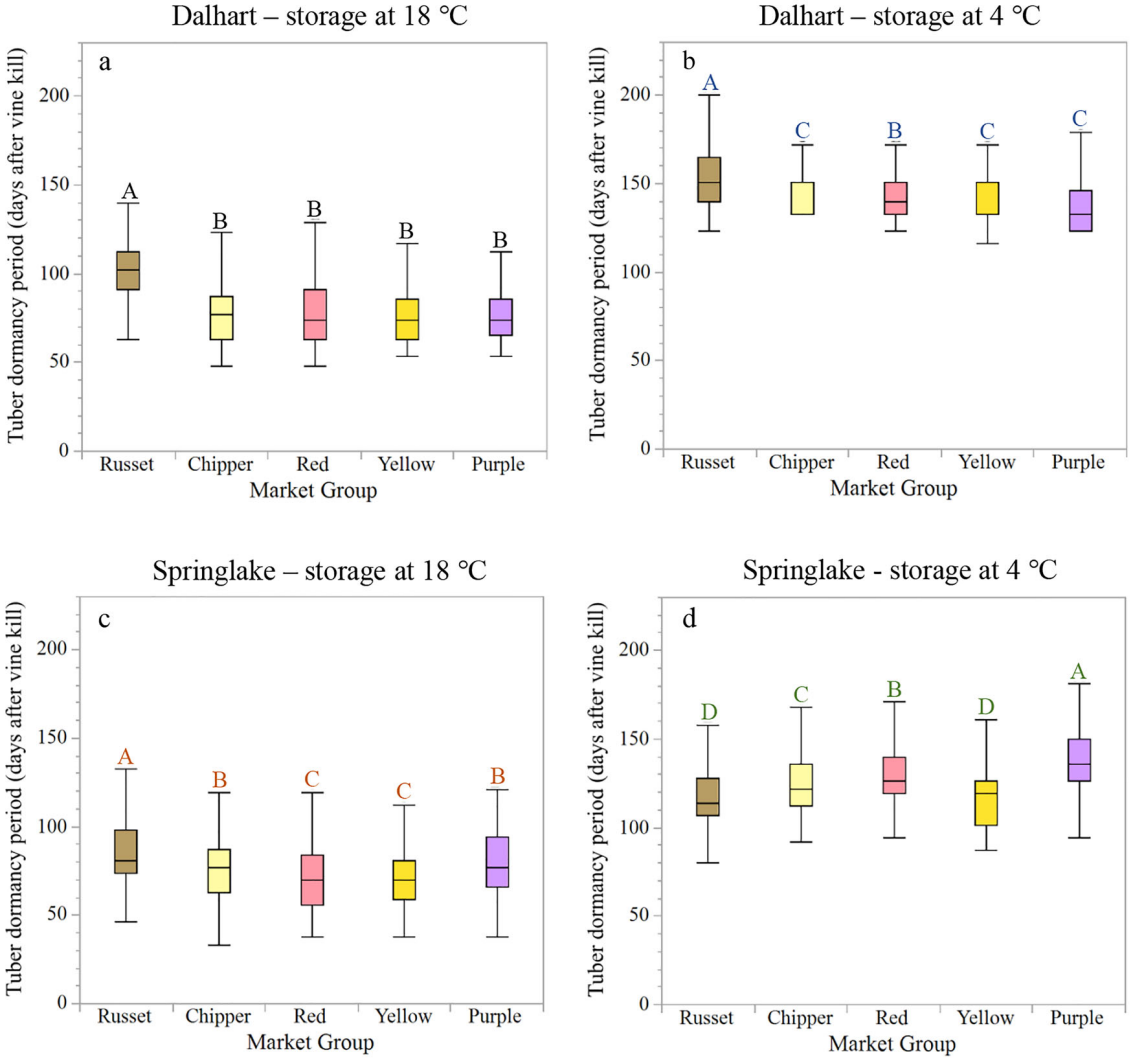
Boxplots comparing tuber dormancy periods among market groups (chippers, purples, reds, russets, and yellows) based on evaluation of 216 potato genotypes in two locations (Dalhart and Springlake, TX) over 3 years (2019–2021) and kept under two storage conditions (room temperature at 18°C and cold storage at 4°C). Each box shows the interquartile range (IQR) from 25th to 75th percentile. The horizontal line within the box represents the median, and whiskers extend to the smallest and largest data points within 1.5 times the IQR from the quartiles. (a) From Dalhart and stored at room temperature (18°C), (b) from Dalhart and kept in cold storage (4°C), (c) from Springlake and stored at room temperature (18°C), and (d) from Springlake and kept in cold storage (4°C).

Since there was no significant difference in the length of tuber dormancy between years, tuber dormancy data from different years were combined when assessing the effect of different location–storage condition combinations. The combination of locations and storage conditions had a significant effect on the length of tuber dormancy (*p* < 0.0001), with potato tubers harvested from Dalhart kept in cold storage (4°C) having, on average, the longest tuber dormancy period of 146.0 days (Table 2; Table S3). The dormancy period varied significantly among market groups when tubers from both field locations were evaluated under both storage conditions (*p* < 0.0001) (Figure 3). Russet potatoes had significantly longer tuber dormancy periods under most location–storage combinations, and yellows consistently had the shortest tuber dormancy periods. For example, when harvested from Dalhart and stored at room temperature, russet potatoes had a mean dormancy length of 99.4 days, compared to 76.7 and 76.2 days for chippers and yellows, respectively (Table 3).

**TABLE 3:** Mean and range (min. and max.) of the tuber dormancy length of the five market groups (73 russets, 31 chippers, 64 reds, 30 yellows, and 18 purples) under each location‐storage condition combination when 216 potato genotypes were evaluated in two locations (Dalhart and Springlake, TX) over 3 years (2019–2021) under two storage conditions (18°C and 4°C).

Location–storage	Market group	Mean	Min.	Max.
			Days	
DAL_R	Russet	99.4a	56	144
	Chippers	76.7b	48	129
	Red	76.7b	48	129
	Yellow	76.2b	53	117
	Purple	80.3b	53	137
DAL_C	Russet	154.5a	123	207
	Chippers	135.1c	81	193
	Red	146.6b	88	214
	Yellow	137.0c	88	214
	Purple	136.9c	88	200
SPR_R	Russet	85.1a	46	147
	Chippers	76.0b	33	126
	Red	70.8c	38	119
	Yellow	69.8c	38	119
	Purple	82.3b	38	168
SPR_C	Russet	117.1d	80	183
	Chippers	125.6c	92	174
	Red	130.2b	94	195
	Yellow	116.7d	87	161
	Purple	138.4a	94	181

*Note*: Means not sharing a letter (within location‐storage) were significantly different at the 5% level of significance according to the Wilcoxon rank‐sum test. DAL and SPR in column 1 (location–storage) denote Dalhart and Springlake, and C and R denote cold storage and room temperature.

Variance component analysis was performed both when all seven environments were combined, and for the two storage conditions separately. When all environments were combined, storage condition explained the highest proportion of phenotypic variance (71.6%). However, when two storage conditions were analyzed separately, genotype explained the highest proportion of phenotypic variance (59.7%) at room temperature, while in cold storage, genotype (22.6%), location (27.7%), and genotype × location interaction (20.0%) each explained a significant proportion of the phenotypic variance (Table S5).

Broad‐sense heritability ranged from 0.64 to 0.86 among different environments, and narrow‐sense heritability varied between 0.46 and 0.80 (Tables 1 and 2). The moderate to high heritability for tuber dormancy indicates that a large proportion of the phenotypic variation can be explained by genotypic effect, thus it is feasible to explore the genetic factors underlying tuber dormancy.

**FIGURE 4:**
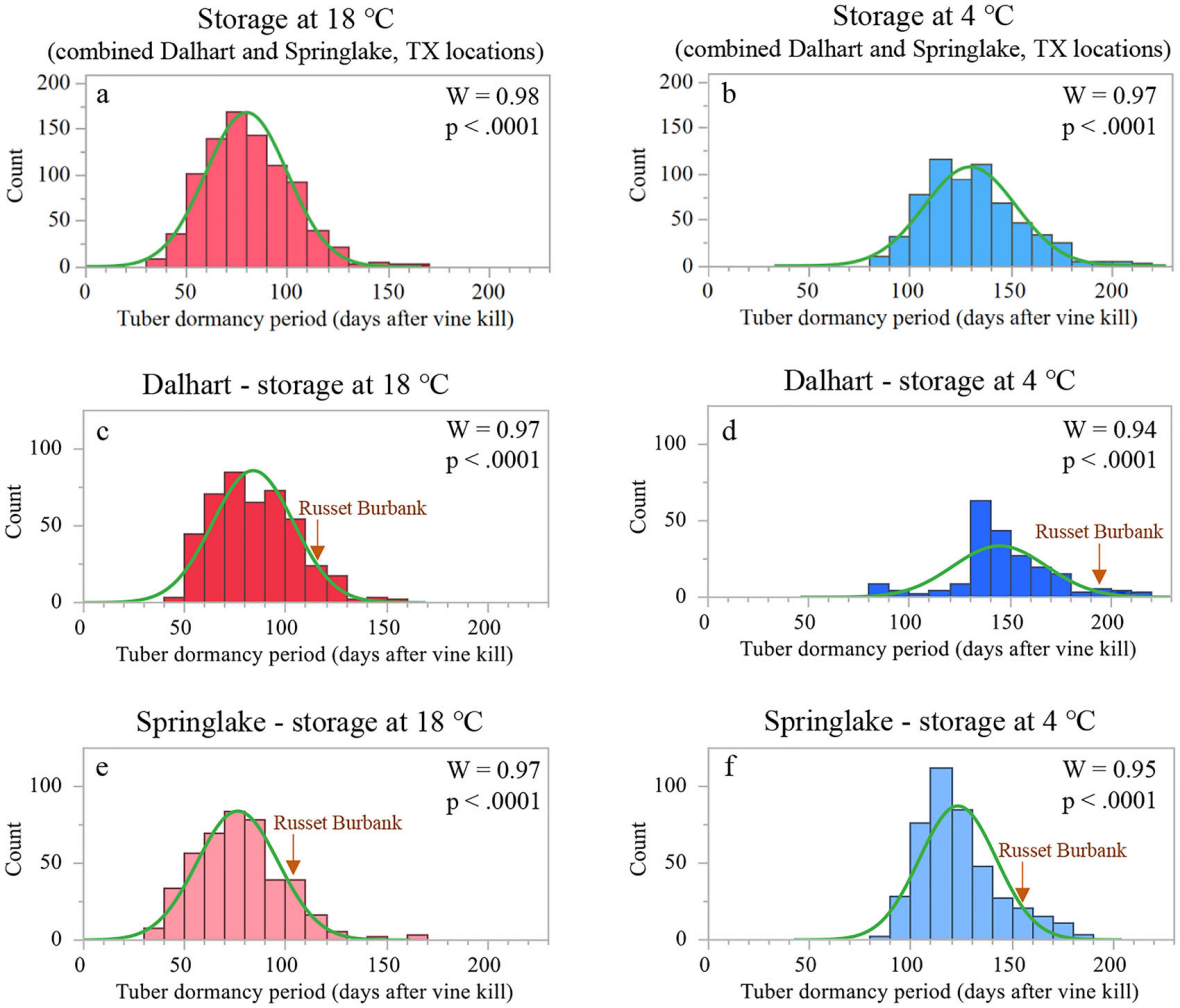
Distribution of the tuber dormancy period based on storage under different conditions (room temperature at 18°C and cold storage at 4°C) of 216 potato genotypes in seven environments involving two locations (Dalhart and Springlake, TX) and 3 years (2019–2021). The test statistic (*W*) and associated *p*‐value from a Shapiro–Wilk normality test are shown. The arrows indicate the dormancy period length of Russet Burbank (the most widely consumed potato in the United States) under each location‐storage condition combination. (a) Stored at room temperature (18°C) (combined Dalhart and Springlake, TX, locations), (b) kept in cold storage (4°C) (combined Dalhart and Springlake, TX, locations), (c) from Dalhart, TX, and stored at room temperature (18°C), (d) from Dalhart, TX, and kept in cold storage (4°C), (e) from Springlake, TX, and stored at room temperature (18°C), and (f) from Springlake, TX, and kept in cold storage (4°C).

When evaluated at room temperature, the potato genotype NDTX059828‐2W (chipper) demonstrated the shortest tuber dormancy at 33 days when harvested from Springlake, while TX12474‐1P/R (purplish skin red flesh) had the longest tuber dormancy period at 168 days after vine kill when harvested from Springlake (Table S4). When tuber dormancy was assessed in cold storage, TX6‐1216‐1Ru (russet) out of Springlake had the shortest dormancy of 80 days, while COTX10226‐1Wpe/Y (white skin with purple eyes and yellow flesh) harvested from Dalhart stayed dormant for the longest, reaching 214 days (Table S4). The phenotypic distributions for tuber dormancy length for each storage condition (room temperature and cold storage), location (Springlake and Dalhart), combination of storage and locations, and under individual environments (year–location–storage condition combination) (Figure S1) differed significantly from normal distribution according to the Shapiro–Wilk test (*p* < 0.05) (Figure 4; Figure S1).

In 2022, a second panel of 82 genotypes was evaluated using tubers from both field locations (Springlake and Dalhart), and the tubers were stored at room temperature and under cold conditions. At room temperature, ATX15097‐1Ru (russet) had the shortest dormancy of 28 days out of Springlake, while TX17805‐12R/R (red skin red flesh) remained dormant for the longest, up to 176 days when harvested from Dalhart (Table S4). While in cold storage, the tuber dormancy length of the genotypes evaluated ranged from 58 days for COTX17288‐3 W (chipper) when grown in Springlake to 193 days for TX19411‐4 W (chipper) when coming from Dalhart (Table S4). The phenotypic distributions in all environments (year–location–storage condition combinations), except for 22DAL‐C (2022–Dalhart–cold storage), were significantly different from the normal distribution (Figure S1).

### 3.2 GWAS and identification of QTLs

GWAS were performed using genotypic and phenotypic data of 216 potato genotypes. QQ plots were generated for each environment and location–storage condition combination, and deviations from the diagonal of the plot at the upper‐right end indicate SNPs with a strong association with the tuber dormancy period (Figures S2 and S3).

**FIGURE 5:**
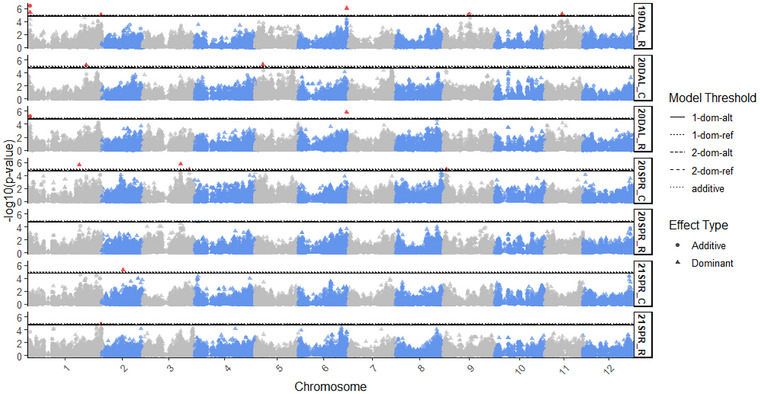
Manhattan plot showing significant marker‐trait associations identified for tuber dormancy length of potatoes for individual environments. Obtained from the evaluation of 216 potato genotypes in seven environments involving two locations (Dalhart and Springlake, TX), 3 years (2019–2021), and two storage conditions (room temperature and cold storage). The numbers 19, 20, and 21 in the artwork denote years 2019, 2020, and 2021; DAL and SPR denote Dalhart and Springlake; and R and C denote room temperature and cold storage.

**FIGURE 6:**
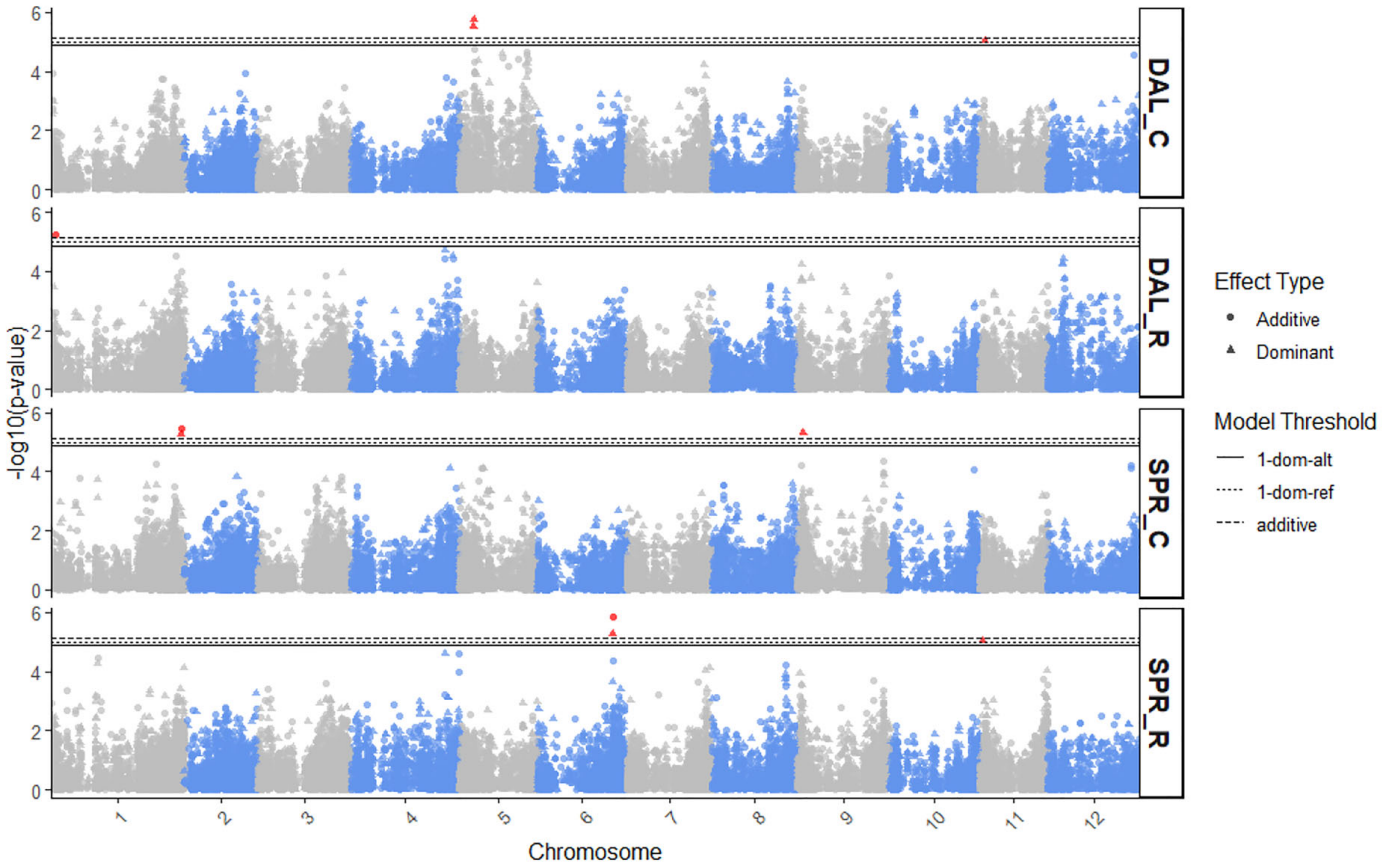
Manhattan plot showing significant marker‐trait associations identified for tuber dormancy length of potatoes when multiple environments were combined. Obtained from the evaluation of 216 potato genotypes in seven environments involving two locations (Dalhart and Springlake, TX), 3 years (2019–2021), and two storage conditions (room temperature and cold storage). C, cold storage; DAL, Dalhart; R, room temperature; SPR, Springlake.

A total of 12 QTLs were identified as having significant associations with the length of tuber dormancy on chromosomes 1, 2, 3, 5, 6, 9, and 11, each explaining up to 11.8% of the phenotypic variation (Figures 5 and 6; Tables 4 and 5). For three of the QTLs with peaks at markers PotVar0072441 (chromosome 1), PotVar0030874 (chromosome 3), and solcap_snp_c1_1400 (chromosome 9), the dominant alleles were found to shorten the tuber dormancy period. In the case of the remaining eight QTLs with peak markers PotVar0122409 (chromosome 1), solcap_snp_c2_17537 (chromosome 1), PotVar0049732 (chromosome 1), solcap_snp_c2_21682 (chromosome 2), solcap_snp_c2_25654 (chromosome 3), solcap_snp_c2_51019 (chromosome 5), PotVar0057253 (chromosome 6), and solcap_snp_c2_4979 (chromosome 11), the presence of the dominant alleles extended the tuber dormancy period. In addition, for the QTL with peak at marker solcap_snp_c2_21216 (chromosome 1), each additional copy of the alternate allele shortens the tuber dormancy period. Notably, the SNP PotVar0057253 at the peak of the QTL on chromosome 6 at 57.2 Mb, explained the highest proportion of phenotypic variation (11.8%) for tuber dormancy (Table 4).

**TABLE 4:** Significant marker‐trait associations identified for tuber dormancy period length of potatoes in individual environments. Analysis was based on the evaluation of 216 potato genotypes in seven environments involving two locations (Dalhart and Springlake, TX), 3 years (2019–2021), and two storage conditions (room temperature and cold storage).

Env.	SNP at QTL peak	Chr	Position in base pairs	Model	Threshold	Score [−log10(p)]	Effect	*R* ^2^ (%)
19DAL_R	solcap_snp_c2_21216	1	2,867,377	Additive	5.03	6.43	−7.3^a^	9.4
	PotVar0057253	6	57,527,967	2‐dom‐ref	4.86	6.00	16.1^b^	11.8
	solcap_snp_c2_4979	11	20,700,395	2‐dom‐ref	4.86	5.07	12.3	7.4
20DAL_C	solcap_snp_c2_17537	1	70,097,516	2‐dom‐alt	4.83	5.20	15.7	8.2
	solcap_snp_c2_51019	5	9,407,434	1‐dom‐alt	4.76	5.24	18.0	7.4
20DAL_R	solcap_snp_c2_21216	1	2,867,377	Additive	5.03	5.19	−6.7^c^	7.4
	PotVar0057253	6	57,527,967	2‐dom‐ref	4.86	5.80	17.1	7.9
20SPR_C	PotVar0049732	1	61,773,942	2‐dom‐ref	4.86	5.63	17.2	10.1
	solcap_snp_c2_25654	3	45,603,662	2‐dom‐ref	4.86	5.79	15.5	6.3
	PotVar0030874	3	55,659,251	1‐dom‐alt	4.76	4.88	−15.4	5.8
	solcap_snp_c1_1400	9	3,920,030	1‐dom‐alt	4.76	4.89	−12.0	5.6
21SPR_C	solcap_snp_c2_21682	2	25,478,266	2‐dom‐alt	4.83	5.30	12.3	9.3
21SPR_R	PotVar0122409	1	87,889,313	2‐dom‐ref	4.86	4.88	13.3	8.8

*Note*: The numbers 19, 20, and 21in column 1 (env.) denote years 2019, 2020, and 2021; DAL and SPR denote Dalhart and Springlake; and C and R denote cold storage and room temperature.

Abbreviations: Env., environment; QTL, quantitative trait loci; SNP, single nucleotide polymorphism.

^a^ Under the additive inheritance model, a negative value indicates that each copy of the alternate allele reduces the mean of the tuber dormancy period.

^b^ Under dominant inheritance models, a positive value indicates that the dominant allele increases the mean of the tuber dormancy period.

^c^ Under dominant inheritance models, a negative value indicates that the dominant allele reduces the mean of the tuber dormancy period.

**TABLE 5:** Significant marker‐trait associations identified for tuber dormancy period length of potatoes when multiple environments were combined. Obtained from the evaluation of 216 potato genotypes in seven environments involving two locations (Dalhart and Springlake, TX), 3 years (2019–2021), and two storage conditions (room temperature and cold storage).

Env.	SNP at QTL peak	Chr	Position in base pairs	Model	Threshold	Score [−log10(p)]	Effect	*R* ^2^ (%)
DAL_R	solcap_snp_c2_21216	1	2,867,377	Additive	5.03	5.84	−6.8^a^	3.7
	PotVar0072441	1	68,293,598	2‐dom‐ref	4.86	4.88	−11.6^b^	2.5
	PotVar0057253	6	57,527,967	2‐dom‐ref	4.86	6.15	16.4^c^	4.0
SPR_C	PotVar0049732	1	61,773,942	2‐dom‐ref	4.86	4.89	15.7	4.0
R	PotVar0057253	6	57,527,967	2‐dom‐ref	4.86	5.27	14.7	2.5
C	solcap_snp_c2_17537	1	70,097,516	2‐dom‐alt	4.83	4.95	10.9	2.4

Note: DAL and SPR in column 1 (env.) denote Dalhart and Springlake and C and R denote cold storage and room temperature.

Abbreviations: Env., environment; QTL, quantitative trait loci; SNP, single nucleotide polymorphism.

^a^ Under an additive inheritance model, a negative value indicates that each additional copy of the alternate allele reduces the mean of the tuber dormancy period.

^b^ Under dominant inheritance models, a negative value indicates that the dominant allele reduces the mean of the tuber dormancy period.

^c^ Under dominant inheritance models, a positive value indicates that the dominant allele increases the mean of the tuber dormancy period.

The SNP marker solcap_snp_c2_21216 was identified in both 19DAL_R and 20DAL_R with *R* ^2^ of 9.4% and 7.4%, respectively, and remained significant when data from both years were combined (with an *R* ^2^ of 3.7%) (Tables 4 and 5). The QTL with the SNP PotVar0057253 at peak showed significant association with tuber dormancy in two environments, explaining 11.8% and 7.9% of phenotypic variation in 19DAL_R and 20DAL_R, respectively, and was subsequently detected when data from multiple years were combined (DAL_R, *R* ^2^ at 4.0%) and when data from Springlake were also included (Room temperature storage—R, with an *R* ^2^ of 2.5%) (Tables 4 and 5). The SNP solcap_snp_c2_17537 at the peak of a QTL on chromosome 1 was detected in 20DAL_C with an *R* ^2^ of 8.2% and remained significant when phenotypic data from Springlake were added in the analysis (Cold storage—C, *R* ^2^ of 2.4%). The QTL with the SNP PotVar0049732 at peak was found to be significantly associated with tuber dormancy in 20SPR_C, with an *R* ^2^ of 10.1%, and was also identified when data from 2020 and 2021 were combined (SPR_C), with an *R* ^2^ of 4.0% (Tables 4 and 5).

In addition, seven of the QTLs were found to be significant in a single environment (i.e., a year, location, and storage condition combination) (Table 4). One QTL with the SNP PotVar0072441 at peak was not detected in individual environments but was found to be significant in DAL_R (when phenotypic data from 2019 and 2020 were combined), with an *R* ^2^ of 2.5% (Table 5).

For GWAS, the effects of allele dosage and different inheritance models were taken into consideration. Three SNPs were identified with the simplex dominant models (three 1‐dom‐alt) to be associated with tuber dormancy period length (Tables 4 and 5). The duplex dominant inheritance models identified eight SNPs (six 2‐dom‐ref and two 2‐dom‐alt) involved in tuber dormancy (Tables  4 and 5). One SNP was identified with an additive inheritance model (Tables 4 and 5).

The genes within the 2‐Mb flanking genomic region of each peak SNP were retrieved from the potato reference genome (Table S6). Genes encoding sugar transporters were detected near SNP PotVar0049732 at 61773942 on chromosome 1. In proximity to the SNP solcap_snp_c2_17537 on chromosome 1, genes encoding for gibberellin (GA) receptor GID1 and gibberellin 20 oxidase were identified. Multiple genes involved in cell cycle regulation were detected within 2 Mb of the SNPs, such as genes encoding cyclins found near the SNP PotVar0057253 on chromosome 6, genes for cyclin‐dependent protein kinase (CDK) near the SNP PotVar0072441 on chromosome 1, and genes encoding cell division control 20 (CDC20) near solcap_snp_c2_25654 on chromosome 3. Considering the broad extent of LD in potatoes, genes regulating dormancy may be located megabases away from the associated SNPs (Figure S4).

### 3.3 GEBVs for length of tuber dormancy periods

GEBVs were calculated for 292 genotypes for four location–storage condition combinations separately (Table S7). They can help guide parental selection and the advancement of genotypes in the breeding pipeline. The model that includes additive, dominance, genotype × environment, and residual variances (Model 4) was selected based on a lower AIC (Figure S5; Table S8).

When harvested from Dalhart and stored at 18°C, the genotype COTX10138‐15Wpe/Y (specialty type white with purple eyes and yellow flesh) had the lowest GEBV for tuber dormancy period length at 58.0. In contrast, the red potato genotype TX17805‐12R/R (red skin, red flesh) had the highest GEBV of 145.3 (Table S7). Among the reference varieties, Atlantic (chipper) and Yukon Gold (yellow) showed shorter tuber dormancy, with GEBVs at 78.6 and 77.1 (or *z*‐scores at −0.40 and −0.50), respectively, while Russet Burbank (processing russet) had a much higher GEBV of 118.7 (*z*‐score of 2.09) (Table 6). Recently released varieties from the Texas A&M Potato Breeding Program, Reveille Russet and Vanguard Russet, were among the clones with long tuber dormancy, having GEBVs at 127.8 and 124.7, or *z*‐scores of 2.66 and 2.47, respectively (Table 6). When tubers produced in Dalhart were stored at 4°C, NDTX081644‐CAB‐2W (chipper) had the lowest GEBV of 90.6, while ATX9202‐3Ru (russet) had the highest GEBV of 195.3 (Table S7). Atlantic had a GEBV of 141.3 (*z*‐score at 0.19), while GEBV of Russet Burbank was at 169.9 (*z*‐score at 1.94). Reveille Russet and Vanguard Russet consistently had high GEBVs (172.5 or *z*‐score of 2.10 for Reveille Russet, and GEBV of 157.2 or *z*‐score of 1.16 for Vanguard Russet) (Table 6).

**TABLE 6:** Genomic‐estimated breeding values (GEBV) and *z*‐scores of released or near‐release potato varieties for tuber dormancy period length evaluated in 11 environments involving two locations (Dalhart and Springlake), 4 years (2019–2022), and two storage conditions (room temperature and cold storage). GEBVs for the remainder genotypes are available in Table S7.

Genotype	DAL_C		DAL_R		SPR_C		SPR_R	
	GEBV	*z*	GEBV	*z*	GEBV	*z*	GEBV	*z*
Atlantic	141.3	0.19	78.6	−0.40	118.3	0.19	72.4	0.19
*ATTX10007‐1Ru* ^a^	143.4	0.32	87.1	0.12	115.7	−0.06	67.1	−0.22
Banana	164.8	1.63	79.1	−0.37	116.4	0.01	57.0	−1.00
Chieftain	141.0	0.17	82.3	−0.17	120.4	0.39	63.8	−0.47
*COTX08365F‐3P/P*	164.3	1.60	118.4	2.08	131.5	1.44	87.6	1.37
*COTX09022‐3RuRE/Y*	133.8	−0.26	80.2	−0.31	99.9	−1.55	65.6	−0.34
*COTX10080‐2Ru*	123.4	−0.90	93.7	0.54	100.7	−1.47	60.9	−0.70
*Dune Russet*	134.8	−0.21	79.8	−0.33	106.1	−0.97	66.8	−0.24
*Krantz*	129.8	−0.51	78.4	−0.41	94.5	−2.06	57.0	−1.00
*PORTX03PG25‐2R/R*	131.8	−0.39	69.6	−0.97	122.3	0.57	65.3	−0.36
Red LaSoda	135.4	−0.17	90.5	0.34	124.6	0.79	78.2	0.63
*Reveille Russet*	172.5	2.10	127.8	2.66	134.2	1.69	89.0	1.47
*Rio Rojo*	135.9	−0.14	91.3	0.39	124.4	0.77	75.9	0.46
Russet Burbank	169.9	1.94	118.7	2.09	125.0	0.82	88.0	1.39
*Russet Norkotah 278*	145.7	0.46	107.7	1.41	103.6	−1.20	83.7	1.06
*Russet Norkotah 296*	145.7	0.46	107.9	1.42	103.7	−1.20	83.6	1.06
*Sierra GoldTM*	134.2	−0.24	66.7	−1.15	105.2	−1.05	54.5	−1.20
*Sierra RoseTM*	131.5	−0.41	68.4	−1.04	109.4	−0.65	54.6	−1.19
*Stampede Russet*	141.9	0.23	85.8	0.04	116.4	0.01	68.7	−0.09
Tacna	130.3	−0.48	73.3	−0.74	108.9	−0.70	66.4	−0.28
*Vanguard Russet*	157.2	1.16	124.7	2.47	116.5	0.02	105.7	2.76
Yukon Gold	126.2	−0.73	77.1	−0.50	115.8	−0.05	59.9	−0.77

Note: DAL and SPR denote Dalhart and Springlake and R and C denote room temperature and cold storage.

Abbreviations: GEBV, genomic‐estimated breeding value; *z*, *z*‐score.

^a^ Genotype names written in italic are varieties or advanced genotypes that were fully or partially developed by the Texas A&M Potato Breeding Program.

**FIGURE 7:**
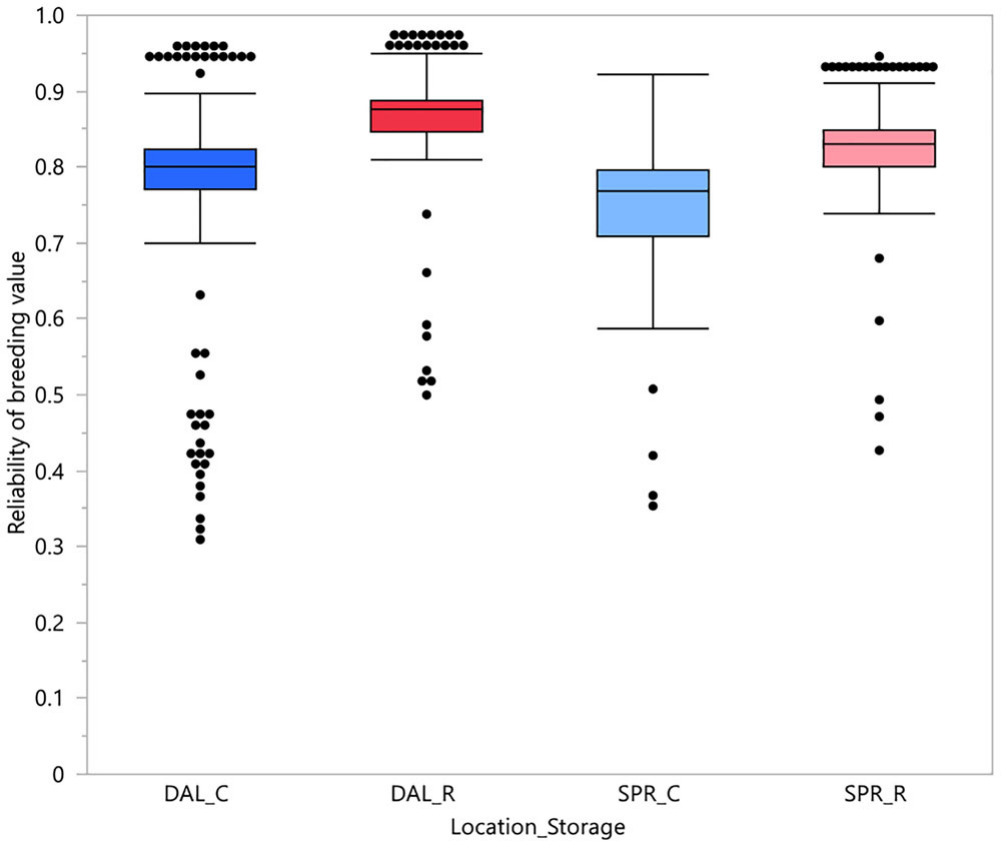
Boxplot of the reliabilities of genomic prediction using both genotypic and phenotypic data. Based on tuber dormancy length measured in 292 potato genotypes in 11 environments involving two locations (Dalhart and Springlake), 4 years (2019–2022), and two storage conditions (room temperature and cold storage).

Tubers harvested from Springlake showed a similar pattern to those from Dalhart when stored at room temperature (18°C), with COTX10138‐15Wpe/Y and TX17805‐12R/R having the lowest (42.5) and the highest (136.9) GEBVs, respectively (Table S7). GEBVs for Atlantic and Russet Burbank were at 72.4 (*z*‐score at 0.19) and 88.0 (*z*‐score at 1.39). Under this location–storage condition combination, Reveille Russet had a GEBV of 89.0 (*z*‐score of 1.47) and Vanguard Russet had a GEBV of 105.7 (*z*‐score of 2.76) (Table 6). When tubers from Springlake were stored at 4°C, GEBV of Krantz was the lowest (94.5) while ATTX10265‐4R/Y had the highest GEBV of 151.0 (Table S7). Atlantic had a GEBV of 118.3 (*z*‐score of 0.19) while Russet Burbank had a GEBV of 125.0 (0.82). Reveille Russet had a high GEBV of 134.2 (*z*‐score of 1.69) while Vanguard Russet had a GEBV of 116.5 (*z*‐score of 0.02) (Table 6). Most of the predicted reliabilities for tuber dormancy length in potatoes were higher than 0.70 (Figure 7).

Spearman's rank correlation analysis was performed to compare how the genotypes rank in GEBV for tuber dormancy length under different location‐storage condition combinations, indicating that significant correlations (*p* < 0.05) were found between all pairs of location–storage condition combinations. The strongest correlation (*ρ* = 0.87) was found between genotype rankings under SPR_R (tubers were produced in Springlake, TX, and stored at 18°C) and DAL_R (tubers were produced in Dalhart, TX, and stored at 18°C).

### 3.4 Cross‐validation

**FIGURE 8:**
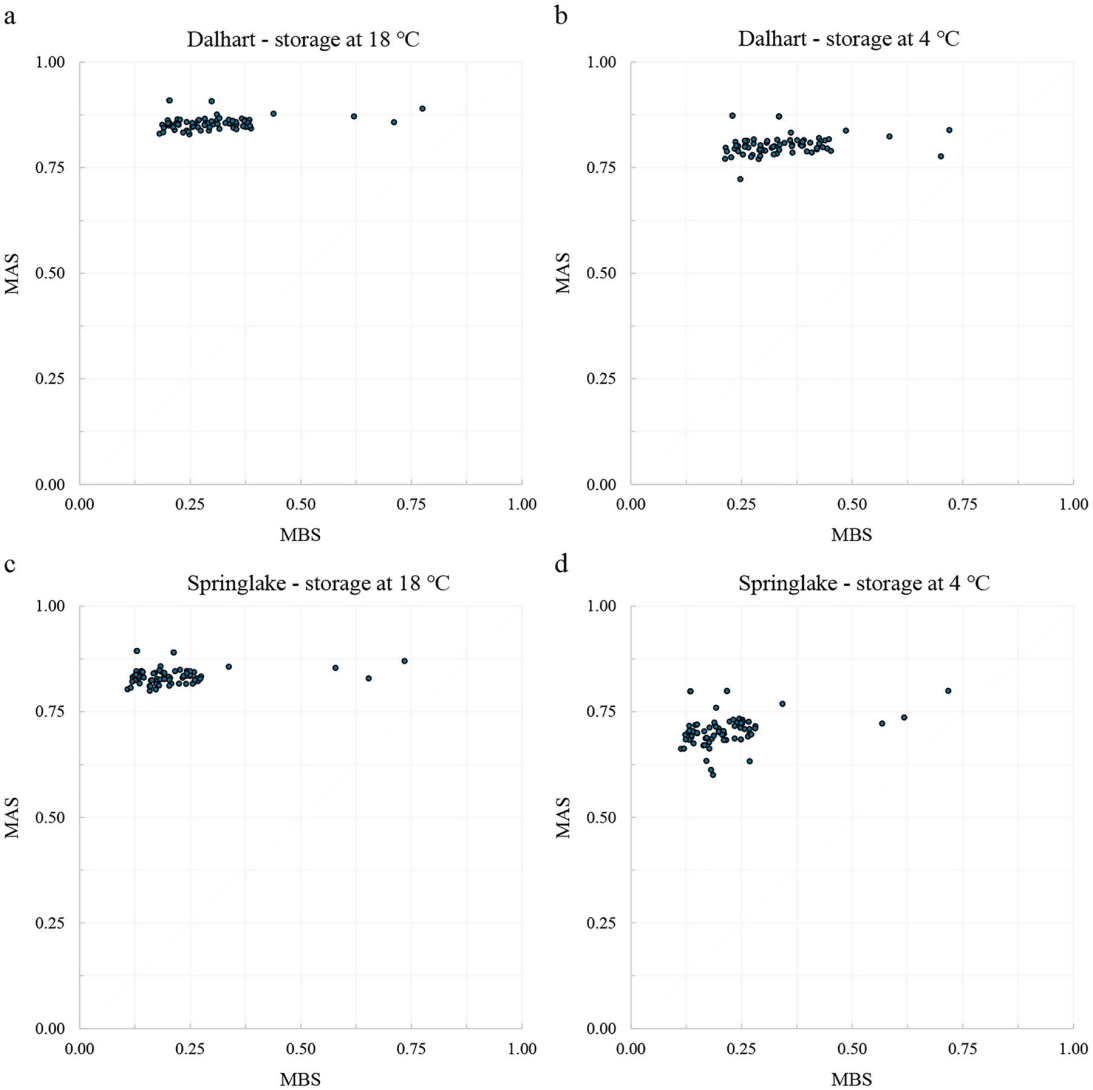
Reliability of the phenotypic predictions of the tuber dormancy period for potato genotypes evaluated in 2022 based on observed phenotype and marker data (marker‐assisted selection [MAS]) and when on only marker data (marker‐based selection [MBS]). (a) From Dalhart and stored at room temperature (18°C), (b) from Dalhart and kept in cold storage (4°C), (c) from Springlake and stored at room temperature (18°C), and (d) from Springlake and kept in cold storage (4°C).

Year‐wise cross‐validation performed using genotypes evaluated between 2019 and 2021 (panel one—216 members) as the training population (both genotyped and phenotyped) and the new genotypes added in 2022 (76 individuals) as the testing set showed that predictions based solely on marker data (MBS) yielded lower reliabilities (0.18–0.78 based on data from Dalhart–room temperature) than predictions using both phenotypic and marker data (MAS) (0.83–0.91 based on data from Dalhart–room temperature) (Figure 8).

**FIGURE 9:**
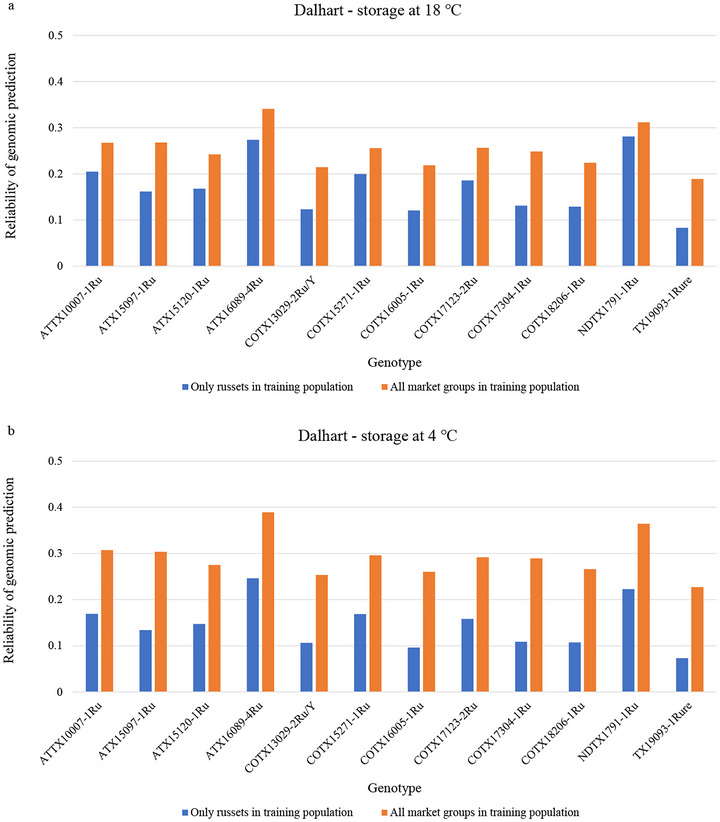
Comparison of reliability of genomic prediction for 12 russet genotypes newly included in dormancy evaluation in 2022 between when only russets were included in the training population (77 genotypes evaluated from 2019 to 2021) and when all market groups were included in the training population (216 genotypes evaluated from 2019 to 2021). (a) From Dalhart and stored at room temperature (18°C) and (b) from Dalhart and kept in cold storage (4°C).

Cross‐validation based on market group was only performed for russets since the other market groups had too few individuals. To demonstrate the targeted approach where russets were used for both the training population (77 genotypes) and the test set (12 genotypes), we focused on data from the Dalhart location. MBS resulted in lower reliabilities (0.08–0.28) than MAS (0.75–0.79). For the untargeted approach, the entire panel one (216 genotypes including all market groups and wide variation for the length of tuber dormancy) was used as the training set and the same test set (12 russet genotypes) as in the targeted approach. The reliabilities of MBS of the untargeted approach were 57.3% and 117.5% higher than the targeted approach for Dalhart at room temperature and cold storage, respectively (Figure 9).

To further assess the predictive performance of the genomic prediction model, a fivefold cross‐validation scheme was conducted. The average reliability of predictions across the five folds ranged from 0.27 to 0.32, with an overall mean reliability of 0.30 (SD = 0.019).

### 3.5 Correlation between tuber dormancy and other traits

Significant correlations were found between tuber dormancy length and traits such as tuber contents of some amino acids, amylose, and minerals, as well as percentage of hollow heart, skin strength, tuber shape, degree of russeting, eye depth, average tuber number per plant, average tuber weight, and total and marketable yield (Table 7). The strongest positive correlation was found between tuber dormancy and tuber calcium content (*ρ* = 0.50), followed by the correlation between tuber dormancy and degree of russeting (*ρ* = 0.48). The average tuber number per plant had the strongest negative correlation with tuber dormancy length (Table 7).

**TABLE 7:** Correlation between length of potato tuber dormancy period and other traits of interest based on evaluation of 216 genotypes in 2019 and 2020 at Dalhart, TX.

Category	Trait	Correlation coefficient *ρ*	Significance
Amino acid	Histidine	−0.10	0.1576
	Arginine	−0.17 ^a^	0.0116
	Asparagine	0.04	0.5209
	Glutamine	0.11	0.1061
	Serine	−0.13	0.0606
	Glutamic Acid	0.13	0.0570
	Aspartic Acid	−0.06	0.3532
	Threonine	−0.19	0.0054
	Glycine	−0.03	0.6663
	Alanine	−0.12	0.0694
	Proline	−0.13	0.0596
	Lysine	−0.05	0.4671
	Tyrosine	0.03	0.6845
	Methionine	0.12	0.0754
	Valine	0.04	0.5941
	Isoleucine	−0.07	0.3393
	Leucine	0.06	0.3945
	Phenylalanine	−0.25	0.0002
Minerals	Nitrogen	−0.07	0.2823
	Phosphorus	0.07	0.2946
	Potassium	−0.12	0.0882
	Calcium	0.50 ^b^	<0.0001
	Magnesium	0.27	<0.0001
	Sulfur	−0.08	0.2213
	Sodium	0.39	<0.0001
	Zinc	−0.16	0.0204
	Iron	0.19	0.0057
	Copper	0.11	0.1092
	Manganese	0.06	0.4036
	Boron	−0.01	0.8880
Defects	Vascular discoloration	0.05	0.4330
	Hollow heart	0.22	0.0010
	Feathering	0.00	0.9938
	Growth cracks	0.05	0.4363
	Knobs	0.06	0.3565
Tuber composition	Protein	−0.08	0.2654
	Amylose	0.26	0.0002
	Solids	−0.14	0.0435
	Dry matter	−0.02	0.7209
	Specific gravity	0.01	0.8922
Tuber trait	Skin strength	0.22	0.0011
	Tuber shape	0.42	<0.0001
	Degree of russeting	0.48	<0.0001
	Eye depth	−0.19	0.0058
Yield	Average tuber number/plant	−0.46	<0.0001
	Average tuber weight	0.30	<0.0001
	Total yield	−0.19	0.0048
	Marketable yield	−0.23	0.0007
	%Marketable yield	−0.14	0.0416

^a^ Significant negative correlations (with *p* value smaller than 0.05) are colored in red.

^b^ Significant positive correlations (with *p* value smaller than 0.05) are colored in blue.

## 4 DISCUSSION

The dormancy period of potato tubers is a crucial trait that significantly impacts storage life, marketability, winter grow‐outs, and subsequent production (in the case of seed potatoes). This study examined the natural variation in tuber dormancy periods in a potato diversity panel comprising materials from the Texas A&M Potato Breeding Program and reference varieties. Based on the first panel evaluated, there was a large variation in tuber dormancy length across different potato genotypes, ranging from 33 to 168 days at room temperature and 80 to 214 days in cold storage (Table 2). Significant differences in tuber dormancy length were also observed among potatoes from different locations, stored under varying storage conditions, and belonging to various market groups. In addition, the genetic basis determining the length of the tuber dormancy period in potatoes was investigated, and GS was utilized to identify genotypes with extended tuber dormancy periods for advancement or use as parents in the breeding program. GWAS and GS, as demonstrated in this study, complement traditional breeding by providing insights into the genetic architecture of tuber dormancy and enabling the selection of genotypes with the desired dormancy length for advancement or use as parents.

The location where potatoes are grown can significantly influence the dormancy period of the tubers. Potato tubers harvested from Springlake had significantly shorter tuber dormancy period (on average 77.5 days when stored at 18°C and 123.7 days at 4°C) compared to tubers out of the Dalhart location (on average 85.0 and 146.0 days at 18°C and 4°C, respectively) (Table 2). This is likely a result of the differences in growing seasons and environmental factors between the two locations. At the Springlake location, temperature continued to increase throughout the growing season (March to July) (Gautam, Pandey, et al., 2024). As a result, high temperatures (>35°C during the day and >20°C at night) were frequent near harvest. While at the Dalhart location, the temperature peaked around 60–70 days after planting and then decreased through the remainder of the growing season (Gautam, Pandey, et al., 2024). Photoperiod changes followed a similar pattern, increasing over the majority of the growing season in Springlake and only starting to decrease late in the growing season. While in Dalhart, photoperiod peaked early in the growing season and was much shorter near harvesting time compared to in Springlake. Previous studies have demonstrated that high temperatures during the growing season can lead to shortened tuber dormancy period (Gautam, Scheuring, et al., 2024; Levy & Veilleux, 2007; G. Zhang et al., 2021). G. Zhang et al. (2021) also found that the GA biosynthesis pathway was enriched in tubers exposed to heat, which potentially contributed to the early sprouting in heat‐stressed tubers. This could be a valuable area for future research to better understand the hormonal regulation of dormancy under different environmental conditions. On the other hand, the direct effect of photoperiod on tuber dormancy is difficult to assess under field conditions. Photoperiod plays a crucial role in regulating tuber initiation and maturation, which in turn affects tuber dormancy (Burton, 1989; Suttle, 2007).

Storage conditions also affected tuber dormancy length, with potato tubers placed in cold storage having significantly longer tuber dormancy period (on average 146 days and 123.7 days for tubers from Dalhart and Springlake, respectively) compared to tubers kept at room temperature (85.0 days and 77.5 days for Dalhart and Springlake tubers, respectively). Cold storage is a common method to delay tuber sprouting in the potato industry. Our study is consistent with previous studies (Espen et al., 1999; Murigi et al., 2021) that highlighted the role of reduced temperatures in maintaining dormancy and preventing premature sprouting, further validating the use of cold storage as a critical tool in postharvest management.

The potato tuber dormancy period also showed significant variation among different market groups. Russet potatoes remained dormant for a longer period under most conditions, while yellows consistently showed the shortest tuber dormancy period from both field locations and under various storage conditions. This variation may be a result of intentional selection by breeders for better storage to cater to various market groups. A potential reason is related to differences in skin properties, which can impact gas exchange and water permeability, potentially affecting tuber dormancy and sprouting. Skin russeting may result in increased tuber transpiration, as reflected by the elevated levels of calcium in russet‐skinned tubers compared to smooth‐skinned tubers of the same variety, as observed by Ginzberg et al. (2012). In apple fruits, water loss was higher from russeted genotypes compared to non‐russeted genotypes, and increased diffusion of water vapor was observed through excised skin segments from russeted surfaces than from non‐russeted surfaces of the same fruit (Khanal et al., 2019). Further research is needed to compare water permeability and rate of gas exchange between russet‐skinned and smooth‐skinned potato tubers under different storage conditions to understand the effect of skin type on tuber dormancy.

Moderate to high heritability was found for tuber dormancy among different environments and location–storage condition combinations, which suggests that genetics play a large role in regulating potato tuber dormancy, and progress can be made through breeding and selection.

To better understand the genetic basis of potato tuber dormancy, we performed GWAS using phenotypic and genotypic data of 216 of the genotypes in the diversity panel, and we uncovered 12 QTL on seven different chromosomes associated with potato tuber dormancy (Figures 5 and 6; Tables 4 and 5). Among the 12 QTL identified, seven were identified in single environments. Many environmental conditions can play a role in regulating tuber dormancy, such as temperature during the growth period (Levy & Veilleux, 2007; G. Zhang et al., 2021), plant nutrient availability and fertilization (H. Zhang et al., 2022), and soil moisture level (Lewis, 2007; Pinhero et al., 2009). Although only two field locations were used for this study, both locations have weather conditions that vary greatly. There were also differences in aspects such as temperature and humidity levels between the two storage conditions. Thus, the QTLs that were only significant in one environment might be involved in the plant's response to specific environmental factors, or to events that occurred only in specific environments, such as flooding and hailstorms in 2021. It is also possible that in some environments, the effects of these QTL were masked by other QTL with greater effects on tuber dormancy. In comparison, the QTL that are stable across multiple environments may contain dormancy‐related genes that are less affected by the environment. Despite the moderate to high heritability of tuber dormancy across environments, no major‐effect QTLs were identified, suggesting that the genetic variance underlying tuber dormancy is distributed across many loci with smaller effects.

Multiple SNPs identified in this study were in proximity to SNPs previously reported by Bisognin et al. (2018) to affect tuber dormancy release and tuber apical dominance release. The SNP solcap_snp_c2_21682 identified on chromosome 2 was only 1.6 Mb away from the SNP chr02_27.1_c2_45308 found by Bisognin et al. (2018) to be related to both tuber dormancy release and tuber apical dominance release. The QTL detected on chromosome 3, with a peak at SNP solcap_snp_c2_25654, was 4.3 Mb from a SNP marker (chr03_49.9_c1_5774) previously reported by Bisognin et al. (2018) to affect apical dominance release. Another SNP identified on chromosome 3, PotVar0030874, was 2.3 Mb from chr03_53.4_c1_16513, which Bisognin et al. (2018) reported to affect tuber dormancy release, and was 4.3 Mb away from chr03_51.4_c2_1722, which Bisognin et al. (2018) found to be related to apical dominance release. The QTL on chromosome 5 with a peak at SNP solcap_snp_c2_51019 was 3.5 Mb from two of the SNPs also identified by Bisognin et al. (2018), one of which (chr05_5.9_c2_47663) affected tuber dormancy release, while the other (chr05_5.9_c2_47646) regulated apical dominance release.

Considering the high LD (window size: 5–10 Mb) in potatoes, all the aforementioned SNP markers we identified are within a reasonable distance from the SNPs detected by Bisognin et al. (2018). In addition, the SNP solcap_snp_c2_21682 on chromosome 2 was also only 2.7 Mb from a SNP discovered by Sharma et al. (2021) (solcap_snp_c1_9719). These SNPs, located in close proximity to each other, may be associated with the same gene or gene set involved in regulating potato tuber dormancy.

We searched the genomic regions within 2 Mb around the peak of each SNP for potential candidate genes related to the regulation of potato tuber dormancy. Candidate genes encoding sugar transporters (such as PGSC0003DMG400025073 and PGSC0003DMG401000567) were also detected within the 2‐Mb region from the SNP PotVar0049732 on chromosome 1. Sugar transporters are involved in the transportation and sensing of reducing sugars in plants. Additionally, in potatoes, they have been found to reduce sugar accumulation in tubers during cold storage (Liu et al., 2023). At room temperature, sugar accumulation in potato tubers also occurs throughout the dormancy period, with sugar concentration peaking at the time of sprouting. It is likely that sugar transporters are involved in this process (Abbasi et al., 2015).

Some of the genes we found were related to phytohormone biosynthesis and response, such as a gene encoding gibberellin 20‐oxidase (PGSC0003DMG400000011) (Table S6). GA 20‐oxidase is involved in the biosynthesis of bioactive GAs (Chen et al., 2022). In previous studies, exogenous GAs, such as GA_1_, GA_3,_ and GA_20_ were found to accelerate the dormancy release process and increase sprout rate in potato tubers (Che et al., 2022; Suttle, 2004). Overexpression of a gene encoding potato GA 20‐oxidase, *StGA20ox1*, led to taller plants, longer internodes, delayed tuberization, and shorter dormancy (Carrera et al., 2000). A different gene encoding gibberellin 20‐oxidase‐1 was among the most differentially expressed genes when sprouted and dormant tubers were compared by G. Zhang et al. (2021).

Genes encoding proteins for cell cycle regulation were also found near the SNPs we detected, such as cyclins (PGSC0003DMG401020104), CDK (PGSC0003DMG400031168), and CDC20 (PGSC0003DMG400000540). Cyclins and CDKs are known to be involved in the transition of cells from G1 to the S phase, while the Arabidopsis homologs of potato CDC20 were found to regulate the degradation of cyclins and the progression of mitosis (Hu et al., 2023; Kevei et al., 2011). During the tuber dormancy period, there is a temporary arrest of the cell cycle of the tuber meristem cells at the G1 phase (M. Campbell et al., 1996). Cyclins, CDKs, and CDC20 likely play a role in the transition of tuber meristematic cells out of the cell cycle arrest state during dormancy release.

Although our search identified some potential candidate genes involved in potato tuber dormancy and sprouting, the resolution of QTL mapping using GWAS in tetraploid potatoes is limited by the high level of LD. Thus, further research is needed to evaluate and verify the roles of the candidate genes in the maintenance and release of potato tuber dormancy. Metabolomics techniques can help analyze changes in phytohormone and sugar contents in potato tubers throughout the tuber dormancy period. At the same time, transcriptomic work will aid in the understanding of gene activities related to tuber dormancy and sprouting.

The fact that only QTLs with mainly small effects were detected suggests that the potato tuber dormancy period is under complex genetic control and is of a more quantitative nature, highlighting the potential of using more comprehensive approaches such as GS. GS is a variant of MAS that utilizes all markers covering the entire genome of a phenotyped and genotyped population to predict GEBVs, which can then be used for selecting parents or genotypes for advancement (Meuwissen et al., 2001). GS in potatoes is made possible by the availability of the complete potato genome sequence and a large amount of molecular marker data (Slater et al., 2016). The recently developed R package StageWise is useful in calculating GEBVs in species with complex polyploid genomes, such as tetraploid potatoes (Endelman, 2023a).

For GS to be applied in the breeding programs, the reliability of GS models needs to be assessed, and it is usually done by employing cross‐validation. In our study, year‐wise cross‐validation utilizing both genotypic and phenotypic data (MAS) consistently resulted in higher reliability compared to using genotypic data alone (MBS) (Figure 8), suggesting that both genotyping and phenotyping are important to improve the estimation of GEBVs and better guide breeding.

When cross‐validation was performed for the russet market group, the targeted approach resulted in lower reliability compared to the untargeted approach (Figure 9). While the targeted approach ensures closer genetic relationship between the training population and the test set, the smaller size of the training population (77 genotypes compared to 216 in the untargeted approach) likely led to the reduced reliability, since size of training population has been shown to affect reliability of genomic prediction (Heffner et al., 2011). Our results were also consistent with previous findings where combining populations was shown to increase reliability of genomic prediction (de Roos et al., 2009). It is possible that genomic prediction conducted within market groups can improve reliability of genomic prediction, provided that the training population within each market group is large enough, which is not the case in our study. Reliability of genomic prediction within market groups will be further assessed in the future with larger and more balanced populations.

Similar to year‐wise cross‐validation, fivefold cross‐validation also revealed relatively low reliability, suggesting that it may be necessary to increase the training population size or to explore alternative prediction models as model choice has been shown to impact genomic prediction reliability (Haile et al., 2021). In addition, once a larger training population is available, further optimization of the training population can be carried out by inclusion of more parents of the genotypes that are being predicted, or by removal of unrelated genotypes.

In our study, GEBVs for the length of the tuber dormancy period were calculated for the 292 genotypes using StageWise and can be useful in selecting genotypes to advance or for use as parents in future breeding projects. For the development of potato genotypes with a long tuber dormancy period, genotypes with higher GEBVs should be selected. On the other hand, shorter dormancy periods may be preferred in regions where potatoes are cultivated for multiple seasons; therefore, genotypes with lower GEBVs can be more desirable.

Significant correlations were found among genotype rankings in GEBV for tuber dormancy under different location‐storage condition combinations. The strongest correlation (*ρ* = 0.87) was observed between genotype rankings in tuber dormancy under SPR_R and DAL_R, indicating that the relative tuber dormancy lengths of genotypes are consistent across different growing environments. Thus, selection for tuber dormancy length under one growing environment will likely be valid when the potato genotypes are grown in a different environment.

The tuber dormancy length was found to be moderately correlated with tuber calcium content, degree of russeting, and average tuber number per plant (Table 6). Although there is limited study on calcium in regulating potato tuber dormancy, calcium has been found to affect bud dormancy in several other plants (Gai et al., 2024; Pang et al., 2007). In grape buds previously induced for sprouting with hydrogen cyanamide (HC) or chilling, when the Ca^2+^ signaling was limited, the inducing effect of HC and chilling was negated. At the same time, the supply of Ca^2+^ was able to eliminate the effect and promote bud break (Pang et al., 2007). Similar effects were observed in the study by Gai et al. (2024) on tree peony. This is likely due to the role of calcium in activating the GA biosynthesis and signaling pathways, as well as in suppressing abscisic acid biosynthesis and signaling (Gai et al., 2024).

The correlation between tuber dormancy and degree of russeting is consistent with previous observations, where russet potatoes remained dormant for a significantly longer period compared to potatoes of other market groups (Table 3). Further research will be necessary to understand the differences in skin properties between russet and smooth‐skinned potatoes and determine if these properties have an impact on tuber dormancy regulation.

Negative correlations exists between the length of tuber dormancy and the average number of tubers per plant, and positive correlations were found between tuber dormancy and average tuber weight. This may be explained bytuber initiation and tuber maturity at the time of harvest. Further studies using populations that have not undergone artificial selection and are allowed to grow until maturity are needed to better understand the relationship between tuber dormancy and yield.

## 5 CONCLUSIONS

The current study aimed to provide valuable insights into the variation in tuber dormancy length among advanced potato genotypes under different growing and storage conditions. Extensive phenotypic variation was observed in dormancy duration across genotypes, environments, storage conditions, and market types. Notably, tubers harvested from Dalhart displayed longer dormancy periods compared to those from Springlake, likely due to differences in temperature and photoperiod during the growing season. Storage at 4°C significantly prolonged dormancy relative to room temperature, reaffirming the role of cold storage in postharvest management. We also identified QTLs for tuber dormancy length by applying GWAS to tetraploid potato germplasm, with several QTLs co‐localizing with regions previously reported for dormancy and apical dominance release. Candidate genes identified within 2 Mb of peak SNPs include those related to sugar transport, GA biosynthesis and signaling (e.g., GA20‐oxidase and GID1), and cell cycle regulators such as cyclins, CDKs, and CDC20. These genes may play pivotal roles in maintaining dormancy and releasing it through hormonal and metabolic pathways. Despite the identification of biologically relevant QTLs, the modest effect sizes and broad LD suggest that tuber dormancy is a polygenic trait under complex genetic control. Consequently, GS offers a more practical route for breeding, as it enables prediction and selection based on genome‐wide marker effects. GEBVs for tuber dormancy length based on both genotypic and phenotypic data are recommended for advancing genotypes in the breeding pipeline and also to identify parents since reliabilities of MAS were much higher than MBS. Future work involving transcriptomics, metabolomics, and physiological studies will be essential to validate candidate genes and elucidate the molecular mechanisms underlying dormancy regulation under diverse environmental conditions.

## AUTHOR CONTRIBUTIONS

**Ao Jiao**: Data curation; formal analysis; investigation; methodology; visualization; writing—original draft; writing—review and editing. **Sanjeev Gautam**: Conceptualization; data curation; investigation; methodology; writing—review and editing. **Jeewan Pandey**: Data curation; formal analysis; visualization; writing—review and editing. **Douglas C. Scheuring**: Funding acquisition; methodology; resources; writing—review and editing. **Jeffrey W. Koym**: Methodology; resources. M. **Isabel Vales**: Conceptualization; funding acquisition; methodology; project administration; resources; supervision; writing—review and editing.

## CONFLICT OF INTEREST STATEMENT

The authors declare no conflicts of interest.

## Supporting information



Supplemental Material

Supplemental Material

Supplemental Material

Supplemental Material

Supplemental Material

Supplemental Material

Supplemental Material

Supplemental Material

Supplemental Material

Supplemental Material

Supplemental Material

## Data Availability

All phenotypic and genotypic data are uploaded in Tables S4 and S9, respectively.
