# Supplementary material for: Genome‐wide association studies and genomics‐informed breeding for tuber dormancy in tetraploid potatoes
Source: Plant Genome. 2026 Feb 19;19(1):e70205. doi: 10.1002/tpg2.70205 (PMC12919731; doi:10.1002/tpg2.70205)
Supplement: Supplementary file 1 [file TPG2-19-e70205-s002.pdf]

Genome-wide association studies and genomics-informed breeding for tuber dormancy in tetraploid potatoes

Ao Jiao, Sanjeev Gautam, Jeewan Pandey, Douglas C. Scheuring, Jeffrey W. Koym, and M. Isabel Vales

Supplementary Table S4. Tuber dormancy lengths of 298 potato genotypes evaluated in two locations (Dalhart and Springlake, Texas) for four years (2019 - 2022) under two storage conditions (room temperature and cold storage).
19, 20, 21, 22 - years 2019, 2020, 2021, 2022; DAL - Dalhart; SPR - Springlake; C - cold storage - 4 ℃; R - room temperature - 18 ℃.

Clone Name	Rep	Year	Location	Storage temperature (℃)	Environment	Dormancy period (days)	Market Group
AOR07781-2	1	2019	Dalhart	18	19DAL-R	56	Russet
AOR07781-2	2	2019	Dalhart	18	19DAL-R	56	Russet
AOR07781-2	1	2020	Dalhart	4	20DAL-C		Russet
AOR07781-2	2	2020	Dalhart	4	20DAL-C		Russet
AOR07781-2	1	2020	Dalhart	18	20DAL-R	48	Russet
AOR07781-2	2	2020	Dalhart	18	20DAL-R	48	Russet
AOR07781-2	1	2020	Springlake	4	20SPR-C	95	Russet
AOR07781-2	2	2020	Springlake	4	20SPR-C	88	Russet
AOR07781-2	1	2020	Springlake	18	20SPR-R	39	Russet
AOR07781-2	2	2020	Springlake	18	20SPR-R	39	Russet
AOR07781-2	1	2021	Springlake	4	21SPR-C	107	Russet
AOR07781-2	2	2021	Springlake	4	21SPR-C	114	Russet
AOR07781-2	1	2021	Springlake	18	21SPR-R	56	Russet
AOR07781-2	2	2021	Springlake	18	21SPR-R	56	Russet
AORTX09037-1W/Y	1	2019	Dalhart	18	19DAL-R	63	Chipper
AORTX09037-1W/Y	2	2019	Dalhart	18	19DAL-R	70	Chipper
AORTX09037-1W/Y	1	2020	Dalhart	4	20DAL-C		Chipper
AORTX09037-1W/Y	2	2020	Dalhart	4	20DAL-C	133	Chipper
AORTX09037-1W/Y	1	2020	Dalhart	18	20DAL-R	81	Chipper
AORTX09037-1W/Y	2	2020	Dalhart	18	20DAL-R	74	Chipper
AORTX09037-1W/Y	1	2020	Springlake	4	20SPR-C	108	Chipper
AORTX09037-1W/Y	2	2020	Springlake	4	20SPR-C	136	Chipper
AORTX09037-1W/Y	1	2020	Springlake	18	20SPR-R	66	Chipper
AORTX09037-1W/Y	2	2020	Springlake	18	20SPR-R	80	Chipper
AORTX09037-1W/Y	1	2021	Springlake	4	21SPR-C	133	Chipper
AORTX09037-1W/Y	2	2021	Springlake	4	21SPR-C	126	Chipper
AORTX09037-1W/Y	1	2021	Springlake	18	21SPR-R	84	Chipper
AORTX09037-1W/Y	2	2021	Springlake	18	21SPR-R	77	Chipper
AORTX09037-5W/Y	1	2022	Dalhart	4	22DAL-C	88	Yellow
AORTX09037-5W/Y	2	2022	Dalhart	4	22DAL-C	88	Yellow
AORTX09037-5W/Y	1	2022	Dalhart	18	22DAL-R	71	Yellow
AORTX09037-5W/Y	2	2022	Dalhart	18	22DAL-R	71	Yellow
AORTX09037-5W/Y	1	2022	Springlake	4	22SPR-C	62	Yellow
AORTX09037-5W/Y	2	2022	Springlake	4	22SPR-C	83	Yellow
AORTX09037-5W/Y	1	2022	Springlake	18	22SPR-R	52	Yellow
AORTX09037-5W/Y	2	2022	Springlake	18	22SPR-R	52	Yellow
AOTX02136-1Ru	1	2019	Dalhart	18	19DAL-R	91	Russet
AOTX02136-1Ru	2	2019	Dalhart	18	19DAL-R	91	Russet

AOTX02136-1Ru	1	2020	Dalhart	4	20DAL-C	179	Russet
AOTX02136-1Ru	2	2020	Dalhart	4	20DAL-C	172	Russet
AOTX02136-1Ru	1	2020	Dalhart	18	20DAL-R	95	Russet
AOTX02136-1Ru	2	2020	Dalhart	18	20DAL-R	95	Russet
AOTX02136-1Ru	1	2020	Springlake	4	20SPR-C	130	Russet
AOTX02136-1Ru	2	2020	Springlake	4	20SPR-C	123	Russet
AOTX02136-1Ru	1	2020	Springlake	18	20SPR-R	81	Russet
AOTX02136-1Ru	2	2020	Springlake	18	20SPR-R	67	Russet
AOTX02136-1Ru	1	2021	Springlake	4	21SPR-C		Russet
AOTX02136-1Ru	2	2021	Springlake	4	21SPR-C	162	Russet
AOTX02136-1Ru	1	2021	Springlake	18	21SPR-R	119	Russet
AOTX02136-1Ru	2	2021	Springlake	18	21SPR-R	105	Russet
AOTX03187-1Ru	1	2019	Dalhart	18	19DAL-R	91	Russet
AOTX03187-1Ru	2	2019	Dalhart	18	19DAL-R	84	Russet
AOTX03187-1Ru	1	2020	Dalhart	4	20DAL-C	165	Russet
AOTX03187-1Ru	2	2020	Dalhart	4	20DAL-C	165	Russet
AOTX03187-1Ru	1	2020	Dalhart	18	20DAL-R	87	Russet
AOTX03187-1Ru	2	2020	Dalhart	18	20DAL-R	95	Russet
AOTX03187-1Ru	1	2020	Springlake	4	20SPR-C	123	Russet
AOTX03187-1Ru	2	2020	Springlake	4	20SPR-C	116	Russet
AOTX03187-1Ru	1	2020	Springlake	18	20SPR-R	74	Russet
AOTX03187-1Ru	2	2020	Springlake	18	20SPR-R	74	Russet
AOTX03187-1Ru	1	2021	Springlake	4	21SPR-C	114	Russet
AOTX03187-1Ru	2	2021	Springlake	4	21SPR-C	128	Russet
AOTX03187-1Ru	1	2021	Springlake	18	21SPR-R	91	Russet
AOTX03187-1Ru	2	2021	Springlake	18	21SPR-R	77	Russet
AOTX05043-1Ru	1	2019	Dalhart	18	19DAL-R	91	Russet
AOTX05043-1Ru	2	2019	Dalhart	18	19DAL-R	91	Russet
AOTX05043-1Ru	1	2020	Dalhart	4	20DAL-C	144	Russet
AOTX05043-1Ru	2	2020	Dalhart	4	20DAL-C	207	Russet
AOTX05043-1Ru	1	2020	Dalhart	18	20DAL-R	129	Russet
AOTX05043-1Ru	2	2020	Dalhart	18	20DAL-R	117	Russet
AOTX05043-1Ru	1	2020	Springlake	4	20SPR-C	116	Russet
AOTX05043-1Ru	2	2020	Springlake	4	20SPR-C	123	Russet
AOTX05043-1Ru	1	2020	Springlake	18	20SPR-R	89	Russet
AOTX05043-1Ru	2	2020	Springlake	18	20SPR-R	89	Russet
AOTX05043-1Ru	1	2021	Springlake	4	21SPR-C	149	Russet
AOTX05043-1Ru	2	2021	Springlake	4	21SPR-C	149	Russet
AOTX05043-1Ru	1	2021	Springlake	18	21SPR-R	98	Russet
AOTX05043-1Ru	2	2021	Springlake	18	21SPR-R	91	Russet
AOTX91861-4R	1	2019	Dalhart	18	19DAL-R	77	Red
AOTX91861-4R	2	2019	Dalhart	18	19DAL-R	77	Red
AOTX91861-4R	1	2020	Dalhart	4	20DAL-C	140	Red
AOTX91861-4R	2	2020	Dalhart	4	20DAL-C	140	Red
AOTX91861-4R	1	2020	Dalhart	18	20DAL-R	102	Red
AOTX91861-4R	2	2020	Dalhart	18	20DAL-R	102	Red
AOTX91861-4R	1	2020	Springlake	4	20SPR-C	129	Red

AOTX91861-4R	2	2020	Springlake	4	20SPR-C	122	Red
AOTX91861-4R	1	2020	Springlake	18	20SPR-R	73	Red
AOTX91861-4R	2	2020	Springlake	18	20SPR-R	73	Red
AOTX91861-4R	1	2021	Springlake	4	21SPR-C	133	Red
AOTX91861-4R	2	2021	Springlake	4	21SPR-C	126	Red
AOTX91861-4R	1	2021	Springlake	18	21SPR-R	112	Red
AOTX91861-4R	2	2021	Springlake	18	21SPR-R	98	Red
AOTX93483-1R	1	2019	Dalhart	18	19DAL-R	56	Red
AOTX93483-1R	2	2019	Dalhart	18	19DAL-R	70	Red
AOTX93483-1R	1	2020	Dalhart	4	20DAL-C	151	Red
AOTX93483-1R	2	2020	Dalhart	4	20DAL-C	133	Red
AOTX93483-1R	1	2020	Dalhart	18	20DAL-R	87	Red
AOTX93483-1R	2	2020	Dalhart	18	20DAL-R	87	Red
AOTX93483-1R	1	2020	Springlake	4	20SPR-C	115	Red
AOTX93483-1R	2	2020	Springlake	4	20SPR-C	115	Red
AOTX93483-1R	1	2020	Springlake	18	20SPR-R	66	Red
AOTX93483-1R	2	2020	Springlake	18	20SPR-R	52	Red
AOTX93483-1R	1	2021	Springlake	4	21SPR-C	174	Red
AOTX93483-1R	2	2021	Springlake	4	21SPR-C		Red
AOTX93483-1R	1	2021	Springlake	18	21SPR-R		Red
AOTX93483-1R	2	2021	Springlake	18	21SPR-R	105	Red
AOTX95265-1Ru	1	2019	Dalhart	18	19DAL-R	112	Russet
AOTX95265-1Ru	2	2019	Dalhart	18	19DAL-R	112	Russet
AOTX95265-1Ru	1	2020	Dalhart	4	20DAL-C	151	Russet
AOTX95265-1Ru	2	2020	Dalhart	4	20DAL-C		Russet
AOTX95265-1Ru	1	2020	Dalhart	18	20DAL-R	102	Russet
AOTX95265-1Ru	2	2020	Dalhart	18	20DAL-R		Russet
AOTX95265-1Ru	1	2020	Springlake	4	20SPR-C	95	Russet
AOTX95265-1Ru	2	2020	Springlake	4	20SPR-C	102	Russet
AOTX95265-1Ru	1	2020	Springlake	18	20SPR-R	81	Russet
AOTX95265-1Ru	2	2020	Springlake	18	20SPR-R	81	Russet
AOTX95265-1Ru	1	2021	Springlake	4	21SPR-C	114	Russet
AOTX95265-1Ru	2	2021	Springlake	4	21SPR-C	107	Russet
AOTX95265-1Ru	1	2021	Springlake	18	21SPR-R	105	Russet
AOTX95265-1Ru	2	2021	Springlake	18	21SPR-R	105	Russet
AOTX95265-2ARu	1	2019	Dalhart	18	19DAL-R	77	Russet
AOTX95265-2ARu	2	2019	Dalhart	18	19DAL-R	84	Russet
AOTX95265-2ARu	1	2020	Dalhart	4	20DAL-C		Russet
AOTX95265-2ARu	2	2020	Dalhart	4	20DAL-C		Russet
AOTX95265-2ARu	1	2020	Dalhart	18	20DAL-R	95	Russet
AOTX95265-2ARu	2	2020	Dalhart	18	20DAL-R	95	Russet
AOTX95265-2ARu	1	2020	Springlake	4	20SPR-C	102	Russet
AOTX95265-2ARu	2	2020	Springlake	4	20SPR-C	95	Russet
AOTX95265-2ARu	1	2020	Springlake	18	20SPR-R	67	Russet
AOTX95265-2ARu	2	2020	Springlake	18	20SPR-R	74	Russet
AOTX95265-2ARu	1	2021	Springlake	4	21SPR-C		Russet
AOTX95265-2ARu	2	2021	Springlake	4	21SPR-C	87	Russet

AOTX95265-2ARu	1	2021	Springlake	18	21SPR-R	119	Russet
AOTX95265-2ARu	2	2021	Springlake	18	21SPR-R	105	Russet
AOTX95265-2Ru	1	2019	Dalhart	18	19DAL-R	91	Russet
AOTX95265-2Ru	2	2019	Dalhart	18	19DAL-R		Russet
AOTX95265-2Ru	1	2020	Dalhart	4	20DAL-C	133	Russet
AOTX95265-2Ru	2	2020	Dalhart	4	20DAL-C	133	Russet
AOTX95265-2Ru	1	2020	Dalhart	18	20DAL-R	102	Russet
AOTX95265-2Ru	2	2020	Dalhart	18	20DAL-R	95	Russet
AOTX95265-2Ru	1	2020	Springlake	4	20SPR-C	109	Russet
AOTX95265-2Ru	2	2020	Springlake	4	20SPR-C	88	Russet
AOTX95265-2Ru	1	2020	Springlake	18	20SPR-R	67	Russet
AOTX95265-2Ru	2	2020	Springlake	18	20SPR-R	74	Russet
AOTX95265-2Ru	1	2021	Springlake	4	21SPR-C	114	Russet
AOTX95265-2Ru	2	2021	Springlake	4	21SPR-C	121	Russet
AOTX95265-2Ru	1	2021	Springlake	18	21SPR-R	119	Russet
AOTX95265-2Ru	2	2021	Springlake	18	21SPR-R	112	Russet
AOTX95265-3Ru	1	2019	Dalhart	18	19DAL-R	112	Russet
AOTX95265-3Ru	2	2019	Dalhart	18	19DAL-R	112	Russet
AOTX95265-3Ru	1	2020	Dalhart	4	20DAL-C	165	Russet
AOTX95265-3Ru	2	2020	Dalhart	4	20DAL-C	151	Russet
AOTX95265-3Ru	1	2020	Dalhart	18	20DAL-R	109	Russet
AOTX95265-3Ru	2	2020	Dalhart	18	20DAL-R	109	Russet
AOTX95265-3Ru	1	2020	Springlake	4	20SPR-C	102	Russet
AOTX95265-3Ru	2	2020	Springlake	4	20SPR-C	116	Russet
AOTX95265-3Ru	1	2020	Springlake	18	20SPR-R	81	Russet
AOTX95265-3Ru	2	2020	Springlake	18	20SPR-R	81	Russet
AOTX95265-3Ru	1	2021	Springlake	4	21SPR-C	107	Russet
AOTX95265-3Ru	2	2021	Springlake	4	21SPR-C	114	Russet
AOTX95265-3Ru	1	2021	Springlake	18	21SPR-R	98	Russet
AOTX95265-3Ru	2	2021	Springlake	18	21SPR-R	112	Russet
AOTX95265-4Ru	1	2019	Dalhart	18	19DAL-R	98	Russet
AOTX95265-4Ru	2	2019	Dalhart	18	19DAL-R		Russet
AOTX95265-4Ru	1	2020	Dalhart	4	20DAL-C	165	Russet
AOTX95265-4Ru	2	2020	Dalhart	4	20DAL-C	165	Russet
AOTX95265-4Ru	1	2020	Dalhart	18	20DAL-R	109	Russet
AOTX95265-4Ru	2	2020	Dalhart	18	20DAL-R	109	Russet
AOTX95265-4Ru	1	2020	Springlake	4	20SPR-C	116	Russet
AOTX95265-4Ru	2	2020	Springlake	4	20SPR-C	95	Russet
AOTX95265-4Ru	1	2020	Springlake	18	20SPR-R	81	Russet
AOTX95265-4Ru	2	2020	Springlake	18	20SPR-R	81	Russet
AOTX95265-4Ru	1	2021	Springlake	4	21SPR-C		Russet
AOTX95265-4Ru	2	2021	Springlake	4	21SPR-C	107	Russet
AOTX95265-4Ru	1	2021	Springlake	18	21SPR-R	105	Russet
AOTX95265-4Ru	2	2021	Springlake	18	21SPR-R	98	Russet
AOTX95295-3Ru	1	2019	Dalhart	18	19DAL-R	98	Russet
AOTX95295-3Ru	2	2019	Dalhart	18	19DAL-R		Russet
AOTX95295-3Ru	1	2020	Dalhart	4	20DAL-C	144	Russet

AOTX95295-3Ru	2	2020	Dalhart	4	20DAL-C	133	Russet
AOTX95295-3Ru	1	2020	Dalhart	18	20DAL-R	95	Russet
AOTX95295-3Ru	2	2020	Dalhart	18	20DAL-R	109	Russet
AOTX95295-3Ru	1	2020	Springlake	4	20SPR-C	109	Russet
AOTX95295-3Ru	2	2020	Springlake	4	20SPR-C	116	Russet
AOTX95295-3Ru	1	2020	Springlake	18	20SPR-R	74	Russet
AOTX95295-3Ru	2	2020	Springlake	18	20SPR-R	81	Russet
AOTX95295-3Ru	1	2021	Springlake	4	21SPR-C		Russet
AOTX95295-3Ru	2	2021	Springlake	4	21SPR-C	107	Russet
AOTX95295-3Ru	1	2021	Springlake	18	21SPR-R		Russet
AOTX95295-3Ru	2	2021	Springlake	18	21SPR-R	84	Russet
AOTX95309-1W	1	2019	Dalhart	18	19DAL-R	56	Chipper
AOTX95309-1W	2	2019	Dalhart	18	19DAL-R	63	Chipper
AOTX95309-1W	1	2020	Dalhart	4	20DAL-C		Chipper
AOTX95309-1W	2	2020	Dalhart	4	20DAL-C		Chipper
AOTX95309-1W	1	2020	Dalhart	18	20DAL-R	81	Chipper
AOTX95309-1W	2	2020	Dalhart	18	20DAL-R	74	Chipper
AOTX95309-1W	1	2020	Springlake	4	20SPR-C	101	Chipper
AOTX95309-1W	2	2020	Springlake	4	20SPR-C	101	Chipper
AOTX95309-1W	1	2020	Springlake	18	20SPR-R	66	Chipper
AOTX95309-1W	2	2020	Springlake	18	20SPR-R	66	Chipper
AOTX95309-1W	1	2021	Springlake	4	21SPR-C	112	Chipper
AOTX95309-1W	2	2021	Springlake	4	21SPR-C	112	Chipper
AOTX95309-1W	1	2021	Springlake	18	21SPR-R	63	Chipper
AOTX95309-1W	2	2021	Springlake	18	21SPR-R	63	Chipper
AOTX95309-2W*	1	2019	Dalhart	18	19DAL-R	56	Chipper
AOTX95309-2W*	2	2019	Dalhart	18	19DAL-R	70	Chipper
AOTX95309-2W*	1	2020	Dalhart	4	20DAL-C	133	Chipper
AOTX95309-2W*	2	2020	Dalhart	4	20DAL-C	151	Chipper
AOTX95309-2W*	1	2020	Dalhart	18	20DAL-R	81	Chipper
AOTX95309-2W*	2	2020	Dalhart	18	20DAL-R	60	Chipper
AOTX95309-2W*	1	2020	Springlake	4	20SPR-C	122	Chipper
AOTX95309-2W*	2	2020	Springlake	4	20SPR-C	101	Chipper
AOTX95309-2W*	1	2020	Springlake	18	20SPR-R	38	Chipper
AOTX95309-2W*	2	2020	Springlake	18	20SPR-R	52	Chipper
AOTX95309-2W*	1	2021	Springlake	4	21SPR-C	112	Chipper
AOTX95309-2W*	2	2021	Springlake	4	21SPR-C	133	Chipper
AOTX95309-2W*	1	2021	Springlake	18	21SPR-R	77	Chipper
AOTX95309-2W*	2	2021	Springlake	18	21SPR-R	84	Chipper
AOTX96075-1Ru	1	2019	Dalhart	18	19DAL-R	105	Russet
AOTX96075-1Ru	2	2019	Dalhart	18	19DAL-R	112	Russet
AOTX96075-1Ru	1	2020	Dalhart	4	20DAL-C	165	Russet
AOTX96075-1Ru	2	2020	Dalhart	4	20DAL-C	158	Russet
AOTX96075-1Ru	1	2020	Dalhart	18	20DAL-R	109	Russet
AOTX96075-1Ru	2	2020	Dalhart	18	20DAL-R	102	Russet
AOTX96075-1Ru	1	2020	Springlake	4	20SPR-C	109	Russet
AOTX96075-1Ru	2	2020	Springlake	4	20SPR-C	95	Russet

AOTX96075-1Ru	1	2020	Springlake	18	20SPR-R	81	Russet
AOTX96075-1Ru	2	2020	Springlake	18	20SPR-R	81	Russet
AOTX96075-1Ru	1	2021	Springlake	4	21SPR-C	107	Russet
AOTX96075-1Ru	2	2021	Springlake	4	21SPR-C	107	Russet
AOTX96075-1Ru	1	2021	Springlake	18	21SPR-R	112	Russet
AOTX96075-1Ru	2	2021	Springlake	18	21SPR-R	98	Russet
AOTX96084-1Ru	1	2019	Dalhart	18	19DAL-R	105	Russet
AOTX96084-1Ru	2	2019	Dalhart	18	19DAL-R	112	Russet
AOTX96084-1Ru	1	2020	Dalhart	4	20DAL-C	165	Russet
AOTX96084-1Ru	2	2020	Dalhart	4	20DAL-C	158	Russet
AOTX96084-1Ru	1	2020	Dalhart	18	20DAL-R	109	Russet
AOTX96084-1Ru	2	2020	Dalhart	18	20DAL-R	109	Russet
AOTX96084-1Ru	1	2020	Springlake	4	20SPR-C	95	Russet
AOTX96084-1Ru	2	2020	Springlake	4	20SPR-C	109	Russet
AOTX96084-1Ru	1	2020	Springlake	18	20SPR-R	81	Russet
AOTX96084-1Ru	2	2020	Springlake	18	20SPR-R	81	Russet
AOTX96084-1Ru	1	2021	Springlake	4	21SPR-C	128	Russet
AOTX96084-1Ru	2	2021	Springlake	4	21SPR-C	121	Russet
AOTX96084-1Ru	1	2021	Springlake	18	21SPR-R	84	Russet
AOTX96084-1Ru	2	2021	Springlake	18	21SPR-R	105	Russet
AOTX96208-1Ru	1	2019	Dalhart	18	19DAL-R	105	Russet
AOTX96208-1Ru	2	2019	Dalhart	18	19DAL-R	112	Russet
AOTX96208-1Ru	1	2020	Dalhart	4	20DAL-C	151	Russet
AOTX96208-1Ru	2	2020	Dalhart	4	20DAL-C	140	Russet
AOTX96208-1Ru	1	2020	Dalhart	18	20DAL-R	109	Russet
AOTX96208-1Ru	2	2020	Dalhart	18	20DAL-R	102	Russet
AOTX96208-1Ru	1	2020	Springlake	4	20SPR-C	95	Russet
AOTX96208-1Ru	2	2020	Springlake	4	20SPR-C	116	Russet
AOTX96208-1Ru	1	2020	Springlake	18	20SPR-R	89	Russet
AOTX96208-1Ru	2	2020	Springlake	18	20SPR-R	81	Russet
AOTX96208-1Ru	1	2021	Springlake	4	21SPR-C	121	Russet
AOTX96208-1Ru	2	2021	Springlake	4	21SPR-C	128	Russet
AOTX96208-1Ru	1	2021	Springlake	18	21SPR-R	105	Russet
AOTX96208-1Ru	2	2021	Springlake	18	21SPR-R	105	Russet
AOTX96216-1Ru	1	2019	Dalhart	18	19DAL-R	91	Russet
AOTX96216-1Ru	2	2019	Dalhart	18	19DAL-R	98	Russet
AOTX96216-1Ru	1	2020	Dalhart	4	20DAL-C	151	Russet
AOTX96216-1Ru	2	2020	Dalhart	4	20DAL-C	165	Russet
AOTX96216-1Ru	1	2020	Dalhart	18	20DAL-R	87	Russet
AOTX96216-1Ru	2	2020	Dalhart	18	20DAL-R	87	Russet
AOTX96216-1Ru	1	2020	Springlake	4	20SPR-C	123	Russet
AOTX96216-1Ru	2	2020	Springlake	4	20SPR-C	144	Russet
AOTX96216-1Ru	1	2020	Springlake	18	20SPR-R	67	Russet
AOTX96216-1Ru	2	2020	Springlake	18	20SPR-R	81	Russet
AOTX96216-1Ru	1	2021	Springlake	4	21SPR-C	114	Russet
AOTX96216-1Ru	2	2021	Springlake	4	21SPR-C	121	Russet
AOTX96216-1Ru	1	2021	Springlake	18	21SPR-R	77	Russet

AOTX96216-1Ru	2	2021	Springlake	18	21SPR-R	91	Russet
AOTX96216-2Ru	1	2019	Dalhart	18	19DAL-R	91	Russet
AOTX96216-2Ru	2	2019	Dalhart	18	19DAL-R		Russet
AOTX96216-2Ru	1	2020	Dalhart	4	20DAL-C	144	Russet
AOTX96216-2Ru	2	2020	Dalhart	4	20DAL-C	165	Russet
AOTX96216-2Ru	1	2020	Dalhart	18	20DAL-R	95	Russet
AOTX96216-2Ru	2	2020	Dalhart	18	20DAL-R	95	Russet
AOTX96216-2Ru	1	2020	Springlake	4	20SPR-C	123	Russet
AOTX96216-2Ru	2	2020	Springlake	4	20SPR-C	102	Russet
AOTX96216-2Ru	1	2020	Springlake	18	20SPR-R	74	Russet
AOTX96216-2Ru	2	2020	Springlake	18	20SPR-R	67	Russet
AOTX96216-2Ru	1	2021	Springlake	4	21SPR-C	114	Russet
AOTX96216-2Ru	2	2021	Springlake	4	21SPR-C	114	Russet
AOTX96216-2Ru	1	2021	Springlake	18	21SPR-R	77	Russet
AOTX96216-2Ru	2	2021	Springlake	18	21SPR-R	91	Russet
AOTX97213-1Ru	1	2019	Dalhart	18	19DAL-R	98	Russet
AOTX97213-1Ru	2	2019	Dalhart	18	19DAL-R		Russet
AOTX97213-1Ru	1	2020	Dalhart	4	20DAL-C	193	Russet
AOTX97213-1Ru	2	2020	Dalhart	4	20DAL-C	193	Russet
AOTX97213-1Ru	1	2020	Dalhart	18	20DAL-R	109	Russet
AOTX97213-1Ru	2	2020	Dalhart	18	20DAL-R	81	Russet
AOTX97213-1Ru	1	2020	Springlake	4	20SPR-C	151	Russet
AOTX97213-1Ru	2	2020	Springlake	4	20SPR-C		Russet
AOTX97213-1Ru	1	2020	Springlake	18	20SPR-R	89	Russet
AOTX97213-1Ru	2	2020	Springlake	18	20SPR-R	81	Russet
AOTX97213-1Ru	1	2021	Springlake	4	21SPR-C	162	Russet
AOTX97213-1Ru	2	2021	Springlake	4	21SPR-C	169	Russet
AOTX97213-1Ru	1	2021	Springlake	18	21SPR-R	112	Russet
AOTX97213-1Ru	2	2021	Springlake	18	21SPR-R	91	Russet
AOTX98096-1Ru*	1	2019	Dalhart	18	19DAL-R	98	Russet
AOTX98096-1Ru*	2	2019	Dalhart	18	19DAL-R	126	Russet
AOTX98096-1Ru*	1	2020	Dalhart	4	20DAL-C	165	Russet
AOTX98096-1Ru*	2	2020	Dalhart	4	20DAL-C	151	Russet
AOTX98096-1Ru*	1	2020	Dalhart	18	20DAL-R	117	Russet
AOTX98096-1Ru*	2	2020	Dalhart	18	20DAL-R	117	Russet
AOTX98096-1Ru*	1	2020	Springlake	4	20SPR-C	116	Russet
AOTX98096-1Ru*	2	2020	Springlake	4	20SPR-C	88	Russet
AOTX98096-1Ru*	1	2020	Springlake	18	20SPR-R	95	Russet
AOTX98096-1Ru*	2	2020	Springlake	18	20SPR-R	74	Russet
AOTX98096-1Ru*	1	2021	Springlake	4	21SPR-C	107	Russet
AOTX98096-1Ru*	2	2021	Springlake	4	21SPR-C	128	Russet
AOTX98096-1Ru*	1	2021	Springlake	18	21SPR-R	91	Russet
AOTX98096-1Ru*	2	2021	Springlake	18	21SPR-R	119	Russet
AOTX98137-1Ru	1	2019	Dalhart	18	19DAL-R	105	Russet
AOTX98137-1Ru	2	2019	Dalhart	18	19DAL-R	105	Russet
AOTX98137-1Ru	1	2020	Dalhart	4	20DAL-C	165	Russet
AOTX98137-1Ru	2	2020	Dalhart	4	20DAL-C	151	Russet

AOTX98137-1Ru	1	2020	Dalhart	18	20DAL-R	109	Russet
AOTX98137-1Ru	2	2020	Dalhart	18	20DAL-R	102	Russet
AOTX98137-1Ru	1	2020	Springlake	4	20SPR-C	102	Russet
AOTX98137-1Ru	2	2020	Springlake	4	20SPR-C	88	Russet
AOTX98137-1Ru	1	2020	Springlake	18	20SPR-R	81	Russet
AOTX98137-1Ru	2	2020	Springlake	18	20SPR-R	81	Russet
AOTX98137-1Ru	1	2021	Springlake	4	21SPR-C		Russet
AOTX98137-1Ru	2	2021	Springlake	4	21SPR-C	114	Russet
AOTX98137-1Ru	1	2021	Springlake	18	21SPR-R	70	Russet
AOTX98137-1Ru	2	2021	Springlake	18	21SPR-R	98	Russet
AOTX98152-3Ru	1	2019	Dalhart	18	19DAL-R	112	Russet
AOTX98152-3Ru	2	2019	Dalhart	18	19DAL-R	119	Russet
AOTX98152-3Ru	1	2020	Dalhart	4	20DAL-C	133	Russet
AOTX98152-3Ru	2	2020	Dalhart	4	20DAL-C	140	Russet
AOTX98152-3Ru	1	2020	Dalhart	18	20DAL-R	117	Russet
AOTX98152-3Ru	2	2020	Dalhart	18	20DAL-R	117	Russet
AOTX98152-3Ru	1	2020	Springlake	4	20SPR-C	116	Russet
AOTX98152-3Ru	2	2020	Springlake	4	20SPR-C	109	Russet
AOTX98152-3Ru	1	2020	Springlake	18	20SPR-R	89	Russet
AOTX98152-3Ru	2	2020	Springlake	18	20SPR-R	95	Russet
AOTX98152-3Ru	1	2021	Springlake	4	21SPR-C	107	Russet
AOTX98152-3Ru	2	2021	Springlake	4	21SPR-C	114	Russet
AOTX98152-3Ru	1	2021	Springlake	18	21SPR-R	119	Russet
AOTX98152-3Ru	2	2021	Springlake	18	21SPR-R	98	Russet
AOTX98202-1Ru	1	2019	Dalhart	18	19DAL-R	98	Russet
AOTX98202-1Ru	2	2019	Dalhart	18	19DAL-R	98	Russet
AOTX98202-1Ru	1	2020	Dalhart	4	20DAL-C	172	Russet
AOTX98202-1Ru	2	2020	Dalhart	4	20DAL-C	200	Russet
AOTX98202-1Ru	1	2020	Dalhart	18	20DAL-R	95	Russet
AOTX98202-1Ru	2	2020	Dalhart	18	20DAL-R	109	Russet
AOTX98202-1Ru	1	2020	Springlake	4	20SPR-C	151	Russet
AOTX98202-1Ru	2	2020	Springlake	4	20SPR-C	158	Russet
AOTX98202-1Ru	1	2020	Springlake	18	20SPR-R	89	Russet
AOTX98202-1Ru	2	2020	Springlake	18	20SPR-R	74	Russet
AOTX98202-1Ru	1	2021	Springlake	4	21SPR-C	183	Russet
AOTX98202-1Ru	2	2021	Springlake	4	21SPR-C	169	Russet
AOTX98202-1Ru	1	2021	Springlake	18	21SPR-R	105	Russet
AOTX98202-1Ru	2	2021	Springlake	18	21SPR-R	91	Russet
Atlantic	1	2019	Dalhart	18	19DAL-R	63	Chipper
Atlantic	2	2019	Dalhart	18	19DAL-R	63	Chipper
Atlantic	1	2020	Dalhart	4	20DAL-C		Chipper
Atlantic	2	2020	Dalhart	4	20DAL-C		Chipper
Atlantic	1	2020	Dalhart	18	20DAL-R	81	Chipper
Atlantic	2	2020	Dalhart	18	20DAL-R	74	Chipper
Atlantic	1	2022	Dalhart	4	22DAL-C	130	Chipper
Atlantic	2	2022	Dalhart	4	22DAL-C	137	Chipper
Atlantic	1	2022	Dalhart	18	22DAL-R	85	Chipper

Atlantic	2	2022	Dalhart	18	22DAL-R	85	Chipper
Atlantic	1	2020	Springlake	4	20SPR-C	115	Chipper
Atlantic	2	2020	Springlake	4	20SPR-C	115	Chipper
Atlantic	1	2020	Springlake	18	20SPR-R	66	Chipper
Atlantic	2	2020	Springlake	18	20SPR-R	72	Chipper
Atlantic	1	2021	Springlake	4	21SPR-C	119	Chipper
Atlantic	2	2021	Springlake	4	21SPR-C	119	Chipper
Atlantic	1	2021	Springlake	18	21SPR-R	77	Chipper
Atlantic	2	2021	Springlake	18	21SPR-R	77	Chipper
Atlantic	1	2022	Springlake	4	22SPR-C	102	Chipper
Atlantic	2	2022	Springlake	4	22SPR-C	109	Chipper
Atlantic	1	2022	Springlake	18	22SPR-R	57	Chipper
Atlantic	2	2022	Springlake	18	22SPR-R	64	Chipper
ATTX00289-4W	1	2019	Dalhart	18	19DAL-R	56	Chipper
ATTX00289-4W	2	2019	Dalhart	18	19DAL-R	56	Chipper
ATTX00289-4W	1	2020	Dalhart	4	20DAL-C	133	Chipper
ATTX00289-4W	2	2020	Dalhart	4	20DAL-C		Chipper
ATTX00289-4W	1	2020	Dalhart	18	20DAL-R	87	Chipper
ATTX00289-4W	2	2020	Dalhart	18	20DAL-R	95	Chipper
ATTX00289-4W	1	2020	Springlake	4	20SPR-C	122	Chipper
ATTX00289-4W	2	2020	Springlake	4	20SPR-C	101	Chipper
ATTX00289-4W	1	2020	Springlake	18	20SPR-R	66	Chipper
ATTX00289-4W	2	2020	Springlake	18	20SPR-R	73	Chipper
ATTX00289-4W	1	2021	Springlake	4	21SPR-C	112	Chipper
ATTX00289-4W	2	2021	Springlake	4	21SPR-C	92	Chipper
ATTX00289-4W	1	2021	Springlake	18	21SPR-R	77	Chipper
ATTX00289-4W	2	2021	Springlake	18	21SPR-R	77	Chipper
ATTX00289-5R/Y	1	2019	Dalhart	18	19DAL-R	63	Red
ATTX00289-5R/Y	2	2019	Dalhart	18	19DAL-R	63	Red
ATTX00289-5R/Y	1	2020	Dalhart	4	20DAL-C		Red
ATTX00289-5R/Y	2	2020	Dalhart	4	20DAL-C	123	Red
ATTX00289-5R/Y	1	2020	Dalhart	18	20DAL-R	67	Red
ATTX00289-5R/Y	2	2020	Dalhart	18	20DAL-R	60	Red
ATTX00289-5R/Y	1	2020	Springlake	4	20SPR-C	108	Red
ATTX00289-5R/Y	2	2020	Springlake	4	20SPR-C	94	Red
ATTX00289-5R/Y	1	2020	Springlake	18	20SPR-R	45	Red
ATTX00289-5R/Y	2	2020	Springlake	18	20SPR-R	38	Red
ATTX00289-5R/Y	1	2021	Springlake	4	21SPR-C	112	Red
ATTX00289-5R/Y	2	2021	Springlake	4	21SPR-C		Red
ATTX00289-5R/Y	1	2021	Springlake	18	21SPR-R	42	Red
ATTX00289-5R/Y	2	2021	Springlake	18	21SPR-R		Red
ATTX00289-6Y/Y	1	2019	Dalhart	18	19DAL-R	63	Yellow
ATTX00289-6Y/Y	2	2019	Dalhart	18	19DAL-R	77	Yellow
ATTX00289-6Y/Y	1	2020	Dalhart	4	20DAL-C	193	Yellow
ATTX00289-6Y/Y	2	2020	Dalhart	4	20DAL-C	172	Yellow
ATTX00289-6Y/Y	1	2020	Dalhart	18	20DAL-R	67	Yellow
ATTX00289-6Y/Y	2	2020	Dalhart	18	20DAL-R	81	Yellow

ATTX00289-6Y/Y	1	2020	Springlake	4	20SPR-C	115	Yellow
ATTX00289-6Y/Y	2	2020	Springlake	4	20SPR-C	122	Yellow
ATTX00289-6Y/Y	1	2020	Springlake	18	20SPR-R	59	Yellow
ATTX00289-6Y/Y	2	2020	Springlake	18	20SPR-R	59	Yellow
ATTX00289-6Y/Y	1	2021	Springlake	4	21SPR-C	154	Yellow
ATTX00289-6Y/Y	2	2021	Springlake	4	21SPR-C	147	Yellow
ATTX00289-6Y/Y	1	2021	Springlake	18	21SPR-R	70	Yellow
ATTX00289-6Y/Y	2	2021	Springlake	18	21SPR-R	70	Yellow
ATTX01178-1R	1	2019	Dalhart	18	19DAL-R	56	Red
ATTX01178-1R	2	2019	Dalhart	18	19DAL-R	56	Red
ATTX01178-1R	1	2020	Dalhart	4	20DAL-C	179	Red
ATTX01178-1R	2	2020	Dalhart	4	20DAL-C	144	Red
ATTX01178-1R	1	2020	Dalhart	18	20DAL-R	74	Red
ATTX01178-1R	2	2020	Dalhart	18	20DAL-R	60	Red
ATTX01178-1R	1	2020	Springlake	4	20SPR-C	136	Red
ATTX01178-1R	2	2020	Springlake	4	20SPR-C	129	Red
ATTX01178-1R	1	2020	Springlake	18	20SPR-R	73	Red
ATTX01178-1R	2	2020	Springlake	18	20SPR-R	66	Red
ATTX01178-1R	1	2021	Springlake	4	21SPR-C	133	Red
ATTX01178-1R	2	2021	Springlake	4	21SPR-C		Red
ATTX01178-1R	1	2021	Springlake	18	21SPR-R	70	Red
ATTX01178-1R	2	2021	Springlake	18	21SPR-R	77	Red
ATTX01180-1R/Y	1	2019	Dalhart	18	19DAL-R	70	Red
ATTX01180-1R/Y	2	2019	Dalhart	18	19DAL-R	70	Red
ATTX01180-1R/Y	1	2020	Dalhart	4	20DAL-C	133	Red
ATTX01180-1R/Y	2	2020	Dalhart	4	20DAL-C	144	Red
ATTX01180-1R/Y	1	2020	Dalhart	18	20DAL-R	81	Red
ATTX01180-1R/Y	2	2020	Dalhart	18	20DAL-R	81	Red
ATTX01180-1R/Y	1	2020	Springlake	4	20SPR-C	143	Red
ATTX01180-1R/Y	2	2020	Springlake	4	20SPR-C	129	Red
ATTX01180-1R/Y	1	2020	Springlake	18	20SPR-R	59	Red
ATTX01180-1R/Y	2	2020	Springlake	18	20SPR-R	52	Red
ATTX01180-1R/Y	1	2021	Springlake	4	21SPR-C	174	Red
ATTX01180-1R/Y	2	2021	Springlake	4	21SPR-C	174	Red
ATTX01180-1R/Y	1	2021	Springlake	18	21SPR-R	70	Red
ATTX01180-1R/Y	2	2021	Springlake	18	21SPR-R	77	Red
ATTX03516-2R	1	2019	Dalhart	18	19DAL-R	84	Red
ATTX03516-2R	2	2019	Dalhart	18	19DAL-R	70	Red
ATTX03516-2R	1	2020	Dalhart	4	20DAL-C	133	Red
ATTX03516-2R	2	2020	Dalhart	4	20DAL-C	133	Red
ATTX03516-2R	1	2020	Dalhart	18	20DAL-R	81	Red
ATTX03516-2R	2	2020	Dalhart	18	20DAL-R	87	Red
ATTX03516-2R	1	2020	Springlake	4	20SPR-C	108	Red
ATTX03516-2R	2	2020	Springlake	4	20SPR-C	129	Red
ATTX03516-2R	1	2020	Springlake	18	20SPR-R	52	Red
ATTX03516-2R	2	2020	Springlake	18	20SPR-R	59	Red
ATTX03516-2R	1	2021	Springlake	4	21SPR-C	126	Red

ATTX03516-2R	2	2021	Springlake	4	21SPR-C	126	Red
ATTX03516-2R	1	2021	Springlake	18	21SPR-R	84	Red
ATTX03516-2R	2	2021	Springlake	18	21SPR-R	77	Red
ATTX05175s-1R/Y	1	2019	Dalhart	18	19DAL-R	84	Red
ATTX05175s-1R/Y	2	2019	Dalhart	18	19DAL-R	63	Red
ATTX05175s-1R/Y	1	2020	Dalhart	4	20DAL-C		Red
ATTX05175s-1R/Y	2	2020	Dalhart	4	20DAL-C	172	Red
ATTX05175s-1R/Y	1	2020	Dalhart	18	20DAL-R	74	Red
ATTX05175s-1R/Y	2	2020	Dalhart	18	20DAL-R	81	Red
ATTX05175s-1R/Y	1	2020	Springlake	4	20SPR-C	136	Red
ATTX05175s-1R/Y	2	2020	Springlake	4	20SPR-C	136	Red
ATTX05175s-1R/Y	1	2020	Springlake	18	20SPR-R	66	Red
ATTX05175s-1R/Y	2	2020	Springlake	18	20SPR-R	73	Red
ATTX05175s-1R/Y	1	2021	Springlake	4	21SPR-C	133	Red
ATTX05175s-1R/Y	2	2021	Springlake	4	21SPR-C		Red
ATTX05175s-1R/Y	1	2021	Springlake	18	21SPR-R	84	Red
ATTX05175s-1R/Y	2	2021	Springlake	18	21SPR-R	77	Red
ATTX05186-2R	1	2019	Dalhart	18	19DAL-R	63	Red
ATTX05186-2R	2	2019	Dalhart	18	19DAL-R	70	Red
ATTX05186-2R	1	2020	Dalhart	4	20DAL-C	133	Red
ATTX05186-2R	2	2020	Dalhart	4	20DAL-C	140	Red
ATTX05186-2R	1	2020	Dalhart	18	20DAL-R	87	Red
ATTX05186-2R	2	2020	Dalhart	18	20DAL-R	74	Red
ATTX05186-2R	1	2020	Springlake	4	20SPR-C	122	Red
ATTX05186-2R	2	2020	Springlake	4	20SPR-C	94	Red
ATTX05186-2R	1	2020	Springlake	18	20SPR-R	73	Red
ATTX05186-2R	2	2020	Springlake	18	20SPR-R	73	Red
ATTX05186-2R	1	2021	Springlake	4	21SPR-C	147	Red
ATTX05186-2R	2	2021	Springlake	4	21SPR-C	147	Red
ATTX05186-2R	1	2021	Springlake	18	21SPR-R		Red
ATTX05186-2R	2	2021	Springlake	18	21SPR-R	84	Red
ATTX06246-1R	1	2019	Dalhart	18	19DAL-R	119	Red
ATTX06246-1R	2	2019	Dalhart	18	19DAL-R	133	Red
ATTX06246-1R	1	2020	Dalhart	4	20DAL-C		Red
ATTX06246-1R	2	2020	Dalhart	4	20DAL-C		Red
ATTX06246-1R	1	2020	Dalhart	18	20DAL-R	155	Red
ATTX06246-1R	2	2020	Dalhart	18	20DAL-R	155	Red
ATTX06246-1R	1	2020	Springlake	4	20SPR-C	101	Red
ATTX06246-1R	2	2020	Springlake	4	20SPR-C	108	Red
ATTX06246-1R	1	2020	Springlake	18	20SPR-R	108	Red
ATTX06246-1R	2	2020	Springlake	18	20SPR-R	115	Red
ATTX06246-1R	1	2021	Springlake	4	21SPR-C	112	Red
ATTX06246-1R	2	2021	Springlake	4	21SPR-C	119	Red
ATTX06246-1R	1	2021	Springlake	18	21SPR-R	119	Red
ATTX06246-1R	2	2021	Springlake	18	21SPR-R	140	Red
ATTX07042-3W	1	2019	Dalhart	18	19DAL-R	56	Chipper
ATTX07042-3W	2	2019	Dalhart	18	19DAL-R	56	Chipper

ATTX07042-3W	1	2020	Dalhart	4	20DAL-C	133	Chipper
ATTX07042-3W	2	2020	Dalhart	4	20DAL-C	140	Chipper
ATTX07042-3W	1	2020	Dalhart	18	20DAL-R	81	Chipper
ATTX07042-3W	2	2020	Dalhart	18	20DAL-R	95	Chipper
ATTX07042-3W	1	2020	Springlake	4	20SPR-C	101	Chipper
ATTX07042-3W	2	2020	Springlake	4	20SPR-C	94	Chipper
ATTX07042-3W	1	2020	Springlake	18	20SPR-R	45	Chipper
ATTX07042-3W	2	2020	Springlake	18	20SPR-R	59	Chipper
ATTX07042-3W	1	2021	Springlake	4	21SPR-C	119	Chipper
ATTX07042-3W	2	2021	Springlake	4	21SPR-C	112	Chipper
ATTX07042-3W	1	2021	Springlake	18	21SPR-R	84	Chipper
ATTX07042-3W	2	2021	Springlake	18	21SPR-R	84	Chipper
ATTX10007-1Ru	1	2022	Dalhart	4	22DAL-C	157	Russet
ATTX10007-1Ru	2	2022	Dalhart	4	22DAL-C	122	Russet
ATTX10007-1Ru	1	2022	Dalhart	18	22DAL-R	91	Russet
ATTX10007-1Ru	2	2022	Dalhart	18	22DAL-R	91	Russet
ATTX10007-1Ru	1	2022	Springlake	4	22SPR-C	99	Russet
ATTX10007-1Ru	2	2022	Springlake	4	22SPR-C	99	Russet
ATTX10007-1Ru	1	2022	Springlake	18	22SPR-R	47	Russet
ATTX10007-1Ru	2	2022	Springlake	18	22SPR-R	54	Russet
ATTX10265-4R/Y	1	2019	Dalhart	18	19DAL-R	98	Red
ATTX10265-4R/Y	2	2019	Dalhart	18	19DAL-R	105	Red
ATTX10265-4R/Y	1	2020	Dalhart	4	20DAL-C	214	Red
ATTX10265-4R/Y	2	2020	Dalhart	4	20DAL-C	214	Red
ATTX10265-4R/Y	1	2020	Dalhart	18	20DAL-R	102	Red
ATTX10265-4R/Y	2	2020	Dalhart	18	20DAL-R	102	Red
ATTX10265-4R/Y	1	2020	Springlake	4	20SPR-C	171	Red
ATTX10265-4R/Y	2	2020	Springlake	4	20SPR-C		Red
ATTX10265-4R/Y	1	2020	Springlake	18	20SPR-R	80	Red
ATTX10265-4R/Y	2	2020	Springlake	18	20SPR-R	80	Red
ATTX10265-4R/Y	1	2021	Springlake	4	21SPR-C	181	Red
ATTX10265-4R/Y	2	2021	Springlake	4	21SPR-C	188	Red
ATTX10265-4R/Y	1	2021	Springlake	18	21SPR-R	105	Red
ATTX10265-4R/Y	2	2021	Springlake	18	21SPR-R	112	Red
ATTX10333-1W/Y	1	2022	Dalhart	4	22DAL-C	158	Yellow
ATTX10333-1W/Y	2	2022	Dalhart	4	22DAL-C	137	Yellow
ATTX10333-1W/Y	1	2022	Dalhart	18	22DAL-R	85	Yellow
ATTX10333-1W/Y	2	2022	Dalhart	18	22DAL-R	92	Yellow
ATTX10333-1W/Y	1	2022	Springlake	4	22SPR-C	90	Yellow
ATTX10333-1W/Y	2	2022	Springlake	4	22SPR-C	111	Yellow
ATTX10333-1W/Y	1	2022	Springlake	18	22SPR-R	66	Yellow
ATTX10333-1W/Y	2	2022	Springlake	18	22SPR-R	59	Yellow
ATTX88481-1P/W	1	2019	Dalhart	18	19DAL-R	63	Purple
ATTX88481-1P/W	2	2019	Dalhart	18	19DAL-R	56	Purple
ATTX88481-1P/W	1	2020	Dalhart	4	20DAL-C	133	Purple
ATTX88481-1P/W	2	2020	Dalhart	4	20DAL-C	140	Purple
ATTX88481-1P/W	1	2020	Dalhart	18	20DAL-R	53	Purple

ATTX88481-1P/W	2	2020	Dalhart	18	20DAL-R	60	Purple
ATTX88481-1P/W	1	2020	Springlake	4	20SPR-C	143	Purple
ATTX88481-1P/W	2	2020	Springlake	4	20SPR-C	143	Purple
ATTX88481-1P/W	1	2020	Springlake	18	20SPR-R	38	Purple
ATTX88481-1P/W	2	2020	Springlake	18	20SPR-R	38	Purple
ATTX88481-1P/W	1	2021	Springlake	4	21SPR-C	140	Purple
ATTX88481-1P/W	2	2021	Springlake	4	21SPR-C	161	Purple
ATTX88481-1P/W	1	2021	Springlake	18	21SPR-R	84	Purple
ATTX88481-1P/W	2	2021	Springlake	18	21SPR-R	77	Purple
ATTX88654-2P/Y	1	2019	Dalhart	18	19DAL-R	77	Purple
ATTX88654-2P/Y	2	2019	Dalhart	18	19DAL-R	84	Purple
ATTX88654-2P/Y	1	2020	Dalhart	4	20DAL-C	144	Purple
ATTX88654-2P/Y	2	2020	Dalhart	4	20DAL-C	133	Purple
ATTX88654-2P/Y	1	2020	Dalhart	18	20DAL-R	87	Purple
ATTX88654-2P/Y	2	2020	Dalhart	18	20DAL-R	74	Purple
ATTX88654-2P/Y	1	2020	Springlake	4	20SPR-C	129	Purple
ATTX88654-2P/Y	2	2020	Springlake	4	20SPR-C	143	Purple
ATTX88654-2P/Y	1	2020	Springlake	18	20SPR-R	66	Purple
ATTX88654-2P/Y	2	2020	Springlake	18	20SPR-R	87	Purple
ATTX88654-2P/Y	1	2021	Springlake	4	21SPR-C	126	Purple
ATTX88654-2P/Y	2	2021	Springlake	4	21SPR-C	140	Purple
ATTX88654-2P/Y	1	2021	Springlake	18	21SPR-R	84	Purple
ATTX88654-2P/Y	2	2021	Springlake	18	21SPR-R	70	Purple
ATTX95490-2W	1	2019	Dalhart	18	19DAL-R	63	Chipper
ATTX95490-2W	2	2019	Dalhart	18	19DAL-R	63	Chipper
ATTX95490-2W	1	2020	Dalhart	4	20DAL-C	102	Chipper
ATTX95490-2W	2	2020	Dalhart	4	20DAL-C	88	Chipper
ATTX95490-2W	1	2020	Dalhart	18	20DAL-R	53	Chipper
ATTX95490-2W	2	2020	Dalhart	18	20DAL-R	48	Chipper
ATTX95490-2W	1	2020	Springlake	4	20SPR-C	108	Chipper
ATTX95490-2W	2	2020	Springlake	4	20SPR-C	101	Chipper
ATTX95490-2W	1	2020	Springlake	18	20SPR-R	73	Chipper
ATTX95490-2W	2	2020	Springlake	18	20SPR-R	59	Chipper
ATTX95490-2W	1	2021	Springlake	4	21SPR-C	92	Chipper
ATTX95490-2W	2	2021	Springlake	4	21SPR-C	119	Chipper
ATTX95490-2W	1	2021	Springlake	18	21SPR-R	77	Chipper
ATTX95490-2W	2	2021	Springlake	18	21SPR-R	63	Chipper
ATTX961014-1AR/Y	1	2019	Dalhart	18	19DAL-R	126	Red
ATTX961014-1AR/Y	2	2019	Dalhart	18	19DAL-R		Red
ATTX961014-1AR/Y	1	2020	Dalhart	4	20DAL-C		Red
ATTX961014-1AR/Y	2	2020	Dalhart	4	20DAL-C	133	Red
ATTX961014-1AR/Y	1	2020	Dalhart	18	20DAL-R	60	Red
ATTX961014-1AR/Y	2	2020	Dalhart	18	20DAL-R	60	Red
ATTX961014-1AR/Y	1	2020	Springlake	4	20SPR-C	101	Red
ATTX961014-1AR/Y	2	2020	Springlake	4	20SPR-C	101	Red
ATTX961014-1AR/Y	1	2020	Springlake	18	20SPR-R	52	Red
ATTX961014-1AR/Y	2	2020	Springlake	18	20SPR-R	45	Red

ATTX961014-1AR/Y	1	2021	Springlake	4	21SPR-C	126	Red
ATTX961014-1AR/Y	2	2021	Springlake	4	21SPR-C	126	Red
ATTX961014-1AR/Y	1	2021	Springlake	18	21SPR-R	56	Red
ATTX961014-1AR/Y	2	2021	Springlake	18	21SPR-R	63	Red
ATTX961014-1Br/Y	1	2019	Dalhart	18	19DAL-R	56	Red
ATTX961014-1Br/Y	2	2019	Dalhart	18	19DAL-R	63	Red
ATTX961014-1Br/Y	1	2020	Dalhart	4	20DAL-C		Red
ATTX961014-1Br/Y	2	2020	Dalhart	4	20DAL-C		Red
ATTX961014-1Br/Y	1	2020	Dalhart	18	20DAL-R	60	Red
ATTX961014-1Br/Y	2	2020	Dalhart	18	20DAL-R	67	Red
ATTX961014-1Br/Y	1	2020	Springlake	4	20SPR-C	101	Red
ATTX961014-1Br/Y	2	2020	Springlake	4	20SPR-C	108	Red
ATTX961014-1Br/Y	1	2020	Springlake	18	20SPR-R	59	Red
ATTX961014-1Br/Y	2	2020	Springlake	18	20SPR-R	52	Red
ATTX961014-1Br/Y	1	2021	Springlake	4	21SPR-C	112	Red
ATTX961014-1Br/Y	2	2021	Springlake	4	21SPR-C	112	Red
ATTX961014-1Br/Y	1	2021	Springlake	18	21SPR-R	63	Red
ATTX961014-1Br/Y	2	2021	Springlake	18	21SPR-R	63	Red
ATTX961014-1R/Y Chimera	1	2019	Dalhart	18	19DAL-R	70	Red
ATTX961014-1R/Y Chimera	2	2019	Dalhart	18	19DAL-R	70	Red
ATTX961014-1R/Y Chimera	1	2020	Dalhart	4	20DAL-C		Red
ATTX961014-1R/Y Chimera	2	2020	Dalhart	4	20DAL-C	151	Red
ATTX961014-1R/Y Chimera	1	2020	Dalhart	18	20DAL-R	74	Red
ATTX961014-1R/Y Chimera	2	2020	Dalhart	18	20DAL-R	87	Red
ATTX961014-1R/Y Chimera	1	2020	Springlake	4	20SPR-C	171	Red
ATTX961014-1R/Y Chimera	2	2020	Springlake	4	20SPR-C	143	Red
ATTX961014-1R/Y Chimera	1	2020	Springlake	18	20SPR-R	73	Red
ATTX961014-1R/Y Chimera	2	2020	Springlake	18	20SPR-R	87	Red
ATTX961014-1R/Y Chimera	1	2021	Springlake	4	21SPR-C	126	Red
ATTX961014-1R/Y Chimera	2	2021	Springlake	4	21SPR-C	126	Red
ATTX961014-1R/Y Chimera	1	2021	Springlake	18	21SPR-R		Red
ATTX961014-1R/Y Chimera	2	2021	Springlake	18	21SPR-R	91	Red
ATTX96746-1R*	1	2019	Dalhart	18	19DAL-R	56	Red
ATTX96746-1R*	2	2019	Dalhart	18	19DAL-R	70	Red
ATTX96746-1R*	1	2020	Dalhart	4	20DAL-C	140	Red
ATTX96746-1R*	2	2020	Dalhart	4	20DAL-C	140	Red
ATTX96746-1R*	1	2020	Dalhart	18	20DAL-R	67	Red
ATTX96746-1R*	2	2020	Dalhart	18	20DAL-R	74	Red
ATTX96746-1R*	1	2020	Springlake	4	20SPR-C	122	Red
ATTX96746-1R*	2	2020	Springlake	4	20SPR-C	108	Red
ATTX96746-1R*	1	2020	Springlake	18	20SPR-R	52	Red
ATTX96746-1R*	2	2020	Springlake	18	20SPR-R	52	Red
ATTX96746-1R*	1	2021	Springlake	4	21SPR-C	133	Red
ATTX96746-1R*	2	2021	Springlake	4	21SPR-C	126	Red
ATTX96746-1R*	1	2021	Springlake	18	21SPR-R	56	Red
ATTX96746-1R*	2	2021	Springlake	18	21SPR-R	56	Red
ATTX98444s-16R/Y	1	2019	Dalhart	18	19DAL-R	70	Red

ATTX98444s-16R/Y	2	2019	Dalhart	18	19DAL-R	70	Red
ATTX98444s-16R/Y	1	2020	Dalhart	4	20DAL-C	140	Red
ATTX98444s-16R/Y	2	2020	Dalhart	4	20DAL-C	151	Red
ATTX98444s-16R/Y	1	2020	Dalhart	18	20DAL-R	74	Red
ATTX98444s-16R/Y	2	2020	Dalhart	18	20DAL-R	95	Red
ATTX98444s-16R/Y	1	2020	Springlake	4	20SPR-C	108	Red
ATTX98444s-16R/Y	2	2020	Springlake	4	20SPR-C	108	Red
ATTX98444s-16R/Y	1	2020	Springlake	18	20SPR-R	73	Red
ATTX98444s-16R/Y	2	2020	Springlake	18	20SPR-R	66	Red
ATTX98444s-16R/Y	1	2021	Springlake	4	21SPR-C	126	Red
ATTX98444s-16R/Y	2	2021	Springlake	4	21SPR-C	119	Red
ATTX98444s-16R/Y	1	2021	Springlake	18	21SPR-R	91	Red
ATTX98444s-16R/Y	2	2021	Springlake	18	21SPR-R	70	Red
ATTX98448-6R/Y	1	2019	Dalhart	18	19DAL-R	63	Red
ATTX98448-6R/Y	2	2019	Dalhart	18	19DAL-R	91	Red
ATTX98448-6R/Y	1	2020	Dalhart	4	20DAL-C		Red
ATTX98448-6R/Y	2	2020	Dalhart	4	20DAL-C		Red
ATTX98448-6R/Y	1	2020	Dalhart	18	20DAL-R	53	Red
ATTX98448-6R/Y	2	2020	Dalhart	18	20DAL-R	53	Red
ATTX98448-6R/Y	1	2020	Springlake	4	20SPR-C	115	Red
ATTX98448-6R/Y	2	2020	Springlake	4	20SPR-C	101	Red
ATTX98448-6R/Y	1	2020	Springlake	18	20SPR-R	45	Red
ATTX98448-6R/Y	2	2020	Springlake	18	20SPR-R	38	Red
ATTX98448-6R/Y	1	2021	Springlake	4	21SPR-C	126	Red
ATTX98448-6R/Y	2	2021	Springlake	4	21SPR-C	112	Red
ATTX98448-6R/Y	1	2021	Springlake	18	21SPR-R	56	Red
ATTX98448-6R/Y	2	2021	Springlake	18	21SPR-R	49	Red
ATTX98453-11Br	1	2019	Dalhart	18	19DAL-R	84	Red
ATTX98453-11Br	2	2019	Dalhart	18	19DAL-R	105	Red
ATTX98453-11BR	1	2020	Dalhart	4	20DAL-C	165	Red
ATTX98453-11BR	2	2020	Dalhart	4	20DAL-C	193	Red
ATTX98453-11Br	1	2020	Dalhart	18	20DAL-R	74	Red
ATTX98453-11Br	2	2020	Dalhart	18	20DAL-R	95	Red
ATTX98453-11BR	1	2020	Springlake	4	20SPR-C	143	Red
ATTX98453-11BR	2	2020	Springlake	4	20SPR-C	136	Red
ATTX98453-11Br	1	2020	Springlake	18	20SPR-R	59	Red
ATTX98453-11Br	2	2020	Springlake	18	20SPR-R	73	Red
ATTX98453-11BR	1	2021	Springlake	4	21SPR-C		Red
ATTX98453-11BR	2	2021	Springlake	4	21SPR-C	161	Red
ATTX98453-11BR	1	2021	Springlake	18	21SPR-R	98	Red
ATTX98453-11BR	2	2021	Springlake	18	21SPR-R	84	Red
ATTX98453-3R	1	2019	Dalhart	18	19DAL-R	105	Red
ATTX98453-3R	2	2019	Dalhart	18	19DAL-R	91	Red
ATTX98453-3R	1	2020	Dalhart	4	20DAL-C	165	Red
ATTX98453-3R	2	2020	Dalhart	4	20DAL-C	172	Red
ATTX98453-3R	1	2020	Dalhart	18	20DAL-R	109	Red
ATTX98453-3R	2	2020	Dalhart	18	20DAL-R	109	Red

ATTX98453-3R	1	2020	Springlake	4	20SPR-C	136	Red
ATTX98453-3R	2	2020	Springlake	4	20SPR-C	122	Red
ATTX98453-3R	1	2020	Springlake	18	20SPR-R	87	Red
ATTX98453-3R	2	2020	Springlake	18	20SPR-R	80	Red
ATTX98453-3R	1	2021	Springlake	4	21SPR-C	154	Red
ATTX98453-3R	2	2021	Springlake	4	21SPR-C	126	Red
ATTX98453-3R	1	2021	Springlake	18	21SPR-R	91	Red
ATTX98453-3R	2	2021	Springlake	18	21SPR-R	98	Red
ATTX98453-6R	1	2019	Dalhart	18	19DAL-R	105	Red
ATTX98453-6R	2	2019	Dalhart	18	19DAL-R	91	Red
ATTX98453-6R	1	2020	Dalhart	4	20DAL-C	172	Red
ATTX98453-6R	2	2020	Dalhart	4	20DAL-C	165	Red
ATTX98453-6R	1	2020	Dalhart	18	20DAL-R	95	Red
ATTX98453-6R	2	2020	Dalhart	18	20DAL-R	87	Red
ATTX98453-6R	1	2020	Springlake	4	20SPR-C	150	Red
ATTX98453-6R	2	2020	Springlake	4	20SPR-C	136	Red
ATTX98453-6R	1	2020	Springlake	18	20SPR-R	66	Red
ATTX98453-6R	2	2020	Springlake	18	20SPR-R	87	Red
ATTX98453-6R	1	2021	Springlake	4	21SPR-C		Red
ATTX98453-6R	2	2021	Springlake	4	21SPR-C		Red
ATTX98453-6R	1	2021	Springlake	18	21SPR-R	119	Red
ATTX98453-6R	2	2021	Springlake	18	21SPR-R	119	Red
ATTX98462s-3R/Y	1	2019	Dalhart	18	19DAL-R	56	Red
ATTX98462s-3R/Y	2	2019	Dalhart	18	19DAL-R	63	Red
ATTX98462s-3R/Y	1	2020	Dalhart	4	20DAL-C	140	Red
ATTX98462s-3R/Y	2	2020	Dalhart	4	20DAL-C	133	Red
ATTX98462s-3R/Y	1	2020	Dalhart	18	20DAL-R	67	Red
ATTX98462s-3R/Y	2	2020	Dalhart	18	20DAL-R	67	Red
ATTX98462s-3R/Y	1	2020	Springlake	4	20SPR-C	101	Red
ATTX98462s-3R/Y	2	2020	Springlake	4	20SPR-C	101	Red
ATTX98462s-3R/Y	1	2020	Springlake	18	20SPR-R	45	Red
ATTX98462s-3R/Y	2	2020	Springlake	18	20SPR-R	52	Red
ATTX98462s-3R/Y	1	2021	Springlake	4	21SPR-C		Red
ATTX98462s-3R/Y	2	2021	Springlake	4	21SPR-C	147	Red
ATTX98462s-3R/Y	1	2021	Springlake	18	21SPR-R	63	Red
ATTX98462s-3R/Y	2	2021	Springlake	18	21SPR-R	77	Red
ATTX98465-1R/Y	1	2019	Dalhart	18	19DAL-R	70	Red
ATTX98465-1R/Y	2	2019	Dalhart	18	19DAL-R	70	Red
ATTX98465-1R/Y	1	2020	Dalhart	4	20DAL-C	151	Red
ATTX98465-1R/Y	2	2020	Dalhart	4	20DAL-C		Red
ATTX98465-1R/Y	1	2020	Dalhart	18	20DAL-R	48	Red
ATTX98465-1R/Y	2	2020	Dalhart	18	20DAL-R	53	Red
ATTX98465-1R/Y	1	2020	Springlake	4	20SPR-C	101	Red
ATTX98465-1R/Y	2	2020	Springlake	4	20SPR-C	101	Red
ATTX98465-1R/Y	1	2020	Springlake	18	20SPR-R	52	Red
ATTX98465-1R/Y	2	2020	Springlake	18	20SPR-R	45	Red
ATTX98465-1R/Y	1	2021	Springlake	4	21SPR-C	133	Red

ATTX98465-1R/Y	2	2021	Springlake	4	21SPR-C	112	Red
ATTX98465-1R/Y	1	2021	Springlake	18	21SPR-R	63	Red
ATTX98465-1R/Y	2	2021	Springlake	18	21SPR-R	56	Red
ATTX98466-5R/WR	1	2019	Dalhart	18	19DAL-R	70	Red
ATTX98466-5R/WR	2	2019	Dalhart	18	19DAL-R	91	Red
ATTX98466-5R/WR	1	2020	Dalhart	4	20DAL-C	144	Red
ATTX98466-5R/WR	2	2020	Dalhart	4	20DAL-C	165	Red
ATTX98466-5R/WR	1	2020	Dalhart	18	20DAL-R	81	Red
ATTX98466-5R/WR	2	2020	Dalhart	18	20DAL-R	74	Red
ATTX98466-5R/WR	1	2020	Springlake	4	20SPR-C	143	Red
ATTX98466-5R/WR	2	2020	Springlake	4	20SPR-C	122	Red
ATTX98466-5R/WR	1	2020	Springlake	18	20SPR-R	66	Red
ATTX98466-5R/WR	2	2020	Springlake	18	20SPR-R	59	Red
ATTX98466-5R/WR	1	2021	Springlake	4	21SPR-C	133	Red
ATTX98466-5R/WR	2	2021	Springlake	4	21SPR-C	126	Red
ATTX98466-5R/WR	1	2021	Springlake	18	21SPR-R	77	Red
ATTX98466-5R/WR	2	2021	Springlake	18	21SPR-R	77	Red
ATTX98468-5Ru/Y*	1	2019	Dalhart	18	19DAL-R	70	Russet
ATTX98468-5Ru/Y*	2	2019	Dalhart	18	19DAL-R	84	Russet
ATTX98468-5Ru/Y*	1	2020	Dalhart	4	20DAL-C	165	Russet
ATTX98468-5Ru/Y*	2	2020	Dalhart	4	20DAL-C	165	Russet
ATTX98468-5Ru/Y*	1	2020	Dalhart	18	20DAL-R	95	Russet
ATTX98468-5Ru/Y*	2	2020	Dalhart	18	20DAL-R	74	Russet
ATTX98468-5Ru/Y*	1	2020	Springlake	4	20SPR-C	95	Russet
ATTX98468-5Ru/Y*	2	2020	Springlake	4	20SPR-C	109	Russet
ATTX98468-5Ru/Y*	1	2020	Springlake	18	20SPR-R	46	Russet
ATTX98468-5Ru/Y*	2	2020	Springlake	18	20SPR-R	60	Russet
ATTX98468-5Ru/Y*	1	2021	Springlake	4	21SPR-C		Russet
ATTX98468-5Ru/Y*	2	2021	Springlake	4	21SPR-C	128	Russet
ATTX98468-5Ru/Y*	1	2021	Springlake	18	21SPR-R	63	Russet
ATTX98468-5Ru/Y*	2	2021	Springlake	18	21SPR-R	70	Russet
ATTX98491-4Yrdspl/Y	1	2019	Dalhart	18	19DAL-R	63	Red
ATTX98491-4Yrdspl/Y	2	2019	Dalhart	18	19DAL-R	70	Red
ATTX98491-4Yrdspl/Y	1	2020	Dalhart	4	20DAL-C		Red
ATTX98491-4Yrdspl/Y	2	2020	Dalhart	4	20DAL-C		Red
ATTX98491-4Yrdspl/Y	1	2020	Dalhart	18	20DAL-R	53	Red
ATTX98491-4Yrdspl/Y	2	2020	Dalhart	18	20DAL-R	53	Red
ATTX98491-4Yrdspl/Y	1	2020	Springlake	4	20SPR-C	101	Red
ATTX98491-4Yrdspl/Y	2	2020	Springlake	4	20SPR-C	94	Red
ATTX98491-4Yrdspl/Y	1	2020	Springlake	18	20SPR-R	45	Red
ATTX98491-4Yrdspl/Y	2	2020	Springlake	18	20SPR-R	52	Red
ATTX98491-4Yrdspl/Y	1	2021	Springlake	4	21SPR-C	119	Red
ATTX98491-4Yrdspl/Y	2	2021	Springlake	4	21SPR-C	126	Red
ATTX98491-4Yrdspl/Y	1	2021	Springlake	18	21SPR-R	70	Red
ATTX98491-4Yrdspl/Y	2	2021	Springlake	18	21SPR-R	63	Red
ATTX98493-1AR	1	2019	Dalhart	18	19DAL-R	77	Red
ATTX98493-1AR	2	2019	Dalhart	18	19DAL-R	77	Red

ATTX98493-1AR	1	2020	Dalhart	4	20DAL-C	140	Red
ATTX98493-1AR	2	2020	Dalhart	4	20DAL-C	140	Red
ATTX98493-1AR	1	2020	Dalhart	18	20DAL-R	74	Red
ATTX98493-1AR	2	2020	Dalhart	18	20DAL-R	81	Red
ATTX98493-1AR	1	2020	Springlake	4	20SPR-C	115	Red
ATTX98493-1AR	2	2020	Springlake	4	20SPR-C	122	Red
ATTX98493-1AR	1	2020	Springlake	18	20SPR-R	66	Red
ATTX98493-1AR	2	2020	Springlake	18	20SPR-R	59	Red
ATTX98493-1AR	1	2021	Springlake	4	21SPR-C	140	Red
ATTX98493-1AR	2	2021	Springlake	4	21SPR-C	126	Red
ATTX98493-1AR	1	2021	Springlake	18	21SPR-R	91	Red
ATTX98493-1AR	2	2021	Springlake	18	21SPR-R	84	Red
ATTX98493-2P/P	1	2019	Dalhart	18	19DAL-R	63	Purple
ATTX98493-2P/P	2	2019	Dalhart	18	19DAL-R	70	Purple
ATTX98493-2P/P	1	2020	Dalhart	4	20DAL-C	123	Purple
ATTX98493-2P/P	2	2020	Dalhart	4	20DAL-C	123	Purple
ATTX98493-2P/P	1	2020	Dalhart	18	20DAL-R	67	Purple
ATTX98493-2P/P	2	2020	Dalhart	18	20DAL-R	60	Purple
ATTX98493-2P/P	1	2020	Springlake	4	20SPR-C	122	Purple
ATTX98493-2P/P	2	2020	Springlake	4	20SPR-C	115	Purple
ATTX98493-2P/P	1	2020	Springlake	18	20SPR-R	52	Purple
ATTX98493-2P/P	2	2020	Springlake	18	20SPR-R	52	Purple
ATTX98493-2P/P	1	2021	Springlake	4	21SPR-C	140	Purple
ATTX98493-2P/P	2	2021	Springlake	4	21SPR-C	140	Purple
ATTX98493-2P/P	1	2021	Springlake	18	21SPR-R	84	Purple
ATTX98493-2P/P	2	2021	Springlake	18	21SPR-R	63	Purple
ATTX98500-2P/Y	1	2019	Dalhart	18	19DAL-R	63	Purple
ATTX98500-2P/Y	2	2019	Dalhart	18	19DAL-R	63	Purple
ATTX98500-2P/Y	1	2020	Dalhart	4	20DAL-C		Purple
ATTX98500-2P/Y	2	2020	Dalhart	4	20DAL-C		Purple
ATTX98500-2P/Y	1	2020	Dalhart	18	20DAL-R	60	Purple
ATTX98500-2P/Y	2	2020	Dalhart	18	20DAL-R	60	Purple
ATTX98500-2P/Y	1	2020	Springlake	4	20SPR-C	101	Purple
ATTX98500-2P/Y	2	2020	Springlake	4	20SPR-C	136	Purple
ATTX98500-2P/Y	1	2020	Springlake	18	20SPR-R	52	Purple
ATTX98500-2P/Y	2	2020	Springlake	18	20SPR-R	45	Purple
ATTX98500-2P/Y	1	2021	Springlake	4	21SPR-C	126	Purple
ATTX98500-2P/Y	2	2021	Springlake	4	21SPR-C	119	Purple
ATTX98500-2P/Y	1	2021	Springlake	18	21SPR-R	56	Purple
ATTX98500-2P/Y	2	2021	Springlake	18	21SPR-R	56	Purple
ATTX98500-3P/Y	1	2019	Dalhart	18	19DAL-R	70	Purple
ATTX98500-3P/Y	2	2019	Dalhart	18	19DAL-R		Purple
ATTX98500-3P/Y	1	2020	Dalhart	4	20DAL-C	172	Purple
ATTX98500-3P/Y	2	2020	Dalhart	4	20DAL-C	172	Purple
ATTX98500-3P/Y	1	2020	Dalhart	18	20DAL-R	74	Purple
ATTX98500-3P/Y	2	2020	Dalhart	18	20DAL-R	81	Purple
ATTX98500-3P/Y	1	2020	Springlake	4	20SPR-C	150	Purple

ATTX98500-3P/Y	2	2020	Springlake	4	20SPR-C		Purple
ATTX98500-3P/Y	1	2020	Springlake	18	20SPR-R	59	Purple
ATTX98500-3P/Y	2	2020	Springlake	18	20SPR-R	80	Purple
ATTX98500-3P/Y	1	2021	Springlake	4	21SPR-C	161	Purple
ATTX98500-3P/Y	2	2021	Springlake	4	21SPR-C	181	Purple
ATTX98500-3P/Y	1	2021	Springlake	18	21SPR-R	119	Purple
ATTX98500-3P/Y	2	2021	Springlake	18	21SPR-R	105	Purple
ATTX98510-1R/Y	1	2019	Dalhart	18	19DAL-R	77	Red
ATTX98510-1R/Y	2	2019	Dalhart	18	19DAL-R	63	Red
ATTX98510-1R/Y	1	2020	Dalhart	4	20DAL-C	140	Red
ATTX98510-1R/Y	2	2020	Dalhart	4	20DAL-C	140	Red
ATTX98510-1R/Y	1	2020	Dalhart	18	20DAL-R	74	Red
ATTX98510-1R/Y	2	2020	Dalhart	18	20DAL-R	53	Red
ATTX98510-1R/Y	1	2020	Springlake	4	20SPR-C	122	Red
ATTX98510-1R/Y	2	2020	Springlake	4	20SPR-C	101	Red
ATTX98510-1R/Y	1	2020	Springlake	18	20SPR-R	52	Red
ATTX98510-1R/Y	2	2020	Springlake	18	20SPR-R	59	Red
ATTX98510-1R/Y	1	2021	Springlake	4	21SPR-C	119	Red
ATTX98510-1R/Y	2	2021	Springlake	4	21SPR-C	133	Red
ATTX98510-1R/Y	1	2021	Springlake	18	21SPR-R	56	Red
ATTX98510-1R/Y	2	2021	Springlake	18	21SPR-R	70	Red
ATTX98514-1R/Y	1	2019	Dalhart	18	19DAL-R	70	Red
ATTX98514-1R/Y	2	2019	Dalhart	18	19DAL-R	77	Red
ATTX98514-1R/Y	1	2020	Dalhart	4	20DAL-C	140	Red
ATTX98514-1R/Y	2	2020	Dalhart	4	20DAL-C	140	Red
ATTX98514-1R/Y	1	2020	Dalhart	18	20DAL-R	81	Red
ATTX98514-1R/Y	2	2020	Dalhart	18	20DAL-R	60	Red
ATTX98514-1R/Y	1	2020	Springlake	4	20SPR-C	108	Red
ATTX98514-1R/Y	2	2020	Springlake	4	20SPR-C	94	Red
ATTX98514-1R/Y	1	2020	Springlake	18	20SPR-R	66	Red
ATTX98514-1R/Y	2	2020	Springlake	18	20SPR-R	45	Red
ATTX98514-1R/Y	1	2021	Springlake	4	21SPR-C	112	Red
ATTX98514-1R/Y	2	2021	Springlake	4	21SPR-C	112	Red
ATTX98514-1R/Y	1	2021	Springlake	18	21SPR-R	63	Red
ATTX98514-1R/Y	2	2021	Springlake	18	21SPR-R	77	Red
ATTX98518-5P/Y	1	2019	Dalhart	18	19DAL-R	70	Purple
ATTX98518-5P/Y	2	2019	Dalhart	18	19DAL-R	70	Purple
ATTX98518-5P/Y	1	2020	Dalhart	4	20DAL-C	123	Purple
ATTX98518-5P/Y	2	2020	Dalhart	4	20DAL-C	140	Purple
ATTX98518-5P/Y	1	2020	Dalhart	18	20DAL-R	81	Purple
ATTX98518-5P/Y	2	2020	Dalhart	18	20DAL-R	74	Purple
ATTX98518-5P/Y	1	2020	Springlake	4	20SPR-C	129	Purple
ATTX98518-5P/Y	2	2020	Springlake	4	20SPR-C	136	Purple
ATTX98518-5P/Y	1	2020	Springlake	18	20SPR-R	66	Purple
ATTX98518-5P/Y	2	2020	Springlake	18	20SPR-R	66	Purple
ATTX98518-5P/Y	1	2021	Springlake	4	21SPR-C	119	Purple
ATTX98518-5P/Y	2	2021	Springlake	4	21SPR-C	112	Purple

ATTX98518-5P/Y	1	2021	Springlake	18	21SPR-R	70	Purple
ATTX98518-5P/Y	2	2021	Springlake	18	21SPR-R	70	Purple
ATTX99325-1P	1	2019	Dalhart	18	19DAL-R	56	Purple
ATTX99325-1P	2	2019	Dalhart	18	19DAL-R	63	Purple
ATTX99325-1P	1	2020	Dalhart	4	20DAL-C	151	Purple
ATTX99325-1P	2	2020	Dalhart	4	20DAL-C	200	Purple
ATTX99325-1P	1	2020	Dalhart	18	20DAL-R	60	Purple
ATTX99325-1P	2	2020	Dalhart	18	20DAL-R	60	Purple
ATTX99325-1P	1	2020	Springlake	4	20SPR-C	143	Purple
ATTX99325-1P	2	2020	Springlake	4	20SPR-C	178	Purple
ATTX99325-1P	1	2020	Springlake	18	20SPR-R	59	Purple
ATTX99325-1P	2	2020	Springlake	18	20SPR-R	38	Purple
ATTX99325-1P	1	2021	Springlake	4	21SPR-C	168	Purple
ATTX99325-1P	2	2021	Springlake	4	21SPR-C	126	Purple
ATTX99325-1P	1	2021	Springlake	18	21SPR-R	56	Purple
ATTX99325-1P	2	2021	Springlake	18	21SPR-R	56	Purple
ATX02263-1R/Y	1	2019	Dalhart	18	19DAL-R	63	Red
ATX02263-1R/Y	2	2019	Dalhart	18	19DAL-R	70	Red
ATX02263-1R/Y	1	2020	Dalhart	4	20DAL-C	165	Red
ATX02263-1R/Y	2	2020	Dalhart	4	20DAL-C		Red
ATX02263-1R/Y	1	2020	Dalhart	18	20DAL-R	95	Red
ATX02263-1R/Y	2	2020	Dalhart	18	20DAL-R	102	Red
ATX02263-1R/Y	1	2020	Springlake	4	20SPR-C	171	Red
ATX02263-1R/Y	2	2020	Springlake	4	20SPR-C	164	Red
ATX02263-1R/Y	1	2020	Springlake	18	20SPR-R	73	Red
ATX02263-1R/Y	2	2020	Springlake	18	20SPR-R	73	Red
ATX02263-1R/Y	1	2021	Springlake	4	21SPR-C	168	Red
ATX02263-1R/Y	2	2021	Springlake	4	21SPR-C	174	Red
ATX02263-1R/Y	1	2021	Springlake	18	21SPR-R	91	Red
ATX02263-1R/Y	2	2021	Springlake	18	21SPR-R	98	Red
ATX03496-3Y/Y	1	2019	Dalhart	18	19DAL-R	70	Yellow
ATX03496-3Y/Y	2	2019	Dalhart	18	19DAL-R	63	Yellow
ATX03496-3Y/Y	1	2020	Dalhart	4	20DAL-C	116	Yellow
ATX03496-3Y/Y	2	2020	Dalhart	4	20DAL-C	133	Yellow
ATX03496-3Y/Y	1	2020	Dalhart	18	20DAL-R	74	Yellow
ATX03496-3Y/Y	2	2020	Dalhart	18	20DAL-R	74	Yellow
ATX03496-3Y/Y	1	2020	Springlake	4	20SPR-C	115	Yellow
ATX03496-3Y/Y	2	2020	Springlake	4	20SPR-C	94	Yellow
ATX03496-3Y/Y	1	2020	Springlake	18	20SPR-R	52	Yellow
ATX03496-3Y/Y	2	2020	Springlake	18	20SPR-R	45	Yellow
ATX03496-3Y/Y	1	2021	Springlake	4	21SPR-C	112	Yellow
ATX03496-3Y/Y	2	2021	Springlake	4	21SPR-C	112	Yellow
ATX03496-3Y/Y	1	2021	Springlake	18	21SPR-R	63	Yellow
ATX03496-3Y/Y	2	2021	Springlake	18	21SPR-R	77	Yellow
ATX03564-1Y/Y	1	2019	Dalhart	18	19DAL-R	63	Yellow
ATX03564-1Y/Y	2	2019	Dalhart	18	19DAL-R	63	Yellow
ATX03564-1Y/Y	1	2020	Dalhart	4	20DAL-C	116	Yellow

ATX03564-1Y/Y	2	2020	Dalhart		4	20DAL-C	133	Yellow
ATX03564-1Y/Y	1	2020	Dalhart		18	20DAL-R	74	Yellow
ATX03564-1Y/Y	2	2020	Dalhart		18	20DAL-R	81	Yellow
ATX03564-1Y/Y	1	2020	Springlake		4	20SPR-C	101	Yellow
ATX03564-1Y/Y	2	2020	Springlake		4	20SPR-C	87	Yellow
ATX03564-1Y/Y	1	2020	Springlake		18	20SPR-R	38	Yellow
ATX03564-1Y/Y	2	2020	Springlake		18	20SPR-R	38	Yellow
ATX03564-1Y/Y	1	2021	Springlake		4	21SPR-C	119	Yellow
ATX03564-1Y/Y	2	2021	Springlake		4	21SPR-C	126	Yellow
ATX03564-1Y/Y	1	2021	Springlake		18	21SPR-R	70	Yellow
ATX03564-1Y/Y	2	2021	Springlake		18	21SPR-R	84	Yellow
ATX05186-1R	1	2019	Dalhart		18	19DAL-R	63	Red
ATX05186-1R	2	2019	Dalhart		18	19DAL-R	91	Red
ATX05186-1R	1	2020	Dalhart	4		20DAL-C	140	Red
ATX05186-1R	2	2020	Dalhart	4		20DAL-C		Red
ATX05186-1R	1	2020	Dalhart		18	20DAL-R	87	Red
ATX05186-1R	2	2020	Dalhart		18	20DAL-R	87	Red
ATX05186-1R	1	2020	Springlake	4		20SPR-C	108	Red
ATX05186-1R	2	2020	Springlake	4		20SPR-C	108	Red
ATX05186-1R	1	2020	Springlake		18	20SPR-R	73	Red
ATX05186-1R	2	2020	Springlake		18	20SPR-R	45	Red
ATX05186-1R	1	2021	Springlake	4		21SPR-C	147	Red
ATX05186-1R	2	2021	Springlake	4		21SPR-C	133	Red
ATX05186-1R	1	2021	Springlake	18		21SPR-R	84	Red
ATX05186-1R	2	2021	Springlake	18		21SPR-R	70	Red
ATX05202s-3W/Y	1	2019	Dalhart		18	19DAL-R	91	Yellow
ATX05202s-3W/Y	2	2019	Dalhart		18	19DAL-R	98	Yellow
ATX05202s-3W/Y	1	2020	Dalhart		4	20DAL-C	151	Yellow
ATX05202s-3W/Y	2	2020	Dalhart		4	20DAL-C	158	Yellow
ATX05202s-3W/Y	1	2020	Dalhart		18	20DAL-R	95	Yellow
ATX05202s-3W/Y	2	2020	Dalhart		18	20DAL-R	102	Yellow
ATX05202s-3W/Y	1	2020	Springlake		4	20SPR-C	122	Yellow
ATX05202s-3W/Y	2	2020	Springlake		4	20SPR-C	101	Yellow
ATX05202s-3W/Y	1	2020	Springlake		18	20SPR-R	80	Yellow
ATX05202s-3W/Y	2	2020	Springlake		18	20SPR-R	73	Yellow
ATX05202s-3W/Y	1	2021	Springlake		4	21SPR-C	154	Yellow
ATX05202s-3W/Y	2	2021	Springlake		4	21SPR-C	126	Yellow
ATX05202s-3W/Y	1	2021	Springlake		18	21SPR-R	119	Yellow
ATX05202s-3W/Y	2	2021	Springlake		18	21SPR-R	98	Yellow
ATX06264s-4R/Y	1	2019	Dalhart		18	19DAL-R	63	Red
ATX06264s-4R/Y	2	2019	Dalhart		18	19DAL-R	56	Red
ATX06264s-4R/Y	1	2020	Dalhart		4	20DAL-C		Red
ATX06264s-4R/Y	2	2020	Dalhart		4	20DAL-C		Red
ATX06264s-4R/Y	1	2020	Dalhart		18	20DAL-R	60	Red
ATX06264s-4R/Y	2	2020	Dalhart		18	20DAL-R	60	Red
ATX06264s-4R/Y	1	2020	Springlake		4	20SPR-C	108	Red
ATX06264s-4R/Y	2	2020	Springlake		4	20SPR-C	108	Red

ATX06264s-4R/Y	1	2020	Springlake	18	20SPR-R	45	Red
ATX06264s-4R/Y	2	2020	Springlake	18	20SPR-R	59	Red
ATX06264s-4R/Y	1	2021	Springlake	4	21SPR-C	112	Red
ATX06264s-4R/Y	2	2021	Springlake	4	21SPR-C	119	Red
ATX06264s-4R/Y	1	2021	Springlake	18	21SPR-R	42	Red
ATX06264s-4R/Y	2	2021	Springlake	18	21SPR-R	56	Red
ATX07305S-1Y/Y	1	2019	Dalhart	18	19DAL-R	98	Yellow
ATX07305S-1Y/Y	2	2019	Dalhart	18	19DAL-R	84	Yellow
ATX07305s-1Y/Y	1	2020	Dalhart	4	20DAL-C	116	Yellow
ATX07305s-1Y/Y	2	2020	Dalhart	4	20DAL-C		Yellow
ATX07305S-1Y/Y	1	2020	Dalhart	18	20DAL-R	81	Yellow
ATX07305S-1Y/Y	2	2020	Dalhart	18	20DAL-R	87	Yellow
ATX07305s-1Y/Y	1	2020	Springlake	4	20SPR-C	122	Yellow
ATX07305s-1Y/Y	2	2020	Springlake	4	20SPR-C	101	Yellow
ATX07305S-1Y/Y	1	2020	Springlake	18	20SPR-R	45	Yellow
ATX07305S-1Y/Y	2	2020	Springlake	18	20SPR-R	52	Yellow
ATX07305s-1Y/Y	1	2021	Springlake	4	21SPR-C	133	Yellow
ATX07305s-1Y/Y	2	2021	Springlake	4	21SPR-C	133	Yellow
ATX07305s-1Y/Y	1	2021	Springlake	18	21SPR-R	105	Yellow
ATX07305s-1Y/Y	2	2021	Springlake	18	21SPR-R	105	Yellow
ATX08181-5Y/Y	1	2019	Dalhart	18	19DAL-R	91	Yellow
ATX08181-5Y/Y	2	2019	Dalhart	18	19DAL-R	84	Yellow
ATX08181-5Y/Y	1	2020	Dalhart	4	20DAL-C	133	Yellow
ATX08181-5Y/Y	2	2020	Dalhart	4	20DAL-C	133	Yellow
ATX08181-5Y/Y	1	2020	Dalhart	18	20DAL-R	102	Yellow
ATX08181-5Y/Y	2	2020	Dalhart	18	20DAL-R	102	Yellow
ATX08181-5Y/Y	1	2020	Springlake	4	20SPR-C	115	Yellow
ATX08181-5Y/Y	2	2020	Springlake	4	20SPR-C	101	Yellow
ATX08181-5Y/Y	1	2020	Springlake	18	20SPR-R	80	Yellow
ATX08181-5Y/Y	2	2020	Springlake	18	20SPR-R	80	Yellow
ATX08181-5Y/Y	1	2021	Springlake	4	21SPR-C	119	Yellow
ATX08181-5Y/Y	2	2021	Springlake	4	21SPR-C	112	Yellow
ATX08181-5Y/Y	1	2021	Springlake	18	21SPR-R	98	Yellow
ATX08181-5Y/Y	2	2021	Springlake	18	21SPR-R	105	Yellow
ATX11549-1P/Y	1	2022	Dalhart	4	22DAL-C	137	Purple
ATX11549-1P/Y	2	2022	Dalhart	4	22DAL-C	151	Purple
ATX11549-1P/Y	1	2022	Dalhart	18	22DAL-R	113	Purple
ATX11549-1P/Y	2	2022	Dalhart	18	22DAL-R	113	Purple
ATX11549-1P/Y	1	2022	Springlake	4	22SPR-C	118	Purple
ATX11549-1P/Y	2	2022	Springlake	4	22SPR-C	111	Purple
ATX11549-1P/Y	1	2022	Springlake	18	22SPR-R	80	Purple
ATX11549-1P/Y	2	2022	Springlake	18	22SPR-R	101	Purple
ATX11586-2R/Y	1	2022	Dalhart	4	22DAL-C	116	Red
ATX11586-2R/Y	2	2022	Dalhart	4	22DAL-C	116	Red
ATX11586-2R/Y	1	2022	Dalhart	18	22DAL-R	85	Red
ATX11586-2R/Y	2	2022	Dalhart	18	22DAL-R	85	Red
ATX11586-2R/Y	1	2022	Springlake	4	22SPR-C	111	Red

ATX11586-2R/Y	2	2022	Springlake	4	22SPR-C		Red
ATX11586-2R/Y	1	2022	Springlake	18	22SPR-R	66	Red
ATX11586-2R/Y	2	2022	Springlake	18	22SPR-R	73	Red
ATX11684f-2W/Y	1	2022	Dalhart	4	22DAL-C		Yellow
ATX11684f-2W/Y	2	2022	Dalhart	4	22DAL-C	123	Yellow
ATX11684f-2W/Y	1	2022	Dalhart	18	22DAL-R	85	Yellow
ATX11684f-2W/Y	2	2022	Dalhart	18	22DAL-R	78	Yellow
ATX11684f-2W/Y	1	2022	Springlake	4	22SPR-C	111	Yellow
ATX11684f-2W/Y	2	2022	Springlake	4	22SPR-C	83	Yellow
ATX11684f-2W/Y	1	2022	Springlake	18	22SPR-R	46	Yellow
ATX11684f-2W/Y	2	2022	Springlake	18	22SPR-R	46	Yellow
ATX13134-3W/Y	1	2022	Dalhart	4	22DAL-C	123	Chipper
ATX13134-3W/Y	2	2022	Dalhart	4	22DAL-C	116	Chipper
ATX13134-3W/Y	1	2022	Dalhart	18	22DAL-R	78	Chipper
ATX13134-3W/Y	2	2022	Dalhart	18	22DAL-R	92	Chipper
ATX13134-3W/Y	1	2022	Springlake	4	22SPR-C	81	Chipper
ATX13134-3W/Y	2	2022	Springlake	4	22SPR-C	79	Chipper
ATX13134-3W/Y	1	2022	Springlake	18	22SPR-R	57	Chipper
ATX13134-3W/Y	2	2022	Springlake	18	22SPR-R	48	Chipper
ATX15097-1Ru	1	2022	Dalhart	4	22DAL-C	122	Russet
ATX15097-1Ru	2	2022	Dalhart	4	22DAL-C	122	Russet
ATX15097-1Ru	1	2022	Dalhart	18	22DAL-R	56	Russet
ATX15097-1Ru	2	2022	Dalhart	18	22DAL-R	70	Russet
ATX15097-1Ru	1	2022	Springlake	4	22SPR-C	99	Russet
ATX15097-1Ru	2	2022	Springlake	4	22SPR-C	93	Russet
ATX15097-1Ru	1	2022	Springlake	18	22SPR-R	47	Russet
ATX15097-1Ru	2	2022	Springlake	18	22SPR-R	28	Russet
ATX15120-1Ru	1	2022	Dalhart	4	22DAL-C	143	Russet
ATX15120-1Ru	2	2022	Dalhart	4	22DAL-C	122	Russet
ATX15120-1Ru	1	2022	Dalhart	18	22DAL-R	91	Russet
ATX15120-1Ru	2	2022	Dalhart	18	22DAL-R	98	Russet
ATX15120-1Ru	1	2022	Springlake	4	22SPR-C	106	Russet
ATX15120-1Ru	2	2022	Springlake	4	22SPR-C	93	Russet
ATX15120-1Ru	1	2022	Springlake	18	22SPR-R	75	Russet
ATX15120-1Ru	2	2022	Springlake	18	22SPR-R	69	Russet
ATX15325-3W/Y	1	2022	Dalhart	4	22DAL-C	95	Yellow
ATX15325-3W/Y	2	2022	Dalhart	4	22DAL-C	95	Yellow
ATX15325-3W/Y	1	2022	Dalhart	18	22DAL-R	67	Yellow
ATX15325-3W/Y	2	2022	Dalhart	18	22DAL-R	67	Yellow
ATX15325-3W/Y	1	2022	Springlake	4	22SPR-C	90	Yellow
ATX15325-3W/Y	2	2022	Springlake	4	22SPR-C	62	Yellow
ATX15325-3W/Y	1	2022	Springlake	18	22SPR-R	59	Yellow
ATX15325-3W/Y	2	2022	Springlake	18	22SPR-R	59	Yellow
ATX16089-4Ru	1	2022	Dalhart	4	22DAL-C	136	Russet
ATX16089-4Ru	2	2022	Dalhart	4	22DAL-C	122	Russet
ATX16089-4Ru	1	2022	Dalhart	18	22DAL-R	70	Russet
ATX16089-4Ru	2	2022	Dalhart	18	22DAL-R	91	Russet

ATX16089-4Ru	1	2022	Springlake	4	22SPR-C	99	Russet
ATX16089-4Ru	2	2022	Springlake	4	22SPR-C	121	Russet
ATX16089-4Ru	1	2022	Springlake	18	22SPR-R	61	Russet
ATX16089-4Ru	2	2022	Springlake	18	22SPR-R	62	Russet
ATX84378-6Ru	1	2019	Dalhart	18	19DAL-R	91	Russet
ATX84378-6Ru	2	2019	Dalhart	18	19DAL-R	105	Russet
ATX84378-6Ru	1	2020	Dalhart	4	20DAL-C	165	Russet
ATX84378-6Ru	2	2020	Dalhart	4	20DAL-C	158	Russet
ATX84378-6Ru	1	2020	Dalhart	18	20DAL-R	95	Russet
ATX84378-6Ru	2	2020	Dalhart	18	20DAL-R	81	Russet
ATX84378-6Ru	1	2020	Springlake	4	20SPR-C	123	Russet
ATX84378-6Ru	2	2020	Springlake	4	20SPR-C	116	Russet
ATX84378-6Ru	1	2020	Springlake	18	20SPR-R	67	Russet
ATX84378-6Ru	2	2020	Springlake	18	20SPR-R	81	Russet
ATX84378-6Ru	1	2021	Springlake	4	21SPR-C	114	Russet
ATX84378-6Ru	2	2021	Springlake	4	21SPR-C	107	Russet
ATX84378-6Ru	1	2021	Springlake	18	21SPR-R	91	Russet
ATX84378-6Ru	2	2021	Springlake	18	21SPR-R	70	Russet
ATX84706-2Ru	1	2019	Dalhart	18	19DAL-R	98	Russet
ATX84706-2Ru	2	2019	Dalhart	18	19DAL-R	112	Russet
ATX84706-2Ru	1	2020	Dalhart	4	20DAL-C	133	Russet
ATX84706-2Ru	2	2020	Dalhart	4	20DAL-C	165	Russet
ATX84706-2Ru	1	2020	Dalhart	18	20DAL-R	117	Russet
ATX84706-2Ru	2	2020	Dalhart	18	20DAL-R	123	Russet
ATX84706-2Ru	1	2020	Springlake	4	20SPR-C	123	Russet
ATX84706-2Ru	2	2020	Springlake	4	20SPR-C	137	Russet
ATX84706-2Ru	1	2020	Springlake	18	20SPR-R	95	Russet
ATX84706-2Ru	2	2020	Springlake	18	20SPR-R	89	Russet
ATX84706-2Ru	1	2021	Springlake	4	21SPR-C		Russet
ATX84706-2Ru	2	2021	Springlake	4	21SPR-C	128	Russet
ATX84706-2Ru	1	2021	Springlake	18	21SPR-R	126	Russet
ATX84706-2Ru	2	2021	Springlake	18	21SPR-R	105	Russet
ATX85404-8W	1	2019	Dalhart	18	19DAL-R	91	Russet
ATX85404-8W	2	2019	Dalhart	18	19DAL-R		Russet
ATX85404-8W	1	2020	Dalhart	4	20DAL-C	151	Russet
ATX85404-8W	2	2020	Dalhart	4	20DAL-C		Russet
ATX85404-8W	1	2020	Dalhart	18	20DAL-R	95	Russet
ATX85404-8W	2	2020	Dalhart	18	20DAL-R		Russet
ATX85404-8W	1	2020	Springlake	4	20SPR-C		Russet
ATX85404-8W	2	2020	Springlake	4	20SPR-C		Russet
ATX85404-8W	1	2020	Springlake	18	20SPR-R		Russet
ATX85404-8W	2	2020	Springlake	18	20SPR-R		Russet
ATX85404-8W	1	2021	Springlake	4	21SPR-C	114	Russet
ATX85404-8W	2	2021	Springlake	4	21SPR-C	128	Russet
ATX85404-8W	1	2021	Springlake	18	21SPR-R	105	Russet
ATX85404-8W	2	2021	Springlake	18	21SPR-R	84	Russet
ATX87184-2Ru	1	2019	Dalhart	18	19DAL-R	98	Russet

ATX87184-2Ru	2	2019	Dalhart	18	19DAL-R	112	Russet
ATX87184-2Ru	1	2020	Dalhart	4	20DAL-C	140	Russet
ATX87184-2Ru	2	2020	Dalhart	4	20DAL-C	133	Russet
ATX87184-2Ru	1	2020	Dalhart	18	20DAL-R	95	Russet
ATX87184-2Ru	2	2020	Dalhart	18	20DAL-R	81	Russet
ATX87184-2Ru	1	2020	Springlake	4	20SPR-C	95	Russet
ATX87184-2Ru	2	2020	Springlake	4	20SPR-C	109	Russet
ATX87184-2Ru	1	2020	Springlake	18	20SPR-R	74	Russet
ATX87184-2Ru	2	2020	Springlake	18	20SPR-R	89	Russet
ATX87184-2Ru	1	2021	Springlake	4	21SPR-C	121	Russet
ATX87184-2Ru	2	2021	Springlake	4	21SPR-C	107	Russet
ATX87184-2Ru	1	2021	Springlake	18	21SPR-R	91	Russet
ATX87184-2Ru	2	2021	Springlake	18	21SPR-R	91	Russet
ATX9117-1Ru	1	2019	Dalhart	18	19DAL-R	77	Russet
ATX9117-1Ru	2	2019	Dalhart	18	19DAL-R	70	Russet
ATX9117-1Ru	1	2020	Dalhart	4	20DAL-C	172	Russet
ATX9117-1Ru	2	2020	Dalhart	4	20DAL-C	158	Russet
ATX9117-1Ru	1	2020	Dalhart	18	20DAL-R	74	Russet
ATX9117-1Ru	2	2020	Dalhart	18	20DAL-R	74	Russet
ATX9117-1Ru	1	2020	Springlake	4	20SPR-C	123	Russet
ATX9117-1Ru	2	2020	Springlake	4	20SPR-C	102	Russet
ATX9117-1Ru	1	2020	Springlake	18	20SPR-R	67	Russet
ATX9117-1Ru	2	2020	Springlake	18	20SPR-R	60	Russet
ATX9117-1Ru	1	2021	Springlake	4	21SPR-C	142	Russet
ATX9117-1Ru	2	2021	Springlake	4	21SPR-C	128	Russet
ATX9117-1Ru	1	2021	Springlake	18	21SPR-R	63	Russet
ATX9117-1Ru	2	2021	Springlake	18	21SPR-R	70	Russet
ATX9130-1Ru	1	2019	Dalhart	18	19DAL-R	84	Russet
ATX9130-1Ru	2	2019	Dalhart	18	19DAL-R	84	Russet
ATX9130-1Ru	1	2020	Dalhart	4	20DAL-C		Russet
ATX9130-1Ru	2	2020	Dalhart	4	20DAL-C	172	Russet
ATX9130-1Ru	1	2020	Dalhart	18	20DAL-R	81	Russet
ATX9130-1Ru	2	2020	Dalhart	18	20DAL-R	81	Russet
ATX9130-1Ru	1	2020	Springlake	4	20SPR-C	123	Russet
ATX9130-1Ru	2	2020	Springlake	4	20SPR-C	116	Russet
ATX9130-1Ru	1	2020	Springlake	18	20SPR-R	67	Russet
ATX9130-1Ru	2	2020	Springlake	18	20SPR-R	60	Russet
ATX9130-1Ru	1	2021	Springlake	4	21SPR-C	114	Russet
ATX9130-1Ru	2	2021	Springlake	4	21SPR-C	114	Russet
ATX9130-1Ru	1	2021	Springlake	18	21SPR-R	63	Russet
ATX9130-1Ru	2	2021	Springlake	18	21SPR-R	70	Russet
ATX9202-3Ru	1	2019	Dalhart	18	19DAL-R	119	Russet
ATX9202-3Ru	2	2019	Dalhart	18	19DAL-R	126	Russet
ATX9202-3Ru	1	2020	Dalhart	4	20DAL-C	207	Russet
ATX9202-3Ru	2	2020	Dalhart	4	20DAL-C	207	Russet
ATX9202-3Ru	1	2020	Dalhart	18	20DAL-R	109	Russet
ATX9202-3Ru	2	2020	Dalhart	18	20DAL-R	129	Russet

ATX9202-3Ru	1	2020	Springlake	4	20SPR-C		Russet
ATX9202-3Ru	2	2020	Springlake	4	20SPR-C		Russet
ATX9202-3Ru	1	2020	Springlake	18	20SPR-R		Russet
ATX9202-3Ru	2	2020	Springlake	18	20SPR-R		Russet
ATX9202-3Ru	1	2021	Springlake	4	21SPR-C	156	Russet
ATX9202-3Ru	2	2021	Springlake	4	21SPR-C	142	Russet
ATX9202-3Ru	1	2021	Springlake	18	21SPR-R	84	Russet
ATX9202-3Ru	2	2021	Springlake	18	21SPR-R	98	Russet
ATX9312-1Ru	1	2019	Dalhart	18	19DAL-R	112	Russet
ATX9312-1Ru	2	2019	Dalhart	18	19DAL-R	91	Russet
ATX9312-1Ru	1	2020	Dalhart	4	20DAL-C	151	Russet
ATX9312-1Ru	2	2020	Dalhart	4	20DAL-C	172	Russet
ATX9312-1Ru	1	2020	Dalhart	18	20DAL-R	117	Russet
ATX9312-1Ru	2	2020	Dalhart	18	20DAL-R	117	Russet
ATX9312-1Ru	1	2020	Springlake	18	20SPR-C		Russet
ATX9312-1Ru	1	2020	Springlake	18	20SPR-R		Russet
ATX9312-1Ru	2	2020	Springlake	18	20SPR-C		Russet
ATX9312-1Ru	2	2020	Springlake	18	20SPR-R		Russet
ATX9312-1Ru	1	2021	Springlake	4	21SPR-C	121	Russet
ATX9312-1Ru	2	2021	Springlake	4	21SPR-C	107	Russet
ATX9312-1Ru	1	2021	Springlake	18	21SPR-R	105	Russet
ATX9312-1Ru	2	2021	Springlake	18	21SPR-R	119	Russet
ATX9332-8Ru	1	2019	Dalhart	18	19DAL-R	84	Russet
ATX9332-8Ru	2	2019	Dalhart	18	19DAL-R	112	Russet
ATX9332-8Ru	1	2020	Dalhart	4	20DAL-C	200	Russet
ATX9332-8Ru	2	2020	Dalhart	4	20DAL-C		Russet
ATX9332-8Ru	1	2020	Dalhart	18	20DAL-R	129	Russet
ATX9332-8Ru	2	2020	Dalhart	18	20DAL-R		Russet
ATX9332-8Ru	1	2020	Springlake	4	20SPR-C	109	Russet
ATX9332-8Ru	2	2020	Springlake	4	20SPR-C	88	Russet
ATX9332-8Ru	1	2020	Springlake	18	20SPR-R	74	Russet
ATX9332-8Ru	2	2020	Springlake	18	20SPR-R	81	Russet
ATX9332-8Ru	1	2021	Springlake	4	21SPR-C	135	Russet
ATX9332-8Ru	2	2021	Springlake	4	21SPR-C		Russet
ATX97147-4Ru	1	2019	Dalhart	18	19DAL-R	112	Russet
ATX97147-4Ru	2	2019	Dalhart	18	19DAL-R	119	Russet
ATX97147-4Ru	1	2020	Dalhart	4	20DAL-C	165	Russet
ATX97147-4Ru	2	2020	Dalhart	4	20DAL-C	179	Russet
ATX97147-4Ru	1	2020	Dalhart	18	20DAL-R	137	Russet
ATX97147-4Ru	2	2020	Dalhart	18	20DAL-R	95	Russet
ATX97147-4Ru	1	2020	Springlake	4	20SPR-C	123	Russet
ATX97147-4Ru	2	2020	Springlake	4	20SPR-C	109	Russet
ATX97147-4Ru	1	2020	Springlake	18	20SPR-R	89	Russet
ATX97147-4Ru	2	2020	Springlake	18	20SPR-R	81	Russet
ATX97147-4Ru	1	2021	Springlake	4	21SPR-C	156	Russet
ATX97147-4Ru	2	2021	Springlake	4	21SPR-C	176	Russet
ATX97147-4Ru	1	2021	Springlake	18	21SPR-R		Russet

ATX97147-4Ru	2	2021	Springlake	18	21SPR-R	91	Russet
ATX99013-1Ru	1	2019	Dalhart	18	19DAL-R	112	Russet
ATX99013-1Ru	2	2019	Dalhart	18	19DAL-R	112	Russet
ATX99013-1Ru	1	2020	Dalhart	4	20DAL-C	144	Russet
ATX99013-1Ru	2	2020	Dalhart	4	20DAL-C	165	Russet
ATX99013-1Ru	1	2020	Dalhart	18	20DAL-R	109	Russet
ATX99013-1Ru	2	2020	Dalhart	18	20DAL-R	109	Russet
ATX99013-1Ru	1	2020	Springlake	4	20SPR-C	95	Russet
ATX99013-1Ru	2	2020	Springlake	4	20SPR-C	102	Russet
ATX99013-1Ru	1	2020	Springlake	18	20SPR-R	81	Russet
ATX99013-1Ru	2	2020	Springlake	18	20SPR-R	89	Russet
ATX99013-1Ru	1	2021	Springlake	4	21SPR-C	107	Russet
ATX99013-1Ru	2	2021	Springlake	4	21SPR-C	107	Russet
ATX99013-1Ru	1	2021	Springlake	18	21SPR-R	105	Russet
ATX99013-1Ru	2	2021	Springlake	18	21SPR-R	105	Russet
Banana	1	2022	Dalhart	4	22DAL-C	165	Yellow
Banana	2	2022	Dalhart	4	22DAL-C	165	Yellow
Banana	1	2022	Dalhart	18	22DAL-R	78	Yellow
Banana	2	2022	Dalhart	18	22DAL-R	78	Yellow
Banana	1	2022	Springlake	4	22SPR-C	111	Yellow
Banana	2	2022	Springlake	4	22SPR-C	93	Yellow
Banana	1	2022	Springlake	18	22SPR-R	46	Yellow
Banana	2	2022	Springlake	18	22SPR-R	34	Yellow
BTX1544-2W/Y	1	2019	Dalhart	18	19DAL-R	77	Yellow
BTX1544-2W/Y	2	2019	Dalhart	18	19DAL-R	70	Yellow
BTX1544-2W/Y	1	2020	Dalhart	4	20DAL-C	151	Yellow
BTX1544-2W/Y	2	2020	Dalhart	4	20DAL-C	140	Yellow
BTX1544-2W/Y	1	2020	Dalhart	18	20DAL-R	95	Yellow
BTX1544-2W/Y	2	2020	Dalhart	18	20DAL-R	67	Yellow
BTX1544-2W/Y	1	2020	Springlake	4	20SPR-C	108	Yellow
BTX1544-2W/Y	2	2020	Springlake	4	20SPR-C	101	Yellow
BTX1544-2W/Y	1	2020	Springlake	18	20SPR-R	66	Yellow
BTX1544-2W/Y	2	2020	Springlake	18	20SPR-R	59	Yellow
BTX1544-2W/Y	1	2021	Springlake	4	21SPR-C	119	Yellow
BTX1544-2W/Y	2	2021	Springlake	4	21SPR-C	112	Yellow
BTX1544-2W/Y	1	2021	Springlake	18	21SPR-R	63	Yellow
BTX1544-2W/Y	2	2021	Springlake	18	21SPR-R	63	Yellow
BTX1749-1W/Y	1	2019	Dalhart	18	19DAL-R	70	Yellow
BTX1749-1W/Y	2	2019	Dalhart	18	19DAL-R	56	Yellow
BTX1749-1W/Y	1	2020	Dalhart	4	20DAL-C		Yellow
BTX1749-1W/Y	2	2020	Dalhart	4	20DAL-C		Yellow
BTX1749-1W/Y	1	2020	Dalhart	18	20DAL-R	67	Yellow
BTX1749-1W/Y	2	2020	Dalhart	18	20DAL-R	67	Yellow
BTX1749-1W/Y	1	2020	Springlake	4	20SPR-C	108	Yellow
BTX1749-1W/Y	2	2020	Springlake	4	20SPR-C	115	Yellow
BTX1749-1W/Y	1	2020	Springlake	18	20SPR-R	52	Yellow
BTX1749-1W/Y	2	2020	Springlake	18	20SPR-R	45	Yellow

BTX1749-1W/Y	1	2021	Springlake	4	21SPR-C	99	Yellow
BTX1749-1W/Y	2	2021	Springlake	4	21SPR-C	99	Yellow
BTX1749-1W/Y	1	2021	Springlake	18	21SPR-R	70	Yellow
BTX1749-1W/Y	2	2021	Springlake	18	21SPR-R	70	Yellow
BTX2103-1R/Y	1	2019	Dalhart	18	19DAL-R	70	Red
BTX2103-1R/Y	2	2019	Dalhart	18	19DAL-R	98	Red
BTX2103-1R/Y	1	2020	Dalhart	4	20DAL-C	140	Red
BTX2103-1R/Y	2	2020	Dalhart	4	20DAL-C	151	Red
BTX2103-1R/Y	1	2020	Dalhart	18	20DAL-R	74	Red
BTX2103-1R/Y	2	2020	Dalhart	18	20DAL-R	102	Red
BTX2103-1R/Y	1	2020	Springlake	4	20SPR-C		Red
BTX2103-1R/Y	2	2020	Springlake	4	20SPR-C	150	Red
BTX2103-1R/Y	1	2020	Springlake	18	20SPR-R	80	Red
BTX2103-1R/Y	2	2020	Springlake	18	20SPR-R	87	Red
BTX2103-1R/Y	1	2021	Springlake	4	21SPR-C	168	Red
BTX2103-1R/Y	2	2021	Springlake	4	21SPR-C	140	Red
BTX2103-1R/Y	1	2021	Springlake	18	21SPR-R	91	Red
BTX2103-1R/Y	2	2021	Springlake	18	21SPR-R	98	Red
BTX2332-1R	1	2019	Dalhart	18	19DAL-R	91	Red
BTX2332-1R	2	2019	Dalhart	18	19DAL-R	91	Red
BTX2332-1R	1	2020	Dalhart	4	20DAL-C	144	Red
BTX2332-1R	2	2020	Dalhart	4	20DAL-C	133	Red
BTX2332-1R	1	2020	Dalhart	18	20DAL-R	102	Red
BTX2332-1R	2	2020	Dalhart	18	20DAL-R	87	Red
BTX2332-1R	1	2020	Springlake	4	20SPR-C	136	Red
BTX2332-1R	2	2020	Springlake	4	20SPR-C	136	Red
BTX2332-1R	1	2020	Springlake	18	20SPR-R	73	Red
BTX2332-1R	2	2020	Springlake	18	20SPR-R	73	Red
BTX2332-1R	1	2021	Springlake	4	21SPR-C	133	Red
BTX2332-1R	2	2021	Springlake	4	21SPR-C	147	Red
BTX2332-1R	1	2021	Springlake	18	21SPR-R	84	Red
BTX2332-1R	2	2021	Springlake	18	21SPR-R	77	Red
Chieftain	1	2022	Dalhart	4	22DAL-C	137	Red
Chieftain	2	2022	Dalhart	4	22DAL-C	137	Red
Chieftain	1	2022	Dalhart	18	22DAL-R	85	Red
Chieftain	2	2022	Dalhart	18	22DAL-R	85	Red
Chieftain	1	2022	Springlake	4	22SPR-C	111	Red
Chieftain	2	2022	Springlake	4	22SPR-C	111	Red
Chieftain	1	2022	Springlake	18	22SPR-R	52	Red
Chieftain	2	2022	Springlake	18	22SPR-R	52	Red
Clearwater Russet	1	2022	Dalhart	4	22DAL-C	80	Russet
Clearwater Russet	2	2022	Dalhart	4	22DAL-C	108	Russet
Clearwater Russet	1	2022	Dalhart	18	22DAL-R	70	Russet
Clearwater Russet	2	2022	Dalhart	18	22DAL-R	77	Russet
Clearwater Russet	1	2022	Springlake	4	22SPR-C	78	Russet
Clearwater Russet	2	2022	Springlake	4	22SPR-C	99	Russet
Clearwater Russet	1	2022	Springlake	18	22SPR-R	34	Russet

Clearwater Russet	2	2022	Springlake	18	22SPR-R	40	Russet
CO112-F2-2P/P	1	2019	Dalhart	18	19DAL-R	126	Purple
CO112-F2-2P/P	2	2019	Dalhart	18	19DAL-R		Purple
CO112-F2-2P/P	1	2020	Dalhart	4	20DAL-C	88	Purple
CO112-F2-2P/P	2	2020	Dalhart	4	20DAL-C	88	Purple
CO112-F2-2P/P	1	2020	Dalhart	18	20DAL-R	137	Purple
CO112-F2-2P/P	2	2020	Dalhart	18	20DAL-R	137	Purple
CO112-F2-2P/P	1	2020	Springlake	4	20SPR-C	94	Purple
CO112-F2-2P/P	2	2020	Springlake	4	20SPR-C	108	Purple
CO112-F2-2P/P	1	2020	Springlake	18	20SPR-R	95	Purple
CO112-F2-2P/P	2	2020	Springlake	18	20SPR-R	121	Purple
CO112-F2-2P/P	1	2021	Springlake	4	21SPR-C	147	Purple
CO112-F2-2P/P	2	2021	Springlake	4	21SPR-C	126	Purple
CO112-F2-2P/P	1	2021	Springlake	18	21SPR-R	168	Purple
CO112-F2-2P/P	2	2021	Springlake	18	21SPR-R	154	Purple
COTX00104-6R	1	2019	Dalhart	18	19DAL-R	63	Red
COTX00104-6R	2	2019	Dalhart	18	19DAL-R	70	Red
COTX00104-6R	1	2020	Dalhart	4	20DAL-C	133	Red
COTX00104-6R	2	2020	Dalhart	4	20DAL-C	133	Red
COTX00104-6R	1	2020	Dalhart	18	20DAL-R	67	Red
COTX00104-6R	2	2020	Dalhart	18	20DAL-R	95	Red
COTX00104-6R	1	2020	Springlake	4	20SPR-C	108	Red
COTX00104-6R	2	2020	Springlake	4	20SPR-C		Red
COTX00104-6R	1	2020	Springlake	18	20SPR-R	52	Red
COTX00104-6R	2	2020	Springlake	18	20SPR-R	66	Red
COTX00104-6R	1	2021	Springlake	4	21SPR-C	112	Red
COTX00104-6R	2	2021	Springlake	4	21SPR-C	119	Red
COTX00104-6R	1	2021	Springlake	18	21SPR-R	70	Red
COTX00104-6R	2	2021	Springlake	18	21SPR-R	63	Red
COTX00104-7R	1	2019	Dalhart	18	19DAL-R	70	Red
COTX00104-7R	2	2019	Dalhart	18	19DAL-R	77	Red
COTX00104-7R	1	2020	Dalhart	4	20DAL-C		Red
COTX00104-7R	2	2020	Dalhart	4	20DAL-C	140	Red
COTX00104-7R	1	2020	Dalhart	18	20DAL-R	81	Red
COTX00104-7R	2	2020	Dalhart	18	20DAL-R	87	Red
COTX00104-7R	1	2020	Springlake	4	20SPR-C	136	Red
COTX00104-7R	2	2020	Springlake	4	20SPR-C	143	Red
COTX00104-7R	1	2020	Springlake	18	20SPR-R	80	Red
COTX00104-7R	2	2020	Springlake	18	20SPR-R	73	Red
COTX00104-7R	1	2021	Springlake	4	21SPR-C	126	Red
COTX00104-7R	2	2021	Springlake	4	21SPR-C	133	Red
COTX00104-7R	1	2021	Springlake	18	21SPR-R	98	Red
COTX00104-7R	2	2021	Springlake	18	21SPR-R	105	Red
COTX01403-4R/Y	1	2019	Dalhart	18	19DAL-R	56	Red
COTX01403-4R/Y	2	2019	Dalhart	18	19DAL-R	70	Red
COTX01403-4R/Y	1	2020	Dalhart	4	20DAL-C		Red
COTX01403-4R/Y	2	2020	Dalhart	4	20DAL-C		Red

COTX01403-4R/Y	1	2020	Dalhart	18	20DAL-R	53	Red
COTX01403-4R/Y	2	2020	Dalhart	18	20DAL-R	67	Red
COTX01403-4R/Y	1	2020	Springlake	4	20SPR-C	101	Red
COTX01403-4R/Y	2	2020	Springlake	4	20SPR-C	101	Red
COTX01403-4R/Y	1	2020	Springlake	18	20SPR-R	52	Red
COTX01403-4R/Y	2	2020	Springlake	18	20SPR-R	59	Red
COTX01403-4R/Y	1	2021	Springlake	4	21SPR-C	112	Red
COTX01403-4R/Y	2	2021	Springlake	4	21SPR-C	119	Red
COTX01403-4R/Y	1	2021	Springlake	18	21SPR-R	70	Red
COTX01403-4R/Y	2	2021	Springlake	18	21SPR-R	49	Red
COTX02172-1R	1	2019	Dalhart	18	19DAL-R	56	Red
COTX02172-1R	2	2019	Dalhart	18	19DAL-R	70	Red
COTX02172-1R	1	2020	Dalhart	4	20DAL-C	151	Red
COTX02172-1R	2	2020	Dalhart	4	20DAL-C	140	Red
COTX02172-1R	1	2020	Dalhart	18	20DAL-R	81	Red
COTX02172-1R	2	2020	Dalhart	18	20DAL-R	81	Red
COTX02172-1R	1	2020	Springlake	4	20SPR-C	122	Red
COTX02172-1R	2	2020	Springlake	4	20SPR-C	122	Red
COTX02172-1R	1	2020	Springlake	18	20SPR-R	59	Red
COTX02172-1R	2	2020	Springlake	18	20SPR-R	66	Red
COTX02172-1R	1	2021	Springlake	4	21SPR-C	112	Red
COTX02172-1R	2	2021	Springlake	4	21SPR-C	126	Red
COTX02172-1R	1	2021	Springlake	18	21SPR-R	77	Red
COTX02172-1R	2	2021	Springlake	18	21SPR-R	70	Red
COTX02293-4R	1	2019	Dalhart	18	19DAL-R	56	Red
COTX02293-4R	2	2019	Dalhart	18	19DAL-R	63	Red
COTX02293-4R	1	2020	Dalhart	4	20DAL-C	133	Red
COTX02293-4R	2	2020	Dalhart	4	20DAL-C		Red
COTX02293-4R	1	2020	Dalhart	18	20DAL-R	60	Red
COTX02293-4R	2	2020	Dalhart	18	20DAL-R	67	Red
COTX02293-4R	1	2020	Springlake	4	20SPR-C	108	Red
COTX02293-4R	2	2020	Springlake	4	20SPR-C	101	Red
COTX02293-4R	1	2020	Springlake	18	20SPR-R	52	Red
COTX02293-4R	2	2020	Springlake	18	20SPR-R	52	Red
COTX02293-4R	1	2021	Springlake	4	21SPR-C	126	Red
COTX02293-4R	2	2021	Springlake	4	21SPR-C	126	Red
COTX02293-4R	1	2021	Springlake	18	21SPR-R	56	Red
COTX02293-4R	2	2021	Springlake	18	21SPR-R	56	Red
COTX03079-1W/W	1	2019	Dalhart	18	19DAL-R	63	Yellow
COTX03079-1W/W	2	2019	Dalhart	18	19DAL-R	70	Yellow
COTX03079-1W/W	1	2020	Dalhart	4	20DAL-C	151	Yellow
COTX03079-1W/W	2	2020	Dalhart	4	20DAL-C	116	Yellow
COTX03079-1W/W	1	2020	Dalhart	18	20DAL-R	81	Yellow
COTX03079-1W/W	2	2020	Dalhart	18	20DAL-R	87	Yellow
COTX03079-1W/W	1	2020	Springlake	4	20SPR-C	101	Yellow
COTX03079-1W/W	2	2020	Springlake	4	20SPR-C	101	Yellow
COTX03079-1W/W	1	2020	Springlake	18	20SPR-R	52	Yellow

COTX03079-1W/W	2	2020	Springlake	18	20SPR-R	38	Yellow
COTX03079-1W/W	1	2021	Springlake	4	21SPR-C	133	Yellow
COTX03079-1W/W	2	2021	Springlake	4	21SPR-C	126	Yellow
COTX03079-1W/W	1	2021	Springlake	18	21SPR-R	77	Yellow
COTX03079-1W/W	2	2021	Springlake	18	21SPR-R	84	Yellow
COTX03134-1Y/W	1	2019	Dalhart	18	19DAL-R	70	Yellow
COTX03134-1Y/W	2	2019	Dalhart	18	19DAL-R	63	Yellow
COTX03134-1Y/W	1	2020	Dalhart	4	20DAL-C		Yellow
COTX03134-1Y/W	2	2020	Dalhart	4	20DAL-C	133	Yellow
COTX03134-1Y/W	1	2020	Dalhart	18	20DAL-R	67	Yellow
COTX03134-1Y/W	2	2020	Dalhart	18	20DAL-R	67	Yellow
COTX03134-1Y/W	1	2020	Springlake	4	20SPR-C		Yellow
COTX03134-1Y/W	2	2020	Springlake	4	20SPR-C		Yellow
COTX03134-1Y/W	1	2020	Springlake	18	20SPR-R		Yellow
COTX03134-1Y/W	2	2020	Springlake	18	20SPR-R		Yellow
COTX03134-1Y/W	1	2021	Springlake	4	21SPR-C	119	Yellow
COTX03134-1Y/W	2	2021	Springlake	4	21SPR-C	126	Yellow
COTX03134-1Y/W	1	2021	Springlake	18	21SPR-R	70	Yellow
COTX03134-1Y/W	2	2021	Springlake	18	21SPR-R	70	Yellow
COTX03187-1W	1	2019	Dalhart	18	19DAL-R	84	Chipper
COTX03187-1W	2	2019	Dalhart	18	19DAL-R	91	Chipper
COTX03187-1W	1	2020	Dalhart	4	20DAL-C	151	Chipper
COTX03187-1W	2	2020	Dalhart	4	20DAL-C	165	Chipper
COTX03187-1W	1	2020	Dalhart	18	20DAL-R	87	Chipper
COTX03187-1W	2	2020	Dalhart	18	20DAL-R	81	Chipper
COTX03187-1W	1	2020	Springlake	4	20SPR-C	136	Chipper
COTX03187-1W	2	2020	Springlake	4	20SPR-C	157	Chipper
COTX03187-1W	1	2020	Springlake	18	20SPR-R	73	Chipper
COTX03187-1W	2	2020	Springlake	18	20SPR-R	66	Chipper
COTX03187-1W	1	2021	Springlake	4	21SPR-C	174	Chipper
COTX03187-1W	2	2021	Springlake	4	21SPR-C	161	Chipper
COTX03187-1W	1	2021	Springlake	18	21SPR-R	98	Chipper
COTX03187-1W	2	2021	Springlake	18	21SPR-R	84	Chipper
COTX04015-3W/Y	1	2019	Dalhart	18	19DAL-R	70	Yellow
COTX04015-3W/Y	2	2019	Dalhart	18	19DAL-R	63	Yellow
COTX04015-3W/Y	1	2020	Dalhart	4	20DAL-C	133	Yellow
COTX04015-3W/Y	2	2020	Dalhart	4	20DAL-C	133	Yellow
COTX04015-3W/Y	1	2020	Dalhart	18	20DAL-R	74	Yellow
COTX04015-3W/Y	2	2020	Dalhart	18	20DAL-R	74	Yellow
COTX04015-3W/Y	1	2020	Springlake	4	20SPR-C	101	Yellow
COTX04015-3W/Y	2	2020	Springlake	4	20SPR-C	101	Yellow
COTX04015-3W/Y	1	2020	Springlake	18	20SPR-R	52	Yellow
COTX04015-3W/Y	2	2020	Springlake	18	20SPR-R	59	Yellow
COTX04015-3W/Y	1	2021	Springlake	4	21SPR-C	119	Yellow
COTX04015-3W/Y	2	2021	Springlake	4	21SPR-C	126	Yellow
COTX04015-3W/Y	1	2021	Springlake	18	21SPR-R	84	Yellow
COTX04015-3W/Y	2	2021	Springlake	18	21SPR-R	84	Yellow

COTX04050s-1P/P	1	2019	Dalhart	18	19DAL-R	84	Purple
COTX04050s-1P/P	2	2019	Dalhart	18	19DAL-R	63	Purple
COTX04050s-1P/P	1	2020	Dalhart	4	20DAL-C	123	Purple
COTX04050s-1P/P	2	2020	Dalhart	4	20DAL-C	123	Purple
COTX04050s-1P/P	1	2020	Dalhart	18	20DAL-R	74	Purple
COTX04050s-1P/P	2	2020	Dalhart	18	20DAL-R	87	Purple
COTX04050s-1P/P	1	2020	Springlake	4	20SPR-C	108	Purple
COTX04050s-1P/P	2	2020	Springlake	4	20SPR-C	129	Purple
COTX04050s-1P/P	1	2020	Springlake	18	20SPR-R	73	Purple
COTX04050s-1P/P	2	2020	Springlake	18	20SPR-R	66	Purple
COTX04050s-1P/P	1	2021	Springlake	4	21SPR-C	140	Purple
COTX04050s-1P/P	2	2021	Springlake	4	21SPR-C	112	Purple
COTX04050s-1P/P	1	2021	Springlake	18	21SPR-R	84	Purple
COTX04050s-1P/P	2	2021	Springlake	18	21SPR-R	63	Purple
COTX04193s-2R/Y	1	2019	Dalhart	18	19DAL-R	56	Red
COTX04193s-2R/Y	2	2019	Dalhart	18	19DAL-R	56	Red
COTX04193s-2R/Y	1	2020	Dalhart	4	20DAL-C	144	Red
COTX04193s-2R/Y	2	2020	Dalhart	4	20DAL-C	172	Red
COTX04193s-2R/Y	1	2020	Dalhart	18	20DAL-R	67	Red
COTX04193s-2R/Y	2	2020	Dalhart	18	20DAL-R	81	Red
COTX04193s-2R/Y	1	2020	Springlake	4	20SPR-C	136	Red
COTX04193s-2R/Y	2	2020	Springlake	4	20SPR-C	171	Red
COTX04193s-2R/Y	1	2020	Springlake	18	20SPR-R	66	Red
COTX04193s-2R/Y	2	2020	Springlake	18	20SPR-R	59	Red
COTX04193s-2R/Y	1	2021	Springlake	4	21SPR-C	174	Red
COTX04193s-2R/Y	2	2021	Springlake	4	21SPR-C	126	Red
COTX04193s-2R/Y	1	2021	Springlake	18	21SPR-R	70	Red
COTX04193s-2R/Y	2	2021	Springlake	18	21SPR-R	70	Red
COTX04303-3Ru/Y*	1	2019	Dalhart	18	19DAL-R	91	Russet
COTX04303-3Ru/Y*	2	2019	Dalhart	18	19DAL-R	112	Russet
COTX04303-3Ru/Y*	1	2020	Dalhart	4	20DAL-C	133	Russet
COTX04303-3Ru/Y*	2	2020	Dalhart	4	20DAL-C	140	Russet
COTX04303-3Ru/Y*	1	2020	Dalhart	18	20DAL-R	102	Russet
COTX04303-3Ru/Y*	2	2020	Dalhart	18	20DAL-R	102	Russet
COTX04303-3Ru/Y*	1	2020	Springlake	4	20SPR-C	109	Russet
COTX04303-3Ru/Y*	2	2020	Springlake	4	20SPR-C	95	Russet
COTX04303-3Ru/Y*	1	2020	Springlake	18	20SPR-R	81	Russet
COTX04303-3Ru/Y*	2	2020	Springlake	18	20SPR-R	89	Russet
COTX04303-3Ru/Y*	1	2021	Springlake	4	21SPR-C	114	Russet
COTX04303-3Ru/Y*	2	2021	Springlake	4	21SPR-C	107	Russet
COTX04303-3Ru/Y*	1	2021	Springlake	18	21SPR-R	91	Russet
COTX04303-3Ru/Y*	2	2021	Springlake	18	21SPR-R	105	Russet
COTX05095-2Ru/Y	1	2019	Dalhart	18	19DAL-R	70	Russet
COTX05095-2Ru/Y	2	2019	Dalhart	18	19DAL-R	77	Russet
COTX05095-2Ru/Y	1	2020	Dalhart	4	20DAL-C	151	Russet
COTX05095-2Ru/Y	2	2020	Dalhart	4	20DAL-C	140	Russet
COTX05095-2Ru/Y	1	2020	Dalhart	18	20DAL-R	81	Russet

COTX05095-2Ru/Y	2	2020	Dalhart	18	20DAL-R	81	Russet
COTX05095-2Ru/Y	1	2020	Springlake	4	20SPR-C	109	Russet
COTX05095-2Ru/Y	2	2020	Springlake	4	20SPR-C	109	Russet
COTX05095-2Ru/Y	1	2020	Springlake	18	20SPR-R	60	Russet
COTX05095-2Ru/Y	2	2020	Springlake	18	20SPR-R	67	Russet
COTX05095-2Ru/Y	1	2021	Springlake	4	21SPR-C	142	Russet
COTX05095-2Ru/Y	2	2021	Springlake	4	21SPR-C	121	Russet
COTX05095-2Ru/Y	1	2021	Springlake	18	21SPR-R	77	Russet
COTX05095-2Ru/Y	2	2021	Springlake	18	21SPR-R	77	Russet
COTX05211-4R	1	2019	Dalhart	18	19DAL-R	70	Red
COTX05211-4R	2	2019	Dalhart	18	19DAL-R	70	Red
COTX05211-4R	1	2020	Dalhart	4	20DAL-C	133	Red
COTX05211-4R	2	2020	Dalhart	4	20DAL-C	133	Red
COTX05211-4R	1	2020	Dalhart	18	20DAL-R	67	Red
COTX05211-4R	2	2020	Dalhart	18	20DAL-R	74	Red
COTX05211-4R	1	2020	Springlake	4	20SPR-C	122	Red
COTX05211-4R	2	2020	Springlake	4	20SPR-C	136	Red
COTX05211-4R	1	2020	Springlake	18	20SPR-R	52	Red
COTX05211-4R	2	2020	Springlake	18	20SPR-R	73	Red
COTX05211-4R	1	2021	Springlake	4	21SPR-C	147	Red
COTX05211-4R	2	2021	Springlake	4	21SPR-C	133	Red
COTX05211-4R	1	2021	Springlake	18	21SPR-R	91	Red
COTX05211-4R	2	2021	Springlake	18	21SPR-R	77	Red
COTX05211-5R	1	2019	Dalhart	18	19DAL-R	56	Red
COTX05211-5R	2	2019	Dalhart	18	19DAL-R	70	Red
COTX05211-5R	1	2020	Dalhart	4	20DAL-C		Red
COTX05211-5R	2	2020	Dalhart	4	20DAL-C	140	Red
COTX05211-5R	1	2020	Dalhart	18	20DAL-R	74	Red
COTX05211-5R	2	2020	Dalhart	18	20DAL-R	60	Red
COTX05211-5R	1	2020	Springlake	4	20SPR-C	129	Red
COTX05211-5R	2	2020	Springlake	4	20SPR-C	122	Red
COTX05211-5R	1	2020	Springlake	18	20SPR-R	52	Red
COTX05211-5R	2	2020	Springlake	18	20SPR-R	45	Red
COTX05211-5R	1	2021	Springlake	4	21SPR-C	147	Red
COTX05211-5R	2	2021	Springlake	4	21SPR-C	161	Red
COTX05211-5R	1	2021	Springlake	18	21SPR-R	63	Red
COTX05211-5R	2	2021	Springlake	18	21SPR-R	70	Red
COTX05211-7R	1	2019	Dalhart	18	19DAL-R	56	Red
COTX05211-7R	2	2019	Dalhart	18	19DAL-R	63	Red
COTX05211-7R	1	2020	Dalhart	4	20DAL-C	133	Red
COTX05211-7R	2	2020	Dalhart	4	20DAL-C	133	Red
COTX05211-7R	1	2020	Dalhart	18	20DAL-R	74	Red
COTX05211-7R	2	2020	Dalhart	18	20DAL-R	67	Red
COTX05211-7R	1	2020	Springlake	4	20SPR-C	129	Red
COTX05211-7R	2	2020	Springlake	4	20SPR-C	115	Red
COTX05211-7R	1	2020	Springlake	18	20SPR-R	52	Red
COTX05211-7R	2	2020	Springlake	18	20SPR-R	52	Red

COTX05211-7R	1	2021	Springlake	4	21SPR-C	119	Red
COTX05211-7R	2	2021	Springlake	4	21SPR-C	126	Red
COTX05211-7R	1	2021	Springlake	18	21SPR-R	49	Red
COTX05211-7R	2	2021	Springlake	18	21SPR-R	49	Red
COTX05249-3W/Y	1	2019	Dalhart	18	19DAL-R	63	Yellow
COTX05249-3W/Y	2	2019	Dalhart	18	19DAL-R	63	Yellow
COTX05249-3W/Y	1	2020	Dalhart	4	20DAL-C	133	Yellow
COTX05249-3W/Y	2	2020	Dalhart	4	20DAL-C	133	Yellow
COTX05249-3W/Y	1	2020	Dalhart	18	20DAL-R	74	Yellow
COTX05249-3W/Y	2	2020	Dalhart	18	20DAL-R	74	Yellow
COTX05249-3W/Y	1	2020	Springlake	4	20SPR-C	115	Yellow
COTX05249-3W/Y	2	2020	Springlake	4	20SPR-C	94	Yellow
COTX05249-3W/Y	1	2020	Springlake	18	20SPR-R	59	Yellow
COTX05249-3W/Y	2	2020	Springlake	18	20SPR-R	45	Yellow
COTX05249-3W/Y	1	2021	Springlake	4	21SPR-C	119	Yellow
COTX05249-3W/Y	2	2021	Springlake	4	21SPR-C		Yellow
COTX05249-3W/Y	1	2021	Springlake	18	21SPR-R	70	Yellow
COTX05249-3W/Y	2	2021	Springlake	18	21SPR-R		Yellow
COTX05261-1R/Y	1	2019	Dalhart	18	19DAL-R	56	Red
COTX05261-1R/Y	2	2019	Dalhart	18	19DAL-R	63	Red
COTX05261-1R/Y	1	2020	Dalhart	4	20DAL-C	158	Red
COTX05261-1R/Y	2	2020	Dalhart	4	20DAL-C	158	Red
COTX05261-1R/Y	1	2020	Dalhart	18	20DAL-R	67	Red
COTX05261-1R/Y	2	2020	Dalhart	18	20DAL-R	81	Red
COTX05261-1R/Y	1	2020	Springlake	4	20SPR-C	136	Red
COTX05261-1R/Y	2	2020	Springlake	4	20SPR-C	108	Red
COTX05261-1R/Y	1	2020	Springlake	18	20SPR-R	59	Red
COTX05261-1R/Y	2	2020	Springlake	18	20SPR-R	59	Red
COTX05261-1R/Y	1	2021	Springlake	4	21SPR-C	126	Red
COTX05261-1R/Y	2	2021	Springlake	4	21SPR-C	126	Red
COTX05261-1R/Y	1	2021	Springlake	18	21SPR-R	70	Red
COTX05261-1R/Y	2	2021	Springlake	18	21SPR-R	63	Red
COTX08039-1P/P*	1	2019	Dalhart	18	19DAL-R	63	Purple
COTX08039-1P/P*	2	2019	Dalhart	18	19DAL-R	70	Purple
COTX08039-1P/P*	1	2020	Dalhart	4	20DAL-C		Purple
COTX08039-1P/P*	2	2020	Dalhart	4	20DAL-C	133	Purple
COTX08039-1P/P*	1	2020	Dalhart	18	20DAL-R	67	Purple
COTX08039-1P/P*	2	2020	Dalhart	18	20DAL-R	60	Purple
COTX08039-1P/P*	1	2020	Springlake	4	20SPR-C	115	Purple
COTX08039-1P/P*	2	2020	Springlake	4	20SPR-C	136	Purple
COTX08039-1P/P*	1	2020	Springlake	18	20SPR-R	66	Purple
COTX08039-1P/P*	2	2020	Springlake	18	20SPR-R	66	Purple
COTX08039-1P/P*	1	2021	Springlake	4	21SPR-C	126	Purple
COTX08039-1P/P*	2	2021	Springlake	4	21SPR-C	140	Purple
COTX08039-1P/P*	1	2021	Springlake	18	21SPR-R	63	Purple
COTX08039-1P/P*	2	2021	Springlake	18	21SPR-R	70	Purple
COTX08063-2Ru	1	2019	Dalhart	18	19DAL-R	77	Russet

COTX08063-2Ru	2	2019	Dalhart	18	19DAL-R	70	Russet
COTX08063-2Ru	1	2020	Dalhart	4	20DAL-C	133	Russet
COTX08063-2Ru	2	2020	Dalhart	4	20DAL-C	140	Russet
COTX08063-2Ru	1	2020	Dalhart	18	20DAL-R	74	Russet
COTX08063-2Ru	2	2020	Dalhart	18	20DAL-R	95	Russet
COTX08063-2Ru	1	2020	Springlake	4	20SPR-C	109	Russet
COTX08063-2Ru	2	2020	Springlake	4	20SPR-C	116	Russet
COTX08063-2Ru	1	2020	Springlake	18	20SPR-R	60	Russet
COTX08063-2Ru	2	2020	Springlake	18	20SPR-R	60	Russet
COTX08063-2Ru	1	2021	Springlake	4	21SPR-C	114	Russet
COTX08063-2Ru	2	2021	Springlake	4	21SPR-C	107	Russet
COTX08063-2Ru	1	2021	Springlake	18	21SPR-R	91	Russet
COTX08063-2Ru	2	2021	Springlake	18	21SPR-R	91	Russet
COTX08121-1Ru	1	2019	Dalhart	18	19DAL-R	91	Russet
COTX08121-1Ru	2	2019	Dalhart	18	19DAL-R	105	Russet
COTX08121-1Ru	1	2020	Dalhart	4	20DAL-C		Russet
COTX08121-1Ru	2	2020	Dalhart	4	20DAL-C	133	Russet
COTX08121-1Ru	1	2020	Dalhart	18	20DAL-R	109	Russet
COTX08121-1Ru	2	2020	Dalhart	18	20DAL-R	109	Russet
COTX08121-1Ru	1	2020	Springlake	4	20SPR-C	95	Russet
COTX08121-1Ru	2	2020	Springlake	4	20SPR-C	109	Russet
COTX08121-1Ru	1	2020	Springlake	18	20SPR-R	74	Russet
COTX08121-1Ru	2	2020	Springlake	18	20SPR-R	74	Russet
COTX08121-1Ru	1	2021	Springlake	4	21SPR-C	128	Russet
COTX08121-1Ru	2	2021	Springlake	4	21SPR-C	114	Russet
COTX08121-1Ru	1	2021	Springlake	18	21SPR-R	91	Russet
COTX08121-1Ru	2	2021	Springlake	18	21SPR-R	77	Russet
COTX08121-4Ru	1	2019	Dalhart	18	19DAL-R	91	Russet
COTX08121-4Ru	2	2019	Dalhart	18	19DAL-R	91	Russet
COTX08121-4Ru	1	2020	Dalhart	4	20DAL-C	133	Russet
COTX08121-4Ru	2	2020	Dalhart	4	20DAL-C	133	Russet
COTX08121-4Ru	1	2020	Dalhart	18	20DAL-R	87	Russet
COTX08121-4Ru	2	2020	Dalhart	18	20DAL-R	81	Russet
COTX08121-4Ru	1	2020	Springlake	4	20SPR-C	102	Russet
COTX08121-4Ru	2	2020	Springlake	4	20SPR-C	116	Russet
COTX08121-4Ru	1	2020	Springlake	18	20SPR-R	60	Russet
COTX08121-4Ru	2	2020	Springlake	18	20SPR-R	81	Russet
COTX08121-4Ru	1	2021	Springlake	4	21SPR-C	156	Russet
COTX08121-4Ru	2	2021	Springlake	4	21SPR-C	162	Russet
COTX08121-4Ru	1	2021	Springlake	18	21SPR-R	77	Russet
COTX08121-4Ru	2	2021	Springlake	18	21SPR-R	105	Russet
COTX08258-6Ru	1	2019	Dalhart	18	19DAL-R	77	Russet
COTX08258-6Ru	2	2019	Dalhart	18	19DAL-R	63	Russet
COTX08258-6Ru	1	2020	Dalhart	4	20DAL-C		Russet
COTX08258-6Ru	2	2020	Dalhart	4	20DAL-C	133	Russet
COTX08258-6Ru	1	2020	Dalhart	18	20DAL-R	74	Russet
COTX08258-6Ru	2	2020	Dalhart	18	20DAL-R	74	Russet

COTX08258-6Ru	1	2020	Springlake	4	20SPR-C	102	Russet
COTX08258-6Ru	2	2020	Springlake	4	20SPR-C	109	Russet
COTX08258-6Ru	1	2020	Springlake	18	20SPR-R	53	Russet
COTX08258-6Ru	2	2020	Springlake	18	20SPR-R	53	Russet
COTX08258-6Ru	1	2021	Springlake	4	21SPR-C	114	Russet
COTX08258-6Ru	2	2021	Springlake	4	21SPR-C	114	Russet
COTX08258-6Ru	1	2021	Springlake	18	21SPR-R	77	Russet
COTX08258-6Ru	2	2021	Springlake	18	21SPR-R	77	Russet
COTX08322-10Ru	1	2019	Dalhart	18	19DAL-R	91	Russet
COTX08322-10Ru	2	2019	Dalhart	18	19DAL-R	98	Russet
COTX08322-10Ru	1	2020	Dalhart	4	20DAL-C	133	Russet
COTX08322-10Ru	2	2020	Dalhart	4	20DAL-C		Russet
COTX08322-10Ru	1	2020	Dalhart	18	20DAL-R	74	Russet
COTX08322-10Ru	2	2020	Dalhart	18	20DAL-R	81	Russet
COTX08322-10Ru	1	2020	Springlake	4	20SPR-C	88	Russet
COTX08322-10Ru	2	2020	Springlake	4	20SPR-C	95	Russet
COTX08322-10Ru	1	2020	Springlake	18	20SPR-R	74	Russet
COTX08322-10Ru	2	2020	Springlake	18	20SPR-R	81	Russet
COTX08322-10Ru	1	2021	Springlake	4	21SPR-C	107	Russet
COTX08322-10Ru	2	2021	Springlake	4	21SPR-C		Russet
COTX08322-10Ru	1	2021	Springlake	18	21SPR-R	63	Russet
COTX08322-10Ru	2	2021	Springlake	18	21SPR-R	84	Russet
COTX08365F-3P/P	1	2019	Dalhart	18	19DAL-R	126	Purple
COTX08365F-3P/P	2	2019	Dalhart	18	19DAL-R	119	Purple
COTX08365F-3P/P	1	2020	Dalhart	4	20DAL-C	172	Purple
COTX08365F-3P/P	2	2020	Dalhart	4	20DAL-C	179	Purple
COTX08365F-3P/P	1	2020	Dalhart	18	20DAL-R	117	Purple
COTX08365F-3P/P	2	2020	Dalhart	18	20DAL-R	123	Purple
COTX08365F-3P/P	1	2020	Springlake	4	20SPR-C	150	Purple
COTX08365F-3P/P	2	2020	Springlake	4	20SPR-C	150	Purple
COTX08365F-3P/P	1	2020	Springlake	18	20SPR-R	108	Purple
COTX08365F-3P/P	2	2020	Springlake	18	20SPR-R	95	Purple
COTX08365F-3P/P	1	2021	Springlake	4	21SPR-C	140	Purple
COTX08365F-3P/P	2	2021	Springlake	4	21SPR-C	133	Purple
COTX08365F-3P/P	1	2021	Springlake	18	21SPR-R	91	Purple
COTX08365F-3P/P	2	2021	Springlake	18	21SPR-R	91	Purple
COTX09022-3RuRE/Y	1	2019	Dalhart	18	19DAL-R	70	Russet
COTX09022-3RuRE/Y	2	2019	Dalhart	18	19DAL-R	70	Russet
COTX09022-3RuRE/Y	1	2020	Dalhart	4	20DAL-C	133	Russet
COTX09022-3RuRE/Y	2	2020	Dalhart	4	20DAL-C		Russet
COTX09022-3RuRE/Y	1	2020	Dalhart	18	20DAL-R	102	Russet
COTX09022-3RuRE/Y	2	2020	Dalhart	18	20DAL-R	87	Russet
COTX09022-3RuRE/Y	1	2020	Springlake	4	20SPR-C	116	Russet
COTX09022-3RuRE/Y	2	2020	Springlake	4	20SPR-C	95	Russet
COTX09022-3RuRE/Y	1	2020	Springlake	18	20SPR-R	67	Russet
COTX09022-3RuRE/Y	2	2020	Springlake	18	20SPR-R	67	Russet
COTX09022-3RuRE/Y	1	2021	Springlake	4	21SPR-C	94	Russet

COTX09022-3RuRE/Y	2	2021	Springlake	4	21SPR-C	114	Russet
COTX09022-3RuRE/Y	1	2021	Springlake	18	21SPR-R	91	Russet
COTX09022-3RuRE/Y	2	2021	Springlake	18	21SPR-R	77	Russet
COTX09052-1Ru	1	2019	Dalhart	18	19DAL-R	112	Russet
COTX09052-1Ru	2	2019	Dalhart	18	19DAL-R	112	Russet
COTX09052-1Ru	1	2020	Dalhart	4	20DAL-C	179	Russet
COTX09052-1Ru	2	2020	Dalhart	4	20DAL-C	179	Russet
COTX09052-1Ru	1	2020	Dalhart	18	20DAL-R	109	Russet
COTX09052-1Ru	2	2020	Dalhart	18	20DAL-R	117	Russet
COTX09052-1Ru	1	2020	Springlake	4	20SPR-C	109	Russet
COTX09052-1Ru	2	2020	Springlake	4	20SPR-C	137	Russet
COTX09052-1Ru	1	2020	Springlake	18	20SPR-R	89	Russet
COTX09052-1Ru	2	2020	Springlake	18	20SPR-R	74	Russet
COTX09052-1Ru	1	2021	Springlake	4	21SPR-C	128	Russet
COTX09052-1Ru	2	2021	Springlake	4	21SPR-C	149	Russet
COTX09052-1Ru	1	2021	Springlake	18	21SPR-R	119	Russet
COTX09052-1Ru	2	2021	Springlake	18	21SPR-R	119	Russet
COTX09052-2Ru	1	2019	Dalhart	18	19DAL-R	112	Russet
COTX09052-2Ru	2	2019	Dalhart	18	19DAL-R	112	Russet
COTX09052-2Ru	1	2020	Dalhart	4	20DAL-C	151	Russet
COTX09052-2Ru	2	2020	Dalhart	4	20DAL-C	158	Russet
COTX09052-2Ru	1	2020	Dalhart	18	20DAL-R	109	Russet
COTX09052-2Ru	2	2020	Dalhart	18	20DAL-R	109	Russet
COTX09052-2Ru	1	2020	Springlake	4	20SPR-C	130	Russet
COTX09052-2Ru	2	2020	Springlake	4	20SPR-C	130	Russet
COTX09052-2Ru	1	2020	Springlake	18	20SPR-R	95	Russet
COTX09052-2Ru	2	2020	Springlake	18	20SPR-R	89	Russet
COTX09052-2Ru	1	2021	Springlake	4	21SPR-C	156	Russet
COTX09052-2Ru	2	2021	Springlake	4	21SPR-C	135	Russet
COTX09052-2Ru	1	2021	Springlake	18	21SPR-R	98	Russet
COTX09052-2Ru	2	2021	Springlake	18	21SPR-R	105	Russet
COTX09089-1Ru	1	2019	Dalhart	18	19DAL-R	98	Russet
COTX09089-1Ru	2	2019	Dalhart	18	19DAL-R	91	Russet
COTX09089-1Ru	1	2020	Dalhart	4	20DAL-C	172	Russet
COTX09089-1Ru	2	2020	Dalhart	4	20DAL-C	172	Russet
COTX09089-1Ru	1	2020	Dalhart	18	20DAL-R	95	Russet
COTX09089-1Ru	2	2020	Dalhart	18	20DAL-R	102	Russet
COTX09089-1Ru	1	2020	Springlake	4	20SPR-C	144	Russet
COTX09089-1Ru	2	2020	Springlake	4	20SPR-C	137	Russet
COTX09089-1Ru	1	2020	Springlake	18	20SPR-R	74	Russet
COTX09089-1Ru	2	2020	Springlake	18	20SPR-R	81	Russet
COTX09089-1Ru	1	2021	Springlake	4	21SPR-C	149	Russet
COTX09089-1Ru	2	2021	Springlake	4	21SPR-C	128	Russet
COTX09089-1Ru	1	2021	Springlake	18	21SPR-R	84	Russet
COTX09089-1Ru	2	2021	Springlake	18	21SPR-R	84	Russet
COTX10012-1Wrdspl/Y	1	2019	Dalhart	18	19DAL-R	91	Yellow
COTX10012-1Wrdspl/Y	2	2019	Dalhart	18	19DAL-R	70	Yellow

COTX10012-1Wrdspl/Y	1	2020	Dalhart	4	20DAL-C	165	Yellow
COTX10012-1Wrdspl/Y	2	2020	Dalhart	4	20DAL-C	165	Yellow
COTX10012-1Wrdspl/Y	1	2020	Dalhart	18	20DAL-R	81	Yellow
COTX10012-1Wrdspl/Y	2	2020	Dalhart	18	20DAL-R	95	Yellow
COTX10012-1Wrdspl/Y	1	2020	Springlake	4	20SPR-C	136	Yellow
COTX10012-1Wrdspl/Y	2	2020	Springlake	4	20SPR-C	101	Yellow
COTX10012-1Wrdspl/Y	1	2020	Springlake	18	20SPR-R	73	Yellow
COTX10012-1Wrdspl/Y	2	2020	Springlake	18	20SPR-R	66	Yellow
COTX10012-1Wrdspl/Y	1	2021	Springlake	4	21SPR-C	106	Yellow
COTX10012-1Wrdspl/Y	2	2021	Springlake	4	21SPR-C	133	Yellow
COTX10012-1Wrdspl/Y	1	2021	Springlake	18	21SPR-R	70	Yellow
COTX10012-1Wrdspl/Y	2	2021	Springlake	18	21SPR-R	77	Yellow
COTX10073s-1W	1	2019	Dalhart	18	19DAL-R	112	Yellow
COTX10073s-1W	2	2019	Dalhart	18	19DAL-R	105	Yellow
COTX10073s-1W	1	2020	Dalhart	4	20DAL-C	165	Yellow
COTX10073s-1W	2	2020	Dalhart	4	20DAL-C	151	Yellow
COTX10073s-1W	1	2020	Dalhart	18	20DAL-R	102	Yellow
COTX10073s-1W	2	2020	Dalhart	18	20DAL-R	95	Yellow
COTX10073s-1W	1	2020	Springlake	4	20SPR-C	136	Yellow
COTX10073s-1W	2	2020	Springlake	4	20SPR-C	94	Yellow
COTX10073s-1W	1	2020	Springlake	18	20SPR-R	95	Yellow
COTX10073s-1W	2	2020	Springlake	18	20SPR-R	80	Yellow
COTX10073s-1W	1	2021	Springlake	4	21SPR-C	119	Yellow
COTX10073s-1W	2	2021	Springlake	4	21SPR-C	161	Yellow
COTX10073s-1W	1	2021	Springlake	18	21SPR-R	112	Yellow
COTX10073s-1W	2	2021	Springlake	18	21SPR-R	119	Yellow
COTX10080-2Ru	1	2019	Dalhart	18	19DAL-R	91	Russet
COTX10080-2Ru	2	2019	Dalhart	18	19DAL-R	91	Russet
COTX10080-2Ru	1	2020	Dalhart	4	20DAL-C	133	Russet
COTX10080-2Ru	2	2020	Dalhart	4	20DAL-C	133	Russet
COTX10080-2Ru	1	2020	Dalhart	18	20DAL-R	95	Russet
COTX10080-2Ru	2	2020	Dalhart	18	20DAL-R	102	Russet
COTX10080-2Ru	1	2022	Dalhart	4	22DAL-C	122	Russet
COTX10080-2Ru	2	2022	Dalhart	4	22DAL-C	73	Russet
COTX10080-2Ru	1	2022	Dalhart	18	22DAL-R	91	Russet
COTX10080-2Ru	2	2022	Dalhart	18	22DAL-R	91	Russet
COTX10080-2Ru	1	2020	Springlake	4	20SPR-C	88	Russet
COTX10080-2Ru	2	2020	Springlake	4	20SPR-C	102	Russet
COTX10080-2Ru	1	2020	Springlake	18	20SPR-R	53	Russet
COTX10080-2Ru	2	2020	Springlake	18	20SPR-R	60	Russet
COTX10080-2Ru	1	2021	Springlake	4	21SPR-C	107	Russet
COTX10080-2Ru	2	2021	Springlake	4	21SPR-C	100	Russet
COTX10080-2Ru	1	2021	Springlake	18	21SPR-R	63	Russet
COTX10080-2Ru	2	2021	Springlake	18	21SPR-R	84	Russet
COTX10080-2Ru	1	2022	Springlake	4	22SPR-C	64	Russet
COTX10080-2Ru	2	2022	Springlake	4	22SPR-C	93	Russet
COTX10080-2Ru	1	2022	Springlake	18	22SPR-R	34	Russet

COTX10080-2Ru	2	2022	Springlake	18	22SPR-R	34	Russet
COTX10118-1Wre/Y	1	2019	Dalhart	18	19DAL-R	70	Yellow
COTX10118-1Wre/Y	2	2019	Dalhart	18	19DAL-R	56	Yellow
COTX10118-1Wre/Y	1	2020	Dalhart	4	20DAL-C	88	Yellow
COTX10118-1Wre/Y	2	2020	Dalhart	4	20DAL-C	116	Yellow
COTX10118-1Wre/Y	1	2020	Dalhart	18	20DAL-R	74	Yellow
COTX10118-1Wre/Y	2	2020	Dalhart	18	20DAL-R	53	Yellow
COTX10118-1Wre/Y	1	2020	Springlake	4	20SPR-C	101	Yellow
COTX10118-1Wre/Y	2	2020	Springlake	4	20SPR-C	136	Yellow
COTX10118-1Wre/Y	1	2020	Springlake	18	20SPR-R	52	Yellow
COTX10118-1Wre/Y	2	2020	Springlake	18	20SPR-R	66	Yellow
COTX10118-1Wre/Y	1	2021	Springlake	4	21SPR-C	119	Yellow
COTX10118-1Wre/Y	2	2021	Springlake	4	21SPR-C	126	Yellow
COTX10118-1Wre/Y	1	2021	Springlake	18	21SPR-R	63	Yellow
COTX10118-1Wre/Y	2	2021	Springlake	18	21SPR-R	84	Yellow
COTX10118-4Wpe/Y	1	2019	Dalhart	18	19DAL-R	56	Yellow
COTX10118-4Wpe/Y	2	2019	Dalhart	18	19DAL-R	56	Yellow
COTX10118-4Wpe/Y	1	2020	Dalhart	4	20DAL-C		Yellow
COTX10118-4Wpe/Y	2	2020	Dalhart	4	20DAL-C	151	Yellow
COTX10118-4Wpe/Y	1	2020	Dalhart	18	20DAL-R	60	Yellow
COTX10118-4Wpe/Y	2	2020	Dalhart	18	20DAL-R	67	Yellow
COTX10118-4Wpe/Y	1	2020	Springlake	4	20SPR-C	122	Yellow
COTX10118-4Wpe/Y	2	2020	Springlake	4	20SPR-C	101	Yellow
COTX10118-4Wpe/Y	1	2020	Springlake	18	20SPR-R	59	Yellow
COTX10118-4Wpe/Y	2	2020	Springlake	18	20SPR-R	52	Yellow
COTX10118-4Wpe/Y	1	2021	Springlake	4	21SPR-C	126	Yellow
COTX10118-4Wpe/Y	2	2021	Springlake	4	21SPR-C	119	Yellow
COTX10118-4Wpe/Y	1	2021	Springlake	18	21SPR-R	63	Yellow
COTX10118-4Wpe/Y	2	2021	Springlake	18	21SPR-R	70	Yellow
COTX10138-15Wpe/Y	1	2019	Dalhart	18	19DAL-R	56	Yellow
COTX10138-15Wpe/Y	2	2019	Dalhart	18	19DAL-R	56	Yellow
COTX10138-15Wpe/Y	1	2020	Dalhart	4	20DAL-C		Yellow
COTX10138-15Wpe/Y	2	2020	Dalhart	4	20DAL-C		Yellow
COTX10138-15Wpe/Y	1	2020	Dalhart	18	20DAL-R	48	Yellow
COTX10138-15Wpe/Y	2	2020	Dalhart	18	20DAL-R	48	Yellow
COTX10138-15Wpe/Y	1	2020	Springlake	4	20SPR-C	101	Yellow
COTX10138-15Wpe/Y	2	2020	Springlake	4	20SPR-C	122	Yellow
COTX10138-15Wpe/Y	1	2020	Springlake	18	20SPR-R	38	Yellow
COTX10138-15Wpe/Y	2	2020	Springlake	18	20SPR-R	38	Yellow
COTX10138-15Wpe/Y	1	2021	Springlake	4	21SPR-C	126	Yellow
COTX10138-15Wpe/Y	2	2021	Springlake	4	21SPR-C	119	Yellow
COTX10138-15Wpe/Y	1	2021	Springlake	18	21SPR-R	42	Yellow
COTX10138-15Wpe/Y	2	2021	Springlake	18	21SPR-R	49	Yellow
COTX10138-19P/Y	1	2019	Dalhart	18	19DAL-R	91	Purple
COTX10138-19P/Y	2	2019	Dalhart	18	19DAL-R		Purple
COTX10138-19P/Y	1	2020	Dalhart	4	20DAL-C	144	Purple
COTX10138-19P/Y	2	2020	Dalhart	4	20DAL-C	123	Purple

COTX10138-19P/Y	1	2020	Dalhart	18	20DAL-R	74	Purple
COTX10138-19P/Y	2	2020	Dalhart	18	20DAL-R	74	Purple
COTX10138-19P/Y	1	2020	Springlake	4	20SPR-C	150	Purple
COTX10138-19P/Y	2	2020	Springlake	4	20SPR-C	150	Purple
COTX10138-19P/Y	1	2020	Springlake	18	20SPR-R	80	Purple
COTX10138-19P/Y	2	2020	Springlake	18	20SPR-R	80	Purple
COTX10138-19P/Y	1	2021	Springlake	4	21SPR-C	174	Purple
COTX10138-19P/Y	2	2021	Springlake	4	21SPR-C	174	Purple
COTX10138-19P/Y	1	2021	Springlake	18	21SPR-R	105	Purple
COTX10138-19P/Y	2	2021	Springlake	18	21SPR-R	112	Purple
COTX10138s-7Wpe/Y	1	2019	Dalhart	18	19DAL-R	56	Yellow
COTX10138s-7Wpe/Y	2	2019	Dalhart	18	19DAL-R	70	Yellow
COTX10138s-7Wpe/Y	1	2020	Dalhart	4	20DAL-C		Yellow
COTX10138s-7Wpe/Y	2	2020	Dalhart	4	20DAL-C	116	Yellow
COTX10138s-7Wpe/Y	1	2020	Dalhart	18	20DAL-R	60	Yellow
COTX10138s-7Wpe/Y	2	2020	Dalhart	18	20DAL-R	60	Yellow
COTX10138s-7Wpe/Y	1	2020	Springlake	4	20SPR-C	122	Yellow
COTX10138s-7Wpe/Y	2	2020	Springlake	4	20SPR-C	101	Yellow
COTX10138s-7Wpe/Y	1	2020	Springlake	18	20SPR-R	45	Yellow
COTX10138s-7Wpe/Y	2	2020	Springlake	18	20SPR-R	38	Yellow
COTX10138s-7Wpe/Y	1	2021	Springlake	4	21SPR-C	119	Yellow
COTX10138s-7Wpe/Y	2	2021	Springlake	4	21SPR-C	112	Yellow
COTX10138s-7Wpe/Y	1	2021	Springlake	18	21SPR-R	63	Yellow
COTX10138s-7Wpe/Y	2	2021	Springlake	18	21SPR-R	70	Yellow
COTX10226-1Wpe/Y	1	2019	Dalhart	18	19DAL-R	70	Yellow
COTX10226-1Wpe/Y	2	2019	Dalhart	18	19DAL-R	70	Yellow
COTX10226-1Wpe/Y	1	2020	Dalhart	4	20DAL-C		Yellow
COTX10226-1Wpe/Y	2	2020	Dalhart	4	20DAL-C	214	Yellow
COTX10226-1Wpe/Y	1	2020	Dalhart	18	20DAL-R	60	Yellow
COTX10226-1Wpe/Y	2	2020	Dalhart	18	20DAL-R	60	Yellow
COTX10226-1Wpe/Y	1	2020	Springlake	4	20SPR-C	136	Yellow
COTX10226-1Wpe/Y	2	2020	Springlake	4	20SPR-C	122	Yellow
COTX10226-1Wpe/Y	1	2020	Springlake	18	20SPR-R	73	Yellow
COTX10226-1Wpe/Y	2	2020	Springlake	18	20SPR-R	73	Yellow
COTX10226-1Wpe/Y	1	2021	Springlake	4	21SPR-C		Yellow
COTX10226-1Wpe/Y	2	2021	Springlake	4	21SPR-C	119	Yellow
COTX10226-1Wpe/Y	1	2021	Springlake	18	21SPR-R	84	Yellow
COTX10226-1Wpe/Y	2	2021	Springlake	18	21SPR-R	84	Yellow
COTX12235-2W	1	2022	Dalhart	4	22DAL-C	137	Chipper
COTX12235-2W	2	2022	Dalhart	4	22DAL-C	116	Chipper
COTX12235-2W	1	2022	Dalhart	18	22DAL-R	78	Chipper
COTX12235-2W	2	2022	Dalhart	18	22DAL-R	78	Chipper
COTX12235-2W	1	2022	Springlake	4	22SPR-C	102	Chipper
COTX12235-2W	2	2022	Springlake	4	22SPR-C	65	Chipper
COTX12235-2W	1	2022	Springlake	18	22SPR-R	57	Chipper
COTX12235-2W	2	2022	Springlake	18	22SPR-R	62	Chipper
COTX13029-2Ru/Y	1	2022	Dalhart	4	22DAL-C	101	Russet

COTX13029-2Ru/Y	2	2022	Dalhart	4	22DAL-C	87	Russet
COTX13029-2Ru/Y	1	2022	Dalhart	18	22DAL-R	63	Russet
COTX13029-2Ru/Y	2	2022	Dalhart	18	22DAL-R	56	Russet
COTX13029-2Ru/Y	1	2022	Springlake	4	22SPR-C	99	Russet
COTX13029-2Ru/Y	2	2022	Springlake	4	22SPR-C	78	Russet
COTX13029-2Ru/Y	1	2022	Springlake	18	22SPR-R	40	Russet
COTX13029-2Ru/Y	2	2022	Springlake	18	22SPR-R	40	Russet
COTX13215-2Ru	1	2019	Dalhart	18	19DAL-R	112	Russet
COTX13215-2Ru	2	2019	Dalhart	18	19DAL-R	112	Russet
COTX13215-2Ru	1	2020	Dalhart	4	20DAL-C	193	Russet
COTX13215-2Ru	2	2020	Dalhart	4	20DAL-C	200	Russet
COTX13215-2Ru	1	2020	Dalhart	18	20DAL-R	117	Russet
COTX13215-2Ru	2	2020	Dalhart	18	20DAL-R	129	Russet
COTX13215-2Ru	1	2020	Springlake	4	20SPR-C	144	Russet
COTX13215-2Ru	2	2020	Springlake	4	20SPR-C	130	Russet
COTX13215-2Ru	1	2020	Springlake	18	20SPR-R	95	Russet
COTX13215-2Ru	2	2020	Springlake	18	20SPR-R	109	Russet
COTX13215-2Ru	1	2021	Springlake	4	21SPR-C	114	Russet
COTX13215-2Ru	2	2021	Springlake	4	21SPR-C	128	Russet
COTX13215-2Ru	1	2021	Springlake	18	21SPR-R	119	Russet
COTX13215-2Ru	2	2021	Springlake	18	21SPR-R	112	Russet
COTX15083-1R	1	2022	Dalhart	4	22DAL-C	137	Red
COTX15083-1R	2	2022	Dalhart	4	22DAL-C	130	Red
COTX15083-1R	1	2022	Dalhart	18	22DAL-R	92	Red
COTX15083-1R	2	2022	Dalhart	18	22DAL-R	85	Red
COTX15083-1R	1	2022	Springlake	4	22SPR-C	111	Red
COTX15083-1R	2	2022	Springlake	4	22SPR-C	111	Red
COTX15083-1R	1	2022	Springlake	18	22SPR-R	66	Red
COTX15083-1R	2	2022	Springlake	18	22SPR-R	66	Red
COTX15111-1R	1	2022	Dalhart	4	22DAL-C	116	Red
COTX15111-1R	2	2022	Dalhart	4	22DAL-C	137	Red
COTX15111-1R	1	2022	Dalhart	18	22DAL-R	85	Red
COTX15111-1R	2	2022	Dalhart	18	22DAL-R	85	Red
COTX15111-1R	1	2022	Springlake	4	22SPR-C	104	Red
COTX15111-1R	2	2022	Springlake	4	22SPR-C	83	Red
COTX15111-1R	1	2022	Springlake	18	22SPR-R	52	Red
COTX15111-1R	2	2022	Springlake	18	22SPR-R	52	Red
COTX15271-1Ru	1	2022	Dalhart	4	22DAL-C	122	Russet
COTX15271-1Ru	2	2022	Dalhart	4	22DAL-C	143	Russet
COTX15271-1Ru	1	2022	Dalhart	18	22DAL-R	91	Russet
COTX15271-1Ru	2	2022	Dalhart	18	22DAL-R	91	Russet
COTX15271-1Ru	1	2022	Springlake	4	22SPR-C	99	Russet
COTX15271-1Ru	2	2022	Springlake	4	22SPR-C	99	Russet
COTX15271-1Ru	1	2022	Springlake	18	22SPR-R	61	Russet
COTX15271-1Ru	2	2022	Springlake	18	22SPR-R	61	Russet
COTX16005-1Ru	1	2022	Dalhart	4	22DAL-C	178	Russet
COTX16005-1Ru	2	2022	Dalhart	4	22DAL-C	150	Russet

COTX16005-1Ru	1	2022	Dalhart	18	22DAL-R	70	Russet
COTX16005-1Ru	2	2022	Dalhart	18	22DAL-R	70	Russet
COTX16005-1Ru	1	2022	Springlake	4	22SPR-C	106	Russet
COTX16005-1Ru	2	2022	Springlake	4	22SPR-C	99	Russet
COTX16005-1Ru	1	2022	Springlake	18	22SPR-R	40	Russet
COTX16005-1Ru	2	2022	Springlake	18	22SPR-R	47	Russet
COTX16054-1W	1	2022	Dalhart	4	22DAL-C	116	Chipper
COTX16054-1W	2	2022	Dalhart	4	22DAL-C	116	Chipper
COTX16054-1W	1	2022	Dalhart	18	22DAL-R	85	Chipper
COTX16054-1W	2	2022	Dalhart	18	22DAL-R	78	Chipper
COTX16054-1W	1	2022	Springlake	4	22SPR-C	81	Chipper
COTX16054-1W	2	2022	Springlake	4	22SPR-C	93	Chipper
COTX16054-1W	1	2022	Springlake	18	22SPR-R	71	Chipper
COTX16054-1W	2	2022	Springlake	18	22SPR-R	48	Chipper
COTX17016f-2P/P	1	2022	Dalhart	4	22DAL-C	193	Purple
COTX17016f-2P/P	2	2022	Dalhart	4	22DAL-C	179	Purple
COTX17016f-2P/P	1	2022	Dalhart	18	22DAL-R	151	Purple
COTX17016f-2P/P	2	2022	Dalhart	18	22DAL-R	169	Purple
COTX17016f-2P/P	1	2022	Springlake	4	22SPR-C	111	Purple
COTX17016f-2P/P	2	2022	Springlake	4	22SPR-C	114	Purple
COTX17016f-2P/P	1	2022	Springlake	18	22SPR-R	101	Purple
COTX17016f-2P/P	2	2022	Springlake	18	22SPR-R	76	Purple
COTX17115f-3R/R	1	2022	Dalhart	4	22DAL-C	179	Red
COTX17115f-3R/R	2	2022	Dalhart	4	22DAL-C	179	Red
COTX17115f-3R/R	1	2022	Dalhart	18	22DAL-R	113	Red
COTX17115f-3R/R	2	2022	Dalhart	18	22DAL-R	120	Red
COTX17115f-3R/R	1	2022	Springlake	4	22SPR-C	139	Red
COTX17115f-3R/R	2	2022	Springlake	4	22SPR-C	111	Red
COTX17115f-3R/R	1	2022	Springlake	18	22SPR-R	94	Red
COTX17115f-3R/R	2	2022	Springlake	18	22SPR-R	94	Red
COTX17123-2Ru	1	2022	Dalhart	4	22DAL-C	143	Russet
COTX17123-2Ru	2	2022	Dalhart	4	22DAL-C	108	Russet
COTX17123-2Ru	1	2022	Dalhart	18	22DAL-R	119	Russet
COTX17123-2Ru	2	2022	Dalhart	18	22DAL-R	119	Russet
COTX17123-2Ru	1	2022	Springlake	4	22SPR-C	99	Russet
COTX17123-2Ru	2	2022	Springlake	4	22SPR-C	99	Russet
COTX17123-2Ru	1	2022	Springlake	18	22SPR-R	68	Russet
COTX17123-2Ru	2	2022	Springlake	18	22SPR-R	82	Russet
COTX17286-6W	1	2022	Dalhart	4	22DAL-C	116	Chipper
COTX17286-6W	2	2022	Dalhart	4	22DAL-C	116	Chipper
COTX17286-6W	1	2022	Dalhart	18	22DAL-R	78	Chipper
COTX17286-6W	2	2022	Dalhart	18	22DAL-R	85	Chipper
COTX17286-6W	1	2022	Springlake	4	22SPR-C	102	Chipper
COTX17286-6W	2	2022	Springlake	4	22SPR-C	93	Chipper
COTX17286-6W	1	2022	Springlake	18	22SPR-R	78	Chipper
COTX17286-6W	2	2022	Springlake	18	22SPR-R	69	Chipper
COTX17288-1W	1	2022	Dalhart	4	22DAL-C	95	Chipper

COTX17288-1W	2	2022	Dalhart	4	22DAL-C	88	Chipper
COTX17288-1W	1	2022	Dalhart	18	22DAL-R	64	Chipper
COTX17288-1W	2	2022	Dalhart	18	22DAL-R	71	Chipper
COTX17288-1W	1	2022	Springlake	4	22SPR-C	102	Chipper
COTX17288-1W	2	2022	Springlake	4	22SPR-C	93	Chipper
COTX17288-1W	1	2022	Springlake	18	22SPR-R	43	Chipper
COTX17288-1W	2	2022	Springlake	18	22SPR-R	48	Chipper
COTX17288-3W	1	2022	Dalhart	4	22DAL-C	116	Chipper
COTX17288-3W	2	2022	Dalhart	4	22DAL-C	158	Chipper
COTX17288-3W	1	2022	Dalhart	18	22DAL-R	78	Chipper
COTX17288-3W	2	2022	Dalhart	18	22DAL-R	92	Chipper
COTX17288-3W	1	2022	Springlake	4	22SPR-C	102	Chipper
COTX17288-3W	2	2022	Springlake	4	22SPR-C	58	Chipper
COTX17288-3W	1	2022	Springlake	18	22SPR-R	50	Chipper
COTX17288-3W	2	2022	Springlake	18	22SPR-R	55	Chipper
COTX17288-4W	1	2022	Dalhart	4	22DAL-C	116	Chipper
COTX17288-4W	2	2022	Dalhart	4	22DAL-C	95	Chipper
COTX17288-4W	1	2022	Dalhart	18	22DAL-R	92	Chipper
COTX17288-4W	2	2022	Dalhart	18	22DAL-R	85	Chipper
COTX17288-4W	1	2022	Springlake	4	22SPR-C	81	Chipper
COTX17288-4W	2	2022	Springlake	4	22SPR-C	72	Chipper
COTX17288-4W	1	2022	Springlake	18	22SPR-R	57	Chipper
COTX17288-4W	2	2022	Springlake	18	22SPR-R	48	Chipper
COTX17304-1Ru	1	2022	Dalhart	4	22DAL-C	150	Russet
COTX17304-1Ru	2	2022	Dalhart	4	22DAL-C	143	Russet
COTX17304-1Ru	1	2022	Dalhart	18	22DAL-R	91	Russet
COTX17304-1Ru	2	2022	Dalhart	18	22DAL-R	91	Russet
COTX17304-1Ru	1	2022	Springlake	4	22SPR-C	99	Russet
COTX17304-1Ru	2	2022	Springlake	4	22SPR-C	99	Russet
COTX17304-1Ru	1	2022	Springlake	18	22SPR-R	61	Russet
COTX17304-1Ru	2	2022	Springlake	18	22SPR-R	61	Russet
COTX18206-1Ru	1	2022	Dalhart	4	22DAL-C	122	Russet
COTX18206-1Ru	2	2022	Dalhart	4	22DAL-C	122	Russet
COTX18206-1Ru	1	2022	Dalhart	18	22DAL-R	119	Russet
COTX18206-1Ru	2	2022	Dalhart	18	22DAL-R	98	Russet
COTX18206-1Ru	1	2022	Springlake	4	22SPR-C	99	Russet
COTX18206-1Ru	2	2022	Springlake	4	22SPR-C	93	Russet
COTX18206-1Ru	1	2022	Springlake	18	22SPR-R	40	Russet
COTX18206-1Ru	2	2022	Springlake	18	22SPR-R	41	Russet
COTX87601-2Ru	1	2019	Dalhart	18	19DAL-R	91	Russet
COTX87601-2Ru	2	2019	Dalhart	18	19DAL-R	105	Russet
COTX87601-2Ru	1	2020	Dalhart	4	20DAL-C	144	Russet
COTX87601-2Ru	2	2020	Dalhart	4	20DAL-C	140	Russet
COTX87601-2Ru	1	2020	Dalhart	18	20DAL-R	74	Russet
COTX87601-2Ru	2	2020	Dalhart	18	20DAL-R	95	Russet
COTX87601-2Ru	1	2020	Springlake	4	20SPR-C	109	Russet
COTX87601-2Ru	2	2020	Springlake	4	20SPR-C	123	Russet

COTX87601-2Ru	1	2020	Springlake	18	20SPR-R	60	Russet
COTX87601-2Ru	2	2020	Springlake	18	20SPR-R	60	Russet
COTX87601-2Ru	1	2021	Springlake	4	21SPR-C	114	Russet
COTX87601-2Ru	2	2021	Springlake	4	21SPR-C	100	Russet
COTX87601-2Ru	1	2021	Springlake	18	21SPR-R	70	Russet
COTX87601-2Ru	2	2021	Springlake	18	21SPR-R	77	Russet
COTX89044-1Ru	1	2019	Dalhart	18	19DAL-R	112	Russet
COTX89044-1Ru	2	2019	Dalhart	18	19DAL-R	84	Russet
COTX89044-1Ru	1	2020	Dalhart	4	20DAL-C	165	Russet
COTX89044-1Ru	2	2020	Dalhart	4	20DAL-C	151	Russet
COTX89044-1Ru	1	2020	Dalhart	18	20DAL-R	102	Russet
COTX89044-1Ru	2	2020	Dalhart	18	20DAL-R	95	Russet
COTX89044-1Ru	1	2020	Springlake	4	20SPR-C	130	Russet
COTX89044-1Ru	2	2020	Springlake	4	20SPR-C	123	Russet
COTX89044-1Ru	1	2020	Springlake	18	20SPR-R	67	Russet
COTX89044-1Ru	2	2020	Springlake	18	20SPR-R	60	Russet
COTX89044-1Ru	1	2021	Springlake	4	21SPR-C	128	Russet
COTX89044-1Ru	2	2021	Springlake	4	21SPR-C	107	Russet
COTX89044-1Ru	1	2021	Springlake	18	21SPR-R	63	Russet
COTX89044-1Ru	2	2021	Springlake	18	21SPR-R	63	Russet
COTX90046-1W	1	2019	Dalhart	18	19DAL-R	63	Chipper
COTX90046-1W	2	2019	Dalhart	18	19DAL-R	56	Chipper
COTX90046-1W	1	2020	Dalhart	4	20DAL-C		Chipper
COTX90046-1W	2	2020	Dalhart	4	20DAL-C		Chipper
COTX90046-1W	1	2020	Dalhart	18	20DAL-R	67	Chipper
COTX90046-1W	2	2020	Dalhart	18	20DAL-R	74	Chipper
COTX90046-1W	1	2020	Springlake	4	20SPR-C	115	Chipper
COTX90046-1W	2	2020	Springlake	4	20SPR-C	136	Chipper
COTX90046-1W	1	2020	Springlake	18	20SPR-R	87	Chipper
COTX90046-1W	2	2020	Springlake	18	20SPR-R	66	Chipper
COTX90046-1W	1	2021	Springlake	4	21SPR-C	119	Chipper
COTX90046-1W	2	2021	Springlake	4	21SPR-C	126	Chipper
COTX90046-1W	1	2021	Springlake	18	21SPR-R	49	Chipper
COTX90046-1W	2	2021	Springlake	18	21SPR-R	70	Chipper
COTX90046-5W	1	2019	Dalhart	18	19DAL-R	56	Russet
COTX90046-5W	2	2019	Dalhart	18	19DAL-R	63	Russet
COTX90046-5W	1	2020	Dalhart	4	20DAL-C		Russet
COTX90046-5W	2	2020	Dalhart	4	20DAL-C	151	Russet
COTX90046-5W	1	2020	Dalhart	18	20DAL-R	74	Russet
COTX90046-5W	2	2020	Dalhart	18	20DAL-R	87	Russet
COTX90046-5W	1	2020	Springlake	4	20SPR-C	94	Russet
COTX90046-5W	2	2020	Springlake	4	20SPR-C	108	Russet
COTX90046-5W	1	2020	Springlake	18	20SPR-R	66	Russet
COTX90046-5W	2	2020	Springlake	18	20SPR-R	80	Russet
COTX90046-5W	1	2021	Springlake	4	21SPR-C	119	Russet
COTX90046-5W	2	2021	Springlake	4	21SPR-C	133	Russet
COTX90046-5W	1	2021	Springlake	18	21SPR-R	84	Russet

COTX90046-5W	2	2021	Springlake	18	21SPR-R	91	Russet
COTX94216-1R	1	2019	Dalhart	18	19DAL-R	91	Red
COTX94216-1R	2	2019	Dalhart	18	19DAL-R	70	Red
COTX94216-1R	1	2020	Dalhart	4	20DAL-C	165	Red
COTX94216-1R	2	2020	Dalhart	4	20DAL-C	172	Red
COTX94216-1R	1	2020	Dalhart	18	20DAL-R	95	Red
COTX94216-1R	2	2020	Dalhart	18	20DAL-R	95	Red
COTX94216-1R	1	2020	Springlake	4	20SPR-C	115	Red
COTX94216-1R	2	2020	Springlake	4	20SPR-C	143	Red
COTX94216-1R	1	2020	Springlake	18	20SPR-R	73	Red
COTX94216-1R	2	2020	Springlake	18	20SPR-R	73	Red
COTX94216-1R	1	2021	Springlake	4	21SPR-C	174	Red
COTX94216-1R	2	2021	Springlake	4	21SPR-C	195	Red
COTX94216-1R	1	2021	Springlake	18	21SPR-R	112	Red
COTX94216-1R	2	2021	Springlake	18	21SPR-R	91	Red
COTX94218-1R	1	2019	Dalhart	18	19DAL-R	63	Red
COTX94218-1R	2	2019	Dalhart	18	19DAL-R	91	Red
COTX94218-1R	1	2020	Dalhart	4	20DAL-C	172	Red
COTX94218-1R	2	2020	Dalhart	4	20DAL-C	172	Red
COTX94218-1R	1	2020	Dalhart	18	20DAL-R	95	Red
COTX94218-1R	2	2020	Dalhart	18	20DAL-R	87	Red
COTX94218-1R	1	2020	Springlake	4	20SPR-C		Red
COTX94218-1R	2	2020	Springlake	4	20SPR-C	171	Red
COTX94218-1R	1	2020	Springlake	18	20SPR-R	52	Red
COTX94218-1R	2	2020	Springlake	18	20SPR-R	66	Red
COTX94218-1R	1	2021	Springlake	4	21SPR-C	154	Red
COTX94218-1R	2	2021	Springlake	4	21SPR-C	174	Red
COTX94218-1R	1	2021	Springlake	18	21SPR-R	91	Red
COTX94218-1R	2	2021	Springlake	18	21SPR-R	77	Red
JTTX75/2003EH-1Yre/Y	1	2019	Dalhart	18	19DAL-R	77	Yellow
JTTX75/2003EH-1Yre/Y	2	2019	Dalhart	18	19DAL-R	70	Yellow
JTTX75/2003EH-1Yre/Y	1	2020	Dalhart	4	20DAL-C	95	Yellow
JTTX75/2003EH-1Yre/Y	2	2020	Dalhart	4	20DAL-C	88	Yellow
JTTX75/2003EH-1Yre/Y	1	2020	Dalhart	18	20DAL-R	74	Yellow
JTTX75/2003EH-1Yre/Y	2	2020	Dalhart	18	20DAL-R	74	Yellow
JTTX75/2003EH-1Yre/Y	1	2020	Springlake	4	20SPR-C	94	Yellow
JTTX75/2003EH-1Yre/Y	2	2020	Springlake	4	20SPR-C	101	Yellow
JTTX75/2003EH-1Yre/Y	1	2020	Springlake	18	20SPR-R	66	Yellow
JTTX75/2003EH-1Yre/Y	2	2020	Springlake	18	20SPR-R	52	Yellow
JTTX75/2003EH-1Yre/Y	1	2021	Springlake	4	21SPR-C	92	Yellow
JTTX75/2003EH-1Yre/Y	2	2021	Springlake	4	21SPR-C	99	Yellow
JTTX75/2003EH-1Yre/Y	1	2021	Springlake	18	21SPR-R	84	Yellow
JTTX75/2003EH-1Yre/Y	2	2021	Springlake	18	21SPR-R	70	Yellow
Krantz	1	2019	Dalhart	18	19DAL-R	77	Russet
Krantz	2	2019	Dalhart	18	19DAL-R		Russet
Krantz	1	2020	Dalhart	4	20DAL-C	133	Russet
Krantz	2	2020	Dalhart	4	20DAL-C		Russet

Krantz	1	2020	Dalhart	18	20DAL-R	74	Russet
Krantz	2	2020	Dalhart	18	20DAL-R	74	Russet
Krantz	1	2020	Springlake	4	20SPR-C	88	Russet
Krantz	2	2020	Springlake	4	20SPR-C	95	Russet
Krantz	1	2020	Springlake	18	20SPR-R	60	Russet
Krantz	2	2020	Springlake	18	20SPR-R	53	Russet
Krantz	1	2021	Springlake	4	21SPR-C	87	Russet
Krantz	2	2021	Springlake	4	21SPR-C	114	Russet
Krantz	1	2021	Springlake	18	21SPR-R	63	Russet
Krantz	2	2021	Springlake	18	21SPR-R	63	Russet
Modoc	1	2022	Dalhart	4	22DAL-C	179	Red
Modoc	2	2022	Dalhart	4	22DAL-C	158	Red
Modoc	1	2022	Dalhart	18	22DAL-R	113	Red
Modoc	2	2022	Dalhart	18	22DAL-R	106	Red
Modoc	1	2022	Springlake	4	22SPR-C	111	Red
Modoc	2	2022	Springlake	4	22SPR-C	111	Red
Modoc	1	2022	Springlake	18	22SPR-R	73	Red
Modoc	2	2022	Springlake	18	22SPR-R	66	Red
MWTX2609-2Ru	1	2019	Dalhart	18	19DAL-R	84	Russet
MWTX2609-2Ru	2	2019	Dalhart	18	19DAL-R	84	Russet
MWTX2609-2Ru	1	2020	Dalhart	4	20DAL-C	123	Russet
MWTX2609-2Ru	2	2020	Dalhart	4	20DAL-C	140	Russet
MWTX2609-2Ru	1	2020	Dalhart	18	20DAL-R	95	Russet
MWTX2609-2Ru	2	2020	Dalhart	18	20DAL-R	81	Russet
MWTX2609-2Ru	1	2020	Springlake	4	20SPR-C	130	Russet
MWTX2609-2Ru	2	2020	Springlake	4	20SPR-C		Russet
MWTX2609-2Ru	1	2020	Springlake	18	20SPR-R	67	Russet
MWTX2609-2Ru	2	2020	Springlake	18	20SPR-R	53	Russet
MWTX2609-2Ru	1	2021	Springlake	4	21SPR-C	121	Russet
MWTX2609-2Ru	2	2021	Springlake	4	21SPR-C	121	Russet
MWTX2609-2Ru	1	2021	Springlake	18	21SPR-R	112	Russet
MWTX2609-2Ru	2	2021	Springlake	18	21SPR-R	84	Russet
MWTX2609-4Ru	1	2019	Dalhart	18	19DAL-R	91	Russet
MWTX2609-4Ru	2	2019	Dalhart	18	19DAL-R	112	Russet
MWTX2609-4Ru	1	2020	Dalhart	4	20DAL-C	151	Russet
MWTX2609-4Ru	2	2020	Dalhart	4	20DAL-C	158	Russet
MWTX2609-4Ru	1	2020	Dalhart	18	20DAL-R	81	Russet
MWTX2609-4Ru	2	2020	Dalhart	18	20DAL-R	102	Russet
MWTX2609-4Ru	1	2020	Springlake	4	20SPR-C	109	Russet
MWTX2609-4Ru	2	2020	Springlake	4	20SPR-C	130	Russet
MWTX2609-4Ru	1	2020	Springlake	18	20SPR-R	67	Russet
MWTX2609-4Ru	2	2020	Springlake	18	20SPR-R	53	Russet
MWTX2609-4Ru	1	2021	Springlake	4	21SPR-C	114	Russet
MWTX2609-4Ru	2	2021	Springlake	4	21SPR-C	107	Russet
MWTX2609-4Ru	1	2021	Springlake	18	21SPR-R	84	Russet
MWTX2609-4Ru	2	2021	Springlake	18	21SPR-R	70	Russet
MWTX548-2Ru	1	2019	Dalhart	18	19DAL-R	91	Russet

MWTX548-2Ru	2	2019	Dalhart	18	19DAL-R	77	Russet
MWTX548-2Ru	1	2020	Dalhart	4	20DAL-C	133	Russet
MWTX548-2Ru	2	2020	Dalhart	4	20DAL-C	133	Russet
MWTX548-2Ru	1	2020	Dalhart	18	20DAL-R	95	Russet
MWTX548-2Ru	2	2020	Dalhart	18	20DAL-R	95	Russet
MWTX548-2Ru	1	2020	Springlake	4	20SPR-C	116	Russet
MWTX548-2Ru	2	2020	Springlake	4	20SPR-C	109	Russet
MWTX548-2Ru	1	2020	Springlake	18	20SPR-R	53	Russet
MWTX548-2Ru	2	2020	Springlake	18	20SPR-R	46	Russet
MWTX548-2Ru	1	2021	Springlake	4	21SPR-C	114	Russet
MWTX548-2Ru	2	2021	Springlake	4	21SPR-C		Russet
MWTX548-2Ru	1	2021	Springlake	18	21SPR-R	84	Russet
MWTX548-2Ru	2	2021	Springlake	18	21SPR-R	77	Russet
NDTX050169-1R	1	2019	Dalhart	18	19DAL-R	91	Red
NDTX050169-1R	2	2019	Dalhart	18	19DAL-R	98	Red
NDTX050169-1R	1	2020	Dalhart	4	20DAL-C	133	Red
NDTX050169-1R	2	2020	Dalhart	4	20DAL-C	151	Red
NDTX050169-1R	1	2020	Dalhart	18	20DAL-R	81	Red
NDTX050169-1R	2	2020	Dalhart	18	20DAL-R	102	Red
NDTX050169-1R	1	2020	Springlake	4	20SPR-C	108	Red
NDTX050169-1R	2	2020	Springlake	4	20SPR-C	108	Red
NDTX050169-1R	1	2020	Springlake	18	20SPR-R	73	Red
NDTX050169-1R	2	2020	Springlake	18	20SPR-R	66	Red
NDTX050169-1R	1	2021	Springlake	4	21SPR-C	133	Red
NDTX050169-1R	2	2021	Springlake	4	21SPR-C	147	Red
NDTX050169-1R	1	2021	Springlake	18	21SPR-R	84	Red
NDTX050169-1R	2	2021	Springlake	18	21SPR-R	91	Red
NDTX050184s-1R/Y	1	2019	Dalhart	18	19DAL-R	56	Red
NDTX050184s-1R/Y	2	2019	Dalhart	18	19DAL-R	70	Red
NDTX050184s-1R/Y	1	2020	Dalhart	4	20DAL-C		Red
NDTX050184s-1R/Y	2	2020	Dalhart	4	20DAL-C		Red
NDTX050184s-1R/Y	1	2020	Dalhart	18	20DAL-R	81	Red
NDTX050184s-1R/Y	2	2020	Dalhart	18	20DAL-R	74	Red
NDTX050184s-1R/Y	1	2020	Springlake	4	20SPR-C	101	Red
NDTX050184s-1R/Y	2	2020	Springlake	4	20SPR-C	108	Red
NDTX050184s-1R/Y	1	2020	Springlake	18	20SPR-R	87	Red
NDTX050184s-1R/Y	2	2020	Springlake	18	20SPR-R	66	Red
NDTX050184s-1R/Y	1	2021	Springlake	4	21SPR-C	99	Red
NDTX050184s-1R/Y	2	2021	Springlake	4	21SPR-C	112	Red
NDTX050184s-1R/Y	1	2021	Springlake	18	21SPR-R	77	Red
NDTX050184s-1R/Y	2	2021	Springlake	18	21SPR-R	63	Red
NDTX059759-3R/Y Pinto	1	2019	Dalhart	18	19DAL-R	91	Red
NDTX059759-3R/Y Pinto	2	2019	Dalhart	18	19DAL-R	98	Red
NDTX059759-3R/Y Pinto	1	2020	Dalhart	4	20DAL-C	140	Red
NDTX059759-3R/Y Pinto	2	2020	Dalhart	4	20DAL-C	140	Red
NDTX059759-3R/Y Pinto	1	2020	Dalhart	18	20DAL-R	95	Red
NDTX059759-3R/Y Pinto	2	2020	Dalhart	18	20DAL-R	95	Red

NDTX059759-3R/Y Pinto	1	2020	Springlake	4	20SPR-C	136	Red
NDTX059759-3R/Y Pinto	2	2020	Springlake	4	20SPR-C	129	Red
NDTX059759-3R/Y Pinto	1	2020	Springlake	18	20SPR-R	80	Red
NDTX059759-3R/Y Pinto	2	2020	Springlake	18	20SPR-R	80	Red
NDTX059759-3R/Y Pinto	1	2021	Springlake	4	21SPR-C	126	Red
NDTX059759-3R/Y Pinto	2	2021	Springlake	4	21SPR-C	133	Red
NDTX059759-3R/Y Pinto	1	2021	Springlake	18	21SPR-R	91	Red
NDTX059759-3R/Y Pinto	2	2021	Springlake	18	21SPR-R	91	Red
NDTX059761-1R/R	1	2019	Dalhart	18	19DAL-R	84	Red
NDTX059761-1R/R	2	2019	Dalhart	18	19DAL-R	98	Red
NDTX059761-1R/R	1	2020	Dalhart	4	20DAL-C	144	Red
NDTX059761-1R/R	2	2020	Dalhart	4	20DAL-C	151	Red
NDTX059761-1R/R	1	2020	Dalhart	18	20DAL-R	95	Red
NDTX059761-1R/R	2	2020	Dalhart	18	20DAL-R	95	Red
NDTX059761-1R/R	1	2020	Springlake	4	20SPR-C	129	Red
NDTX059761-1R/R	2	2020	Springlake	4	20SPR-C	108	Red
NDTX059761-1R/R	1	2020	Springlake	18	20SPR-R	73	Red
NDTX059761-1R/R	2	2020	Springlake	18	20SPR-R	73	Red
NDTX059761-1R/R	1	2021	Springlake	4	21SPR-C	112	Red
NDTX059761-1R/R	2	2021	Springlake	4	21SPR-C	126	Red
NDTX059761-1R/R	1	2021	Springlake	18	21SPR-R	70	Red
NDTX059761-1R/R	2	2021	Springlake	18	21SPR-R	84	Red
NDTX059775-1W	1	2019	Dalhart	18	19DAL-R	77	Yellow
NDTX059775-1W	2	2019	Dalhart	18	19DAL-R	98	Yellow
NDTX059775-1W	1	2020	Dalhart	4	20DAL-C	133	Yellow
NDTX059775-1W	2	2020	Dalhart	4	20DAL-C	151	Yellow
NDTX059775-1W	1	2020	Dalhart	18	20DAL-R	102	Yellow
NDTX059775-1W	2	2020	Dalhart	18	20DAL-R	117	Yellow
NDTX059775-1W	1	2020	Springlake	4	20SPR-C	108	Yellow
NDTX059775-1W	2	2020	Springlake	4	20SPR-C	101	Yellow
NDTX059775-1W	1	2020	Springlake	18	20SPR-R	80	Yellow
NDTX059775-1W	2	2020	Springlake	18	20SPR-R	59	Yellow
NDTX059775-1W	1	2021	Springlake	4	21SPR-C	119	Yellow
NDTX059775-1W	2	2021	Springlake	4	21SPR-C		Yellow
NDTX059775-1W	1	2021	Springlake	18	21SPR-R	70	Yellow
NDTX059775-1W	2	2021	Springlake	18	21SPR-R		Yellow
NDTX059828-2W	1	2019	Dalhart	18	19DAL-R	56	Chipper
NDTX059828-2W	2	2019	Dalhart	18	19DAL-R	56	Chipper
NDTX059828-2W	1	2020	Dalhart	4	20DAL-C	102	Chipper
NDTX059828-2W	2	2020	Dalhart	4	20DAL-C	95	Chipper
NDTX059828-2W	1	2020	Dalhart	18	20DAL-R	67	Chipper
NDTX059828-2W	2	2020	Dalhart	18	20DAL-R	53	Chipper
NDTX059828-2W	1	2020	Springlake	4	20SPR-C	101	Chipper
NDTX059828-2W	2	2020	Springlake	4	20SPR-C	94	Chipper
NDTX059828-2W	1	2020	Springlake	18	20SPR-R	33	Chipper
NDTX059828-2W	2	2020	Springlake	18	20SPR-R	45	Chipper
NDTX059828-2W	1	2021	Springlake	4	21SPR-C		Chipper

NDTX059828-2W	2	2021	Springlake	4	21SPR-C	112	Chipper
NDTX059828-2W	1	2021	Springlake	18	21SPR-R	42	Chipper
NDTX059828-2W	2	2021	Springlake	18	21SPR-R	42	Chipper
NDTX059886S-1Y/Y	1	2019	Dalhart	18	19DAL-R	63	Yellow
NDTX059886S-1Y/Y	2	2019	Dalhart	18	19DAL-R	56	Yellow
NDTX059886s-1Y/Y	1	2020	Dalhart	4	20DAL-C	133	Yellow
NDTX059886s-1Y/Y	2	2020	Dalhart	4	20DAL-C	133	Yellow
NDTX059886S-1Y/Y	1	2020	Dalhart	18	20DAL-R	74	Yellow
NDTX059886S-1Y/Y	2	2020	Dalhart	18	20DAL-R	74	Yellow
NDTX059886s-1Y/Y	1	2020	Springlake	4	20SPR-C	122	Yellow
NDTX059886s-1Y/Y	2	2020	Springlake	4	20SPR-C	101	Yellow
NDTX059886S-1Y/Y	1	2020	Springlake	18	20SPR-R	66	Yellow
NDTX059886S-1Y/Y	2	2020	Springlake	18	20SPR-R	66	Yellow
NDTX059886s-1Y/Y	1	2021	Springlake	4	21SPR-C	119	Yellow
NDTX059886s-1Y/Y	2	2021	Springlake	4	21SPR-C	99	Yellow
NDTX059886s-1Y/Y	1	2021	Springlake	18	21SPR-R		Yellow
NDTX059886s-1Y/Y	2	2021	Springlake	18	21SPR-R	49	Yellow
NDTX060700C-1W	1	2019	Dalhart	18	19DAL-R	70	Chipper
NDTX060700C-1W	2	2019	Dalhart	18	19DAL-R	70	Chipper
NDTX060700C-1W	1	2020	Dalhart	4	20DAL-C		Chipper
NDTX060700C-1W	2	2020	Dalhart	4	20DAL-C	88	Chipper
NDTX060700C-1W	1	2020	Dalhart	18	20DAL-R	53	Chipper
NDTX060700C-1W	2	2020	Dalhart	18	20DAL-R	48	Chipper
NDTX060700C-1W	1	2020	Springlake	4	20SPR-C	115	Chipper
NDTX060700C-1W	2	2020	Springlake	4	20SPR-C	122	Chipper
NDTX060700C-1W	1	2020	Springlake	18	20SPR-R	45	Chipper
NDTX060700C-1W	2	2020	Springlake	18	20SPR-R	45	Chipper
NDTX060700C-1W	1	2021	Springlake	4	21SPR-C	126	Chipper
NDTX060700C-1W	2	2021	Springlake	4	21SPR-C	126	Chipper
NDTX060700C-1W	1	2021	Springlake	18	21SPR-R	56	Chipper
NDTX060700C-1W	2	2021	Springlake	18	21SPR-R	63	Chipper
NDTX071109C-1W	1	2019	Dalhart	18	19DAL-R	84	Chipper
NDTX071109C-1W	2	2019	Dalhart	18	19DAL-R	63	Chipper
NDTX071109C-1W	1	2020	Dalhart	4	20DAL-C	172	Chipper
NDTX071109C-1W	2	2020	Dalhart	4	20DAL-C	133	Chipper
NDTX071109C-1W	1	2020	Dalhart	18	20DAL-R	102	Chipper
NDTX071109C-1W	2	2020	Dalhart	18	20DAL-R	87	Chipper
NDTX071109C-1W	1	2020	Springlake	4	20SPR-C	157	Chipper
NDTX071109C-1W	2	2020	Springlake	4	20SPR-C	115	Chipper
NDTX071109C-1W	1	2020	Springlake	18	20SPR-R	80	Chipper
NDTX071109C-1W	2	2020	Springlake	18	20SPR-R	73	Chipper
NDTX071109C-1W	1	2021	Springlake	4	21SPR-C	126	Chipper
NDTX071109C-1W	2	2021	Springlake	4	21SPR-C		Chipper
NDTX071109C-1W	1	2021	Springlake	18	21SPR-R	98	Chipper
NDTX071109C-1W	2	2021	Springlake	18	21SPR-R	105	Chipper
NDTX071217CB-1W/Y	1	2019	Dalhart	18	19DAL-R	77	Yellow
NDTX071217CB-1W/Y	2	2019	Dalhart	18	19DAL-R	63	Yellow

NDTX071217CB-1W/Y	1	2020	Dalhart	4	20DAL-C	140	Yellow
NDTX071217CB-1W/Y	2	2020	Dalhart	4	20DAL-C	133	Yellow
NDTX071217CB-1W/Y	1	2020	Dalhart	18	20DAL-R	95	Yellow
NDTX071217CB-1W/Y	2	2020	Dalhart	18	20DAL-R	95	Yellow
NDTX071217CB-1W/Y	1	2020	Springlake	4	20SPR-C		Yellow
NDTX071217CB-1W/Y	2	2020	Springlake	4	20SPR-C	136	Yellow
NDTX071217CB-1W/Y	1	2020	Springlake	18	20SPR-R	87	Yellow
NDTX071217CB-1W/Y	2	2020	Springlake	18	20SPR-R	80	Yellow
NDTX071217CB-1W/Y	1	2021	Springlake	4	21SPR-C	126	Yellow
NDTX071217CB-1W/Y	2	2021	Springlake	4	21SPR-C	133	Yellow
NDTX071217CB-1W/Y	1	2021	Springlake	18	21SPR-R	91	Yellow
NDTX071217CB-1W/Y	2	2021	Springlake	18	21SPR-R	91	Yellow
NDTX071258B-1R	1	2019	Dalhart	18	19DAL-R	56	Red
NDTX071258B-1R	2	2019	Dalhart	18	19DAL-R	63	Red
NDTX071258B-1R	1	2020	Dalhart	4	20DAL-C		Red
NDTX071258B-1R	2	2020	Dalhart	4	20DAL-C		Red
NDTX071258B-1R	1	2020	Dalhart	18	20DAL-R	60	Red
NDTX071258B-1R	2	2020	Dalhart	18	20DAL-R	60	Red
NDTX071258B-1R	1	2020	Springlake	4	20SPR-C	129	Red
NDTX071258B-1R	2	2020	Springlake	4	20SPR-C	115	Red
NDTX071258B-1R	1	2020	Springlake	18	20SPR-R	52	Red
NDTX071258B-1R	2	2020	Springlake	18	20SPR-R	45	Red
NDTX071258B-1R	1	2021	Springlake	4	21SPR-C	112	Red
NDTX071258B-1R	2	2021	Springlake	4	21SPR-C	112	Red
NDTX071258B-1R	1	2021	Springlake	18	21SPR-R	56	Red
NDTX071258B-1R	2	2021	Springlake	18	21SPR-R	56	Red
NDTX081451CBs-1Y/Y	1	2019	Dalhart	18	19DAL-R	63	Yellow
NDTX081451CBs-1Y/Y	2	2019	Dalhart	18	19DAL-R	63	Yellow
NDTX081451CBs-1Y/Y	1	2020	Dalhart	4	20DAL-C	133	Yellow
NDTX081451CBs-1Y/Y	2	2020	Dalhart	4	20DAL-C	133	Yellow
NDTX081451CBs-1Y/Y	1	2020	Dalhart	18	20DAL-R	74	Yellow
NDTX081451CBs-1Y/Y	2	2020	Dalhart	18	20DAL-R	67	Yellow
NDTX081451CBs-1Y/Y	1	2020	Springlake	4	20SPR-C	122	Yellow
NDTX081451CBs-1Y/Y	2	2020	Springlake	4	20SPR-C	101	Yellow
NDTX081451CBs-1Y/Y	1	2020	Springlake	18	20SPR-R	45	Yellow
NDTX081451CBs-1Y/Y	2	2020	Springlake	18	20SPR-R	45	Yellow
NDTX081451CBs-1Y/Y	1	2021	Springlake	4	21SPR-C	126	Yellow
NDTX081451CBs-1Y/Y	2	2021	Springlake	4	21SPR-C	126	Yellow
NDTX081451CBs-1Y/Y	1	2021	Springlake	18	21SPR-R	70	Yellow
NDTX081451CBs-1Y/Y	2	2021	Springlake	18	21SPR-R	84	Yellow
NDTX081618-1P/P	1	2019	Dalhart	18	19DAL-R	63	Purple
NDTX081618-1P/P	2	2019	Dalhart	18	19DAL-R	84	Purple
NDTX081618-1P/P	1	2020	Dalhart	4	20DAL-C	123	Purple
NDTX081618-1P/P	2	2020	Dalhart	4	20DAL-C		Purple
NDTX081618-1P/P	1	2020	Dalhart	18	20DAL-R	74	Purple
NDTX081618-1P/P	2	2020	Dalhart	18	20DAL-R	81	Purple
NDTX081618-1P/P	1	2020	Springlake	4	20SPR-C	129	Purple

NDTX081618-1P/P	2	2020	Springlake	4	20SPR-C	122	Purple
NDTX081618-1P/P	1	2020	Springlake	18	20SPR-R	80	Purple
NDTX081618-1P/P	2	2020	Springlake	18	20SPR-R	73	Purple
NDTX081618-1P/P	1	2021	Springlake	4	21SPR-C	126	Purple
NDTX081618-1P/P	2	2021	Springlake	4	21SPR-C	140	Purple
NDTX081618-1P/P	1	2021	Springlake	18	21SPR-R	84	Purple
NDTX081618-1P/P	2	2021	Springlake	18	21SPR-R	77	Purple
NDTX081644-CAB-2W	1	2019	Dalhart	18	19DAL-R	63	Chipper
NDTX081644-CAB-2W	2	2019	Dalhart	18	19DAL-R	63	Chipper
NDTX081644-CAB-2W	1	2020	Dalhart	4	20DAL-C		Chipper
NDTX081644-CAB-2W	2	2020	Dalhart	4	20DAL-C	81	Chipper
NDTX081644-CAB-2W	1	2020	Dalhart	18	20DAL-R	60	Chipper
NDTX081644-CAB-2W	2	2020	Dalhart	18	20DAL-R	81	Chipper
NDTX081644-CAB-2W	1	2020	Springlake	4	20SPR-C	108	Chipper
NDTX081644-CAB-2W	2	2020	Springlake	4	20SPR-C	122	Chipper
NDTX081644-CAB-2W	1	2020	Springlake	18	20SPR-R	59	Chipper
NDTX081644-CAB-2W	2	2020	Springlake	18	20SPR-R	59	Chipper
NDTX081644-CAB-2W	1	2021	Springlake	4	21SPR-C	133	Chipper
NDTX081644-CAB-2W	2	2021	Springlake	4	21SPR-C	119	Chipper
NDTX081644-CAB-2W	1	2021	Springlake	18	21SPR-R	84	Chipper
NDTX081644-CAB-2W	2	2021	Springlake	18	21SPR-R		Chipper
NDTX081648CB-13W	1	2019	Dalhart	18	19DAL-R	56	Chipper
NDTX081648CB-13W	2	2019	Dalhart	18	19DAL-R	56	Chipper
NDTX081648CB-13W	1	2020	Dalhart	4	20DAL-C	81	Chipper
NDTX081648CB-13W	2	2020	Dalhart	4	20DAL-C		Chipper
NDTX081648CB-13W	1	2020	Dalhart	18	20DAL-R	67	Chipper
NDTX081648CB-13W	2	2020	Dalhart	18	20DAL-R	74	Chipper
NDTX081648CB-13W	1	2020	Springlake	4	20SPR-C	94	Chipper
NDTX081648CB-13W	2	2020	Springlake	4	20SPR-C	101	Chipper
NDTX081648CB-13W	1	2020	Springlake	18	20SPR-R	45	Chipper
NDTX081648CB-13W	2	2020	Springlake	18	20SPR-R	52	Chipper
NDTX081648CB-13W	1	2021	Springlake	4	21SPR-C	112	Chipper
NDTX081648CB-13W	2	2021	Springlake	4	21SPR-C	92	Chipper
NDTX081648CB-13W	1	2021	Springlake	18	21SPR-R	70	Chipper
NDTX081648CB-13W	2	2021	Springlake	18	21SPR-R	63	Chipper
NDTX081648CB-1W	1	2019	Dalhart	18	19DAL-R	105	Chipper
NDTX081648CB-1W	2	2019	Dalhart	18	19DAL-R	119	Chipper
NDTX081648CB-1W	1	2020	Dalhart	4	20DAL-C	133	Chipper
NDTX081648CB-1W	2	2020	Dalhart	4	20DAL-C	133	Chipper
NDTX081648CB-1W	1	2020	Dalhart	18	20DAL-R	102	Chipper
NDTX081648CB-1W	2	2020	Dalhart	18	20DAL-R	95	Chipper
NDTX081648CB-1W	1	2020	Springlake	4	20SPR-C	115	Chipper
NDTX081648CB-1W	2	2020	Springlake	4	20SPR-C	136	Chipper
NDTX081648CB-1W	1	2020	Springlake	18	20SPR-R	80	Chipper
NDTX081648CB-1W	2	2020	Springlake	18	20SPR-R	95	Chipper
NDTX081648CB-1W	1	2021	Springlake	4	21SPR-C	133	Chipper
NDTX081648CB-1W	2	2021	Springlake	4	21SPR-C	140	Chipper

NDTX081648CB-1W	1	2021	Springlake	18	21SPR-R	105	Chipper
NDTX081648CB-1W	2	2021	Springlake	18	21SPR-R	119	Chipper
NDTX081648CB-4W	1	2019	Dalhart	18	19DAL-R	77	Chipper
NDTX081648CB-4W	2	2019	Dalhart	18	19DAL-R	119	Chipper
NDTX081648CB-4W	1	2020	Dalhart	4	20DAL-C	133	Chipper
NDTX081648CB-4W	2	2020	Dalhart	4	20DAL-C	133	Chipper
NDTX081648CB-4W	1	2020	Dalhart	18	20DAL-R	129	Chipper
NDTX081648CB-4W	2	2020	Dalhart	18	20DAL-R	81	Chipper
NDTX081648CB-4W	1	2020	Springlake	4	20SPR-C	136	Chipper
NDTX081648CB-4W	2	2020	Springlake	4	20SPR-C	122	Chipper
NDTX081648CB-4W	1	2020	Springlake	18	20SPR-R	87	Chipper
NDTX081648CB-4W	2	2020	Springlake	18	20SPR-R	87	Chipper
NDTX081648CB-4W	1	2021	Springlake	4	21SPR-C	133	Chipper
NDTX081648CB-4W	2	2021	Springlake	4	21SPR-C	133	Chipper
NDTX081648CB-4W	1	2021	Springlake	18	21SPR-R	105	Chipper
NDTX081648CB-4W	2	2021	Springlake	18	21SPR-R	98	Chipper
NDTX091886-3P/P	1	2019	Dalhart	18	19DAL-R	77	Purple
NDTX091886-3P/P	2	2019	Dalhart	18	19DAL-R	91	Purple
NDTX091886-3P/P	1	2020	Dalhart	4	20DAL-C	140	Purple
NDTX091886-3P/P	2	2020	Dalhart	4	20DAL-C	140	Purple
NDTX091886-3P/P	1	2020	Dalhart	18	20DAL-R	102	Purple
NDTX091886-3P/P	2	2020	Dalhart	18	20DAL-R	102	Purple
NDTX091886-3P/P	1	2020	Springlake	4	20SPR-C	129	Purple
NDTX091886-3P/P	2	2020	Springlake	4	20SPR-C	129	Purple
NDTX091886-3P/P	1	2020	Springlake	18	20SPR-R	95	Purple
NDTX091886-3P/P	2	2020	Springlake	18	20SPR-R	87	Purple
NDTX091886-3P/P	1	2021	Springlake	4	21SPR-C		Purple
NDTX091886-3P/P	2	2021	Springlake	4	21SPR-C		Purple
NDTX091886-3P/P	1	2021	Springlake	18	21SPR-R	112	Purple
NDTX091886-3P/P	2	2021	Springlake	18	21SPR-R	105	Purple
NDTX091908AB-2W	1	2019	Dalhart	18	19DAL-R	91	Chipper
NDTX091908AB-2W	2	2019	Dalhart	18	19DAL-R	84	Chipper
NDTX091908AB-2W	1	2020	Dalhart	4	20DAL-C	144	Chipper
NDTX091908AB-2W	2	2020	Dalhart	4	20DAL-C	140	Chipper
NDTX091908AB-2W	1	2020	Dalhart	18	20DAL-R	74	Chipper
NDTX091908AB-2W	2	2020	Dalhart	18	20DAL-R	81	Chipper
NDTX091908AB-2W	1	2020	Springlake	4	20SPR-C	108	Chipper
NDTX091908AB-2W	2	2020	Springlake	4	20SPR-C	122	Chipper
NDTX091908AB-2W	1	2020	Springlake	18	20SPR-R	87	Chipper
NDTX091908AB-2W	2	2020	Springlake	18	20SPR-R	80	Chipper
NDTX091908AB-2W	1	2021	Springlake	4	21SPR-C	174	Chipper
NDTX091908AB-2W	2	2021	Springlake	4	21SPR-C	154	Chipper
NDTX091908AB-2W	1	2021	Springlake	18	21SPR-R	105	Chipper
NDTX091908AB-2W	2	2021	Springlake	18	21SPR-R	98	Chipper
NDTX092237C-2P/W	1	2019	Dalhart	18	19DAL-R	119	Red
NDTX092237C-2P/W	2	2019	Dalhart	18	19DAL-R	126	Red
NDTX092237C-2P/W	1	2020	Dalhart	4	20DAL-C	165	Red

NDTX092237C-2P/W	2	2020	Dalhart	4	20DAL-C	186	Red
NDTX092237C-2P/W	1	2020	Dalhart	18	20DAL-R		Red
NDTX092237C-2P/W	2	2020	Dalhart	18	20DAL-R	129	Red
NDTX092237C-2P/W	1	2020	Springlake	4	20SPR-C	150	Red
NDTX092237C-2P/W	2	2020	Springlake	4	20SPR-C	143	Red
NDTX092237C-2P/W	1	2020	Springlake	18	20SPR-R	87	Red
NDTX092237C-2P/W	2	2020	Springlake	18	20SPR-R	87	Red
NDTX092237C-2P/W	1	2021	Springlake	4	21SPR-C	154	Red
NDTX092237C-2P/W	2	2021	Springlake	4	21SPR-C	140	Red
NDTX092237C-2P/W	1	2021	Springlake	18	21SPR-R	119	Red
NDTX092237C-2P/W	2	2021	Springlake	18	21SPR-R	91	Red
NDTX092237C-2P/WP	1	2022	Dalhart	4	22DAL-C	137	Purple
NDTX092237C-2P/WP	2	2022	Dalhart	4	22DAL-C	116	Purple
NDTX092237C-2P/WP	1	2022	Dalhart	18	22DAL-R	85	Purple
NDTX092237C-2P/WP	2	2022	Dalhart	18	22DAL-R	92	Purple
NDTX092237C-2P/WP	1	2022	Springlake	4	22SPR-C	111	Purple
NDTX092237C-2P/WP	2	2022	Springlake	4	22SPR-C		Purple
NDTX092237C-2P/WP	1	2022	Springlake	18	22SPR-R	52	Purple
NDTX092237C-2P/WP	2	2022	Springlake	18	22SPR-R	52	Purple
NDTX092238Cs-1P/W	1	2019	Dalhart	18	19DAL-R	84	Purple
NDTX092238Cs-1P/W	2	2019	Dalhart	18	19DAL-R	70	Purple
NDTX092238Cs-1P/W	1	2020	Dalhart	4	20DAL-C	151	Purple
NDTX092238Cs-1P/W	2	2020	Dalhart	4	20DAL-C	133	Purple
NDTX092238Cs-1P/W	1	2020	Dalhart	18	20DAL-R	74	Purple
NDTX092238Cs-1P/W	2	2020	Dalhart	18	20DAL-R	74	Purple
NDTX092238Cs-1P/W	1	2020	Springlake	4	20SPR-C	143	Purple
NDTX092238Cs-1P/W	2	2020	Springlake	4	20SPR-C	136	Purple
NDTX092238Cs-1P/W	1	2020	Springlake	18	20SPR-R	52	Purple
NDTX092238Cs-1P/W	2	2020	Springlake	18	20SPR-R	66	Purple
NDTX092238Cs-1P/W	1	2021	Springlake	4	21SPR-C	154	Purple
NDTX092238Cs-1P/W	2	2021	Springlake	4	21SPR-C	181	Purple
NDTX092238Cs-1P/W	1	2021	Springlake	18	21SPR-R	70	Purple
NDTX092238Cs-1P/W	2	2021	Springlake	18	21SPR-R	70	Purple
NDTX12248Y-1R	1	2022	Dalhart	4	22DAL-C	193	Red
NDTX12248Y-1R	2	2022	Dalhart	4	22DAL-C	158	Red
NDTX12248Y-1R	1	2022	Dalhart	18	22DAL-R	106	Red
NDTX12248Y-1R	2	2022	Dalhart	18	22DAL-R	92	Red
NDTX12248Y-1R	1	2022	Springlake	4	22SPR-C	111	Red
NDTX12248Y-1R	2	2022	Springlake	4	22SPR-C	111	Red
NDTX12248Y-1R	1	2022	Springlake	18	22SPR-R	59	Red
NDTX12248Y-1R	2	2022	Springlake	18	22SPR-R	52	Red
NDTX1244-3W/Y	1	2022	Dalhart	4	22DAL-C	95	Yellow
NDTX1244-3W/Y	2	2022	Dalhart	4	22DAL-C	95	Yellow
NDTX1244-3W/Y	1	2022	Dalhart	18	22DAL-R	85	Yellow
NDTX1244-3W/Y	2	2022	Dalhart	18	22DAL-R	85	Yellow
NDTX1244-3W/Y	1	2022	Springlake	4	22SPR-C	104	Yellow
NDTX1244-3W/Y	2	2022	Springlake	4	22SPR-C	90	Yellow

NDTX1244-3W/Y	1	2022	Springlake	18	22SPR-R	59	Yellow
NDTX1244-3W/Y	2	2022	Springlake	18	22SPR-R	52	Yellow
NDTX1246-5W/Y	1	2022	Dalhart	4	22DAL-C	116	Yellow
NDTX1246-5W/Y	2	2022	Dalhart	4	22DAL-C	116	Yellow
NDTX1246-5W/Y	1	2022	Dalhart	18	22DAL-R	85	Yellow
NDTX1246-5W/Y	2	2022	Dalhart	18	22DAL-R	85	Yellow
NDTX1246-5W/Y	1	2022	Springlake	4	22SPR-C	104	Yellow
NDTX1246-5W/Y	2	2022	Springlake	4	22SPR-C	104	Yellow
NDTX1246-5W/Y	1	2022	Springlake	18	22SPR-R		Yellow
NDTX1246-5W/Y	2	2022	Springlake	18	22SPR-R	59	Yellow
NDTX14156-2RWpinto/Y	1	2022	Dalhart	18	22DAL-R		Red
NDTX14156-2RWpinto/Y	2	2022	Dalhart	18	22DAL-R		Red
NDTX14156-2RWpinto/Y	1	2022	Springlake	4	22SPR-C	111	Red
NDTX14156-2RWpinto/Y	2	2022	Springlake	4	22SPR-C	111	Red
NDTX14156-2RWpinto/Y	1	2022	Springlake	18	22SPR-R	52	Red
NDTX14156-2RWpinto/Y	2	2022	Springlake	18	22SPR-R	52	Red
NDTX14156-3P/WP	1	2022	Dalhart	4	22DAL-C	116	Purple
NDTX14156-3P/WP	2	2022	Dalhart	4	22DAL-C	137	Purple
NDTX14156-3P/WP	1	2022	Dalhart	18	22DAL-R	92	Purple
NDTX14156-3P/WP	2	2022	Dalhart	18	22DAL-R	106	Purple
NDTX14156-3P/WP	1	2022	Springlake	4	22SPR-C	153	Purple
NDTX14156-3P/WP	2	2022	Springlake	4	22SPR-C	111	Purple
NDTX14156-3P/WP	1	2022	Springlake	18	22SPR-R	66	Purple
NDTX14156-3P/WP	2	2022	Springlake	18	22SPR-R	73	Purple
NDTX14156A-3RWpinto/Y	1	2022	Dalhart	18	22DAL-R		Red
NDTX14156A-3RWpinto/Y	2	2022	Dalhart	18	22DAL-R		Red
NDTX14156A-3RWpinto/Y	1	2022	Springlake	4	22SPR-C	139	Red
NDTX14156A-3RWpinto/Y	2	2022	Springlake	4	22SPR-C	93	Red
NDTX14156A-3RWpinto/Y	1	2022	Springlake	18	22SPR-R	52	Red
NDTX14156A-3RWpinto/Y	2	2022	Springlake	18	22SPR-R	41	Red
NDTX14247CAB-1W	1	2022	Dalhart	4	22DAL-C	137	Chipper
NDTX14247CAB-1W	2	2022	Dalhart	4	22DAL-C	137	Chipper
NDTX14247CAB-1W	1	2022	Dalhart	18	22DAL-R	92	Chipper
NDTX14247CAB-1W	2	2022	Dalhart	18	22DAL-R	92	Chipper
NDTX14247CAB-1W	1	2022	Springlake	4	22SPR-C	102	Chipper
NDTX14247CAB-1W	2	2022	Springlake	4	22SPR-C	72	Chipper
NDTX14247CAB-1W	1	2022	Springlake	18	22SPR-R	64	Chipper
NDTX14247CAB-1W	2	2022	Springlake	18	22SPR-R	55	Chipper
NDTX14247CAB-2W	1	2022	Dalhart	4	22DAL-C	130	Chipper
NDTX14247CAB-2W	2	2022	Dalhart	4	22DAL-C	130	Chipper
NDTX14247CAB-2W	1	2022	Dalhart	18	22DAL-R	92	Chipper
NDTX14247CAB-2W	2	2022	Dalhart	18	22DAL-R	92	Chipper
NDTX14247CAB-2W	1	2022	Springlake	4	22SPR-C	102	Chipper
NDTX14247CAB-2W	2	2022	Springlake	4	22SPR-C	65	Chipper
NDTX14247CAB-2W	1	2022	Springlake	18	22SPR-R	57	Chipper
NDTX14247CAB-2W	2	2022	Springlake	18	22SPR-R	55	Chipper
NDTX14263BC-3W	1	2022	Dalhart	4	22DAL-C	116	Chipper

NDTX14263BC-3W	2	2022	Dalhart	4	22DAL-C	137	Chipper
NDTX14263BC-3W	1	2022	Dalhart	18	22DAL-R	92	Chipper
NDTX14263BC-3W	2	2022	Dalhart	18	22DAL-R	85	Chipper
NDTX14263BC-3W	1	2022	Springlake	4	22SPR-C	102	Chipper
NDTX14263BC-3W	2	2022	Springlake	4	22SPR-C		Chipper
NDTX14263BC-3W	1	2022	Springlake	18	22SPR-R	50	Chipper
NDTX14263BC-3W	2	2022	Springlake	18	22SPR-R	48	Chipper
NDTX14362AB-1W	1	2022	Dalhart	4	22DAL-C	151	Chipper
NDTX14362AB-1W	2	2022	Dalhart	4	22DAL-C	137	Chipper
NDTX14362AB-1W	1	2022	Dalhart	18	22DAL-R	85	Chipper
NDTX14362AB-1W	2	2022	Dalhart	18	22DAL-R	78	Chipper
NDTX14362AB-1W	1	2022	Springlake	4	22SPR-C	102	Chipper
NDTX14362AB-1W	2	2022	Springlake	4	22SPR-C	93	Chipper
NDTX14362AB-1W	1	2022	Springlake	18	22SPR-R	43	Chipper
NDTX14362AB-1W	2	2022	Springlake	18	22SPR-R	41	Chipper
NDTX1482YB-1W	1	2022	Dalhart	4	22DAL-C	137	Chipper
NDTX1482YB-1W	2	2022	Dalhart	4	22DAL-C	130	Chipper
NDTX1482YB-1W	1	2022	Dalhart	18	22DAL-R	106	Chipper
NDTX1482YB-1W	2	2022	Dalhart	18	22DAL-R	113	Chipper
NDTX1482YB-1W	1	2022	Springlake	4	22SPR-C	102	Chipper
NDTX1482YB-1W	2	2022	Springlake	4	22SPR-C	93	Chipper
NDTX1482YB-1W	1	2022	Springlake	18	22SPR-R	64	Chipper
NDTX1482YB-1W	2	2022	Springlake	18	22SPR-R	62	Chipper
NDTX1753Y-1R	1	2022	Dalhart	4	22DAL-C	137	Red
NDTX1753Y-1R	2	2022	Dalhart	4	22DAL-C	116	Red
NDTX1753Y-1R	1	2022	Dalhart	18	22DAL-R	78	Red
NDTX1753Y-1R	2	2022	Dalhart	18	22DAL-R	85	Red
NDTX1753Y-1R	1	2022	Springlake	4	22SPR-C	111	Red
NDTX1753Y-1R	2	2022	Springlake	4	22SPR-C	111	Red
NDTX1753Y-1R	1	2022	Springlake	18	22SPR-R	52	Red
NDTX1753Y-1R	2	2022	Springlake	18	22SPR-R	52	Red
NDTX1759s-1R/Y	1	2022	Dalhart	4	22DAL-C	95	Red
NDTX1759s-1R/Y	2	2022	Dalhart	4	22DAL-C	116	Red
NDTX1759s-1R/Y	1	2022	Dalhart	18	22DAL-R	78	Red
NDTX1759s-1R/Y	2	2022	Dalhart	18	22DAL-R	85	Red
NDTX1759s-1R/Y	1	2022	Springlake	4	22SPR-C	104	Red
NDTX1759s-1R/Y	2	2022	Springlake	4	22SPR-C	111	Red
NDTX1759s-1R/Y	1	2022	Springlake	18	22SPR-R	73	Red
NDTX1759s-1R/Y	2	2022	Springlake	18	22SPR-R	80	Red
NDTX1791-1Ru	1	2022	Dalhart	4	22DAL-C	136	Russet
NDTX1791-1Ru	2	2022	Dalhart	4	22DAL-C	143	Russet
NDTX1791-1Ru	1	2022	Dalhart	18	22DAL-R	91	Russet
NDTX1791-1Ru	2	2022	Dalhart	18	22DAL-R	70	Russet
NDTX1791-1Ru	1	2022	Springlake	4	22SPR-C	99	Russet
NDTX1791-1Ru	2	2022	Springlake	4	22SPR-C	93	Russet
NDTX1791-1Ru	1	2022	Springlake	18	22SPR-R	47	Russet
NDTX1791-1Ru	2	2022	Springlake	18	22SPR-R	41	Russet

NDTX1824Y-1R	1	2022	Dalhart	4	22DAL-C	116	Red
NDTX1824Y-1R	2	2022	Dalhart	4	22DAL-C	95	Red
NDTX1824Y-1R	1	2022	Dalhart	18	22DAL-R	71	Red
NDTX1824Y-1R	2	2022	Dalhart	18	22DAL-R	85	Red
NDTX1824Y-1R	1	2022	Springlake	4	22SPR-C	111	Red
NDTX1824Y-1R	2	2022	Springlake	4	22SPR-C	97	Red
NDTX1824Y-1R	1	2022	Springlake	18	22SPR-R	52	Red
NDTX1824Y-1R	2	2022	Springlake	18	22SPR-R	59	Red
NDTX1824Y-4R	1	2022	Dalhart	4	22DAL-C	116	Red
NDTX1824Y-4R	2	2022	Dalhart	4	22DAL-C	123	Red
NDTX1824Y-4R	1	2022	Dalhart	18	22DAL-R	78	Red
NDTX1824Y-4R	2	2022	Dalhart	18	22DAL-R	78	Red
NDTX1824Y-4R	1	2022	Springlake	4	22SPR-C	111	Red
NDTX1824Y-4R	2	2022	Springlake	4	22SPR-C	97	Red
NDTX1824Y-4R	1	2022	Springlake	18	22SPR-R	52	Red
NDTX1824Y-4R	2	2022	Springlake	18	22SPR-R	52	Red
NDTX4271-5R	1	2019	Dalhart	18	19DAL-R	56	Red
NDTX4271-5R	2	2019	Dalhart	18	19DAL-R	56	Red
NDTX4271-5R	1	2020	Dalhart	4	20DAL-C	123	Red
NDTX4271-5R	2	2020	Dalhart	4	20DAL-C	133	Red
NDTX4271-5R	1	2020	Dalhart	18	20DAL-R	48	Red
NDTX4271-5R	2	2020	Dalhart	18	20DAL-R	53	Red
NDTX4271-5R	1	2020	Springlake	4	20SPR-C	108	Red
NDTX4271-5R	2	2020	Springlake	4	20SPR-C	122	Red
NDTX4271-5R	1	2020	Springlake	18	20SPR-R	38	Red
NDTX4271-5R	2	2020	Springlake	18	20SPR-R	38	Red
NDTX4271-5R	1	2021	Springlake	4	21SPR-C	133	Red
NDTX4271-5R	2	2021	Springlake	4	21SPR-C	119	Red
NDTX4271-5R	1	2021	Springlake	18	21SPR-R	42	Red
NDTX4271-5R	2	2021	Springlake	18	21SPR-R	49	Red
NDTX4756-1R/Y	1	2019	Dalhart	18	19DAL-R	56	Red
NDTX4756-1R/Y	2	2019	Dalhart	18	19DAL-R	63	Red
NDTX4756-1R/Y	1	2020	Dalhart	4	20DAL-C	151	Red
NDTX4756-1R/Y	2	2020	Dalhart	4	20DAL-C	140	Red
NDTX4756-1R/Y	1	2020	Dalhart	18	20DAL-R	60	Red
NDTX4756-1R/Y	2	2020	Dalhart	18	20DAL-R	67	Red
NDTX4756-1R/Y	1	2020	Springlake	4	20SPR-C	101	Red
NDTX4756-1R/Y	2	2020	Springlake	4	20SPR-C	101	Red
NDTX4756-1R/Y	1	2020	Springlake	18	20SPR-R	66	Red
NDTX4756-1R/Y	2	2020	Springlake	18	20SPR-R	59	Red
NDTX4756-1R/Y	1	2021	Springlake	4	21SPR-C	126	Red
NDTX4756-1R/Y	2	2021	Springlake	4	21SPR-C	126	Red
NDTX4756-1R/Y	1	2021	Springlake	18	21SPR-R	63	Red
NDTX4756-1R/Y	2	2021	Springlake	18	21SPR-R	56	Red
NDTX4784-7R	1	2019	Dalhart	18	19DAL-R	56	Red
NDTX4784-7R	2	2019	Dalhart	18	19DAL-R	56	Red
NDTX4784-7R	1	2020	Dalhart	4	20DAL-C		Red

NDTX4784-7R	2	2020	Dalhart	4	20DAL-C		Red
NDTX4784-7R	1	2020	Dalhart	18	20DAL-R	67	Red
NDTX4784-7R	2	2020	Dalhart	18	20DAL-R	53	Red
NDTX4784-7R	1	2020	Springlake	4	20SPR-C	129	Red
NDTX4784-7R	2	2020	Springlake	4	20SPR-C	122	Red
NDTX4784-7R	1	2020	Springlake	18	20SPR-R	38	Red
NDTX4784-7R	2	2020	Springlake	18	20SPR-R	38	Red
NDTX4784-7R	1	2021	Springlake	4	21SPR-C	126	Red
NDTX4784-7R	2	2021	Springlake	4	21SPR-C	154	Red
NDTX4784-7R	1	2021	Springlake	18	21SPR-R	49	Red
NDTX4784-7R	2	2021	Springlake	18	21SPR-R	56	Red
NDTX4828-2R	1	2019	Dalhart	18	19DAL-R	98	Red
NDTX4828-2R	2	2019	Dalhart	18	19DAL-R	105	Red
NDTX4828-2R	1	2020	Dalhart	4	20DAL-C		Red
NDTX4828-2R	2	2020	Dalhart	4	20DAL-C	144	Red
NDTX4828-2R	1	2020	Dalhart	18	20DAL-R	87	Red
NDTX4828-2R	2	2020	Dalhart	18	20DAL-R	81	Red
NDTX4828-2R	1	2020	Springlake	4	20SPR-C	122	Red
NDTX4828-2R	2	2020	Springlake	4	20SPR-C	101	Red
NDTX4828-2R	1	2020	Springlake	18	20SPR-R	87	Red
NDTX4828-2R	2	2020	Springlake	18	20SPR-R	87	Red
NDTX4828-2R	1	2021	Springlake	4	21SPR-C	119	Red
NDTX4828-2R	2	2021	Springlake	4	21SPR-C	119	Red
NDTX4828-2R	1	2021	Springlake	18	21SPR-R	98	Red
NDTX4828-2R	2	2021	Springlake	18	21SPR-R	84	Red
NDTX4930-5W	1	2019	Dalhart	18	19DAL-R	70	Chipper
NDTX4930-5W	2	2019	Dalhart	18	19DAL-R	56	Chipper
NDTX4930-5W	1	2020	Dalhart	4	20DAL-C	151	Chipper
NDTX4930-5W	2	2020	Dalhart	4	20DAL-C	151	Chipper
NDTX4930-5W	1	2020	Dalhart	18	20DAL-R	87	Chipper
NDTX4930-5W	2	2020	Dalhart	18	20DAL-R	81	Chipper
NDTX4930-5W	1	2020	Springlake	4	20SPR-C	143	Chipper
NDTX4930-5W	2	2020	Springlake	4	20SPR-C	122	Chipper
NDTX4930-5W	1	2020	Springlake	18	20SPR-R	66	Chipper
NDTX4930-5W	2	2020	Springlake	18	20SPR-R	80	Chipper
NDTX4930-5W	1	2021	Springlake	4	21SPR-C	119	Chipper
NDTX4930-5W	2	2021	Springlake	4	21SPR-C	119	Chipper
NDTX4930-5W	1	2021	Springlake	18	21SPR-R	105	Chipper
NDTX4930-5W	2	2021	Springlake	18	21SPR-R	84	Chipper
NDTX5003-2R	1	2019	Dalhart	18	19DAL-R	56	Red
NDTX5003-2R	2	2019	Dalhart	18	19DAL-R	56	Red
NDTX5003-2R	1	2020	Dalhart	4	20DAL-C	123	Red
NDTX5003-2R	2	2020	Dalhart	4	20DAL-C	140	Red
NDTX5003-2R	1	2020	Dalhart	18	20DAL-R	60	Red
NDTX5003-2R	2	2020	Dalhart	18	20DAL-R	60	Red
NDTX5003-2R	1	2020	Springlake	4	20SPR-C	129	Red
NDTX5003-2R	2	2020	Springlake	4	20SPR-C		Red

NDTX5003-2R	1	2020	Springlake	18	20SPR-R	52	Red
NDTX5003-2R	2	2020	Springlake	18	20SPR-R		Red
NDTX5003-2R	1	2021	Springlake	4	21SPR-C		Red
NDTX5003-2R	2	2021	Springlake	4	21SPR-C		Red
NDTX5003-2R	1	2021	Springlake	18	21SPR-R	70	Red
NDTX5003-2R	2	2021	Springlake	18	21SPR-R	70	Red
NDTX5067-2R	1	2019	Dalhart	18	19DAL-R	56	Red
NDTX5067-2R	2	2019	Dalhart	18	19DAL-R	56	Red
NDTX5067-2R	1	2020	Dalhart	4	20DAL-C		Red
NDTX5067-2R	2	2020	Dalhart	4	20DAL-C	133	Red
NDTX5067-2R	1	2020	Dalhart	18	20DAL-R	53	Red
NDTX5067-2R	2	2020	Dalhart	18	20DAL-R	53	Red
NDTX5067-2R	1	2020	Springlake	4	20SPR-C	129	Red
NDTX5067-2R	2	2020	Springlake	4	20SPR-C	122	Red
NDTX5067-2R	1	2020	Springlake	18	20SPR-R	45	Red
NDTX5067-2R	2	2020	Springlake	18	20SPR-R	45	Red
NDTX5067-2R	1	2021	Springlake	4	21SPR-C	140	Red
NDTX5067-2R	2	2021	Springlake	4	21SPR-C	133	Red
NDTX5067-2R	1	2021	Springlake	18	21SPR-R	56	Red
NDTX5067-2R	2	2021	Springlake	18	21SPR-R	56	Red
NDTX5438-11R	1	2019	Dalhart	18	19DAL-R	63	Red
NDTX5438-11R	2	2019	Dalhart	18	19DAL-R	63	Red
NDTX5438-11R	1	2020	Dalhart	4	20DAL-C	133	Red
NDTX5438-11R	2	2020	Dalhart	4	20DAL-C		Red
NDTX5438-11R	1	2020	Dalhart	18	20DAL-R	74	Red
NDTX5438-11R	2	2020	Dalhart	18	20DAL-R		Red
NDTX5438-11R	1	2020	Springlake	4	20SPR-C	122	Red
NDTX5438-11R	2	2020	Springlake	4	20SPR-C	115	Red
NDTX5438-11R	1	2020	Springlake	18	20SPR-R	59	Red
NDTX5438-11R	2	2020	Springlake	18	20SPR-R	52	Red
NDTX5438-11R	1	2021	Springlake	4	21SPR-C		Red
NDTX5438-11R	2	2021	Springlake	4	21SPR-C		Red
NDTX5438-11R	1	2021	Springlake	18	21SPR-R	91	Red
NDTX5438-11R	2	2021	Springlake	18	21SPR-R	77	Red
NDTX6773-1W	1	2019	Dalhart	18	19DAL-R	63	Chipper
NDTX6773-1W	2	2019	Dalhart	18	19DAL-R	70	Chipper
NDTX6773-1W	1	2020	Dalhart	4	20DAL-C	140	Chipper
NDTX6773-1W	2	2020	Dalhart	4	20DAL-C		Chipper
NDTX6773-1W	1	2020	Dalhart	18	20DAL-R	60	Chipper
NDTX6773-1W	2	2020	Dalhart	18	20DAL-R	60	Chipper
NDTX6773-1W	1	2020	Springlake	4	20SPR-C	115	Chipper
NDTX6773-1W	2	2020	Springlake	4	20SPR-C	101	Chipper
NDTX6773-1W	1	2020	Springlake	18	20SPR-R	59	Chipper
NDTX6773-1W	2	2020	Springlake	18	20SPR-R	59	Chipper
NDTX6773-1W	1	2021	Springlake	4	21SPR-C	133	Chipper
NDTX6773-1W	2	2021	Springlake	4	21SPR-C	126	Chipper
NDTX6773-1W	1	2021	Springlake	18	21SPR-R	70	Chipper

NDTX6773-1W	2	2021	Springlake	18	21SPR-R	77	Chipper
NDTX731-1R	1	2019	Dalhart	18	19DAL-R	77	Red
NDTX731-1R	2	2019	Dalhart	18	19DAL-R	77	Red
NDTX731-1R	1	2020	Dalhart	4	20DAL-C		Red
NDTX731-1R	2	2020	Dalhart	4	20DAL-C		Red
NDTX731-1R	1	2020	Dalhart	18	20DAL-R	95	Red
NDTX731-1R	2	2020	Dalhart	18	20DAL-R	102	Red
NDTX731-1R	1	2020	Springlake	4	20SPR-C	122	Red
NDTX731-1R	2	2020	Springlake	4	20SPR-C	122	Red
NDTX731-1R	1	2020	Springlake	18	20SPR-R	80	Red
NDTX731-1R	2	2020	Springlake	18	20SPR-R	73	Red
NDTX731-1R	1	2021	Springlake	4	21SPR-C	133	Red
NDTX731-1R	2	2021	Springlake	4	21SPR-C	133	Red
NDTX731-1R	1	2021	Springlake	18	21SPR-R	70	Red
NDTX731-1R	2	2021	Springlake	18	21SPR-R	84	Red
NDTX7590-3R	1	2019	Dalhart	18	19DAL-R	63	Red
NDTX7590-3R	2	2019	Dalhart	18	19DAL-R	70	Red
NDTX7590-3R	1	2020	Dalhart	4	20DAL-C	123	Red
NDTX7590-3R	2	2020	Dalhart	4	20DAL-C	151	Red
NDTX7590-3R	1	2020	Dalhart	18	20DAL-R	74	Red
NDTX7590-3R	2	2020	Dalhart	18	20DAL-R	95	Red
NDTX7590-3R	1	2020	Springlake	4	20SPR-C	122	Red
NDTX7590-3R	2	2020	Springlake	4	20SPR-C	108	Red
NDTX7590-3R	1	2020	Springlake	18	20SPR-R	59	Red
NDTX7590-3R	2	2020	Springlake	18	20SPR-R	52	Red
NDTX7590-3R	1	2021	Springlake	4	21SPR-C	133	Red
NDTX7590-3R	2	2021	Springlake	4	21SPR-C	126	Red
NDTX7590-3R	1	2021	Springlake	18	21SPR-R	63	Red
NDTX7590-3R	2	2021	Springlake	18	21SPR-R	70	Red
NDTX8773-4Ru	1	2019	Dalhart	18	19DAL-R	112	Russet
NDTX8773-4Ru	2	2019	Dalhart	18	19DAL-R	112	Russet
NDTX8773-4Ru	1	2020	Dalhart	4	20DAL-C	140	Russet
NDTX8773-4Ru	2	2020	Dalhart	4	20DAL-C	151	Russet
NDTX8773-4Ru	1	2020	Dalhart	18	20DAL-R	102	Russet
NDTX8773-4Ru	2	2020	Dalhart	18	20DAL-R	102	Russet
NDTX8773-4Ru	1	2020	Springlake	4	20SPR-C	109	Russet
NDTX8773-4Ru	2	2020	Springlake	4	20SPR-C	116	Russet
NDTX8773-4Ru	1	2020	Springlake	18	20SPR-R	81	Russet
NDTX8773-4Ru	2	2020	Springlake	18	20SPR-R	67	Russet
NDTX8773-4Ru	1	2021	Springlake	4	21SPR-C	114	Russet
NDTX8773-4Ru	2	2021	Springlake	4	21SPR-C	121	Russet
NDTX8773-4Ru	1	2021	Springlake	18	21SPR-R	91	Russet
NDTX8773-4Ru	2	2021	Springlake	18	21SPR-R	91	Russet
NDTX91068-11R	1	2019	Dalhart	18	19DAL-R	70	Red
NDTX91068-11R	2	2019	Dalhart	18	19DAL-R	84	Red
NDTX91068-11R	1	2020	Dalhart	4	20DAL-C	133	Red
NDTX91068-11R	2	2020	Dalhart	4	20DAL-C	140	Red

NDTX91068-11R	1	2020	Dalhart	18	20DAL-R	74	Red
NDTX91068-11R	2	2020	Dalhart	18	20DAL-R	74	Red
NDTX91068-11R	1	2020	Springlake	4	20SPR-C	136	Red
NDTX91068-11R	2	2020	Springlake	4	20SPR-C	136	Red
NDTX91068-11R	1	2020	Springlake	18	20SPR-R	59	Red
NDTX91068-11R	2	2020	Springlake	18	20SPR-R	52	Red
NDTX91068-11R	1	2021	Springlake	4	21SPR-C	133	Red
NDTX91068-11R	2	2021	Springlake	4	21SPR-C		Red
NDTX91068-11R	1	2021	Springlake	18	21SPR-R		Red
NDTX91068-11R	2	2021	Springlake	18	21SPR-R	56	Red
PORTX03PG25-2R/R	1	2019	Dalhart	18	19DAL-R	63	Purple
PORTX03PG25-2R/R	2	2019	Dalhart	18	19DAL-R	63	Purple
PORTX03PG25-2R/R	1	2020	Dalhart	4	20DAL-C	140	Purple
PORTX03PG25-2R/R	2	2020	Dalhart	4	20DAL-C	133	Purple
PORTX03PG25-2R/R	1	2020	Dalhart	18	20DAL-R	67	Purple
PORTX03PG25-2R/R	2	2020	Dalhart	18	20DAL-R	74	Purple
PORTX03PG25-2R/R	1	2020	Springlake	4	20SPR-C	108	Purple
PORTX03PG25-2R/R	2	2020	Springlake	4	20SPR-C	136	Purple
PORTX03PG25-2R/R	1	2020	Springlake	18	20SPR-R	73	Purple
PORTX03PG25-2R/R	2	2020	Springlake	18	20SPR-R	59	Purple
PORTX03PG25-2R/R	1	2021	Springlake	4	21SPR-C		Purple
PORTX03PG25-2R/R	2	2021	Springlake	4	21SPR-C	133	Purple
PORTX03PG25-2R/R	1	2021	Springlake	18	21SPR-R	77	Purple
PORTX03PG25-2R/R	2	2021	Springlake	18	21SPR-R	84	Purple
PTTX05PG07-1W	1	2019	Dalhart	18	19DAL-R	84	Chipper
PTTX05PG07-1W	2	2019	Dalhart	18	19DAL-R	84	Chipper
PTTX05PG07-1W	1	2020	Dalhart	4	20DAL-C	140	Chipper
PTTX05PG07-1W	2	2020	Dalhart	4	20DAL-C		Chipper
PTTX05PG07-1W	1	2020	Dalhart	18	20DAL-R	87	Chipper
PTTX05PG07-1W	2	2020	Dalhart	18	20DAL-R	95	Chipper
PTTX05PG07-1W	1	2020	Springlake	4	20SPR-C	129	Chipper
PTTX05PG07-1W	2	2020	Springlake	4	20SPR-C	143	Chipper
PTTX05PG07-1W	1	2020	Springlake	18	20SPR-R	87	Chipper
PTTX05PG07-1W	2	2020	Springlake	18	20SPR-R	80	Chipper
PTTX05PG07-1W	1	2021	Springlake	4	21SPR-C		Chipper
PTTX05PG07-1W	2	2021	Springlake	4	21SPR-C		Chipper
PTTX05PG07-1W	1	2021	Springlake	18	21SPR-R	105	Chipper
PTTX05PG07-1W	2	2021	Springlake	18	21SPR-R		Chipper
Ranger Russet	1	2022	Dalhart	4	22DAL-C	108	Russet
Ranger Russet	2	2022	Dalhart	4	22DAL-C	108	Russet
Ranger Russet	1	2022	Dalhart	18	22DAL-R	98	Russet
Ranger Russet	2	2022	Dalhart	18	22DAL-R	98	Russet
Ranger Russet	1	2022	Springlake	4	22SPR-C	99	Russet
Ranger Russet	2	2022	Springlake	4	22SPR-C	99	Russet
Ranger Russet	1	2022	Springlake	18	22SPR-R	68	Russet
Ranger Russet	2	2022	Springlake	18	22SPR-R	68	Russet
Red LaSoda	1	2022	Dalhart	4	22DAL-C	137	Red

Red LaSoda	2	2022	Dalhart	4	22DAL-C		Red
Red LaSoda	1	2022	Dalhart	18	22DAL-R	106	Red
Red LaSoda	2	2022	Dalhart	18	22DAL-R	106	Red
Red LaSoda	1	2022	Springlake	4	22SPR-C	111	Red
Red LaSoda	2	2022	Springlake	4	22SPR-C	111	Red
Red LaSoda	1	2022	Springlake	18	22SPR-R	80	Red
Red LaSoda	2	2022	Springlake	18	22SPR-R	73	Red
Reveille Russet	1	2019	Dalhart	18	19DAL-R	140	Russet
Reveille Russet	2	2019	Dalhart	18	19DAL-R		Russet
Reveille Russet	1	2020	Dalhart	4	20DAL-C	172	Russet
Reveille Russet	2	2020	Dalhart	4	20DAL-C	172	Russet
Reveille Russet	1	2020	Dalhart	18	20DAL-R	129	Russet
Reveille Russet	2	2020	Dalhart	18	20DAL-R	129	Russet
Reveille Russet	1	2022	Dalhart	4	22DAL-C	192	Russet
Reveille Russet	2	2022	Dalhart	4	22DAL-C	192	Russet
Reveille Russet	1	2022	Dalhart	18	22DAL-R	136	Russet
Reveille Russet	2	2022	Dalhart	18	22DAL-R	136	Russet
Reveille Russet	1	2020	Springlake	4	20SPR-C		Russet
Reveille Russet	2	2020	Springlake	4	20SPR-C	165	Russet
Reveille Russet	1	2020	Springlake	18	20SPR-R	89	Russet
Reveille Russet	2	2020	Springlake	18	20SPR-R	81	Russet
Reveille Russet	1	2021	Springlake	4	21SPR-C	169	Russet
Reveille Russet	2	2021	Springlake	4	21SPR-C	169	Russet
Reveille Russet	1	2021	Springlake	18	21SPR-R	126	Russet
Reveille Russet	2	2021	Springlake	18	21SPR-R	119	Russet
Reveille Russet	1	2022	Springlake	4	22SPR-C	102	Russet
Reveille Russet	2	2022	Springlake	4	22SPR-C	102	Russet
Reveille Russet	1	2022	Springlake	18	22SPR-R	82	Russet
Reveille Russet	2	2022	Springlake	18	22SPR-R	68	Russet
Rio Rojo	1	2019	Dalhart	18	19DAL-R	77	Red
Rio Rojo	2	2019	Dalhart	18	19DAL-R	91	Red
Rio Rojo	1	2020	Dalhart	4	20DAL-C	140	Red
Rio Rojo	2	2020	Dalhart	4	20DAL-C		Red
Rio Rojo	1	2020	Dalhart	18	20DAL-R	109	Red
Rio Rojo	2	2020	Dalhart	18	20DAL-R	102	Red
Rio Rojo	1	2020	Springlake	4	20SPR-C	136	Red
Rio Rojo	2	2020	Springlake	4	20SPR-C	150	Red
Rio Rojo	1	2020	Springlake	18	20SPR-R	87	Red
Rio Rojo	2	2020	Springlake	18	20SPR-R	87	Red
Rio Rojo	1	2021	Springlake	4	21SPR-C	133	Red
Rio Rojo	2	2021	Springlake	4	21SPR-C	133	Red
Rio Rojo	1	2021	Springlake	18	21SPR-R	98	Red
Rio Rojo	2	2021	Springlake	18	21SPR-R	91	Red
Russet Burbank	1	2019	Dalhart	18	19DAL-R	112	Russet
Russet Burbank	2	2019	Dalhart	18	19DAL-R	119	Russet
Russet Burbank	1	2020	Dalhart	4	20DAL-C		Russet
Russet Burbank	2	2020	Dalhart	4	20DAL-C	193	Russet

Russet Burbank	1	2020	Dalhart	18	20DAL-R	123	Russet
Russet Burbank	2	2020	Dalhart	18	20DAL-R	123	Russet
Russet Burbank	1	2022	Dalhart	4	22DAL-C	171	Russet
Russet Burbank	2	2022	Dalhart	4	22DAL-C	171	Russet
Russet Burbank	1	2022	Dalhart	18	22DAL-R	154	Russet
Russet Burbank	2	2022	Dalhart	18	22DAL-R	136	Russet
Russet Burbank	1	2020	Springlake	4	20SPR-C	151	Russet
Russet Burbank	2	2020	Springlake	4	20SPR-C	151	Russet
Russet Burbank	1	2020	Springlake	18	20SPR-R	95	Russet
Russet Burbank	2	2020	Springlake	18	20SPR-R	81	Russet
Russet Burbank	1	2021	Springlake	4	21SPR-C	156	Russet
Russet Burbank	2	2021	Springlake	4	21SPR-C	156	Russet
Russet Burbank	1	2021	Springlake	18	21SPR-R	133	Russet
Russet Burbank	2	2021	Springlake	18	21SPR-R	119	Russet
Russet Burbank	1	2022	Springlake	4	22SPR-C	99	Russet
Russet Burbank	2	2022	Springlake	4	22SPR-C	99	Russet
Russet Burbank	1	2022	Springlake	18	22SPR-R	82	Russet
Russet Burbank	2	2022	Springlake	18	22SPR-R	68	Russet
Russet Norkotah	1	2019	Dalhart	18	19DAL-R	98	Russet
Russet Norkotah	2	2019	Dalhart	18	19DAL-R	98	Russet
Russet Norkotah	1	2020	Dalhart	4	20DAL-C	158	Russet
Russet Norkotah	2	2020	Dalhart	4	20DAL-C	151	Russet
Russet Norkotah	1	2020	Dalhart	18	20DAL-R	109	Russet
Russet Norkotah	2	2020	Dalhart	18	20DAL-R	95	Russet
Russet Norkotah	1	2022	Dalhart	4	22DAL-C	143	Russet
Russet Norkotah	2	2022	Dalhart	4	22DAL-C	122	Russet
Russet Norkotah	1	2022	Dalhart	18	22DAL-R	98	Russet
Russet Norkotah	2	2022	Dalhart	18	22DAL-R	98	Russet
Russet Norkotah	1	2020	Springlake	4	20SPR-C	95	Russet
Russet Norkotah	2	2020	Springlake	4	20SPR-C	95	Russet
Russet Norkotah	1	2020	Springlake	18	20SPR-R	74	Russet
Russet Norkotah	2	2020	Springlake	18	20SPR-R	74	Russet
Russet Norkotah	1	2021	Springlake	4	21SPR-C		Russet
Russet Norkotah	2	2021	Springlake	4	21SPR-C	94	Russet
Russet Norkotah	1	2021	Springlake	18	21SPR-R	112	Russet
Russet Norkotah	2	2021	Springlake	18	21SPR-R	84	Russet
Russet Norkotah	1	2022	Springlake	4	22SPR-C	71	Russet
Russet Norkotah	2	2022	Springlake	4	22SPR-C	99	Russet
Russet Norkotah	1	2022	Springlake	18	22SPR-R	68	Russet
Russet Norkotah	2	2022	Springlake	18	22SPR-R	75	Russet
Russet Norkotah 102	1	2019	Dalhart	18	19DAL-R	112	Russet
Russet Norkotah 102	2	2019	Dalhart	18	19DAL-R	112	Russet
Russet Norkotah 102	1	2020	Dalhart	4	20DAL-C	151	Russet
Russet Norkotah 102	2	2020	Dalhart	4	20DAL-C	144	Russet
Russet Norkotah 102	1	2020	Dalhart	18	20DAL-R	109	Russet
Russet Norkotah 102	2	2020	Dalhart	18	20DAL-R	109	Russet
Russet Norkotah 102	1	2020	Springlake	4	20SPR-C	109	Russet

Russet Norkotah 102	2	2020	Springlake	4	20SPR-C	102	Russet
Russet Norkotah 102	1	2020	Springlake	18	20SPR-R	81	Russet
Russet Norkotah 102	2	2020	Springlake	18	20SPR-R	89	Russet
Russet Norkotah 102	1	2021	Springlake	4	21SPR-C	107	Russet
Russet Norkotah 102	2	2021	Springlake	4	21SPR-C	114	Russet
Russet Norkotah 102	1	2021	Springlake	18	21SPR-R	98	Russet
Russet Norkotah 102	2	2021	Springlake	18	21SPR-R	84	Russet
Russet Norkotah 112	1	2019	Dalhart	18	19DAL-R	112	Russet
Russet Norkotah 112	2	2019	Dalhart	18	19DAL-R	105	Russet
Russet Norkotah 112	1	2020	Dalhart	4	20DAL-C	158	Russet
Russet Norkotah 112	2	2020	Dalhart	4	20DAL-C	144	Russet
Russet Norkotah 112	1	2020	Dalhart	18	20DAL-R	102	Russet
Russet Norkotah 112	2	2020	Dalhart	18	20DAL-R	109	Russet
Russet Norkotah 112	1	2020	Springlake	4	20SPR-C	109	Russet
Russet Norkotah 112	2	2020	Springlake	4	20SPR-C	95	Russet
Russet Norkotah 112	1	2020	Springlake	18	20SPR-R	81	Russet
Russet Norkotah 112	2	2020	Springlake	18	20SPR-R	95	Russet
Russet Norkotah 112	1	2021	Springlake	4	21SPR-C	121	Russet
Russet Norkotah 112	2	2021	Springlake	4	21SPR-C	128	Russet
Russet Norkotah 112	1	2021	Springlake	18	21SPR-R	98	Russet
Russet Norkotah 112	2	2021	Springlake	18	21SPR-R	119	Russet
Russet Norkotah 223	1	2019	Dalhart	18	19DAL-R	98	Russet
Russet Norkotah 223	2	2019	Dalhart	18	19DAL-R	112	Russet
Russet Norkotah 223	1	2020	Dalhart	4	20DAL-C		Russet
Russet Norkotah 223	2	2020	Dalhart	4	20DAL-C	165	Russet
Russet Norkotah 223	1	2020	Dalhart	18	20DAL-R		Russet
Russet Norkotah 223	2	2020	Dalhart	18	20DAL-R	117	Russet
Russet Norkotah 223	1	2020	Springlake	4	20SPR-C		Russet
Russet Norkotah 223	2	2020	Springlake	4	20SPR-C	109	Russet
Russet Norkotah 223	1	2020	Springlake	18	20SPR-R		Russet
Russet Norkotah 223	2	2020	Springlake	18	20SPR-R	81	Russet
Russet Norkotah 223	1	2021	Springlake	4	21SPR-C		Russet
Russet Norkotah 223	2	2021	Springlake	4	21SPR-C	121	Russet
Russet Norkotah 223	1	2021	Springlake	18	21SPR-R	98	Russet
Russet Norkotah 223	2	2021	Springlake	18	21SPR-R	119	Russet
Russet Norkotah 278	1	2019	Dalhart	18	19DAL-R	105	Russet
Russet Norkotah 278	2	2019	Dalhart	18	19DAL-R	112	Russet
Russet Norkotah 278	1	2020	Dalhart	4	20DAL-C	140	Russet
Russet Norkotah 278	2	2020	Dalhart	4	20DAL-C	140	Russet
Russet Norkotah 278	1	2020	Dalhart	18	20DAL-R	117	Russet
Russet Norkotah 278	2	2020	Dalhart	18	20DAL-R	102	Russet
Russet Norkotah 278	1	2020	Springlake	4	20SPR-C	95	Russet
Russet Norkotah 278	2	2020	Springlake	4	20SPR-C	116	Russet
Russet Norkotah 278	1	2020	Springlake	18	20SPR-R	81	Russet
Russet Norkotah 278	2	2020	Springlake	18	20SPR-R	81	Russet
Russet Norkotah 278	1	2021	Springlake	4	21SPR-C	114	Russet
Russet Norkotah 278	2	2021	Springlake	4	21SPR-C	114	Russet

Russet Norkotah 278	1	2021	Springlake	18	21SPR-R	91	Russet
Russet Norkotah 278	2	2021	Springlake	18	21SPR-R	105	Russet
Russet Norkotah 296	1	2019	Dalhart	18	19DAL-R	112	Russet
Russet Norkotah 296	2	2019	Dalhart	18	19DAL-R	112	Russet
Russet Norkotah 296	1	2020	Dalhart	4	20DAL-C	151	Russet
Russet Norkotah 296	2	2020	Dalhart	4	20DAL-C	144	Russet
Russet Norkotah 296	1	2020	Dalhart	18	20DAL-R	109	Russet
Russet Norkotah 296	2	2020	Dalhart	18	20DAL-R	109	Russet
Russet Norkotah 296	1	2020	Springlake	4	20SPR-C	109	Russet
Russet Norkotah 296	2	2020	Springlake	4	20SPR-C	116	Russet
Russet Norkotah 296	1	2020	Springlake	18	20SPR-R	81	Russet
Russet Norkotah 296	2	2020	Springlake	18	20SPR-R	81	Russet
Russet Norkotah 296	1	2021	Springlake	4	21SPR-C	114	Russet
Russet Norkotah 296	2	2021	Springlake	4	21SPR-C	107	Russet
Russet Norkotah 296	1	2021	Springlake	18	21SPR-R	91	Russet
Russet Norkotah 296	2	2021	Springlake	18	21SPR-R	105	Russet
Shepody	1	2022	Dalhart	4	22DAL-C	122	Russet
Shepody	2	2022	Dalhart	4	22DAL-C	108	Russet
Shepody	1	2022	Dalhart	18	22DAL-R	105	Russet
Shepody	2	2022	Dalhart	18	22DAL-R	91	Russet
Shepody	1	2022	Springlake	4	22SPR-C	99	Russet
Shepody	2	2022	Springlake	4	22SPR-C	99	Russet
Shepody	1	2022	Springlake	18	22SPR-R	61	Russet
Shepody	2	2022	Springlake	18	22SPR-R	54	Russet
Sierra GoldTM	1	2019	Dalhart	18	19DAL-R	56	Russet
Sierra GoldTM	2	2019	Dalhart	18	19DAL-R	56	Russet
Sierra GoldTM	1	2020	Dalhart	4	20DAL-C	158	Russet
Sierra GoldTM	2	2020	Dalhart	4	20DAL-C	133	Russet
Sierra GoldTM	1	2020	Dalhart	18	20DAL-R	74	Russet
Sierra GoldTM	2	2020	Dalhart	18	20DAL-R	81	Russet
Sierra GoldTM	1	2022	Dalhart	4	22DAL-C	122	Russet
Sierra GoldTM	2	2022	Dalhart	4	22DAL-C	122	Russet
Sierra GoldTM	1	2022	Dalhart	18	22DAL-R	56	Russet
Sierra GoldTM	2	2022	Dalhart	18	22DAL-R	63	Russet
Sierra GoldTM	1	2020	Springlake	4	20SPR-C	109	Russet
Sierra GoldTM	2	2020	Springlake	4	20SPR-C	81	Russet
Sierra GoldTM	1	2020	Springlake	18	20SPR-R	46	Russet
Sierra GoldTM	2	2020	Springlake	18	20SPR-R	53	Russet
Sierra GoldTM	1	2021	Springlake	4	21SPR-C	114	Russet
Sierra GoldTM	2	2021	Springlake	4	21SPR-C	114	Russet
Sierra GoldTM	1	2021	Springlake	18	21SPR-R	63	Russet
Sierra GoldTM	2	2021	Springlake	18	21SPR-R	63	Russet
Sierra GoldTM	1	2022	Springlake	4	22SPR-C	102	Russet
Sierra GoldTM	2	2022	Springlake	4	22SPR-C	102	Russet
Sierra GoldTM	1	2022	Springlake	18	22SPR-R	40	Russet
Sierra GoldTM	2	2022	Springlake	18	22SPR-R	34	Russet
Sierra RoseTM	1	2019	Dalhart	18	19DAL-R	63	Red

Sierra RoseTM	2	2019	Dalhart	18	19DAL-R	63	Red
Sierra RoseTM	1	2020	Dalhart	4	20DAL-C		Red
Sierra RoseTM	2	2020	Dalhart	4	20DAL-C	144	Red
Sierra RoseTM	1	2020	Dalhart	18	20DAL-R	53	Red
Sierra RoseTM	2	2020	Dalhart	18	20DAL-R	67	Red
Sierra RoseTM	1	2020	Springlake	4	20SPR-C	108	Red
Sierra RoseTM	2	2020	Springlake	4	20SPR-C	108	Red
Sierra RoseTM	1	2020	Springlake	18	20SPR-R	45	Red
Sierra RoseTM	2	2020	Springlake	18	20SPR-R	66	Red
Sierra RoseTM	1	2021	Springlake	4	21SPR-C	126	Red
Sierra RoseTM	2	2021	Springlake	4	21SPR-C	133	Red
Sierra RoseTM	1	2021	Springlake	18	21SPR-R	63	Red
Sierra RoseTM	2	2021	Springlake	18	21SPR-R	63	Red
Snowden	1	2022	Dalhart	4	22DAL-C	116	Chipper
Snowden	2	2022	Dalhart	4	22DAL-C	137	Chipper
Snowden	1	2022	Dalhart	18	22DAL-R	78	Chipper
Snowden	2	2022	Dalhart	18	22DAL-R	78	Chipper
Snowden	1	2022	Springlake	4	22SPR-C	102	Chipper
Snowden	2	2022	Springlake	4	22SPR-C	102	Chipper
Snowden	1	2022	Springlake	18	22SPR-R	50	Chipper
Snowden	2	2022	Springlake	18	22SPR-R	50	Chipper
Stampede Russet	1	2019	Dalhart	18	19DAL-R	91	Russet
Stampede Russet	2	2019	Dalhart	18	19DAL-R	77	Russet
Stampede Russet	1	2020	Dalhart	4	20DAL-C	151	Russet
Stampede Russet	2	2020	Dalhart	4	20DAL-C	140	Russet
Stampede Russet	1	2020	Dalhart	18	20DAL-R	81	Russet
Stampede Russet	2	2020	Dalhart	18	20DAL-R	81	Russet
Stampede Russet	1	2020	Springlake	4	20SPR-C	116	Russet
Stampede Russet	2	2020	Springlake	4	20SPR-C	123	Russet
Stampede Russet	1	2020	Springlake	18	20SPR-R	74	Russet
Stampede Russet	2	2020	Springlake	18	20SPR-R	67	Russet
Stampede Russet	1	2021	Springlake	4	21SPR-C	142	Russet
Stampede Russet	2	2021	Springlake	4	21SPR-C	128	Russet
Stampede Russet	1	2021	Springlake	18	21SPR-R		Russet
Stampede Russet	2	2021	Springlake	18	21SPR-R	77	Russet
Tacna	1	2019	Dalhart	18	19DAL-R	56	Yellow
Tacna	2	2019	Dalhart	18	19DAL-R		Yellow
Tacna	1	2020	Dalhart	4	20DAL-C		Yellow
Tacna	2	2020	Dalhart	4	20DAL-C	133	Yellow
Tacna	1	2020	Dalhart	18	20DAL-R	74	Yellow
Tacna	2	2020	Dalhart	18	20DAL-R	81	Yellow
Tacna	1	2020	Springlake	4	20SPR-C		Yellow
Tacna	2	2020	Springlake	4	20SPR-C		Yellow
Tacna	1	2020	Springlake	18	20SPR-R		Yellow
Tacna	2	2020	Springlake	18	20SPR-R		Yellow
Tacna	1	2021	Springlake	4	21SPR-C	126	Yellow
Tacna	2	2021	Springlake	4	21SPR-C	112	Yellow

Tacna	1	2021	Springlake	18	21SPR-R	70	Yellow
Tacna	2	2021	Springlake	18	21SPR-R	84	Yellow
Tokio	1	2019	Dalhart	18	19DAL-R	63	Yellow
Tokio	2	2019	Dalhart	18	19DAL-R	63	Yellow
Tokio	1	2020	Dalhart	4	20DAL-C		Yellow
Tokio	2	2020	Dalhart	4	20DAL-C	88	Yellow
Tokio	1	2020	Dalhart	18	20DAL-R	67	Yellow
Tokio	2	2020	Dalhart	18	20DAL-R	67	Yellow
Tokio	1	2020	Springlake	4	20SPR-C	101	Yellow
Tokio	2	2020	Springlake	4	20SPR-C	94	Yellow
Tokio	1	2020	Springlake	18	20SPR-R	52	Yellow
Tokio	2	2020	Springlake	18	20SPR-R	38	Yellow
Tokio	1	2021	Springlake	4	21SPR-C	112	Yellow
Tokio	2	2021	Springlake	4	21SPR-C		Yellow
Tokio	1	2021	Springlake	18	21SPR-R	63	Yellow
Tokio	2	2021	Springlake	18	21SPR-R		Yellow
TX03196-1W	1	2019	Dalhart	18	19DAL-R		Chipper
TX03196-1W	2	2019	Dalhart	18	19DAL-R	70	Chipper
TX03196-1W	1	2020	Dalhart	4	20DAL-C	172	Chipper
TX03196-1W	2	2020	Dalhart	4	20DAL-C	151	Chipper
TX03196-1W	1	2020	Dalhart	18	20DAL-R	95	Chipper
TX03196-1W	2	2020	Dalhart	18	20DAL-R	95	Chipper
TX03196-1W	1	2020	Springlake	4	20SPR-C	157	Chipper
TX03196-1W	2	2020	Springlake	4	20SPR-C	157	Chipper
TX03196-1W	1	2020	Springlake	18	20SPR-R	80	Chipper
TX03196-1W	2	2020	Springlake	18	20SPR-R	87	Chipper
TX03196-1W	1	2021	Springlake	4	21SPR-C		Chipper
TX03196-1W	2	2021	Springlake	4	21SPR-C	161	Chipper
TX03196-1W	1	2021	Springlake	18	21SPR-R	105	Chipper
TX03196-1W	2	2021	Springlake	18	21SPR-R	84	Chipper
TX05249-10W	1	2019	Dalhart	18	19DAL-R	91	Russet
TX05249-10W	2	2019	Dalhart	18	19DAL-R	112	Russet
TX05249-10W	1	2020	Dalhart	4	20DAL-C		Russet
TX05249-10W	2	2020	Dalhart	4	20DAL-C	207	Russet
TX05249-10W	1	2020	Dalhart	18	20DAL-R	102	Russet
TX05249-10W	2	2020	Dalhart	18	20DAL-R	102	Russet
TX05249-10W	1	2020	Springlake	4	20SPR-C	150	Russet
TX05249-10W	2	2020	Springlake	4	20SPR-C		Russet
TX05249-10W	1	2020	Springlake	18	20SPR-R	80	Russet
TX05249-10W	2	2020	Springlake	18	20SPR-R	52	Russet
TX05249-10W	1	2021	Springlake	4	21SPR-C	147	Russet
TX05249-10W	2	2021	Springlake	4	21SPR-C	140	Russet
TX05249-10W	1	2021	Springlake	18	21SPR-R	105	Russet
TX05249-10W	2	2021	Springlake	18	21SPR-R	98	Russet
TX05249-11W	1	2019	Dalhart	18	19DAL-R	98	Chipper
TX05249-11W	2	2019	Dalhart	18	19DAL-R	119	Chipper
TX05249-11W	1	2020	Dalhart	4	20DAL-C	193	Chipper

TX05249-11W	2	2020	Dalhart	4	20DAL-C	193	Chipper
TX05249-11W	1	2020	Dalhart	18	20DAL-R	123	Chipper
TX05249-11W	2	2020	Dalhart	18	20DAL-R	123	Chipper
TX05249-11W	1	2020	Springlake	4	20SPR-C		Chipper
TX05249-11W	2	2020	Springlake	4	20SPR-C	150	Chipper
TX05249-11W	1	2020	Springlake	18	20SPR-R	95	Chipper
TX05249-11W	2	2020	Springlake	18	20SPR-R	115	Chipper
TX05249-11W	1	2021	Springlake	4	21SPR-C	168	Chipper
TX05249-11W	2	2021	Springlake	4	21SPR-C	168	Chipper
TX05249-11W	1	2021	Springlake	18	21SPR-R		Chipper
TX05249-11W	2	2021	Springlake	18	21SPR-R	126	Chipper
TX05249-3W	1	2019	Dalhart	18	19DAL-R	63	Chipper
TX05249-3W	2	2019	Dalhart	18	19DAL-R	70	Chipper
TX05249-3W	1	2020	Dalhart	4	20DAL-C		Chipper
TX05249-3W	2	2020	Dalhart	4	20DAL-C	144	Chipper
TX05249-3W	1	2020	Dalhart	18	20DAL-R	81	Chipper
TX05249-3W	2	2020	Dalhart	18	20DAL-R	87	Chipper
TX05249-3W	1	2020	Springlake	4	20SPR-C		Chipper
TX05249-3W	2	2020	Springlake	4	20SPR-C	136	Chipper
TX05249-3W	1	2020	Springlake	18	20SPR-R	80	Chipper
TX05249-3W	2	2020	Springlake	18	20SPR-R	80	Chipper
TX05249-3W	1	2021	Springlake	4	21SPR-C		Chipper
TX05249-3W	2	2021	Springlake	4	21SPR-C	154	Chipper
TX05249-3W	1	2021	Springlake	18	21SPR-R	84	Chipper
TX05249-3W	2	2021	Springlake	18	21SPR-R	98	Chipper
TX08350-12Ru	1	2019	Dalhart	18	19DAL-R	91	Russet
TX08350-12Ru	2	2019	Dalhart	18	19DAL-R	91	Russet
TX08350-12Ru	1	2020	Dalhart	4	20DAL-C	133	Russet
TX08350-12Ru	2	2020	Dalhart	4	20DAL-C		Russet
TX08350-12Ru	1	2020	Dalhart	18	20DAL-R	102	Russet
TX08350-12Ru	2	2020	Dalhart	18	20DAL-R	87	Russet
TX08350-12Ru	1	2020	Springlake	4	20SPR-C	95	Russet
TX08350-12Ru	2	2020	Springlake	4	20SPR-C	88	Russet
TX08350-12Ru	1	2020	Springlake	18	20SPR-R	89	Russet
TX08350-12Ru	2	2020	Springlake	18	20SPR-R	81	Russet
TX08350-12Ru	1	2021	Springlake	4	21SPR-C	107	Russet
TX08350-12Ru	2	2021	Springlake	4	21SPR-C	107	Russet
TX08350-12Ru	1	2021	Springlake	18	21SPR-R	91	Russet
TX08350-12Ru	2	2021	Springlake	18	21SPR-R	84	Russet
TX08385-2P/YP	1	2019	Dalhart	18	19DAL-R	70	Purple
TX08385-2P/YP	2	2019	Dalhart	18	19DAL-R	70	Purple
TX08385-2P/YP	1	2020	Dalhart	4	20DAL-C		Purple
TX08385-2P/YP	2	2020	Dalhart	4	20DAL-C	133	Purple
TX08385-2P/YP	1	2020	Dalhart	18	20DAL-R	67	Purple
TX08385-2P/YP	2	2020	Dalhart	18	20DAL-R	81	Purple
TX08385-2P/YP	1	2020	Springlake	4	20SPR-C	136	Purple
TX08385-2P/YP	2	2020	Springlake	4	20SPR-C	122	Purple

TX08385-2P/YP	1	2020	Springlake	18	20SPR-R	73	Purple
TX08385-2P/YP	2	2020	Springlake	18	20SPR-R	66	Purple
TX08385-2P/YP	1	2021	Springlake	4	21SPR-C	126	Purple
TX08385-2P/YP	2	2021	Springlake	4	21SPR-C	133	Purple
TX08385-2P/YP	1	2021	Springlake	18	21SPR-R	70	Purple
TX08385-2P/YP	2	2021	Springlake	18	21SPR-R	84	Purple
TX09396-1W	1	2019	Dalhart	18	19DAL-R	63	Chipper
TX09396-1W	2	2019	Dalhart	18	19DAL-R	77	Chipper
TX09396-1W	1	2020	Dalhart	4	20DAL-C	140	Chipper
TX09396-1W	2	2020	Dalhart	4	20DAL-C	151	Chipper
TX09396-1W	1	2020	Dalhart	18	20DAL-R	81	Chipper
TX09396-1W	2	2020	Dalhart	18	20DAL-R	81	Chipper
TX09396-1W	1	2020	Springlake	4	20SPR-C	150	Chipper
TX09396-1W	2	2020	Springlake	4	20SPR-C		Chipper
TX09396-1W	1	2020	Springlake	18	20SPR-R	73	Chipper
TX09396-1W	2	2020	Springlake	18	20SPR-R	80	Chipper
TX09396-1W	1	2021	Springlake	4	21SPR-C	168	Chipper
TX09396-1W	2	2021	Springlake	4	21SPR-C	174	Chipper
TX09396-1W	1	2021	Springlake	18	21SPR-R	98	Chipper
TX09396-1W	2	2021	Springlake	18	21SPR-R	105	Chipper
TX09403-15W	1	2019	Dalhart	18	19DAL-R	63	Chipper
TX09403-15W	2	2019	Dalhart	18	19DAL-R	56	Chipper
TX09403-15W	1	2020	Dalhart	4	20DAL-C	133	Chipper
TX09403-15W	2	2020	Dalhart	4	20DAL-C		Chipper
TX09403-15W	1	2020	Dalhart	18	20DAL-R	67	Chipper
TX09403-15W	2	2020	Dalhart	18	20DAL-R	67	Chipper
TX09403-15W	1	2020	Springlake	4	20SPR-C	122	Chipper
TX09403-15W	2	2020	Springlake	4	20SPR-C	115	Chipper
TX09403-15W	1	2020	Springlake	18	20SPR-R	66	Chipper
TX09403-15W	2	2020	Springlake	18	20SPR-R	59	Chipper
TX09403-15W	1	2021	Springlake	4	21SPR-C		Chipper
TX09403-15W	2	2021	Springlake	4	21SPR-C		Chipper
TX09403-15W	1	2021	Springlake	18	21SPR-R	56	Chipper
TX09403-15W	2	2021	Springlake	18	21SPR-R	63	Chipper
TX09403-21W	1	2019	Dalhart	18	19DAL-R	56	Chipper
TX09403-21W	2	2019	Dalhart	18	19DAL-R	63	Chipper
TX09403-21W	1	2020	Dalhart	4	20DAL-C		Chipper
TX09403-21W	2	2020	Dalhart	4	20DAL-C		Chipper
TX09403-21W	1	2020	Dalhart	18	20DAL-R	60	Chipper
TX09403-21W	2	2020	Dalhart	18	20DAL-R	53	Chipper
TX09403-21W	1	2020	Springlake	4	20SPR-C	122	Chipper
TX09403-21W	2	2020	Springlake	4	20SPR-C	108	Chipper
TX09403-21W	1	2020	Springlake	18	20SPR-R	66	Chipper
TX09403-21W	2	2020	Springlake	18	20SPR-R	66	Chipper
TX09403-21W	1	2021	Springlake	4	21SPR-C		Chipper
TX09403-21W	2	2021	Springlake	4	21SPR-C	119	Chipper
TX09403-21W	1	2021	Springlake	18	21SPR-R	70	Chipper

TX09403-21W	2	2021	Springlake	18	21SPR-R	56	Chipper
TX09414-1W	1	2019	Dalhart	18	19DAL-R	84	Chipper
TX09414-1W	2	2019	Dalhart	18	19DAL-R	77	Chipper
TX09414-1W	1	2020	Dalhart	4	20DAL-C		Chipper
TX09414-1W	2	2020	Dalhart	4	20DAL-C	133	Chipper
TX09414-1W	1	2020	Dalhart	18	20DAL-R	81	Chipper
TX09414-1W	2	2020	Dalhart	18	20DAL-R	87	Chipper
TX09414-1W	1	2020	Springlake	4	20SPR-C	122	Chipper
TX09414-1W	2	2020	Springlake	4	20SPR-C	94	Chipper
TX09414-1W	1	2020	Springlake	18	20SPR-R	66	Chipper
TX09414-1W	2	2020	Springlake	18	20SPR-R	80	Chipper
TX09414-1W	1	2021	Springlake	4	21SPR-C	133	Chipper
TX09414-1W	2	2021	Springlake	4	21SPR-C	112	Chipper
TX09414-1W	1	2021	Springlake	18	21SPR-R	105	Chipper
TX09414-1W	2	2021	Springlake	18	21SPR-R	77	Chipper
TX10437-9Pyspl/Y	1	2019	Dalhart	18	19DAL-R	91	Yellow
TX10437-9Pyspl/Y	2	2019	Dalhart	18	19DAL-R		Yellow
TX10437-9Pyspl/Y	1	2020	Dalhart	4	20DAL-C	151	Yellow
TX10437-9Pyspl/Y	2	2020	Dalhart	4	20DAL-C	151	Yellow
TX10437-9Pyspl/Y	1	2020	Dalhart	18	20DAL-R	81	Yellow
TX10437-9Pyspl/Y	2	2020	Dalhart	18	20DAL-R	95	Yellow
TX10437-9Pyspl/Y	1	2020	Springlake	4	20SPR-C		Yellow
TX10437-9Pyspl/Y	2	2020	Springlake	4	20SPR-C		Yellow
TX10437-9Pyspl/Y	1	2020	Springlake	18	20SPR-R		Yellow
TX10437-9Pyspl/Y	2	2020	Springlake	18	20SPR-R		Yellow
TX10437-9Pyspl/Y	1	2021	Springlake	4	21SPR-C	133	Yellow
TX10437-9Pyspl/Y	2	2021	Springlake	4	21SPR-C	133	Yellow
TX10437-9Pyspl/Y	1	2021	Springlake	18	21SPR-R	98	Yellow
TX10437-9Pyspl/Y	2	2021	Springlake	18	21SPR-R	119	Yellow
TX11454-9Ru/Y	1	2019	Dalhart	18	19DAL-R	91	Russet
TX11454-9Ru/Y	2	2019	Dalhart	18	19DAL-R	91	Russet
TX11454-9Ru/Y	1	2020	Dalhart	4	20DAL-C	140	Russet
TX11454-9Ru/Y	2	2020	Dalhart	4	20DAL-C	151	Russet
TX11454-9Ru/Y	1	2020	Dalhart	18	20DAL-R	102	Russet
TX11454-9Ru/Y	2	2020	Dalhart	18	20DAL-R	95	Russet
TX11454-9Ru/Y	1	2020	Springlake	4	20SPR-C	116	Russet
TX11454-9Ru/Y	2	2020	Springlake	4	20SPR-C	88	Russet
TX11454-9Ru/Y	1	2020	Springlake	18	20SPR-R	67	Russet
TX11454-9Ru/Y	2	2020	Springlake	18	20SPR-R	67	Russet
TX11454-9Ru/Y	1	2021	Springlake	4	21SPR-C		Russet
TX11454-9Ru/Y	2	2021	Springlake	4	21SPR-C		Russet
TX11454-9Ru/Y	1	2021	Springlake	18	21SPR-R		Russet
TX11454-9Ru/Y	2	2021	Springlake	18	21SPR-R		Russet
TX11461-2W	1	2019	Dalhart	18	19DAL-R	77	Chipper
TX11461-2W	2	2019	Dalhart	18	19DAL-R	70	Chipper
TX11461-2W	1	2020	Dalhart	4	20DAL-C	133	Chipper
TX11461-2W	2	2020	Dalhart	4	20DAL-C	133	Chipper

TX11461-2W	1	2020	Dalhart	18	20DAL-R	87	Chipper
TX11461-2W	2	2020	Dalhart	18	20DAL-R	81	Chipper
TX11461-2W	1	2020	Springlake	4	20SPR-C	108	Chipper
TX11461-2W	2	2020	Springlake	4	20SPR-C	122	Chipper
TX11461-2W	1	2020	Springlake	18	20SPR-R	66	Chipper
TX11461-2W	2	2020	Springlake	18	20SPR-R	66	Chipper
TX11461-2W	1	2021	Springlake	4	21SPR-C	119	Chipper
TX11461-2W	2	2021	Springlake	4	21SPR-C	119	Chipper
TX11461-2W	1	2021	Springlake	18	21SPR-R	70	Chipper
TX11461-2W	2	2021	Springlake	18	21SPR-R	84	Chipper
TX11461-3W	1	2019	Dalhart	18	19DAL-R	70	Chipper
TX11461-3W	2	2019	Dalhart	18	19DAL-R	56	Chipper
TX11461-3W	1	2020	Dalhart	4	20DAL-C		Chipper
TX11461-3W	2	2020	Dalhart	4	20DAL-C		Chipper
TX11461-3W	1	2020	Dalhart	18	20DAL-R	81	Chipper
TX11461-3W	2	2020	Dalhart	18	20DAL-R	81	Chipper
TX11461-3W	1	2020	Springlake	4	20SPR-C	115	Chipper
TX11461-3W	2	2020	Springlake	4	20SPR-C		Chipper
TX11461-3W	1	2020	Springlake	18	20SPR-R	66	Chipper
TX11461-3W	2	2020	Springlake	18	20SPR-R	66	Chipper
TX11461-3W	1	2021	Springlake	4	21SPR-C	106	Chipper
TX11461-3W	2	2021	Springlake	4	21SPR-C	112	Chipper
TX11461-3W	1	2021	Springlake	18	21SPR-R	63	Chipper
TX11461-3W	2	2021	Springlake	18	21SPR-R	63	Chipper
TX12474-1P/R	1	2019	Dalhart	18	19DAL-R	112	Purple
TX12474-1P/R	2	2019	Dalhart	18	19DAL-R	119	Purple
TX12474-1P/R	1	2020	Dalhart	4	20DAL-C		Purple
TX12474-1P/R	2	2020	Dalhart	4	20DAL-C	88	Purple
TX12474-1P/R	1	2020	Dalhart	18	20DAL-R	109	Purple
TX12474-1P/R	2	2020	Dalhart	18	20DAL-R	81	Purple
TX12474-1P/R	1	2020	Springlake	4	20SPR-C	171	Purple
TX12474-1P/R	2	2020	Springlake	4	20SPR-C	178	Purple
TX12474-1P/R	1	2020	Springlake	18	20SPR-R	108	Purple
TX12474-1P/R	2	2020	Springlake	18	20SPR-R	115	Purple
TX12474-1P/R	1	2021	Springlake	4	21SPR-C		Purple
TX12474-1P/R	2	2021	Springlake	4	21SPR-C		Purple
TX12474-1P/R	1	2021	Springlake	18	21SPR-R	168	Purple
TX12474-1P/R	2	2021	Springlake	18	21SPR-R	161	Purple
TX12484-2WZC	1	2022	Dalhart	4	22DAL-C	130	Chipper
TX12484-2WZC	2	2022	Dalhart	4	22DAL-C	130	Chipper
TX12484-2WZC	1	2022	Dalhart	18	22DAL-R	71	Chipper
TX12484-2WZC	2	2022	Dalhart	18	22DAL-R	78	Chipper
TX12484-2WZC	1	2022	Springlake	4	22SPR-C	95	Chipper
TX12484-2WZC	2	2022	Springlake	4	22SPR-C	93	Chipper
TX12484-2WZC	1	2022	Springlake	18	22SPR-R	43	Chipper
TX12484-2WZC	2	2022	Springlake	18	22SPR-R	41	Chipper
TX12484-3WZC	1	2022	Dalhart	4	22DAL-C	88	Chipper

TX12484-3WZC	2	2022	Dalhart	4	22DAL-C	116	Chipper
TX12484-3WZC	1	2022	Dalhart	18	22DAL-R	64	Chipper
TX12484-3WZC	2	2022	Dalhart	18	22DAL-R	71	Chipper
TX12484-3WZC	1	2022	Springlake	4	22SPR-C	88	Chipper
TX12484-3WZC	2	2022	Springlake	4	22SPR-C	86	Chipper
TX12484-3WZC	1	2022	Springlake	18	22SPR-R	37	Chipper
TX12484-3WZC	2	2022	Springlake	18	22SPR-R	34	Chipper
TX12484-4W	1	2019	Dalhart	18	19DAL-R	56	Chipper
TX12484-4W	2	2019	Dalhart	18	19DAL-R	56	Chipper
TX12484-4W	1	2020	Dalhart	4	20DAL-C		Chipper
TX12484-4W	2	2020	Dalhart	4	20DAL-C	88	Chipper
TX12484-4W	1	2020	Dalhart	18	20DAL-R	53	Chipper
TX12484-4W	2	2020	Dalhart	18	20DAL-R	53	Chipper
TX12484-4W	1	2020	Springlake	4	20SPR-C	101	Chipper
TX12484-4W	2	2020	Springlake	4	20SPR-C	115	Chipper
TX12484-4W	1	2020	Springlake	18	20SPR-R	45	Chipper
TX12484-4W	2	2020	Springlake	18	20SPR-R	38	Chipper
TX12484-4W	1	2021	Springlake	4	21SPR-C	112	Chipper
TX12484-4W	2	2021	Springlake	4	21SPR-C	112	Chipper
TX12484-4W	1	2021	Springlake	18	21SPR-R	56	Chipper
TX12484-4W	2	2021	Springlake	18	21SPR-R	56	Chipper
TX13590-9Ru	1	2019	Dalhart	18	19DAL-R	112	Russet
TX13590-9Ru	2	2019	Dalhart	18	19DAL-R	84	Russet
TX13590-9Ru	1	2020	Dalhart	4	20DAL-C	133	Russet
TX13590-9Ru	2	2020	Dalhart	4	20DAL-C	133	Russet
TX13590-9Ru	1	2020	Dalhart	18	20DAL-R	117	Russet
TX13590-9Ru	2	2020	Dalhart	18	20DAL-R	102	Russet
TX13590-9Ru	1	2020	Springlake	4	20SPR-C	95	Russet
TX13590-9Ru	2	2020	Springlake	4	20SPR-C	109	Russet
TX13590-9Ru	1	2020	Springlake	18	20SPR-R	95	Russet
TX13590-9Ru	2	2020	Springlake	18	20SPR-R	102	Russet
TX13590-9Ru	1	2021	Springlake	4	21SPR-C	107	Russet
TX13590-9Ru	2	2021	Springlake	4	21SPR-C	107	Russet
TX13590-9Ru	1	2021	Springlake	18	21SPR-R	119	Russet
TX13590-9Ru	2	2021	Springlake	18	21SPR-R	112	Russet
TX14611-1R	1	2019	Dalhart	18	19DAL-R	63	Red
TX14611-1R	2	2019	Dalhart	18	19DAL-R	77	Red
TX14611-1R	1	2020	Dalhart	4	20DAL-C		Red
TX14611-1R	2	2020	Dalhart	4	20DAL-C	140	Red
TX14611-1R	1	2020	Dalhart	18	20DAL-R	74	Red
TX14611-1R	2	2020	Dalhart	18	20DAL-R	95	Red
TX14611-1R	1	2020	Springlake	4	20SPR-C	150	Red
TX14611-1R	2	2020	Springlake	4	20SPR-C	108	Red
TX14611-1R	1	2020	Springlake	18	20SPR-R	73	Red
TX14611-1R	2	2020	Springlake	18	20SPR-R	66	Red
TX14611-1R	1	2021	Springlake	4	21SPR-C	126	Red
TX14611-1R	2	2021	Springlake	4	21SPR-C	140	Red

TX14611-1R	1	2021	Springlake	18	21SPR-R	84	Red
TX14611-1R	2	2021	Springlake	18	21SPR-R	84	Red
TX1475-3W	1	2019	Dalhart	18	19DAL-R	91	Chipper
TX1475-3W	2	2019	Dalhart	18	19DAL-R	91	Chipper
TX1475-3W	1	2020	Dalhart	4	20DAL-C	165	Chipper
TX1475-3W	2	2020	Dalhart	4	20DAL-C	133	Chipper
TX1475-3W	1	2020	Dalhart	18	20DAL-R	102	Chipper
TX1475-3W	2	2020	Dalhart	18	20DAL-R	109	Chipper
TX1475-3W	1	2020	Springlake	4	20SPR-C	122	Chipper
TX1475-3W	2	2020	Springlake	4	20SPR-C	129	Chipper
TX1475-3W	1	2020	Springlake	18	20SPR-R	87	Chipper
TX1475-3W	2	2020	Springlake	18	20SPR-R	80	Chipper
TX1475-3W	1	2021	Springlake	4	21SPR-C	168	Chipper
TX1475-3W	2	2021	Springlake	4	21SPR-C	140	Chipper
TX1475-3W	1	2021	Springlake	18	21SPR-R	98	Chipper
TX1475-3W	2	2021	Springlake	18	21SPR-R	91	Chipper
TX1617-1W/Y	1	2019	Dalhart	18	19DAL-R	63	Yellow
TX1617-1W/Y	2	2019	Dalhart	18	19DAL-R		Yellow
TX1617-1W/Y	1	2020	Dalhart	4	20DAL-C		Yellow
TX1617-1W/Y	2	2020	Dalhart	4	20DAL-C	133	Yellow
TX1617-1W/Y	1	2020	Dalhart	18	20DAL-R	81	Yellow
TX1617-1W/Y	2	2020	Dalhart	18	20DAL-R	87	Yellow
TX1617-1W/Y	1	2020	Springlake	4	20SPR-C	101	Yellow
TX1617-1W/Y	2	2020	Springlake	4	20SPR-C	143	Yellow
TX1617-1W/Y	1	2020	Springlake	18	20SPR-R	59	Yellow
TX1617-1W/Y	2	2020	Springlake	18	20SPR-R	52	Yellow
TX1617-1W/Y	1	2021	Springlake	4	21SPR-C	119	Yellow
TX1617-1W/Y	2	2021	Springlake	4	21SPR-C	133	Yellow
TX1617-1W/Y	1	2021	Springlake	18	21SPR-R	63	Yellow
TX1617-1W/Y	2	2021	Springlake	18	21SPR-R	91	Yellow
TX1673-1W/Y	1	2019	Dalhart	18	19DAL-R	70	Yellow
TX1673-1W/Y	2	2019	Dalhart	18	19DAL-R	70	Yellow
TX1673-1W/Y	1	2020	Dalhart	4	20DAL-C	144	Yellow
TX1673-1W/Y	2	2020	Dalhart	4	20DAL-C	144	Yellow
TX1673-1W/Y	1	2020	Dalhart	18	20DAL-R	117	Yellow
TX1673-1W/Y	2	2020	Dalhart	18	20DAL-R	95	Yellow
TX1673-1W/Y	1	2020	Springlake	4	20SPR-C	122	Yellow
TX1673-1W/Y	2	2020	Springlake	4	20SPR-C	115	Yellow
TX1673-1W/Y	1	2020	Springlake	18	20SPR-R	59	Yellow
TX1673-1W/Y	2	2020	Springlake	18	20SPR-R	38	Yellow
TX1673-1W/Y	1	2021	Springlake	4	21SPR-C	112	Yellow
TX1673-1W/Y	2	2021	Springlake	4	21SPR-C	112	Yellow
TX1673-1W/Y	1	2021	Springlake	18	21SPR-R	70	Yellow
TX1673-1W/Y	2	2021	Springlake	18	21SPR-R	77	Yellow
TX17734-1Y/Y	1	2022	Dalhart	4	22DAL-C	95	Yellow
TX17734-1Y/Y	2	2022	Dalhart	4	22DAL-C	95	Yellow
TX17734-1Y/Y	1	2022	Dalhart	18	22DAL-R	85	Yellow

TX17734-1Y/Y	2	2022	Dalhart	18	22DAL-R	92	Yellow
TX17734-1Y/Y	1	2022	Springlake	4	22SPR-C	111	Yellow
TX17734-1Y/Y	2	2022	Springlake	4	22SPR-C	76	Yellow
TX17734-1Y/Y	1	2022	Springlake	18	22SPR-R	59	Yellow
TX17734-1Y/Y	2	2022	Springlake	18	22SPR-R	59	Yellow
TX17742-19R/R	1	2022	Dalhart	4	22DAL-C	172	Red
TX17742-19R/R	2	2022	Dalhart	4	22DAL-C	172	Red
TX17742-19R/R	1	2022	Dalhart	18	22DAL-R	120	Red
TX17742-19R/R	2	2022	Dalhart	18	22DAL-R	120	Red
TX17742-19R/R	1	2022	Springlake	4	22SPR-C	111	Red
TX17742-19R/R	2	2022	Springlake	4	22SPR-C	111	Red
TX17742-19R/R	1	2022	Springlake	18	22SPR-R	59	Red
TX17742-19R/R	2	2022	Springlake	18	22SPR-R	73	Red
TX17742-23P/P	1	2022	Dalhart	4	22DAL-C	151	Purple
TX17742-23P/P	2	2022	Dalhart	4	22DAL-C	158	Purple
TX17742-23P/P	1	2022	Dalhart	18	22DAL-R	120	Purple
TX17742-23P/P	2	2022	Dalhart	18	22DAL-R	113	Purple
TX17742-23P/P	1	2022	Springlake	4	22SPR-C	111	Purple
TX17742-23P/P	2	2022	Springlake	4	22SPR-C	111	Purple
TX17742-23P/P	1	2022	Springlake	18	22SPR-R	87	Purple
TX17742-23P/P	2	2022	Springlake	18	22SPR-R	87	Purple
TX17742-5YPpinto/YP	1	2022	Dalhart	4	22DAL-C	130	Purple
TX17742-5YPpinto/YP	2	2022	Dalhart	4	22DAL-C	151	Purple
TX17742-5YPpinto/YP	1	2022	Dalhart	18	22DAL-R	92	Purple
TX17742-5YPpinto/YP	2	2022	Dalhart	18	22DAL-R	92	Purple
TX17742-5YPpinto/YP	1	2022	Springlake	4	22SPR-C	111	Purple
TX17742-5YPpinto/YP	2	2022	Springlake	4	22SPR-C	111	Purple
TX17742-5YPpinto/YP	1	2022	Springlake	18	22SPR-R	59	Purple
TX17742-5YPpinto/YP	2	2022	Springlake	18	22SPR-R	46	Purple
TX17755-2Psplash	1	2022	Dalhart	4	22DAL-C	158	Yellow
TX17755-2Psplash	2	2022	Dalhart	4	22DAL-C	158	Yellow
TX17755-2Psplash	1	2022	Dalhart	18	22DAL-R	106	Yellow
TX17755-2Psplash	2	2022	Dalhart	18	22DAL-R	106	Yellow
TX17755-2Psplash	1	2022	Springlake	4	22SPR-C	111	Yellow
TX17755-2Psplash	2	2022	Springlake	4	22SPR-C	111	Yellow
TX17755-2Psplash	1	2022	Springlake	18	22SPR-R	66	Yellow
TX17755-2Psplash	2	2022	Springlake	18	22SPR-R	73	Yellow
TX17763-2Y/Y	1	2022	Dalhart	4	22DAL-C	158	Yellow
TX17763-2Y/Y	2	2022	Dalhart	4	22DAL-C	137	Yellow
TX17763-2Y/Y	1	2022	Dalhart	18	22DAL-R	106	Yellow
TX17763-2Y/Y	2	2022	Dalhart	18	22DAL-R	106	Yellow
TX17763-2Y/Y	1	2022	Springlake	4	22SPR-C	111	Yellow
TX17763-2Y/Y	2	2022	Springlake	4	22SPR-C	111	Yellow
TX17763-2Y/Y	1	2022	Springlake	18	22SPR-R	59	Yellow
TX17763-2Y/Y	2	2022	Springlake	18	22SPR-R	59	Yellow
TX17788f-4R/R	1	2022	Dalhart	4	22DAL-C	158	Red
TX17788f-4R/R	2	2022	Dalhart	4	22DAL-C	158	Red

TX17788f-4R/R	1	2022	Dalhart	18	22DAL-R	92	Red
TX17788f-4R/R	2	2022	Dalhart	18	22DAL-R	92	Red
TX17788f-4R/R	1	2022	Springlake	4	22SPR-C	111	Red
TX17788f-4R/R	2	2022	Springlake	4	22SPR-C	111	Red
TX17788f-4R/R	1	2022	Springlake	18	22SPR-R	52	Red
TX17788f-4R/R	2	2022	Springlake	18	22SPR-R	59	Red
TX17797-11Y/Y	1	2022	Dalhart	4	22DAL-C	116	Yellow
TX17797-11Y/Y	2	2022	Dalhart	4	22DAL-C	116	Yellow
TX17797-11Y/Y	1	2022	Dalhart	18	22DAL-R	78	Yellow
TX17797-11Y/Y	2	2022	Dalhart	18	22DAL-R	71	Yellow
TX17797-11Y/Y	1	2022	Springlake	4	22SPR-C	83	Yellow
TX17797-11Y/Y	2	2022	Springlake	4	22SPR-C		Yellow
TX17797-11Y/Y	1	2022	Springlake	18	22SPR-R	46	Yellow
TX17797-11Y/Y	2	2022	Springlake	18	22SPR-R	46	Yellow
TX17805-10RYpinto/Y	1	2022	Dalhart	4	22DAL-C	158	Red
TX17805-10RYpinto/Y	2	2022	Dalhart	4	22DAL-C	137	Red
TX17805-10RYpinto/Y	1	2022	Dalhart	18	22DAL-R	106	Red
TX17805-10RYpinto/Y	2	2022	Dalhart	18	22DAL-R	106	Red
TX17805-10RYpinto/Y	1	2022	Springlake	4	22SPR-C	111	Red
TX17805-10RYpinto/Y	2	2022	Springlake	4	22SPR-C	111	Red
TX17805-10RYpinto/Y	1	2022	Springlake	18	22SPR-R	66	Red
TX17805-10RYpinto/Y	2	2022	Springlake	18	22SPR-R	73	Red
TX17805-12R/R	1	2022	Dalhart	4	22DAL-C	137	Red
TX17805-12R/R	2	2022	Dalhart	4	22DAL-C	151	Red
TX17805-12R/R	1	2022	Dalhart	18	22DAL-R	169	Red
TX17805-12R/R	2	2022	Dalhart	18	22DAL-R	176	Red
TX17805-12R/R	1	2022	Springlake	4	22SPR-C	111	Red
TX17805-12R/R	2	2022	Springlake	4	22SPR-C	111	Red
TX17805-12R/R	1	2022	Springlake	18	22SPR-R	159	Red
TX17805-12R/R	2	2022	Springlake	18	22SPR-R	159	Red
TX17805-1RWpinto/R	1	2022	Dalhart	4	22DAL-C	137	Red
TX17805-1RWpinto/R	2	2022	Dalhart	4	22DAL-C	130	Red
TX17805-1RWpinto/R	1	2022	Dalhart	18	22DAL-R	106	Red
TX17805-1RWpinto/R	2	2022	Dalhart	18	22DAL-R	106	Red
TX17805-1RWpinto/R	1	2022	Springlake	4	22SPR-C	111	Red
TX17805-1RWpinto/R	2	2022	Springlake	4	22SPR-C	111	Red
TX17805-1RWpinto/R	1	2022	Springlake	18	22SPR-R	66	Red
TX17805-1RWpinto/R	2	2022	Springlake	18	22SPR-R	66	Red
TX17805-8R/R	1	2022	Dalhart	4	22DAL-C	137	Red
TX17805-8R/R	2	2022	Dalhart	4	22DAL-C	137	Red
TX17805-8R/R	1	2022	Dalhart	18	22DAL-R	106	Red
TX17805-8R/R	2	2022	Dalhart	18	22DAL-R	106	Red
TX17805-8R/R	1	2022	Springlake	4	22SPR-C	111	Red
TX17805-8R/R	2	2022	Springlake	4	22SPR-C	111	Red
TX17805-8R/R	1	2022	Springlake	18	22SPR-R	87	Red
TX17805-8R/R	2	2022	Springlake	18	22SPR-R	80	Red
TX17846-1W	1	2022	Dalhart	4	22DAL-C	137	Chipper

TX17846-1W	2	2022	Dalhart	4	22DAL-C	158	Chipper
TX17846-1W	1	2022	Dalhart	18	22DAL-R	85	Chipper
TX17846-1W	2	2022	Dalhart	18	22DAL-R	78	Chipper
TX17846-1W	1	2022	Springlake	4	22SPR-C	102	Chipper
TX17846-1W	2	2022	Springlake	4	22SPR-C	93	Chipper
TX17846-1W	1	2022	Springlake	18	22SPR-R	57	Chipper
TX17846-1W	2	2022	Springlake	18	22SPR-R	55	Chipper
TX18042-1Ru	1	2022	Dalhart	4	22DAL-C	130	Chipper
TX18042-1Ru	2	2022	Dalhart	4	22DAL-C	158	Chipper
TX18042-1Ru	1	2022	Dalhart	18	22DAL-R	78	Chipper
TX18042-1Ru	2	2022	Dalhart	18	22DAL-R	63	Chipper
TX18042-1Ru	1	2022	Springlake	4	22SPR-C	102	Chipper
TX18042-1Ru	2	2022	Springlake	4	22SPR-C	93	Chipper
TX18042-1Ru	1	2022	Springlake	18	22SPR-R	50	Chipper
TX18042-1Ru	2	2022	Springlake	18	22SPR-R	34	Chipper
TX18192-2R/Y	1	2022	Dalhart	4	22DAL-C	130	Red
TX18192-2R/Y	2	2022	Dalhart	4	22DAL-C	88	Red
TX18192-2R/Y	1	2022	Dalhart	18	22DAL-R	85	Red
TX18192-2R/Y	2	2022	Dalhart	18	22DAL-R	85	Red
TX18192-2R/Y	1	2022	Springlake	4	22SPR-C	111	Red
TX18192-2R/Y	2	2022	Springlake	4	22SPR-C	90	Red
TX18192-2R/Y	1	2022	Springlake	18	22SPR-R	52	Red
TX18192-2R/Y	2	2022	Springlake	18	22SPR-R	73	Red
TX19009s-2W/Y	1	2022	Dalhart	4	22DAL-C	88	Chipper
TX19009s-2W/Y	2	2022	Dalhart	4	22DAL-C	116	Chipper
TX19009s-2W/Y	1	2022	Dalhart	18	22DAL-R	85	Chipper
TX19009s-2W/Y	2	2022	Dalhart	18	22DAL-R	78	Chipper
TX19009s-2W/Y	1	2022	Springlake	4	22SPR-C	74	Chipper
TX19009s-2W/Y	2	2022	Springlake	4	22SPR-C	72	Chipper
TX19009s-2W/Y	1	2022	Springlake	18	22SPR-R	57	Chipper
TX19009s-2W/Y	2	2022	Springlake	18	22SPR-R	55	Chipper
TX19083-1Ru	1	2022	Dalhart	4	22DAL-C	157	Russet
TX19083-1Ru	2	2022	Dalhart	4	22DAL-C	143	Russet
TX19083-1Ru	1	2022	Dalhart	18	22DAL-R	91	Russet
TX19083-1Ru	2	2022	Dalhart	18	22DAL-R	77	Russet
TX19083-1Ru	1	2022	Springlake	4	22SPR-C	99	Russet
TX19083-1Ru	2	2022	Springlake	4	22SPR-C	86	Russet
TX19083-1Ru	1	2022	Springlake	18	22SPR-R	40	Russet
TX19083-1Ru	2	2022	Springlake	18	22SPR-R	48	Russet
TX19093-1Rure	1	2022	Dalhart	4	22DAL-C	108	Russet
TX19093-1Rure	2	2022	Dalhart	4	22DAL-C	101	Russet
TX19093-1Rure	1	2022	Dalhart	18	22DAL-R	77	Russet
TX19093-1Rure	2	2022	Dalhart	18	22DAL-R	91	Russet
TX19093-1Rure	1	2022	Springlake	4	22SPR-C	99	Russet
TX19093-1Rure	2	2022	Springlake	4	22SPR-C	65	Russet
TX19093-1Rure	1	2022	Springlake	18	22SPR-R	40	Russet
TX19093-1Rure	2	2022	Springlake	18	22SPR-R	48	Russet

TX19407-5Ru	1	2022	Dalhart	4	22DAL-C	88	Chipper
TX19407-5Ru	2	2022	Dalhart	4	22DAL-C	88	Chipper
TX19407-5Ru	1	2022	Dalhart	18	22DAL-R	78	Chipper
TX19407-5Ru	2	2022	Dalhart	18	22DAL-R	63	Chipper
TX19407-5Ru	1	2022	Springlake	4	22SPR-C	67	Chipper
TX19407-5Ru	2	2022	Springlake	4	22SPR-C	72	Chipper
TX19407-5Ru	1	2022	Springlake	18	22SPR-R	37	Chipper
TX19407-5Ru	2	2022	Springlake	18	22SPR-R	34	Chipper
TX19411-4W	1	2022	Dalhart	4	22DAL-C	158	Chipper
TX19411-4W	2	2022	Dalhart	4	22DAL-C	193	Chipper
TX19411-4W	1	2022	Dalhart	18	22DAL-R	78	Chipper
TX19411-4W	2	2022	Dalhart	18	22DAL-R	85	Chipper
TX19411-4W	1	2022	Springlake	4	22SPR-C	102	Chipper
TX19411-4W	2	2022	Springlake	4	22SPR-C	93	Chipper
TX19411-4W	1	2022	Springlake	18	22SPR-R	57	Chipper
TX19411-4W	2	2022	Springlake	18	22SPR-R	55	Chipper
TX19460-1W	1	2022	Dalhart	4	22DAL-C	102	Chipper
TX19460-1W	2	2022	Dalhart	4	22DAL-C	130	Chipper
TX19460-1W	1	2022	Dalhart	18	22DAL-R	67	Chipper
TX19460-1W	2	2022	Dalhart	18	22DAL-R	71	Chipper
TX19460-1W	1	2022	Springlake	4	22SPR-C	88	Chipper
TX19460-1W	2	2022	Springlake	4	22SPR-C	93	Chipper
TX19460-1W	1	2022	Springlake	18	22SPR-R	43	Chipper
TX19460-1W	2	2022	Springlake	18	22SPR-R	41	Chipper
TX19489s-3Y/Y	1	2022	Dalhart	4	22DAL-C	116	Yellow
TX19489s-3Y/Y	2	2022	Dalhart	4	22DAL-C	116	Yellow
TX19489s-3Y/Y	1	2022	Dalhart	18	22DAL-R	85	Yellow
TX19489s-3Y/Y	2	2022	Dalhart	18	22DAL-R	85	Yellow
TX19489s-3Y/Y	1	2022	Springlake	4	22SPR-C	153	Yellow
TX19489s-3Y/Y	2	2022	Springlake	4	22SPR-C	111	Yellow
TX19489s-3Y/Y	1	2022	Springlake	18	22SPR-R	73	Yellow
TX19489s-3Y/Y	2	2022	Springlake	18	22SPR-R	80	Yellow
TX19495-16RYpinto	1	2022	Dalhart	18	22DAL-R		Red
TX19495-16RYpinto	2	2022	Dalhart	18	22DAL-R		Red
TX19495-16RYpinto	1	2022	Springlake	4	22SPR-C	111	Red
TX19495-16RYpinto	2	2022	Springlake	4	22SPR-C	111	Red
TX19495-16RYpinto	1	2022	Springlake	18	22SPR-R	80	Red
TX19495-16RYpinto	2	2022	Springlake	18	22SPR-R	87	Red
TX19495-17R/Y-R	1	2022	Dalhart	4	22DAL-C	158	Red
TX19495-17R/Y-R	2	2022	Dalhart	4	22DAL-C	179	Red
TX19495-17R/Y-R	1	2022	Dalhart	18	22DAL-R	120	Red
TX19495-17R/Y-R	2	2022	Dalhart	18	22DAL-R	120	Red
TX19495-17R/Y-R	1	2022	Springlake	4	22SPR-C	111	Red
TX19495-17R/Y-R	2	2022	Springlake	4	22SPR-C	93	Red
TX19495-17R/Y-R	1	2022	Springlake	18	22SPR-R	80	Red
TX19495-17R/Y-R	2	2022	Springlake	18	22SPR-R	69	Red
TX19495-9RYpinto	1	2022	Dalhart	4	22DAL-C	123	Red

TX19495-9RYpinto	2	2022	Dalhart	4	22DAL-C	116	Red
TX19495-9RYpinto	1	2022	Dalhart	18	22DAL-R	113	Red
TX19495-9RYpinto	2	2022	Dalhart	18	22DAL-R	113	Red
TX19495-9RYpinto	1	2022	Springlake	4	22SPR-C	111	Red
TX19495-9RYpinto	2	2022	Springlake	4	22SPR-C	93	Red
TX19495-9RYpinto	1	2022	Springlake	18	22SPR-R	73	Red
TX19495-9RYpinto	2	2022	Springlake	18	22SPR-R	62	Red
TX19508-5RYpinto/Y	1	2022	Dalhart	4	22DAL-C	137	Red
TX19508-5RYpinto/Y	2	2022	Dalhart	4	22DAL-C	137	Red
TX19508-5RYpinto/Y	1	2022	Dalhart	18	22DAL-R	92	Red
TX19508-5RYpinto/Y	2	2022	Dalhart	18	22DAL-R	106	Red
TX19508-5RYpinto/Y	1	2022	Springlake	4	22SPR-C	111	Red
TX19508-5RYpinto/Y	2	2022	Springlake	4	22SPR-C	93	Red
TX19508-5RYpinto/Y	1	2022	Springlake	18	22SPR-R	66	Red
TX19508-5RYpinto/Y	2	2022	Springlake	18	22SPR-R	48	Red
TX19511-5RYpinto/Y	1	2022	Dalhart	4	22DAL-C	88	Red
TX19511-5RYpinto/Y	2	2022	Dalhart	4	22DAL-C	88	Red
TX19511-5RYpinto/Y	1	2022	Dalhart	18	22DAL-R	67	Red
TX19511-5RYpinto/Y	2	2022	Dalhart	18	22DAL-R	67	Red
TX19511-5RYpinto/Y	1	2022	Springlake	4	22SPR-C	90	Red
TX19511-5RYpinto/Y	2	2022	Springlake	4	22SPR-C		Red
TX19511-5RYpinto/Y	1	2022	Springlake	18	22SPR-R	59	Red
TX19511-5RYpinto/Y	2	2022	Springlake	18	22SPR-R	41	Red
TX6-1216-1Ru	1	2019	Dalhart	18	19DAL-R	70	Russet
TX6-1216-1Ru	2	2019	Dalhart	18	19DAL-R		Russet
TX6-1216-1Ru	1	2020	Dalhart	4	20DAL-C	133	Russet
TX6-1216-1Ru	2	2020	Dalhart	4	20DAL-C	133	Russet
TX6-1216-1Ru	1	2020	Dalhart	18	20DAL-R	81	Russet
TX6-1216-1Ru	2	2020	Dalhart	18	20DAL-R	95	Russet
TX6-1216-1Ru	1	2020	Springlake	4	20SPR-C	102	Russet
TX6-1216-1Ru	2	2020	Springlake	4	20SPR-C	88	Russet
TX6-1216-1Ru	1	2020	Springlake	18	20SPR-R	60	Russet
TX6-1216-1Ru	2	2020	Springlake	18	20SPR-R	53	Russet
TX6-1216-1Ru	1	2021	Springlake	4	21SPR-C	100	Russet
TX6-1216-1Ru	2	2021	Springlake	4	21SPR-C	80	Russet
TX6-1216-1Ru	1	2021	Springlake	18	21SPR-R	63	Russet
TX6-1216-1Ru	2	2021	Springlake	18	21SPR-R	84	Russet
TXA549-1Ru	1	2019	Dalhart	18	19DAL-R	91	Russet
TXA549-1Ru	2	2019	Dalhart	18	19DAL-R	105	Russet
TXA549-1Ru	1	2020	Dalhart	4	20DAL-C	133	Russet
TXA549-1Ru	2	2020	Dalhart	4	20DAL-C	123	Russet
TXA549-1Ru	1	2020	Dalhart	18	20DAL-R	109	Russet
TXA549-1Ru	2	2020	Dalhart	18	20DAL-R	117	Russet
TXA549-1Ru	1	2020	Springlake	4	20SPR-C	95	Russet
TXA549-1Ru	2	2020	Springlake	4	20SPR-C	109	Russet
TXA549-1Ru	1	2020	Springlake	18	20SPR-R	102	Russet
TXA549-1Ru	2	2020	Springlake	18	20SPR-R	81	Russet

TXA549-1Ru	1	2021	Springlake	4	21SPR-C	100	Russet
TXA549-1Ru	2	2021	Springlake	4	21SPR-C	100	Russet
TXA549-1Ru	1	2021	Springlake	18	21SPR-R	119	Russet
TXA549-1Ru	2	2021	Springlake	18	21SPR-R	91	Russet
TXNS106	1	2019	Dalhart	18	19DAL-R	98	Russet
TXNS106	2	2019	Dalhart	18	19DAL-R		Russet
TXNS106	1	2020	Dalhart	4	20DAL-C	144	Russet
TXNS106	2	2020	Dalhart	4	20DAL-C	133	Russet
TXNS106	1	2020	Dalhart	18	20DAL-R	102	Russet
TXNS106	2	2020	Dalhart	18	20DAL-R	117	Russet
TXNS106	1	2020	Springlake	4	20SPR-C		Russet
TXNS106	2	2020	Springlake	4	20SPR-C		Russet
TXNS106	1	2020	Springlake	18	20SPR-R		Russet
TXNS106	2	2020	Springlake	18	20SPR-R		Russet
TXNS106	1	2021	Springlake	4	21SPR-C	114	Russet
TXNS106	2	2021	Springlake	4	21SPR-C	121	Russet
TXNS106	1	2021	Springlake	18	21SPR-R	105	Russet
TXNS106	2	2021	Springlake	18	21SPR-R	105	Russet
TXNS118	1	2019	Dalhart	18	19DAL-R	105	Russet
TXNS118	2	2019	Dalhart	18	19DAL-R	112	Russet
TXNS118	1	2020	Dalhart	4	20DAL-C	158	Russet
TXNS118	2	2020	Dalhart	4	20DAL-C	165	Russet
TXNS118	1	2020	Dalhart	18	20DAL-R	102	Russet
TXNS118	2	2020	Dalhart	18	20DAL-R	109	Russet
TXNS118	1	2020	Springlake	4	20SPR-C	102	Russet
TXNS118	2	2020	Springlake	4	20SPR-C	116	Russet
TXNS118	1	2020	Springlake	18	20SPR-R	81	Russet
TXNS118	2	2020	Springlake	18	20SPR-R	74	Russet
TXNS118	1	2021	Springlake	4	21SPR-C	107	Russet
TXNS118	2	2021	Springlake	4	21SPR-C	94	Russet
TXNS118	1	2021	Springlake	18	21SPR-R	98	Russet
TXNS118	2	2021	Springlake	18	21SPR-R	105	Russet
TXNS249	1	2019	Dalhart	18	19DAL-R	98	Russet
TXNS249	2	2019	Dalhart	18	19DAL-R	105	Russet
TXNS249	1	2020	Dalhart	4	20DAL-C	151	Russet
TXNS249	2	2020	Dalhart	4	20DAL-C	144	Russet
TXNS249	1	2020	Dalhart	18	20DAL-R	102	Russet
TXNS249	2	2020	Dalhart	18	20DAL-R	102	Russet
TXNS249	1	2020	Springlake	4	20SPR-C	109	Russet
TXNS249	2	2020	Springlake	4	20SPR-C	88	Russet
TXNS249	1	2020	Springlake	18	20SPR-R	74	Russet
TXNS249	2	2020	Springlake	18	20SPR-R	81	Russet
TXNS249	1	2021	Springlake	4	21SPR-C	107	Russet
TXNS249	2	2021	Springlake	4	21SPR-C	121	Russet
TXNS249	1	2021	Springlake	18	21SPR-R	105	Russet
TXNS249	2	2021	Springlake	18	21SPR-R	98	Russet
TXYG79	1	2019	Dalhart	18	19DAL-R	63	Yellow

TXYG79	2	2019	Dalhart	18	19DAL-R	63	Yellow
TXYG79	1	2020	Dalhart	4	20DAL-C		Yellow
TXYG79	2	2020	Dalhart	4	20DAL-C	116	Yellow
TXYG79	1	2020	Dalhart	18	20DAL-R	81	Yellow
TXYG79	2	2020	Dalhart	18	20DAL-R	81	Yellow
TXYG79	1	2020	Springlake	4	20SPR-C	101	Yellow
TXYG79	2	2020	Springlake	4	20SPR-C	115	Yellow
TXYG79	1	2020	Springlake	18	20SPR-R	52	Yellow
TXYG79	2	2020	Springlake	18	20SPR-R	66	Yellow
TXYG79	1	2021	Springlake	4	21SPR-C	119	Yellow
TXYG79	2	2021	Springlake	4	21SPR-C	126	Yellow
TXYG79	1	2021	Springlake	18	21SPR-R	63	Yellow
TXYG79	2	2021	Springlake	18	21SPR-R	63	Yellow
UMTX383-3Yrdspl/Y	1	2019	Dalhart	18	19DAL-R	56	Red
UMTX383-3Yrdspl/Y	2	2019	Dalhart	18	19DAL-R	56	Red
UMTX383-3Yrdspl/Y	1	2020	Dalhart	4	20DAL-C	88	Red
UMTX383-3Yrdspl/Y	2	2020	Dalhart	4	20DAL-C		Red
UMTX383-3Yrdspl/Y	1	2020	Dalhart	18	20DAL-R	60	Red
UMTX383-3Yrdspl/Y	2	2020	Dalhart	18	20DAL-R	53	Red
UMTX383-3Yrdspl/Y	1	2020	Springlake	4	20SPR-C	101	Red
UMTX383-3Yrdspl/Y	2	2020	Springlake	4	20SPR-C	94	Red
UMTX383-3Yrdspl/Y	1	2020	Springlake	18	20SPR-R	45	Red
UMTX383-3Yrdspl/Y	2	2020	Springlake	18	20SPR-R	45	Red
UMTX383-3Yrdspl/Y	1	2021	Springlake	4	21SPR-C	119	Red
UMTX383-3Yrdspl/Y	2	2021	Springlake	4	21SPR-C	119	Red
UMTX383-3Yrdspl/Y	1	2021	Springlake	18	21SPR-R	70	Red
UMTX383-3Yrdspl/Y	2	2021	Springlake	18	21SPR-R	56	Red
Vanguard Russet	1	2019	Dalhart	18	19DAL-R	133	Russet
Vanguard Russet	2	2019	Dalhart	18	19DAL-R	126	Russet
Vanguard Russet	1	2020	Dalhart	4	20DAL-C		Russet
Vanguard Russet	2	2020	Dalhart	4	20DAL-C	165	Russet
Vanguard Russet	1	2020	Dalhart	18	20DAL-R	144	Russet
Vanguard Russet	2	2020	Dalhart	18	20DAL-R	144	Russet
Vanguard Russet	1	2022	Dalhart	4	22DAL-C	157	Russet
Vanguard Russet	2	2022	Dalhart	4	22DAL-C	150	Russet
Vanguard Russet	1	2022	Dalhart	18	22DAL-R	126	Russet
Vanguard Russet	2	2022	Dalhart	18	22DAL-R	126	Russet
Vanguard Russet	1	2020	Springlake	4	20SPR-C	123	Russet
Vanguard Russet	2	2020	Springlake	4	20SPR-C	137	Russet
Vanguard Russet	1	2020	Springlake	18	20SPR-R	115	Russet
Vanguard Russet	2	2020	Springlake	18	20SPR-R	115	Russet
Vanguard Russet	1	2021	Springlake	4	21SPR-C	114	Russet
Vanguard Russet	2	2021	Springlake	4	21SPR-C	142	Russet
Vanguard Russet	1	2021	Springlake	18	21SPR-R	147	Russet
Vanguard Russet	2	2021	Springlake	18	21SPR-R	133	Russet
Vanguard Russet	1	2022	Springlake	4	22SPR-C	102	Russet
Vanguard Russet	2	2022	Springlake	4	22SPR-C	102	Russet

Vanguard Russet	1	2022	Springlake	18	22SPR-R	96	Russet
Vanguard Russet	2	2022	Springlake	18	22SPR-R	103	Russet
White LaSoda	1	2019	Dalhart	18	19DAL-R	77	Red
White LaSoda	2	2019	Dalhart	18	19DAL-R	77	Red
White LaSoda	1	2020	Dalhart	4	20DAL-C	140	Red
White LaSoda	2	2020	Dalhart	4	20DAL-C	140	Red
White LaSoda	1	2020	Dalhart	18	20DAL-R	95	Red
White LaSoda	2	2020	Dalhart	18	20DAL-R	95	Red
White LaSoda	1	2020	Springlake	4	20SPR-C	136	Red
White LaSoda	2	2020	Springlake	4	20SPR-C		Red
White LaSoda	1	2020	Springlake	18	20SPR-R	73	Red
White LaSoda	2	2020	Springlake	18	20SPR-R	80	Red
White LaSoda	1	2021	Springlake	4	21SPR-C	154	Red
White LaSoda	2	2021	Springlake	4	21SPR-C	154	Red
White LaSoda	1	2021	Springlake	18	21SPR-R	84	Red
White LaSoda	2	2021	Springlake	18	21SPR-R	91	Red
Yukon Gold	1	2022	Dalhart	4	22DAL-C	123	Yellow
Yukon Gold	2	2022	Dalhart	4	22DAL-C	116	Yellow
Yukon Gold	1	2022	Dalhart	18	22DAL-R	85	Yellow
Yukon Gold	2	2022	Dalhart	18	22DAL-R	85	Yellow
Yukon Gold	1	2022	Springlake	4	22SPR-C	111	Yellow
Yukon Gold	2	2022	Springlake	4	22SPR-C	111	Yellow
Yukon Gold	1	2022	Springlake	18	22SPR-R	52	Yellow
Yukon Gold	2	2022	Springlake	18	22SPR-R	46	Yellow
