# Supplementary material for: Genome‐wide association studies and genomics‐informed breeding for tuber dormancy in tetraploid potatoes
Source: Plant Genome. 2026 Feb 19;19(1):e70205. doi: 10.1002/tpg2.70205 (PMC12919731; doi:10.1002/tpg2.70205)
Supplement: Supplementary file 2 [file TPG2-19-e70205-s004.pdf]

**Genome-wide association studies and genomics-informed breeding for tuber dormancy in tetraploid potatoes**

Ao Jiao, Sanjeev Gautam, Jeewan Pandey, Douglas C. Scheuring, Jeffrey W. Koym, and M. Isabel Vales

**Supplementary Table S6**. Candidate genes, within a 2-Mb (Megabase) flanking genomic region of the peak of each QTL detected for the length of potato tuber dormancy, identified using the potato reference genome of *Solanum tuberosum* group Phureja DM1-3 PGSC v4.03 from SpudDB. Note: Separate sheets were used for each QTL peak.

| chr | start | end | mid | ID | Name | hit_dist |
|---|---|---|---|---|---|---|
| ch01 | 2865723 | 2867672 | 2866697.5 | PGSC0003DMG400021329 | Helix-loop-helix DNA-binding domain containing protein | 679.5 |
| ch01 | 2860255 | 2864212 | 2862233.5 | PGSC0003DMG400021362 | Acyl-carrier-protein S-malonyltransferase/ transferase | 5143.5 |
| ch01 | 2875111 | 2876052 | 2875581.5 | PGSC0003DMG400021361 | Dof zinc finger protein DOF3.5 | 8204.5 |
| ch01 | 2879354 | 2880631 | 2879992.5 | PGSC0003DMG400021360 | U-box protein | 12615.5 |
| ch01 | 2847937 | 2859298 | 2853617.5 | PGSC0003DMG400021363 | Helicase domain-containing protein | 13759.5 |
| ch01 | 2807645 | 2809862 | 2808753.5 | PGSC0003DMG400021331 | PEN1 | 58623.5 |
| ch01 | 2804438 | 2807368 | 2805903 | PGSC0003DMG400021364 | 33 kDa ribonucleoprotein, chloroplastic | 61474 |
| ch01 | 2798848 | 2803545 | 2801196.5 | PGSC0003DMG400021365 | YA1 | 66180.5 |
| ch01 | 2935544 | 2937305 | 2936424.5 | PGSC0003DMG400021324 | Spotted leaf protein | 69047.5 |
| ch01 | 2777315 | 2779888 | 2778601.5 | PGSC0003DMG400021367 | Pentatricopeptide repeat-containing protein | 88775.5 |
| ch01 | 2955443 | 2959120 | 2957281.5 | PGSC0003DMG400021323 | BEL29 protein | 89904.5 |
| ch01 | 2771324 | 2772570 | 2771947 | PGSC0003DMG400021333 | Ring finger protein | 95430 |
| ch01 | 2760364 | 2766127 | 2763245.5 | PGSC0003DMG401021334 | ML domain protein | 104131.5 |
| ch01 | 2757873 | 2758994 | 2758433.5 | PGSC0003DMG402021334 | Electron transporter | 108943.5 |
| ch01 | 2982705 | 2987854 | 2985279.5 | PGSC0003DMG400039398 | Aluminum-activated malate transporter | 117902.5 |
| ch01 | 2688615 | 2693871 | 2691243 | PGSC0003DMG400021337 | Co-chaperone protein HscB, mitochondrial | 176134 |
| ch01 | 2676876 | 2680782 | 2678829 | PGSC0003DMG400021338 | Calcium-dependent protein kinase | 188548 |
| ch01 | 3061072 | 3066062 | 3063567 | PGSC0003DMG400021322 | Digalactosyldiacylglycerol synthase 1 | 196190 |
| ch01 | 2664864 | 2669654 | 2667259 | PGSC0003DMG400021339 | Lymphoid organ expressed yellow head virus receptor protein | 200118 |
| ch01 | 3068949 | 3071477 | 3070213 | PGSC0003DMG400021354 | NC domain-containing protein | 202836 |
| ch01 | 3071733 | 3076949 | 3074341 | PGSC0003DMG400021321 | Serine/threonine-protein kinase rio2 | 206964 |
| ch01 | 2655464 | 2659843 | 2657653.5 | PGSC0003DMG400021372 | Fasciclin-like arabinogalactan protein 14 | 209723.5 |
| ch01 | 3085540 | 3089347 | 3087443.5 | PGSC0003DMG400021353 | Serine/threonine-protein kinase PBS1 | 220066.5 |
| ch01 | 2636858 | 2643415 | 2640136.5 | PGSC0003DMG400021373 | Zinc finger, RING-type | 227240.5 |
| ch01 | 2624593 | 2631661 | 2628127 | PGSC0003DMG400021374 | Methionine aminopeptidase | 239250 |
| ch01 | 3121985 | 3131464 | 3126724.5 | PGSC0003DMG402021320 | Coatomer subunit beta-1 | 259347.5 |
| ch01 | 2605465 | 2610590 | 2608027.5 | PGSC0003DMG400021376 | Exonuclease | 259349.5 |
| ch01 | 2601451 | 2604263 | 2602857 | PGSC0003DMG400043597 | Fringe | 264520 |
| ch01 | 2595304 | 2595992 | 2595648 | PGSC0003DMG400021384 | PGPS/D7 | 271729 |
| ch01 | 2587959 | 2592710 | 2590334.5 | PGSC0003DMG400021341 | Sucrose-phosphatase 1 | 277042.5 |

| | | | | | | |
|---|---|---|---|---|---|---|
| ch01 | 2578483 | 2583492 | 2580987.5 | PGSC0003DMG400021342 | Calcium-dependent protein kinase 20 | 286389.5 |
| ch01 | 3150921 | 3156724 | 3153822.5 | PGSC0003DMG400021351 | Cohesion establishment factor 7 | 286445.5 |
| ch01 | 3157247 | 3160524 | 3158885.5 | PGSC0003DMG400021319 | C2 domain-containing protein | 291508.5 |
| ch01 | 2556992 | 2566447 | 2561719.5 | PGSC0003DMG400021343 | White-brown-complex ABC transporter family | 305657.5 |
| ch01 | 2538088 | 2556723 | 2547405.5 | PGSC0003DMG400021378 | ATP-dependent RNA helicase | 319971.5 |
| ch01 | 3224345 | 3225328 | 3224836.5 | PGSC0003DMG400016420 | VAMP protein SEC22 | 357459.5 |
| ch01 | 2499796 | 2505473 | 2502634.5 | PGSC0003DMG400021346 | Transcription factor | 364742.5 |
| ch01 | 3234950 | 3235780 | 3235365 | PGSC0003DMG400016390 | Hin1 | 367988 |
| ch01 | 2497296 | 2498845 | 2498070.5 | PGSC0003DMG400021347 | UDP-glucosyltransferase | 369306.5 |
| ch01 | 3241142 | 3246196 | 3243669 | PGSC0003DMG400016389 | U3 small nucleolar ribonucleoprotein protein imp4 | 376292 |
| ch01 | 2466455 | 2476089 | 2471272 | PGSC0003DMG400021348 | Subtilisin-type protease | 396105 |
| ch01 | 2460769 | 2464670 | 2462719.5 | PGSC0003DMG400021381 | EIL1 | 404657.5 |
| ch01 | 3283634 | 3289271 | 3286452.5 | PGSC0003DMG400016419 | Cytokinin riboside 5'-monophosphate phosphoribohydrolase LOG7 | 419075.5 |
| ch01 | 3296133 | 3300692 | 3298412.5 | PGSC0003DMG400016387 | Thioredoxin peroxidase | 431035.5 |
| ch01 | 2421019 | 2422447 | 2421733 | PGSC0003DMG400021383 | Oxidoreductase, 2OG-Fe(II) oxygenase family protein | 445644 |
| ch01 | 3311893 | 3317036 | 3314464.5 | PGSC0003DMG400016418 | E2F4,5 | 447087.5 |
| ch01 | 3324727 | 3331069 | 3327898 | PGSC0003DMG400016386 | HVA22 i | 460521 |
| ch01 | 3402394 | 3404656 | 3403525 | PGSC0003DMG400016431 | Reverse transcriptase | 536148 |
| ch01 | 3411973 | 3413078 | 3412525.5 | PGSC0003DMG400016383 | Ccr4-associated factor | 545148.5 |
| ch01 | 3437177 | 3441780 | 3439478.5 | PGSC0003DMG400016382 | Ubiquitin-conjugating enzyme family protein | 572101.5 |
| ch01 | 3502584 | 3504030 | 3503307 | PGSC0003DMG400016379 | Zinc finger protein | 635930 |
| ch01 | 3519482 | 3522279 | 3520880.5 | PGSC0003DMG400016377 | BHLH transcription factor | 653503.5 |
| ch01 | 3532561 | 3539645 | 3536103 | PGSC0003DMG400016376 | Tobamovirus multiplication 3 | 668726 |
| ch01 | 3546392 | 3554819 | 3550605.5 | PGSC0003DMG400016412 | Succinyl-CoA ligase alpha 1 subunit | 683228.5 |
| ch01 | 3555785 | 3558766 | 3557275.5 | PGSC0003DMG400016411 | Isochorismatase | 689898.5 |
| ch01 | 3561734 | 3563050 | 3562392 | PGSC0003DMG400016375 | Anthranilate N-benzoyltransferase protein | 695015 |
| ch01 | 3563812 | 3567617 | 3565714.5 | PGSC0003DMG400016374 | F-box protein | 698337.5 |
| ch01 | 3571031 | 3572031 | 3571531 | PGSC0003DMG400016373 | Alanine-2-oxoglutarate aminotransferase 1 | 704154 |
| ch01 | 3573820 | 3580122 | 3576971 | PGSC0003DMG400016410 | Transposase | 709594 |
| ch01 | 2151329 | 2152141 | 2151735 | PGSC0003DMG400032207 | Lipoxygenase | 715642 |
| ch01 | 3589210 | 3593472 | 3591341 | PGSC0003DMG400016372 | Resistance protein PSH-RGH7 | 723964 |
| ch01 | 3596648 | 3598780 | 3597714 | PGSC0003DMG400016371 | Peroxidase 44 | 730337 |
| ch01 | 3599829 | 3605009 | 3602419 | PGSC0003DMG401016370 | Lipase | 735042 |
| ch01 | 3617273 | 3617745 | 3617509 | PGSC0003DMG400016429 | Kinase | 750132 |
| ch01 | 2090383 | 2095569 | 2092976 | PGSC0003DMG400032154 | Alcohol dehydrogenase | 774401 |

| | | | | | | |
|---|---|---|---|---|---|---|
| ch01 | 2084195 | 2089035 | 2086615 | PGSC0003DMG400032206 | Pentatricopeptide repeat-containing protein, chloroplastic | 780762 |
| ch01 | 3650550 | 3651557 | 3651053.5 | PGSC0003DMG400016409 | Protein kinase family protein | 783676.5 |
| ch01 | 2075479 | 2082422 | 2078950.5 | PGSC0003DMG400032205 | Selenoprotein O | 788426.5 |
| ch01 | 2051651 | 2051976 | 2051813.5 | PGSC0003DMG400032236 | COG5125 | 815563.5 |
| ch01 | 3682716 | 3686865 | 3684790.5 | PGSC0003DMG402016407 | ADP-ribosylation factor | 817413.5 |
| ch01 | 2043182 | 2048782 | 2045982 | PGSC0003DMG400032152 | Enoyl-acyl-carrier-protein reductase | 821395 |
| ch01 | 3690775 | 3694788 | 3692781.5 | PGSC0003DMG401016407 | OBP32pep | 825404.5 |
| ch01 | 3694969 | 3698957 | 3696963 | PGSC0003DMG400016369 | Nucleoside transporter | 829586 |
| ch01 | 3708814 | 3710345 | 3709579.5 | PGSC0003DMG400016368 | Ubiquitin-protein ligase | 842202.5 |
| ch01 | 3711223 | 3713638 | 3712430.5 | PGSC0003DMG400016405 | Leaf senescence protein | 845053.5 |
| ch01 | 3714757 | 3723678 | 3719217.5 | PGSC0003DMG402016404 | RNase H domain-containing protein | 851840.5 |
| ch01 | 2007377 | 2013255 | 2010316 | PGSC0003DMG400032204 | D12 oleate desaturase | 857061 |
| ch01 | 3732415 | 3740649 | 3736532 | PGSC0003DMG400016367 | Ribosomal protein S27 | 869155 |
| ch01 | 3748840 | 3752545 | 3750692.5 | PGSC0003DMG400016403 | Transferase, transferring glycosyl groups | 883315.5 |
| ch01 | 1974045 | 1975488 | 1974766.5 | PGSC0003DMG402032203 | Extensin | 892610.5 |
| ch01 | 3760162 | 3763399 | 3761780.5 | PGSC0003DMG400016402 | Obtusifoliol 14alpha-demethylase | 894403.5 |
| ch01 | 1969263 | 1969672 | 1969467.5 | PGSC0003DMG400032233 | Cysteine-rich extensin-2 | 897909.5 |
| ch01 | 1929493 | 1935765 | 1932629 | PGSC0003DMG400032151 | U box | 934748 |
| ch01 | 1926908 | 1928815 | 1927861.5 | PGSC0003DMG400032232 | U-box domain-containing protein | 939515.5 |
| ch01 | 3818246 | 3821930 | 3820088 | PGSC0003DMG400016365 | Phytosulfokine receptor | 952711 |
| ch01 | 1895208 | 1918478 | 1906843 | PGSC0003DMG400032202 | Callose synthase 3 | 960534 |
| ch01 | 3836168 | 3837130 | 3836649 | PGSC0003DMG400036477 | Tetratricopeptide repeat-containing protein | 969272 |
| ch01 | 3847699 | 3852524 | 3850111.5 | PGSC0003DMG400016400 | Zinc finger CCCH domain-containing protein 32 | 982734.5 |
| ch01 | 1828099 | 1849607 | 1838853 | PGSC0003DMG400032200 | 1,3-beta-glucan synthase | 1028524 |
| ch01 | 3911390 | 3922736 | 3917063 | PGSC0003DMG400016363 | Transcription factor TCP7 | 1049686 |
| ch01 | 1799935 | 1805184 | 1802559.5 | PGSC0003DMG400032150 | DEAD/DEAH box helicase family protein | 1064817.5 |
| ch01 | 3931848 | 3936000 | 3933924 | PGSC0003DMG400016362 | Monosaccharide transporter 1 | 1066547 |
| ch01 | 1793983 | 1798785 | 1796384 | PGSC0003DMG400032149 | Structural molecule | 1070993 |
| ch01 | 3938178 | 3941009 | 3939593.5 | PGSC0003DMG400016361 | Thioredoxin x | 1072216.5 |
| ch01 | 1783477 | 1784286 | 1783881.5 | PGSC0003DMG400032231 | NDR1 | 1083495.5 |
| ch01 | 3952873 | 3958333 | 3955603 | PGSC0003DMG400016397 | Prefoldin subunit 5 | 1088226 |
| ch01 | 3959400 | 3963503 | 3961451.5 | PGSC0003DMG402016396 | Phosphotyrosyl phosphatase activator protein | 1094074.5 |
| ch01 | 3965155 | 3974504 | 3969829.5 | PGSC0003DMG401016396 | Dihydroorotate dehydrogenase | 1102452.5 |
| ch01 | 1761454 | 1764298 | 1762876 | PGSC0003DMG400032199 | Peroxidase | 1104501 |
| ch01 | 3975107 | 3976846 | 3975976.5 | PGSC0003DMG400016360 | 3'-N-debenzoyl-2'-deoxytaxol N-benzoyltransferase | 1108599.5 |

| | | | | | | |
|---|---|---|---|---|---|---|
| ch01 | 1742874 | 1749507 | 1746190.5 | PGSC0003DMG400032146 | 10-formyltetrahydrofolate synthetase | 1121186.5 |
| ch01 | 1737327 | 1740870 | 1739098.5 | PGSC0003DMG400032145 | Lipoate-protein ligase B | 1128278.5 |
| ch01 | 1724682 | 1729852 | 1727267 | PGSC0003DMG400032197 | Restricted tev movement 1 | 1140110 |
| ch01 | 1717631 | 1724040 | 1720835.5 | PGSC0003DMG400032144 | Cysteine protease ATG4B | 1146541.5 |
| ch01 | 1695771 | 1700088 | 1697929.5 | PGSC0003DMG400032195 | Nse1 non-SMC component of SMC5-6 complex family protein | 1169447.5 |
| ch01 | 4033151 | 4047992 | 4040571.5 | PGSC0003DMG400016395 | Acetyl co-enzyme A carboxylase biotin carboxylase subunit | 1173194.5 |
| ch01 | 1676231 | 1684412 | 1680321.5 | PGSC0003DMG401032194 | Ubiquitin ligase SINAT2 | 1187055.5 |
| ch01 | 4054168 | 4064222 | 4059195 | PGSC0003DMG400016394 | L19 ribosomal protein | 1191818 |
| ch01 | 1669876 | 1674402 | 1672139 | PGSC0003DMG400032193 | GTP-binding protein | 1195238 |
| ch01 | 4064893 | 4067142 | 4066017.5 | PGSC0003DMG400016393 | Pre-mRNA branch site protein p14 | 1198640.5 |
| ch01 | 1666818 | 1668625 | 1667721.5 | PGSC0003DMG400032192 | RRNA-processing protein EBP2 | 1199655.5 |
| ch01 | 1662189 | 1664271 | 1663230 | PGSC0003DMG400032191 | Transcription or splicing factor | 1204147 |
| ch01 | 4070910 | 4076760 | 4073835 | PGSC0003DMG400016358 | 26S proteasome regulatory particle non-ATPase subunit 8 | 1206458 |
| ch01 | 1651485 | 1653969 | 1652727 | PGSC0003DMG400032190 | Acidic ribosomal protein P1a | 1214650 |
| ch01 | 1642133 | 1648615 | 1645374 | PGSC0003DMG400032189 | Metal tolerance protein | 1222003 |
| ch01 | 4095153 | 4099676 | 4097414.5 | PGSC0003DMG400016357 | Hcr9-OR2C | 1230037.5 |
| ch01 | 4099729 | 4100935 | 4100332 | PGSC0003DMG400016424 | Hcr9-OR2A | 1232955 |
| ch01 | 1615105 | 1622855 | 1618980 | PGSC0003DMG400032142 | Casein kinase | 1248397 |
| ch01 | 1605710 | 1611808 | 1608759 | PGSC0003DMG400032187 | Tap46 | 1258618 |
| ch01 | 1576195 | 1577007 | 1576601 | PGSC0003DMG400032229 | Dirigent 2 | 1290776 |
| ch01 | 4191121 | 4199105 | 4195113 | PGSC0003DMG400010706 | Aluminum-activated citrate transporter | 1327736 |
| ch01 | 1533698 | 1542060 | 1537879 | PGSC0003DMG400032140 | KH domain protein | 1329498 |
| ch01 | 1516479 | 1518210 | 1517344.5 | PGSC0003DMG400032182 | Non-specific lipid-transfer protein | 1350032.5 |
| ch01 | 1500584 | 1506897 | 1503740.5 | PGSC0003DMG400032138 | Protein AFR | 1363636.5 |
| ch01 | 1486493 | 1493275 | 1489884 | PGSC0003DMG400032137 | Dead box ATP-dependent RNA helicase | 1377493 |
| ch01 | 1477411 | 1484952 | 1481181.5 | PGSC0003DMG400032180 | Serine/threonine-protein phosphatase | 1386195.5 |
| ch01 | 4257240 | 4264145 | 4260692.5 | PGSC0003DMG400010704 | Calcium-dependent protein kinase 8 | 1393315.5 |
| ch01 | 1459458 | 1462273 | 1460865.5 | PGSC0003DMG400032227 | Amino acid transporter | 1406511.5 |
| ch01 | 1454410 | 1459157 | 1456783.5 | PGSC0003DMG400032135 | Uncharacterized BCR, COG1963 family protein | 1410593.5 |
| ch01 | 4276919 | 4282378 | 4279648.5 | PGSC0003DMG400010698 | Adrenodoxin | 1412271.5 |
| ch01 | 1451057 | 1453250 | 1452153.5 | PGSC0003DMG400032226 | Neutral amino acid transport protein | 1415223.5 |
| ch01 | 4283178 | 4290239 | 4286708.5 | PGSC0003DMG400010703 | Translocation protein sec62 | 1419331.5 |
| ch01 | 1439744 | 1441644 | 1440694 | PGSC0003DMG400032223 | Extensin Dif54 | 1426683 |
| ch01 | 1432776 | 1434117 | 1433446.5 | PGSC0003DMG400032222 | Extensin Dif10 | 1433930.5 |
| ch01 | 4308774 | 4314022 | 4311398 | PGSC0003DMG400010697 | YA4 | 1444021 |

| | | | | | | |
|---|---|---|---|---|---|---|
| ch01 | 1413744 | 1419552 | 1416648 | PGSC0003DMG400032133 | Glucan endo-1,3-beta-glucosidase 4 | 1450729 |
| ch01 | 1405032 | 1411492 | 1408262 | PGSC0003DMG400032132 | Serine/arginine-rich protein | 1459115 |
| ch01 | 1399692 | 1404111 | 1401901.5 | PGSC0003DMG400032131 | Maintenance of killer 16 (Mak16) protein | 1465475.5 |
| ch01 | 1371469 | 1372587 | 1372028 | PGSC0003DMG400032130 | NL0E | 1495349 |
| ch01 | 4374603 | 4379187 | 4376895 | PGSC0003DMG401031484 | DNA repair protein XRCC2 homolog | 1509518 |
| ch01 | 1327911 | 1328314 | 1328112.5 | PGSC0003DMG400032217 | Late blight resistance protein | 1539264.5 |
| ch01 | 1308531 | 1313164 | 1310847.5 | PGSC0003DMG400032129 | Carboxy-lyase | 1556529.5 |
| ch01 | 1286822 | 1288111 | 1287466.5 | PGSC0003DMG400032215 | HEAT | 1579910.5 |
| ch01 | 1281299 | 1283170 | 1282234.5 | PGSC0003DMG400032173 | Importin beta-3 | 1585142.5 |
| ch01 | 1279054 | 1281173 | 1280113.5 | PGSC0003DMG400032172 | HEAT repeat family protein | 1587263.5 |
| ch01 | 4479857 | 4486139 | 4482998 | PGSC0003DMG401010496 | X-ray repair cross complementing protein 2, xrcc2 | 1615621 |
| ch01 | 4492632 | 4493071 | 4492851.5 | PGSC0003DMG400010489 | Receptor serine/threonine kinase | 1625474.5 |
| ch01 | 4502206 | 4503003 | 4502604.5 | PGSC0003DMG400010499 | Stress-induced receptor-like kinase | 1635227.5 |
| ch01 | 4534430 | 4535052 | 4534741 | PGSC0003DMG400010498 | Photosystem II 5 kDa protein, chloroplast | 1667364 |
| ch01 | 4536282 | 4539092 | 4537687 | PGSC0003DMG400010497 | Isoform 2 of DNA repair protein XRCC2 homolog | 1670310 |
| ch01 | 1154022 | 1158699 | 1156360.5 | PGSC0003DMG400032169 | Oxoglutarate malate translocator | 1711016.5 |
| ch01 | 1143577 | 1149975 | 1146776 | PGSC0003DMG400032168 | 5'-adenylylsulfate reductase-like 5 | 1720601 |
| ch01 | 1114264 | 1120380 | 1117322 | PGSC0003DMG400032124 | Isocitrate dehydrogenase [NADP] | 1750055 |
| ch01 | 4637077 | 4645541 | 4641309 | PGSC0003DMG400010485 | Poly(P)/ATP NAD kinase | 1773932 |
| ch01 | 1076767 | 1090205 | 1083486 | PGSC0003DMG400032166 | Lysyl-tRNA synthetase | 1783891 |
| ch01 | 4650987 | 4652484 | 4651735.5 | PGSC0003DMG400010484 | BRASSINOSTEROID INSENSITIVE 1-associated receptor kinase 1 | 1784358.5 |
| ch01 | 1066567 | 1069506 | 1068036.5 | PGSC0003DMG400032122 | Binding protein | 1799340.5 |
| ch01 | 4666796 | 4667210 | 4667003 | PGSC0003DMG400010494 | Ulp1 protease family, C-terminal catalytic domain containing protein | 1799626 |
| ch01 | 1062929 | 1065071 | 1064000 | PGSC0003DMG400032210 | Ntp201 | 1803377 |
| ch01 | 1060655 | 1062515 | 1061585 | PGSC0003DMG400032121 | Short chain alcohol dehydrogenase | 1805792 |
| ch01 | 4673152 | 4676876 | 4675014 | PGSC0003DMG400036144 | ZIP transporter | 1807637 |
| ch01 | 1044463 | 1048743 | 1046603 | PGSC0003DMG401032161 | Iaa-amino acid hydrolase 4 | 1820774 |
| ch01 | 4686048 | 4690262 | 4688155 | PGSC0003DMG400010493 | Nucleic acid binding protein | 1820778 |
| ch01 | 1036595 | 1043578 | 1040086.5 | PGSC0003DMG400032120 | Isoform 2 of UPF0496 protein | 1827290.5 |
| ch01 | 4702485 | 4704272 | 4703378.5 | PGSC0003DMG400010483 | Glucan endo-1,3-beta-glucosidase | 1836001.5 |
| ch01 | 1021496 | 1024380 | 1022938 | PGSC0003DMG400032159 | Dynein light chain type 1 family protein | 1844439 |
| ch01 | 1017725 | 1020684 | 1019204.5 | PGSC0003DMG402032158 | F-box protein family | 1848172.5 |
| ch01 | 1010384 | 1013834 | 1012109 | PGSC0003DMG400032119 | Jasmonate ZIM-domain protein 3 | 1855268 |
| ch01 | 4725859 | 4735834 | 4730846.5 | PGSC0003DMG401010492 | Acidic class II 1,3-beta-glucanase | 1863469.5 |
| ch01 | 992189 | 998571 | 995380 | PGSC0003DMG400032118 | CDC48-interacting UBX-domain protein | 1871997 |

| | | | | | | |
|---|---|---|---|---|---|---|
| ch01 | 4738993 | 4739994 | 4739493.5 | PGSC0003DMG400010491 | Glucan endo-1,3-beta-D-glucosidase | 1872116.5 |
| ch01 | 4808244 | 4808912 | 4808578 | PGSC0003DMG400009812 | Cytochrome P450 | 1941201 |
| ch01 | 4847253 | 4848359 | 4847806 | PGSC0003DMG400009810 | Cytochrome P450 hydroxylase | 1980429 |

| chr | start | end | mid | ID | Name | hit_dist |
|---|---|---|---|---|---|---|
| ch01 | 61728105 | 61729376 | 61728740.5 | PGSC0003DMG400031133 | Heat-shock protein | 45201.5 |
| ch01 | 61724332 | 61726516 | 61725424 | PGSC0003DMG400031148 | Oxidoreductase | 48518 |
| ch01 | 61686433 | 61691678 | 61689055.5 | PGSC0003DMG400031149 | Nam 7 | 84886.5 |
| ch01 | 61860884 | 61864152 | 61862518 | PGSC0003DMG400021966 | Laccase | 88576 |
| ch01 | 61677628 | 61680091 | 61678859.5 | PGSC0003DMG400031150 | Protein FRIGIDA | 95082.5 |
| ch01 | 61654202 | 61662237 | 61658219.5 | PGSC0003DMG400031136 | Sur2 hydroxylase/desaturase | 115722.5 |
| ch01 | 61651963 | 61653994 | 61652978.5 | PGSC0003DMG400031152 | Pentatricopeptide repeat-containing protein | 120963.5 |
| ch01 | 61647473 | 61650205 | 61648839 | PGSC0003DMG400031137 | Ripening regulated protein DDTFR8 | 125103 |
| ch01 | 61909635 | 61910149 | 61909892 | PGSC0003DMG400021969 | DNA binding protein | 135950 |
| ch01 | 61919888 | 61920358 | 61920123 | PGSC0003DMG400045386 | Phosphofructokinase | 146181 |
| ch01 | 61618773 | 61628341 | 61623557 | PGSC0003DMG400031154 | Pseudouridine synthase | 150385 |
| ch01 | 61604525 | 61606660 | 61605592.5 | PGSC0003DMG400031138 | MA3 domain-containing protein | 168349.5 |
| ch01 | 61963115 | 61966049 | 61964582 | PGSC0003DMG400021962 | F-box family protein | 190640 |
| ch01 | 61579493 | 61582536 | 61581014.5 | PGSC0003DMG400031140 | WRKY transcription factor | 192927.5 |
| ch01 | 61568524 | 61576469 | 61572496.5 | PGSC0003DMG400031141 | Nt-rab7c protein | 201445.5 |
| ch01 | 61534555 | 61535316 | 61534935.5 | PGSC0003DMG400031146 | NAC transcription factor | 239006.5 |
| ch01 | 61533602 | 61534291 | 61533946.5 | PGSC0003DMG400035454 | Zinc finger CCCH domain-containing protein 42 | 239995.5 |
| ch01 | 61490312 | 61494715 | 61492513.5 | PGSC0003DMG400025052 | ATP synthase 24 kDa subunit, mitochondrial | 281428.5 |
| ch01 | 61484812 | 61486350 | 61485581 | PGSC0003DMG400025073 | Sugar transporter | 288361 |
| ch01 | 62068377 | 62074553 | 62071465 | PGSC0003DMG400027565 | Electron transporter | 297523 |
| ch01 | 61474172 | 61476805 | 61475488.5 | PGSC0003DMG400025074 | Polyadenylate-binding protein | 298453.5 |
| ch01 | 61467750 | 61469288 | 61468519 | PGSC0003DMG400025054 | Hexose transporter 2 | 305423 |
| ch01 | 62089160 | 62092843 | 62091001.5 | PGSC0003DMG400027544 | Trafficking protein particle complex subunit 6b | 317059.5 |
| ch01 | 61452932 | 61456159 | 61454545.5 | PGSC0003DMG400025055 | Hypoxanthine-guanine phosphoribosyltransferase | 319396.5 |
| ch01 | 62097847 | 62106961 | 62102404 | PGSC0003DMG400027563 | DNA-directed RNA polymerase II | 328462 |
| ch01 | 61438028 | 61442579 | 61440303.5 | PGSC0003DMG400025076 | Leucine-rich repeat receptor protein kinase EXS | 333638.5 |
| ch01 | 62106665 | 62109962 | 62108313.5 | PGSC0003DMG400027543 | Sodium/potassium/calcium exchanger | 334371.5 |
| ch01 | 61420993 | 61427977 | 61424485 | PGSC0003DMG400025077 | S1 RNA-binding domain-containing protein | 349457 |
| ch01 | 62132907 | 62137088 | 62134997.5 | PGSC0003DMG400027562 | Transcription factor TGA7 | 361055.5 |
| ch01 | 62140022 | 62144055 | 62142038.5 | PGSC0003DMG401027561 | Ubiquitin-protein ligase | 368096.5 |
| ch01 | 61387907 | 61397045 | 61392476 | PGSC0003DMG401025057 | Inositol-1,4,5-triphosphate-5-phosphatase | 381466 |
| ch01 | 62165076 | 62165746 | 62165411 | PGSC0003DMG400027572 | Gag-pol polyprotein | 391469 |
| ch01 | 61377642 | 61382462 | 61380052 | PGSC0003DMG402025057 | Short-chain dehydrogenase/reductase family protein | 393890 |
| ch01 | 61376309 | 61376722 | 61376515.5 | PGSC0003DMG400037102 | Plant disease resistant protein | 397426.5 |

| | | | | | | |
|---|---|---|---|---|---|---|
| ch01 | 61371320 | 61373660 | 61372490 | PGSC0003DMG400025058 | Interferon-induced GTP-binding protein mx | 401452 |
| ch01 | 62173875 | 62177100 | 62175487.5 | PGSC0003DMG400027542 | Cornichon | 401545.5 |
| ch01 | 62178572 | 62180136 | 62179354 | PGSC0003DMG400027541 | Protein EPIDERMAL PATTERNING FACTOR 2 | 405412 |
| ch01 | 61365638 | 61370163 | 61367900.5 | PGSC0003DMG400025078 | Signal peptidase complex subunit 2 | 406041.5 |
| ch01 | 61341617 | 61343477 | 61342547 | PGSC0003DMG400025059 | Phosphoglycerate mutase | 431395 |
| ch01 | 61283653 | 61287279 | 61285466 | PGSC0003DMG400025081 | Choline/ethanolamine kinase | 488476 |
| ch01 | 62259837 | 62270224 | 62265030.5 | PGSC0003DMG400027536 | Somatic embryogenesis receptor-like kinase 1 | 491088.5 |
| ch01 | 62290487 | 62290957 | 62290722 | PGSC0003DMG400027535 | 40S ribosomal protein S25 | 516780 |
| ch01 | 61242553 | 61244259 | 61243406 | PGSC0003DMG400025063 | Class IV chitinase | 530536 |
| ch01 | 61179401 | 61186016 | 61182708.5 | PGSC0003DMG400025065 | Holocarboxylase synthetase | 591233.5 |
| ch01 | 61153438 | 61155662 | 61154550 | PGSC0003DMG402025066 | Short-chain dehydrogenase/reductase | 619392 |
| ch01 | 62390758 | 62396494 | 62393626 | PGSC0003DMG400027534 | NTGP5 | 619684 |
| ch01 | 62419812 | 62421817 | 62420814.5 | PGSC0003DMG400027532 | 60S ribosomal protein L30 | 646872.5 |
| ch01 | 61122954 | 61125373 | 61124163.5 | PGSC0003DMG400025068 | One-helix protein | 649778.5 |
| ch01 | 62423206 | 62431915 | 62427560.5 | PGSC0003DMG402027531 | SAP domain containing protein | 653618.5 |
| ch01 | 61117538 | 61121018 | 61119278 | PGSC0003DMG400025069 | Cell division protein kinase 7 | 654664 |
| ch01 | 61099775 | 61103157 | 61101466 | PGSC0003DMG402025083 | Peroxidase | 672476 |
| ch01 | 62448469 | 62450355 | 62449412 | PGSC0003DMG400009465 | Aspartic proteinase nepenthesin-1 | 675470 |
| ch01 | 62455552 | 62456793 | 62456172.5 | PGSC0003DMG400009466 | Metal-binding isoprenylated protein | 682230.5 |
| ch01 | 61089724 | 61091577 | 61090650.5 | PGSC0003DMG401025083 | Peroxidase 12 | 683291.5 |
| ch01 | 61081697 | 61083354 | 61082525.5 | PGSC0003DMG400025084 | Peroxidase 4 | 691416.5 |
| ch01 | 61066880 | 61076670 | 61071775 | PGSC0003DMG400025085 | Pyrophosphate-energized membrane proton pump | 702167 |
| ch01 | 62479681 | 62480683 | 62480182 | PGSC0003DMG400009467 | Metal ion binding protein | 706240 |
| ch01 | 61061509 | 61063813 | 61062661 | PGSC0003DMG400025086 | Receptor serine-threonine protein kinase | 711281 |
| ch01 | 61031936 | 61032842 | 61032389 | PGSC0003DMG400025070 | Zinc finger protein | 741553 |
| ch01 | 61010181 | 61011047 | 61010614 | PGSC0003DMG400025071 | P18 protein | 763328 |
| ch01 | 62548111 | 62552557 | 62550334 | PGSC0003DMG400009480 | Sulfate transporter | 776392 |
| ch01 | 60992318 | 60998422 | 60995370 | PGSC0003DMG400025072 | Mutator-like transposase | 778572 |
| ch01 | 62567049 | 62569733 | 62568391 | PGSC0003DMG400009468 | Sulfate transporter 2 | 794449 |
| ch01 | 62571169 | 62578553 | 62574861 | PGSC0003DMG400009470 | Gtpase mss1/trme | 800919 |
| ch01 | 62605489 | 62610064 | 62607776.5 | PGSC0003DMG400009481 | Cellulose synthase | 833834.5 |
| ch01 | 62630354 | 62635755 | 62633054.5 | PGSC0003DMG400009482 | Transporter | 859112.5 |
| ch01 | 62642399 | 62649983 | 62646191 | PGSC0003DMG400009483 | Splicing endonuclease positive effector sen1 | 872249 |
| ch01 | 60879015 | 60880900 | 60879957.5 | PGSC0003DMG400005580 | Cytochrome P450 92B1 | 893984.5 |
| ch01 | 62667701 | 62673256 | 62670478.5 | PGSC0003DMG400009484 | Periplasmic beta-glucosidase | 896536.5 |

| | | | | | | |
|---|---|---|---|---|---|---|
| ch01 | 62724925 | 62729424 | 62727174.5 | PGSC0003DMG400009488 | Late blight resistance protein | 953232.5 |
| ch01 | 62730105 | 62736557 | 62733331 | PGSC0003DMG400009473 | CAK associated cyclinH homolog | 959389 |
| ch01 | 62749585 | 62756970 | 62753277.5 | PGSC0003DMG400009474 | WD-repeat protein | 979335.5 |
| ch01 | 62774550 | 62776590 | 62775570 | PGSC0003DMG400009475 | Dehydration-responsive element binding protein 3 | 1001628 |
| ch01 | 60757490 | 60764951 | 60761220.5 | PGSC0003DMG400006366 | Integrase core domain containing protein | 1012721.5 |
| ch01 | 62803371 | 62805787 | 62804579 | PGSC0003DMG400009476 | Ulp1 protease family, C-terminal catalytic domain containing protein | 1030637 |
| ch01 | 62838516 | 62840884 | 62839700 | PGSC0003DMG400009477 | Trehalose-6-phosphate synthase | 1065758 |
| ch01 | 62913771 | 62916439 | 62915105 | PGSC0003DMG400009478 | F-box protein | 1141163 |
| ch01 | 60610154 | 60612270 | 60611212 | PGSC0003DMG400006369 | AP2/ERF domain-containing transcription factor | 1162730 |
| ch01 | 62952813 | 62956568 | 62954690.5 | PGSC0003DMG400017725 | Vacuolar H+-pyrophosphatase | 1180748.5 |
| ch01 | 62962862 | 62965065 | 62963963.5 | PGSC0003DMG400017715 | Heat shock protein binding protein | 1190021.5 |
| ch01 | 60578099 | 60578491 | 60578295 | PGSC0003DMG400046959 | RING-H2 finger protein ATL32 | 1195647 |
| ch01 | 60573846 | 60577736 | 60575791 | PGSC0003DMG400006371 | Phosphoric diester hydrolase | 1198151 |
| ch01 | 60551213 | 60558696 | 60554954.5 | PGSC0003DMG400006372 | Syntaxin-81 | 1218987.5 |
| ch01 | 60542417 | 60549674 | 60546045.5 | PGSC0003DMG400006362 | COV1 | 1227896.5 |
| ch01 | 63000511 | 63004805 | 63002658 | PGSC0003DMG400017723 | Carbohydrate transporter | 1228716 |
| ch01 | 60538956 | 60542275 | 60540615.5 | PGSC0003DMG400006373 | 5-formyltetrahydrofolate cyclo-ligase family protein | 1233326.5 |
| ch01 | 60526488 | 60529104 | 60527796 | PGSC0003DMG400006364 | Receptor protein kinase | 1246146 |
| ch01 | 60509869 | 60519043 | 60514456 | PGSC0003DMG400006365 | Fimbriae-associated protein Fap1 | 1259486 |
| ch01 | 63034802 | 63039194 | 63036998 | PGSC0003DMG400017721 | APUM7 (Arabidopsis Pumilio 7) | 1263056 |
| ch01 | 63039534 | 63041935 | 63040734.5 | PGSC0003DMG400017714 | Annexin p34 | 1266792.5 |
| ch01 | 63058475 | 63061948 | 63060211.5 | PGSC0003DMG400017713 | LRR receptor-like serine/threonine-protein kinase | 1286269.5 |
| ch01 | 63072775 | 63077256 | 63075015.5 | PGSC0003DMG400017719 | Serine/arginine rich splicing factor | 1301073.5 |
| ch01 | 60454916 | 60455242 | 60455079 | PGSC0003DMG400036667 | Retrotransposon protein | 1318863 |
| ch01 | 63152661 | 63155745 | 63154203 | PGSC0003DMG400017712 | RNA helicase | 1380261 |
| ch01 | 63165449 | 63165940 | 63165694.5 | PGSC0003DMG400043680 | Leucine-rich repeat family protein / protein kinase family protein | 1391752.5 |
| ch01 | 63243827 | 63244737 | 63244282 | PGSC0003DMG400027965 | ORF64c | 1470340 |
| ch01 | 60250479 | 60257545 | 60254012 | PGSC0003DMG400006660 | Cellulose synthase catalytic subunit | 1519930 |
| ch01 | 60221619 | 60222186 | 60221902.5 | PGSC0003DMG400006658 | ECA1 protein | 1552039.5 |
| ch01 | 60205627 | 60207072 | 60206349.5 | PGSC0003DMG400006661 | ASR4 | 1567592.5 |
| ch01 | 60197696 | 60199823 | 60198759.5 | PGSC0003DMG400006662 | Ci21A protein | 1575182.5 |
| ch01 | 60193702 | 60194573 | 60194137.5 | PGSC0003DMG400006663 | ASR3 | 1579804.5 |
| ch01 | 63373149 | 63377238 | 63375193.5 | PGSC0003DMG402006401 | Hexose transporter HT2 | 1601251.5 |
| ch01 | 60138729 | 60139537 | 60139133 | PGSC0003DMG400024093 | Ci21B protein | 1634809 |
| ch01 | 60133288 | 60137988 | 60135638 | PGSC0003DMG400024094 | SET domain protein | 1638304 |

| | | | | | | |
|---|---|---|---|---|---|---|
| ch01 | 60129640 | 60133197 | 60131418.5 | PGSC0003DMG400024095 | Thioredoxin Y1, chloroplastic | 1642523.5 |
| ch01 | 63434502 | 63439658 | 63437080 | PGSC0003DMG400006404 | Carbohydrate esterase | 1663138 |
| ch01 | 60109220 | 60110866 | 60110043 | PGSC0003DMG400024097 | Sterol glucosyltransferase | 1663899 |
| ch01 | 63442002 | 63449194 | 63445598 | PGSC0003DMG400006412 | Ribosome-binding protein | 1671656 |
| ch01 | 63452294 | 63460122 | 63456208 | PGSC0003DMG400006405 | Protein brittle-1, chloroplast | 1682266 |
| ch01 | 63467869 | 63468784 | 63468326.5 | PGSC0003DMG400006413 | Protein trm112 | 1694384.5 |
| ch01 | 63487743 | 63489452 | 63488597.5 | PGSC0003DMG400006408 | Transcription factor MYB102 | 1714655.5 |
| ch01 | 60045801 | 60046965 | 60046383 | PGSC0003DMG400024099 | Heat shock protein | 1727559 |
| ch01 | 63543890 | 63547963 | 63545926.5 | PGSC0003DMG400006410 | Abhydrolase domain containing | 1771984.5 |
| ch01 | 63548091 | 63551999 | 63550045 | PGSC0003DMG400006415 | 14-3-3 protein 7 | 1776103 |
| ch01 | 63558530 | 63562377 | 63560453.5 | PGSC0003DMG400006416 | 5'-adenylylsulfate reductase-like 4 | 1786511.5 |
| ch01 | 63569518 | 63578097 | 63573807.5 | PGSC0003DMG400006417 | Methyl binding domain protein | 1799865.5 |
| ch01 | 59914619 | 59917062 | 59915840.5 | PGSC0003DMG400024106 | Auxin-induced protein 5NG4 | 1858101.5 |
| ch01 | 63656366 | 63659819 | 63658092.5 | PGSC0003DMG400031471 | Leucine-rich repeat family protein | 1884150.5 |
| ch01 | 59881127 | 59884571 | 59882849 | PGSC0003DMG400024107 | GT2 protein | 1891093 |
| ch01 | 63665734 | 63672730 | 63669232 | PGSC0003DMG400031473 | S-adenosylmethionine-dependent methyltransferase | 1895290 |
| ch01 | 59853773 | 59856789 | 59855281 | PGSC0003DMG400024108 | Exocyst complex component | 1918661 |
| ch01 | 59825146 | 59829212 | 59827179 | PGSC0003DMG400024109 | Fructose-1,6-bisphosphatase, cytosolic | 1946763 |
| ch01 | 59819044 | 59824774 | 59821909 | PGSC0003DMG400024110 | Armadillo repeat-containing protein | 1952033 |
| ch01 | 59815932 | 59816707 | 59816319.5 | PGSC0003DMG401024092 | Ubiquitin interaction motif-containing protein | 1957622.5 |
| ch01 | 59793976 | 59802833 | 59798404.5 | PGSC0003DMG400024111 | Cak assembly factor | 1975537.5 |
| ch01 | 59777593 | 59784114 | 59780853.5 | PGSC0003DMG400008617 | Mitochondrial carrier protein | 1993088.5 |
| ch01 | 63767222 | 63769065 | 63768143.5 | PGSC0003DMG400030802 | 40S ribosomal protein S2 | 1994201.5 |

| chr | start | end | mid | ID | Name | hit_dist |
|---|---|---|---|---|---|---|
| ch01 | 70272620 | 70273289 | 70272955 | PGSC0003DMG400025972 | 14 kDa proline-rich protein DC2.15 | 1979357 |
| ch01 | 67222159 | 67224785 | 67223472 | PGSC0003DMG400031113 | 3-hydroxyisobutyrate dehydrogenase | 1070126 |
| ch01 | 69199481 | 69200299 | 69199890 | PGSC0003DMG400042273 | 4,5-DOPA dioxygenase extradiol | 906292 |
| ch01 | 67862982 | 67866574 | 67864778 | PGSC0003DMG400001820 | 5-hydroxyisourate hydrolase | 428820 |
| ch01 | 68412758 | 68416658 | 68414708 | PGSC0003DMG400001685 | Acyl carrier protein | 121110 |
| ch01 | 70015974 | 70017193 | 70016584 | PGSC0003DMG400026025 | AG-motif binding protein-3 | 1722986 |
| ch01 | 67095532 | 67096611 | 67096072 | PGSC0003DMG400031118 | Alcohol dehydrogenase | 1197527 |
| ch01 | 68063361 | 68065025 | 68064193 | PGSC0003DMG400038943 | Amidophosphoribosyltransferase | 229405 |
| ch01 | 67699404 | 67700904 | 67700154 | PGSC0003DMG400001811 | AMP dependent ligase | 593444 |
| ch01 | 68684277 | 68690525 | 68687401 | PGSC0003DMG400001695 | Ankyrin repeat-containing protein | 393803 |
| ch01 | 67206859 | 67209583 | 67208221 | PGSC0003DMG400040554 | Annexin | 1085377 |
| ch01 | 69817898 | 69819217 | 69818558 | PGSC0003DMG400045296 | Anther-specific protein | 1524960 |
| ch01 | 67761505 | 67763389 | 67762447 | PGSC0003DMG400001813 | Anthranilic acid methyltransferase 3 | 531151 |
| ch01 | 69794407 | 69795381 | 69794894 | PGSC0003DMG400026035 | AP2/ERF domain-containing transcription factor | 1501296 |
| ch01 | 67471987 | 67473129 | 67472558 | PGSC0003DMG400001781 | Aspartic proteinase nepenthesin-1 | 821040 |
| ch01 | 67485835 | 67487142 | 67486489 | PGSC0003DMG400043036 | Aspartyl protease family protein | 807109.5 |
| ch01 | 70177223 | 70177747 | 70177485 | PGSC0003DMG400046061 | ATOFP17/OFP17 | 1883887 |
| ch01 | 68436394 | 68440496 | 68438445 | PGSC0003DMG400001718 | ATP binding protein | 144847 |
| ch01 | 67037348 | 67042571 | 67039960 | PGSC0003DMG400028976 | ATP synthase subunit d | 1253639 |
| ch01 | 68399085 | 68402467 | 68400776 | PGSC0003DMG400001715 | ATPUP11 | 107178 |
| ch01 | 69324464 | 69328799 | 69326632 | PGSC0003DMG400026052 | AvrPto-dependent Pto-interacting protein 3 | 1033034 |
| ch01 | 66433339 | 66437362 | 66435351 | PGSC0003DMG400025240 | Axi 1 protein | 1858248 |
| ch01 | 67439347 | 67443109 | 67441228 | PGSC0003DMG400001798 | B3 domain-containing protein Os06g0112300 | 852370 |
| ch01 | 67801439 | 67802863 | 67802151 | PGSC0003DMG400001816 | Benzoate carboxyl methyltransferase | 491447 |
| ch01 | 70112674 | 70117387 | 70115031 | PGSC0003DMG400026022 | Beta expansin 1 | 1821433 |
| ch01 | 66472071 | 66477550 | 66474811 | PGSC0003DMG400025239 | Beta-glucosidase | 1818788 |
| ch01 | 66544367 | 66552432 | 66548400 | PGSC0003DMG400006319 | Beta-glucosidase 01 | 1745199 |
| ch01 | 67135288 | 67137796 | 67136542 | PGSC0003DMG400031128 | Beta-hexosaminidase 1 | 1157056 |
| ch01 | 69331877 | 69340819 | 69336348 | PGSC0003DMG400026051 | Binding protein | 1042750 |
| ch01 | 69344715 | 69350106 | 69347411 | PGSC0003DMG400025994 | BZIP domain class transcription factor | 1053813 |
| ch01 | 66393564 | 66396002 | 66394783 | PGSC0003DMG400036775 | C2 domain-containing protein | 1898815 |
| ch01 | 70137276 | 70138605 | 70137941 | PGSC0003DMG400026020 | C2H2 zinc-finger protein ZPT3-3 | 1844343 |
| ch01 | 67604291 | 67606190 | 67605241 | PGSC0003DMG400001805 | Calcium ion binding protein | 688357.5 |
| ch01 | 69869750 | 69873902 | 69871826 | PGSC0003DMG400026032 | Carotenoid cleavage dioxygenase 7 | 1578228 |

| | | | | | | |
|---|---|---|---|---|---|---|
| ch01 | 66895605 | 66896786 | 66896196 | PGSC0003DMG400028973 | CHP-rich zinc finger protein | 1397403 |
| ch01 | 67227146 | 67233452 | 67230299 | PGSC0003DMG400031112 | Chromo domain protein LHP1 | 1063299 |
| ch01 | 66938299 | 66947643 | 66942971 | PGSC0003DMG400028982 | Citrate synthase | 1350627 |
| ch01 | 66407306 | 66415021 | 66411164 | PGSC0003DMG400025255 | Cohesin subunit rad21 | 1882435 |
| ch01 | 69380916 | 69390403 | 69385660 | PGSC0003DMG400026050 | COP9 signalosome complex subunit 7 | 1092062 |
| ch01 | 68938385 | 68940434 | 68939410 | PGSC0003DMG400031168 | Cyclin-dependent protein kinase | 645811.5 |
| ch01 | 70121232 | 70122734 | 70121983 | PGSC0003DMG400026021 | Cys2/His2 zinc-finger transcription factor | 1828385 |
| ch01 | 69395996 | 69400356 | 69398176 | PGSC0003DMG400025992 | Cysteine-type peptidase | 1104578 |
| ch01 | 68654425 | 68656886 | 68655656 | PGSC0003DMG400001692 | Cytochrome P450 | 362057.5 |
| ch01 | 66880971 | 66882008 | 66881490 | PGSC0003DMG400028970 | DC1 domain containing protein | 1412109 |
| ch01 | 68279098 | 68282671 | 68280885 | PGSC0003DMG400001678 | Dead box ATP-dependent RNA helicase | 12713.5 |
| ch01 | 66628353 | 66633556 | 66630955 | PGSC0003DMG400006314 | DNA binding protein | 1662644 |
| ch01 | 66353830 | 66364552 | 66359191 | PGSC0003DMG400025245 | DnaJ | 1934407 |
| ch01 | 69766024 | 69772325 | 69769175 | PGSC0003DMG400026036 | DnaJ homolog protein | 1475577 |
| ch01 | 68312971 | 68314669 | 68313820 | PGSC0003DMG400001682 | Dynamin | 20222 |
| ch01 | 68450239 | 68451857 | 68451048 | PGSC0003DMG400001719 | E3 ubiquitin-protein ligase ATL4 | 157450 |
| ch01 | 70209330 | 70213905 | 70211618 | PGSC0003DMG400025974 | EF hand family protein | 1918020 |
| ch01 | 69052102 | 69060134 | 69056118 | PGSC0003DMG402031176 | ELMO domain-containing protein | 762520 |
| ch01 | 67876687 | 67879875 | 67878281 | PGSC0003DMG400001797 | Enoyl-CoA-hydratase | 415317 |
| ch01 | 67908155 | 67912635 | 67910395 | PGSC0003DMG400001823 | Ent-kaurenoic acid oxidase | 383203 |
| ch01 | 69392503 | 69393111 | 69392807 | PGSC0003DMG400026049 | ERF1 | 1099209 |
| ch01 | 67854726 | 67855786 | 67855256 | PGSC0003DMG400001796 | ESC | 438342 |
| ch01 | 68509358 | 68510493 | 68509926 | PGSC0003DMG400001688 | Ethphon-induced protein | 216327.5 |
| ch01 | 69601395 | 69602081 | 69601738 | PGSC0003DMG400026046 | Ethylene-responsive transcription factor 13 | 1308140 |
| ch01 | 66342545 | 66350046 | 66346296 | PGSC0003DMG400025257 | Eukaryotic translation initiation factor 3 subunit | 1947303 |
| ch01 | 68067005 | 68072473 | 68069739 | PGSC0003DMG400001674 | Eukaryotic translation initiation factor 3 subunit B | 223859 |
| ch01 | 68539964 | 68548535 | 68544250 | PGSC0003DMG400001722 | Eukaryotic translation initiation factor 4g | 250651.5 |
| ch01 | 66675424 | 66685174 | 66680299 | PGSC0003DMG400006329 | Extensin | 1613299 |
| ch01 | 68989555 | 68993801 | 68991678 | PGSC0003DMG400031165 | F-box/LRR-repeat protein | 698080 |
| ch01 | 68441335 | 68444851 | 68443093 | PGSC0003DMG400001687 | Ferredoxin | 149495 |
| ch01 | 69155411 | 69156957 | 69156184 | PGSC0003DMG400042297 | Flavonol synthase/flavanone 3-hydroxylase | 862586 |
| ch01 | 68980183 | 68982067 | 68981125 | PGSC0003DMG400031173 | Flotillin-1 | 687527 |
| ch01 | 70148568 | 70157759 | 70153164 | PGSC0003DMG400026019 | Gallus gallus similar to zinc finger RNA binding protein (LOC427010), mRNA. | 1859566 |
| ch01 | 67459553 | 67460821 | 67460187 | PGSC0003DMG400044320 | Glucose-6-phosphate/phosphate translocator 1, chloroplast | 833411 |

| | | | | | | |
|---|---|---|---|---|---|---|
| ch01 | 68931018 | 68932658 | 68931838 | PGSC0003DMG400031163 | Glutamyl-tRNA reductase | 638240 |
| ch01 | 67558231 | 67567291 | 67562761 | PGSC0003DMG400001804 | Glutathione s-transferase | 730837 |
| ch01 | 66412488 | 66415021 | 66413755 | PGSC0003DMG400025242 | Glutathione S-transferase T1 | 1879844 |
| ch01 | 67531450 | 67535443 | 67533447 | PGSC0003DMG400001801 | Glutathione S-transferase T3 | 760151.5 |
| ch01 | 67276651 | 67279879 | 67278265 | PGSC0003DMG40003112C | Glycine-rich protein A3 | 1015333 |
| ch01 | 68959031 | 68976708 | 68967870 | PGSC0003DMG402031172 | Glycosyltransferase | 674271.5 |
| ch01 | 70243821 | 70246832 | 70245327 | PGSC0003DMG400025973 | GRAS family transcription factor | 1951729 |
| ch01 | 69699773 | 69702503 | 69701138 | PGSC0003DMG400025984 | HD-ZIP protein | 1407540 |
| ch01 | 69848053 | 69863450 | 69855752 | PGSC0003DMG400025981 | Helicase | 1562154 |
| ch01 | 66470987 | 66471589 | 66471288 | PGSC0003DMG400025253 | Hemolysin-type calcium binding protein | 1822310 |
| ch01 | 66602933 | 66605971 | 66604452 | PGSC0003DMG401006315 | Histone H3.2 | 1689146 |
| ch01 | 66698104 | 66700398 | 66699251 | PGSC0003DMG400006311 | Homeobox-leucine zipper protein HAT14 | 1594347 |
| ch01 | 70177360 | 70181112 | 70179236 | PGSC0003DMG400025975 | Hydrolase, acting on ester bonds | 1885638 |
| ch01 | 67858537 | 67860865 | 67859701 | PGSC0003DMG40000181S | LEA protein Le25 homolog | 433897 |
| ch01 | 67108734 | 67112137 | 67110436 | PGSC0003DMG40003112S | Legume lectin, beta domain | 1183163 |
| ch01 | 69939727 | 69942519 | 69941123 | PGSC0003DMG400026027 | LIGULELESS1 protein | 1647525 |
| ch01 | 68559538 | 68566171 | 68562855 | PGSC0003DMG400001723 | LOL3 (LSD ONE LIKE 3) | 269256.5 |
| ch01 | 67618878 | 67620455 | 67619667 | PGSC0003DMG400001807 | Major latex | 673931.5 |
| ch01 | 69916466 | 69919053 | 69917760 | PGSC0003DMG40002602S | Malate dehydrogenase | 1624162 |
| ch01 | 67269122 | 67275035 | 67272079 | PGSC0003DMG400031121 | Methyltransferase | 1021520 |
| ch01 | 69890544 | 69903140 | 69896842 | PGSC0003DMG40002603C | Mitochondrial elongation factor | 1603244 |
| ch01 | 66951119 | 66959864 | 66955492 | PGSC0003DMG401028974 | Molybdopterin cofactor synthesis protein A | 1338107 |
| ch01 | 69726953 | 69729366 | 69728160 | PGSC0003DMG40002604C | MTERF family protein | 1434562 |
| ch01 | 67189254 | 67202002 | 67195628 | PGSC0003DMG400031124 | Myosin XI-F | 1097970 |
| ch01 | 69072285 | 69073109 | 69072697 | PGSC0003DMG400047167 | NAC transcription factor | 779099 |
| ch01 | 66597399 | 66601144 | 66599272 | PGSC0003DMG40000631E | NBS-LRR type disease resistance protein | 1694327 |
| ch01 | 67142688 | 67143468 | 67143078 | PGSC0003DMG400031125 | Non-specific lipid-transfer protein | 1150520 |
| ch01 | 69673834 | 69679058 | 69676446 | PGSC0003DMG401026043 | NRC1 | 1382848 |
| ch01 | 66666962 | 66673976 | 66670469 | PGSC0003DMG400006312 | N-terminal asparagine amidohydrolase family protein | 1623129 |
| ch01 | 68404213 | 68409952 | 68407083 | PGSC0003DMG40000171E | Nt-rab6 protein | 113484.5 |
| ch01 | 70096672 | 70097905 | 70097289 | PGSC0003DMG400026023 | Nuc-1 negative regulatory protein preg | 1803691 |
| ch01 | 67463466 | 67470772 | 67467119 | PGSC0003DMG40000178C | Nuclear matrix protein 1 | 826479 |
| ch01 | 69200898 | 69206465 | 69203682 | PGSC0003DMG40002599E | Nuclear RNA binding protein | 910083.5 |
| ch01 | 69049967 | 69051659 | 69050813 | PGSC0003DMG40003116E | Nucleoside diphosphate kinase | 757215 |
| ch01 | 70159793 | 70160440 | 70160117 | PGSC0003DMG40102601E | Nucleotidyltransferase | 1866519 |

| | | | | | | |
|---|---|---|---|---|---|---|
| ch01 | 69831351 | 69832652 | 69832002 | PGSC0003DMG402026034 | Oxidoreductase | 1538404 |
| ch01 | 69647628 | 69650467 | 69649048 | PGSC0003DMG401026044 | Palmitoyl-protein thioesterase | 1355450 |
| ch01 | 68429704 | 68436264 | 68432984 | PGSC0003DMG40100168€ | PDE320 | 139386 |
| ch01 | 67179111 | 67182447 | 67180779 | PGSC0003DMG400031114 | Pentatricopeptide | 1112819 |
| ch01 | 68302248 | 68302703 | 68302476 | PGSC0003DMG40100171C | Pentatricopeptide repeat-containing protein | 8877.5 |
| ch01 | 67747968 | 67752039 | 67750004 | PGSC0003DMG400001794 | Pentatricopeptide repeat-containing protein, chloroplastic | 543594.5 |
| ch01 | 67991306 | 67996008 | 67993657 | PGSC0003DMG400001671 | Peptide transporter | 299941 |
| ch01 | 67765345 | 67767189 | 67766267 | PGSC0003DMG400001814 | Peroxisomal 3-hydroxyisobutyryl-coenzyme A hydrolase | 527331 |
| ch01 | 70215605 | 70224102 | 70219854 | PGSC0003DMG400026014 | Phosphatidate cytidylyltransferase | 1926256 |
| ch01 | 68311771 | 68312245 | 68312008 | PGSC0003DMG400001681 | Phosphatidylinositol n-acetylglucosaminyltransferase subunit p | 18410 |
| ch01 | 67536139 | 67543044 | 67539592 | PGSC0003DMG400001802 | Phosphoribosylaminoimidazole-succinocarboxamide synthase | 754006.5 |
| ch01 | 67614957 | 67617034 | 67615996 | PGSC0003DMG40000180€ | Phototropic-responsive NPH3 family protein | 677602.5 |
| ch01 | 68957377 | 68958419 | 68957898 | PGSC0003DMG401031172 | PRA1 family protein F3 | 664300 |
| ch01 | 68622386 | 68630553 | 68626470 | PGSC0003DMG401001724 | Pre-mRNA-splicing factor cwc23 | 332871.5 |
| ch01 | 66899619 | 66901220 | 66900420 | PGSC0003DMG40002898C | PRLI-interacting factor A | 1393179 |
| ch01 | 67587322 | 67589395 | 67588359 | PGSC0003DMG40000178€ | Protein AFR | 705239.5 |
| ch01 | 69711842 | 69719292 | 69715567 | PGSC0003DMG400026041 | Protein BFR2 | 1421969 |
| ch01 | 68092498 | 68096447 | 68094473 | PGSC0003DMG40000170З | Protein binding / zinc ion binding | 199125.5 |
| ch01 | 68641011 | 68645559 | 68643285 | PGSC0003DMG403001724 | Protein kinase Ck2 regulatory subunit 2 | 349687 |
| ch01 | 66571353 | 66577464 | 66574409 | PGSC0003DMG40200633З | Protein kinase family protein | 1719190 |
| ch01 | 67759892 | 67761193 | 67760543 | PGSC0003DMG40000179Ѕ | Protein phosphatase 2c | 533055.5 |
| ch01 | 68567511 | 68569355 | 68568433 | PGSC0003DMG40000169C | Protein synthesis initiation factor | 274835 |
| ch01 | 67656226 | 67658773 | 67657500 | PGSC0003DMG40000180ξ | Protein transport protein sec23 | 636098.5 |
| ch01 | 67996592 | 68005603 | 68001098 | PGSC0003DMG400001672 | Protoheme IX farnesyltransferase | 292500.5 |
| ch01 | 70077080 | 70081427 | 70079254 | PGSC0003DMG40002597€ | Putative bHLH transcription factor | 1785656 |
| ch01 | 69731163 | 69732951 | 69732057 | PGSC0003DMG40102603S | Putative protein | 1438459 |
| ch01 | 69758450 | 69759707 | 69759079 | PGSC0003DMG400026037 | R2r3-myb transcription factor | 1465481 |
| ch01 | 68660665 | 68664195 | 68662430 | PGSC0003DMG40100169З | Rac gtpase | 368832 |
| ch01 | 67962992 | 67970112 | 67966552 | PGSC0003DMG40000167C | Receptor protein kinase | 327046 |
| ch01 | 67651255 | 67655176 | 67653216 | PGSC0003DMG400001792 | Replication factor C subunit | 640382.5 |
| ch01 | 68235639 | 68236447 | 68236043 | PGSC0003DMG400001677 | RING finger | 57555 |
| ch01 | 68463948 | 68470284 | 68467116 | PGSC0003DMG40100172C | Ring finger protein | 173518 |
| ch01 | 68209799 | 68210574 | 68210187 | PGSC0003DMG40000170ξ | RING-H2 finger protein ATL1Q | 83411.5 |
| ch01 | 68183363 | 68183852 | 68183608 | PGSC0003DMG40000170€ | RING-H2 finger protein ATL5A | 109990.5 |
| ch01 | 68676739 | 68682593 | 68679666 | PGSC0003DMG400001694 | RNA binding protein | 386068 |

| | | | | | | |
|---|---|---|---|---|---|---|
| ch01 | 67608823 | 67614620 | 67611722 | PGSC0003DMG40000178 | RNA polymerase | 681876.5 |
| ch01 | 67494948 | 67496902 | 67495925 | PGSC0003DMG40000180 | S-adenosyl-L-methionine:nicotinic acid carboxyl methyltransferase | 797673 |
| ch01 | 66427545 | 66431765 | 66429655 | PGSC0003DMG400025254 | S-adenosyl-L-methionine-dependent uroporphyrinogen III methyltransferase | 1863943 |
| ch01 | 66293455 | 66296235 | 66294845 | PGSC0003DMG40002524 | Serine hydroxymethyltransferase | 1998753 |
| ch01 | 67546901 | 67550739 | 67548820 | PGSC0003DMG40000178 | Serine/threonine-protein kinase PEPKR2 | 744778 |
| ch01 | 67081206 | 67090020 | 67085613 | PGSC0003DMG40003113 | Serine/threonine-protein phosphatase PP2A-5 catalytic subunit | 1207985 |
| ch01 | 67245732 | 67251341 | 67248537 | PGSC0003DMG40003111 | SIGB (SIGMA FACTOR B) | 1045062 |
| ch01 | 69143165 | 69147315 | 69145240 | PGSC0003DMG40003758 | SRG1 | 851642 |
| ch01 | 68168410 | 68171996 | 68170203 | PGSC0003DMG40000167 | Sterol desaturase | 123395 |
| ch01 | 67289511 | 67294681 | 67292096 | PGSC0003DMG40003111 | Stress-induced protein | 1001502 |
| ch01 | 68291453 | 68295376 | 68293415 | PGSC0003DMG40000168 | Structural constituent of ribosome | 183.5 |
| ch01 | 68419618 | 68420311 | 68419965 | PGSC0003DMG40000171 | Sts15 protein | 126366.5 |
| ch01 | 66371104 | 66374451 | 66372778 | PGSC0003DMG40002525 | Synaptotagmin | 1920821 |
| ch01 | 69998302 | 70008263 | 70003283 | PGSC0003DMG40002602 | T-complex protein 1 subunit alpha | 1709685 |
| ch01 | 68691922 | 68694120 | 68693021 | PGSC0003DMG40000169 | Transcription factor | 399423 |
| ch01 | 67646181 | 67648267 | 67647224 | PGSC0003DMG400036434 | Transcription factor ICE1 | 646374 |
| ch01 | 66901701 | 66905176 | 66903439 | PGSC0003DMG400028981 | Transferase, transferring glycosyl groups | 1390160 |
| ch01 | 68472784 | 68477551 | 68475168 | PGSC0003DMG40200172 | Transferase, transferring glycosyl groups / transferase, transferring hexosyl groups | 181569.5 |
| ch01 | 69257909 | 69259559 | 69258734 | PGSC0003DMG40002599 | Triacylglycerol lipase | 965136 |
| ch01 | 67259521 | 67268311 | 67263916 | PGSC0003DMG40003112 | Ubiquinone/menaquinone biosynthesis methyltransferase | 1029682 |
| ch01 | 69016768 | 69019388 | 69018078 | PGSC0003DMG400031181 | Ubiquitin-protein ligase | 724480 |
| ch01 | 69130572 | 69132107 | 69131340 | PGSC0003DMG400026001 | Uclacyanin-2 | 837741.5 |
| ch01 | 67694571 | 67696316 | 67695444 | PGSC0003DMG40004521 | Ulp1 protease family, C-terminal catalytic domain containing protein | 598154.5 |
| ch01 | 68293297 | 68298692 | 68295995 | PGSC0003DMG40000170 | Uridylate kinase | 2396.5 |
| ch01 | 67210726 | 67220039 | 67215383 | PGSC0003DMG40103112 | Utp-glucose-1-phosphate uridylyltransferase | 1078216 |
| ch01 | 70202895 | 70210238 | 70206567 | PGSC0003DMG40002601 | VAP27 | 1912969 |
| ch01 | 70282424 | 70286582 | 70284503 | PGSC0003DMG402025971 | Vesicle-associated membrane protein | 1990905 |
| ch01 | 66962853 | 66963320 | 66963087 | PGSC0003DMG40002898 | VQ motif-containing protein | 1330512 |
| ch01 | 70183000 | 70185203 | 70184102 | PGSC0003DMG40002601 | Xenotropic and polytropic murine leukemia virus receptor | 1890504 |
| ch01 | 67682760 | 67686262 | 67684511 | PGSC0003DMG40000180 | Xyloglucan endotransglucosylase-hydrolase XTH5 | 609087 |
| ch01 | 69952819 | 69954589 | 69953704 | PGSC0003DMG40002597 | Zinc ion binding protein | 1660106 |
| ch01 | 69182059 | 69184515 | 69183287 | PGSC0003DMG40003910 | Zinc knuckle family protein | 889689 |

| chr | start | end | mid | ID | Name | hit_dist |
|---|---|---|---|---|---|---|
| ch01 | 70096672 | 70097905 | 70097289 | PGSC0003DMG400026023 | Nuc-1 negative regulatory protein preg | 227.5 |
| ch01 | 70112674 | 70117387 | 70115031 | PGSC0003DMG400026022 | Beta expansin 1 | 17514.5 |
| ch01 | 70077080 | 70081427 | 70079254 | PGSC0003DMG400025976 | Putative bHLH transcription factor | 18262.5 |
| ch01 | 70121232 | 70122734 | 70121983 | PGSC0003DMG400026021 | Cys2/His2 zinc-finger transcription factor | 24467 |
| ch01 | 70137276 | 70138605 | 70137941 | PGSC0003DMG400026020 | C2H2 zinc-finger protein ZPT3-3 | 40424.5 |
| ch01 | 70148568 | 70157759 | 70153164 | PGSC0003DMG400026019 | Gallus gallus similar to zinc finger RNA binding protein (LOC427010), mRNA. | 55647.5 |
| ch01 | 70159793 | 70160440 | 70160117 | PGSC0003DMG401026018 | Nucleotidyltransferase | 62600.5 |
| ch01 | 70177223 | 70177747 | 70177485 | PGSC0003DMG400046061 | ATOFP17/OFP17 | 79969 |
| ch01 | 70015974 | 70017193 | 70016584 | PGSC0003DMG400026025 | AG-motif binding protein-3 | 80932.5 |
| ch01 | 70177360 | 70181112 | 70179236 | PGSC0003DMG400025975 | Hydrolase, acting on ester bonds | 81720 |
| ch01 | 70183000 | 70185203 | 70184102 | PGSC0003DMG400026017 | Xenotropic and polytropic murine leukemia virus receptor | 86585.5 |
| ch01 | 69998302 | 70008263 | 70003283 | PGSC0003DMG400026026 | T-complex protein 1 subunit alpha | 94233.5 |
| ch01 | 70202895 | 70210238 | 70206567 | PGSC0003DMG400026015 | VAP27 | 109050.5 |
| ch01 | 70209330 | 70213905 | 70211618 | PGSC0003DMG400025974 | EF hand family protein | 114101.5 |
| ch01 | 70215605 | 70224102 | 70219854 | PGSC0003DMG400026014 | Phosphatidate cytidylyltransferase | 122337.5 |
| ch01 | 69952819 | 69954589 | 69953704 | PGSC0003DMG400025978 | Zinc ion binding protein | 143812 |
| ch01 | 70243821 | 70246832 | 70245327 | PGSC0003DMG400025973 | GRAS family transcription factor | 147810.5 |
| ch01 | 69939727 | 69942519 | 69941123 | PGSC0003DMG400026027 | LIGULELESS1 protein | 156393 |
| ch01 | 70272620 | 70273289 | 70272955 | PGSC0003DMG400025972 | 14 kDa proline-rich protein DC2.15 | 175438.5 |
| ch01 | 69916466 | 69919053 | 69917760 | PGSC0003DMG400026029 | Malate dehydrogenase | 179756.5 |
| ch01 | 70282424 | 70286582 | 70284503 | PGSC0003DMG402025971 | Vesicle-associated membrane protein | 186987 |
| ch01 | 69890544 | 69903140 | 69896842 | PGSC0003DMG400026030 | Mitochondrial elongation factor | 200674 |
| ch01 | 69869750 | 69873902 | 69871826 | PGSC0003DMG400026032 | Carotenoid cleavage dioxygenase 7 | 225690 |
| ch01 | 70321879 | 70326102 | 70323991 | PGSC0003DMG400025969 | YABBY1 | 226474.5 |
| ch01 | 69848053 | 69863450 | 69855752 | PGSC0003DMG400025981 | Helicase | 241764.5 |
| ch01 | 70335685 | 70343801 | 70339743 | PGSC0003DMG400025968 | Golgi SNARE 12 protein | 242227 |
| ch01 | 69846381 | 69848331 | 69847356 | PGSC0003DMG400026033 | Oxidoreductase | 250160 |
| ch01 | 70355500 | 70356465 | 70355983 | PGSC0003DMG400026010 | Auxin-induced protein X10A | 258466.5 |
| ch01 | 70365852 | 70368134 | 70366993 | PGSC0003DMG400026009 | Kinase | 269477 |
| ch01 | 70371571 | 70374619 | 70373095 | PGSC0003DMG400025967 | Pectinesterase | 275579 |
| ch01 | 69817898 | 69819217 | 69818558 | PGSC0003DMG400045296 | Anther-specific protein | 278958.5 |
| ch01 | 69794407 | 69795381 | 69794894 | PGSC0003DMG400026035 | AP2/ERF domain-containing transcription factor | 302622 |

| | | | | | | |
|---|---|---|---|---|---|---|
| ch01 | 70397117 | 70407312 | 70402215 | PGSC0003DMG40002600‌8 | Methionine aminopeptidase | 304698.5 |
| ch01 | 70408201 | 70417727 | 70412964 | PGSC0003DMG40002596‌6 | Beta-tubulin cofactor d | 315448 |
| ch01 | 69766024 | 69772325 | 69769175 | PGSC0003DMG40002603‌6 | DnaJ homolog protein | 328341.5 |
| ch01 | 70430424 | 70435743 | 70433084 | PGSC0003DMG400025964 | Ulp1 protease family, C-terminal catalytic domain containing protein | 335567.5 |
| ch01 | 69758450 | 69759707 | 69759079 | PGSC0003DMG400026037 | R2r3-myb transcription factor | 338437.5 |
| ch01 | 69735179 | 69738867 | 69737023 | PGSC0003DMG400026038 | Pentatricopeptide repeat-containing protein | 360493 |
| ch01 | 69731163 | 69732951 | 69732057 | PGSC0003DMG401026039 | Putative protein | 365459 |
| ch01 | 69726953 | 69729366 | 69728160 | PGSC0003DMG400026040 | MTERF family protein | 369356.5 |
| ch01 | 69711842 | 69719292 | 69715567 | PGSC0003DMG400026041 | Protein BFR2 | 381949 |
| ch01 | 69699773 | 69702503 | 69701138 | PGSC0003DMG400025984 | HD-ZIP protein | 396378 |
| ch01 | 70501630 | 70503321 | 70502476 | PGSC0003DMG400025961 | Proton-dependent oligopeptide transport family protein | 404959.5 |
| ch01 | 69682241 | 69687450 | 69684846 | PGSC0003DMG404026043 | NRC1 | 412670.5 |
| ch01 | 70510274 | 70516740 | 70513507 | PGSC0003DMG400026006 | 3-phosphoshikimate 1-carboxyvinyltransferase, chloroplastic | 415991 |
| ch01 | 70524032 | 70526177 | 70525105 | PGSC0003DMG400026005 | UDP-glucuronate 4-epimerase 2 | 427588.5 |
| ch01 | 70535571 | 70537827 | 70536699 | PGSC0003DMG401025960 | Cytoplasmic ribosomal protein S13 | 439183 |
| ch01 | 69647628 | 69650467 | 69649048 | PGSC0003DMG401026044 | Palmitoyl-protein thioesterase | 448468.5 |
| ch01 | 70544467 | 70547797 | 70546132 | PGSC0003DMG400026004 | Leucine-rich repeat receptor kinase | 448616 |
| ch01 | 70549160 | 70551466 | 70550313 | PGSC0003DMG400025959 | Pentatricopeptide repeat-containing protein, chloroplastic | 452797 |
| ch01 | 70559765 | 70563379 | 70561572 | PGSC0003DMG401025958 | Glycine-rich protein | 464056 |
| ch01 | 69625546 | 69626202 | 69625874 | PGSC0003DMG400040573 | ERF1 | 471642 |
| ch01 | 70567670 | 70574819 | 70571245 | PGSC0003DMG400025956 | Zinc finger protein | 473728.5 |
| ch01 | 69601395 | 69602081 | 69601738 | PGSC0003DMG400026046 | Ethylene-responsive transcription factor 13 | 495778 |
| ch01 | 69566214 | 69566992 | 69566603 | PGSC0003DMG400025987 | Non-specific lipid-transfer protein | 530913 |
| ch01 | 70632834 | 70639345 | 70636090 | PGSC0003DMG400007771 | Protein transport Sec1a | 538573.5 |
| ch01 | 70693695 | 70702211 | 70697953 | PGSC0003DMG400012776 | Nucleotide binding protein | 600437 |
| ch01 | 70705183 | 70708003 | 70706593 | PGSC0003DMG400012763 | C-4 sterol methyl oxidase | 609077 |
| ch01 | 70720425 | 70728217 | 70724321 | PGSC0003DMG400012762 | Glutathione-s-transferase theta, gst | 626805 |
| ch01 | 70729284 | 70737764 | 70733524 | PGSC0003DMG400012775 | Caldesmon | 636008 |
| ch01 | 70737961 | 70744453 | 70741207 | PGSC0003DMG400012761 | Ku P80 DNA helicase | 643691 |
| ch01 | 70749317 | 70751290 | 70750304 | PGSC0003DMG401012773 | Cornichon family protein | 652787.5 |
| ch01 | 70751585 | 70756876 | 70754231 | PGSC0003DMG400012760 | DNA binding protein | 656714.5 |
| ch01 | 70769929 | 70775575 | 70772752 | PGSC0003DMG400012759 | Protein regulator of cytokinesis | 675236 |
| ch01 | 70793623 | 70795762 | 70794693 | PGSC0003DMG400012757 | LOB domain-containing protein | 697176.5 |
| ch01 | 69395996 | 69400356 | 69398176 | PGSC0003DMG400025992 | Cysteine-type peptidase | 699340 |

| | | | | | | |
|---|---|---|---|---|---|---|
| ch01 | 70798350 | 70800247 | 70799299 | PGSC0003DMG40001275€ | Gibberellin receptor GID1 | 701782.5 |
| ch01 | 69380916 | 69390403 | 69385660 | PGSC0003DMG40002605C | COP9 signalosome complex subunit 7 | 711856.5 |
| ch01 | 70810690 | 70811236 | 70810963 | PGSC0003DMG40201277Z | LOB domain protein 18 | 713447 |
| ch01 | 70827468 | 70842750 | 70835109 | PGSC0003DMG400012771 | Cytohesin 1, 2, 3 | 737593 |
| ch01 | 70844121 | 70845424 | 70844773 | PGSC0003DMG40001277C | Calmodulin | 747256.5 |
| ch01 | 69344715 | 69350106 | 69347411 | PGSC0003DMG400025994 | BZIP domain class transcription factor | 750105.5 |
| ch01 | 69331877 | 69340819 | 69336348 | PGSC0003DMG400026051 | Binding protein | 761168 |
| ch01 | 70865903 | 70867479 | 70866691 | PGSC0003DMG40001276§ | ESC | 769175 |
| ch01 | 69324464 | 69328799 | 69326632 | PGSC0003DMG400026052 | AvrPto-dependent Pto-interacting protein 3 | 770884.5 |
| ch01 | 70892050 | 70901244 | 70896647 | PGSC0003DMG400012752 | Forkhead-associated domain-containing protein | 799131 |
| ch01 | 70903029 | 70903840 | 70903435 | PGSC0003DMG400012751 | Acyltransferase | 805918.5 |
| ch01 | 70921191 | 70922159 | 70921675 | PGSC0003DMG40001276٤ | Carnitine racemase | 824159 |
| ch01 | 70926465 | 70928437 | 70927451 | PGSC0003DMG40001274§ | Fasciclin-like arabinogalactan protein 13 | 829935 |
| ch01 | 69261522 | 69269505 | 69265514 | PGSC0003DMG40002599€ | RING finger protein | 832002.5 |
| ch01 | 69257909 | 69259559 | 69258734 | PGSC0003DMG400025997 | Triacylglycerol lipase | 838782 |
| ch01 | 69255614 | 69257775 | 69256695 | PGSC0003DMG400026054 | ATP binding protein | 840821.5 |
| ch01 | 70939165 | 70942478 | 70940822 | PGSC0003DMG400012767 | Growth-regulating factor 10 | 843305.5 |
| ch01 | 70988168 | 70992188 | 70990178 | PGSC0003DMG400027784 | Acyl-CoA thioesterase | 892662 |
| ch01 | 69200898 | 69206465 | 69203682 | PGSC0003DMG40002599٤ | Nuclear RNA binding protein | 893834.5 |
| ch01 | 69199481 | 69200299 | 69199890 | PGSC0003DMG400042273 | 4,5-DOPA dioxygenase extradiol | 897626 |
| ch01 | 70996061 | 71003680 | 70999871 | PGSC0003DMG400027773 | Cell division protein ftsy | 902354.5 |
| ch01 | 69182059 | 69184515 | 69183287 | PGSC0003DMG400039102 | Zinc knuckle family protein | 914229 |
| ch01 | 71011362 | 71016936 | 71014149 | PGSC0003DMG400027772 | Cleavage and polyadenylation specificity factor | 916633 |
| ch01 | 69171420 | 69173348 | 69172384 | PGSC0003DMG400026057 | Flavonol synthase/flavanone 3-hydroxylase | 925132 |
| ch01 | 71035288 | 71039572 | 71037430 | PGSC0003DMG400027771 | Shaggy-like kinase 6 (Nsk6) | 939914 |
| ch01 | 69143165 | 69147315 | 69145240 | PGSC0003DMG400037587 | SRG1 | 952276 |
| ch01 | 69130572 | 69132107 | 69131340 | PGSC0003DMG400026001 | Uclacyanin-2 | 966176.5 |
| ch01 | 71062191 | 71068244 | 71065218 | PGSC0003DMG40002777C | Cutin deficient 2 | 967701.5 |
| ch01 | 71076166 | 71083933 | 71080050 | PGSC0003DMG40002776§ | Methyltransferase PMT13 | 982533.5 |
| ch01 | 71084833 | 71091174 | 71088004 | PGSC0003DMG40002776٤ | Cop11 protein | 990487.5 |
| ch01 | 71113182 | 71114219 | 71113701 | PGSC0003DMG40002777§ | Flavonol 3-sulfotransferase | 1016184.5 |
| ch01 | 69072285 | 69073109 | 69072697 | PGSC0003DMG400047167 | NAC transcription factor | 1024819 |
| ch01 | 71127573 | 71128606 | 71128090 | PGSC0003DMG400027767 | Alcohol dehydrogenase | 1030573.5 |
| ch01 | 71132925 | 71134313 | 71133619 | PGSC0003DMG40002777٤ | Progesterone 5beta reductase-A | 1036103 |

| | | | | | | |
|---|---|---|---|---|---|---|
| ch01 | 69052102 | 69060134 | 69056118 | PGSC0003DMG40203117€ | ELMO domain-containing protein | 1041398 |
| ch01 | 69049967 | 69051659 | 69050813 | PGSC0003DMG40003116€ | Nucleoside diphosphate kinase | 1046703 |
| ch01 | 71142591 | 71147054 | 71144823 | PGSC0003DMG40002776€ | Generic methyltransferase | 1047306.5 |
| ch01 | 71161448 | 71163580 | 71162514 | PGSC0003DMG40002777 | F-box family protein | 1064998 |
| ch01 | 69021571 | 69027216 | 69024394 | PGSC0003DMG40003117 | Transcription factor | 1073122.5 |
| ch01 | 69016768 | 69019388 | 69018078 | PGSC0003DMG400031181 | Ubiquitin-protein ligase | 1079438 |
| ch01 | 71186017 | 71189886 | 71187952 | PGSC0003DMG40002776 | Peroxisomal biogenesis factor | 1090435.5 |
| ch01 | 68989555 | 68993801 | 68991678 | PGSC0003DMG40003116 | F-box/LRR-repeat protein | 1105838 |
| ch01 | 71203580 | 71208336 | 71205958 | PGSC0003DMG40001705 | Splicing factor | 1108442 |
| ch01 | 68980183 | 68982067 | 68981125 | PGSC0003DMG40003117 | Flotillin-1 | 1116391 |
| ch01 | 71220383 | 71227844 | 71224114 | PGSC0003DMG40001705 | Fructosamine kinase family protein | 1126597.5 |
| ch01 | 68959031 | 68976708 | 68967870 | PGSC0003DMG40203117 | Glycosyltransferase | 1129646.5 |
| ch01 | 71229477 | 71234944 | 71232211 | PGSC0003DMG40001705C | Dimethyladenosine transferase | 1134694.5 |
| ch01 | 71235278 | 71237736 | 71236507 | PGSC0003DMG40003857S | Two-component system sensor histidine kinase/response regulator | 1138991 |
| ch01 | 68957377 | 68958419 | 68957898 | PGSC0003DMG40103117 | PRA1 family protein F3 | 1139618 |
| ch01 | 68938385 | 68940434 | 68939410 | PGSC0003DMG40003116 | Cyclin-dependent protein kinase | 1158106.5 |
| ch01 | 68933326 | 68934798 | 68934062 | PGSC0003DMG40003116 | Ankyrin repeat-containing protein | 1163454 |
| ch01 | 68931018 | 68932658 | 68931838 | PGSC0003DMG40003116 | Glutamyl-tRNA reductase | 1165678 |
| ch01 | 71266068 | 71266811 | 71266440 | PGSC0003DMG400000251 | Adenosine 3'-phospho 5'-phosphosulfate transporter | 1168923.5 |
| ch01 | 71271111 | 71273034 | 71272073 | PGSC0003DMG400000002 | Vacuolar iron family transporter | 1174556.5 |
| ch01 | 71271149 | 71279706 | 71275428 | PGSC0003DMG400000131 | SET domain-containing protein | 1177911.5 |
| ch01 | 71280779 | 71281974 | 71281377 | PGSC0003DMG400000252 | KDEL motif-containing protein 1 | 1183860.5 |
| ch01 | 71293234 | 71298560 | 71295897 | PGSC0003DMG400000003 | SPX domain-containing membrane protein | 1198381 |
| ch01 | 71299563 | 71304982 | 71302273 | PGSC0003DMG400000004 | Arsenical pump-driving atpase | 1204756.5 |
| ch01 | 71314568 | 71319702 | 71317135 | PGSC0003DMG400000005 | Shrunken seed protein | 1219619 |
| ch01 | 71322006 | 71322848 | 71322427 | PGSC0003DMG400045197 | Transcription factor TCP9 | 1224911 |
| ch01 | 71321742 | 71324760 | 71323251 | PGSC0003DMG40000013 | Phospholipase PLDb2 | 1225735 |
| ch01 | 71337837 | 71340591 | 71339214 | PGSC0003DMG400000134 | Xylem serine proteinase 1 | 1241698 |
| ch01 | 71360200 | 71361420 | 71360810 | PGSC0003DMG401000007 | 1-acylglycerophosphocholine O-acyltransferase | 1263294 |
| ch01 | 71369799 | 71373015 | 71371407 | PGSC0003DMG402000007 | JD1 | 1273891 |
| ch01 | 71421222 | 71426490 | 71423856 | PGSC0003DMG40000013€ | PMADS4 protein | 1326340 |
| ch01 | 71463509 | 71465702 | 71464606 | PGSC0003DMG400000011 | Gibberellin 20-oxidase 4 | 1367089.5 |
| ch01 | 68676739 | 68682593 | 68679666 | PGSC0003DMG400001694 | RNA binding protein | 1417850 |
| ch01 | 68660665 | 68664195 | 68662430 | PGSC0003DMG401001693 | Rac gtpase | 1435086 |

| | | | | | | |
|---|---|---|---|---|---|---|
| ch01 | 68654425 | 68656886 | 68655656 | PGSC0003DMG400001692 | Cytochrome P450 | 1441860.5 |
| ch01 | 68641011 | 68645559 | 68643285 | PGSC0003DMG403001724 | Protein kinase Ck2 regulatory subunit 2 | 1454231 |
| ch01 | 71551698 | 71554110 | 71552904 | PGSC0003DMG400000142 | Copper ion binding protein | 1455388 |
| ch01 | 68622386 | 68630553 | 68626470 | PGSC0003DMG401001724 | Pre-mRNA-splicing factor cwc23 | 1471046.5 |
| ch01 | 71622323 | 71624371 | 71623347 | PGSC0003DMG400000145 | Cytochrome P450 monooxygenase | 1525831 |
| ch01 | 68567511 | 68569355 | 68568433 | PGSC0003DMG40000169C | Protein synthesis initiation factor | 1529083 |
| ch01 | 71626524 | 71628209 | 71627367 | PGSC0003DMG400000013 | Globulin | 1529850.5 |
| ch01 | 68559538 | 68566171 | 68562855 | PGSC0003DMG400001723 | LOL3 (LSD ONE LIKE 3) | 1534661.5 |
| ch01 | 71644624 | 71648773 | 71646699 | PGSC0003DMG400000014 | Enoyl-CoA-hydratase | 1549182.5 |
| ch01 | 68539964 | 68548535 | 68544250 | PGSC0003DMG400001722 | Eukaryotic translation initiation factor 4g | 1553266.5 |
| ch01 | 68509358 | 68510493 | 68509926 | PGSC0003DMG400001688 | Ethphon-induced protein | 1587590.5 |
| ch01 | 71688539 | 71694700 | 71691620 | PGSC0003DMG400000017 | Beta-hydroxyisobutyryl-CoA hydrolase 1 | 1594103.5 |
| ch01 | 71712906 | 71715353 | 71714130 | PGSC0003DMG401000018 | Mosaic virus helicase domain-binding protein | 1616613.5 |
| ch01 | 68472784 | 68477551 | 68475168 | PGSC0003DMG40200172C | Transferase, transferring glycosyl groups / transferase, transferring hexosyl groups | 1622348.5 |
| ch01 | 68450239 | 68451857 | 68451048 | PGSC0003DMG400001719 | E3 ubiquitin-protein ligase ATL4 | 1646468 |
| ch01 | 68441335 | 68444851 | 68443093 | PGSC0003DMG400001687 | Ferredoxin | 1654423 |
| ch01 | 68429704 | 68436264 | 68432984 | PGSC0003DMG401001686 | PDE320 | 1664532 |
| ch01 | 68422373 | 68423026 | 68422700 | PGSC0003DMG400001728 | Sts15 protein | 1674816.5 |
| ch01 | 68412758 | 68416658 | 68414708 | PGSC0003DMG400001685 | Acyl carrier protein | 1682808 |
| ch01 | 71779104 | 71784498 | 71781801 | PGSC0003DMG400000151 | Digalactosyldiacylglycerol synthase 2, chloroplastic | 1684285 |
| ch01 | 68404213 | 68409952 | 68407083 | PGSC0003DMG400001716 | Nt-rab6 protein | 1690433.5 |
| ch01 | 68399085 | 68402467 | 68400776 | PGSC0003DMG400001715 | ATPUP11 | 1696740 |
| ch01 | 71792375 | 71797530 | 71794953 | PGSC0003DMG402000019 | Dtdp-4-dehydrorhamnose dehydrogenase | 1697436.5 |
| ch01 | 71807804 | 71817295 | 71812550 | PGSC0003DMG40100002C | NAD-dependent malic enzyme 59 kDa isoform, mitochondrial | 1715033.5 |
| ch01 | 68350161 | 68358169 | 68354165 | PGSC0003DMG400001714 | Dynamin | 1743351 |
| ch01 | 71841960 | 71845603 | 71843782 | PGSC0003DMG400000021 | Carbonyl reductase | 1746265.5 |
| ch01 | 68348103 | 68350059 | 68349081 | PGSC0003DMG400001683 | Phosphatidylinositol n-acetylglucosaminyltransferase subunit p | 1748435 |
| ch01 | 71847600 | 71847956 | 71847778 | PGSC0003DMG40003823C | (+)-neomenthol dehydrogenase | 1750262 |
| ch01 | 71862638 | 71866307 | 71864473 | PGSC0003DMG400000152 | Methylenetetrahydrofolate dehydrogenase | 1766956.5 |
| ch01 | 71866659 | 71868287 | 71867473 | PGSC0003DMG400000153 | KEA1 (K EFFLUX ANTIPORTER 1) | 1769957 |
| ch01 | 71887333 | 71888703 | 71888018 | PGSC0003DMG400000023 | Pre-mRNA processing ribonucleoprotein, binding region | 1790502 |
| ch01 | 68293297 | 68298692 | 68295995 | PGSC0003DMG400001709 | Uridylate kinase | 1801521.5 |
| ch01 | 71900075 | 71903042 | 71901559 | PGSC0003DMG400000155 | LIM domain protein PLIM-2 | 1804042.5 |

| | | | | | | |
|---|---|---|---|---|---|---|
| ch01 | 68291453 | 68295376 | 68293415 | PGSC0003DMG40000168C | Structural constituent of ribosome | 1804101.5 |
| ch01 | 68279098 | 68282671 | 68280885 | PGSC0003DMG400001678 | Dead box ATP-dependent RNA helicase | 1816631.5 |
| ch01 | 71916272 | 71921680 | 71918976 | PGSC0003DMG400000025 | Acetyl-coenzyme A carboxylase carboxyl transferase alpha | 1821460 |
| ch01 | 71942359 | 71946082 | 71944221 | PGSC0003DMG400000157 | Remorin 2 | 1846704.5 |
| ch01 | 68235639 | 68236447 | 68236043 | PGSC0003DMG400001677 | RING finger | 1861473 |
| ch01 | 71963933 | 71965552 | 71964743 | PGSC0003DMG400000158 | Actin-depolymerizing factor 12 | 1867226.5 |
| ch01 | 71971630 | 71978556 | 71975093 | PGSC0003DMG401000029 | Synaptotagmin | 1877577 |
| ch01 | 68209799 | 68210574 | 68210187 | PGSC0003DMG400001708 | RING-H2 finger protein ATL1Q | 1887329.5 |
| ch01 | 71990951 | 71991520 | 71991236 | PGSC0003DMG400000034 | Kiwellin | 1893719.5 |
| ch01 | 72001885 | 72002610 | 72002248 | PGSC0003DMG400039861 | Translation initiation factor | 1904731.5 |
| ch01 | 72011016 | 72011580 | 72011298 | PGSC0003DMG400000259 | Transposase | 1913782 |
| ch01 | 68183363 | 68183852 | 68183608 | PGSC0003DMG400001706 | RING-H2 finger protein ATL5A | 1913908.5 |
| ch01 | 72019764 | 72024183 | 72021974 | PGSC0003DMG400000162 | Ccr4-not transcription complex | 1924457.5 |
| ch01 | 68168410 | 68171996 | 68170203 | PGSC0003DMG400001676 | Sterol desaturase | 1927313 |
| ch01 | 72050665 | 72053201 | 72051933 | PGSC0003DMG400000164 | Cytidine/deoxycytidylate deaminase family protein | 1954417 |
| ch01 | 72056268 | 72059559 | 72057914 | PGSC0003DMG400000165 | Protein AFR | 1960397.5 |
| ch01 | 72070531 | 72073869 | 72072200 | PGSC0003DMG400000167 | Small nuclear ribonucleoprotein-associated protein | 1974684 |
| ch01 | 72094678 | 72097321 | 72096000 | PGSC0003DMG400000168 | 60S ribosomal protein L36 | 1998483.5 |

| chr | start | end | mid | ID | Name | hit_dist |
|---|---|---|---|---|---|---|
| ch01 | 86309556 | 86315959 | 86312758 | PGSC0003DMG400030960 | 26S proteasome AAA-ATPase subunit RPT4a | 1576555.5 |
| ch01 | 87659543 | 87664185 | 87661864 | PGSC0003DMG401006167 | 26S proteasome non-ATPase regulatory subunit 3 | 227449 |
| ch01 | 87655461 | 87658447 | 87656954 | PGSC0003DMG402006167 | 26S proteasome regulatory subunit S3 | 232359 |
| ch01 | 85919727 | 85922285 | 85921006 | PGSC0003DMG400000725 | 2-hydroxyacid dehydrogenase | 1968307 |
| ch01 | 87128757 | 87132021 | 87130389 | PGSC0003DMG400001638 | 39 kDa EF-Hand containing protein | 758924 |
| ch01 | 87351050 | 87352477 | 87351764 | PGSC0003DMG401006199 | Actin depolymerizing factor 5 | 537549.5 |
| ch01 | 87932556 | 87935363 | 87933960 | PGSC0003DMG400018652 | Activator of basal transcription | 44646.5 |
| ch01 | 85896082 | 85900169 | 85898126 | PGSC0003DMG400000723 | Adenylyl cyclase-associated protein | 1991187.5 |
| ch01 | 86628053 | 86629634 | 86628844 | PGSC0003DMG400003126 | AG-motif binding protein-4 | 1260469.5 |
| ch01 | 86850534 | 86854153 | 86852344 | PGSC0003DMG400001661 | Aldo-keto reductase | 1036969.5 |
| ch01 | 87240584 | 87242941 | 87241763 | PGSC0003DMG400001625 | Amidase | 647550.5 |
| ch01 | 86902852 | 86905000 | 86903926 | PGSC0003DMG400001618 | Amidophosphoribosyltransferase | 985387 |
| ch01 | 87962706 | 87965704 | 87964205 | PGSC0003DMG400018651 | Amino acid transporter | 74892 |
| ch01 | 85942494 | 85946070 | 85944282 | PGSC0003DMG400000707 | AP-3 complex subunit sigma | 1945031 |
| ch01 | 87631164 | 87632633 | 87631899 | PGSC0003DMG400006183 | Aquaporin | 257414.5 |
| ch01 | 87104708 | 87105454 | 87105081 | PGSC0003DMG400001640 | Aquaporin, MIP family, PIP subfamily | 784232 |
| ch01 | 86846484 | 86849599 | 86848042 | PGSC0003DMG400001662 | Arginine decarboxylase | 1041271.5 |
| ch01 | 87273424 | 87276518 | 87274971 | PGSC0003DMG400001620 | ARM repeat containing protein | 614342 |
| ch01 | 87445881 | 87451796 | 87448839 | PGSC0003DMG400006175 | Ascorbate peroxidase | 440474.5 |
| ch01 | 88106519 | 88107940 | 88107230 | PGSC0003DMG400037545 | Aspartic proteinase nepenthesin-1 | 217916.5 |
| ch01 | 87597051 | 87598241 | 87597646 | PGSC0003DMG400006187 | ATP binding protein | 291667 |
| ch01 | 87094453 | 87096858 | 87095656 | PGSC0003DMG400034487 | ATP-dependent RNA helicase | 793657.5 |
| ch01 | 88296289 | 88299206 | 88297748 | PGSC0003DMG400014674 | ATSLY1 | 408434.5 |
| ch01 | 86927637 | 86928507 | 86928072 | PGSC0003DMG400001655 | Auxin-induced protein 6B | 961241 |
| ch01 | 87102606 | 87103638 | 87103122 | PGSC0003DMG400001641 | Auxin-induced SAUR | 786191 |
| ch01 | 87062996 | 87063708 | 87063352 | PGSC0003DMG400001601 | Auxin-responsive family protein | 825961 |
| ch01 | 87877940 | 87882652 | 87880296 | PGSC0003DMG400018658 | Axi 1 protein | 9017 |
| ch01 | 87981368 | 87986533 | 87983951 | PGSC0003DMG401018633 | Axoneme-associated protein mst101 | 94637.5 |
| ch01 | 86038141 | 86041879 | 86040010 | PGSC0003DMG400000711 | Basic helix-loop-helix protein BHLH7 | 1849303 |
| ch01 | 86354505 | 86360354 | 86357430 | PGSC0003DMG400030961 | BEL30 protein | 1531883.5 |
| ch01 | 87524941 | 87529189 | 87527065 | PGSC0003DMG400006191 | Beta-galactosidase | 362248 |
| ch01 | 86138599 | 86145134 | 86141867 | PGSC0003DMG400000736 | Bifunctional dihydrofolate reductase-thymidylate synthase | 1747446.5 |
| ch01 | 86264083 | 86265430 | 86264757 | PGSC0003DMG400030946 | BZIP transcription factor | 1624556.5 |
| ch01 | 87734657 | 87738766 | 87736712 | PGSC0003DMG400018042 | Bzip-like transcription factor | 152601.5 |

| | | | | | | |
|---|---|---|---|---|---|---|
| ch01 | 88271449 | 88272913 | 88272181 | PGSC0003DMG400014672 | C-7 protein | 382868 |
| ch01 | 87143395 | 87145217 | 87144306 | PGSC0003DMG400001635 | Chalcone synthase | 745007 |
| ch01 | 86277264 | 86283435 | 86280350 | PGSC0003DMG400030947 | Chaperone protein dnaJ | 1608963.5 |
| ch01 | 87600173 | 87604333 | 87602253 | PGSC0003DMG400006169 | Chloroplast zebra-necrosis protein | 287060 |
| ch01 | 86346271 | 86354249 | 86350260 | PGSC0003DMG400030952 | Chromatin remodeling complex subunit | 1539053 |
| ch01 | 86608965 | 86615561 | 86612263 | PGSC0003DMG400003111 | Clathrin binding protein | 1277050 |
| ch01 | 86570347 | 86573835 | 86572091 | PGSC0003DMG400003128 | CRS1 / YhbY domain containing protein | 1317222 |
| ch01 | 86321337 | 86322923 | 86322130 | PGSC0003DMG400037681 | CT099 | 1567183 |
| ch01 | 87361285 | 87363990 | 87362638 | PGSC0003DMG400006198 | Cucumisin | 526675.5 |
| ch01 | 86439555 | 86442130 | 86440843 | PGSC0003DMG400030957 | Cysteine proteinase | 1448470.5 |
| ch01 | 88590113 | 88591214 | 88590664 | PGSC0003DMG400014671 | Cytohesin 1, 2, 3 | 701350.5 |
| ch01 | 86148173 | 86153485 | 86150829 | PGSC0003DMG400000737 | Diaminopimelate decarboxylase 1, chloroplastic | 1738484 |
| ch01 | 87833371 | 87841550 | 87837461 | PGSC0003DMG400018659 | Dimethylaniline monooxygenase | 51852.5 |
| ch01 | 87247582 | 87248148 | 87247865 | PGSC0003DMG400001623 | Disease resistance response protein | 641448 |
| ch01 | 87564439 | 87569506 | 87566973 | PGSC0003DMG401006188 | DNA binding protein | 322340.5 |
| ch01 | 87852373 | 87860890 | 87856632 | PGSC0003DMG400018638 | DNA-damage-inducible protein f | 32681.5 |
| ch01 | 87390883 | 87395297 | 87393090 | PGSC0003DMG402006194 | Early-responsive to dehydration 7 | 496223 |
| ch01 | 85937523 | 85941074 | 85939299 | PGSC0003DMG400000727 | EMB2219 | 1950014.5 |
| ch01 | 86651856 | 86657814 | 86654835 | PGSC0003DMG400003124 | Endo-1,4-beta-glucanase | 1234478 |
| ch01 | 87316007 | 87318162 | 87317085 | PGSC0003DMG400006180 | Endo-1,4-beta-xylanase A | 572228.5 |
| ch01 | 87153114 | 87160149 | 87156632 | PGSC0003DMG400001597 | ER lumen protein retaining receptor | 732681.5 |
| ch01 | 87862741 | 87865242 | 87863992 | PGSC0003DMG400018637 | Erwinia induced protein 1 | 25321.5 |
| ch01 | 87935729 | 87937391 | 87936560 | PGSC0003DMG400018635 | Expansin | 47247 |
| ch01 | 87996455 | 88001717 | 87999086 | PGSC0003DMG400018632 | F-box family protein | 109773 |
| ch01 | 86663461 | 86666351 | 86664906 | PGSC0003DMG400003123 | Fructose-bisphosphate aldolase | 1224407 |
| ch01 | 86875171 | 86879531 | 86877351 | PGSC0003DMG400001660 | GAGA-binding transcriptional activator BBR/BPC4 | 1011962 |
| ch01 | 86092273 | 86097263 | 86094768 | PGSC0003DMG400000735 | Glucose-1-phosphate adenylyltransferase | 1794545 |
| ch01 | 88379429 | 88385233 | 88382331 | PGSC0003DMG400014663 | Glutamate receptor 3 plant | 493018 |
| ch01 | 88260213 | 88261601 | 88260907 | PGSC0003DMG400014655 | Glutathione S-transferase | 371594 |
| ch01 | 88256440 | 88257947 | 88257194 | PGSC0003DMG400014654 | Glutathione S-transferase/peroxidase | 367880.5 |
| ch01 | 87865977 | 87872886 | 87869432 | PGSC0003DMG400018636 | Glycerol-3-phosphate acyltransferase | 19881.5 |
| ch01 | 87281207 | 87282758 | 87281983 | PGSC0003DMG400001590 | Glycine-rich protein 2 | 607330.5 |
| ch01 | 85987215 | 85988480 | 85987848 | PGSC0003DMG400000708 | Glycine-rich RNA binding protein | 1901465.5 |
| ch01 | 88344294 | 88349989 | 88347142 | PGSC0003DMG400014677 | Glycosyltransferase QUASIMODO1 | 457828.5 |
| ch01 | 86051737 | 86057675 | 86054706 | PGSC0003DMG400000732 | Hydrolase, alpha/beta fold family protein | 1834607 |

| | | | | | | |
|---|---|---|---|---|---|---|
| ch01 | 87612479 | 87616575 | 87614527 | PGSC0003DMG400006186 | Hydroxypyruvate reductase | 274786 |
| ch01 | 88307743 | 88312694 | 88310219 | PGSC0003DMG400014684 | Integrase core domain containing protein | 420905.5 |
| ch01 | 87909461 | 87913813 | 87911637 | PGSC0003DMG400018655 | Intron maturase, type II family protein | 22324 |
| ch01 | 86087334 | 86091394 | 86089364 | PGSC0003DMG400000714 | Isoform 2 of F-box protein | 1799949 |
| ch01 | 86334523 | 86337144 | 86335834 | PGSC0003DMG400043836 | Kinase | 1553479.5 |
| ch01 | 86302942 | 86303860 | 86303401 | PGSC0003DMG400030949 | Late embryogenesis abundant protein | 1585912 |
| ch01 | 85948415 | 85955290 | 85951853 | PGSC0003DMG400000728 | Lrr receptor protein kinase | 1937460.5 |
| ch01 | 86454723 | 86460664 | 86457694 | PGSC0003DMG400030968 | Minichromosome maintenance 4 protein | 1431619.5 |
| ch01 | 87533756 | 87537370 | 87535563 | PGSC0003DMG400006190 | Minichromosome maintenance protein 10 isoform 1 | 353750 |
| ch01 | 88395361 | 88399887 | 88397624 | PGSC0003DMG401014665 | Mitochondrial phosphate carrier protein | 508311 |
| ch01 | 86632715 | 86636141 | 86634428 | PGSC0003DMG400003110 | Mitochondrion protein | 1254885 |
| ch01 | 87135798 | 87142796 | 87139297 | PGSC0003DMG400001636 | Mta/sah nucleosidase | 750016 |
| ch01 | 87970174 | 87974234 | 87972204 | PGSC0003DMG400018650 | Multicopper oxidase | 82891 |
| ch01 | 86305394 | 86310803 | 86308099 | PGSC0003DMG400030950 | Myo inositol monophosphatase | 1581214.5 |
| ch01 | 88362828 | 88366404 | 88364616 | PGSC0003DMG403014680 | N-acetylglucosaminyltransferase I | 475303 |
| ch01 | 86741752 | 86750553 | 86746153 | PGSC0003DMG400005571 | NADH dehydrogenase | 1143160.5 |
| ch01 | 85925678 | 85931347 | 85928513 | PGSC0003DMG400000726 | NADH:ubiquinone oxidoreductase | 1960800.5 |
| ch01 | 88168554 | 88170661 | 88169608 | PGSC0003DMG400003415 | Neutral amino acid transport protein | 280294.5 |
| ch01 | 86075422 | 86078050 | 86076736 | PGSC0003DMG400000713 | Nitrate and chloride transporter | 1812577 |
| ch01 | 87319694 | 87323981 | 87321838 | PGSC0003DMG400006179 | Nodulin family protein | 567475.5 |
| ch01 | 87223461 | 87227818 | 87225640 | PGSC0003DMG400001629 | Non-imprinted in Prader-Willi/Angelman syndrome region protein | 663673.5 |
| ch01 | 87829221 | 87831072 | 87830147 | PGSC0003DMG400018663 | Non-LTR retroelement reverse transcriptase | 59166.5 |
| ch01 | 87569991 | 87573211 | 87571601 | PGSC0003DMG400006171 | Nucleosome/chromatin assembly factor group | 317712 |
| ch01 | 88017235 | 88018529 | 88017882 | PGSC0003DMG401018646 | O-methyltransferase | 128569 |
| ch01 | 88420646 | 88435054 | 88427850 | PGSC0003DMG400014681 | Pantothenate kinase | 538537 |
| ch01 | 86067242 | 86069150 | 86068196 | PGSC0003DMG400000712 | Pectinesterase 11 | 1821117 |
| ch01 | 87924533 | 87930883 | 87927708 | PGSC0003DMG400018653 | Pentatricopeptide repeat-containing protein | 38395 |
| ch01 | 86390093 | 86393366 | 86391730 | PGSC0003DMG400030962 | Pentatricopeptide repeat-containing protein, chloroplastic | 1497583.5 |
| ch01 | 87335620 | 87340245 | 87337933 | PGSC0003DMG400006200 | Peptidyl-prolyl cis-trans isomerase | 551380.5 |
| ch01 | 86908135 | 86911506 | 86909821 | PGSC0003DMG400001617 | Peptidyl-prolyl cis-trans isomerase Pin1 | 979492.5 |
| ch01 | 88359122 | 88359717 | 88359420 | PGSC0003DMG400014679 | PGPS/NH21 | 470106.5 |
| ch01 | 86393950 | 86398907 | 86396429 | PGSC0003DMG400030955 | Phd finger transcription factor | 1492884.5 |
| ch01 | 88618988 | 88627702 | 88623345 | PGSC0003DMG400018039 | PhD-finger protein | 734032 |
| ch01 | 86160370 | 86165726 | 86163048 | PGSC0003DMG400000738 | Phosphatidylinositol transporter | 1726265 |
| ch01 | 87264139 | 87265609 | 87264874 | PGSC0003DMG400001621 | Phospholipase C | 624439 |

| | | | | | | |
|---|---|---|---|---|---|---|
| ch01 | 87454143 | 87463868 | 87459006 | PGSC0003DMG401006174 | Plant synaptotagmin | 430307.5 |
| ch01 | 87210776 | 87213515 | 87212146 | PGSC0003DMG400001631 | Proline-rich protein | 677167.5 |
| ch01 | 87397428 | 87400764 | 87399096 | PGSC0003DMG400006193 | Proteasome subunit alpha type | 490217 |
| ch01 | 87132254 | 87135002 | 87133628 | PGSC0003DMG400001637 | Protein COBRA | 755685 |
| ch01 | 87196467 | 87202723 | 87199595 | PGSC0003DMG400001633 | Protein FAM50A | 689718 |
| ch01 | 87378745 | 87385317 | 87382031 | PGSC0003DMG401006195 | Protein farnesyltransferase alpha subunit | 507282 |
| ch01 | 88067669 | 88076117 | 88071893 | PGSC0003DMG400018644 | Protein kinase | 182580 |
| ch01 | 87745867 | 87751858 | 87748863 | PGSC0003DMG400018043 | Protein phosphatase 2c | 140450.5 |
| ch01 | 86078315 | 86082128 | 86080222 | PGSC0003DMG400000734 | Protein RER1B | 1809091.5 |
| ch01 | 86568059 | 86568658 | 86568359 | PGSC0003DMG400003129 | Protein terminal ear1 homolog | 1320954.5 |
| ch01 | 87248648 | 87255682 | 87252165 | PGSC0003DMG400001593 | Protein translocase | 637148 |
| ch01 | 86412833 | 86417264 | 86415049 | PGSC0003DMG400030965 | Purple acid phosphatase | 1474264.5 |
| ch01 | 87160535 | 87164471 | 87162503 | PGSC0003DMG400001596 | Putative neutral invertase | 726810 |
| ch01 | 87542094 | 87549232 | 87545663 | PGSC0003DMG400006189 | Rac-like GTP-binding protein | 343650 |
| ch01 | 86043675 | 86046985 | 86045330 | PGSC0003DMG400000731 | Response to dessication RD2 | 1843983 |
| ch01 | 87079180 | 87080289 | 87079735 | PGSC0003DMG400001600 | Retinoblastoma-binding protein | 809578.5 |
| ch01 | 86914257 | 86919750 | 86917004 | PGSC0003DMG400001616 | Ribosome-binding factor A | 972309.5 |
| ch01 | 87025401 | 87032148 | 87028775 | PGSC0003DMG400001606 | RNA binding protein | 860538.5 |
| ch01 | 86365852 | 86367431 | 86366642 | PGSC0003DMG400030953 | RNA-binding protein | 1522671.5 |
| ch01 | 88047327 | 88049108 | 88048218 | PGSC0003DMG400018630 | RNase H family protein | 158904.5 |
| ch01 | 87994278 | 87999370 | 87996824 | PGSC0003DMG400018648 | Rop guanine nucleotide exchange factor | 107511 |
| ch01 | 88336794 | 88341542 | 88339168 | PGSC0003DMG400014662 | RWP-RK domain-containing protein | 449855 |
| ch01 | 88126157 | 88127569 | 88126863 | PGSC0003DMG400018641 | S-adenosyl-methionine-sterol-C-methyltransferase | 237550 |
| ch01 | 86666771 | 86673688 | 86670230 | PGSC0003DMG400003109 | Salt-tolerance protein | 1219083.5 |
| ch01 | 87056324 | 87057059 | 87056692 | PGSC0003DMG400001602 | SAUR family protein | 832621.5 |
| ch01 | 86992768 | 86993100 | 86992934 | PGSC0003DMG400036638 | SAUR9-auxin-responsive SAUR family member | 896379 |
| ch01 | 86156827 | 86160116 | 86158472 | PGSC0003DMG400000719 | Sec14 cytosolic factor | 1730841.5 |
| ch01 | 88171084 | 88172578 | 88171831 | PGSC0003DMG400003416 | Serine/threonine protein phosphatase 2A 57 kDa regulatory subunit B' beta isoform | 282518 |
| ch01 | 88078283 | 88079567 | 88078925 | PGSC0003DMG402018643 | Serine/threonine-protein kinase ATR | 189612 |
| ch01 | 88351126 | 88353936 | 88352531 | PGSC0003DMG400014678 | Serine/threonine-protein kinase cx32 | 463218 |
| ch01 | 85902204 | 85906523 | 85904364 | PGSC0003DMG400000724 | Serine/threonine-protein kinase PBS1 | 1984949.5 |
| ch01 | 87977131 | 87980816 | 87978974 | PGSC0003DMG403018633 | Serine-threonine protein kinase, plant-type | 89660.5 |
| ch01 | 87621468 | 87623667 | 87622568 | PGSC0003DMG400006184 | Skp1 | 266745.5 |
| ch01 | 87619565 | 87621021 | 87620293 | PGSC0003DMG400006185 | Skp1 1 | 269020 |

| | | | | | | |
|---|---|---|---|---|---|---|
| ch01 | 87145671 | 87146801 | 87146236 | PGSC0003DMG400001598 | Snakin-2 | 743077 |
| ch01 | 88151620 | 88156317 | 88153969 | PGSC0003DMG400018627 | Structural constituent of ribosome | 264655.5 |
| ch01 | 88386348 | 88390053 | 88388201 | PGSC0003DMG400035539 | Sugar transporter | 498887.5 |
| ch01 | 86072484 | 86074144 | 86073314 | PGSC0003DMG400000733 | Syntaxin | 1815999 |
| ch01 | 86548402 | 86549649 | 86549026 | PGSC0003DMG400040138 | TEL1 (TERMINAL EAR1-LIKE 1) | 1340287.5 |
| ch01 | 86556182 | 86557218 | 86556700 | PGSC0003DMG400035883 | Terminal ear1-like 2 protein | 1332613 |
| ch01 | 88109832 | 88111824 | 88110828 | PGSC0003DMG400018629 | Tetracycline transporter | 221515 |
| ch01 | 87300111 | 87304770 | 87302441 | PGSC0003DMG400006202 | Thaumatin | 586872.5 |
| ch01 | 87438016 | 87439876 | 87438946 | PGSC0003DMG400006176 | Transcription factor | 450367 |
| ch01 | 87122442 | 87128024 | 87125233 | PGSC0003DMG400001639 | Transducin family protein | 764080 |
| ch01 | 87229243 | 87229810 | 87229527 | PGSC0003DMG400001628 | Translation initiation factor | 659786.5 |
| ch01 | 87665036 | 87671203 | 87668120 | PGSC0003DMG400006166 | Translation initiation factor if-3 | 221193.5 |
| ch01 | 87203582 | 87208255 | 87205919 | PGSC0003DMG400001632 | Translocon-associated protein, alpha subunit | 683394.5 |
| ch01 | 87179147 | 87185680 | 87182414 | PGSC0003DMG400001595 | Triosephosphate isomerase, chloroplastic | 706899.5 |
| ch01 | 86894067 | 86898854 | 86896461 | PGSC0003DMG400001658 | TRNA-dihydrouridine synthase | 992852.5 |
| ch01 | 86759392 | 86765562 | 86762477 | PGSC0003DMG401005570 | Ubiquitin fusion-degradation protein | 1126836 |
| ch01 | 86324339 | 86333693 | 86329016 | PGSC0003DMG400030951 | Ubiquitin-activating enzyme E1b | 1560297 |
| ch01 | 87638813 | 87648550 | 87643682 | PGSC0003DMG400006168 | Ubiquitin-conjugating enzyme E2 23 | 245631.5 |
| ch01 | 88051523 | 88058221 | 88054872 | PGSC0003DMG400018645 | Ubiquitin-protein ligase | 165559 |
| ch01 | 87086178 | 87088612 | 87087395 | PGSC0003DMG400001646 | Ubiquitin-protein ligase BRE1A | 801918 |
| ch01 | 88198015 | 88203917 | 88200966 | PGSC0003DMG400003419 | Vacuolar ATPase subunit B | 311653 |
| ch01 | 88097001 | 88105050 | 88101026 | PGSC0003DMG400018642 | WD-repeat protein | 211712.5 |
| ch01 | 87313357 | 87315412 | 87314385 | PGSC0003DMG400006201 | Zeamatin | 574928.5 |
| ch01 | 86524540 | 86526577 | 86525559 | PGSC0003DMG400030958 | Zinc finger (B-box type) family protein | 1363754.5 |
| ch01 | 87974590 | 87977051 | 87975821 | PGSC0003DMG400018649 | Zinc finger protein | 86507.5 |

| chr | start | end | mid | ID | Name | hit_dist |
|---|---|---|---|---|---|---|
| ch02 | 25643324 | 25644416 | 25643870 | PGSC0003DMG400006631 | 60S ribosomal protein L34 | 165604 |
| ch02 | 26215534 | 26217231 | 26216383 | PGSC0003DMG400006944 | Abscission polygalacturonase | 738116.5 |
| ch02 | 24696172 | 24698146 | 24697159 | PGSC0003DMG400001181 | ACI19 | 781107 |
| ch02 | 26568162 | 26572977 | 26570570 | PGSC0003DMG400006918 | Aldose 1-epimerase | 1092304 |
| ch02 | 27100625 | 27106372 | 27103499 | PGSC0003DMG400009430 | Allantoate amidohydrolase | 1625233 |
| ch02 | 24907881 | 24913763 | 24910822 | PGSC0003DMG400026549 | Amino acid transporter | 567444 |
| ch02 | 23549631 | 23551346 | 23550489 | PGSC0003DMG400025470 | Aspartic proteinase nepenthesin-1 | 1927778 |
| ch02 | 23729016 | 23730239 | 23729628 | PGSC0003DMG401000764 | Asymmetric leaves 2 | 1748639 |
| ch02 | 26872447 | 26876735 | 26874591 | PGSC0003DMG400030198 | AT.I.24-7 protein | 1396325 |
| ch02 | 23826195 | 23831677 | 23828936 | PGSC0003DMG400000748 | ATGSL04 (glucan synthase-like 4) | 1649330 |
| ch02 | 23491685 | 23493155 | 23492420 | PGSC0003DMG400025472 | ATP binding protein | 1985846 |
| ch02 | 25951421 | 25953871 | 25952646 | PGSC0003DMG402006935 | Avr9/Cf-9 rapidly elicited protein 126 | 474380 |
| ch02 | 26167611 | 26169974 | 26168793 | PGSC0003DMG400006903 | B12D | 690526.5 |
| ch02 | 26778585 | 26783552 | 26781069 | PGSC0003DMG400006977 | B2 protein | 1302803 |
| ch02 | 24225414 | 24226353 | 24225884 | PGSC0003DMG400043353 | B3 domain-containing protein REM10 | 1252383 |
| ch02 | 26140528 | 26143532 | 26142030 | PGSC0003DMG400006942 | BRASSINOSTEROID INSENSITIVE 1-associated receptor kinase 1 | 663764 |
| ch02 | 26429429 | 26430754 | 26430092 | PGSC0003DMG400006954 | C2H2L domain class transcription factor | 951825.5 |
| ch02 | 23675189 | 23681226 | 23678208 | PGSC0003DMG400000754 | Calcium/calmodulin-dependent protein kinase CaMK3 | 1800059 |
| ch02 | 25570056 | 25570855 | 25570456 | PGSC0003DMG400025131 | Calcium-binding EF hand family protein | 92189.5 |
| ch02 | 26259385 | 26259951 | 26259668 | PGSC0003DMG400045678 | Calmodulin-binding protein | 781402 |
| ch02 | 26445716 | 26447338 | 26446527 | PGSC0003DMG400006956 | Carbonic anhydrase | 968261 |
| ch02 | 24751550 | 24758595 | 24755073 | PGSC0003DMG400001179 | Carboxylesterase | 723193.5 |
| ch02 | 26197721 | 26201109 | 26199415 | PGSC0003DMG400006943 | Cation efflux family protein | 721149 |
| ch02 | 26573252 | 26573997 | 26573625 | PGSC0003DMG400006961 | Chalcone isomerase | 1095359 |
| ch02 | 27143835 | 27151765 | 27147800 | PGSC0003DMG400009432 | Cholinephosphate cytidylyltransferase | 1669534 |
| ch02 | 26769358 | 26772980 | 26771169 | PGSC0003DMG400006925 | Chromatin remodeling complex subunit | 1292903 |
| ch02 | 26743487 | 26748156 | 26745822 | PGSC0003DMG400006975 | CLC-Nt2 protein | 1267556 |
| ch02 | 26139078 | 26140386 | 26139732 | PGSC0003DMG400006901 | CLE25 | 661466 |
| ch02 | 26553518 | 26558167 | 26555843 | PGSC0003DMG400006917 | Clostridium pasteurianum ferredoxin homolog | 1077577 |
| ch02 | 25678236 | 25679521 | 25678879 | PGSC0003DMG400006628 | Condensin complex components subunit | 200612.5 |
| ch02 | 25028328 | 25030025 | 25029177 | PGSC0003DMG400037406 | Cop9 complex subunit 7a | 449089.5 |
| ch02 | 24385896 | 24392008 | 24388952 | PGSC0003DMG400002745 | COR413-PM2 | 1089314 |
| ch02 | 27267027 | 27269674 | 27268351 | PGSC0003DMG400010436 | Ctp synthase | 1790085 |
| ch02 | 27027966 | 27033908 | 27030937 | PGSC0003DMG400009414 | Cucumisin | 1552671 |

| | | | | | | |
|---|---|---|---|---|---|---|
| ch02 | 23998168 | 23999105 | 23998637 | PGSC0003DMG400000740 | Cyclic nucleotide-gated ion channel | 1479630 |
| ch02 | 25509435 | 25515225 | 25512330 | PGSC0003DMG400025134 | Cystathionine gamma synthase | 34064 |
| ch02 | 24744793 | 24750751 | 24747772 | PGSC0003DMG400001180 | Cytochrome b561 | 730494 |
| ch02 | 24977514 | 24981300 | 24979407 | PGSC0003DMG400037578 | Cytochrome P450 85A3 | 498859 |
| ch02 | 26927957 | 26933255 | 26930606 | PGSC0003DMG400009407 | Dead box ATP-dependent RNA helicase | 1452340 |
| ch02 | 26992914 | 26997907 | 26995411 | PGSC0003DMG400009423 | Diacylglycerol O-acyltransferase 2 | 1517145 |
| ch02 | 24949315 | 24949686 | 24949501 | PGSC0003DMG400042222 | DNA binding protein | 528765.5 |
| ch02 | 24191712 | 24192813 | 24192263 | PGSC0003DMG400013103 | DNA-binding protein | 1286004 |
| ch02 | 26307874 | 26316954 | 26312414 | PGSC0003DMG400006911 | DNA-repair protein UVH3 | 834148 |
| ch02 | 24148416 | 24149311 | 24148864 | PGSC0003DMG400013105 | DOF domain class transcription factor | 1329403 |
| ch02 | 24734365 | 24735140 | 24734753 | PGSC0003DMG400001188 | DVL protein | 743513.5 |
| ch02 | 26773990 | 26778122 | 26776056 | PGSC0003DMG401006926 | E2 protein isoform 5 | 1297790 |
| ch02 | 23514915 | 23515515 | 23515215 | PGSC0003DMG400025477 | EF hand family protein | 1963051 |
| ch02 | 24525986 | 24529953 | 24527970 | PGSC0003DMG400022012 | Electron transporter | 950296.5 |
| ch02 | 24135282 | 24140915 | 24138099 | PGSC0003DMG400013101 | Ethylene-induced esterase | 1340168 |
| ch02 | 24507215 | 24508933 | 24508074 | PGSC0003DMG400044365 | F-box family protein | 970192 |
| ch02 | 23955312 | 23957463 | 23956388 | PGSC0003DMG400000758 | Geraniol 10-hydroxylase | 1521879 |
| ch02 | 27064966 | 27067520 | 27066243 | PGSC0003DMG400009427 | Gibberellin 3-beta-dioxygenase | 1587977 |
| ch02 | 27134922 | 27142882 | 27138902 | PGSC0003DMG400009431 | Glioma tumor suppressor | 1660636 |
| ch02 | 26613043 | 26615973 | 26614508 | PGSC0003DMG400006965 | Glycerol-3-phosphate dehydrogenase | 1136242 |
| ch02 | 24654053 | 24655674 | 24654864 | PGSC0003DMG400001183 | Glycosyltransferase family GT8 protein | 823402.5 |
| ch02 | 25451506 | 25457946 | 25454726 | PGSC0003DMG400025139 | Glycosyltransferase QUASIMODO1 | 23540 |
| ch02 | 26187591 | 26189049 | 26188320 | PGSC0003DMG400006907 | Glycosyltransferase UGT90A7 | 710054 |
| ch02 | 26535433 | 26540694 | 26538064 | PGSC0003DMG400006959 | GPI transamidase component family protein / Gaa1-like family protein | 1059798 |
| ch02 | 26394119 | 26396910 | 26395515 | PGSC0003DMG400006952 | HEAT repeat family protein | 917248.5 |
| ch02 | 27194995 | 27203856 | 27199426 | PGSC0003DMG400009436 | Heavy metal ATPase | 1721160 |
| ch02 | 24202861 | 24204696 | 24203779 | PGSC0003DMG400013104 | High mobility group protein 2 HMG2 | 1274488 |
| ch02 | 25814780 | 25816152 | 25815466 | PGSC0003DMG400006928 | Homeobox domain, ZF-HD class | 337200 |
| ch02 | 25943284 | 25944649 | 25943967 | PGSC0003DMG400006895 | Homeobox protein | 465700.5 |
| ch02 | 24575906 | 24581104 | 24578505 | PGSC0003DMG400022011 | Homeodomain protein | 899761 |
| ch02 | 26023981 | 26028539 | 26026260 | PGSC0003DMG400006939 | Hypersensitive-induced response protein | 547994 |
| ch02 | 26632792 | 26633124 | 26632958 | PGSC0003DMG400039400 | Integrase core domain containing protein | 1154692 |
| ch02 | 25558357 | 25566224 | 25562291 | PGSC0003DMG400025132 | KH domain-containing protein | 84024.5 |
| ch02 | 27092727 | 27100291 | 27096509 | PGSC0003DMG400009417 | Kinase | 1618243 |
| ch02 | 27068991 | 27078771 | 27073881 | PGSC0003DMG400009428 | Kinesin heavy chain | 1595615 |

| | | | | | | |
|---|---|---|---|---|---|---|
| ch02 | 27046801 | 27053562 | 27050182 | PGSC0003DMG401009415 | Lectin kinase | 1571916 |
| ch02 | 26728850 | 26734020 | 26731435 | PGSC0003DMG400006923 | Leucine-rich repeat protein | 1253169 |
| ch02 | 24183991 | 24188089 | 24186040 | PGSC0003DMG400013102 | Leucyl-tRNA synthetase | 1292226 |
| ch02 | 26734234 | 26740368 | 26737301 | PGSC0003DMG400006924 | Lipase | 1259035 |
| ch02 | 26516824 | 26519994 | 26518409 | PGSC0003DMG400006914 | LOB domain-containing protein | 1040143 |
| ch02 | 25493501 | 25494016 | 25493759 | PGSC0003DMG400039595 | Mads box protein | 15492.5 |
| ch02 | 24952023 | 24959857 | 24955940 | PGSC0003DMG400026563 | MADS-box transcription factor FBP29 | 522326 |
| ch02 | 23711545 | 23718325 | 23714935 | PGSC0003DMG400000752 | MAP protein kinase | 1763331 |
| ch02 | 26022012 | 26025794 | 26023903 | PGSC0003DMG400006896 | Mitochondrial outer membrane protein porin of 34 kDa | 545637 |
| ch02 | 24376556 | 24381507 | 24379032 | PGSC0003DMG400002783 | MRNA, clone: rafl24-09-C04 | 1099235 |
| ch02 | 23796212 | 23800871 | 23798542 | PGSC0003DMG400000761 | Multicopper oxidase | 1679725 |
| ch02 | 26192369 | 26194461 | 26193415 | PGSC0003DMG400034811 | NADH-ubiquinone oxidoreductase chain 4 | 715149 |
| ch02 | 27449297 | 27449785 | 27449541 | PGSC0003DMG400042077 | NB-ARC domain containing protein | 1971275 |
| ch02 | 25185566 | 25186519 | 25186043 | PGSC0003DMG400014083 | Ninja-family protein AFP2 | 292223.5 |
| ch02 | 25168907 | 25170514 | 25169711 | PGSC0003DMG400014084 | Ninja-family protein AFP3 | 308555.5 |
| ch02 | 25251637 | 25252262 | 25251950 | PGSC0003DMG400039185 | Ninja-family protein Os07g0602900 | 226316.5 |
| ch02 | 26506508 | 26508700 | 26507604 | PGSC0003DMG400006913 | Nitrate transporter | 1029338 |
| ch02 | 26798546 | 26802505 | 26800526 | PGSC0003DMG400006978 | Nodulin | 1322260 |
| ch02 | 26894502 | 26898165 | 26896334 | PGSC0003DMG400030196 | Nuclear transport factor | 1418068 |
| ch02 | 26280621 | 26281582 | 26281102 | PGSC0003DMG400035480 | Nuclease | 802835.5 |
| ch02 | 25930821 | 25936500 | 25933661 | PGSC0003DMG400006894 | Nucleic acid binding protein | 455394.5 |
| ch02 | 25294273 | 25298142 | 25296208 | PGSC0003DMG400025156 | Organic anion transporter | 182058.5 |
| ch02 | 26070074 | 26077966 | 26074020 | PGSC0003DMG400006940 | Oxidoreductase | 595754 |
| ch02 | 24407426 | 24409769 | 24408598 | PGSC0003DMG400002782 | Oxygen-evolving enhancer protein 1, chloroplastic | 1069669 |
| ch02 | 23709715 | 23713122 | 23711419 | PGSC0003DMG400000765 | Patatin 3 | 1766848 |
| ch02 | 25387468 | 25389338 | 25388403 | PGSC0003DMG400025142 | Pc21g16870 protein | 89863 |
| ch02 | 26003650 | 26005412 | 26004531 | PGSC0003DMG400006937 | Pectate lyase | 526265 |
| ch02 | 26017220 | 26019334 | 26018277 | PGSC0003DMG400006938 | Pectate lyase P59 | 540011 |
| ch02 | 25544055 | 25547783 | 25545919 | PGSC0003DMG400025133 | Pentatricopeptide repeat-containing protein | 67653 |
| ch02 | 26618546 | 26618893 | 26618720 | PGSC0003DMG400037530 | PGPS/D11 | 1140454 |
| ch02 | 23688287 | 23693389 | 23690838 | PGSC0003DMG400000753 | Phosphoglycerate mutase | 1787428 |
| ch02 | 24915574 | 24918901 | 24917238 | PGSC0003DMG400026550 | Phytoene dehydrogenase | 561028.5 |
| ch02 | 26235068 | 26236611 | 26235840 | PGSC0003DMG402006909 | Polygalacturonase | 757573.5 |
| ch02 | 26240574 | 26242080 | 26241327 | PGSC0003DMG403006909 | Polygalacturonase 1 | 763061 |
| ch02 | 27118598 | 27120620 | 27119609 | PGSC0003DMG400009418 | Polygalacturonase QRT3 | 1641343 |

| | | | | | | |
|---|---|---|---|---|---|---|
| ch02 | 27417702 | 27427277 | 27422490 | PGSC0003DMG400010431 | Potassium transporter 11 | 1944224 |
| ch02 | 25497876 | 25499585 | 25498731 | PGSC0003DMG400025147 | Pro-resilin | 20464.5 |
| ch02 | 25864790 | 25870666 | 25867728 | PGSC0003DMG400006931 | Protease degQ | 389462 |
| ch02 | 23736316 | 23739528 | 23737922 | PGSC0003DMG402000764 | Protein ASYMMETRIC LEAVES2 | 1740344 |
| ch02 | 24921508 | 24922442 | 24921975 | PGSC0003DMG401026561 | Protein bem46 | 556291 |
| ch02 | 25001720 | 25005439 | 25003580 | PGSC0003DMG400026565 | Protein COBRA | 474686.5 |
| ch02 | 26500044 | 26504296 | 26502170 | PGSC0003DMG400006912 | Protein kinase family protein | 1023904 |
| ch02 | 26650250 | 26658080 | 26654165 | PGSC0003DMG400006970 | Protein phosphatase 2a, regulatory subunit | 1175899 |
| ch02 | 25304888 | 25313841 | 25309365 | PGSC0003DMG400025145 | Protein RIK | 168901.5 |
| ch02 | 26029561 | 26036145 | 26032853 | PGSC0003DMG400039723 | Retroelement pol polyprotein | 554587 |
| ch02 | 26332879 | 26333997 | 26333438 | PGSC0003DMG400038862 | Ring finger protein | 855172 |
| ch02 | 24670741 | 24677960 | 24674351 | PGSC0003DMG400001182 | RING zinc finger protein 1 | 803915.5 |
| ch02 | 27158860 | 27160255 | 27159558 | PGSC0003DMG400009433 | RNA binding protein | 1681292 |
| ch02 | 25302984 | 25306536 | 25304760 | PGSC0003DMG400025154 | RNA-binding glycine-rich protein | 173506 |
| ch02 | 26359800 | 26360257 | 26360029 | PGSC0003DMG400006983 | RNase H family protein | 881762.5 |
| ch02 | 26582917 | 26587189 | 26585053 | PGSC0003DMG400006963 | S-adenosylmethionine-dependent methyltransferase | 1106787 |
| ch02 | 23995047 | 23995985 | 23995516 | PGSC0003DMG400000755 | Salicylic acid-binding protein 2 | 1482750 |
| ch02 | 27408577 | 27417302 | 27412940 | PGSC0003DMG400010445 | Serine/threonine protein kinase | 1934674 |
| ch02 | 24620485 | 24622699 | 24621592 | PGSC0003DMG400001185 | Serine-threonine protein kinase, plant-type | 856674 |
| ch02 | 26717100 | 26723429 | 26720265 | PGSC0003DMG400006974 | SET domain-containing protein | 1241999 |
| ch02 | 25893333 | 25900884 | 25897109 | PGSC0003DMG400006933 | SET protein, phospatase 2A inhibitor | 418842.5 |
| ch02 | 23667729 | 23670191 | 23668960 | PGSC0003DMG400000767 | Short-chain dehydrogenase | 1809306 |
| ch02 | 26547774 | 26552711 | 26550243 | PGSC0003DMG400006916 | Stpk1 protein kinase | 1071977 |
| ch02 | 26758507 | 26761691 | 26760099 | PGSC0003DMG400006976 | Structural constituent of ribosome | 1281833 |
| ch02 | 25705395 | 25710573 | 25707984 | PGSC0003DMG400013212 | Structural maintenance of chromosome | 229718 |
| ch02 | 24524132 | 24524853 | 24524493 | PGSC0003DMG400022013 | STS14 protein | 953773.5 |
| ch02 | 24371891 | 24374190 | 24373041 | PGSC0003DMG400002784 | Sulfotransferase | 1105226 |
| ch02 | 23769344 | 23777049 | 23773197 | PGSC0003DMG400000750 | SWIB/MDM2 domain containing protein | 1705070 |
| ch02 | 27087805 | 27091498 | 27089652 | PGSC0003DMG402009429 | Taz protein | 1611386 |
| ch02 | 25936946 | 25937917 | 25937432 | PGSC0003DMG400006934 | Thioredoxin fold | 459165.5 |
| ch02 | 27281186 | 27291149 | 27286168 | PGSC0003DMG402010449 | Thioredoxin I | 1807902 |
| ch02 | 27010985 | 27011812 | 27011399 | PGSC0003DMG402009425 | Tonoplast intrinsic protein, alpha (Alpha-TIP) | 1533133 |
| ch02 | 25838187 | 25840887 | 25839537 | PGSC0003DMG400006930 | Transcription factor | 361271 |
| ch02 | 25922325 | 25923640 | 25922983 | PGSC0003DMG400006893 | Transcription factor style2.1 | 444716.5 |
| ch02 | 26681493 | 26682797 | 26682145 | PGSC0003DMG400006972 | Transcription regulator | 1203879 |

| | | | | | | |
|---|---|---|---|---|---|---|
| ch02 | 26127443 | 26132055 | 26129749 | PGSC0003DMG400006899 | Type 2 proly 4-hydroxylase | 651483 |
| ch02 | 25945963 | 25950226 | 25948095 | PGSC0003DMG401006935 | Ubiquitin carrier protein | 469828.5 |
| ch02 | 26619695 | 26620634 | 26620165 | PGSC0003DMG400006966 | Ubiquitin family protein | 1141899 |
| ch02 | 27185531 | 27193793 | 27189662 | PGSC0003DMG400009435 | Ubiquitin specific protease 39 and snrnp assembly factor | 1711396 |
| ch02 | 24845097 | 24851501 | 24848299 | PGSC0003DMG400026556 | Ubiquitin-protein ligase | 629967 |
| ch02 | 26941268 | 26941924 | 26941596 | PGSC0003DMG400009439 | UDP-glucoronosyl/UDP-glucosyl transferase family protein | 1463330 |
| ch02 | 25469249 | 25472361 | 25470805 | PGSC0003DMG400025137 | UDP-glucose dehydrogenase 2 | 7461 |
| ch02 | 24899116 | 24900456 | 24899786 | PGSC0003DMG400026560 | UDP-glucose:glucosyltransferase | 578480 |
| ch02 | 24968977 | 24972151 | 24970564 | PGSC0003DMG400026564 | UDP-glucose:protein transglucosylase SlUPTG1 | 507702 |
| ch02 | 26942142 | 26942887 | 26942515 | PGSC0003DMG400009408 | UDP-glucosyltransferase | 1464249 |
| ch02 | 25338148 | 25340599 | 25339374 | PGSC0003DMG400025152 | UDP-glucuronosyltransferase | 138892.5 |
| ch02 | 27324833 | 27333897 | 27329365 | PGSC0003DMG400010448 | UDP-N-acetylglucosamine pyrophosphorylase | 1851099 |
| ch02 | 26109544 | 26111023 | 26110284 | PGSC0003DMG400006898 | Ulp1 protease family, C-terminal catalytic domain containing protein | 632017.5 |
| ch02 | 25971279 | 25974859 | 25973069 | PGSC0003DMG400006936 | UPA19 | 494803 |
| ch02 | 25541222 | 25542809 | 25542016 | PGSC0003DMG400025146 | UPF0497 membrane protein 3 | 63749.5 |
| ch02 | 26577704 | 26580316 | 26579010 | PGSC0003DMG400006962 | Uridine kinase | 1100744 |
| ch02 | 27085928 | 27087552 | 27086740 | PGSC0003DMG401009429 | Uroporphyrinogen-III synthase | 1608474 |
| ch02 | 27162898 | 27169334 | 27166116 | PGSC0003DMG400009421 | Vacuolar ATP synthase subunit F | 1687850 |
| ch02 | 27170369 | 27171095 | 27170732 | PGSC0003DMG400009442 | VQ motif-containing protein | 1692466 |
| ch02 | 25411538 | 25412823 | 25412181 | PGSC0003DMG400025151 | WIN1 | 66085.5 |
| ch02 | 25500408 | 25508317 | 25504363 | PGSC0003DMG400025135 | Xenotropic and polytropic murine leukemia virus receptor ids-4 | 26096.5 |
| ch02 | 25725358 | 25726928 | 25726143 | PGSC0003DMG400013213 | ZF-HD homeobox protein | 247877 |
| ch02 | 25703464 | 25704788 | 25704126 | PGSC0003DMG400036472 | Zinc finger family protein | 225860 |
| ch02 | 25588000 | 25591776 | 25589888 | PGSC0003DMG400025129 | Zinc finger protein | 111622 |

| chr | start | end | mid | ID | Name | hit_dist |
|---|---|---|---|---|---|---|
| ch03 | 46449680 | 46458376 | 46454028 | PGSC0003DMG400000582 | 1-aminocyclopropane-1-carboxylate oxidase | 850366 |
| ch03 | 44499154 | 44501806 | 44500480 | PGSC0003DMG400019959 | 24 kDa seed maturation protein | 1103182 |
| ch03 | 45948013 | 45954437 | 45951225 | PGSC0003DMG400018513 | 26S proteasome regulatory particle chain RPT6 | 347563 |
| ch03 | 45803438 | 45810464 | 45806951 | PGSC0003DMG400018542 | 3-5 exonuclease | 203289 |
| ch03 | 45467420 | 45471442 | 45469431 | PGSC0003DMG400009128 | 40S ribosomal protein S12 | 134231 |
| ch03 | 47072062 | 47077037 | 47074550 | PGSC0003DMG400003155 | 4-coumarate:CoA ligase | 1470887.5 |
| ch03 | 45999593 | 46004023 | 46001808 | PGSC0003DMG400018511 | 6-phosphogluconolactonase | 398146 |
| ch03 | 44147538 | 44148506 | 44148022 | PGSC0003DMG400009497 | ABC-type Co2+ transport system, permease component | 1455640 |
| ch03 | 45726159 | 45730989 | 45728574 | PGSC0003DMG400018524 | Acyl-CoA-binding domain-containing protein 1 | 124912 |
| ch03 | 46866705 | 46875162 | 46870934 | PGSC0003DMG400000536 | ADP-ribosylation factor, arf | 1267271.5 |
| ch03 | 46006400 | 46010878 | 46008639 | PGSC0003DMG400018510 | Aldo/keto reductase | 404977 |
| ch03 | 44492668 | 44495289 | 44493979 | PGSC0003DMG400045095 | Alpha-farnesene synthase | 1109683.5 |
| ch03 | 43777496 | 43780468 | 43778982 | PGSC0003DMG400010166 | Anthocyanin permease | 1824680 |
| ch03 | 44865282 | 44866451 | 44865867 | PGSC0003DMG400036493 | AP2/ERF domain-containing transcription factor | 737795.5 |
| ch03 | 44915279 | 44916259 | 44915769 | PGSC0003DMG400009099 | Arabinogalactan peptide 22 | 687893 |
| ch03 | 43950496 | 43951544 | 43951020 | PGSC0003DMG400010129 | Aspartic protease inhibitor 10 | 1652642 |
| ch03 | 44035251 | 44036076 | 44035664 | PGSC0003DMG400009511 | Aspartic protease inhibitor 8 | 1567998.5 |
| ch03 | 46248039 | 46250632 | 46249336 | PGSC0003DMG400003220 | AtGRF7 | 645673.5 |
| ch03 | 44679792 | 44685639 | 44682716 | PGSC0003DMG400006594 | ATMRP14 | 920946.5 |
| ch03 | 45936235 | 45940161 | 45938198 | PGSC0003DMG400018514 | ATP binding protein | 334536 |
| ch03 | 45485675 | 45495233 | 45490454 | PGSC0003DMG400009130 | ATP-dependent clp protease ATP-binding subunit clpx | 113208 |
| ch03 | 44988465 | 45001965 | 44995215 | PGSC0003DMG400009105 | ATP-dependent peptidase | 608447 |
| ch03 | 46568886 | 46572210 | 46570548 | PGSC0003DMG400000553 | Auxin-induced protein 5NG4 | 966886 |
| ch03 | 46304803 | 46305439 | 46305121 | PGSC0003DMG400003217 | Avr9/Cf-9 rapidly elicited protein 65 | 701459 |
| ch03 | 46601337 | 46608827 | 46605082 | PGSC0003DMG400000577 | Band 7 family protein | 1001420 |
| ch03 | 44781628 | 44786960 | 44784294 | PGSC0003DMG400009066 | Beta-1,3-galactosyltransferase sqv-2 | 819368 |
| ch03 | 43936158 | 43938595 | 43937377 | PGSC0003DMG400010169 | Beta-carotene hydroxylase | 1666285.5 |
| ch03 | 45141336 | 45145109 | 45143223 | PGSC0003DMG400009114 | BRASSINOSTEROID INSENSITIVE 1-associated receptor kinase 1 | 460439.5 |
| ch03 | 46540632 | 46545106 | 46542869 | PGSC0003DMG401000554 | Calcium ion binding protein | 939207 |
| ch03 | 45438804 | 45442941 | 45440873 | PGSC0003DMG401009127 | Calcium sensor calcineurin B | 162789.5 |
| ch03 | 47134220 | 47136308 | 47135264 | PGSC0003DMG400003146 | Calmodulin | 1531602 |
| ch03 | 45023262 | 45026042 | 45024652 | PGSC0003DMG400009108 | CAPIP1 | 579010 |
| ch03 | 45662577 | 45668394 | 45665486 | PGSC0003DMG401018530 | Carboxylesterase np | 61823.5 |
| ch03 | 46454335 | 46460428 | 46457382 | PGSC0003DMG400000560 | Catechol O-methyltransferase | 853719.5 |

| | | | | | | |
|---|---|---|---|---|---|---|
| ch03 | 46827669 | 46830411 | 46829040 | PGSC0003DMG400000540 | Cell division control 20 | 1225378 |
| ch03 | 47254461 | 47259263 | 47256862 | PGSC0003DMG400003152 | Cinnamoyl-CoA reductase | 1653200 |
| ch03 | 44670486 | 44671710 | 44671098 | PGSC0003DMG400006591 | Class III chitinase | 932564 |
| ch03 | 46512761 | 46515488 | 46514125 | PGSC0003DMG400000556 | Copper ion binding protein | 910462.5 |
| ch03 | 44615207 | 44616546 | 44615877 | PGSC0003DMG400006586 | Crooked neck protein | 987785.5 |
| ch03 | 46357554 | 46358916 | 46358235 | PGSC0003DMG402000586 | Cucumisin | 754573 |
| ch03 | 45090645 | 45098806 | 45094726 | PGSC0003DMG400009076 | Cyclic nucleotide-gated calmodulin-binding ion channel | 508936.5 |
| ch03 | 47368721 | 47373776 | 47371249 | PGSC0003DMG400026473 | Cystatin | 1767586.5 |
| ch03 | 45976310 | 45979862 | 45978086 | PGSC0003DMG400018534 | Cytochome b5 | 374424 |
| ch03 | 46939241 | 46939699 | 46939470 | PGSC0003DMG400036058 | Cytochrome P450 | 1335808 |
| ch03 | 47411197 | 47411568 | 47411383 | PGSC0003DMG400026475 | Cytosolic tRNA-Ala synthetase | 1807720.5 |
| ch03 | 43854705 | 43861472 | 43858089 | PGSC0003DMG400010123 | Dentin sialophosphoprotein | 1745573.5 |
| ch03 | 45428623 | 45435879 | 45432251 | PGSC0003DMG400009126 | Dipeptidyl peptidase IV | 171411 |
| ch03 | 44928819 | 44931676 | 44930248 | PGSC0003DMG400009101 | DNA binding protein | 673414.5 |
| ch03 | 46646919 | 46651119 | 46649019 | PGSC0003DMG400000575 | DNA mismatch repair muts family protein | 1045357 |
| ch03 | 47494514 | 47503012 | 47498763 | PGSC0003DMG400026493 | DNA-binding protein smubp-2 | 1895101 |
| ch03 | 47319516 | 47323406 | 47321461 | PGSC0003DMG400026487 | DNAJ heat shock N-terminal domain-containing protein | 1717799 |
| ch03 | 44179180 | 44187383 | 44183282 | PGSC0003DMG402009494 | DnaJ protein | 1420380.5 |
| ch03 | 45817646 | 45818926 | 45818286 | PGSC0003DMG400018541 | Dof zinc finger protein | 214624 |
| ch03 | 46816841 | 46820046 | 46818444 | PGSC0003DMG400000569 | Electron transporter | 1214781.5 |
| ch03 | 47394265 | 47394802 | 47394534 | PGSC0003DMG400026496 | Endonuclease/exonuclease/phosphatase family protein | 1790871.5 |
| ch03 | 47099905 | 47106067 | 47102986 | PGSC0003DMG402003156 | Exostosin family protein | 1499324 |
| ch03 | 45843121 | 45844847 | 45843984 | PGSC0003DMG400018518 | Fad NAD binding oxidoreductases | 240322 |
| ch03 | 45902446 | 45904168 | 45903307 | PGSC0003DMG400018537 | F-box domain containing protein | 299645 |
| ch03 | 47197328 | 47198464 | 47197896 | PGSC0003DMG400003149 | F-box protein family | 1594234 |
| ch03 | 44190073 | 44194303 | 44192188 | PGSC0003DMG401009494 | Ferric-chelate reductase | 1411474 |
| ch03 | 43661736 | 43664809 | 43663273 | PGSC0003DMG400010119 | Fibrillarin homolog | 1940389.5 |
| ch03 | 47314426 | 47315417 | 47314922 | PGSC0003DMG400026472 | Gamma-glutamylcyclotransferase | 1711259.5 |
| ch03 | 44477966 | 44480215 | 44479091 | PGSC0003DMG402019961 | Glossy1 protein | 1124571.5 |
| ch03 | 45731312 | 45733480 | 45732396 | PGSC0003DMG400018523 | Glucan endo-1,3-beta-glucosidase | 128734 |
| ch03 | 44071418 | 44078170 | 44074794 | PGSC0003DMG400009500 | Glutamine cyclotransferase | 1528868 |
| ch03 | 47424062 | 47430953 | 47427508 | PGSC0003DMG400026490 | Glutamine-rich protein | 1823845.5 |
| ch03 | 44067922 | 44069141 | 44068532 | PGSC0003DMG402009510 | Glycoside hydrolase, family 28 | 1535130.5 |
| ch03 | 47090594 | 47096240 | 47093417 | PGSC0003DMG401003156 | Glycosyltransferase, CAZy family GT2 | 1489755 |
| ch03 | 43813189 | 43815687 | 43814438 | PGSC0003DMG400010121 | GRAS10 | 1789224 |

| | | | | | | |
|---|---|---|---|---|---|---|
| ch03 | 46178186 | 46182940 | 46180563 | PGSC0003DMG400003212 | GRF domain class transcription factor | 576901 |
| ch03 | 46854624 | 46865779 | 46860202 | PGSC0003DMG400000537 | Guanosine-3',5'-bis(Diphosphate) 3'-pyrophosphohydrolase | 1256539.5 |
| ch03 | 43601021 | 43608131 | 43604576 | PGSC0003DMG400010161 | HCF106 | 1999086 |
| ch03 | 46013114 | 46014288 | 46013701 | PGSC0003DMG400018509 | HD-ZIP | 410039 |
| ch03 | 44107135 | 44110582 | 44108859 | PGSC0003DMG400009509 | Heat shock protein 83 | 1494803.5 |
| ch03 | 45060840 | 45066005 | 45063423 | PGSC0003DMG400009110 | Heat shock protein binding protein | 540239.5 |
| ch03 | 47153279 | 47158739 | 47156009 | PGSC0003DMG400003160 | Heat stress transcription factor A-1 | 1552347 |
| ch03 | 44154316 | 44157849 | 44156083 | PGSC0003DMG400009496 | Hemolysin | 1447579.5 |
| ch03 | 47585757 | 47589106 | 47587432 | PGSC0003DMG400026483 | HVA22 e | 1983769.5 |
| ch03 | 46804543 | 46806857 | 46805700 | PGSC0003DMG400000541 | Hydrogen peroxide-induced 1 | 1202038 |
| ch03 | 45014701 | 45021885 | 45018293 | PGSC0003DMG400009075 | Hydrolase | 585369 |
| ch03 | 43628236 | 43632818 | 43630527 | PGSC0003DMG400010118 | Hydroxyproline-rich glycoprotein family protein | 1973135 |
| ch03 | 45535460 | 45542309 | 45538885 | PGSC0003DMG400009085 | Hydroxyproline-rich glycoprotein GAS31 | 64777.5 |
| ch03 | 45826505 | 45829316 | 45827911 | PGSC0003DMG402018519 | Impaired sucrose induction 1 | 224248.5 |
| ch03 | 45709899 | 45714807 | 45712353 | PGSC0003DMG400018525 | Importin beta-1 | 108691 |
| ch03 | 47563557 | 47564906 | 47564232 | PGSC0003DMG400026481 | Inositol polyphosphate kinase | 1960569.5 |
| ch03 | 47232759 | 47234456 | 47233608 | PGSC0003DMG400003161 | Inositol-tetrakisphosphate 1-kinase | 1629945.5 |
| ch03 | 47123624 | 47132425 | 47128025 | PGSC0003DMG400003158 | Jumonji domain protein | 1524362.5 |
| ch03 | 44888869 | 44893015 | 44890942 | PGSC0003DMG400009070 | Kinesin | 712720 |
| ch03 | 45674908 | 45677904 | 45676406 | PGSC0003DMG400018528 | Kinesin light chain | 72744 |
| ch03 | 46188547 | 46195822 | 46192185 | PGSC0003DMG400003211 | Leucine Rich Repeat family protein | 588522.5 |
| ch03 | 45718209 | 45722293 | 45720251 | PGSC0003DMG400018544 | Luminal-binding protein 5 | 116589 |
| ch03 | 46233891 | 46235741 | 46234816 | PGSC0003DMG400003221 | Lung seven transmembrane receptor | 631154 |
| ch03 | 45157567 | 45160096 | 45158832 | PGSC0003DMG400009116 | Membrane receptor 1 | 444830.5 |
| ch03 | 44133491 | 44134628 | 44134060 | PGSC0003DMG400009507 | Metal ion binding protein | 1469602.5 |
| ch03 | 44906302 | 44914558 | 44910430 | PGSC0003DMG400009071 | Mitochondrial carrier protein | 693232 |
| ch03 | 47553314 | 47554113 | 47553714 | PGSC0003DMG400026480 | Mitotic-spindle organizing protein 1B | 1950051.5 |
| ch03 | 44751543 | 44757288 | 44754416 | PGSC0003DMG400009095 | Multidrug resistance-associated protein 1, 3 (Mrp1, 3), abc-transoprter | 849246.5 |
| ch03 | 47176453 | 47180747 | 47178600 | PGSC0003DMG400003148 | N-acetyltransferase | 1574938 |
| ch03 | 46918617 | 46923827 | 46921222 | PGSC0003DMG402000567 | NADH dehydrogenase [ubiquinone] 1 alpha subcomplex subunit 12 | 1317560 |
| ch03 | 46525122 | 46529422 | 46527272 | PGSC0003DMG400000555 | Nam 1 | 923610 |
| ch03 | 46472833 | 46475727 | 46474280 | PGSC0003DMG400000579 | Nascent polypeptide associated complex alpha subunit | 870618 |
| ch03 | 46403201 | 46404093 | 46403647 | PGSC0003DMG400000583 | NDR1/HIN1 | 799985 |
| ch03 | 47257523 | 47264798 | 47261161 | PGSC0003DMG400003162 | NERD domain containing protein | 1657498.5 |
| ch03 | 43781751 | 43785196 | 43783474 | PGSC0003DMG400010120 | Nodulation receptor kinase | 1820188.5 |

| | | | | | | |
|---|---|---|---|---|---|---|
| ch03 | 44980011 | 44981624 | 44980818 | PGSC0003DMG400009073 | Nodulin MtN3 family protein | 622844.5 |
| ch03 | 45684752 | 45692019 | 45688386 | PGSC0003DMG400018527 | Nucleic acid binding protein | 84723.5 |
| ch03 | 45855441 | 45859324 | 45857383 | PGSC0003DMG400018539 | Oligopeptide transporter OPT family | 253720.5 |
| ch03 | 47046944 | 47048711 | 47047828 | PGSC0003DMG402003142 | Orf147a protein | 1444165.5 |
| ch03 | 47249415 | 47250055 | 47249735 | PGSC0003DMG400003165 | ORF41g | 1646073 |
| ch03 | 46673480 | 46674670 | 46674075 | PGSC0003DMG400000573 | Osmotin | 1070413 |
| ch03 | 47531214 | 47533611 | 47532413 | PGSC0003DMG400026494 | Palmitoyl-acyl carrier protein thioesterase | 1928750.5 |
| ch03 | 45695259 | 45699252 | 45697256 | PGSC0003DMG400018546 | PDE225/PTAC7 | 93593.5 |
| ch03 | 45385282 | 45389409 | 45387346 | PGSC0003DMG400009081 | Pectin methylesterase 1 | 216316.5 |
| ch03 | 45473992 | 45476391 | 45475192 | PGSC0003DMG400009084 | Pentatricopeptide repeat-containing protein | 128470.5 |
| ch03 | 46473742 | 46476367 | 46475055 | PGSC0003DMG400000559 | Peroxidase 5 | 871392.5 |
| ch03 | 46614171 | 46615243 | 46614707 | PGSC0003DMG400000576 | PGPS/D3 | 1011045 |
| ch03 | 46317594 | 46322270 | 46319932 | PGSC0003DMG400003208 | Phosphatidylcholine transfer protein | 716270 |
| ch03 | 43921044 | 43922498 | 43921771 | PGSC0003DMG400010127 | Plastid division regulator MinD | 1681891 |
| ch03 | 46934640 | 46936686 | 46935663 | PGSC0003DMG400000566 | Pm52 protein | 1332001 |
| ch03 | 44062395 | 44071156 | 44066776 | PGSC0003DMG401009510 | Polygalacturonase | 1536886.5 |
| ch03 | 45002385 | 45010618 | 45006502 | PGSC0003DMG400009106 | Polypyrimidine tract binding protein | 597160.5 |
| ch03 | 46294400 | 46302826 | 46298613 | PGSC0003DMG400003218 | Pre-mRNA splicing factor | 694951 |
| ch03 | 44788920 | 44796025 | 44792473 | PGSC0003DMG400009067 | Proline-rich cell wall protein | 811189.5 |
| ch03 | 47065338 | 47065851 | 47065595 | PGSC0003DMG401003154 | Proteasome-activating nucleotidase | 1461932.5 |
| ch03 | 45653256 | 45657804 | 45655530 | PGSC0003DMG400018548 | Protein ABIL2 | 51868 |
| ch03 | 44794980 | 44801906 | 44798443 | PGSC0003DMG400009096 | Protein dimerization | 805219 |
| ch03 | 47578499 | 47579122 | 47578811 | PGSC0003DMG400026482 | Protein HVA22 | 1975148.5 |
| ch03 | 47463695 | 47467667 | 47465681 | PGSC0003DMG400026492 | Protein kinase family protein | 1862019 |
| ch03 | 45699362 | 45703149 | 45701256 | PGSC0003DMG400018545 | Protein phosphatase | 97593.5 |
| ch03 | 45072898 | 45080669 | 45076784 | PGSC0003DMG400009111 | Protein phosphatase 2C | 526878.5 |
| ch03 | 45121502 | 45125081 | 45123292 | PGSC0003DMG400009112 | Protein phosphatase 2C 8 | 480370.5 |
| ch03 | 46684803 | 46689118 | 46686961 | PGSC0003DMG400000572 | Protein translocase | 1083298.5 |
| ch03 | 46394146 | 46399285 | 46396716 | PGSC0003DMG400000584 | Pseudo-response regulator 5 | 793053.5 |
| ch03 | 46338696 | 46341017 | 46339857 | PGSC0003DMG400000587 | PTAC15 | 736194.5 |
| ch03 | 44654298 | 44661981 | 44658140 | PGSC0003DMG400006590 | Pyruvate kinase | 945522.5 |
| ch03 | 45980442 | 45988412 | 45984427 | PGSC0003DMG400018512 | Rab GTPase activator | 380765 |
| ch03 | 45161379 | 45164145 | 45162762 | PGSC0003DMG400009078 | Receptor serine/threonine kinase | 440900 |
| ch03 | 43665908 | 43668302 | 43667105 | PGSC0003DMG400010163 | Retinoblastoma-binding protein | 1936557 |
| ch03 | 47296189 | 47300760 | 47298475 | PGSC0003DMG400026470 | Ribokinase | 1694812.5 |

| | | | | | | |
|---|---|---|---|---|---|---|
| ch03 | 45164232 | 45168275 | 45166254 | PGSC0003DMG400009117 | Ring finger protein | 437408.5 |
| ch03 | 44970721 | 44975407 | 44973064 | PGSC0003DMG400009072 | RNA polymerase sigma factor rpoD | 630598 |
| ch03 | 46254552 | 46262238 | 46258395 | PGSC0003DMG402003209 | RNA-binding protein | 654733 |
| ch03 | 45297991 | 45302323 | 45300157 | PGSC0003DMG400009123 | Salt tolerance protein 5 | 303505 |
| ch03 | 46101962 | 46102626 | 46102294 | PGSC0003DMG400003228 | SAUR family protein | 498632 |
| ch03 | 46379888 | 46380220 | 46380054 | PGSC0003DMG402000585 | Second of four adjacent putative subtilase family> | 776392 |
| ch03 | 43944960 | 43945792 | 43945376 | PGSC0003DMG400010128 | Serine protease inhibitor 7 | 1658286 |
| ch03 | 46148789 | 46157600 | 46153195 | PGSC0003DMG400003213 | Serine/threonine protein kinase pk23 | 549532.5 |
| ch03 | 46586643 | 46589142 | 46587893 | PGSC0003DMG400000578 | Serine-threonine protein kinase, plant-type | 984230.5 |
| ch03 | 46610270 | 46611272 | 46610771 | PGSC0003DMG400000552 | Serine-type endopeptidase inhibitor | 1007109 |
| ch03 | 45780159 | 45789450 | 45784805 | PGSC0003DMG400018520 | SET domain protein | 181142.5 |
| ch03 | 45591994 | 45595010 | 45593502 | PGSC0003DMG400009086 | SF16 | 10160 |
| ch03 | 44340875 | 44345864 | 44343370 | PGSC0003DMG400020884 | SGT1 | 1260292.5 |
| ch03 | 47449204 | 47458885 | 47454045 | PGSC0003DMG400026491 | Sigma factor | 1850382.5 |
| ch03 | 46254502 | 46257461 | 46255982 | PGSC0003DMG400003219 | Small heat shock protein, chloroplastic | 652319.5 |
| ch03 | 45603558 | 45603887 | 45603723 | PGSC0003DMG401018552 | Soluble starch synthase 1, chloroplastic/amyloplastic | 60.5 |
| ch03 | 45114443 | 45121294 | 45117869 | PGSC0003DMG400009077 | Spermine synthase | 485793.5 |
| ch03 | 45217824 | 45227638 | 45222731 | PGSC0003DMG400009079 | Splicing factor 3a | 380931 |
| ch03 | 44673248 | 44675884 | 44674566 | PGSC0003DMG400006592 | ST6-40 | 929096 |
| ch03 | 47596864 | 47599863 | 47598364 | PGSC0003DMG400026485 | Steroleosin | 1994701.5 |
| ch03 | 46359335 | 46359952 | 46359644 | PGSC0003DMG401000586 | Subtilase family protein | 755981.5 |
| ch03 | 46924696 | 46933824 | 46929260 | PGSC0003DMG401000567 | Sugar transporter | 1325598 |
| ch03 | 47330926 | 47334705 | 47332816 | PGSC0003DMG400026489 | Surface antigen protein | 1729153.5 |
| ch03 | 45464734 | 45467864 | 45466299 | PGSC0003DMG400009083 | Surfeit locus protein 5 family protein | 137363 |
| ch03 | 44586616 | 44590410 | 44588513 | PGSC0003DMG400019960 | Tellurite resistance protein tehA | 1015149 |
| ch03 | 45733931 | 45736863 | 45735397 | PGSC0003DMG400018522 | Thylakoid lumenal 17.4 kDa protein, chloroplast | 131735 |
| ch03 | 45645461 | 45647253 | 45646357 | PGSC0003DMG400018531 | Transcription factor | 42695 |
| ch03 | 47487516 | 47493633 | 47490575 | PGSC0003DMG400026478 | Transcription regulator | 1886912.5 |
| ch03 | 46418857 | 46419888 | 46419373 | PGSC0003DMG400044039 | Transparent testa glabra 1 | 815710.5 |
| ch03 | 47437840 | 47440384 | 47439112 | PGSC0003DMG400026476 | Transporter | 1835450 |
| ch03 | 45041922 | 45043941 | 45042932 | PGSC0003DMG400009109 | Trehalose-6-phosphate synthase | 560730.5 |
| ch03 | 45344129 | 45345954 | 45345042 | PGSC0003DMG400009080 | Triacylglycerol lipase | 258620.5 |
| ch03 | 45390011 | 45396657 | 45393334 | PGSC0003DMG400009125 | Ubiquitin | 210328 |
| ch03 | 44824641 | 44828570 | 44826606 | PGSC0003DMG400009069 | Ubiquitin carrier protein | 777056.5 |
| ch03 | 46005659 | 46007926 | 46006793 | PGSC0003DMG400018533 | Ubiquitin fusion protein | 403130.5 |

| | | | | | | |
|---|---|---|---|---|---|---|
| ch03 | 45477271 | 45481764 | 45479518 | PGSC0003DMG400009129 | Ubiquitin ligase SINAT3 | 124144.5 |
| ch03 | 45859509 | 45865161 | 45862335 | PGSC0003DMG400018516 | Ubiquitin-protein ligase | 258673 |
| ch03 | 43922786 | 43929929 | 43926358 | PGSC0003DMG400010168 | UDP-N-acetylmuramate-alanine ligase | 1677304.5 |
| ch03 | 45628226 | 45632156 | 45630191 | PGSC0003DMG400018532 | Ulp1 protease family, C-terminal catalytic domain containing protein | 26529 |
| ch03 | 46694501 | 46695229 | 46694865 | PGSC0003DMG400000547 | UP-9A | 1091203 |
| ch03 | 45741087 | 45746014 | 45743551 | PGSC0003DMG400018521 | WD-repeat protein | 139888.5 |
| ch03 | 44358543 | 44360403 | 44359473 | PGSC0003DMG400020888 | White-brown-complex ABC transporter family | 1244189 |
| ch03 | 45454755 | 45463045 | 45458900 | PGSC0003DMG400009082 | WREBP-2 protein | 144762 |
| ch03 | 45836974 | 45837675 | 45837325 | PGSC0003DMG400045376 | WRKY transcription factor | 233662.5 |
| ch03 | 45831532 | 45832311 | 45831922 | PGSC0003DMG400044842 | WRKY transcription factor 22 | 228259.5 |
| ch03 | 44309485 | 44310468 | 44309977 | PGSC0003DMG400039175 | WRKY transcription factor 27 | 1293685.5 |
| ch03 | 45835336 | 45835857 | 45835597 | PGSC0003DMG400034476 | WRKY33 | 231934.5 |
| ch03 | 45963294 | 45967167 | 45965231 | PGSC0003DMG400018535 | Yellow stripe | 361568.5 |

| chr | start | end | mid | ID | Name | hit_dist |
|---|---|---|---|---|---|---|
| ch03 | 55248140 | 55252302 | 55250221 | PGSC0003DMG400024644 | 101 kDa heat shock protein | 409030 |
| ch03 | 54368432 | 54374033 | 54371233 | PGSC0003DMG400024503 | 1-deoxy-D-xylulose-5-phosphate reductoisomerase | 1288018.5 |
| ch03 | 55212848 | 55216845 | 55214847 | PGSC0003DMG400024641 | 24 kDa seed maturation protein | 444404.5 |
| ch03 | 55343022 | 55344967 | 55343995 | PGSC0003DMG400024546 | 40S ribosomal protein S19 | 315256.5 |
| ch03 | 56080206 | 56081282 | 56080744 | PGSC0003DMG400019538 | 42kDa chitin-binding protein | 421493 |
| ch03 | 57566692 | 57569946 | 57568319 | PGSC0003DMG400014223 | 4-coumarate--CoA ligase 2 | 1909068 |
| ch03 | 57166577 | 57170997 | 57168787 | PGSC0003DMG400014202 | 50S ribosomal protein L3 | 1509536 |
| ch03 | 56963696 | 56967940 | 56965818 | PGSC0003DMG400014191 | 60S ribosomal protein L7 | 1306567 |
| ch03 | 55116413 | 55120021 | 55118217 | PGSC0003DMG400024634 | 70 kDa subunit of replication protein A | 541034 |
| ch03 | 54546628 | 54553861 | 54550245 | PGSC0003DMG402024509 | Acetyl-CoA synthetase | 1109006.5 |
| ch03 | 57394342 | 57396693 | 57395518 | PGSC0003DMG400014257 | Acireductone dioxygenase | 1736266.5 |
| ch03 | 53703782 | 53706897 | 53705340 | PGSC0003DMG400024577 | Actin binding protein | 1953911.5 |
| ch03 | 56016933 | 56022364 | 56019649 | PGSC0003DMG400019513 | Activating signal cointegrator | 360397.5 |
| ch03 | 54102305 | 54107157 | 54104731 | PGSC0003DMG400024490 | Aldehyde dehydrogenase | 1554520 |
| ch03 | 57283003 | 57283575 | 57283289 | PGSC0003DMG400014142 | Allergen Pru p 1.06B | 1624038 |
| ch03 | 55783142 | 55785146 | 55784144 | PGSC0003DMG400019505 | Alpha-1,2-fucosyltransferase | 124893 |
| ch03 | 53775263 | 53778132 | 53776698 | PGSC0003DMG400024584 | Alpha-n-acetylglucosaminidase | 1882553.5 |
| ch03 | 57581455 | 57584689 | 57583072 | PGSC0003DMG400014224 | Amino acid binding protein | 1923821 |
| ch03 | 57184598 | 57186764 | 57185681 | PGSC0003DMG400014203 | Amino acid transporter | 1526430 |
| ch03 | 53757940 | 53766241 | 53762091 | PGSC0003DMG400024582 | Aminoaldehyde dehydrogenase 2 | 1897160.5 |
| ch03 | 56490832 | 56494056 | 56492444 | PGSC0003DMG400000612 | AP2 domain-containing protein AP29 | 833193 |
| ch03 | 57470351 | 57474972 | 57472662 | PGSC0003DMG400014157 | AP2 domain-containing transcription factor | 1813410.5 |
| ch03 | 56522932 | 56524108 | 56523520 | PGSC0003DMG400000636 | AP2 transcription factor | 864269 |
| ch03 | 56959060 | 56962258 | 56960659 | PGSC0003DMG400014123 | Aquaporin, MIP family, NIP subfamily | 1301408 |
| ch03 | 57458317 | 57460129 | 57459223 | PGSC0003DMG400014156 | Aspartic proteinase nepenthesin-1 | 1799972 |
| ch03 | 56404492 | 56409141 | 56406817 | PGSC0003DMG400000627 | AtEB1b | 747565.5 |
| ch03 | 55126898 | 55130102 | 55128500 | PGSC0003DMG400024539 | ATEXO70E2 | 530751 |
| ch03 | 57510170 | 57517119 | 57513645 | PGSC0003DMG402014218 | ATP binding / kinase/ protein kinase/ protein serine/threonine kinase/ protein tyrosine kinase | 1854393.5 |
| ch03 | 55596037 | 55603079 | 55599558 | PGSC0003DMG400024559 | ATP binding protein | 59693 |
| ch03 | 55148871 | 55153448 | 55151160 | PGSC0003DMG400024638 | ATP synthase subunit gamma, mitochondrial | 508091.5 |
| ch03 | 53683860 | 53686179 | 53685020 | PGSC0003DMG400024466 | ATP-binding cassette transporter | 1974231.5 |
| ch03 | 54355980 | 54363363 | 54359672 | PGSC0003DMG400024502 | AtPex14p | 1299579.5 |
| ch03 | 53717816 | 53719670 | 53718743 | PGSC0003DMG400024468 | B12D protein | 1940508 |

| | | | | | | |
|---|---|---|---|---|---|---|
| ch03 | 54027001 | 54035855 | 54031428 | PGSC0003DMG400024484 | BAH domain-containing protein | 1627823 |
| ch03 | 55114549 | 55115862 | 55115206 | PGSC0003DMG400024538 | Basic 7S globulin 2 small subunit | 544045.5 |
| ch03 | 57237010 | 57238131 | 57237571 | PGSC0003DMG402014139 | Beta transducin | 1578319.5 |
| ch03 | 55544743 | 55548544 | 55546644 | PGSC0003DMG400024661 | Brassinosteroid LRR receptor kinase | 112607.5 |
| ch03 | 54823953 | 54829101 | 54826527 | PGSC0003DMG403024620 | BSD domain containing protein | 832724 |
| ch03 | 53828218 | 53829118 | 53828668 | PGSC0003DMG402024472 | C2H2L domain class transcription factor | 1830583 |
| ch03 | 57287784 | 57292017 | 57289901 | PGSC0003DMG400014207 | Calcineurin | 1630649.5 |
| ch03 | 55872209 | 55873150 | 55872680 | PGSC0003DMG400019527 | Calcium ion binding protein | 213428.5 |
| ch03 | 54508886 | 54509597 | 54509242 | PGSC0003DMG400024604 | Calmodulin | 1150009.5 |
| ch03 | 53891239 | 53893065 | 53892152 | PGSC0003DMG400024477 | Calmodulin binding protein | 1767099 |
| ch03 | 53911132 | 53916455 | 53913794 | PGSC0003DMG400024479 | Calmodulin-binding protein | 1745457.5 |
| ch03 | 56506793 | 56508408 | 56507601 | PGSC0003DMG400000634 | CAPIP2 | 848349.5 |
| ch03 | 55564054 | 55569528 | 55566791 | PGSC0003DMG401024557 | Carbamoyl phosphate synthase small subunit | 92460 |
| ch03 | 55451344 | 55451992 | 55451668 | PGSC0003DMG400024653 | Catalytic/ oxidoreductase, acting on NADH or NADPH | 207583 |
| ch03 | 56926874 | 56928354 | 56927614 | PGSC0003DMG400014121 | Ccr4-associated factor | 1268363 |
| ch03 | 53719600 | 53725446 | 53722523 | PGSC0003DMG400024579 | Cell division cycle | 1936728 |
| ch03 | 54554175 | 54558025 | 54556100 | PGSC0003DMG403024509 | Cell growth defect factor | 1103151 |
| ch03 | 56045150 | 56046685 | 56045918 | PGSC0003DMG400019516 | Chitin-binding lectin | 386666.5 |
| ch03 | 56065422 | 56065940 | 56065681 | PGSC0003DMG400019517 | Chitin-binding lectin 1 | 406430 |
| ch03 | 55862891 | 55864115 | 55863503 | PGSC0003DMG400019508 | Chlorophyll a/b binding protein | 204252 |
| ch03 | 54472475 | 54477646 | 54475061 | PGSC0003DMG400024505 | Chromosome-associated kinesin KIF4A | 1184190.5 |
| ch03 | 56814215 | 56816945 | 56815580 | PGSC0003DMG400019825 | Cinnamoyl CoA reductase | 1156329 |
| ch03 | 54963884 | 54966403 | 54965144 | PGSC0003DMG400024628 | COBRA 3 | 694107.5 |
| ch03 | 56746680 | 56752798 | 56749739 | PGSC0003DMG400019821 | Cyclic nucleotide-gated ion channel | 1090488 |
| ch03 | 54061000 | 54066772 | 54063886 | PGSC0003DMG400024591 | Cyclic nucleotide-gated ion channel 16 | 1595365 |
| ch03 | 54992291 | 54994660 | 54993476 | PGSC0003DMG400024630 | Cytochrome P450 | 665775.5 |
| ch03 | 54853249 | 54860120 | 54856685 | PGSC0003DMG400024525 | Dead box ATP-dependent RNA helicase | 802566.5 |
| ch03 | 57279271 | 57282389 | 57280830 | PGSC0003DMG400014141 | DEAD-box protein abstrakt | 1621579 |
| ch03 | 57019240 | 57025146 | 57022193 | PGSC0003DMG400014194 | Defective in cullin neddylation protein | 1362942 |
| ch03 | 54116300 | 54119783 | 54118042 | PGSC0003DMG400024492 | Deleted in split hand/splt foot protein 1 | 1541209.5 |
| ch03 | 56602125 | 56605038 | 56603582 | PGSC0003DMG400000639 | Delta 9 desaturase | 944330.5 |
| ch03 | 55347770 | 55354797 | 55351284 | PGSC0003DMG400024547 | Diacylglycerol kinase, theta | 307967.5 |
| ch03 | 55513446 | 55517088 | 55515267 | PGSC0003DMG400024556 | Dimethylaniline monooxygenase | 143984 |
| ch03 | 55495567 | 55498080 | 55496824 | PGSC0003DMG400024554 | DNA binding protein | 162427.5 |
| ch03 | 56769711 | 56776369 | 56773040 | PGSC0003DMG400019819 | DNA-3-methyladenine glycosylase | 1113789 |

| | | | | | | |
|---|---|---|---|---|---|---|
| ch03 | 56379966 | 56383979 | 56381973 | PGSC0003DMG400000599 | DNA-binding protein | 722721.5 |
| ch03 | 57385089 | 57386521 | 57385805 | PGSC0003DMG400014210 | DnaJ protein | 1726554 |
| ch03 | 55882564 | 55885296 | 55883930 | PGSC0003DMG400019528 | Dof zinc finger protein | 224679 |
| ch03 | 54482662 | 54484036 | 54483349 | PGSC0003DMG402024603 | DR1 | 1175902 |
| ch03 | 53732690 | 53737067 | 53734879 | PGSC0003DMG402024469 | E2F | 1924372.5 |
| ch03 | 55379182 | 55379793 | 55379488 | PGSC0003DMG401024649 | EMB2733/ESP3 (EMBRYO DEFECTIVE 2733) | 279763.5 |
| ch03 | 55919907 | 55921627 | 55920767 | PGSC0003DMG400019531 | EMB514 | 261516 |
| ch03 | 53823505 | 53827469 | 53825487 | PGSC0003DMG401024472 | Endonuclease III | 1833764 |
| ch03 | 54580617 | 54585154 | 54582886 | PGSC0003DMG401024510 | Enolase | 1076365.5 |
| ch03 | 57613695 | 57621016 | 57617356 | PGSC0003DMG400014225 | ERD1 protein, chloroplast | 1958104.5 |
| ch03 | 54533650 | 54534364 | 54534007 | PGSC0003DMG400024606 | ERF transcription factor | 1125244 |
| ch03 | 57039987 | 57041187 | 57040587 | PGSC0003DMG400014196 | Ethylene response factor | 1381336 |
| ch03 | 55575040 | 55577312 | 55576176 | PGSC0003DMG400024558 | Eukaryotic translation initiation factor 5A-5 | 83075 |
| ch03 | 55520373 | 55524372 | 55522373 | PGSC0003DMG400024658 | Excision repair cross-complementing 1 ercc1 | 136878.5 |
| ch03 | 55745709 | 55749972 | 55747841 | PGSC0003DMG400019519 | Exonuclease | 88589.5 |
| ch03 | 55854740 | 55856726 | 55855733 | PGSC0003DMG400019507 | Expansin | 196482 |
| ch03 | 54537065 | 54540400 | 54538733 | PGSC0003DMG400024508 | Extracellular Ca2+ sensing receptor | 1120518.5 |
| ch03 | 53797414 | 53798920 | 53798167 | PGSC0003DMG400024470 | F-box and wd40 domain protein | 1861084 |
| ch03 | 57123593 | 57125091 | 57124342 | PGSC0003DMG400014133 | F-box family protein | 1465091 |
| ch03 | 57074634 | 57076797 | 57075716 | PGSC0003DMG400014130 | F-box/LRR-repeat protein | 1416464.5 |
| ch03 | 55232191 | 55236265 | 55234228 | PGSC0003DMG400024643 | Flavonoid 3'-monooxygenase | 425023 |
| ch03 | 54865153 | 54866555 | 54865854 | PGSC0003DMG400024622 | Flavonol 4'-sulfotransferase | 793397 |
| ch03 | 56323681 | 56324169 | 56323925 | PGSC0003DMG400000593 | Flavonol synthase/flavanone 3-hydroxylase | 664674 |
| ch03 | 57113395 | 57115435 | 57114415 | PGSC0003DMG400014200 | Flotillin-1 | 1455164 |
| ch03 | 57323252 | 57329559 | 57326406 | PGSC0003DMG400014146 | Formamidopyrimidine-DNA glycosylase | 1667154.5 |
| ch03 | 55615061 | 55624395 | 55619728 | PGSC0003DMG400024560 | Formin 20 | 39523 |
| ch03 | 57381282 | 57384733 | 57383008 | PGSC0003DMG400014151 | GAG1At protein | 1723756.5 |
| ch03 | 55943853 | 55944385 | 55944119 | PGSC0003DMG402019533 | Gasa4 | 284868 |
| ch03 | 55904950 | 55906678 | 55905814 | PGSC0003DMG400019510 | Geranylgeranyl diphosphate reductase, chloroplastic | 246563 |
| ch03 | 55943853 | 55945734 | 55944794 | PGSC0003DMG401019533 | Gip1 | 285542.5 |
| ch03 | 55220548 | 55224682 | 55222615 | PGSC0003DMG400024642 | Glucan endo-1,3-beta-glucosidase 3 | 436636 |
| ch03 | 56004967 | 56005756 | 56005362 | PGSC0003DMG400019540 | Glutathione s-transferase | 346110.5 |
| ch03 | 57309534 | 57312556 | 57311045 | PGSC0003DMG400014144 | Glycerol kinase | 1651794 |
| ch03 | 56502402 | 56502712 | 56502557 | PGSC0003DMG400000633 | Glycoside hydrolase, family 28 | 843306 |
| ch03 | 57427581 | 57432248 | 57429915 | PGSC0003DMG400014153 | Glycosylphosphatidylinositol anchor biosynthesis protein 11 | 1770663.5 |

| | | | | | | |
|---|---|---|---|---|---|---|
| ch03 | 55668600 | 55672390 | 55670495 | PGSC0003DMG400024667 | Glycosyltransferase | 11244 |
| ch03 | 54867980 | 54873469 | 54870725 | PGSC0003DMG400024623 | Glycosyltransferase QUASIMODO1 | 788526.5 |
| ch03 | 54125552 | 54128245 | 54126899 | PGSC0003DMG400024594 | Glycosyltransferase UGT90A7 | 1532352.5 |
| ch03 | 53841278 | 53844424 | 53842851 | PGSC0003DMG400024474 | Gonadotropin beta chain | 1816400 |
| ch03 | 54934288 | 54936181 | 54935235 | PGSC0003DMG400024627 | GRP 2 | 724016.5 |
| ch03 | 56586492 | 56587506 | 56586999 | PGSC0003DMG400000637 | GTP binding protein | 927748 |
| ch03 | 54625568 | 54632815 | 54629192 | PGSC0003DMG400024512 | H/ACA ribonucleoprotein complex subunit | 1030059.5 |
| ch03 | 55540500 | 55542597 | 55541549 | PGSC0003DMG400024660 | HCG23534, partial | 117702.5 |
| ch03 | 55167492 | 55170026 | 55168759 | PGSC0003DMG400024541 | Heat shock protein | 490492 |
| ch03 | 55785591 | 55789344 | 55787468 | PGSC0003DMG400019522 | Heat shock protein binding protein | 128216.5 |
| ch03 | 53878612 | 53879610 | 53879111 | PGSC0003DMG400024476 | Heat-shock protein | 1780140 |
| ch03 | 55453167 | 55454136 | 55453652 | PGSC0003DMG401024654 | Heparanase 1 | 205599.5 |
| ch03 | 55453167 | 55458448 | 55455808 | PGSC0003DMG402024654 | Heparanase-2 | 203443.5 |
| ch03 | 56593561 | 56594955 | 56594258 | PGSC0003DMG400000638 | Histidine-containing phosphotransfer protein | 935007 |
| ch03 | 55170152 | 55172782 | 55171467 | PGSC0003DMG400024639 | Histone deacetylase | 487784 |
| ch03 | 54329272 | 54335041 | 54332157 | PGSC0003DMG400024500 | HT1 protein kinase | 1327094.5 |
| ch03 | 57404623 | 57408393 | 57406508 | PGSC0003DMG400014152 | Hydroxycinnamoyl transferase | 1747257 |
| ch03 | 57293005 | 57297878 | 57295442 | PGSC0003DMG400014208 | Inorganic pyrophosphatase | 1636190.5 |
| ch03 | 54072511 | 54073496 | 54073004 | PGSC0003DMG400024488 | IS10 transposase | 1586247.5 |
| ch03 | 56777004 | 56780104 | 56778554 | PGSC0003DMG400019824 | JA-induced WRKY protein | 1119303 |
| ch03 | 57164954 | 57166123 | 57165539 | PGSC0003DMG400014201 | Jp18 | 1506287.5 |
| ch03 | 55646289 | 55648550 | 55647420 | PGSC0003DMG400024665 | Kinase | 11831.5 |
| ch03 | 57368545 | 57370994 | 57369770 | PGSC0003DMG400014150 | Kinase family protein | 1710518.5 |
| ch03 | 54348113 | 54351370 | 54349742 | PGSC0003DMG400024501 | Kinesin light chain | 1309509.5 |
| ch03 | 56476942 | 56478370 | 56477656 | PGSC0003DMG400000631 | Lactoylglutathione lyase | 818405 |
| ch03 | 54860582 | 54863323 | 54861953 | PGSC0003DMG400024526 | L-asparaginase | 797298.5 |
| ch03 | 56420226 | 56421126 | 56420676 | PGSC0003DMG400000628 | Late embryogenesis abundant protein | 761425 |
| ch03 | 55408732 | 55414637 | 55411685 | PGSC0003DMG401024548 | Leucine-rich repeat containing protein | 247566.5 |
| ch03 | 57078465 | 57081047 | 57079756 | PGSC0003DMG400014131 | Leucine-rich repeat family protein | 1420505 |
| ch03 | 54926596 | 54932614 | 54929605 | PGSC0003DMG400024527 | LIGULELESS1 protein | 729646 |
| ch03 | 56508806 | 56513240 | 56511023 | PGSC0003DMG400000635 | Longin | 851772 |
| ch03 | 54879384 | 54886641 | 54883013 | PGSC0003DMG400024625 | MADS box protein | 776238.5 |
| ch03 | 55865548 | 55867778 | 55866663 | PGSC0003DMG400019525 | MADS-box family protein | 207412 |
| ch03 | 54896515 | 54901975 | 54899245 | PGSC0003DMG400024626 | MADS-box protein 1 | 760006 |
| ch03 | 57285513 | 57286258 | 57285886 | PGSC0003DMG400014143 | Major allergen Pru ar | 1626634.5 |

| | | | | | | |
|---|---|---|---|---|---|---|
| ch03 | 53751474 | 53756993 | 53754234 | PGSC0003DMG402024581 | Mannose-1-phosphate guanyltransferase | 1905017.5 |
| ch03 | 53830836 | 53833876 | 53832356 | PGSC0003DMG401024473 | Mannosyl-oligosaccharide 1,2-alpha-mannosidase IA | 1826895 |
| ch03 | 55924195 | 55930016 | 55927106 | PGSC0003DMG400019512 | Membrane associated ring finger 1,8 | 267854.5 |
| ch03 | 57498040 | 57503305 | 57500673 | PGSC0003DMG400014159 | Membrane protein PB1A10.07c | 1841421.5 |
| ch03 | 53799509 | 53804520 | 53802015 | PGSC0003DMG400024585 | Methyltransferase gidB | 1857236.5 |
| ch03 | 54835543 | 54840071 | 54837807 | PGSC0003DMG400024524 | Microtubule-associated protein | 821444 |
| ch03 | 53664130 | 53668654 | 53666392 | PGSC0003DMG400024575 | Microtubule-associated protein TORTIFOLIA1 | 1992859 |
| ch03 | 54592800 | 54599988 | 54596394 | PGSC0003DMG400024511 | MIS12 homologue | 1062857 |
| ch03 | 56988976 | 56991846 | 56990411 | PGSC0003DMG400014192 | Mitochondrial glycoprotein family protein | 1331160 |
| ch03 | 55108954 | 55112010 | 55110482 | PGSC0003DMG400024536 | Mitochondrial import receptor subunit TOM5 homolog | 548769 |
| ch03 | 57362420 | 57364406 | 57363413 | PGSC0003DMG400014149 | Multidrug Resistance associated Protein 1 | 1704162 |
| ch03 | 55020280 | 55031726 | 55026003 | PGSC0003DMG400024532 | Multidrug resistance protein | 633248 |
| ch03 | 55984819 | 55986615 | 55985717 | PGSC0003DMG400019535 | MYB domain class transcription factor | 326466 |
| ch03 | 55559293 | 55561461 | 55560377 | PGSC0003DMG400024662 | Myosin heavy chain | 98874 |
| ch03 | 55815778 | 55817714 | 55816746 | PGSC0003DMG400019523 | NAC domain-containing protein 21/22 | 157495 |
| ch03 | 54460915 | 54461706 | 54461311 | PGSC0003DMG400045663 | NAC transcription factor | 1197940.5 |
| ch03 | 56755917 | 56757865 | 56756891 | PGSC0003DMG400019822 | N-acetyltransferase | 1097640 |
| ch03 | 55907758 | 55912183 | 55909971 | PGSC0003DMG400019511 | NAD-malate dehydrogenase | 250719.5 |
| ch03 | 57110681 | 57112638 | 57111660 | PGSC0003DMG400014199 | Nodulin | 1452408.5 |
| ch03 | 56577846 | 56578716 | 56578281 | PGSC0003DMG400000621 | NtEIG-A1 protein | 919030 |
| ch03 | 56499164 | 56501938 | 56500551 | PGSC0003DMG400000613 | Nucleic acid binding protein | 841300 |
| ch03 | 57026302 | 57034757 | 57030530 | PGSC0003DMG400014195 | Nucleoporin | 1371278.5 |
| ch03 | 56571862 | 56574223 | 56573043 | PGSC0003DMG400000619 | Nucleosome-binding protein | 913791.5 |
| ch03 | 54316402 | 54322812 | 54319607 | PGSC0003DMG400024499 | Para-hydroxybenzoate--polyprenyltransferase | 1339644 |
| ch03 | 53957004 | 53961462 | 53959233 | PGSC0003DMG400024586 | PBng143 | 1700018 |
| ch03 | 53973615 | 53976517 | 53975066 | PGSC0003DMG400024482 | Pentatricopeptide repeat protein | 1684185 |
| ch03 | 55752344 | 55753969 | 55753157 | PGSC0003DMG400019503 | Pentatricopeptide repeat-containing protein | 93905.5 |
| ch03 | 53992509 | 53999728 | 53996119 | PGSC0003DMG400024588 | Permease I | 1663132.5 |
| ch03 | 57527570 | 57531577 | 57529574 | PGSC0003DMG400014219 | Phosphate abc transporter | 1870322.5 |
| ch03 | 54266590 | 54268781 | 54267686 | PGSC0003DMG400024494 | Phosphoglycerate mutase | 1391565.5 |
| ch03 | 56535683 | 56540302 | 56537993 | PGSC0003DMG400000614 | Phospholipase PLDa1 | 878741.5 |
| ch03 | 56672676 | 56681324 | 56677000 | PGSC0003DMG400000644 | Phox domain-containing protein | 1017749 |
| ch03 | 57050846 | 57054023 | 57052435 | PGSC0003DMG400014128 | Plasma membrane proton ATPase | 1393183.5 |
| ch03 | 53970890 | 53973411 | 53972151 | PGSC0003DMG400024481 | Pollen specific LIM domain-containing protein | 1687100.5 |
| ch03 | 56461474 | 56466007 | 56463741 | PGSC0003DMG400000608 | Polygalacturonase | 804489.5 |

| | | | | | | |
|---|---|---|---|---|---|---|
| ch03 | 54259488 | 54260780 | 54260134 | PGSC0003DMG400024599 | Polygalacturonase-1 non-catalytic subunit beta | 1399117 |
| ch03 | 55132684 | 55134160 | 55133422 | PGSC0003DMG400024636 | Presenilin | 525829 |
| ch03 | 57270931 | 57278996 | 57274964 | PGSC0003DMG400014140 | Prli-interacting factor l | 1615712.5 |
| ch03 | 57171533 | 57179260 | 57175397 | PGSC0003DMG400014136 | Prolyl-tRNA synthetase | 1516145.5 |
| ch03 | 54691474 | 54694326 | 54692900 | PGSC0003DMG401024517 | Protease | 966351 |
| ch03 | 54656234 | 54659205 | 54657720 | PGSC0003DMG400024613 | Protein ASC1 | 1001531.5 |
| ch03 | 54961544 | 54963584 | 54962564 | PGSC0003DMG400024530 | Protein COBRA | 696687 |
| ch03 | 56388027 | 56390843 | 56389435 | PGSC0003DMG400000600 | Protein HVA22 | 730184 |
| ch03 | 54141229 | 54146751 | 54143990 | PGSC0003DMG400024596 | Protein kinase atmrk1 | 1515261 |
| ch03 | 54006159 | 54007939 | 54007049 | PGSC0003DMG400024589 | Protein PRD1 | 1652202 |
| ch03 | 57209218 | 57211637 | 57210428 | PGSC0003DMG400014137 | Protein TOPLESS | 1551176.5 |
| ch03 | 55279369 | 55282340 | 55280855 | PGSC0003DMG400024543 | Protein tyrosine phosphatase | 378396.5 |
| ch03 | 57527085 | 57527421 | 57527253 | PGSC0003DMG402014160 | Protein WAX2 | 1868002 |
| ch03 | 55734633 | 55741670 | 55738152 | PGSC0003DMG400019518 | Pseudo response regulator | 78900.5 |
| ch03 | 53694121 | 53695966 | 53695044 | PGSC0003DMG400024467 | Putative anti-virus transcriptional factor | 1964207.5 |
| ch03 | 53743129 | 53750825 | 53746977 | PGSC0003DMG401024581 | Pyridoxamine 5-phosphate oxidase | 1912274 |
| ch03 | 54042117 | 54044321 | 54043219 | PGSC0003DMG401024590 | Rac protein | 1616032 |
| ch03 | 56437399 | 56442899 | 56440149 | PGSC0003DMG402000630 | Racemase and epimerase, acting on amino acids and derivatives | 780898 |
| ch03 | 57553531 | 57562572 | 57558052 | PGSC0003DMG400014221 | Ribulose bisphosphate carboxylase/oxygenase activase, chloroplast | 1898800.5 |
| ch03 | 55775640 | 55782005 | 55778823 | PGSC0003DMG400019521 | Ribulose-phosphate 3-epimerase, chloroplastic | 119571.5 |
| ch03 | 54057013 | 54057951 | 54057482 | PGSC0003DMG400040994 | Ring finger protein | 1601769 |
| ch03 | 55443299 | 55443883 | 55443591 | PGSC0003DMG400024652 | RING-H2 finger protein ATL1M | 215660 |
| ch03 | 54068745 | 54069866 | 54069306 | PGSC0003DMG400024487 | RNA binding protein | 1589945.5 |
| ch03 | 56522242 | 56522732 | 56522487 | PGSC0003DMG400000646 | RNase H family protein | 863236 |
| ch03 | 55765639 | 55771810 | 55768725 | PGSC0003DMG400019504 | Sand family protein | 109473.5 |
| ch03 | 54572440 | 54573754 | 54573097 | PGSC0003DMG400024608 | Senescence-associated protein | 1086154 |
| ch03 | 54561847 | 54562704 | 54562276 | PGSC0003DMG400045477 | Senescence-associated protein 5 | 1096975.5 |
| ch03 | 55641081 | 55645781 | 55643431 | PGSC0003DMG400024561 | Ser/Thr protein kinase | 15820 |
| ch03 | 54653788 | 54658842 | 54656315 | PGSC0003DMG400024513 | Serine/threonine protein kinase | 1002936 |
| ch03 | 57054280 | 57061338 | 57057809 | PGSC0003DMG403014197 | Serine/threonine-protein kinase PBS1 | 1398558 |
| ch03 | 54051726 | 54054216 | 54052971 | PGSC0003DMG400024485 | Serine-threonine protein kinase, plant-type | 1606280 |
| ch03 | 56036627 | 56043628 | 56040128 | PGSC0003DMG400019515 | SET protein, phospatase 2A inhibitor | 380876.5 |
| ch03 | 54139914 | 54140882 | 54140398 | PGSC0003DMG400024493 | Seven-transmembrane-domain protein 1 | 1518853 |
| ch03 | 56892955 | 56894017 | 56893486 | PGSC0003DMG400014119 | SFT2 domain containing 2 | 1234235 |
| ch03 | 56666693 | 56672595 | 56669644 | PGSC0003DMG400000625 | Signal recognition particle 54 kDa protein 1 | 1010393 |

| | | | | | | |
|---|---|---|---|---|---|---|
| ch03 | 55474349 | 55475978 | 55475164 | PGSC0003DMG400024655 | SNF2 domain-containing protein / helicase domain-containing protein / HNH endonuclease domain-containing protein | 184087.5 |
| ch03 | 55057180 | 55060002 | 55058591 | PGSC0003DMG400024631 | SP1L | 600660 |
| ch03 | 54110767 | 54113181 | 54111974 | PGSC0003DMG400024491 | Spotted leaf protein | 1547277 |
| ch03 | 56574769 | 56575915 | 56575342 | PGSC0003DMG400000620 | Stellacyanin CASLP1 | 916091 |
| ch03 | 56436555 | 56437339 | 56436947 | PGSC0003DMG402000629 | Stigma-specific protein Stig1 | 777696 |
| ch03 | 56920573 | 56925568 | 56923071 | PGSC0003DMG400014120 | Stromal cell-derived factor 2 | 1263819.5 |
| ch03 | 54016250 | 54023228 | 54019739 | PGSC0003DMG400024483 | Structural constituent of ribosome | 1639512 |
| ch03 | 54088348 | 54093737 | 54091043 | PGSC0003DMG400024489 | Sugar transporter | 1568208.5 |
| ch03 | 55445620 | 55449745 | 55447683 | PGSC0003DMG400024552 | Synaptosomal associated protein | 211568.5 |
| ch03 | 54482662 | 54486225 | 54484444 | PGSC0003DMG401024603 | TATA-binding protein-associated phosphoprotein | 1174807.5 |
| ch03 | 55841662 | 55843149 | 55842406 | PGSC0003DMG400019506 | Thioredoxin II | 183154.5 |
| ch03 | 54979843 | 54986094 | 54982969 | PGSC0003DMG400024531 | Thylakoid lumenal 25.6 kDa protein, chloroplast | 676282.5 |
| ch03 | 55959016 | 55959501 | 55959259 | PGSC0003DMG400019534 | Transcription factor | 300007.5 |
| ch03 | 54796953 | 54803667 | 54800310 | PGSC0003DMG400024523 | Transcription factor BIM1 | 858941 |
| ch03 | 57191604 | 57192393 | 57191999 | PGSC0003DMG400014204 | Transcription factor TSRF1 | 1532747.5 |
| ch03 | 56886710 | 56887250 | 56886980 | PGSC0003DMG400019832 | Transport protein | 1227729 |
| ch03 | 54941445 | 54943770 | 54942608 | PGSC0003DMG400024528 | Transposase Tam3 | 716643.5 |
| ch03 | 57475758 | 57479238 | 57477498 | PGSC0003DMG400014215 | Tubby protein | 1818247 |
| ch03 | 53710389 | 53712417 | 53711403 | PGSC0003DMG400024578 | Type-A response regulator | 1947848 |
| ch03 | 57610106 | 57618777 | 57614442 | PGSC0003DMG400014166 | U2 small nuclear ribonucleoprotein A | 1955190.5 |
| ch03 | 55397524 | 55398549 | 55398037 | PGSC0003DMG400024651 | Ubiquitin-protein ligase | 261214.5 |
| ch03 | 55359886 | 55361328 | 55360607 | PGSC0003DMG400036966 | UDP-glucose 6-dehydrogenase | 298644 |
| ch03 | 54778153 | 54779955 | 54779054 | PGSC0003DMG400024618 | UDP-glucosyltransferase | 880197 |
| ch03 | 54289906 | 54290359 | 54290133 | PGSC0003DMG400024496 | Ulp1 protease family, C-terminal catalytic domain containing protein | 1369118.5 |
| ch03 | 54312619 | 54315441 | 54314030 | PGSC0003DMG400024498 | UPF0497 membrane protein | 1345221 |
| ch03 | 54627728 | 54639244 | 54633486 | PGSC0003DMG400024612 | Uridine cytidine kinase I | 1025765 |
| ch03 | 57503815 | 57508531 | 57506173 | PGSC0003DMG401014218 | Uv excision repair protein rad23 | 1846922 |
| ch03 | 57066724 | 57068528 | 57067626 | PGSC0003DMG400014129 | Vegetative cell wall protein gp1 | 1408375 |
| ch03 | 55730618 | 55734374 | 55732496 | PGSC0003DMG400019502 | Vesicle transport v-SNARE 11 | 73245 |
| ch03 | 57316831 | 57317674 | 57317253 | PGSC0003DMG400014145 | VQ motif-containing protein | 1658001.5 |
| ch03 | 54724838 | 54731754 | 54728296 | PGSC0003DMG400024615 | WD-repeat protein | 930955 |
| ch03 | 56931977 | 56937779 | 56934878 | PGSC0003DMG402014189 | XH/XS domain-containing protein | 1275627 |
| ch03 | 55826686 | 55830767 | 55828727 | PGSC0003DMG400019524 | YGL010w | 169475.5 |
| ch03 | 56426550 | 56428641 | 56427596 | PGSC0003DMG400000604 | Zinc finger (CCCH-type) family protein | 768344.5 |

| | | | | | | |
|---|---|---|---|---|---|---|
| ch03 | 54304129 | 54310985 | 54307557 | PGSC0003DMG400024497 | Zinc finger family protein | 1351694 |
| ch03 | 55869333 | 55870654 | 55869994 | PGSC0003DMG400019526 | Zinc finger protein | 210742.5 |
| ch03 | 55187626 | 55190646 | 55189136 | PGSC0003DMG400024640 | Zinc ion binding protein | 470115 |
| ch03 | 55433276 | 55435278 | 55434277 | PGSC0003DMG400024550 | ZPT4-3 | 224974 |
| ch03 | 56396018 | 56403865 | 56399942 | PGSC0003DMG400000601 | ZR1 protein | 740690.5 |

| chr | start | end | mid | ID | Name | hit_dist |
|---|---|---|---|---|---|---|
| ch05 | 9404647 | 9407789 | 9406218 | PGSC0003DMG400013523 | Endo-1,4-beta-glucanase | 1216 |
| ch05 | 9401791 | 9403597 | 9402694 | PGSC0003DMG400013495 | Pto-like serine/threonine kinase | 4740 |
| ch05 | 9453059 | 9457342 | 9455201 | PGSC0003DMG400013524 | Protein kinase family protein | 47766.5 |
| ch05 | 9465568 | 9469319 | 9467444 | PGSC0003DMG400013525 | Kinase | 60009.5 |
| ch05 | 9324497 | 9325042 | 9324770 | PGSC0003DMG400035463 | Type I MADS box transcription factor | 82664.5 |
| ch05 | 9287263 | 9291478 | 9289371 | PGSC0003DMG401013522 | Prf | 118063.5 |
| ch05 | 9523854 | 9529432 | 9526643 | PGSC0003DMG400013497 | Polygalacturonase | 119209 |
| ch05 | 9273070 | 9278152 | 9275611 | PGSC0003DMG400013520 | F-Box protein | 131823 |
| ch05 | 9549109 | 9550821 | 9549965 | PGSC0003DMG400013499 | Pto disease resistance protein | 142531 |
| ch05 | 9557984 | 9559078 | 9558531 | PGSC0003DMG400037460 | Pto | 151097 |
| ch05 | 9248324 | 9258140 | 9253232 | PGSC0003DMG400013493 | POTH1 | 154202 |
| ch05 | 9560447 | 9567505 | 9563976 | PGSC0003DMG400026867 | LescPth4 | 156542 |
| ch05 | 9199239 | 9203522 | 9201381 | PGSC0003DMG400013492 | Lustrin A | 206053.5 |
| ch05 | 9646117 | 9647499 | 9646808 | PGSC0003DMG400016991 | LescPth2 | 239374 |
| ch05 | 9164717 | 9167941 | 9166329 | PGSC0003DMG400013519 | Ubiquitin-protein ligase | 241105 |
| ch05 | 9131587 | 9137722 | 9134655 | PGSC0003DMG400013489 | Leucine-rich repeat receptor-like kinase | 272779.5 |
| ch05 | 9119072 | 9119692 | 9119382 | PGSC0003DMG400038329 | Plant disease resistant protein | 288052 |
| ch05 | 9111929 | 9113418 | 9112674 | PGSC0003DMG400013488 | F-box/kelch protein | 294760.5 |
| ch05 | 9714775 | 9717165 | 9715970 | PGSC0003DMG400016996 | Nitrate transporter | 308536 |
| ch05 | 9065839 | 9067059 | 9066449 | PGSC0003DMG400013533 | Flavonol 4'-sulfotransferase | 340985 |
| ch05 | 9020646 | 9026828 | 9023737 | PGSC0003DMG400013486 | Disease resistance protein | 383697 |
| ch05 | 9823451 | 9838970 | 9831211 | PGSC0003DMG400007677 | Starch-granule-bound R1 protein | 423776.5 |
| ch05 | 8981205 | 8982062 | 8981634 | PGSC0003DMG403013517 | Polyprotein | 425800.5 |
| ch05 | 8974024 | 8980534 | 8977279 | PGSC0003DMG400013485 | ATP-citrate synthase | 430155 |
| ch05 | 8955439 | 8955840 | 8955640 | PGSC0003DMG400041786 | chromo' domain containing protein | 451794.5 |
| ch05 | 9858133 | 9863611 | 9860872 | PGSC0003DMG400007678 | Carboxyl-terminal proteinase | 453438 |
| ch05 | 9879031 | 9883513 | 9881272 | PGSC0003DMG400007679 | Lipase | 473838 |
| ch05 | 9927383 | 9929323 | 9928353 | PGSC0003DMG400007680 | Alliin lyase | 520919 |
| ch05 | 8873846 | 8878416 | 8876131 | PGSC0003DMG400013515 | Naphthoate synthase | 531303 |
| ch05 | 8869309 | 8872830 | 8871070 | PGSC0003DMG400013514 | ADP-ribosylation factor | 536364.5 |
| ch05 | 8864326 | 8868536 | 8866431 | PGSC0003DMG400013484 | Pyroglutamyl-peptidase I | 541003 |
| ch05 | 8844712 | 8850667 | 8847690 | PGSC0003DMG400013483 | ATP binding protein | 559744.5 |
| ch05 | 9990538 | 9991511 | 9991025 | PGSC0003DMG400007682 | SRF-type transcription factor family protein | 583590.5 |
| ch05 | 8807178 | 8817341 | 8812260 | PGSC0003DMG400013511 | Pantothenate kinase family protein | 595174.5 |

| | | | | | | |
|---|---|---|---|---|---|---|
| ch05 | 10017565 | 10018209 | 10017887 | PGSC0003DMG40003729 | Serine-threonine protein kinase, plant-type | 610453 |
| ch05 | 10018833 | 10020779 | 10019806 | PGSC0003DMG40001862 | Class III peroxidase 105 | 612372 |
| ch05 | 10026168 | 10026958 | 10026563 | PGSC0003DMG40001862 | Mads box protein | 619129 |
| ch05 | 10028440 | 10036840 | 10032640 | PGSC0003DMG40001862 | AlaT1 | 625206 |
| ch05 | 8776499 | 8779677 | 8778088 | PGSC0003DMG40001352 | Non-LTR retroelement reverse transcriptase | 629346 |
| ch05 | 8769870 | 8773433 | 8771652 | PGSC0003DMG40001348 | Seryl-tRNA synthetase | 635782.5 |
| ch05 | 10050524 | 10056913 | 10053719 | PGSC0003DMG40001860 | Tetratricopeptide-like helical | 646284.5 |
| ch05 | 10063175 | 10068988 | 10066082 | PGSC0003DMG40001862 | F-box family protein | 658647.5 |
| ch05 | 10072559 | 10072900 | 10072730 | PGSC0003DMG40004248 | MYBR domain class transcription factor | 665295.5 |
| ch05 | 10076697 | 10077296 | 10076997 | PGSC0003DMG40001862 | Myb family transcription factor | 669562.5 |
| ch05 | 10091268 | 10095994 | 10093631 | PGSC0003DMG40001861 | Tospovirus resistance protein B | 686197 |
| ch05 | 8714120 | 8715128 | 8714624 | PGSC0003DMG40001350 | I-box binding factor | 692810 |
| ch05 | 8701720 | 8702313 | 8702017 | PGSC0003DMG40001347 | Cytochrome P450 | 705417.5 |
| ch05 | 8663454 | 8668307 | 8665881 | PGSC0003DMG40001347 | Sentrin/sumo-specific protease, senp8 | 741553.5 |
| ch05 | 10174685 | 10181177 | 10177931 | PGSC0003DMG40001861 | Multidrug resistance pump | 770497 |
| ch05 | 8632422 | 8637714 | 8635068 | PGSC0003DMG40001347 | Helix-loop-helix DNA-binding | 772366 |
| ch05 | 8619648 | 8627296 | 8623472 | PGSC0003DMG40001350 | Late blight resistance protein homolog R1B-16 | 783962 |
| ch05 | 8607037 | 8608018 | 8607528 | PGSC0003DMG40001352 | Leafy cotyledon1 | 799906.5 |
| ch05 | 8606007 | 8606832 | 8606420 | PGSC0003DMG40001347 | Leafy cotyledon 1 | 801014.5 |
| ch05 | 8556583 | 8559010 | 8557797 | PGSC0003DMG40001347 | Glycine-rich protein 2 | 849637.5 |
| ch05 | 10268787 | 10274376 | 10271582 | PGSC0003DMG40001860 | Binding protein | 864147.5 |
| ch05 | 10287933 | 10288346 | 10288140 | PGSC0003DMG40003495 | Hydrolase, acting on ester bonds | 880705.5 |
| ch05 | 10314934 | 10318381 | 10316658 | PGSC0003DMG40001860 | Chloroplast phosphate transporter | 909223.5 |
| ch05 | 10391299 | 10392706 | 10392003 | PGSC0003DMG40001861 | AP2/ERF domain-containing transcription factor | 984568.5 |
| ch05 | 8404032 | 8405976 | 8405004 | PGSC0003DMG40003098 | Protein SUPPRESSOR OF GENE SILENCING 3 | 1002430 |
| ch05 | 8392953 | 8396882 | 8394918 | PGSC0003DMG40003100 | Nucleoredoxin | 1012517 |
| ch05 | 10418735 | 10422776 | 10420756 | PGSC0003DMG40101860 | Signal transducer | 1013322 |
| ch05 | 8388762 | 8389579 | 8389171 | PGSC0003DMG40103099 | Protein disulfide isomerase (PDI) 3 | 1018264 |
| ch05 | 8383814 | 8387263 | 8385539 | PGSC0003DMG40003099 | Alcohol dehydrogenase | 1021896 |
| ch05 | 8377372 | 8382917 | 8380145 | PGSC0003DMG40003098 | Carbonic anhydrase | 1027290 |
| ch05 | 8357300 | 8358240 | 8357770 | PGSC0003DMG40003099 | Protein phosphatase inhibitor 2 containing protein | 1049664 |
| ch05 | 10459329 | 10461897 | 10460613 | PGSC0003DMG40001861 | Glycosyl transferase family 17 protein | 1053179 |
| ch05 | 10463092 | 10468294 | 10465693 | PGSC0003DMG40001860 | Cucumisin | 1058259 |
| ch05 | 8344302 | 8346958 | 8345630 | PGSC0003DMG40003098 | Polygalacturonase non-catalytic subunit AroGP3 | 1061804 |
| ch05 | 10479601 | 10480300 | 10479951 | PGSC0003DMG40001861 | Elicitor resposible protein | 1072517 |

| | | | | | | |
|---|---|---|---|---|---|---|
| ch05 | 10524338 | 10532794 | 10528566 | PGSC0003DMG400018598 | CSK (CHLOROPLAST SENSOR KINASE) | 1121132 |
| ch05 | 10537459 | 10540083 | 10538771 | PGSC0003DMG402018597 | Alpha-L-fucosidase 2 | 1131337 |
| ch05 | 10556176 | 10563449 | 10559813 | PGSC0003DMG400018596 | EIF4B | 1152379 |
| ch05 | 8252711 | 8256080 | 8254396 | PGSC0003DMG400030978 | Polygalacturonase non-catalytic subunit AroGP2 | 1153039 |
| ch05 | 8222925 | 8225436 | 8224181 | PGSC0003DMG400030995 | Polygalacturonase-1 non-catalytic subunit beta | 1183254 |
| ch05 | 10591041 | 10595083 | 10593062 | PGSC0003DMG400011729 | Fe-S metabolism associated domain-containing protein | 1185628 |
| ch05 | 10597983 | 10604575 | 10601279 | PGSC0003DMG400011728 | Alpha-galactosidase/alpha-n-acetylgalactosaminidase | 1193845 |
| ch05 | 8193004 | 8200744 | 8196874 | PGSC0003DMG400030992 | Radical SAM domain-containing protein | 1210560 |
| ch05 | 10614999 | 10623108 | 10619054 | PGSC0003DMG400011727 | Auxin-independent growth promoter | 1211620 |
| ch05 | 8178837 | 8186133 | 8182485 | PGSC0003DMG400030991 | Metal-dependent phosphohydrolase HD domain-containing protein | 1224949 |
| ch05 | 8164118 | 8172460 | 8168289 | PGSC0003DMG400030977 | RNA-binding region RNP-1 and Splicing factor PWI family member protein | 1239145 |
| ch05 | 10659337 | 10663550 | 10661444 | PGSC0003DMG400011726 | MAP kinase phosphatase 1 | 1254010 |
| ch05 | 8139152 | 8140385 | 8139769 | PGSC0003DMG400030989 | Sentrin/sumo-specific protease | 1267666 |
| ch05 | 10676679 | 10681051 | 10678865 | PGSC0003DMG400011725 | PK12 protein kinase | 1271431 |
| ch05 | 8131824 | 8135732 | 8133778 | PGSC0003DMG400030976 | UDP-N-acetylglucosamine transferase subunit alg13 | 1273656 |
| ch05 | 10714245 | 10719910 | 10717078 | PGSC0003DMG400011723 | Paramyosin | 1309644 |
| ch05 | 8094887 | 8096351 | 8095619 | PGSC0003DMG400004029 | Spotted leaf protein | 1311815 |
| ch05 | 10777310 | 10782285 | 10779798 | PGSC0003DMG400016968 | EMB1745 | 1372364 |
| ch05 | 8018695 | 8022801 | 8020748 | PGSC0003DMG400017005 | Aldehyde dehydrogenase (NAD+) | 1386686 |
| ch05 | 10800594 | 10802791 | 10801693 | PGSC0003DMG400016975 | RNase H family protein | 1394259 |
| ch05 | 10803005 | 10809245 | 10806125 | PGSC0003DMG400016965 | Hydrolase, hydrolyzing O-glycosyl compounds | 1398691 |
| ch05 | 10806557 | 10816168 | 10811363 | PGSC0003DMG400016969 | Proteasome subunit beta type | 1403929 |
| ch05 | 10930199 | 10931820 | 10931010 | PGSC0003DMG400016973 | F-box and wd40 domain protein | 1523576 |
| ch05 | 10943260 | 10946646 | 10944953 | PGSC0003DMG400017965 | Calmodulin | 1537519 |
| ch05 | 10953441 | 10958557 | 10955999 | PGSC0003DMG400017970 | Armadillo/beta-catenin repeat family protein | 1548565 |
| ch05 | 10959337 | 10962640 | 10960989 | PGSC0003DMG400017969 | Hcr2-0A | 1553555 |
| ch05 | 10963833 | 10964924 | 10964379 | PGSC0003DMG400017968 | Serine/threonine-protein kinase bri1 | 1556945 |
| ch05 | 10969744 | 10971986 | 10970865 | PGSC0003DMG400017967 | Hcr2-0B | 1563431 |
| ch05 | 10974827 | 10977109 | 10975968 | PGSC0003DMG400017966 | Hcr2-p3 | 1568534 |
| ch05 | 7834994 | 7836879 | 7835937 | PGSC0003DMG400004109 | Xyloglucan endotransglycosylase hydrolase | 1571498 |
| ch05 | 7831932 | 7832724 | 7832328 | PGSC0003DMG400004124 | Ribosomal protein S9 | 1575106 |
| ch05 | 7774270 | 7779490 | 7776880 | PGSC0003DMG402004125 | Mitochondrial benzaldehyde dehydrogenase | 1630554 |
| ch05 | 11042954 | 11047763 | 11045359 | PGSC0003DMG400010774 | Multidrug resistance protein 1, 2 | 1637925 |
| ch05 | 11056609 | 11058305 | 11057457 | PGSC0003DMG400010780 | Vignain | 1650023 |
| ch05 | 7752878 | 7756398 | 7754638 | PGSC0003DMG400004111 | Spermidine synthase | 1652796 |

| | | | | | | |
|---|---|---|---|---|---|---|
| ch05 | 11074323 | 11076135 | 11075229 | PGSC0003DMG40001077З | Terminal ear1-like 2 protein | 1667795 |
| ch05 | 7716491 | 7722639 | 7719565 | PGSC0003DMG40000411З | Potassium transporter | 1687869 |
| ch05 | 7712490 | 7715076 | 7713783 | PGSC0003DMG40000412б | F-box | 1693651 |
| ch05 | 7684187 | 7691671 | 7687929 | PGSC0003DMG400004114 | Trehalose 6-phosphate synthase | 1719505 |
| ch05 | 7673124 | 7676670 | 7674897 | PGSC0003DMG400004115 | NHL repeat-containing protein | 1732537 |
| ch05 | 11149675 | 11157917 | 11153796 | PGSC0003DMG40001077٩ | Oxysterol-binding family protein | 1746362 |
| ch05 | 7645185 | 7664375 | 7654780 | PGSC0003DMG40000411б | AP-1 complex subunit gamma-2 | 1752654 |
| ch05 | 7640385 | 7641906 | 7641146 | PGSC0003DMG40000412ȣ | Ring finger protein | 1766289 |
| ch05 | 11199199 | 11204974 | 11202087 | PGSC0003DMG40001077ȣ | RNA binding protein | 1794653 |
| ch05 | 11213912 | 11214833 | 11214373 | PGSC0003DMG40001077б | Chaperonin containing t-complex protein 1, epsilon subunit, tcpe | 1806939 |
| ch05 | 11221031 | 11223925 | 11222478 | PGSC0003DMG400010771 | Pectate lyase | 1815044 |
| ch05 | 7584954 | 7588163 | 7586559 | PGSC0003DMG40000413C | Glyceraldehyde-3-phosphate dehydrogenase C subunit | 1820876 |
| ch05 | 7577951 | 7581368 | 7579660 | PGSC0003DMG40000411ȣ | Auxin-induced protein 5NG4 | 1827775 |
| ch05 | 11252622 | 11256056 | 11254339 | PGSC0003DMG40001073٩ | RCC1 and BTB domain-containing protein | 1846905 |
| ch05 | 7546929 | 7548191 | 7547560 | PGSC0003DMG400004131 | Major latex | 1859874 |
| ch05 | 7538829 | 7541985 | 7540407 | PGSC0003DMG400004122 | 30S ribosomal protein S13, chloroplastic | 1867027 |
| ch05 | 7531834 | 7537250 | 7534542 | PGSC0003DMG40000412З | Serine/threonine-protein kinase PBS1 | 1872892 |
| ch05 | 7530524 | 7537250 | 7533887 | PGSC0003DMG400004132 | N-acetyltransferase | 1873547 |
| ch05 | 11279513 | 11286927 | 11283220 | PGSC0003DMG40001074C | ALBINO3 1, chloroplastic | 1875786 |
| ch05 | 7528121 | 7529797 | 7528959 | PGSC0003DMG40000413З | LATD/NIP | 1878475 |
| ch05 | 11304205 | 11310209 | 11307207 | PGSC0003DMG400010741 | Uro-adherence factor A | 1899773 |
| ch05 | 11311414 | 11317293 | 11314354 | PGSC0003DMG400010742 | 3-ketoacyl-CoA reductase 2 | 1906920 |
| ch05 | 11375191 | 11379026 | 11377109 | PGSC0003DMG40001073б | Prenyl-dependent CAAX protease | 1969675 |

| chr | start | end | mid | ID | Name | hit_dist |
|---|---|---|---|---|---|---|
| ch06 | 57521113 | 57526091 | 57523602 | PGSC0003DMG400030459 | Spliceosomal protein | 4365 |
| ch06 | 57547785 | 57551261 | 57549523 | PGSC0003DMG400030381 | Nodulin | 21556 |
| ch06 | 57556039 | 57558160 | 57557100 | PGSC0003DMG400030382 | Class III peroxidase | 29132.5 |
| ch06 | 57561148 | 57563084 | 57562116 | PGSC0003DMG400030461 | LOB domain-containing protein | 34149 |
| ch06 | 57566917 | 57568428 | 57567673 | PGSC0003DMG400030462 | Avr9/Cf-9 rapidly elicited protein 216 | 39705.5 |
| ch06 | 57482051 | 57483896 | 57482974 | PGSC0003DMG400030455 | UDP-glucosyltransferase | 44993.5 |
| ch06 | 57580219 | 57584530 | 57582375 | PGSC0003DMG400030463 | NN mitogen-activated protein kinase | 54407.5 |
| ch06 | 57470079 | 57473694 | 57471887 | PGSC0003DMG400030453 | Mitochondrial carrier protein | 56080.5 |
| ch06 | 57464531 | 57469331 | 57466931 | PGSC0003DMG400030377 | Pectinesterase | 61036 |
| ch06 | 57589308 | 57597820 | 57593564 | PGSC0003DMG400030464 | Nucleic acid binding protein | 65597 |
| ch06 | 57460344 | 57462342 | 57461343 | PGSC0003DMG400030452 | Periplasmic beta-glucosidase | 66624 |
| ch06 | 57455374 | 57459031 | 57457203 | PGSC0003DMG400030451 | Mitochondrial import inner membrane translocase subunit Tim17 | 70764.5 |
| ch06 | 57602757 | 57605815 | 57604286 | PGSC0003DMG400030465 | Alpha-1,3-mannosyltransferase | 76319 |
| ch06 | 57446996 | 57453781 | 57450389 | PGSC0003DMG400030376 | Laccase | 77578.5 |
| ch06 | 57606543 | 57612100 | 57609322 | PGSC0003DMG400030466 | UDP-glucuronic acid/UDP-N-acetylgalactosamine transporter | 81354.5 |
| ch06 | 57441666 | 57444726 | 57443196 | PGSC0003DMG400030375 | ATCHX14 | 84771 |
| ch06 | 57614588 | 57617481 | 57616035 | PGSC0003DMG400030385 | Telomeric repeat binding protein | 88067.5 |
| ch06 | 57434391 | 57439890 | 57437141 | PGSC0003DMG400030374 | RNA binding protein | 90826.5 |
| ch06 | 57619926 | 57623337 | 57621632 | PGSC0003DMG400030467 | Transcription factor | 93664.5 |
| ch06 | 57429801 | 57430733 | 57430267 | PGSC0003DMG400034612 | Gamma-interferon-inducible lysosomal thiol reductase | 97700 |
| ch06 | 57636790 | 57638946 | 57637868 | PGSC0003DMG400030468 | GRAS4 | 109901 |
| ch06 | 57643272 | 57647008 | 57645140 | PGSC0003DMG401030469 | Cinnamic acid 4-hydroxylase | 117173 |
| ch06 | 57664520 | 57666462 | 57665491 | PGSC0003DMG400030470 | RAB6-interacting protein | 137524 |
| ch06 | 57668069 | 57674442 | 57671256 | PGSC0003DMG400030471 | Eukaryotic translation initiation factor 2 beta subunit | 143288.5 |
| ch06 | 57379912 | 57384899 | 57382406 | PGSC0003DMG400030450 | Protein kinase | 145561.5 |
| ch06 | 57680111 | 57681571 | 57680841 | PGSC0003DMG400030472 | TSI1 | 152874 |
| ch06 | 57371491 | 57372841 | 57372166 | PGSC0003DMG400030449 | Glutaredoxin family protein | 155801 |
| ch06 | 57684685 | 57689801 | 57687243 | PGSC0003DMG400030473 | Ubiquitin-conjugating enzyme E2-17 kDa | 159276 |
| ch06 | 57691785 | 57697068 | 57694427 | PGSC0003DMG400030474 | Nodulation receptor kinase | 166459.5 |
| ch06 | 57359099 | 57361686 | 57360393 | PGSC0003DMG400030371 | Transcription factor PosF21 | 167574.5 |
| ch06 | 57703304 | 57708640 | 57705972 | PGSC0003DMG400030476 | 26S protease regulatory subunit 6B homolog | 178005 |
| ch06 | 57336016 | 57349568 | 57342792 | PGSC0003DMG400030370 | Fructose-6-phosphate 2-kinase/fructose-2,6-bisphosphatase | 185175 |
| ch06 | 57711928 | 57714382 | 57713155 | PGSC0003DMG400030386 | 60S ribosomal protein L10 | 185188 |
| ch06 | 57714831 | 57717305 | 57716068 | PGSC0003DMG400030477 | Cytochrome b-c1 complex subunit 8 | 188101 |

| | | | | | | |
|---|---|---|---|---|---|---|
| ch06 | 57718112 | 57719053 | 57718583 | PGSC0003DMG40003038 | Protein phosphatase 2C 51 | 190615.5 |
| ch06 | 57330850 | 57333771 | 57332311 | PGSC0003DMG40003036 | Pyruvate decarboxylase isozyme 2 | 195656.5 |
| ch06 | 57326191 | 57329883 | 57328037 | PGSC0003DMG40003036 | Ran binding protein | 199930 |
| ch06 | 57325143 | 57325595 | 57325369 | PGSC0003DMG40003036 | U520 | 202598 |
| ch06 | 57730676 | 57731368 | 57731022 | PGSC0003DMG40003047 | GTP-binding nuclear protein Ran-1 | 203055 |
| ch06 | 57314827 | 57323565 | 57319196 | PGSC0003DMG40003036 | EMB1507 | 208771 |
| ch06 | 57309861 | 57313784 | 57311823 | PGSC0003DMG40003036 | EBP1 | 216144.5 |
| ch06 | 57298365 | 57300540 | 57299453 | PGSC0003DMG40003036 | Avr9/Cf-9 rapidly elicited protein 284 | 228514.5 |
| ch06 | 57254939 | 57261466 | 57258203 | PGSC0003DMG40003036 | 2OG-Fe(II) oxidoreductase | 269764.5 |
| ch06 | 57807447 | 57810622 | 57809035 | PGSC0003DMG40002767 | 50S ribosomal protein L17, chloroplastic | 281067.5 |
| ch06 | 57242335 | 57245635 | 57243985 | PGSC0003DMG40003036 | Cys-3-His zinc finger protein | 283982 |
| ch06 | 57232602 | 57238419 | 57235511 | PGSC0003DMG40003036 | PI-phospholipase C PLC6 | 292456.5 |
| ch06 | 57230608 | 57231909 | 57231259 | PGSC0003DMG40003035 | Pyridoxine biosynthesis protein isoform A | 296708.5 |
| ch06 | 57826539 | 57827916 | 57827228 | PGSC0003DMG40002767 | Pentatricopeptide repeat-containing protein | 299260.5 |
| ch06 | 57221218 | 57224548 | 57222883 | PGSC0003DMG40003035 | Lipopolysaccharide-modifying protein | 305084 |
| ch06 | 57216287 | 57220094 | 57218191 | PGSC0003DMG40003044 | Peptidyl-prolyl cis-trans isomerase | 309776.5 |
| ch06 | 57212675 | 57216008 | 57214342 | PGSC0003DMG40003035 | ELF4 | 313625.5 |
| ch06 | 57207307 | 57211071 | 57209189 | PGSC0003DMG40003035 | UPA14 | 318778 |
| ch06 | 57845590 | 57850342 | 57847966 | PGSC0003DMG40002766 | Exonuclease family protein | 319999 |
| ch06 | 57200282 | 57204128 | 57202205 | PGSC0003DMG40003035 | NudC domain-containing protein 2 | 325762 |
| ch06 | 57855801 | 57859958 | 57857880 | PGSC0003DMG40002766 | MLO 1 | 329912.5 |
| ch06 | 57864189 | 57871210 | 57867700 | PGSC0003DMG40202768 | Auxilin | 339732.5 |
| ch06 | 57179298 | 57179699 | 57179499 | PGSC0003DMG40004595 | Transposon protein | 348468.5 |
| ch06 | 57875529 | 57879156 | 57877343 | PGSC0003DMG40002768 | Dopamine beta-monooxygenase | 349375.5 |
| ch06 | 57884390 | 57888412 | 57886401 | PGSC0003DMG40002766 | Ribosomal protein L5 | 358434 |
| ch06 | 57160730 | 57167644 | 57164187 | PGSC0003DMG40003044 | Receptor protein kinase | 363780 |
| ch06 | 57892514 | 57900201 | 57896358 | PGSC0003DMG40002768 | RNase NGR2 | 368390.5 |
| ch06 | 57158086 | 57160320 | 57159203 | PGSC0003DMG40004657 | Pentatricopeptide repeat-containing protein, chloroplastic | 368764 |
| ch06 | 57153371 | 57154108 | 57153740 | PGSC0003DMG40003035 | Plekhh protein | 374227.5 |
| ch06 | 57148354 | 57152102 | 57150228 | PGSC0003DMG40003035 | Flavoprotein wrbA | 377739 |
| ch06 | 57143295 | 57145909 | 57144602 | PGSC0003DMG40003044 | 60S acidic ribosomal protein P0 | 383365 |
| ch06 | 57135818 | 57142781 | 57139300 | PGSC0003DMG40003035 | Cdc6 | 388667.5 |
| ch06 | 57915453 | 57919498 | 57917476 | PGSC0003DMG40002766 | FH protein NFH2 | 389508.5 |
| ch06 | 57935560 | 57937260 | 57936410 | PGSC0003DMG40002767 | Photosystem I subunit XI | 408443 |
| ch06 | 57111860 | 57115524 | 57113692 | PGSC0003DMG40003044 | Binding protein | 414275 |

| | | | | | | |
|---|---|---|---|---|---|---|
| ch06 | 57943526 | 57945216 | 57944371 | PGSC0003DMG400027673 | DNAJ heat shock N-terminal domain-containing protein | 416404 |
| ch06 | 57946272 | 57948790 | 57947531 | PGSC0003DMG400027684 | C-8,7 sterol isomerase | 419564 |
| ch06 | 57951353 | 57954837 | 57953095 | PGSC0003DMG401027685 | Uncharacterized CRM domain-containing protein, chloroplastic | 425128 |
| ch06 | 57101142 | 57103475 | 57102309 | PGSC0003DMG400044141 | Myb family transcription factor | 425658.5 |
| ch06 | 57955198 | 57955839 | 57955519 | PGSC0003DMG402027685 | Calcium-binding protein | 427551.5 |
| ch06 | 57959435 | 57960193 | 57959814 | PGSC0003DMG400027686 | Ferredoxin | 431847 |
| ch06 | 57094190 | 57097929 | 57096060 | PGSC0003DMG402030350 | GONST2 Golgi Nucleotide sugar transporter | 431907.5 |
| ch06 | 57960905 | 57968842 | 57964874 | PGSC0003DMG401027687 | Wound-inducible carboxypeptidase | 436906.5 |
| ch06 | 57087896 | 57089374 | 57088635 | PGSC0003DMG401030350 | Zinc finger family protein | 439332 |
| ch06 | 57074754 | 57076893 | 57075824 | PGSC0003DMG400030349 | CYP86A33 fatty acid omega-hydroxylase | 452143.5 |
| ch06 | 57071491 | 57072354 | 57071923 | PGSC0003DMG400030440 | Thylakoid soluble phosphoprotein | 456044.5 |
| ch06 | 57056946 | 57061434 | 57059190 | PGSC0003DMG400030439 | Hydrolase, hydrolyzing O-glycosyl compounds | 468777 |
| ch06 | 57052558 | 57055245 | 57053902 | PGSC0003DMG400030348 | MYB-like transcription factor DIVARICATA | 474065.5 |
| ch06 | 57021923 | 57025562 | 57023743 | PGSC0003DMG400030438 | Peptide transporter | 504224.5 |
| ch06 | 57009063 | 57011249 | 57010156 | PGSC0003DMG400030437 | DNA binding protein | 517811 |
| ch06 | 58059700 | 58063683 | 58061692 | PGSC0003DMG402020115 | Protein FAM96B | 533724.5 |
| ch06 | 58064308 | 58071118 | 58067713 | PGSC0003DMG401020115 | Fimbrin | 539746 |
| ch06 | 56980819 | 56984611 | 56982715 | PGSC0003DMG400030434 | Acyl-CoA thioesterase | 545252 |
| ch06 | 56974899 | 56978351 | 56976625 | PGSC0003DMG400030345 | FUS-interacting serine-arginine-rich protein 1 | 551342 |
| ch06 | 56972156 | 56973956 | 56973056 | PGSC0003DMG400030432 | Proliferating cell nuclear antigen | 554911 |
| ch06 | 56952637 | 56971724 | 56962181 | PGSC0003DMG400030344 | Enolase | 565786.5 |
| ch06 | 56942398 | 56945344 | 56943871 | PGSC0003DMG400030431 | Beta-tubulin 2 | 584096 |
| ch06 | 56939869 | 56941213 | 56940541 | PGSC0003DMG400030430 | Anionic peroxidase swpa7 | 587426 |
| ch06 | 56927255 | 56931286 | 56929271 | PGSC0003DMG400030428 | Zinc finger protein | 598696.5 |
| ch06 | 58132109 | 58139668 | 58135889 | PGSC0003DMG402020176 | Mitochondrial import receptor | 607921.5 |
| ch06 | 56916610 | 56916966 | 56916788 | PGSC0003DMG400037544 | S-protein homologue | 611179 |
| ch06 | 56907909 | 56908742 | 56908326 | PGSC0003DMG400030341 | 17.6 kDa class I heat shock protein | 619641.5 |
| ch06 | 56905147 | 56905872 | 56905510 | PGSC0003DMG400030340 | 17.6 kD class I small heat shock protein | 622457.5 |
| ch06 | 58154214 | 58155473 | 58154844 | PGSC0003DMG400020111 | EPIDERMAL PATTERNING FACTOR 9 | 626876.5 |
| ch06 | 56898542 | 56900374 | 56899458 | PGSC0003DMG400030338 | UDP-glucuronosyltransferase | 628509 |
| ch06 | 56896895 | 56897612 | 56897254 | PGSC0003DMG400030427 | Hsp20.1 protein | 630713.5 |
| ch06 | 58161970 | 58163163 | 58162567 | PGSC0003DMG400020110 | Auxin-induced in root cultures protein 12 | 634599.5 |
| ch06 | 56886703 | 56892891 | 56889797 | PGSC0003DMG400030337 | Phosphoserine phosphatase | 638170 |
| ch06 | 58165520 | 58169772 | 58167646 | PGSC0003DMG400020175 | Heat shock protein 70 (HSP70)-interacting protein | 639679 |
| ch06 | 56873853 | 56879282 | 56876568 | PGSC0003DMG400030336 | IMP dehydrogenase/GMP reductase | 651399.5 |

| | | | | | | |
|---|---|---|---|---|---|---|
| ch06 | 58191152 | 58193957 | 58192555 | PGSC0003DMG400020108 | 40S ribosomal protein S8 | 664587.5 |
| ch06 | 56859456 | 56860115 | 56859786 | PGSC0003DMG400034939 | Thylakoid lumenal 15 kDa protein, chloroplast | 668181.5 |
| ch06 | 58194667 | 58198707 | 58196687 | PGSC0003DMG400020107 | Peptidylprolyl isomerase | 668720 |
| ch06 | 56845461 | 56850790 | 56848126 | PGSC0003DMG400030334 | Transcription elongation factor B polypeptide | 679841.5 |
| ch06 | 58205585 | 58212461 | 58209023 | PGSC0003DMG400020173 | Mak | 681056 |
| ch06 | 56841442 | 56843552 | 56842497 | PGSC0003DMG400030425 | RabA2 | 685470 |
| ch06 | 58213279 | 58218141 | 58215710 | PGSC0003DMG400020172 | ATP binding protein | 687743 |
| ch06 | 56837940 | 56840493 | 56839217 | PGSC0003DMG400030333 | Metal tolerance protein | 688750.5 |
| ch06 | 58218330 | 58221998 | 58220164 | PGSC0003DMG400020105 | GTP cyclohydrolase I | 692197 |
| ch06 | 56829948 | 56835522 | 56832735 | PGSC0003DMG400030424 | Serine/threonine-protein phosphatase PP1 isozyme 2 | 695232 |
| ch06 | 58224782 | 58232366 | 58228574 | PGSC0003DMG401020104 | Cyclin-C1-2 | 700607 |
| ch06 | 56825065 | 56826579 | 56825822 | PGSC0003DMG400030423 | Extensin | 702145 |
| ch06 | 58231764 | 58232366 | 58232065 | PGSC0003DMG402020104 | Cyclin-C1-1 | 704098 |
| ch06 | 56817964 | 56824008 | 56820986 | PGSC0003DMG400030422 | C-4 methyl sterol oxidase | 706981 |
| ch06 | 58236670 | 58239331 | 58238001 | PGSC0003DMG400020170 | Cytochrome P450 | 710033.5 |
| ch06 | 56814529 | 56816918 | 56815724 | PGSC0003DMG400030332 | Protein phosphatase 2C | 712243.5 |
| ch06 | 58242666 | 58246276 | 58244471 | PGSC0003DMG400020169 | ER lumen retaining receptor family | 716504 |
| ch06 | 58250186 | 58251133 | 58250660 | PGSC0003DMG400020167 | RNA-binding protein | 722692.5 |
| ch06 | 58257594 | 58261161 | 58259378 | PGSC0003DMG400020103 | Glycosyltransferase QUASIMODO1 | 731410.5 |
| ch06 | 56791368 | 56798026 | 56794697 | PGSC0003DMG400030421 | TFIIA | 733270 |
| ch06 | 56787427 | 56788731 | 56788079 | PGSC0003DMG400030329 | Eukaryotic RNA polymerase II heptapeptide repeat | 739888 |
| ch06 | 58267208 | 58268606 | 58267907 | PGSC0003DMG400020101 | Wound-induced basic protein | 739940 |
| ch06 | 56781624 | 56786025 | 56783825 | PGSC0003DMG400030420 | Sorting and assembly machinery (Sam50) protein | 744142.5 |
| ch06 | 58270365 | 58276035 | 58273200 | PGSC0003DMG400020100 | Transcriptional activator | 745233 |
| ch06 | 56777855 | 56780854 | 56779355 | PGSC0003DMG400030328 | StPCL1 protein | 748612.5 |
| ch06 | 58278397 | 58280845 | 58279621 | PGSC0003DMG400020165 | DNA-directed RNA polymerases I, II, and III subunit RPABC5 | 751654 |
| ch06 | 56763310 | 56772409 | 56767860 | PGSC0003DMG400030327 | Pumilio | 760107.5 |
| ch06 | 58288217 | 58294124 | 58291171 | PGSC0003DMG400020163 | Acyl-ACP thioesterase | 763203.5 |
| ch06 | 58295329 | 58297535 | 58296432 | PGSC0003DMG400020099 | RPM1 interacting protein 4 transcript 2 | 768465 |
| ch06 | 58299279 | 58300337 | 58299808 | PGSC0003DMG400041434 | Xyloglucan endotransglucosylase/hydrolase protein 1 | 771841 |
| ch06 | 58309639 | 58313014 | 58311327 | PGSC0003DMG400020162 | Protein XRI1 | 783359.5 |
| ch06 | 56740708 | 56741202 | 56740955 | PGSC0003DMG400030326 | FtsJ cell division protein | 787012 |
| ch06 | 56734658 | 56737790 | 56736224 | PGSC0003DMG400030418 | Ripening protein | 791743 |
| ch06 | 56715994 | 56720030 | 56718012 | PGSC0003DMG400030324 | MYBR domain class transcription factor | 809955 |
| ch06 | 56705258 | 56710563 | 56707911 | PGSC0003DMG400030417 | Rab5-interacting protein | 820056.5 |

| | | | | | | |
|---|---|---|---|---|---|---|
| ch06 | 58349594 | 58352516 | 58351055 | PGSC0003DMG40002016( | Cytochrome b5 | 823088 |
| ch06 | 58352196 | 58354657 | 58353427 | PGSC0003DMG40002009; | Catechol O-methyltransferase | 825459.5 |
| ch06 | 58362614 | 58363525 | 58363070 | PGSC0003DMG40002009! | O-methyltransferase | 835102.5 |
| ch06 | 56687730 | 56689959 | 56688845 | PGSC0003DMG40003041! | Expansin18 | 839122.5 |
| ch06 | 56682457 | 56686521 | 56684489 | PGSC0003DMG40003032; | XH/XS domain-containing protein | 843478 |
| ch06 | 58370922 | 58378851 | 58374887 | PGSC0003DMG40002009; | STY-L protein | 846919.5 |
| ch06 | 56662677 | 56664444 | 56663561 | PGSC0003DMG40003041 | Glucan endo-1,3-beta-glucosidase | 864406.5 |
| ch06 | 56645024 | 56649683 | 56647354 | PGSC0003DMG40003041; | Translation-initiation factor 3 subunit | 880613.5 |
| ch06 | 56643280 | 56644282 | 56643781 | PGSC0003DMG40003032 | Metallothionein type 2 | 884186 |
| ch06 | 56633709 | 56637197 | 56635453 | PGSC0003DMG40003032( | GTP1/OBG family member | 892514 |
| ch06 | 56623576 | 56625165 | 56624371 | PGSC0003DMG40003041 | Fasciclin-like arabinogalactan protein 9 | 903596.5 |
| ch06 | 58441442 | 58445444 | 58443443 | PGSC0003DMG40202009 | Receptor serine-threonine protein kinase | 915476 |
| ch06 | 58451093 | 58456305 | 58453699 | PGSC0003DMG40002015! | Nuclear GTPase | 925732 |
| ch06 | 56600282 | 56603782 | 56602032 | PGSC0003DMG40003041( | Protein phosphatase 2C 4 | 925935 |
| ch06 | 58457801 | 58460751 | 58459276 | PGSC0003DMG40002015 | Vesicle-associated membrane protein | 931309 |
| ch06 | 58461318 | 58464631 | 58462975 | PGSC0003DMG40102009( | Nucleic acid binding | 935007.5 |
| ch06 | 56589763 | 56592303 | 56591033 | PGSC0003DMG40003031! | Actin | 936934 |
| ch06 | 58465264 | 58471072 | 58468168 | PGSC0003DMG40002008! | F-box/kelch-repeat protein | 940201 |
| ch06 | 58481971 | 58488674 | 58485323 | PGSC0003DMG40002015( | Pectase lyase | 957355.5 |
| ch06 | 56564175 | 56567014 | 56565595 | PGSC0003DMG40003031 | Ring zinc finger protein | 962372.5 |
| ch06 | 58489492 | 58492357 | 58490925 | PGSC0003DMG40002008 | VP1-ABI3 | 962957.5 |
| ch06 | 58498775 | 58501744 | 58500260 | PGSC0003DMG40002015! | ABI3 | 972292.5 |
| ch06 | 56550311 | 56555870 | 56553091 | PGSC0003DMG40003040; | Spotted leaf protein | 974876.5 |
| ch06 | 58504579 | 58506607 | 58505593 | PGSC0003DMG40002008; | Late embryo abundance protein | 977626 |
| ch06 | 58509430 | 58513326 | 58511378 | PGSC0003DMG40002008( | 26S proteasome subunit 4 | 983411 |
| ch06 | 58513948 | 58518525 | 58516237 | PGSC0003DMG40202008! | Protein YIF1A | 988269.5 |
| ch06 | 56537028 | 56538529 | 56537779 | PGSC0003DMG40003040( | Dof zinc finger protein | 990188.5 |
| ch06 | 58519113 | 58527585 | 58523349 | PGSC0003DMG40102008! | Leucine-rich repeat family protein | 995382 |
| ch06 | 56527986 | 56530975 | 56529481 | PGSC0003DMG40003040! | Heat shock cognate 70 kDa protein 1 | 998486.5 |
| ch06 | 56520583 | 56522059 | 56521321 | PGSC0003DMG40003031( | WOX2 | 1006646 |
| ch06 | 56507691 | 56508709 | 56508200 | PGSC0003DMG40003031! | Lactoylglutathione lyase family protein | 1019767 |
| ch06 | 56500563 | 56506714 | 56503639 | PGSC0003DMG40003040 | CRAL/TRIO domain containing protein | 1024328.5 |
| ch06 | 58564982 | 58566400 | 58565691 | PGSC0003DMG40002015 | Photosystem I reaction center subunit IV A isoform 2 | 1037724 |
| ch06 | 58565713 | 58573300 | 58569507 | PGSC0003DMG40002008( | Glutaredoxin S12 | 1041539.5 |
| ch06 | 56477428 | 56477965 | 56477697 | PGSC0003DMG40003040; | Histone H4 | 1050270.5 |

| | | | | | | |
|---|---|---|---|---|---|---|
| ch06 | 58577357 | 58579120 | 58578239 | PGSC0003DMG40002007§ | Flavin monooxygenase | 1050271.5 |
| ch06 | 56471436 | 56474544 | 56472990 | PGSC0003DMG40003031 | Bifunctional endo-1,4-beta-xylanase xylA | 1054977 |
| ch06 | 56457555 | 56466850 | 56462203 | PGSC0003DMG40004484 | GF13792 | 1065764.5 |
| ch06 | 56455838 | 56456761 | 56456300 | PGSC0003DMG40003512( | Secreted protein | 1071667.5 |
| ch06 | 56449905 | 56451080 | 56450493 | PGSC0003DMG40004539 | ORF_19 | 1077474.5 |
| ch06 | 56447742 | 56448785 | 56448264 | PGSC0003DMG40003944 | Cell surface protein | 1079703.5 |
| ch06 | 56437071 | 56440222 | 56438647 | PGSC0003DMG40004133( | GK22155 | 1089320.5 |
| ch06 | 58613300 | 58622800 | 58618050 | PGSC0003DMG40002015 | DENN domain containing protein | 1090083 |
| ch06 | 56427732 | 56429492 | 56428612 | PGSC0003DMG40003657 | Curlin associated repeat protein | 1099355 |
| ch06 | 56424740 | 56425949 | 56425345 | PGSC0003DMG40004501 | Glycine-rich protein | 1102622.5 |
| ch06 | 58630179 | 58638893 | 58634536 | PGSC0003DMG40002015 | Ras-GTPase-activating protein-binding protein | 1106569 |
| ch06 | 56415509 | 56419248 | 56417379 | PGSC0003DMG40004109 | Fad NAD binding oxidoreductases | 1110588.5 |
| ch06 | 58640829 | 58643258 | 58642044 | PGSC0003DMG40002007 | DUF26 domain-containing protein 2 | 1114076.5 |
| ch06 | 58651024 | 58655467 | 58653246 | PGSC0003DMG40002015 | SET domain protein | 1125278.5 |
| ch06 | 56399429 | 56400977 | 56400203 | PGSC0003DMG40003039 | NADH dehydrogenase | 1127764 |
| ch06 | 58657005 | 58659956 | 58658481 | PGSC0003DMG40002007 | Glutathione transferase | 1130513.5 |
| ch06 | 58660552 | 58662650 | 58661601 | PGSC0003DMG40002015( | 60S ribosomal protein L23 | 1133634 |
| ch06 | 56393801 | 56394491 | 56394146 | PGSC0003DMG40303031 | Histone H2B | 1133821 |
| ch06 | 58663416 | 58669546 | 58666481 | PGSC0003DMG40002014 | Succinyl CoA ligase beta subunit | 1138514 |
| ch06 | 58669933 | 58671770 | 58670852 | PGSC0003DMG40202007 | Desiccation-associated protein | 1142884.5 |
| ch06 | 56383260 | 56384106 | 56383683 | PGSC0003DMG40103031 | ZPT2-12 | 1144284 |
| ch06 | 58672068 | 58677026 | 58674547 | PGSC0003DMG40102007 | Glycolipid transfer protein | 1146580 |
| ch06 | 56379375 | 56379923 | 56379649 | PGSC0003DMG40003031 | ZPT2-8 | 1148318 |
| ch06 | 58678579 | 58681102 | 58679841 | PGSC0003DMG40002014 | Glycoprotein | 1151873.5 |
| ch06 | 58681668 | 58689067 | 58685368 | PGSC0003DMG40002007 | Casein kinase | 1157400.5 |
| ch06 | 58697805 | 58699947 | 58698876 | PGSC0003DMG40002014 | NAC domain protein | 1170909 |
| ch06 | 56329703 | 56332793 | 56331248 | PGSC0003DMG40003030 | Auxin-regulated protein | 1196719 |
| ch06 | 58733409 | 58736573 | 58734991 | PGSC0003DMG40002014 | Structural maintenance of chromosome 1 protein | 1207024 |
| ch06 | 58737145 | 58743220 | 58740183 | PGSC0003DMG40002007 | Step II splicing factor slu7 | 1212215.5 |
| ch06 | 56314781 | 56316561 | 56315671 | PGSC0003DMG40003039 | Isoform MYB48-1 of Transcription factor MYB48 | 1212296 |
| ch06 | 56310987 | 56312173 | 56311580 | PGSC0003DMG40003030 | Nodulin-26 | 1216387 |
| ch06 | 56304924 | 56308549 | 56306737 | PGSC0003DMG40003030 | Male sterility MS5 | 1221230.5 |
| ch06 | 56299197 | 56302118 | 56300658 | PGSC0003DMG40003039 | 60S ribosomal protein L35 | 1227309.5 |
| ch06 | 58756763 | 58758225 | 58757494 | PGSC0003DMG40002007 | MYB8 | 1229527 |
| ch06 | 56283292 | 56285669 | 56284481 | PGSC0003DMG40003039 | Ankyrin repeat-containing protein | 1243486.5 |

| | | | | | | |
|---|---|---|---|---|---|---|
| ch06 | 58772076 | 58773812 | 58772944 | PGSC0003DMG400020070 | 1-aminocyclopropane-1-carboxylate oxidase | 1244977 |
| ch06 | 58776636 | 58782360 | 58779498 | PGSC0003DMG400020145 | Triacylglycerol lipase | 1251531 |
| ch06 | 56269795 | 56282772 | 56276284 | PGSC0003DMG400030306 | CCR4-NOT transcription complex subunit | 1251683.5 |
| ch06 | 56243692 | 56251283 | 56247488 | PGSC0003DMG400030391 | Bipolar kinesin KRP-130 | 1280479.5 |
| ch06 | 56238355 | 56242764 | 56240560 | PGSC0003DMG400030390 | NOX1 | 1287407.5 |
| ch06 | 58821631 | 58822665 | 58822148 | PGSC0003DMG400020144 | Mucin | 1294181 |
| ch06 | 58826305 | 58830434 | 58828370 | PGSC0003DMG400020066 | BHLH domain class transcription factor | 1300402.5 |
| ch06 | 58831176 | 58836385 | 58833781 | PGSC0003DMG400020142 | Protein mrp | 1305813.5 |
| ch06 | 58837026 | 58845589 | 58841308 | PGSC0003DMG400020065 | Poly(RC)-binding protein | 1313340.5 |
| ch06 | 58854543 | 58855689 | 58855116 | PGSC0003DMG400020064 | Histone H1 | 1327149 |
| ch06 | 58857193 | 58862114 | 58859654 | PGSC0003DMG400020063 | S-adenosylmethionine-dependent methyltransferase | 1331686.5 |
| ch06 | 58863298 | 58863705 | 58863502 | PGSC0003DMG400020062 | Agenet domain-containing protein | 1335534.5 |
| ch06 | 58865552 | 58872528 | 58869040 | PGSC0003DMG400020061 | E3 ubiquitin protein ligase DRIP1 | 1341073 |
| ch06 | 58878641 | 58879841 | 58879241 | PGSC0003DMG400020141 | Photosystem II reaction center W protein, chloroplastic | 1351274 |
| ch06 | 58881091 | 58885812 | 58883452 | PGSC0003DMG400020140 | F-box/leucine rich repeat protein | 1355484.5 |
| ch06 | 56166080 | 56166412 | 56166246 | PGSC0003DMG400006609 | Narrow sheath protein | 1361721 |
| ch06 | 58890143 | 58891120 | 58890632 | PGSC0003DMG400020139 | Auxin-induced protein 22B | 1362664.5 |
| ch06 | 58892220 | 58894280 | 58893250 | PGSC0003DMG400020060 | Interferon-induced guanylate-binding protein | 1365283 |
| ch06 | 58899896 | 58901447 | 58900672 | PGSC0003DMG402020138 | Beta-ketoacyl-coa synthase family protein | 1372704.5 |
| ch06 | 58903342 | 58906004 | 58904673 | PGSC0003DMG401020138 | Histone H2A variant 1 | 1376706 |
| ch06 | 58913467 | 58917383 | 58915425 | PGSC0003DMG401020059 | Protein TIFY 4B | 1387458 |
| ch06 | 56138222 | 56140469 | 56139346 | PGSC0003DMG400006603 | Transferase, transferring glycosyl groups | 1388621.5 |
| ch06 | 58921480 | 58922635 | 58922058 | PGSC0003DMG400020137 | Transmembrane BAX inhibitor motif-containing protein | 1394090.5 |
| ch06 | 56124422 | 56129994 | 56127208 | PGSC0003DMG402006611 | Hydrolase | 1400759 |
| ch06 | 58936957 | 58943104 | 58940031 | PGSC0003DMG400020136 | Sulfate/bicarbonate/oxalate exchanger and transporter sat-1 | 1412063.5 |
| ch06 | 58949370 | 58953019 | 58951195 | PGSC0003DMG400020135 | O-sialoglycoprotein endopeptidase | 1423227.5 |
| ch06 | 58958198 | 58962574 | 58960386 | PGSC0003DMG400020134 | Small nuclear ribonucleoprotein sm d1 | 1432419 |
| ch06 | 56089078 | 56092424 | 56090751 | PGSC0003DMG400006604 | Apetala 2 | 1437216 |
| ch06 | 56084617 | 56088096 | 56086357 | PGSC0003DMG400006605 | RNA 3' terminal phosphate cyclase | 1441610.5 |
| ch06 | 58972011 | 58972501 | 58972256 | PGSC0003DMG400020057 | Cysteine-rich protein | 1444289 |
| ch06 | 58973431 | 58974725 | 58974078 | PGSC0003DMG402020132 | Non-specific lipid-transfer protein | 1446111 |
| ch06 | 59003004 | 59006851 | 59004928 | PGSC0003DMG400020130 | Protein transport protein Sec24C | 1476960.5 |
| ch06 | 59011300 | 59012273 | 59011787 | PGSC0003DMG402020055 | 40S ribosomal protein S24 | 1483819.5 |
| ch06 | 59030077 | 59036300 | 59033189 | PGSC0003DMG400020053 | Copalyl diphosphate synthase | 1505221.5 |
| ch06 | 59046945 | 59048821 | 59047883 | PGSC0003DMG400020128 | Patatin T5 | 1519916 |

| | | | | | | |
|---|---|---|---|---|---|---|
| ch06 | 56007197 | 56008144 | 56007671 | PGSC0003DMG400007512 | Nitrate excretion transporter 2 | 1520296.5 |
| ch06 | 55971792 | 55976592 | 55974192 | PGSC0003DMG400007508 | Major Facilitator Superfamily protein | 1553775 |
| ch06 | 59078775 | 59101491 | 59090133 | PGSC0003DMG401020126 | Armadillo/beta-catenin repeat family protein | 1562166 |
| ch06 | 59089122 | 59097623 | 59093373 | PGSC0003DMG400020127 | Chalcone isomerase | 1565405.5 |
| ch06 | 55952230 | 55961384 | 55956807 | PGSC0003DMG400007498 | Vacuolar proton atpase | 1571160 |
| ch06 | 59098338 | 59101102 | 59099720 | PGSC0003DMG402020126 | Armadillo/beta-catenin repeat family protein / BTB/POZ domain-containing protein | 1571753 |
| ch06 | 59101980 | 59109840 | 59105910 | PGSC0003DMG400020125 | Nucleolar GTP-binding protein | 1577943 |
| ch06 | 55944618 | 55951403 | 55948011 | PGSC0003DMG400007507 | 3'-5'-exoribonuclease/RNA binding | 1579956.5 |
| ch06 | 55935605 | 55937762 | 55936684 | PGSC0003DMG400007506 | DOF domain class transcription factor | 1591283.5 |
| ch06 | 55922946 | 55926333 | 55924640 | PGSC0003DMG400007505 | Senescence-associated protein | 1603327.5 |
| ch06 | 55920368 | 55924144 | 55922256 | PGSC0003DMG400007497 | TRNA-splicing endonuclease subunit Sen2-2 | 1605711 |
| ch06 | 59138371 | 59138859 | 59138615 | PGSC0003DMG400020049 | Outer envelope membrane protein | 1610648 |
| ch06 | 55904773 | 55910691 | 55907732 | PGSC0003DMG400007504 | Carbamoyl phosphate synthase large subunit | 1620235 |
| ch06 | 59150535 | 59153223 | 59151879 | PGSC0003DMG400020123 | AAA ATPase containing von Willebrand factor type A | 1623912 |
| ch06 | 59158020 | 59159136 | 59158578 | PGSC0003DMG400020122 | Circadian clock coupling factor ZGT | 1630611 |
| ch06 | 55889622 | 55891351 | 55890487 | PGSC0003DMG401007500 | Vicilin | 1637480.5 |
| ch06 | 55887840 | 55890547 | 55889194 | PGSC0003DMG400007496 | Adenylate kinase isoenzyme 6 | 1638773.5 |
| ch06 | 59166918 | 59170937 | 59168928 | PGSC0003DMG400020045 | Speckle-type POZ protein | 1640960.5 |
| ch06 | 59193490 | 59197152 | 59195321 | PGSC0003DMG400020118 | Oxidoreductase | 1667354 |
| ch06 | 55856610 | 55857687 | 55857149 | PGSC0003DMG400003371 | Arabinogalactan protein 2 | 1670818.5 |
| ch06 | 59197495 | 59201044 | 59199270 | PGSC0003DMG401020044 | ER auxin binding protein 1 | 1671302.5 |
| ch06 | 55851346 | 55854976 | 55853161 | PGSC0003DMG400003370 | Protein disulfide isomerase | 1674806 |
| ch06 | 59203792 | 59204909 | 59204351 | PGSC0003DMG402020117 | Methyl binding domain protein | 1676383.5 |
| ch06 | 59207716 | 59212039 | 59209878 | PGSC0003DMG401020117 | Histidine phosphotransfer protein | 1681910.5 |
| ch06 | 55837541 | 55838284 | 55837913 | PGSC0003DMG400003368 | 60S ribosomal protein L12 | 1690054.5 |
| ch06 | 59222168 | 59223944 | 59223056 | PGSC0003DMG400020178 | Serine-threonine protein kinase, plant-type | 1695089 |
| ch06 | 55830386 | 55830798 | 55830592 | PGSC0003DMG400003375 | NADH-ubiquinone oxidoreductase chain 2 | 1697375 |
| ch06 | 59230710 | 59232156 | 59231433 | PGSC0003DMG400024029 | Histone H2A | 1703466 |
| ch06 | 59232572 | 59237366 | 59234969 | PGSC0003DMG400024040 | NPL4 family protein | 1707002 |
| ch06 | 55810550 | 55814998 | 55812774 | PGSC0003DMG400024322 | SIT4 phosphatase-associated family protein | 1715193 |
| ch06 | 59246145 | 59249374 | 59247760 | PGSC0003DMG400024030 | Gtpase activating protein | 1719792.5 |
| ch06 | 55805067 | 55808251 | 55806659 | PGSC0003DMG400024320 | Auxin response factor 16 | 1721308 |
| ch06 | 59250255 | 59256905 | 59253580 | PGSC0003DMG401024041 | Ribonucleoprotein | 1725613 |
| ch06 | 55797189 | 55801725 | 55799457 | PGSC0003DMG400024319 | GATA-1 zinc finger protein | 1728510 |

| | | | | | | |
|---|---|---|---|---|---|---|
| ch06 | 59259214 | 59264255 | 59261735 | PGSC0003DMG402024041 | Shikimate dehydrogenase | 1733767.5 |
| ch06 | 55788699 | 55796265 | 55792482 | PGSC0003DMG400024318 | Pleckstrin homology (PH) domain-containing protein / lipid-binding START domain-containing protein | 1735485 |
| ch06 | 55777231 | 55784144 | 55780688 | PGSC0003DMG400024317 | Storage protein | 1747279.5 |
| ch06 | 59275353 | 59295069 | 59285211 | PGSC0003DMG401024031 | Serine endopeptidase degp2 | 1757244 |
| ch06 | 59295548 | 59296160 | 59295854 | PGSC0003DMG400024032 | Classical arabinogalactan protein 26 | 1767887 |
| ch06 | 59298298 | 59301733 | 59300016 | PGSC0003DMG400024033 | Transport inhibitor response 1 | 1772048.5 |
| ch06 | 55743306 | 55748450 | 55745878 | PGSC0003DMG400024321 | Mutt/nudix hydrolase | 1782089 |
| ch06 | 59315293 | 59322145 | 59318719 | PGSC0003DMG400024045 | Cullin 1B | 1790752 |
| ch06 | 55730289 | 55736311 | 55733300 | PGSC0003DMG400024315 | Nectarin-2 | 1794667 |
| ch06 | 59347791 | 59348560 | 59348176 | PGSC0003DMG400024050 | Reverse transcriptase | 1820208.5 |
| ch06 | 59359342 | 59360748 | 59360045 | PGSC0003DMG400024035 | Fiber protein Fb19 | 1832078 |
| ch06 | 59361466 | 59364902 | 59363184 | PGSC0003DMG400024046 | Protein translocase | 1835217 |
| ch06 | 55652701 | 55654197 | 55653449 | PGSC0003DMG400008794 | Major latex | 1874518 |
| ch06 | 59408564 | 59415544 | 59412054 | PGSC0003DMG400024037 | Aminotransferase ybdL | 1884087 |
| ch06 | 59476003 | 59476545 | 59476274 | PGSC0003DMG400024039 | 30S ribosomal protein S4, chloroplastic | 1948307 |
| ch06 | 55574774 | 55583712 | 55579243 | PGSC0003DMG400012001 | White-brown-complex ABC transporter family | 1948724 |
| ch06 | 55568679 | 55573501 | 55571090 | PGSC0003DMG400011997 | CPN60A | 1956877 |
| ch06 | 55535257 | 55537119 | 55536188 | PGSC0003DMG401011998 | High-affinity nitrate transporter | 1991779 |

| chr | start | end | mid | ID | Name | hit_dist |
|---|---|---|---|---|---|---|
| ch09 | 3919011 | 3927016 | 3923014 | PGSC0003DMG400002720 | Delta14-sterol reductase | 2983.5 |
| ch09 | 3930789 | 3935360 | 3933075 | PGSC0003DMG400002684 | Histone deacetylase HDT1 | 13044.5 |
| ch09 | 3942320 | 3947552 | 3944936 | PGSC0003DMG402002721 | Enolase | 24906 |
| ch09 | 3888677 | 3898320 | 3893499 | PGSC0003DMG400002719 | Repressor of silencing 1 | 26531.5 |
| ch09 | 3953064 | 3955006 | 3954035 | PGSC0003DMG401002721 | Peroxiredoxin | 34005 |
| ch09 | 3957641 | 3964503 | 3961072 | PGSC0003DMG400002722 | Cellulose synthase-like A1 | 41042 |
| ch09 | 3849902 | 3856182 | 3853042 | PGSC0003DMG401002683 | Heat stress transcription factor A3 | 66988 |
| ch09 | 3996218 | 4000730 | 3998474 | PGSC0003DMG400002725 | DNA binding protein | 78444 |
| ch09 | 3834289 | 3837465 | 3835877 | PGSC0003DMG400002682 | Phytosulfokine peptide | 84153 |
| ch09 | 3811715 | 3815312 | 3813514 | PGSC0003DMG400002716 | Gamma-tubulin complex component | 106516.5 |
| ch09 | 4028005 | 4032971 | 4030488 | PGSC0003DMG400002687 | Geranyl geranyl pyrophosphate synthase | 110458 |
| ch09 | 3805472 | 3806977 | 3806225 | PGSC0003DMG400042886 | ATP binding protein | 113805.5 |
| ch09 | 4035115 | 4037057 | 4036086 | PGSC0003DMG400002726 | Cytochrome P450 | 116056 |
| ch09 | 4046703 | 4049119 | 4047911 | PGSC0003DMG400002688 | Cytochrome P450 76A1 | 127881 |
| ch09 | 3786204 | 3795284 | 3790744 | PGSC0003DMG400002681 | OO_Ba0013J05-OO_Ba0033A15.20 protein | 129286 |
| ch09 | 3767865 | 3771494 | 3769680 | PGSC0003DMG400002713 | ACI14 | 150350.5 |
| ch09 | 4071465 | 4072193 | 4071829 | PGSC0003DMG400042439 | Transcription regulation | 151799 |
| ch09 | 3764077 | 3766397 | 3765237 | PGSC0003DMG400002680 | Heat shock protein binding protein | 154793 |
| ch09 | 4076521 | 4076946 | 4076734 | PGSC0003DMG400040607 | F-box protein | 156703.5 |
| ch09 | 4077439 | 4078800 | 4078120 | PGSC0003DMG400045355 | Diacylglycerol kinase eta | 158089.5 |
| ch09 | 4088416 | 4091351 | 4089884 | PGSC0003DMG400002689 | Receptor kinase | 169853.5 |
| ch09 | 4094029 | 4101896 | 4097963 | PGSC0003DMG400002727 | Homeodomain-like | 177932.5 |
| ch09 | 4106395 | 4114036 | 4110216 | PGSC0003DMG400002690 | Pyruvate kinase | 190185.5 |
| ch09 | 3726996 | 3729484 | 3728240 | PGSC0003DMG400002679 | GRF domain class transcription factor | 191790 |
| ch09 | 4116215 | 4117299 | 4116757 | PGSC0003DMG400002728 | Homeodomain | 196727 |
| ch09 | 4130843 | 4138182 | 4134513 | PGSC0003DMG400002691 | ATPP2-A13 | 214482.5 |
| ch09 | 4156189 | 4159105 | 4157647 | PGSC0003DMG400002692 | Homeobox protein | 237617 |
| ch09 | 4160572 | 4163365 | 4161969 | PGSC0003DMG400002729 | Ribosomal protein L24 | 241938.5 |
| ch09 | 4165168 | 4171658 | 4168413 | PGSC0003DMG400002693 | Srpk | 248383 |
| ch09 | 3658371 | 3658775 | 3658573 | PGSC0003DMG400002676 | Dehydration-responsive element-binding protein 1B | 261457 |
| ch09 | 4195814 | 4197685 | 4196750 | PGSC0003DMG400002731 | LEA protein group 3 | 276719.5 |
| ch09 | 4199867 | 4200806 | 4200337 | PGSC0003DMG400002732 | VQ motif-containing protein | 280306.5 |
| ch09 | 3631615 | 3635107 | 3633361 | PGSC0003DMG400002710 | Transcription factor | 286669 |
| ch09 | 3617855 | 3619986 | 3618921 | PGSC0003DMG400002675 | Fructose-bisphosphate aldolase | 301109.5 |

| | | | | | | |
|---|---|---|---|---|---|---|
| ch09 | 3610664 | 3611117 | 3610891 | PGSC0003DMG400002674 | Integrase core domain containing protein | 309139.5 |
| ch09 | 3605730 | 3612142 | 3608936 | PGSC0003DMG400002673 | Protein ELC | 311094 |
| ch09 | 3573687 | 3578971 | 3576329 | PGSC0003DMG400002708 | Ankyrin repeat domain-containing protein EMB506, chloroplastic | 343701 |
| ch09 | 3569661 | 3573576 | 3571619 | PGSC0003DMG400002671 | Protein G10 | 348411.5 |
| ch09 | 4296272 | 4301454 | 4298863 | PGSC0003DMG400012984 | GTP-binding protein engB | 378833 |
| ch09 | 4305336 | 4306376 | 4305856 | PGSC0003DMG402012985 | Glycosyltransferase | 385826 |
| ch09 | 3524983 | 3531231 | 3528107 | PGSC0003DMG400002669 | Meiotic recombination repair protein | 391923 |
| ch09 | 3523410 | 3524522 | 3523966 | PGSC0003DMG400045693 | F-box protein family | 396064 |
| ch09 | 4328377 | 4329124 | 4328751 | PGSC0003DMG400038079 | Zinc finger protein | 408720.5 |
| ch09 | 4331846 | 4335660 | 4333753 | PGSC0003DMG400012980 | FAD binding protein | 413723 |
| ch09 | 4336457 | 4342614 | 4339536 | PGSC0003DMG400012987 | Threonine dehydratase biosynthetic, chloroplastic | 419505.5 |
| ch09 | 4352151 | 4362118 | 4357135 | PGSC0003DMG402012981 | Serine-threonine protein kinase, plant-type | 437104.5 |
| ch09 | 4363478 | 4369003 | 4366241 | PGSC0003DMG403012981 | DNA replication complex GINS protein PSF1 | 446210.5 |
| ch09 | 4369320 | 4376418 | 4372869 | PGSC0003DMG400012988 | Polyadenylate-binding protein | 452839 |
| ch09 | 4391620 | 4400527 | 4396074 | PGSC0003DMG400012989 | Aspartic proteinase Asp1 | 476043.5 |
| ch09 | 3432260 | 3434590 | 3433425 | PGSC0003DMG400002706 | Vacuolar protein sorting 13C protein | 486605 |
| ch09 | 4412463 | 4413542 | 4413003 | PGSC0003DMG400012983 | Pollen ole e 1 allergen and extensin family protein | 492972.5 |
| ch09 | 4463156 | 4463671 | 4463414 | PGSC0003DMG400046725 | Guanine-nucleotide binding protein | 543383.5 |
| ch09 | 3344754 | 3349807 | 3347281 | PGSC0003DMG400002664 | Serine/threonine protein kinase | 572749.5 |
| ch09 | 3322259 | 3327483 | 3324871 | PGSC0003DMG400002703 | Monodehydroascorbate reductase | 595159 |
| ch09 | 3321461 | 3322085 | 3321773 | PGSC0003DMG400002663 | APO protein 3, mitochondrial | 598257 |
| ch09 | 4559716 | 4563700 | 4561708 | PGSC0003DMG400003880 | Leucoanthocyanidin dioxygenase | 641678 |
| ch09 | 3268985 | 3273075 | 3271030 | PGSC0003DMG402002661 | FtsZ | 649000 |
| ch09 | 4579283 | 4580779 | 4580031 | PGSC0003DMG400003879 | Uracil permease | 660001 |
| ch09 | 4582541 | 4583950 | 4583246 | PGSC0003DMG400043390 | Hydrolase | 663215.5 |
| ch09 | 3242899 | 3243959 | 3243429 | PGSC0003DMG401002701 | Iron-sulfur cluster assembly protein | 676601 |
| ch09 | 4594241 | 4600175 | 4597208 | PGSC0003DMG400003903 | ATSMC2 | 677178 |
| ch09 | 3223202 | 3226836 | 3225019 | PGSC0003DMG403002701 | F-box/LRR-repeat protein | 695011 |
| ch09 | 3218108 | 3220290 | 3219199 | PGSC0003DMG400002700 | F-box family protein | 700831 |
| ch09 | 4627066 | 4628833 | 4627950 | PGSC0003DMG400003901 | UDP-glucose:glucosyltransferase | 707919.5 |
| ch09 | 4630113 | 4631132 | 4630623 | PGSC0003DMG400003878 | UDP-glucosyltransferase | 710592.5 |
| ch09 | 3193379 | 3200094 | 3196737 | PGSC0003DMG401002659 | Esterase/lipase superfamily protein | 723293.5 |
| ch09 | 4670703 | 4671360 | 4671032 | PGSC0003DMG400003910 | Non-specific lipid-transfer protein | 751001.5 |
| ch09 | 3143568 | 3145963 | 3144766 | PGSC0003DMG400040097 | SP9D | 775264.5 |
| ch09 | 3124523 | 3129689 | 3127106 | PGSC0003DMG401002698 | Fatty acyl-CoA reductase 2 | 792924 |

| | | | | | | |
|---|---|---|---|---|---|---|
| ch09 | 3093428 | 3094597 | 3094013 | PGSC0003DMG400044298 | Plant disease resistant protein | 826017.5 |
| ch09 | 3090742 | 3091761 | 3091252 | PGSC0003DMG400002697 | Stress-associated protein 8 | 828778.5 |
| ch09 | 3084678 | 3089288 | 3086983 | PGSC0003DMG400002655 | Purple acid phosphatase 22 | 833047 |
| ch09 | 3076086 | 3079625 | 3077856 | PGSC0003DMG400002696 | Purple acid phosphatase 20 | 842174.5 |
| ch09 | 4766730 | 4767880 | 4767305 | PGSC0003DMG402003900 | U3 small nucleolar RNA-associated protein | 847275 |
| ch09 | 3051942 | 3060272 | 3056107 | PGSC0003DMG401002654 | Farnesylated protein-converting enzyme 2 | 863923 |
| ch09 | 4792637 | 4793994 | 4793316 | PGSC0003DMG402003899 | PIP5K9 | 873285.5 |
| ch09 | 4797026 | 4801131 | 4799079 | PGSC0003DMG401003899 | Phosphatidylinositol-4-phosphate 5-kinase | 879048.5 |
| ch09 | 4801232 | 4805760 | 4803496 | PGSC0003DMG400003874 | Splicing factor 3A subunit | 883466 |
| ch09 | 4812445 | 4818046 | 4815246 | PGSC0003DMG400003898 | RAB1X | 895215.5 |
| ch09 | 4818292 | 4822369 | 4820331 | PGSC0003DMG400003873 | Ankyrin repeat-containing protein | 900300.5 |
| ch09 | 4829603 | 4830786 | 4830195 | PGSC0003DMG400003872 | ZPT2-11 | 910164.5 |
| ch09 | 4843300 | 4843812 | 4843556 | PGSC0003DMG400039537 | Mads box protein | 923526 |
| ch09 | 4864072 | 4870265 | 4867169 | PGSC0003DMG400003897 | PGPD14 | 947138.5 |
| ch09 | 4871399 | 4877917 | 4874658 | PGSC0003DMG400037605 | Carbonic anhydrase | 954628 |
| ch09 | 4881150 | 4889479 | 4885315 | PGSC0003DMG400003896 | Peptidyl-prolyl cis-trans isomerase cyclophilin-type family protein | 965284.5 |
| ch09 | 2951568 | 2956460 | 2954014 | PGSC0003DMG401008964 | Solute carrier family 35, member F1 | 966016 |
| ch09 | 2937043 | 2946288 | 2941666 | PGSC0003DMG400008963 | Tata-associated factor II 58 | 978364.5 |
| ch09 | 2928778 | 2934859 | 2931819 | PGSC0003DMG400008962 | Inosine-uridine preferring nucleoside hydrolase | 988211.5 |
| ch09 | 4914082 | 4917121 | 4915602 | PGSC0003DMG400003871 | Pectate lyase | 995571.5 |
| ch09 | 2915590 | 2925490 | 2920540 | PGSC0003DMG400008961 | Carboxylic ester hydrolase | 999490 |
| ch09 | 4935966 | 4937654 | 4936810 | PGSC0003DMG400045310 | Nodulin family protein | 1016780 |
| ch09 | 4938991 | 4944221 | 4941606 | PGSC0003DMG400003894 | D8Ertd354e protein | 1021576 |
| ch09 | 4946791 | 4951876 | 4949334 | PGSC0003DMG400003868 | Amino acid binding protein | 1029303.5 |
| ch09 | 2868109 | 2868809 | 2868459 | PGSC0003DMG400008958 | Ubiquitin carrier protein | 1051571 |
| ch09 | 4969817 | 4974200 | 4972009 | PGSC0003DMG402003893 | Zinc finger family protein | 1051978.5 |
| ch09 | 4997614 | 5000435 | 4999025 | PGSC0003DMG400003866 | Xyloglucan endotransglucosylase/hydrolase 12 | 1078994.5 |
| ch09 | 2836765 | 2837139 | 2836952 | PGSC0003DMG400008977 | Pre-mRNA-splicing factor cwc24 | 1083078 |
| ch09 | 2815705 | 2824432 | 2820069 | PGSC0003DMG400008957 | Cucumisin | 1099961.5 |
| ch09 | 5027768 | 5032848 | 5030308 | PGSC0003DMG400003892 | Molybdopterin-binding | 1110278 |
| ch09 | 5033204 | 5035024 | 5034114 | PGSC0003DMG400045602 | Pentatricopeptide repeat-containing protein | 1114084 |
| ch09 | 2800029 | 2804470 | 2802250 | PGSC0003DMG400008882 | Calmodulin binding protein | 1117780.5 |
| ch09 | 5036405 | 5039710 | 5038058 | PGSC0003DMG400003891 | Thioredoxin | 1118027.5 |
| ch09 | 5041031 | 5042203 | 5041617 | PGSC0003DMG400037250 | S-adenosylmethionine synthase 2 | 1121587 |
| ch09 | 2791921 | 2793475 | 2792698 | PGSC0003DMG400008956 | Glucosamine 6-phosphate N-acetyltransferase | 1127332 |

| | | | | | | |
|---|---|---|---|---|---|---|
| ch09 | 5087875 | 5089659 | 5088767 | PGSC0003DMG400003890 | Transcription factor DcMYB4 | 1168737 |
| ch09 | 5129152 | 5136729 | 5132941 | PGSC0003DMG400003889 | P-glycoprotein | 1212910.5 |
| ch09 | 2693540 | 2703978 | 2698759 | PGSC0003DMG400008954 | Carbonic anhydrase family protein | 1221271 |
| ch09 | 5143194 | 5146624 | 5144909 | PGSC0003DMG400003863 | Zinc finger protein 593 | 1224879 |
| ch09 | 5159990 | 5162335 | 5161163 | PGSC0003DMG400003888 | Mannan synthase | 1241132.5 |
| ch09 | 2671505 | 2672947 | 2672226 | PGSC0003DMG400008952 | Glutaredoxin family protein | 1247804 |
| ch09 | 5176268 | 5179031 | 5177650 | PGSC0003DMG400003887 | Farnesylated protein | 1257619.5 |
| ch09 | 2633920 | 2641471 | 2637696 | PGSC0003DMG400008877 | Nucleolar phosphoprotein | 1282334.5 |
| ch09 | 5208520 | 5220413 | 5214467 | PGSC0003DMG400003886 | Acylphosphatase | 1294436.5 |
| ch09 | 2619469 | 2622482 | 2620976 | PGSC0003DMG400008876 | Nucleic acid-binding, OB-fold | 1299054.5 |
| ch09 | 5224541 | 5225099 | 5224820 | PGSC0003DMG400003885 | SAUR family protein | 1304790 |
| ch09 | 2609470 | 2618505 | 2613988 | PGSC0003DMG401008875 | ABC transporter family, retinal flippase subfamily | 1306042.5 |
| ch09 | 2607522 | 2608451 | 2607987 | PGSC0003DMG400008874 | Cyclin-like F-box | 1312043.5 |
| ch09 | 5238175 | 5240588 | 5239382 | PGSC0003DMG400003883 | Tbp-associated factor taf | 1319351.5 |
| ch09 | 2589906 | 2598035 | 2593971 | PGSC0003DMG400008871 | Signal recognition particle protein | 1326059.5 |
| ch09 | 5248091 | 5256466 | 5252279 | PGSC0003DMG400003882 | Phosphoglycerate kinase | 1332248.5 |
| ch09 | 2580946 | 2583843 | 2582395 | PGSC0003DMG402008870 | Magnesium ion binding protein | 1337635.5 |
| ch09 | 2574867 | 2580006 | 2577437 | PGSC0003DMG401008870 | Rac GTPase activating protein 1 | 1342593.5 |
| ch09 | 5260643 | 5267305 | 5263974 | PGSC0003DMG400003859 | ATP-dependent RNA helicase | 1343944 |
| ch09 | 5267599 | 5271002 | 5269301 | PGSC0003DMG400021559 | RNA helicase | 1349270.5 |
| ch09 | 2548789 | 2551005 | 2549897 | PGSC0003DMG400008949 | Cullin 1B | 1370133 |
| ch09 | 2545692 | 2548009 | 2546851 | PGSC0003DMG400008868 | Desacetoxyvindoline 4-hydroxylase | 1373179.5 |
| ch09 | 2529981 | 2532190 | 2531086 | PGSC0003DMG400008867 | 50S ribosomal protein L7/L12 | 1388944.5 |
| ch09 | 5325988 | 5330633 | 5328311 | PGSC0003DMG400021556 | Transitional endoplasmic reticulum ATPase | 1408280.5 |
| ch09 | 2490499 | 2499108 | 2494804 | PGSC0003DMG400008945 | Bipolar kinesin KRP-130 | 1425226.5 |
| ch09 | 5356245 | 5363388 | 5359817 | PGSC0003DMG400021553 | RNA polymerase sigma factor rpoD | 1439786.5 |
| ch09 | 2478536 | 2478850 | 2478693 | PGSC0003DMG400008974 | Invertase | 1441337 |
| ch09 | 5366727 | 5367739 | 5367233 | PGSC0003DMG400021568 | Proliferating cell nuclear antigen gene-controlling element binding factor | 1447203 |
| ch09 | 5368445 | 5375156 | 5371801 | PGSC0003DMG400021552 | Cysteine-type peptidase | 1451770.5 |
| ch09 | 2457978 | 2460563 | 2459271 | PGSC0003DMG400008865 | Ribosomal protein S14 | 1460759.5 |
| ch09 | 2438315 | 2447127 | 2442721 | PGSC0003DMG400008862 | Sorting nexin 3 | 1477309 |
| ch09 | 2419913 | 2429073 | 2424493 | PGSC0003DMG400008940 | Short-chain dehydrogenase | 1495537 |
| ch09 | 5427150 | 5434231 | 5430691 | PGSC0003DMG400021551 | Protein kinase APK1A, chloroplast | 1510660.5 |
| ch09 | 2396033 | 2398318 | 2397176 | PGSC0003DMG400008861 | Vacuolar protein sorting 55 containing protein | 1522854.5 |

| | | | | | | |
|---|---|---|---|---|---|---|
| ch09 | 2376254 | 2391773 | 2384014 | PGSC0003DMG400008939 | UDP-N-acetylglucosamine--peptide N-acetylglucosaminyltransferase SPINDLY | 1536016.5 |
| ch09 | 2361998 | 2367446 | 2364722 | PGSC0003DMG400008860 | Peptidyl-prolyl cis-trans isomerase | 1555308 |
| ch09 | 2359223 | 2360688 | 2359956 | PGSC0003DMG400008859 | UPF0497 membrane protein 9 | 1560074.5 |
| ch09 | 2345621 | 2351097 | 2348359 | PGSC0003DMG400008938 | Endo-1,4-beta-glucanase | 1571671 |
| ch09 | 5493862 | 5498839 | 5496351 | PGSC0003DMG400021550 | Adenosine kinase isoform 1T | 1576320.5 |
| ch09 | 5502194 | 5505885 | 5504040 | PGSC0003DMG402021549 | Phenylalanine ammonia-lyase | 1584009.5 |
| ch09 | 5550240 | 5558623 | 5554432 | PGSC0003DMG400021547 | Ethylene signaling protein | 1634401.5 |
| ch09 | 2264372 | 2267937 | 2266155 | PGSC0003DMG400008935 | Intron maturase, type II family protein | 1653875.5 |
| ch09 | 2253315 | 2262393 | 2257854 | PGSC0003DMG400008857 | Dcp1-like decapping family | 1662176 |
| ch09 | 5584978 | 5591550 | 5588264 | PGSC0003DMG400021563 | Calcium lipid binding protein | 1668234 |
| ch09 | 5594175 | 5598929 | 5596552 | PGSC0003DMG400021562 | Single-stranded DNA binding protein | 1676522 |
| ch09 | 2238168 | 2239382 | 2238775 | PGSC0003DMG400008971 | Nodulin-26 | 1681255 |
| ch09 | 2207973 | 2215599 | 2211786 | PGSC0003DMG400008934 | Spliceosome associated protein | 1708244 |
| ch09 | 5628981 | 5631704 | 5630343 | PGSC0003DMG400038158 | Cyclic nucleotide-gated ion channel 15 | 1710312.5 |
| ch09 | 2202612 | 2205636 | 2204124 | PGSC0003DMG400008855 | Transcription factor BTF3 | 1715906 |
| ch09 | 5646681 | 5651020 | 5648851 | PGSC0003DMG400021561 | Cytokinin riboside 5'-monophosphate phosphoribohydrolase LOG3 | 1728820.5 |
| ch09 | 2189668 | 2191621 | 2190645 | PGSC0003DMG400045841 | SRR380 | 1729385.5 |
| ch09 | 5660041 | 5661839 | 5660940 | PGSC0003DMG400043102 | Non-phototropic hypocotyl | 1740910 |
| ch09 | 2170462 | 2173408 | 2171935 | PGSC0003DMG400008854 | Nodulin | 1748095 |
| ch09 | 2153784 | 2158868 | 2156326 | PGSC0003DMG400008853 | Small GTP-binding protein | 1763704 |
| ch09 | 5682392 | 5686326 | 5684359 | PGSC0003DMG400021560 | Auxin response factor ARF16 | 1764329 |
| ch09 | 2142107 | 2145943 | 2144025 | PGSC0003DMG400008932 | Magnesium dependent soluble inorganic pyrophosphatase | 1776005 |
| ch09 | 5701627 | 5702180 | 5701904 | PGSC0003DMG400021571 | ATP-dependent Clp protease proteolytic subunit | 1781873.5 |
| ch09 | 2134346 | 2135450 | 2134898 | PGSC0003DMG400008930 | Histone H2A | 1785132 |
| ch09 | 2120565 | 2124035 | 2122300 | PGSC0003DMG400008929 | BRI1-KD interacting protein 130 | 1797730 |
| ch09 | 2111862 | 2120481 | 2116172 | PGSC0003DMG400008852 | Arginine biosynthesis bifunctional protein ArgJ beta chain | 1803858.5 |
| ch09 | 2095653 | 2099120 | 2097387 | PGSC0003DMG400008928 | Actin-depolymerizing factor 1 | 1822643.5 |
| ch09 | 2088131 | 2092685 | 2090408 | PGSC0003DMG400008927 | Pentatricopeptide repeat-containing protein, chloroplastic | 1829622 |
| ch09 | 2077110 | 2085336 | 2081223 | PGSC0003DMG400008926 | Eukaryotic translation initiation factor 3 subunit A | 1838807 |
| ch09 | 2075540 | 2075947 | 2075744 | PGSC0003DMG400042556 | NBS domain resistance protein | 1844286.5 |
| ch09 | 2074803 | 2075324 | 2075064 | PGSC0003DMG400045729 | Prf | 1844966.5 |
| ch09 | 2074057 | 2074638 | 2074348 | PGSC0003DMG400008925 | Disease resistance protein | 1845682.5 |
| ch09 | 2062700 | 2071946 | 2067323 | PGSC0003DMG400008924 | UNC93 | 1852707 |
| ch09 | 2052460 | 2054590 | 2053525 | PGSC0003DMG400043103 | LOB domain-containing protein 22 | 1866505 |

| | | | | | | |
|---|---|---|---|---|---|---|
| ch09 | 2046649 | 2049534 | 2048092 | PGSC0003DMG400008922 | DNA-directed RNA polymerase IIa | 1871938.5 |
| ch09 | 5791781 | 5792554 | 5792168 | PGSC0003DMG402020908 | Plasma intrinsic protein 2,1 | 1872137.5 |
| ch09 | 5809494 | 5813147 | 5811321 | PGSC0003DMG400020906 | Plasma membrane intrinsic protein | 1891290.5 |
| ch09 | 2021152 | 2025289 | 2023221 | PGSC0003DMG400008921 | Arf gtpase-activating protein | 1896809.5 |
| ch09 | 2015092 | 2020514 | 2017803 | PGSC0003DMG400008849 | Monovalent cation:proton antiporter | 1902227 |
| ch09 | 1995951 | 2002477 | 1999214 | PGSC0003DMG400008847 | 5-oxoprolinase | 1920816 |
| ch09 | 1983767 | 1988922 | 1986345 | PGSC0003DMG400008846 | P-type trefoil | 1933685.5 |
| ch09 | 1967427 | 1973922 | 1970675 | PGSC0003DMG400008845 | Singapore isolate B (sub-type 7) whole genome shotgun sequence assembly, scaffold_10 | 1949355.5 |
| ch09 | 1966115 | 1967038 | 1966577 | PGSC0003DMG400008918 | PP1/PP2A phosphatases pleiotropic regulator PRL1 | 1953453.5 |
| ch09 | 1958523 | 1961992 | 1960258 | PGSC0003DMG400008917 | Heat shock protein 70 | 1959772.5 |
| ch09 | 1955153 | 1957770 | 1956462 | PGSC0003DMG400008844 | SLT1 protein | 1963568.5 |
| ch09 | 5888349 | 5888919 | 5888634 | PGSC0003DMG400020913 | NL25 | 1968604 |
| ch09 | 1940928 | 1941721 | 1941325 | PGSC0003DMG400008916 | RING-H2 finger protein ATL2J | 1978705.5 |
| ch09 | 1932109 | 1937727 | 1934918 | PGSC0003DMG402008915 | Acetylglucosaminyltransferase | 1985112 |
| ch09 | 1924149 | 1931315 | 1927732 | PGSC0003DMG401008915 | Transcription factor DP, E2 dimerization partner protein | 1992298 |

| chr | start | end | mid | ID | Name | hit_dist |
|---|---|---|---|---|---|---|
| ch11 | 22621716 | 22625330 | 22623523 | PGSC0003DMG400041723 | chromo' domain containing protein | 1923128 |
| ch11 | 18769794 | 18776428 | 18773111 | PGSC0003DMG400002189 | DedA | 1927284 |
| ch11 | 18921453 | 18922659 | 18922056 | PGSC0003DMG400002185 | Ethylene-responsive transcription factor | 1778339 |
| ch11 | 20351502 | 20357592 | 20354547 | PGSC0003DMG400007646 | Eukaryotic initiation factor 4A-3 | 345848 |
| ch11 | 19458327 | 19463687 | 19461007 | PGSC0003DMG401028027 | FK506 binding protein | 1239388 |
| ch11 | 20563716 | 20568490 | 20566103 | PGSC0003DMG400041557 | Gag-pol polyprotein | 134292 |
| ch11 | 20904916 | 20906799 | 20905858 | PGSC0003DMG400034438 | Gag-pol protein | 205462.5 |
| ch11 | 20016762 | 20024953 | 20020858 | PGSC0003DMG400015909 | Glycyl-tRNA synthetase | 679537.5 |
| ch11 | 20530088 | 20539471 | 20534780 | PGSC0003DMG400019760 | Indigoidine synthase A family protein | 165615.5 |
| ch11 | 19974339 | 19979424 | 19976882 | PGSC0003DMG400037478 | Integrase core domain containing protein | 723513.5 |
| ch11 | 20684710 | 20686486 | 20685598 | PGSC0003DMG400019766 | Lignin-forming anionic peroxidase | 14797 |
| ch11 | 20937363 | 20939548 | 20938456 | PGSC0003DMG400042088 | LINE-type retrotransposon LIb DNA, complete sequence, Insertion at the S14 site | 238060.5 |
| ch11 | 20358151 | 20363517 | 20360834 | PGSC0003DMG400007647 | MAPKKK7 | 339561 |
| ch11 | 18776894 | 18782595 | 18779745 | PGSC0003DMG400002190 | Minichromosome maintenance factor | 1920651 |
| ch11 | 20700267 | 20705864 | 20703066 | PGSC0003DMG400019767 | Mitochondrial import inner membrane translocase subunit Tim17 | 2670.5 |
| ch11 | 20584130 | 20589150 | 20586640 | PGSC0003DMG400019765 | Polyprotein | 113755 |
| ch11 | 19452106 | 19456164 | 19454135 | PGSC0003DMG400028026 | Proton-dependent oligopeptide transport family protein | 1246260 |
| ch11 | 22466751 | 22467200 | 22466976 | PGSC0003DMG400047000 | Retrotransposon protein, Ty3-gypsy sub-class | 1766581 |
| ch11 | 20780547 | 20782538 | 20781543 | PGSC0003DMG400019764 | Ribosomal protein L41 | 81147.5 |
| ch11 | 20890365 | 20899827 | 20895096 | PGSC0003DMG400026862 | RNA recognition motif-containing protein | 194701 |
| ch11 | 20163156 | 20165011 | 20164084 | PGSC0003DMG400015911 | RNase H family protein | 536311.5 |
| ch11 | 20871344 | 20879315 | 20875330 | PGSC0003DMG400026863 | SET domain protein | 174934.5 |
| ch11 | 19466409 | 19470044 | 19468227 | PGSC0003DMG402028027 | Transaldolase | 1232169 |
| ch11 | 20676784 | 20678451 | 20677618 | PGSC0003DMG400019762 | Transferase | 22777.5 |
| ch11 | 21437878 | 21438942 | 21438410 | PGSC0003DMG400035178 | Transposon protein, CACTA, En/Spm sub-class | 738015 |
| ch11 | 20026699 | 20030240 | 20028470 | PGSC0003DMG400015910 | Ubiquinol-cytochrome c reductase iron-sulfur subunit | 671925.5 |
| ch11 | 18760292 | 18763405 | 18761849 | PGSC0003DMG400002188 | Xyloglucan endotransglucosylase/hydrolase 5 | 1938547 |
| ch11 | 18910218 | 18915114 | 18912666 | PGSC0003DMG400002184 | Zinc finger protein | 1787729 |
