# Supplementary material for: Genome‐wide association studies and genomics‐informed breeding for tuber dormancy in tetraploid potatoes
Source: Plant Genome. 2026 Feb 19;19(1):e70205. doi: 10.1002/tpg2.70205 (PMC12919731; doi:10.1002/tpg2.70205)
Supplement: Supplementary file 3 [file TPG2-19-e70205-s005.pdf]

Genome-wide association studies and genomics-informed breeding for tuber dormancy in tetraploid potatoes

Ao Jiao, Sanjeev Gautam, Jeewan Pandey, Douglas C. Scheuring, Jeffrey W. Koym, and M. Isabel Vales

Supplementary Table S7. Genomic-estimated breeding values (GEBVs), standardized scores (z), and reliability for tuber dormancy length of 292 potato genotypes evaluated in Texas (Dalhart and Springlake) for four years (2019-2022) under two storage conditions (R - room temperature - 18 ℃; C - cold storage - 4 ℃. Note: Separate sheets show different location-storage combinations.

Genotype	GEBV	r2	z_score_GEBV
NDTX081644-CAB-2W	90.6	0.77	-2.91
NDTX060700C-1W	99.1	0.78	-2.39
Tokio	99.1	0.76	-2.39
CO112-F2-2P/P	99.4	0.79	-2.37
NDTX059828-2W	99.5	0.83	-2.36
UMTX383-3Yrdspl/Y	103.7	0.77	-2.11
TX12484-4W	103.7	0.79	-2.11
ATTX98491-4Yrdspl/Y	104.3	0.76	-2.07
ATTX95490-2W	104.6	0.80	-2.05
NDTX081648CB-13W	105.2	0.77	-2.01
NDTX1244-3W/Y	106.6	0.78	-1.93
AORTX09037-5W/Y	108.3	0.77	-1.83
JTTX75/2003EH-1Yre/Y	109.3	0.81	-1.76
TX19407-5Ru	109.4	0.79	-1.76
COTX17288-1W	110.4	0.78	-1.70
ATX15325-3W/Y	110.5	0.78	-1.69
TX12484-3WZC	111.1	0.78	-1.66
COTX13029-2Ru/Y	111.5	0.75	-1.63
TX17734-1Y/Y	112.3	0.76	-1.58
COTX10118-1Wre/Y	113.8	0.80	-1.49
TX12474-1P/R	114.0	0.82	-1.48
TX19009s-2W/Y	114.1	0.77	-1.47
NDTX1246-5W/Y	114.8	0.78	-1.43
NDTX1824Y-1R	116.0	0.76	-1.35
ATX07305S-1Y/Y	117.9	0.76	-1.24
ATTX00289-5R/Y	118.1	0.76	-1.23
ATTX98448-6R/Y	118.3	0.75	-1.21
COTX16054-1W	118.4	0.78	-1.21
TX19511-5RYpinto/Y	118.5	0.78	-1.20
TX19489s-3Y/Y	118.6	0.77	-1.20
TX17797-11Y/Y	118.7	0.76	-1.19
ATTX06246-1R	119.4	0.31	-1.15
ATTX98493-2P/P	120.2	0.82	-1.10
NDTX081648CB-4W	120.9	0.80	-1.05
TX18192-2R/Y	121.1	0.79	-1.04
ATX03564-1Y/Y	121.4	0.86	-1.03
ATX03496-3Y/Y	121.5	0.86	-1.02
NDTX1759s-1R/Y	121.5	0.79	-1.02
ATX08181-5Y/Y	122.0	0.80	-0.99
COTX10138s-7Wpe/Y	122.7	0.78	-0.94
COTX17286-6W	123.4	0.78	-0.90
COTX10080-2Ru	123.4	0.86	-0.90

TX19093-1Rure	124.1	0.75	-0.86
COTX04050s-1P/P	124.2	0.80	-0.86
ATX11586-2R/Y	124.3	0.77	-0.85
TX11461-3W	124.6	0.46	-0.83
TXYG79	125.0	0.82	-0.81
ATX13134-3W/Y	125.0	0.77	-0.80
NDTX4271-5R	125.7	0.83	-0.77
NDTX050184s-1R/Y	125.7	0.41	-0.76
NDTX6773-1W	126.1	0.79	-0.74
Yukon Gold	126.2	0.82	-0.73
TX11461-2W	126.4	0.81	-0.72
NDTX4784-7R	126.7	0.81	-0.70
COTX04015-3W/Y	127.2	0.79	-0.67
TX19460-1W	127.6	0.77	-0.65
ATTX01180-1R/Y	128.0	0.81	-0.62
COTX17288-4W	128.3	0.84	-0.60
ATTX07042-3W	128.4	0.81	-0.60
ATTX961014-1AR/Y	128.4	0.85	-0.60
TX08350-12Ru	128.5	0.78	-0.59
NDTX14263BC-3W	128.5	0.78	-0.59
COTX00104-7R	128.7	0.75	-0.58
COTX17288-3W	128.7	0.84	-0.58
COTX03079-1W/W	128.7	0.82	-0.58
NDTX071217CB-1W/Y	128.8	0.82	-0.58
TX12484-2WZC	128.8	0.81	-0.57
NDTX5003-2R	128.8	0.85	-0.57
NDTX1824Y-4R	129.1	0.75	-0.55
ATTX03516-2R	129.4	0.80	-0.54
NDTX5067-2R	129.5	0.85	-0.53
COTX05249-3W/Y	129.6	0.79	-0.53
ATX15097-1Ru	129.7	0.77	-0.52
TX08385-2P/YP	129.8	0.74	-0.51
COTX10138-15Wpe/Y	129.8	0.53	-0.51
Krantz	129.8	0.75	-0.51
NDTX081451CBs-1Y/Y	129.9	0.81	-0.51
COTX08039-1P/P*	129.9	0.80	-0.51
COTX03134-1Y/W	130.1	0.77	-0.50
AORTX09037-1W/Y	130.2	0.76	-0.49
TX09414-1W	130.2	0.76	-0.49
NDTX5438-11R	130.3	0.42	-0.48
Tacna	130.3	0.74	-0.48
White LaSoda	130.6	0.80	-0.46
TX19495-9RYpinto	130.7	0.79	-0.46
COTX15111-1R	130.7	0.78	-0.45
TX09403-15W	130.9	0.77	-0.45
ATX11684f-2W/Y	131.3	0.70	-0.42
NDTX081618-1P/P	131.3	0.73	-0.42
BTX2103-1R/Y	131.3	0.81	-0.42
TX09403-21W	131.4	0.76	-0.41
AOTX95309-2W*	131.4	0.88	-0.41

AOTX98152-3Ru	131.5	0.88	-0.41
TX13590-9Ru	131.5	0.88	-0.41
Sierra RoseTM	131.5	0.88	-0.41
ATTX98462s-3R/Y	131.5	0.88	-0.41
ATTX961014-1Br/Y	131.6	0.87	-0.40
TXA549-1Ru	131.6	0.88	-0.40
COTX02293-4R	131.7	0.78	-0.40
COTX00104-6R	131.8	0.82	-0.39
PORTX03PG25-2R/R	131.8	0.81	-0.39
ATTX98518-5P/Y	132.2	0.79	-0.37
ATTX98493-1AR	132.2	0.82	-0.37
COTX05211-5R	132.4	0.85	-0.35
COTX05211-7R	132.5	0.80	-0.35
NDTX7590-3R	132.5	0.80	-0.35
COTX05211-4R	132.5	0.85	-0.35
COTX17123-2Ru	132.6	0.77	-0.34
NDTX091908AB-2W	133.1	0.82	-0.31
NDTX092237C-2P/WP	133.1	0.79	-0.31
COTX01403-4R/Y	133.2	0.40	-0.30
NDTX091886-3P/P	133.4	0.80	-0.29
AOTX91861-4R	133.5	0.81	-0.29
ATTX96746-1R*	133.5	0.80	-0.29
COTX08258-6Ru	133.8	0.77	-0.27
TX14611-1R	133.8	0.75	-0.27
COTX09022-3RuRE/Y	133.8	0.75	-0.26
ATTX98514-1R/Y	134.0	0.78	-0.26
NDTX14156-3P/WP	134.0	0.78	-0.25
Sierra GoldTM	134.2	0.87	-0.24
ATX87184-2Ru	134.2	0.81	-0.24
COTX12235-2W	134.4	0.76	-0.23
BTX2332-1R	134.5	0.81	-0.22
COTX08121-4Ru	134.6	0.82	-0.22
NDTX081648CB-1W	134.7	0.42	-0.21
NDTX092238Cs-1P/W	134.7	0.80	-0.21
COTX08063-2Ru	134.8	0.81	-0.21
ATTX98465-1R/Y	134.9	0.77	-0.20
ATTX98510-1R/Y	135.2	0.86	-0.18
Red LaSoda	135.4	0.76	-0.17
NDTX4756-1R/Y	135.4	0.86	-0.17
ATTX05186-2R	135.5	0.83	-0.16
NDTX059775-1W	135.6	0.81	-0.16
COTX10138-19P/Y	135.8	0.81	-0.15
TX19495-16RYpinto	135.8	0.55	-0.14
ATX05186-1R	135.9	0.83	-0.14
Rio Rojo	135.9	0.76	-0.14
NDTX059886S-1Y/Y	135.9	0.47	-0.14
COTX08322-10Ru	135.9	0.79	-0.14
COTX18206-1Ru	136.0	0.77	-0.13
NDTX91068-11R	136.2	0.83	-0.12
NDTX4828-2R	136.3	0.77	-0.11

ATTX00289-4W	136.3	0.83	-0.11
NDTX14247CAB-1W	136.7	0.84	-0.09
TX1617-1W/Y	136.8	0.77	-0.09
COTX90046-1W	136.8	0.41	-0.09
MWTX548-2Ru	136.8	0.88	-0.08
TX11454-9Ru/Y	136.8	0.81	-0.08
NDTX14247CAB-2W	136.9	0.84	-0.08
TX18042-1Ru	136.9	0.78	-0.08
MWTX2609-2Ru	137.0	0.88	-0.07
NDTX1753Y-1R	137.3	0.82	-0.05
NDTX1482YB-1W	137.4	0.76	-0.05
TX1673-1W/Y	137.4	0.84	-0.05
COTX10118-4Wpe/Y	137.5	0.78	-0.04
AOR07781-2	137.7	0.37	-0.03
ATTX88654-2P/Y	137.8	0.80	-0.02
MWTX2609-4Ru	137.8	0.88	-0.02
COTX08121-1Ru	138.3	0.79	0.01
COTX02172-1R	138.4	0.81	0.01
BTX1544-2W/Y	138.4	0.80	0.02
NDTX059759-3R/Y Pinto	138.6	0.83	0.02
COTX87601-2Ru	138.6	0.79	0.03
NDTX050169-1R	139.0	0.84	0.05
NDTX059761-1R/R	139.0	0.83	0.05
COTX15083-1R	139.3	0.75	0.07
NDTX071109C-1W	139.4	0.81	0.07
ATTX98444s-16R/Y	139.5	0.81	0.08
TX17805-1RWpinto/R	139.6	0.80	0.09
COTX15271-1Ru	139.8	0.77	0.10
AOTX95309-1W	139.8	0.76	0.10
ATX85404-8W	139.8	0.76	0.10
TX09396-1W	139.8	0.82	0.10
TX6-1216-1Ru	140.7	0.80	0.15
ATX16089-4Ru	140.8	0.78	0.16
TX17805-8R/R	140.9	0.80	0.17
Chieftain	141.0	0.78	0.17
ATX05202s-3W/Y	141.1	0.81	0.18
NDTX071258B-1R	141.2	0.37	0.19
Atlantic	141.3	0.81	0.19
TX19508-5RYpinto/Y	141.5	0.78	0.20
AOTX93483-1R	141.6	0.81	0.21
PTTX05PG07-1W	141.9	0.79	0.23
Stampede Russet	141.9	0.80	0.23
NDTX8773-4Ru	142.1	0.80	0.24
TX17742-5YPpinto/YP	142.2	0.78	0.25
ATX11549-1P/Y	142.3	0.77	0.25
COTX05095-2Ru/Y	142.4	0.82	0.26
TX17763-2Y/Y	142.7	0.77	0.28
AOTX95265-2Ru	142.8	0.89	0.29
ATTX88481-1P/W	143.0	0.86	0.30
COTX90046-5W	143.1	0.77	0.30

ATTX10007-1Ru	143.4	0.78	0.32
ATTX98466-5R/WR	143.5	0.85	0.33
AOTX95265-2ARu	143.7	0.46	0.34
COTX10073s-1W	143.7	0.63	0.34
ATTX961014-1R/Y Chimera	143.9	0.84	0.35
COTX05261-1R/Y	144.2	0.80	0.37
TX17805-10RYpinto/Y	144.3	0.80	0.37
TX17846-1W	144.4	0.76	0.38
AOTX96208-1Ru	144.4	0.92	0.38
NDTX4930-5W	144.7	0.80	0.40
COTX04193s-2R/Y	145.0	0.79	0.42
AOTX95265-4Ru	145.4	0.94	0.44
ATX84706-2Ru	145.4	0.80	0.44
ATTX10333-1W/Y	145.5	0.77	0.45
Russet Norkotah 102	145.5	0.95	0.45
TX1475-3W	145.5	0.81	0.45
ATX99013-1Ru	145.5	0.95	0.45
TXNS106	145.6	0.95	0.45
TXNS249	145.6	0.95	0.45
Russet Norkotah 278	145.7	0.95	0.46
Russet Norkotah	145.7	0.95	0.46
COTX04303-3Ru/Y*	145.7	0.95	0.46
Russet Norkotah 112	145.7	0.95	0.46
AOTX98137-1Ru	145.7	0.95	0.46
AOTX96084-1Ru	145.7	0.90	0.46
Russet Norkotah 296	145.7	0.95	0.46
AOTX98096-1Ru*	145.7	0.95	0.46
AOTX95295-3Ru	145.7	0.95	0.46
TXNS118	145.8	0.95	0.46
ATX15120-1Ru	145.8	0.75	0.47
AOTX96075-1Ru	145.8	0.95	0.47
AOTX95265-1Ru	145.8	0.95	0.47
Russet Norkotah 223	145.8	0.95	0.47
AOTX95265-3Ru	145.9	0.95	0.47
TX05249-3W	145.9	0.77	0.47
ATTX98500-2P/Y	146.1	0.44	0.48
COTX17304-1Ru	147.0	0.75	0.54
ATTX99325-1P	147.4	0.86	0.56
ATX06264s-4R/Y	147.5	0.41	0.57
TX17805-12R/R	147.8	0.79	0.59
TX17788f-4R/R	148.2	0.78	0.61
NDTX1791-1Ru	148.3	0.78	0.62
TX10437-9Pyspl/Y	148.6	0.82	0.63
NDTX14362AB-1W	148.6	0.79	0.64
ATTX01178-1R	148.7	0.81	0.64
AOTX96216-2Ru	148.9	0.84	0.65
ATX9117-1Ru	149.0	0.80	0.66
AOTX96216-1Ru	149.1	0.85	0.67
BTX1749-1W/Y	149.2	0.32	0.67
ATX84378-6Ru	150.8	0.85	0.77

COTX94216-1R	152.5	0.56	0.87
TX03196-1W	153.4	0.79	0.93
TX19495-17R/Y-R	154.2	0.79	0.98
ATX02263-1R/Y	154.2	0.76	0.98
AOTX03187-1Ru	154.3	0.86	0.99
COTX03187-1W	154.3	0.86	0.99
COTX10012-1Wrdspl/Y	154.9	0.80	1.02
ATX9312-1Ru	155.3	0.82	1.04
COTX89044-1Ru	155.9	0.81	1.09
TX19411-4W	156.2	0.79	1.10
NDTX12248Y-1R	156.4	0.77	1.12
ATTX05175s-1R/Y	156.4	0.74	1.12
NDTX731-1R	156.9	0.34	1.14
ATTX98500-3P/Y	157.1	0.84	1.15
Vanguard Russet	157.2	0.83	1.16
COTX09089-1Ru	157.3	0.81	1.17
TX17742-23P/P	157.8	0.79	1.20
NDTX14156-2RWpinto/Y	158.1	0.48	1.22
COTX09052-2Ru	158.3	0.86	1.23
NDTX14156A-3RWpinto/Y	158.3	0.48	1.23
COTX09052-1Ru	158.4	0.86	1.24
ATTX98453-3R	158.7	0.82	1.25
COTX16005-1Ru	158.7	0.77	1.26
TX17755-2Psplash	159.5	0.78	1.30
ATTX98453-6R	160.5	0.80	1.36
COTX94218-1R	160.5	0.81	1.37
ATTX98468-5Ru/Y*	160.8	0.82	1.38
AOTX05043-1Ru	162.5	0.81	1.49
ATTX00289-6Y/Y	163.2	0.82	1.53
ATTX98453-11Br	164.2	0.82	1.59
COTX08365F-3P/P	164.3	0.82	1.60
ATX9130-1Ru	164.6	0.79	1.62
Banana	164.8	0.79	1.63
NDTX092237C-2P/W	165.0	0.82	1.64
ATX97147-4Ru	165.1	0.81	1.64
Russet Burbank	169.9	0.83	1.94
TX17742-19R/R	169.9	0.78	1.94
AOTX02136-1Ru	171.2	0.82	2.02
Reveille Russet	172.5	0.85	2.10
AOTX98202-1Ru	172.6	0.81	2.10
COTX17115f-3R/R	174.9	0.77	2.24
COTX17016f-2P/P	175.5	0.77	2.28
COTX13215-2Ru	175.7	0.79	2.29
AOTX97213-1Ru	176.6	0.82	2.35
ATX9332-8Ru	177.1	0.78	2.38
TX05249-10W	178.7	0.76	2.48
COTX10226-1Wpe/Y	179.3	0.78	2.52
TX05249-11W	180.1	0.83	2.56
ATTX10265-4R/Y	188.3	0.80	3.07
ATX9202-3Ru	195.3	0.82	3.49

Genotype	GEBV	r2	z_score_GEBV
COTX10138-15Wpe/Y	58.0	0.88	-1.69
NDTX5003-2R	58.5	0.91	-1.65
NDTX5067-2R	58.7	0.91	-1.64
ATTX98491-4Yrdspl/Y	59.9	0.91	-1.57
UMTX383-3Yrdspl/Y	59.9	0.91	-1.57
TX09403-21W	61.0	0.91	-1.50
TX09403-15W	61.4	0.91	-1.48
NDTX4271-5R	62.0	0.91	-1.44
COTX01403-4R/Y	62.2	0.87	-1.43
AOR07781-2	62.2	0.88	-1.43
TX12484-4W	62.5	0.88	-1.41
NDTX4784-7R	62.6	0.91	-1.40
NDTX059828-2W	63.3	0.89	-1.36
COTX10138s-7Wpe/Y	63.5	0.88	-1.34
COTX13029-2Ru/Y	63.8	0.81	-1.33
COTX10118-4Wpe/Y	63.8	0.88	-1.33
NDTX060700C-1W	63.9	0.88	-1.32
ATTX00289-5R/Y	64.1	0.91	-1.31
ATTX98448-6R/Y	64.2	0.91	-1.30
ATTX98465-1R/Y	64.4	0.88	-1.29
ATX06264s-4R/Y	64.8	0.86	-1.27
ATX15097-1Ru	64.9	0.83	-1.26
TX18042-1Ru	65.1	0.83	-1.24
NDTX071258B-1R	65.4	0.88	-1.22
COTX02293-4R	65.5	0.88	-1.22
ATTX88481-1P/W	65.7	0.91	-1.21
ATTX99325-1P	65.9	0.90	-1.19
COTX17288-1W	66.0	0.83	-1.19
ATTX98500-2P/Y	66.3	0.87	-1.17
NDTX4756-1R/Y	66.6	0.91	-1.15
Sierra GoldTM	66.7	0.91	-1.15
ATTX98510-1R/Y	66.7	0.91	-1.14
NDTX6773-1W	66.8	0.88	-1.14
TX12484-3WZC	66.9	0.83	-1.13
COTX10118-1Wre/Y	67.0	0.87	-1.13
Tokio	67.0	0.87	-1.13
AORTX09037-5W/Y	67.3	0.83	-1.11
TX19460-1W	67.4	0.83	-1.10
ATTX01178-1R	67.8	0.88	-1.08
TX12484-2WZC	67.8	0.86	-1.07
COTX03134-1Y/W	68.0	0.88	-1.06
ATX15325-3W/Y	68.1	0.84	-1.06
AOTX95309-2W*	68.3	0.93	-1.05
ATTX961014-1Br/Y	68.3	0.93	-1.05
ATTX98462s-3R/Y	68.4	0.93	-1.04
Sierra RoseTM	68.4	0.93	-1.04
TX19407-5Ru	68.6	0.84	-1.03
COTX05261-1R/Y	68.6	0.87	-1.02
COTX04193s-2R/Y	68.7	0.87	-1.02

ATTX96746-1R*	68.9	0.87	-1.01
ATTX95490-2W	69.1	0.87	-0.99
COTX05211-7R	69.2	0.87	-0.99
TX17797-11Y/Y	69.2	0.82	-0.99
NDTX081644-CAB-2W	69.3	0.87	-0.98
NDTX081451CBs-1Y/Y	69.4	0.88	-0.98
PORTX03PG25-2R/R	69.6	0.88	-0.97
NDTX059886S-1Y/Y	70.1	0.58	-0.93
BTX1749-1W/Y	70.4	0.87	-0.91
COTX04015-3W/Y	70.5	0.87	-0.91
ATTX98493-2P/P	70.7	0.88	-0.90
COTX08039-1P/P*	70.9	0.88	-0.88
ATTX07042-3W	70.9	0.87	-0.88
COTX90046-1W	70.9	0.87	-0.88
NDTX081648CB-13W	71.1	0.87	-0.87
COTX10226-1Wpe/Y	71.2	0.88	-0.87
NDTX1824Y-1R	71.3	0.82	-0.86
NDTX050184s-1R/Y	71.4	0.87	-0.85
TX19511-5RYpinto/Y	71.6	0.83	-0.84
COTX05249-3W/Y	71.6	0.87	-0.84
COTX05211-5R	71.7	0.91	-0.84
COTX05211-4R	71.8	0.91	-0.83
COTX16005-1Ru	72.2	0.83	-0.80
TX11461-3W	72.3	0.88	-0.80
Tacna	73.3	0.85	-0.74
COTX90046-5W	73.4	0.88	-0.73
ATTX961014-1AR/Y	73.6	0.90	-0.72
ATX03564-1Y/Y	73.6	0.91	-0.72
ATX03496-3Y/Y	73.6	0.91	-0.71
COTX12235-2W	73.7	0.82	-0.71
COTX02172-1R	74.1	0.88	-0.69
TX19411-4W	74.2	0.84	-0.68
TXYG79	74.2	0.89	-0.68
AORTX09037-1W/Y	74.5	0.88	-0.66
NDTX5438-11R	74.6	0.53	-0.66
ATTX98514-1R/Y	74.7	0.86	-0.65
ATX13134-3W/Y	74.7	0.83	-0.65
ATTX01180-1R/Y	74.8	0.88	-0.64
TX08385-2P/YP	74.9	0.86	-0.64
ATTX05175s-1R/Y	75.1	0.87	-0.62
NDTX4930-5W	76.0	0.87	-0.57
TX11461-2W	76.0	0.88	-0.56
NDTX7590-3R	76.1	0.87	-0.56
NDTX081618-1P/P	76.3	0.85	-0.55
COTX16054-1W	76.4	0.83	-0.54
ATX9117-1Ru	76.8	0.87	-0.52
TX09396-1W	76.9	0.88	-0.51
NDTX91068-11R	77.0	0.89	-0.51
ATTX98518-5P/Y	77.0	0.87	-0.50
Yukon Gold	77.1	0.87	-0.50

COTX08258-6Ru	77.1	0.88	-0.50
ATTX98466-5R/WR	77.2	0.91	-0.49
ATX11684f-2W/Y	77.3	0.82	-0.49
ATTX00289-6Y/Y	77.3	0.88	-0.49
NDTX14263BC-3W	77.3	0.83	-0.49
NDTX1244-3W/Y	77.3	0.84	-0.48
ATTX98493-1AR	77.3	0.88	-0.48
ATTX961014-1R/Y Chimera	77.4	0.91	-0.48
NDTX1824Y-4R	77.4	0.81	-0.48
COTX03079-1W/W	77.6	0.88	-0.47
COTX17286-6W	77.6	0.83	-0.46
ATTX98500-3P/Y	78.0	0.89	-0.44
ATTX98444s-16R/Y	78.1	0.88	-0.44
NDTX092238Cs-1P/W	78.1	0.87	-0.44
TX1617-1W/Y	78.1	0.86	-0.44
NDTX1759s-1R/Y	78.3	0.84	-0.42
AOTX93483-1R	78.4	0.88	-0.42
Krantz	78.4	0.86	-0.41
TX17846-1W	78.6	0.82	-0.41
ATTX88654-2P/Y	78.6	0.87	-0.40
Atlantic	78.6	0.91	-0.40
NDTX1791-1Ru	78.9	0.83	-0.39
TX19009s-2W/Y	78.9	0.83	-0.39
NDTX14362AB-1W	78.9	0.84	-0.38
NDTX1246-5W/Y	78.9	0.83	-0.38
COTX00104-7R	78.9	0.87	-0.38
Banana	79.1	0.84	-0.37
BTX1544-2W/Y	79.1	0.87	-0.37
COTX00104-6R	79.2	0.88	-0.37
NDTX092237C-2P/WP	79.5	0.84	-0.35
JTTX75/2003EH-1Yre/Y	79.5	0.88	-0.35
COTX05095-2Ru/Y	79.6	0.88	-0.34
COTX08063-2Ru	79.8	0.87	-0.33
COTX15111-1R	80.0	0.83	-0.32
COTX09022-3RuRE/Y	80.2	0.87	-0.31
TX05249-3W	80.3	0.88	-0.30
TX19093-1Rure	80.5	0.81	-0.29
NDTX091908AB-2W	80.5	0.88	-0.29
ATTX05186-2R	80.5	0.89	-0.29
COTX10138-19P/Y	80.5	0.86	-0.28
AOTX95309-1W	80.6	0.90	-0.28
TX14611-1R	80.8	0.87	-0.26
TX18192-2R/Y	80.9	0.84	-0.26
ATX85404-8W	81.0	0.90	-0.26
ATX11586-2R/Y	81.1	0.83	-0.25
ATTX00289-4W	81.2	0.90	-0.24
ATTX03516-2R	81.4	0.87	-0.23
COTX04050s-1P/P	81.5	0.87	-0.22
COTX17288-4W	81.5	0.88	-0.22
COTX17288-3W	81.5	0.88	-0.22

TX03196-1W	81.8	0.86	-0.20
ATX05186-1R	82.3	0.90	-0.17
Chieftain	82.3	0.83	-0.17
TX19489s-3Y/Y	82.3	0.82	-0.17
ATX16089-4Ru	82.5	0.83	-0.16
BTX2103-1R/Y	82.8	0.88	-0.14
TX1673-1W/Y	82.8	0.90	-0.14
COTX94216-1R	83.0	0.66	-0.13
COTX10012-1Wrdspl/Y	83.0	0.87	-0.13
COTX15083-1R	83.0	0.81	-0.13
COTX94218-1R	83.4	0.87	-0.10
ATTX10333-1W/Y	83.6	0.83	-0.09
TX17734-1Y/Y	83.8	0.82	-0.08
White LaSoda	84.0	0.87	-0.07
NDTX071109C-1W	84.0	0.88	-0.07
NDTX14247CAB-1W	84.1	0.88	-0.06
NDTX14247CAB-2W	84.1	0.88	-0.06
ATX02263-1R/Y	84.2	0.87	-0.05
COTX15271-1Ru	84.5	0.83	-0.04
TX09414-1W	84.7	0.87	-0.03
NDTX071217CB-1W/Y	84.9	0.88	-0.01
NDTX1753Y-1R	84.9	0.86	-0.01
COTX17304-1Ru	85.3	0.81	0.02
TX17742-5YPpinto/YP	85.4	0.83	0.02
Stampede Russet	85.8	0.87	0.04
NDTX081648CB-1W	86.1	0.50	0.06
ATTX10007-1Ru	87.1	0.84	0.12
ATX07305S-1Y/Y	87.7	0.87	0.16
TX6-1216-1Ru	88.0	0.86	0.18
COTX08322-10Ru	88.5	0.89	0.22
BTX2332-1R	88.7	0.88	0.22
ATX9130-1Ru	88.7	0.88	0.23
TX10437-9Pyspl/Y	89.2	0.87	0.25
ATTX98468-5Ru/Y*	89.6	0.88	0.28
ATTX98453-11Br	89.6	0.88	0.28
NDTX12248Y-1R	89.9	0.82	0.30
AOTX95265-2ARu	90.1	0.89	0.31
NDTX731-1R	90.1	0.87	0.31
Red LaSoda	90.5	0.84	0.34
COTX03187-1W	90.5	0.90	0.34
NDTX14156-3P/WP	90.5	0.83	0.34
MWTX548-2Ru	90.7	0.92	0.35
MWTX2609-2Ru	90.8	0.92	0.36
AOTX96216-1Ru	91.1	0.89	0.37
ATX05202s-3W/Y	91.3	0.88	0.39
Rio Rojo	91.3	0.87	0.39
AOTX03187-1Ru	91.4	0.90	0.39
ATX15120-1Ru	91.5	0.81	0.40
MWTX2609-4Ru	91.6	0.92	0.40
AOTX91861-4R	91.6	0.87	0.41

TX17788f-4R/R	91.8	0.83	0.42
COTX09089-1Ru	91.8	0.88	0.42
AOTX96216-2Ru	92.2	0.89	0.44
NDTX4828-2R	92.5	0.88	0.46
NDTX050169-1R	92.7	0.89	0.47
COTX08121-4Ru	92.8	0.89	0.48
ATX84378-6Ru	92.9	0.89	0.49
ATX08181-5Y/Y	93.1	0.87	0.50
COTX10073s-1W	93.1	0.74	0.50
NDTX081648CB-4W	93.2	0.87	0.51
COTX87601-2Ru	93.3	0.87	0.51
NDTX1482YB-1W	93.5	0.82	0.52
ATTX98453-6R	93.5	0.87	0.53
COTX10080-2Ru	93.7	0.90	0.54
NDTX14156-2RWpinto/Y	93.7	0.52	0.54
NDTX14156A-3RWpinto/Y	93.8	0.52	0.54
NDTX091886-3P/P	94.0	0.87	0.55
TX11454-9Ru/Y	94.0	0.88	0.56
TX19508-5RYpinto/Y	94.3	0.83	0.58
NDTX059759-3R/Y Pinto	94.4	0.89	0.58
TX17763-2Y/Y	95.0	0.82	0.62
PTTX05PG07-1W	95.1	0.88	0.62
TX08350-12Ru	95.4	0.88	0.64
AOTX02136-1Ru	95.8	0.88	0.67
ATTX10265-4R/Y	96.0	0.87	0.68
COTX89044-1Ru	96.5	0.87	0.71
NDTX059775-1W	96.5	0.88	0.71
TX1475-3W	96.9	0.88	0.73
ATX87184-2Ru	97.1	0.88	0.75
NDTX059761-1R/R	97.6	0.89	0.78
TX19495-16RYpinto	97.7	0.59	0.79
TX05249-10W	97.8	0.87	0.79
TX17755-2Psplash	97.9	0.83	0.80
COTX18206-1Ru	98.0	0.82	0.80
AOTX97213-1Ru	98.8	0.87	0.85
ATX11549-1P/Y	98.9	0.82	0.86
AOTX98202-1Ru	99.8	0.87	0.92
COTX08121-1Ru	101.2	0.88	1.00
ATTX98453-3R	103.0	0.88	1.11
TX17805-10RYpinto/Y	103.1	0.85	1.12
TX17805-8R/R	103.3	0.84	1.13
NDTX8773-4Ru	104.0	0.87	1.18
AOTX95265-2Ru	104.5	0.91	1.21
AOTX05043-1Ru	104.5	0.88	1.21
TX17805-1RWpinto/R	104.5	0.85	1.21
TX19495-9RYpinto	105.5	0.84	1.27
TX17742-23P/P	105.9	0.84	1.30
COTX17123-2Ru	106.7	0.83	1.35
AOTX96208-1Ru	107.0	0.94	1.36
COTX09052-1Ru	107.1	0.91	1.37

AOTX95265-4Ru	107.1	0.95	1.37
COTX09052-2Ru	107.1	0.91	1.37
AOTX96084-1Ru	107.2	0.92	1.38
Russet Norkotah 223	107.6	0.96	1.40
AOTX95265-1Ru	107.6	0.96	1.41
Russet Norkotah	107.7	0.96	1.41
COTX04303-3Ru/Y*	107.7	0.96	1.41
AOTX98137-1Ru	107.7	0.96	1.41
AOTX95295-3Ru	107.7	0.96	1.41
TXNS118	107.7	0.96	1.41
Russet Norkotah 278	107.7	0.96	1.41
TXNS106	107.8	0.96	1.41
TXNS249	107.8	0.96	1.41
ATX99013-1Ru	107.8	0.96	1.41
AOTX95265-3Ru	107.8	0.96	1.41
Russet Norkotah 102	107.9	0.96	1.42
Russet Norkotah 296	107.9	0.96	1.42
AOTX96075-1Ru	107.9	0.96	1.42
Russet Norkotah 112	107.9	0.96	1.42
TX13590-9Ru	107.9	0.92	1.42
AOTX98096-1Ru*	108.0	0.96	1.43
TX12474-1P/R	108.1	0.89	1.43
TXA549-1Ru	108.1	0.92	1.44
ATX84706-2Ru	108.4	0.87	1.45
AOTX98152-3Ru	108.5	0.92	1.46
TX19495-17R/Y-R	108.9	0.84	1.49
ATX9332-8Ru	109.8	0.87	1.54
COTX17115f-3R/R	110.2	0.82	1.56
ATX9312-1Ru	110.4	0.88	1.58
TX05249-11W	110.8	0.89	1.60
TX17742-19R/R	111.1	0.83	1.62
COTX13215-2Ru	112.0	0.86	1.68
ATX97147-4Ru	114.1	0.88	1.81
COTX08365F-3P/P	118.4	0.88	2.08
Russet Burbank	118.7	0.90	2.09
ATX9202-3Ru	119.3	0.88	2.13
CO112-F2-2P/P	120.7	0.85	2.22
ATTX06246-1R	124.1	0.86	2.43
Vanguard Russet	124.7	0.89	2.47
NDTX092237C-2P/W	126.5	0.87	2.58
Reveille Russet	127.8	0.89	2.66
COTX17016f-2P/P	134.7	0.82	3.09
TX17805-12R/R	145.3	0.84	3.75

Genotype	GEBV	r2	z_score_GEBV
Krantz	94.5	0.76	-2.06
TX19407-5Ru	96.8	0.70	-1.84
COTX08322-10Ru	97.9	0.78	-1.74
TX08350-12Ru	99.0	0.79	-1.64
AOTX95265-2ARu	99.2	0.78	-1.62
TX19009s-2W/Y	99.8	0.67	-1.57
COTX09022-3RuRE/Y	99.9	0.76	-1.55
JTTX75/2003EH-1Yre/Y	100.0	0.78	-1.54
COTX10080-2Ru	100.7	0.81	-1.47
TX6-1216-1Ru	101.5	0.77	-1.40
AOTX98152-3Ru	101.6	0.85	-1.39
TX13590-9Ru	101.6	0.85	-1.39
TXA549-1Ru	101.7	0.85	-1.39
ATX15325-3W/Y	102.2	0.68	-1.34
Tokio	102.4	0.75	-1.32
NDTX081648CB-13W	102.4	0.77	-1.31
AOR07781-2	102.6	0.78	-1.30
AORTX09037-5W/Y	102.9	0.67	-1.27
AOTX95265-4Ru	103.4	0.91	-1.22
UMTX383-3Yrdspl/Y	103.5	0.83	-1.21
ATX99013-1Ru	103.5	0.92	-1.21
AOTX95265-1Ru	103.6	0.92	-1.21
AOTX98137-1Ru	103.6	0.92	-1.20
AOTX95265-3Ru	103.6	0.92	-1.20
TXNS118	103.6	0.92	-1.20
TXNS249	103.6	0.92	-1.20
Russet Norkotah	103.6	0.92	-1.20
TXNS106	103.6	0.92	-1.20
Russet Norkotah 278	103.6	0.92	-1.20
COTX04303-3Ru/Y*	103.6	0.92	-1.20
Russet Norkotah 223	103.7	0.92	-1.20
Russet Norkotah 296	103.7	0.92	-1.20
AOTX95295-3Ru	103.7	0.92	-1.19
AOTX96075-1Ru	103.7	0.92	-1.19
Russet Norkotah 112	103.7	0.92	-1.19
AOTX98096-1Ru*	103.7	0.92	-1.19
Russet Norkotah 102	103.8	0.92	-1.19
ATTX98491-4Yrdspl/Y	103.8	0.83	-1.19
ATTX98465-1R/Y	104.0	0.78	-1.17
COTX16054-1W	104.1	0.67	-1.16
BTX1544-2W/Y	104.4	0.76	-1.13
ATX87184-2Ru	104.6	0.78	-1.11
ATX13134-3W/Y	104.6	0.66	-1.11
TX12484-3WZC	104.7	0.69	-1.10
AOTX96208-1Ru	104.7	0.90	-1.10
TX11454-9Ru/Y	104.7	0.69	-1.10
ATX9312-1Ru	104.8	0.72	-1.09
Sierra GoldTM	105.2	0.82	-1.05
AOTX95265-2Ru	105.2	0.86	-1.05

NDTX050184s-1R/Y	105.5	0.77	-1.03
AOTX96084-1Ru	105.6	0.87	-1.01
COTX17288-4W	105.9	0.76	-0.98
COTX17288-3W	106.0	0.76	-0.98
COTX08063-2Ru	106.1	0.77	-0.97
TX11461-3W	106.3	0.76	-0.95
COTX04015-3W/Y	106.3	0.75	-0.95
ATTX98448-6R/Y	106.4	0.82	-0.94
ATTX00289-5R/Y	106.4	0.82	-0.93
TX12484-2WZC	106.4	0.73	-0.93
ATX85404-8W	106.5	0.81	-0.93
AOTX95309-1W	106.5	0.81	-0.92
NDTX059828-2W	106.6	0.78	-0.92
ATTX00289-4W	106.6	0.81	-0.91
ATTX07042-3W	106.7	0.77	-0.91
ATX08181-5Y/Y	107.0	0.77	-0.88
ATTX95490-2W	107.0	0.76	-0.88
COTX08258-6Ru	107.1	0.78	-0.87
TX18042-1Ru	107.1	0.67	-0.87
TX1673-1W/Y	107.3	0.81	-0.85
ATX03496-3Y/Y	107.3	0.83	-0.85
ATX03564-1Y/Y	107.4	0.83	-0.85
COTX01403-4R/Y	107.4	0.78	-0.84
ATTX98514-1R/Y	107.6	0.75	-0.82
NDTX14247CAB-1W	107.8	0.74	-0.81
TX19093-1Rure	107.9	0.64	-0.80
ATTX06246-1R	107.9	0.74	-0.80
NDTX14247CAB-2W	107.9	0.74	-0.79
ATTX961014-1AR/Y	108.0	0.83	-0.78
TX17797-11Y/Y	108.3	0.60	-0.76
ATX9332-8Ru	108.4	0.77	-0.75
TX09414-1W	108.5	0.77	-0.74
COTX13029-2Ru/Y	108.5	0.64	-0.74
COTX90046-5W	108.6	0.78	-0.73
NDTX8773-4Ru	108.7	0.77	-0.72
Tacna	108.9	0.67	-0.70
TX11461-2W	109.0	0.78	-0.69
TX12484-4W	109.1	0.79	-0.68
AOTX95309-2W*	109.3	0.85	-0.66
ATTX961014-1Br/Y	109.4	0.85	-0.65
COTX12235-2W	109.4	0.65	-0.65
Sierra RoseTM	109.4	0.85	-0.65
BTX1749-1W/Y	109.5	0.77	-0.65
ATTX98462s-3R/Y	109.5	0.85	-0.64
COTX08121-1Ru	109.5	0.79	-0.64
COTX87601-2Ru	109.6	0.76	-0.64
TX19460-1W	110.1	0.66	-0.59
ATX11684f-2W/Y	110.1	0.65	-0.59
ATX84378-6Ru	110.4	0.81	-0.56
COTX10138s-7Wpe/Y	110.6	0.80	-0.54

COTX10118-4Wpe/Y	110.8	0.79	-0.52
AOTX96216-2Ru	110.8	0.80	-0.52
COTX03079-1W/W	111.3	0.79	-0.48
TX19411-4W	111.5	0.69	-0.45
MWTX548-2Ru	111.6	0.83	-0.44
MWTX2609-4Ru	111.8	0.83	-0.43
MWTX2609-2Ru	111.9	0.83	-0.42
AOTX96216-1Ru	111.9	0.81	-0.42
ATTX98510-1R/Y	111.9	0.82	-0.41
COTX03134-1Y/W	112.0	0.70	-0.41
TXYG79	112.0	0.80	-0.40
NDTX4756-1R/Y	112.0	0.82	-0.40
COTX00104-6R	112.1	0.77	-0.40
NDTX14263BC-3W	112.1	0.61	-0.40
COTX10138-15Wpe/Y	112.1	0.79	-0.40
NDTX1244-3W/Y	112.1	0.69	-0.39
ATX15097-1Ru	112.2	0.67	-0.39
NDTX081644-CAB-2W	112.2	0.77	-0.39
ATX05202s-3W/Y	112.2	0.78	-0.39
TX17734-1Y/Y	112.3	0.66	-0.38
NDTX081451CBs-1Y/Y	112.6	0.77	-0.36
COTX02293-4R	112.6	0.79	-0.35
COTX05095-2Ru/Y	112.6	0.79	-0.35
NDTX1791-1Ru	112.6	0.68	-0.35
COTX17288-1W	112.7	0.68	-0.34
TX09403-21W	112.8	0.80	-0.34
NDTX6773-1W	112.8	0.79	-0.33
TX09403-15W	112.9	0.80	-0.32
COTX05249-3W/Y	113.1	0.74	-0.30
ATX9117-1Ru	113.3	0.76	-0.29
COTX17304-1Ru	113.4	0.64	-0.27
ATX06264s-4R/Y	113.5	0.75	-0.27
CO112-F2-2P/P	113.5	0.75	-0.26
ATX9130-1Ru	113.6	0.79	-0.25
TX17846-1W	113.7	0.65	-0.25
COTX10012-1Wrdspl/Y	113.8	0.77	-0.24
NDTX1482YB-1W	114.2	0.65	-0.20
ATX07305S-1Y/Y	114.3	0.77	-0.19
COTX17123-2Ru	114.4	0.67	-0.18
NDTX14362AB-1W	114.4	0.69	-0.18
NDTX071258B-1R	114.6	0.78	-0.16
AORTX09037-1W/Y	114.9	0.77	-0.14
ATTX10333-1W/Y	114.9	0.67	-0.13
NDTX4828-2R	114.9	0.78	-0.13
COTX10118-1Wre/Y	114.9	0.77	-0.13
NDTX060700C-1W	115.1	0.78	-0.11
COTX02172-1R	115.3	0.78	-0.10
NDTX059775-1W	115.4	0.76	-0.09
TX19511-5RYpinto/Y	115.4	0.63	-0.08
COTX18206-1Ru	115.5	0.66	-0.07

ATTX10007-1Ru	115.7	0.68	-0.06
TX1617-1W/Y	115.7	0.77	-0.05
COTX15271-1Ru	115.8	0.66	-0.05
Yukon Gold	115.8	0.76	-0.05
COTX89044-1Ru	115.8	0.77	-0.04
ATTX98444s-16R/Y	116.0	0.78	-0.03
ATTX96746-1R*	116.0	0.77	-0.03
NDTX059886S-1Y/Y	116.1	0.42	-0.02
NDTX4930-5W	116.2	0.77	-0.01
Banana	116.4	0.71	0.01
Stampede Russet	116.4	0.77	0.01
Vanguard Russet	116.5	0.80	0.02
ATTX03516-2R	116.6	0.76	0.03
NDTX7590-3R	116.6	0.77	0.03
ATTX98493-1AR	116.8	0.79	0.04
COTX17286-6W	116.9	0.68	0.06
NDTX1246-5W/Y	117.3	0.68	0.09
COTX04050s-1P/P	117.3	0.76	0.09
ATTX98468-5Ru/Y*	117.4	0.77	0.10
COTX90046-1W	117.4	0.76	0.10
NDTX059761-1R/R	117.4	0.79	0.11
NDTX081648CB-4W	117.5	0.76	0.11
COTX05261-1R/Y	117.7	0.77	0.13
COTX16005-1Ru	117.8	0.67	0.14
COTX10226-1Wpe/Y	118.1	0.76	0.17
ATTX98518-5P/Y	118.3	0.76	0.19
Atlantic	118.3	0.82	0.19
NDTX050169-1R	118.6	0.80	0.21
NDTX5438-11R	118.6	0.35	0.22
TX18192-2R/Y	118.7	0.69	0.22
COTX15111-1R	118.7	0.69	0.23
COTX05211-7R	118.8	0.77	0.24
NDTX1824Y-4R	119.1	0.64	0.27
COTX13215-2Ru	119.1	0.75	0.27
TX17763-2Y/Y	119.2	0.66	0.27
COTX00104-7R	119.5	0.77	0.30
NDTX1824Y-1R	119.6	0.65	0.31
TX08385-2P/YP	119.6	0.75	0.31
TX19495-9RYpinto	119.7	0.70	0.32
NDTX071217CB-1W/Y	119.8	0.77	0.33
NDTX1759s-1R/Y	119.9	0.70	0.34
ATTX98493-2P/P	119.9	0.79	0.34
ATX16089-4Ru	120.0	0.68	0.35
NDTX071109C-1W	120.2	0.76	0.37
TX19508-5RYpinto/Y	120.2	0.70	0.37
COTX09052-1Ru	120.3	0.83	0.37
COTX09052-2Ru	120.3	0.83	0.38
Chieftain	120.4	0.68	0.39
COTX08121-4Ru	120.7	0.79	0.42
ATX15120-1Ru	120.7	0.64	0.42

ATTX98500-2P/Y	120.8	0.77	0.43
NDTX059759-3R/Y Pinto	120.8	0.80	0.43
NDTX081648CB-1W	121.2	0.36	0.46
ATTX05186-2R	121.2	0.81	0.46
ATX05186-1R	121.2	0.81	0.47
ATX11586-2R/Y	121.3	0.60	0.47
TX14611-1R	121.4	0.76	0.48
ATX84706-2Ru	121.6	0.75	0.50
AOTX91861-4R	121.7	0.77	0.51
NDTX4271-5R	122.1	0.84	0.55
PORTX03PG25-2R/R	122.3	0.75	0.57
NDTX5003-2R	122.7	0.80	0.60
TX19495-17R/Y-R	122.8	0.70	0.61
ATTX00289-6Y/Y	122.8	0.79	0.61
NDTX5067-2R	122.9	0.80	0.62
NDTX4784-7R	122.9	0.84	0.62
COTX10073s-1W	122.9	0.59	0.63
AOTX03187-1Ru	123.5	0.82	0.68
TX19495-16RYpinto	123.5	0.71	0.68
NDTX91068-11R	123.5	0.79	0.68
ATTX05175s-1R/Y	123.7	0.73	0.70
ATTX98466-5R/WR	123.7	0.82	0.70
ATX11549-1P/Y	123.8	0.67	0.71
TX1475-3W	123.9	0.78	0.71
NDTX1753Y-1R	123.9	0.75	0.72
TX17742-5YPpinto/YP	123.9	0.69	0.72
White LaSoda	124.3	0.75	0.75
ATTX961014-1R/Y Chimera	124.3	0.82	0.76
COTX15083-1R	124.4	0.64	0.76
Rio Rojo	124.4	0.77	0.77
Red LaSoda	124.6	0.72	0.79
COTX08039-1P/P*	124.6	0.78	0.79
COTX09089-1Ru	124.9	0.78	0.82
Russet Burbank	125.0	0.80	0.82
AOTX05043-1Ru	125.0	0.78	0.83
NDTX081618-1P/P	125.0	0.74	0.83
NDTX731-1R	125.1	0.76	0.83
ATTX88654-2P/Y	125.1	0.77	0.83
NDTX091908AB-2W	125.4	0.78	0.86
NDTX092237C-2P/WP	125.8	0.68	0.90
NDTX091886-3P/P	126.0	0.67	0.92
COTX03187-1W	126.1	0.82	0.92
TX17788f-4R/R	126.1	0.68	0.92
TX10437-9Pyspl/Y	126.2	0.73	0.94
ATTX01178-1R	126.5	0.76	0.96
TX17742-23P/P	126.9	0.71	1.00
COTX17016f-2P/P	127.2	0.66	1.03
NDTX12248Y-1R	127.4	0.67	1.05
TX17805-8R/R	127.8	0.71	1.09
COTX05211-4R	127.9	0.83	1.09

COTX05211-5R	127.9	0.83	1.10
BTX2103-1R/Y	128.0	0.77	1.10
ATX97147-4Ru	128.4	0.78	1.15
ATTX98453-3R	128.8	0.78	1.18
TX17755-2Psplash	129.2	0.68	1.22
TX17805-10RYpinto/Y	129.6	0.71	1.25
TX17805-1RWpinto/R	129.6	0.72	1.26
TX05249-3W	129.7	0.73	1.27
COTX04193s-2R/Y	129.9	0.76	1.28
NDTX14156-2RWpinto/Y	130.0	0.75	1.30
NDTX14156A-3RWpinto/Y	130.1	0.75	1.30
TX19489s-3Y/Y	130.2	0.66	1.31
TX17742-19R/R	130.8	0.69	1.37
AOTX02136-1Ru	131.1	0.77	1.40
BTX2332-1R	131.2	0.77	1.41
AOTX93483-1R	131.2	0.75	1.41
TX17805-12R/R	131.5	0.70	1.44
COTX08365F-3P/P	131.5	0.78	1.44
ATX9202-3Ru	131.6	0.73	1.44
PTTX05PG07-1W	131.8	0.71	1.46
NDTX092237C-2P/W	132.6	0.78	1.54
ATTX01180-1R/Y	132.6	0.78	1.54
COTX17115f-3R/R	132.6	0.65	1.54
TX05249-10W	132.7	0.75	1.55
AOTX97213-1Ru	133.8	0.78	1.66
ATTX98453-6R	133.9	0.68	1.67
Reveille Russet	134.2	0.78	1.69
NDTX14156-3P/WP	134.8	0.70	1.75
ATTX88481-1P/W	134.9	0.83	1.76
COTX94216-1R	135.0	0.51	1.77
TX09396-1W	135.1	0.77	1.78
ATTX99325-1P	135.7	0.82	1.84
ATTX98500-3P/Y	136.0	0.80	1.86
NDTX092238Cs-1P/W	136.5	0.76	1.91
ATTX98453-11Br	136.6	0.77	1.92
COTX10138-19P/Y	138.3	0.77	2.08
TX03196-1W	139.7	0.73	2.21
TX05249-11W	141.7	0.78	2.40
AOTX98202-1Ru	144.6	0.77	2.68
COTX94218-1R	146.8	0.75	2.88
ATX02263-1R/Y	147.4	0.77	2.94
TX12474-1P/R	148.2	0.76	3.01
ATTX10265-4R/Y	151.0	0.75	3.29

Genotype	GEBV	r2	z_score_GEBV
COTX10138-15Wpe/Y	42.5	0.84	-2.12
ATTX98448-6R/Y	44.6	0.86	-1.96
ATTX00289-5R/Y	44.8	0.86	-1.94
NDTX059828-2W	45.7	0.85	-1.87
NDTX4271-5R	46.2	0.88	-1.83
NDTX4784-7R	46.4	0.88	-1.82
UMTX383-3Yrdspl/Y	48.1	0.87	-1.69
ATTX98491-4Yrdspl/Y	48.1	0.87	-1.69
ATTX98465-1R/Y	48.5	0.83	-1.65
NDTX5067-2R	48.6	0.87	-1.65
NDTX5003-2R	48.7	0.87	-1.64
Tokio	48.9	0.82	-1.63
COTX10138s-7Wpe/Y	49.2	0.84	-1.60
COTX01403-4R/Y	49.8	0.83	-1.56
NDTX071258B-1R	50.7	0.83	-1.49
NDTX060700C-1W	50.8	0.83	-1.48
ATTX96746-1R*	51.2	0.83	-1.45
ATX06264s-4R/Y	51.6	0.82	-1.42
TX12484-2WZC	51.6	0.82	-1.42
AOR07781-2	51.7	0.83	-1.41
COTX05211-7R	51.8	0.83	-1.40
TX12484-4W	51.9	0.84	-1.39
ATTX961014-1AR/Y	52.2	0.86	-1.37
TX19407-5Ru	52.3	0.80	-1.37
ATTX98500-2P/Y	52.8	0.83	-1.33
TX12484-3WZC	53.1	0.79	-1.30
TX18042-1Ru	53.3	0.79	-1.29
COTX02293-4R	54.2	0.84	-1.22
AOTX95309-2W*	54.5	0.88	-1.20
Sierra GoldTM	54.5	0.87	-1.20
ATTX98462s-3R/Y	54.5	0.88	-1.19
ATTX961014-1Br/Y	54.5	0.88	-1.19
Sierra RoseTM	54.6	0.88	-1.19
ATX15097-1Ru	54.9	0.78	-1.16
NDTX91068-11R	55.3	0.84	-1.13
ATTX99325-1P	55.6	0.86	-1.11
ATTX88481-1P/W	55.8	0.86	-1.09
NDTX1791-1Ru	56.1	0.79	-1.07
TX09403-15W	56.3	0.87	-1.05
TX09403-21W	56.6	0.87	-1.03
COTX03134-1Y/W	56.7	0.76	-1.02
COTX10118-4Wpe/Y	56.8	0.84	-1.02
NDTX081648CB-13W	56.8	0.83	-1.01
COTX05261-1R/Y	56.9	0.82	-1.01
TXYG79	56.9	0.85	-1.01
NDTX7590-3R	56.9	0.83	-1.01
Krantz	57.0	0.82	-1.00
ATX03564-1Y/Y	57.0	0.87	-1.00
Banana	57.0	0.79	-1.00

ATX03496-3Y/Y	57.1	0.87	-0.99
COTX13029-2Ru/Y	57.4	0.77	-0.97
ATTX98510-1R/Y	57.5	0.86	-0.96
ATTX01180-1R/Y	57.5	0.83	-0.96
NDTX4756-1R/Y	57.5	0.86	-0.96
BTX1544-2W/Y	58.1	0.82	-0.91
TX19460-1W	58.4	0.78	-0.89
BTX1749-1W/Y	58.5	0.83	-0.88
NDTX081451CBs-1Y/Y	58.6	0.83	-0.88
COTX16005-1Ru	58.8	0.78	-0.86
NDTX14263BC-3W	58.8	0.78	-0.86
ATTX07042-3W	59.0	0.83	-0.84
COTX05249-3W/Y	59.7	0.81	-0.79
Yukon Gold	59.9	0.83	-0.77
COTX03079-1W/W	60.0	0.84	-0.77
COTX18206-1Ru	60.0	0.78	-0.77
ATX11684f-2W/Y	60.3	0.77	-0.74
TX11461-3W	60.6	0.83	-0.72
TX19093-1Rure	60.8	0.77	-0.70
COTX10080-2Ru	60.9	0.86	-0.70
ATTX98514-1R/Y	61.3	0.81	-0.67
COTX04193s-2R/Y	61.4	0.82	-0.66
NDTX081644-CAB-2W	61.4	0.81	-0.66
COTX08258-6Ru	61.5	0.84	-0.66
TX1673-1W/Y	61.7	0.85	-0.64
ATTX98493-2P/P	61.7	0.84	-0.63
ATX9117-1Ru	61.8	0.83	-0.63
COTX89044-1Ru	61.8	0.83	-0.63
NDTX059886S-1Y/Y	61.8	0.49	-0.63
COTX17288-1W	61.8	0.79	-0.63
COTX00104-6R	61.9	0.84	-0.62
COTX05211-5R	61.9	0.87	-0.62
COTX04015-3W/Y	62.1	0.82	-0.60
COTX05211-4R	62.2	0.87	-0.60
TX1617-1W/Y	62.2	0.83	-0.60
NDTX14362AB-1W	62.3	0.79	-0.59
ATTX03516-2R	62.4	0.83	-0.58
ATX13134-3W/Y	62.4	0.78	-0.58
COTX05095-2Ru/Y	62.5	0.83	-0.58
COTX10118-1Wre/Y	62.8	0.83	-0.55
COTX08039-1P/P*	63.2	0.84	-0.52
ATTX98518-5P/Y	63.3	0.82	-0.52
ATTX00289-4W	63.4	0.85	-0.51
TX19411-4W	63.5	0.79	-0.50
TX11461-2W	63.5	0.84	-0.50
NDTX6773-1W	63.5	0.84	-0.50
TX17797-11Y/Y	63.5	0.77	-0.50
ATTX00289-6Y/Y	63.6	0.84	-0.49
NDTX1824Y-1R	63.7	0.77	-0.48
NDTX092238Cs-1P/W	63.7	0.83	-0.48

COTX02172-1R	63.8	0.84	-0.48
AORTX09037-5W/Y	63.8	0.78	-0.48
Chieftain	63.8	0.79	-0.47
ATTX98468-5Ru/Y*	64.2	0.84	-0.45
MWTX548-2Ru	64.8	0.87	-0.40
ATX9130-1Ru	65.0	0.84	-0.39
NDTX092237C-2P/WP	65.0	0.80	-0.38
ATTX98493-1AR	65.1	0.84	-0.37
ATTX88654-2P/Y	65.2	0.83	-0.37
NDTX14156A-3RWpinto/Y	65.3	0.83	-0.36
MWTX2609-4Ru	65.3	0.87	-0.36
PORTX03PG25-2R/R	65.3	0.83	-0.36
NDTX14156-2RWpinto/Y	65.4	0.83	-0.35
NDTX050184s-1R/Y	65.5	0.83	-0.34
TX6-1216-1Ru	65.6	0.83	-0.34
COTX09022-3RuRE/Y	65.6	0.82	-0.34
MWTX2609-2Ru	65.7	0.87	-0.33
ATTX98466-5R/WR	65.9	0.86	-0.32
COTX94218-1R	65.9	0.83	-0.31
AOTX95309-1W	66.0	0.85	-0.30
NDTX5438-11R	66.1	0.47	-0.30
ATX85404-8W	66.1	0.85	-0.30
TX19511-5RYpinto/Y	66.1	0.79	-0.29
JTTX75/2003EH-1Yre/Y	66.2	0.84	-0.29
COTX90046-1W	66.2	0.82	-0.29
ATTX05175s-1R/Y	66.3	0.82	-0.28
Tacna	66.4	0.74	-0.28
COTX10012-1Wrdspl/Y	66.5	0.83	-0.27
NDTX1244-3W/Y	66.5	0.79	-0.26
TX17742-5YPpinto/YP	66.6	0.79	-0.26
COTX04050s-1P/P	66.7	0.82	-0.25
COTX08063-2Ru	66.8	0.83	-0.24
COTX15111-1R	67.0	0.79	-0.23
ATTX961014-1R/Y Chimera	67.0	0.86	-0.23
COTX87601-2Ru	67.1	0.82	-0.22
ATTX10007-1Ru	67.1	0.79	-0.22
ATTX95490-2W	67.3	0.83	-0.21
ATTX01178-1R	67.4	0.84	-0.20
TX08385-2P/YP	67.5	0.82	-0.19
NDTX1824Y-4R	67.6	0.76	-0.18
TX17763-2Y/Y	67.6	0.78	-0.18
COTX17288-4W	67.8	0.84	-0.17
COTX17288-3W	67.9	0.84	-0.16
TX19009s-2W/Y	68.0	0.78	-0.15
ATX05186-1R	68.1	0.85	-0.14
ATTX98444s-16R/Y	68.3	0.83	-0.12
NDTX14247CAB-2W	68.4	0.83	-0.12
NDTX14247CAB-1W	68.4	0.83	-0.12
COTX08322-10Ru	68.6	0.85	-0.11
Stampede Russet	68.7	0.81	-0.09

ATX07305S-1Y/Y	68.8	0.83	-0.09
ATTX98453-11Br	69.1	0.84	-0.06
TX17846-1W	69.2	0.77	-0.06
ATX15325-3W/Y	69.5	0.79	-0.03
NDTX1246-5W/Y	69.6	0.76	-0.03
COTX17304-1Ru	69.6	0.76	-0.03
NDTX059775-1W	69.7	0.83	-0.02
NDTX081618-1P/P	69.9	0.81	0.00
AOTX96216-1Ru	70.0	0.85	0.01
BTX2332-1R	70.2	0.83	0.02
ATX84378-6Ru	70.3	0.85	0.02
NDTX12248Y-1R	70.3	0.78	0.03
COTX15271-1Ru	70.3	0.78	0.03
AOTX96216-2Ru	70.3	0.84	0.03
COTX03187-1W	70.5	0.86	0.04
ATTX05186-2R	70.5	0.84	0.04
AORTX09037-1W/Y	70.6	0.83	0.05
TX17734-1Y/Y	70.6	0.77	0.05
COTX09089-1Ru	70.7	0.83	0.06
TX14611-1R	70.9	0.82	0.07
COTX12235-2W	70.9	0.77	0.07
COTX16054-1W	70.9	0.79	0.07
COTX10226-1Wpe/Y	71.2	0.84	0.09
AOTX93483-1R	71.6	0.82	0.12
AOTX03187-1Ru	71.6	0.85	0.13
TX11454-9Ru/Y	71.7	0.79	0.13
NDTX1482YB-1W	71.8	0.77	0.14
COTX90046-5W	72.0	0.83	0.16
ATX02263-1R/Y	72.1	0.83	0.16
NDTX050169-1R	72.1	0.85	0.16
TX18192-2R/Y	72.3	0.79	0.18
Atlantic	72.4	0.87	0.19
NDTX731-1R	72.4	0.82	0.19
NDTX059761-1R/R	72.8	0.85	0.22
COTX08121-1Ru	73.2	0.84	0.25
COTX94216-1R	73.3	0.60	0.26
TX19508-5RYpinto/Y	73.5	0.79	0.28
NDTX1753Y-1R	73.9	0.82	0.31
White LaSoda	74.2	0.83	0.33
NDTX081648CB-1W	74.2	0.43	0.33
ATX16089-4Ru	74.3	0.79	0.33
COTX15083-1R	74.3	0.76	0.34
NDTX8773-4Ru	74.4	0.83	0.35
ATTX98500-3P/Y	74.5	0.86	0.35
BTX2103-1R/Y	74.5	0.84	0.35
ATTX10333-1W/Y	74.9	0.78	0.38
TX17788f-4R/R	75.0	0.79	0.39
NDTX4930-5W	75.3	0.83	0.41
COTX00104-7R	75.5	0.83	0.43
TX05249-10W	75.8	0.83	0.45

Rio Rojo	75.9	0.83	0.46
TX03196-1W	76.3	0.82	0.49
COTX08121-4Ru	76.5	0.85	0.50
ATX05202s-3W/Y	76.5	0.84	0.51
TX09414-1W	76.6	0.83	0.51
TX09396-1W	76.7	0.84	0.52
NDTX059759-3R/Y Pinto	77.1	0.85	0.55
NDTX091908AB-2W	77.2	0.84	0.56
ATX11586-2R/Y	77.9	0.78	0.61
COTX10138-19P/Y	78.0	0.83	0.62
TX05249-3W	78.1	0.83	0.63
Red LaSoda	78.2	0.80	0.63
NDTX14156-3P/WP	78.2	0.79	0.64
NDTX071109C-1W	78.3	0.83	0.64
ATX87184-2Ru	78.4	0.83	0.65
ATX9332-8Ru	78.6	0.79	0.67
NDTX1759s-1R/Y	78.8	0.80	0.69
NDTX081648CB-4W	79.2	0.82	0.72
NDTX071217CB-1W/Y	79.3	0.84	0.72
ATX9202-3Ru	79.4	0.78	0.73
COTX17286-6W	79.7	0.79	0.75
TX17755-2Psplash	79.9	0.78	0.76
ATX08181-5Y/Y	80.0	0.83	0.78
TX1475-3W	80.1	0.83	0.79
AOTX91861-4R	80.3	0.83	0.80
ATX97147-4Ru	80.4	0.83	0.81
ATTX10265-4R/Y	80.4	0.82	0.81
TX19495-9RYpinto	80.5	0.80	0.81
NDTX4828-2R	80.8	0.84	0.84
AOTX98202-1Ru	80.8	0.83	0.84
ATTX98453-3R	80.9	0.84	0.84
AOTX05043-1Ru	81.1	0.83	0.86
TX08350-12Ru	81.3	0.84	0.87
ATX15120-1Ru	81.3	0.77	0.88
AOTX95265-2ARu	81.6	0.85	0.90
AOTX97213-1Ru	81.6	0.84	0.90
COTX17123-2Ru	81.9	0.78	0.92
ATTX98453-6R	82.0	0.83	0.93
TX17742-19R/R	82.3	0.79	0.95
COTX10073s-1W	82.6	0.68	0.97
COTX09052-2Ru	82.6	0.87	0.98
COTX09052-1Ru	82.6	0.87	0.98
AOTX95265-4Ru	83.0	0.92	1.01
AOTX02136-1Ru	83.2	0.84	1.02
AOTX96084-1Ru	83.4	0.89	1.04
TX10437-9Pyspl/Y	83.5	0.78	1.05
Russet Norkotah 296	83.6	0.93	1.06
Russet Norkotah 223	83.6	0.93	1.06
TXNS118	83.6	0.93	1.06
AOTX95295-3Ru	83.6	0.93	1.06

Russet Norkotah	83.7	0.93	1.06
AOTX95265-1Ru	83.7	0.93	1.06
Russet Norkotah 278	83.7	0.93	1.06
AOTX95265-3Ru	83.7	0.93	1.06
AOTX98137-1Ru	83.7	0.93	1.06
Russet Norkotah 102	83.7	0.93	1.06
TXNS249	83.7	0.93	1.06
COTX04303-3Ru/Y*	83.7	0.93	1.06
ATX99013-1Ru	83.7	0.93	1.07
TXNS106	83.8	0.93	1.07
AOTX98096-1Ru*	83.8	0.93	1.07
AOTX96075-1Ru	83.8	0.93	1.07
TX19489s-3Y/Y	83.8	0.78	1.07
Russet Norkotah 112	84.0	0.93	1.08
AOTX96208-1Ru	84.3	0.91	1.11
AOTX95265-2Ru	84.3	0.89	1.11
TX17805-10RYpinto/Y	85.1	0.81	1.17
TX19495-17R/Y-R	86.2	0.80	1.26
ATX11549-1P/Y	87.1	0.78	1.33
PTTX05PG07-1W	87.2	0.83	1.33
COTX08365F-3P/P	87.6	0.84	1.37
Russet Burbank	88.0	0.85	1.39
TX17805-1RWpinto/R	88.3	0.81	1.41
COTX17016f-2P/P	88.4	0.78	1.43
TX17742-23P/P	88.5	0.80	1.43
NDTX091886-3P/P	88.7	0.82	1.45
Reveille Russet	89.0	0.85	1.47
TX19495-16RYpinto	89.1	0.81	1.48
ATX9312-1Ru	90.6	0.78	1.60
ATX84706-2Ru	91.0	0.83	1.63
AOTX98152-3Ru	91.8	0.88	1.69
TXA549-1Ru	91.8	0.88	1.69
TX13590-9Ru	91.9	0.88	1.70
COTX13215-2Ru	92.7	0.81	1.76
NDTX092237C-2P/W	92.8	0.83	1.76
TX17805-8R/R	93.6	0.80	1.82
ATTX06246-1R	94.2	0.81	1.87
COTX17115f-3R/R	96.5	0.78	2.05
TX05249-11W	97.4	0.84	2.12
Vanguard Russet	105.7	0.85	2.76
CO112-F2-2P/P	107.5	0.82	2.90
TX12474-1P/R	115.6	0.85	3.53
TX17805-12R/R	136.9	0.80	5.17
