# Supplementary material for: Genome‐wide association studies and genomics‐informed breeding for tuber dormancy in tetraploid potatoes
Source: Plant Genome. 2026 Feb 19;19(1):e70205. doi: 10.1002/tpg2.70205 (PMC12919731; doi:10.1002/tpg2.70205)
Supplement: Supplementary file 4 [file TPG2-19-e70205-s006.pdf]

Genome-wide association studies and genomics-informed breeding for tuber dormancy in tetraploid potatoes

Ao Jiao, Sanjeev Gautam, Jeewan Pandey, Douglas C. Scheuring, Jeffrey W. Koym, and M. Isabel Vales

Supplementary Figures

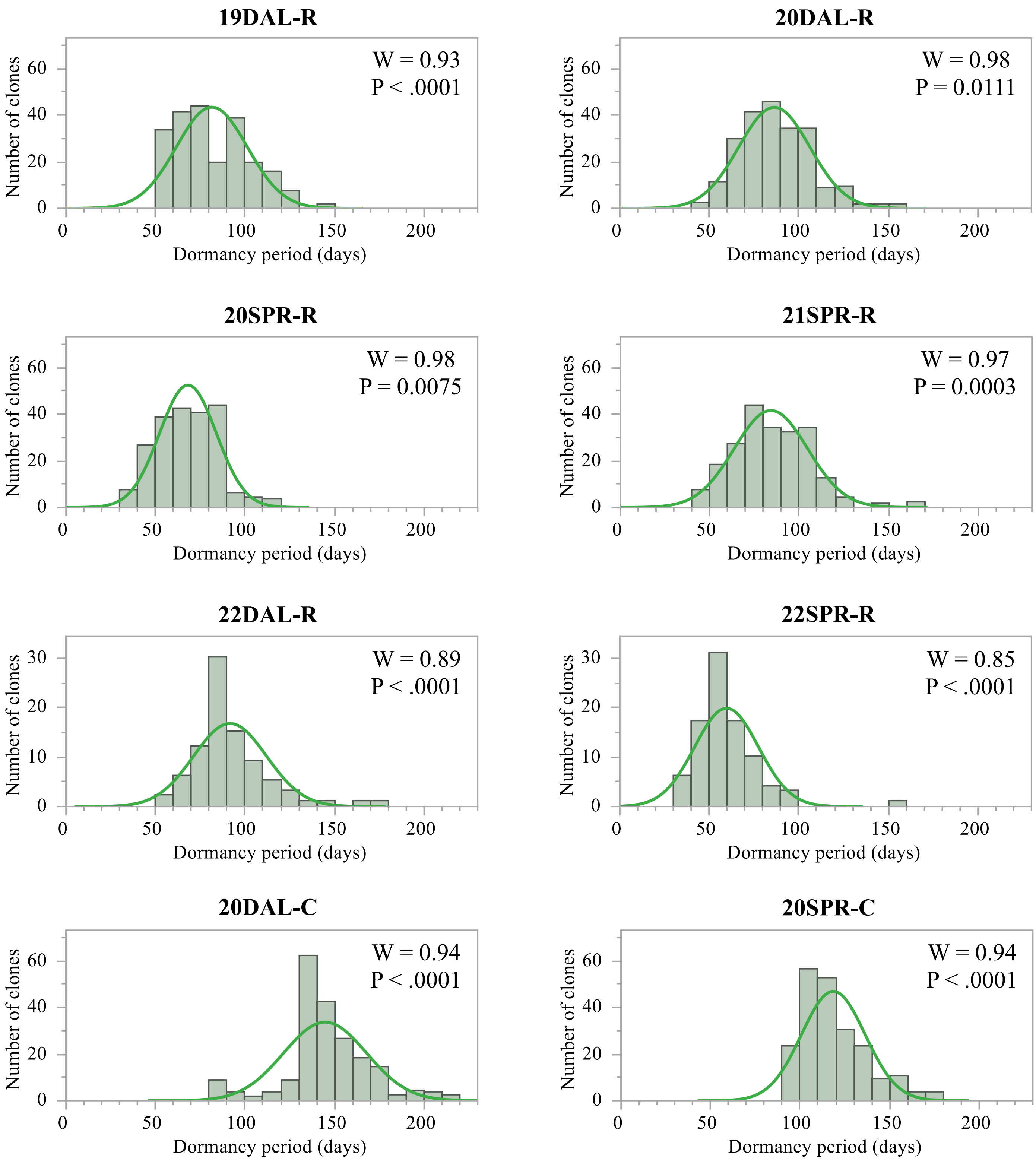

19DAL-R
W = 0.93
P < .0001
20DAL-R
W = 0.98
P = 0.0111
20SPR-R
W = 0.98
P = 0.0075
21SPR-R
W = 0.97
P = 0.0003
22DAL-R
W = 0.89
P < .0001
22SPR-R
W = 0.85
P < .0001
20DAL-C
W = 0.94
P < .0001
20SPR-C
W = 0.94
P < .0001
Number of clones
Dormancy period (days)
0
50
100
150
200
0
20
40
60
0
10
20
30

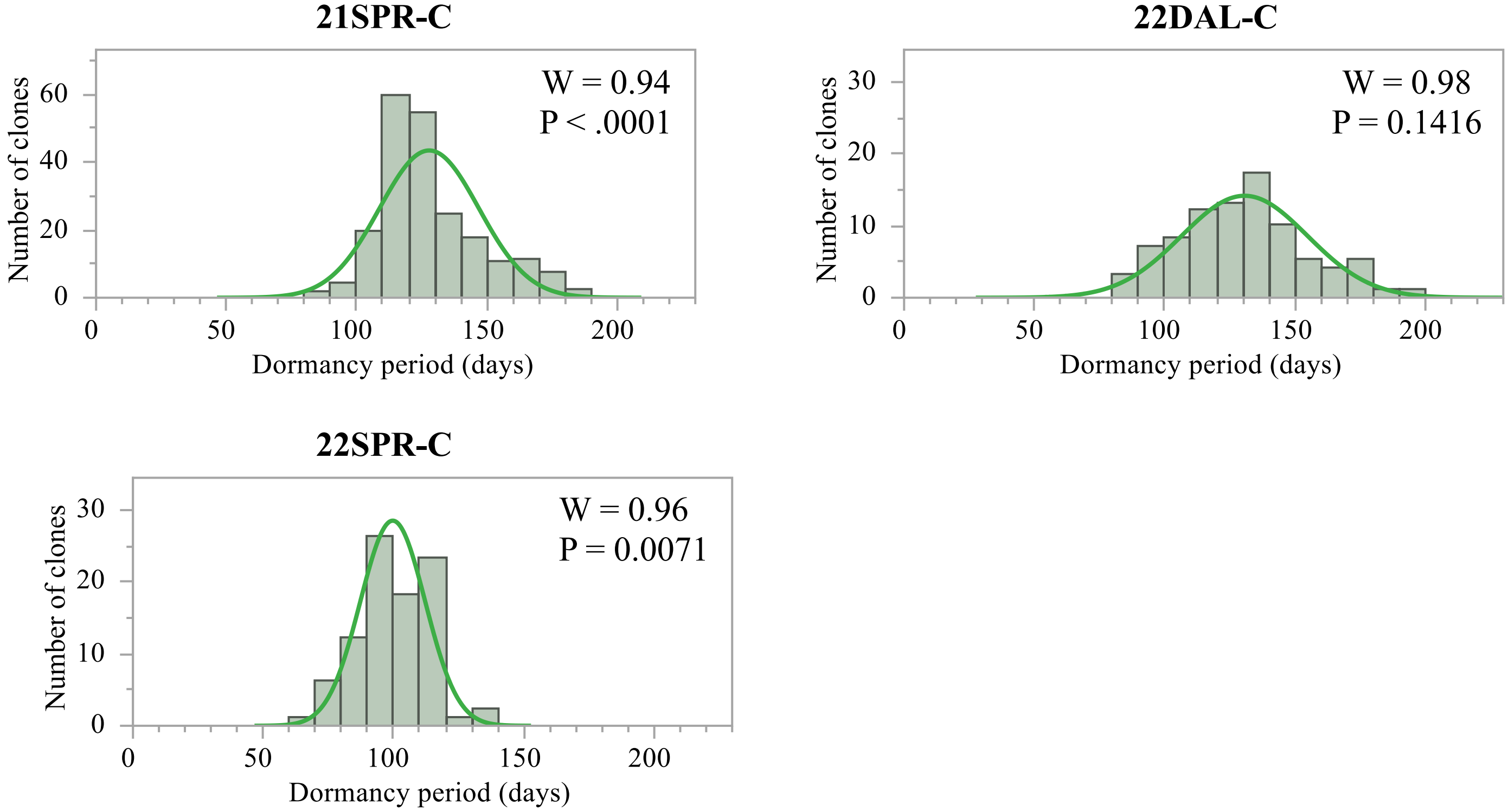

Supplementary Figure S1. Distribution of tuber dormancy period lengths measured in 298 potato genotypes in eleven environments involving two locations (Dalhart and Springlake), four years (2019 - 2022), and two storage conditions (room temperature and cold storage). 19, 20, 21, 22 – years 2019, 2020, 2021, 2022; DAL – Dalhart; SPR – Springlake; R – room temperature; C – cold storage.

19DAL_R

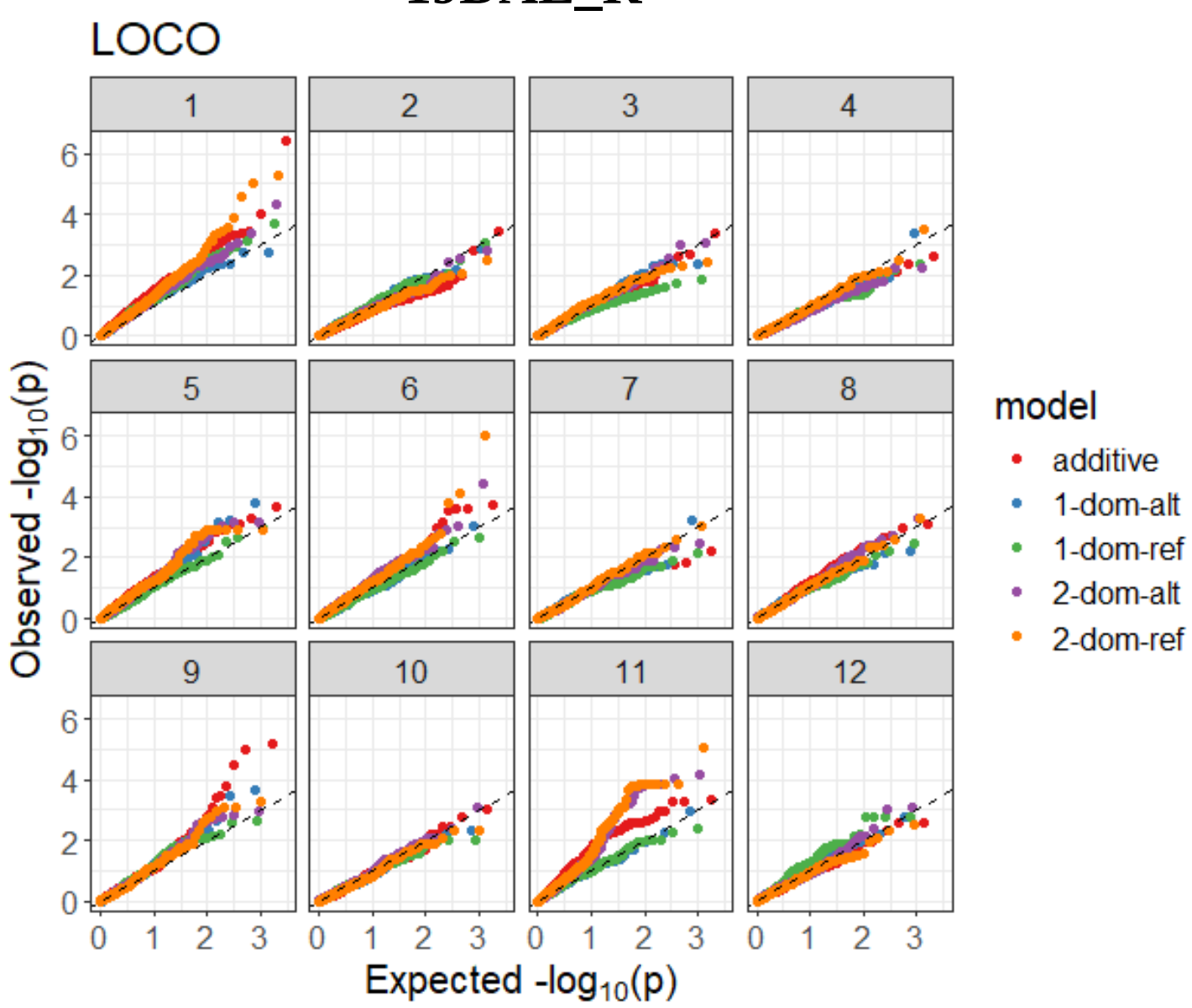

20DAL_R

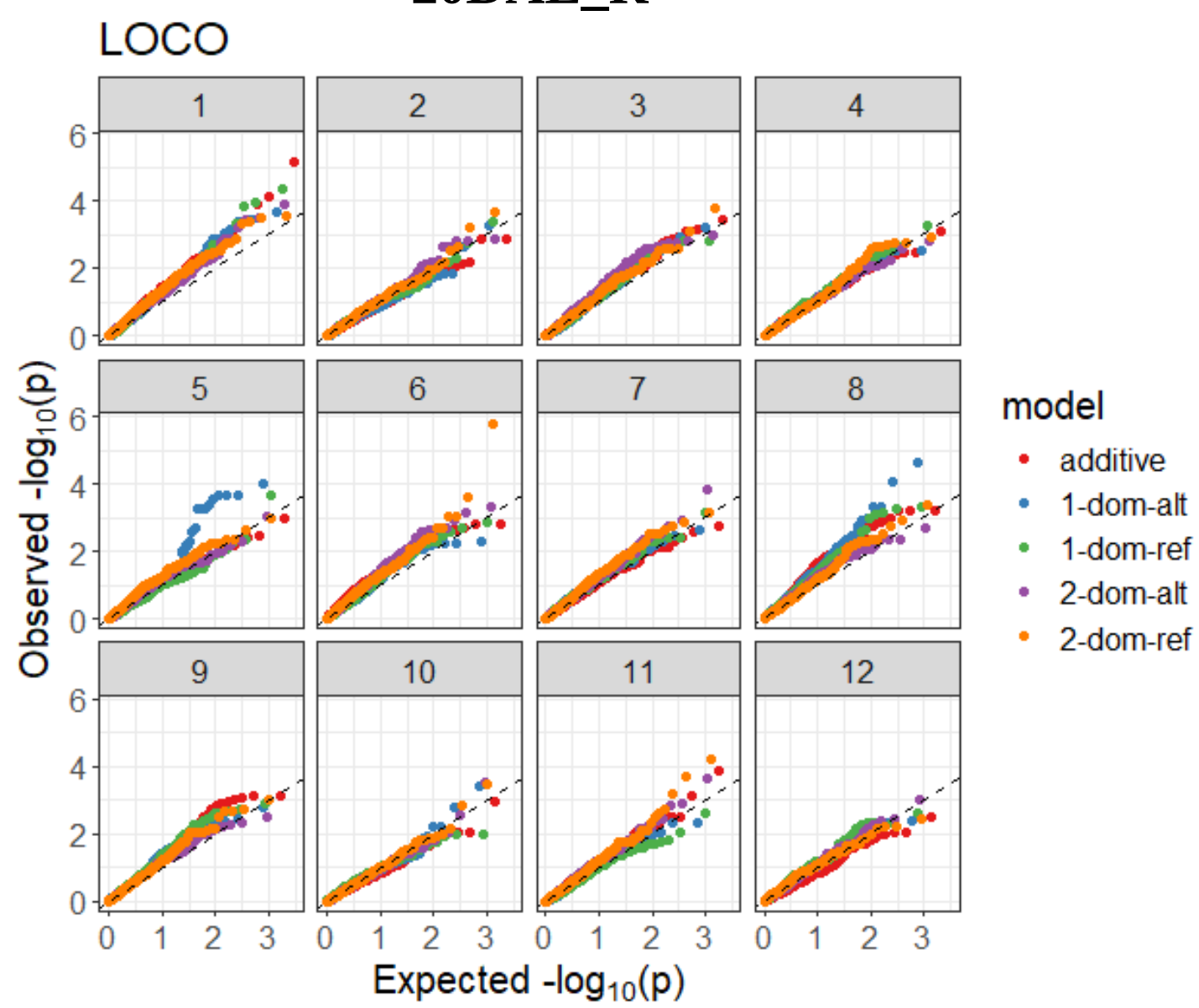

20DAL_C

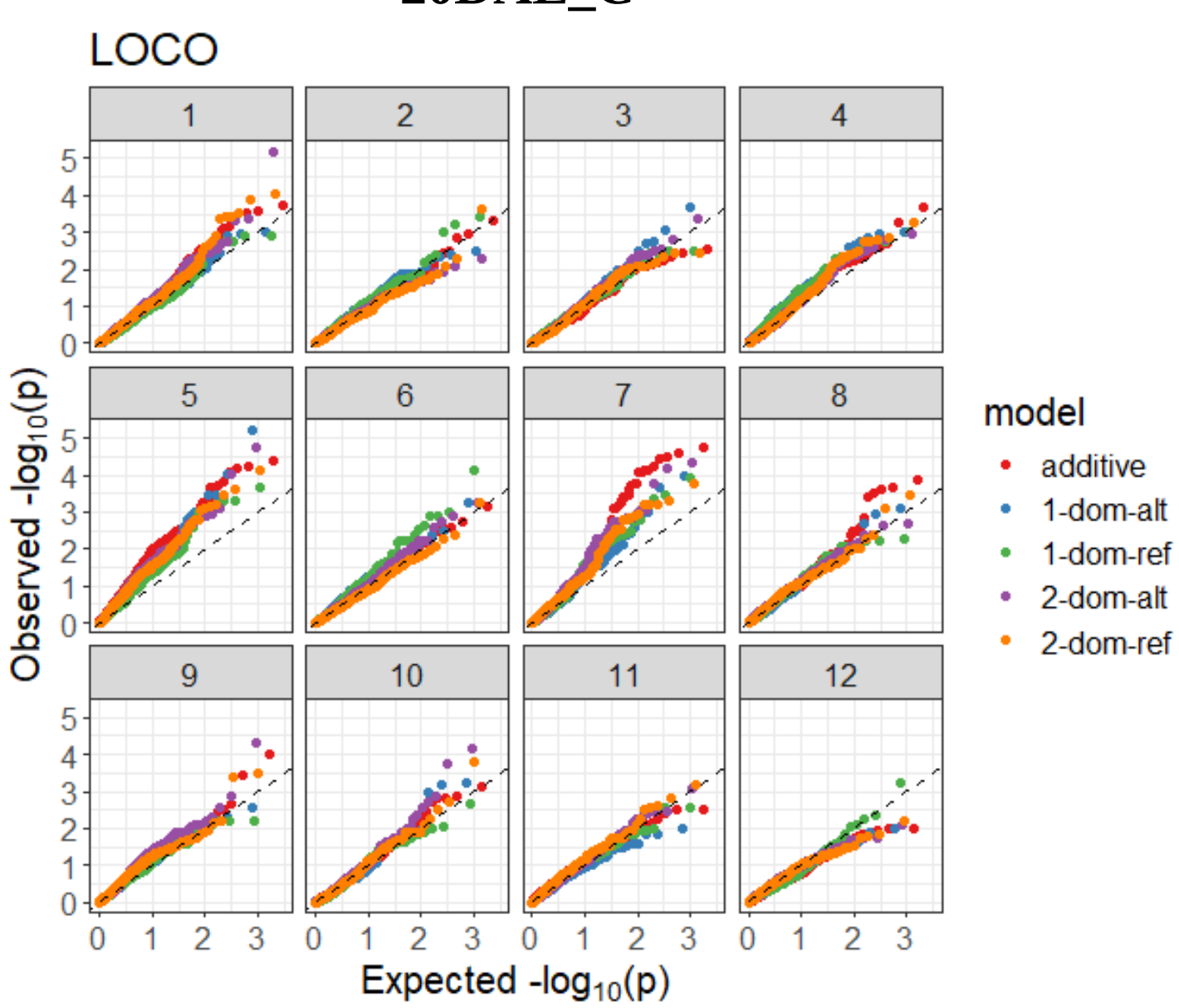

20SPR_R

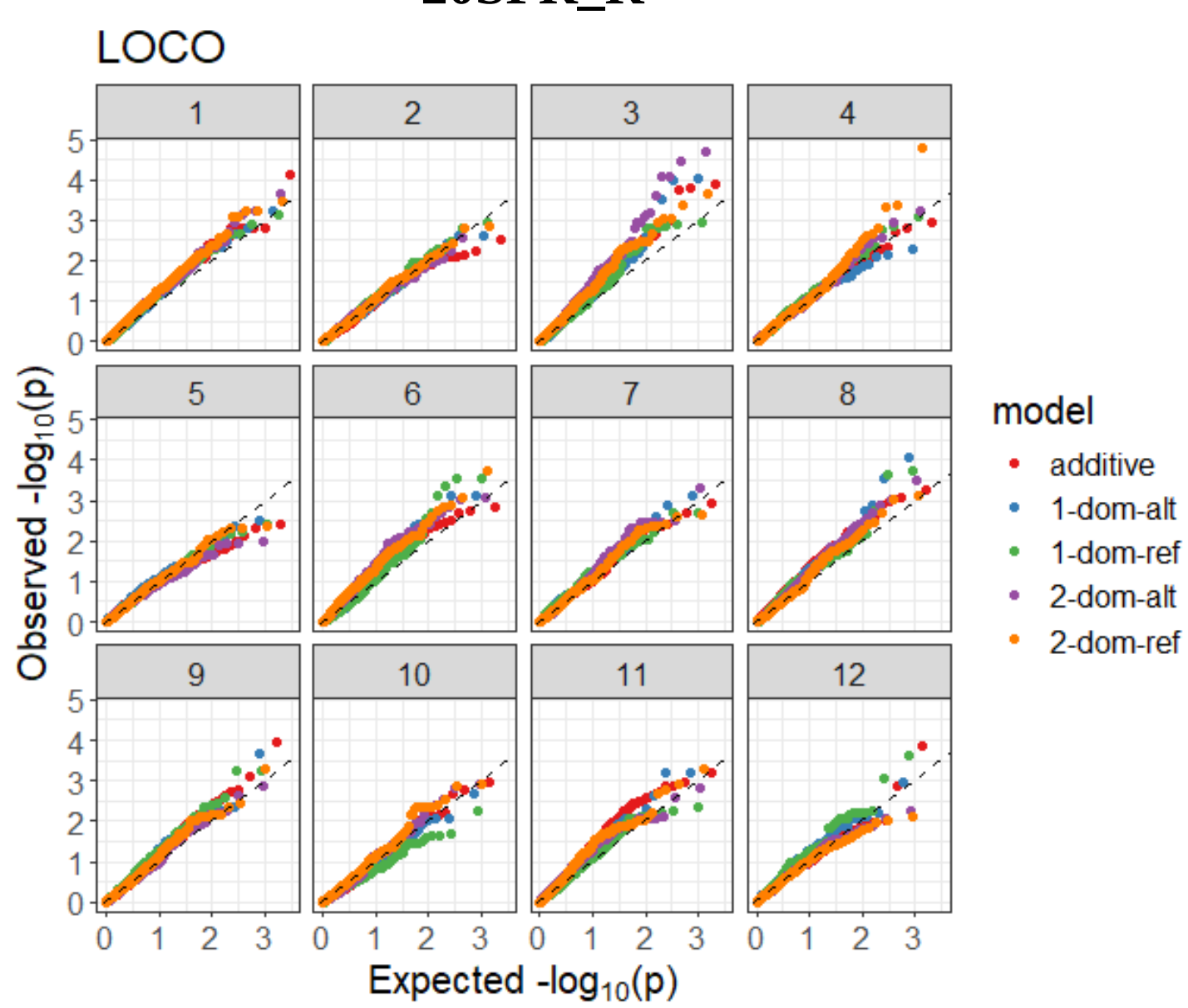

21SPR_C

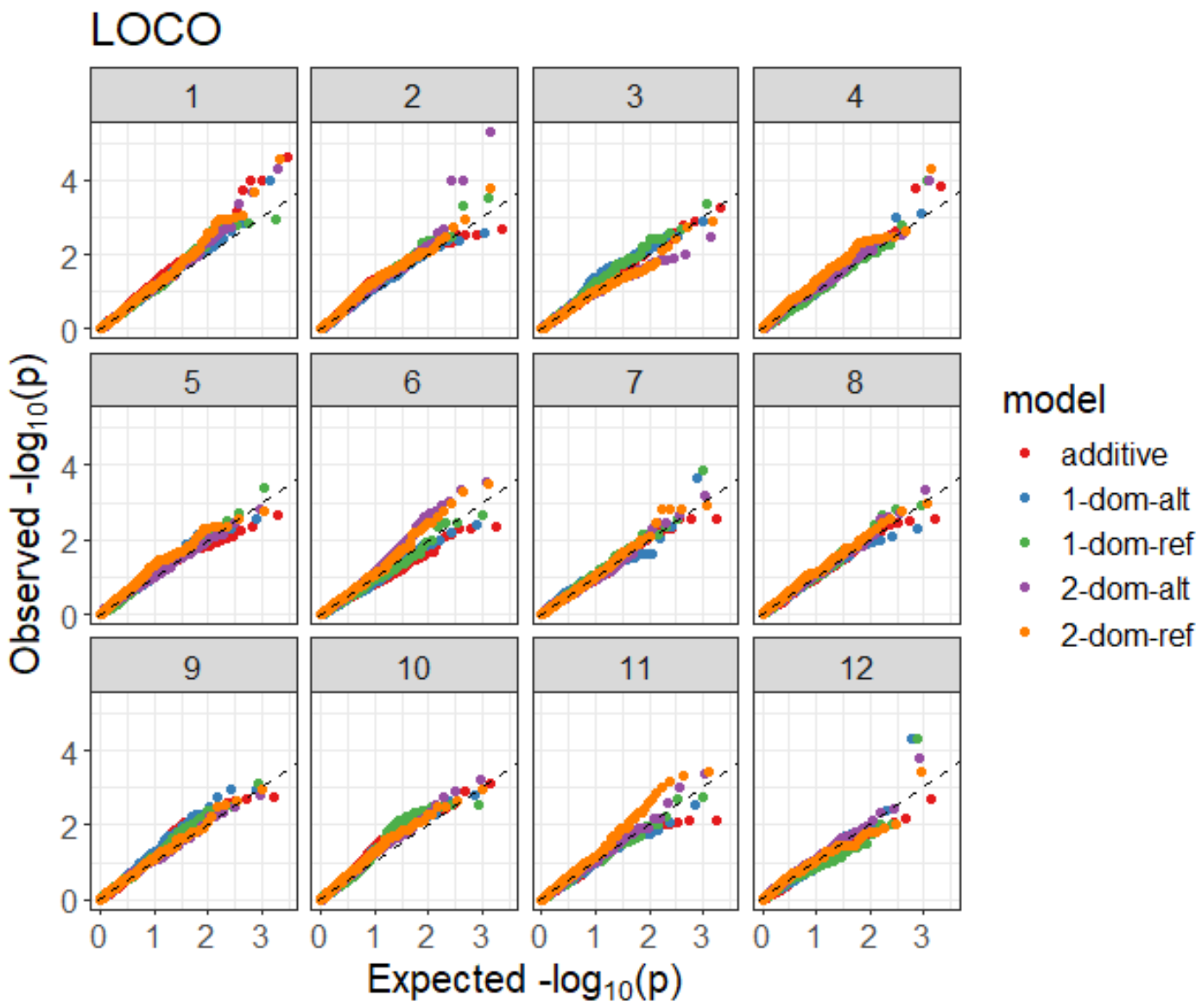

20SPR_C

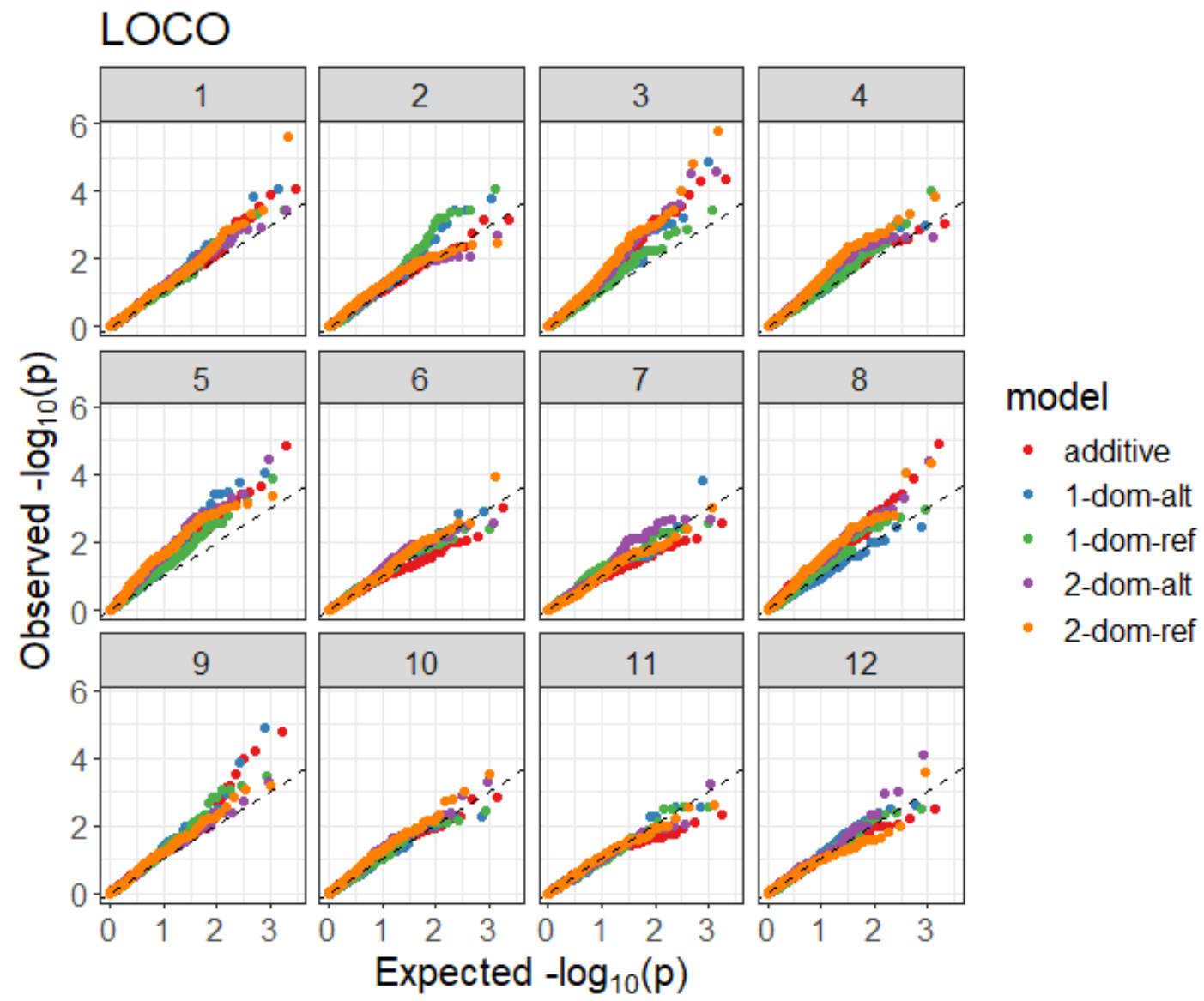

21SPR_R

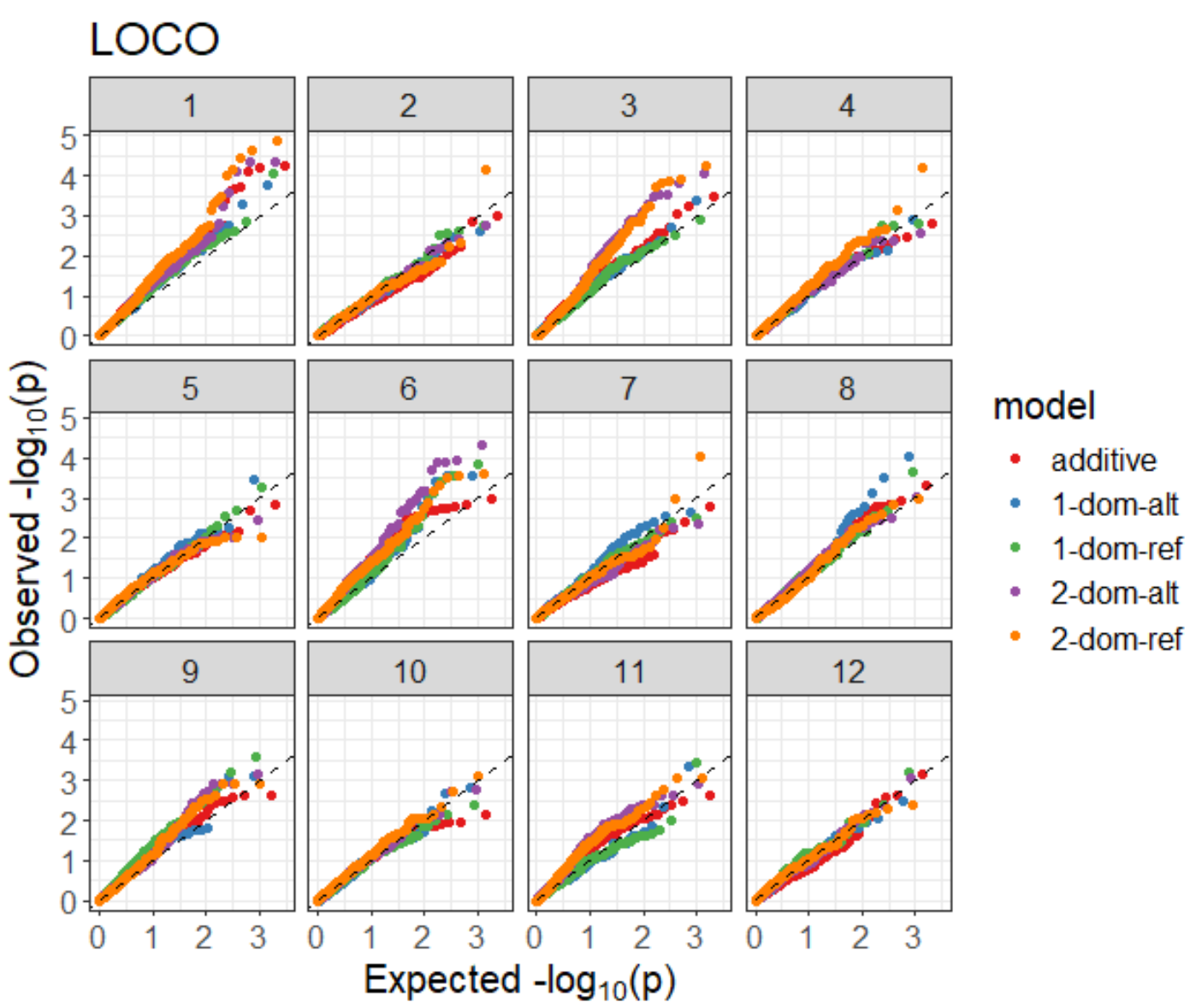

Supplementary Figure S2. Quantile-quantile (QQ) plots of observed vs. expected -log10(p values) for potato tuber dormancy using the additive and dominant model. Based on 216 potato genotypes evaluated in seven environments involving two locations (Dalhart and Springlake, Texas), three years (2019 - 2021), and two storage conditions (room temperature and cold storage). 19, 20, 21 – years 2019, 2020, 2021; DAL – Dalhart; SPR – Springlake; R – room temperature; C – cold storage.

DAL_R

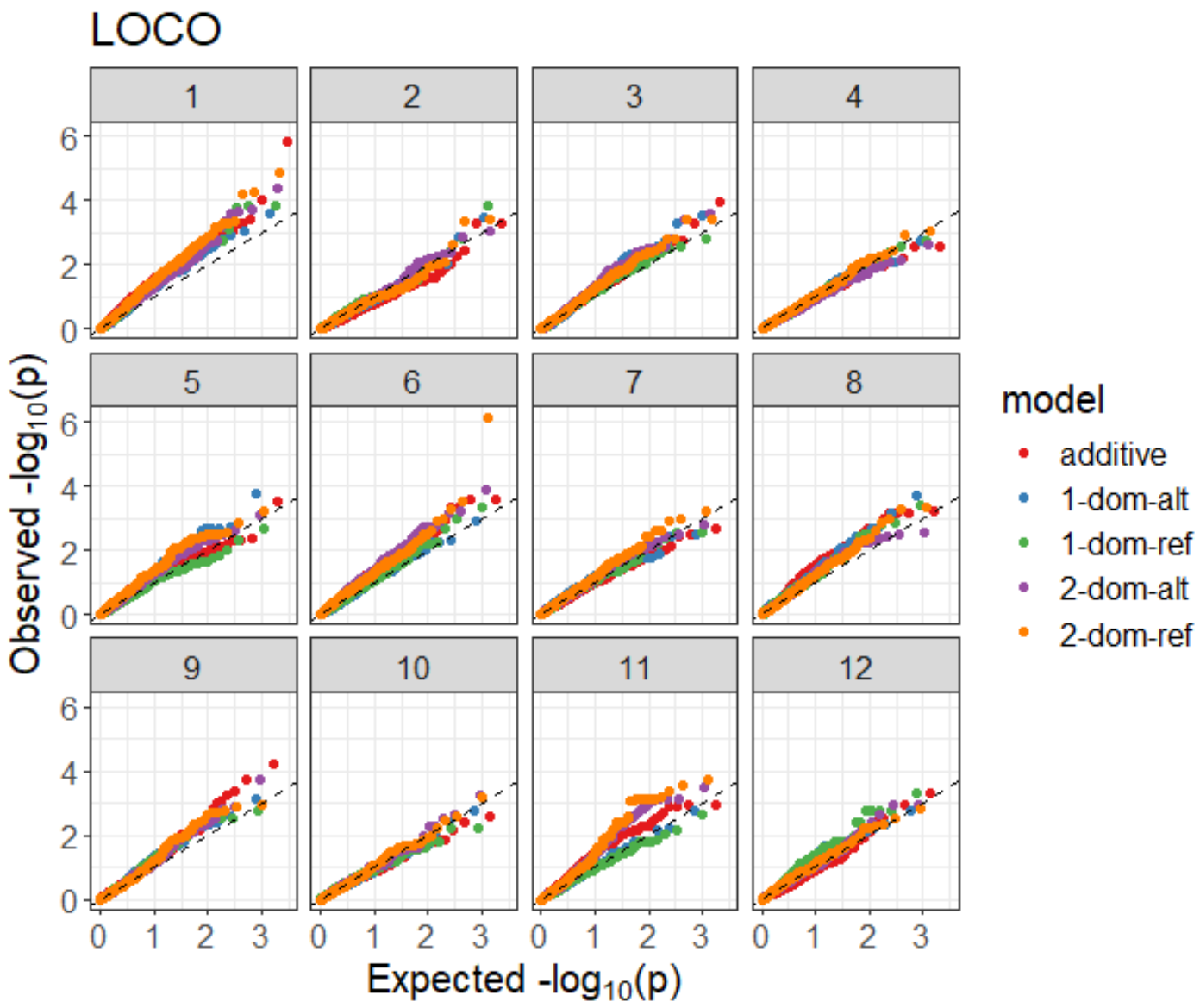

DAL_C

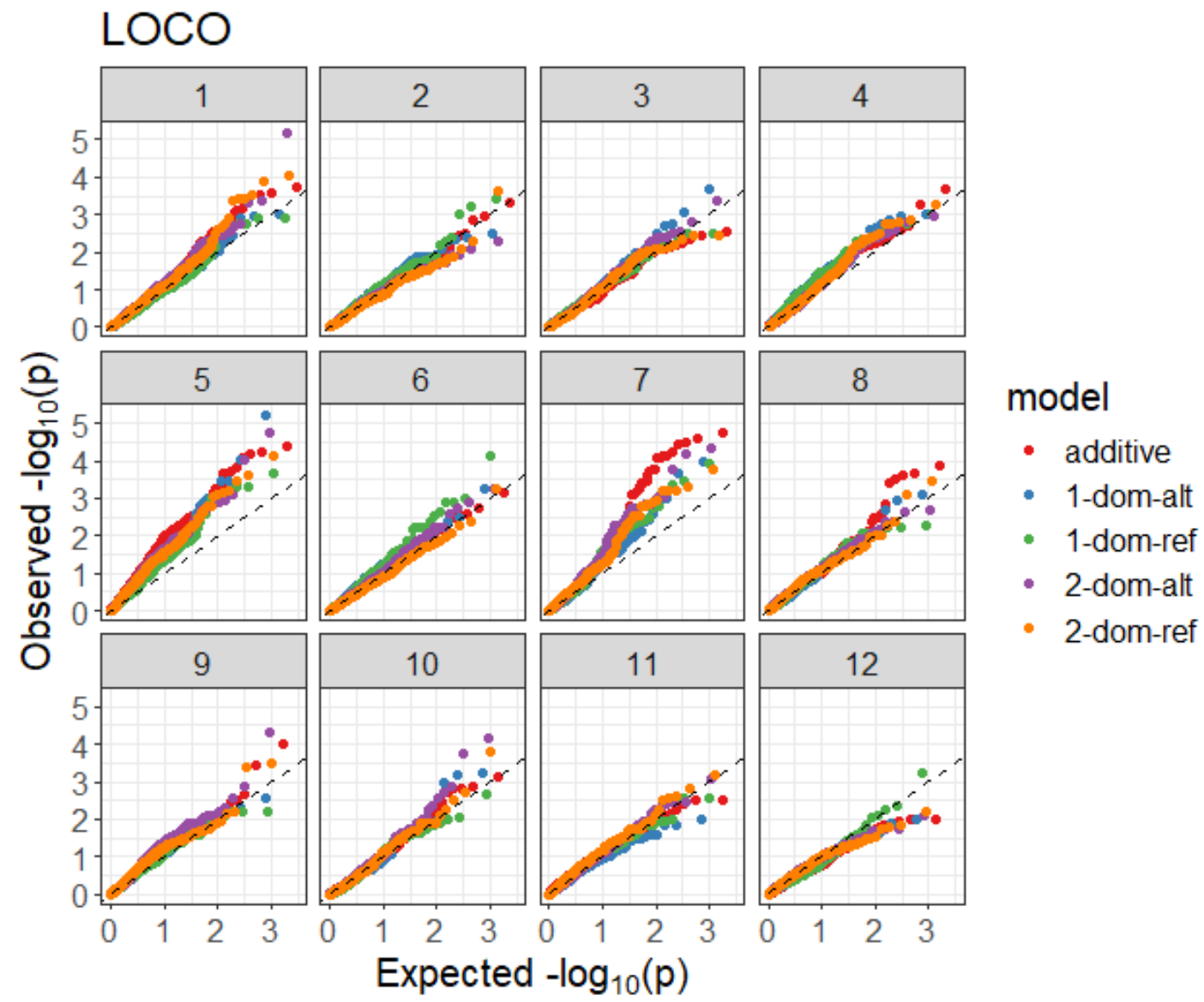

SPR_R

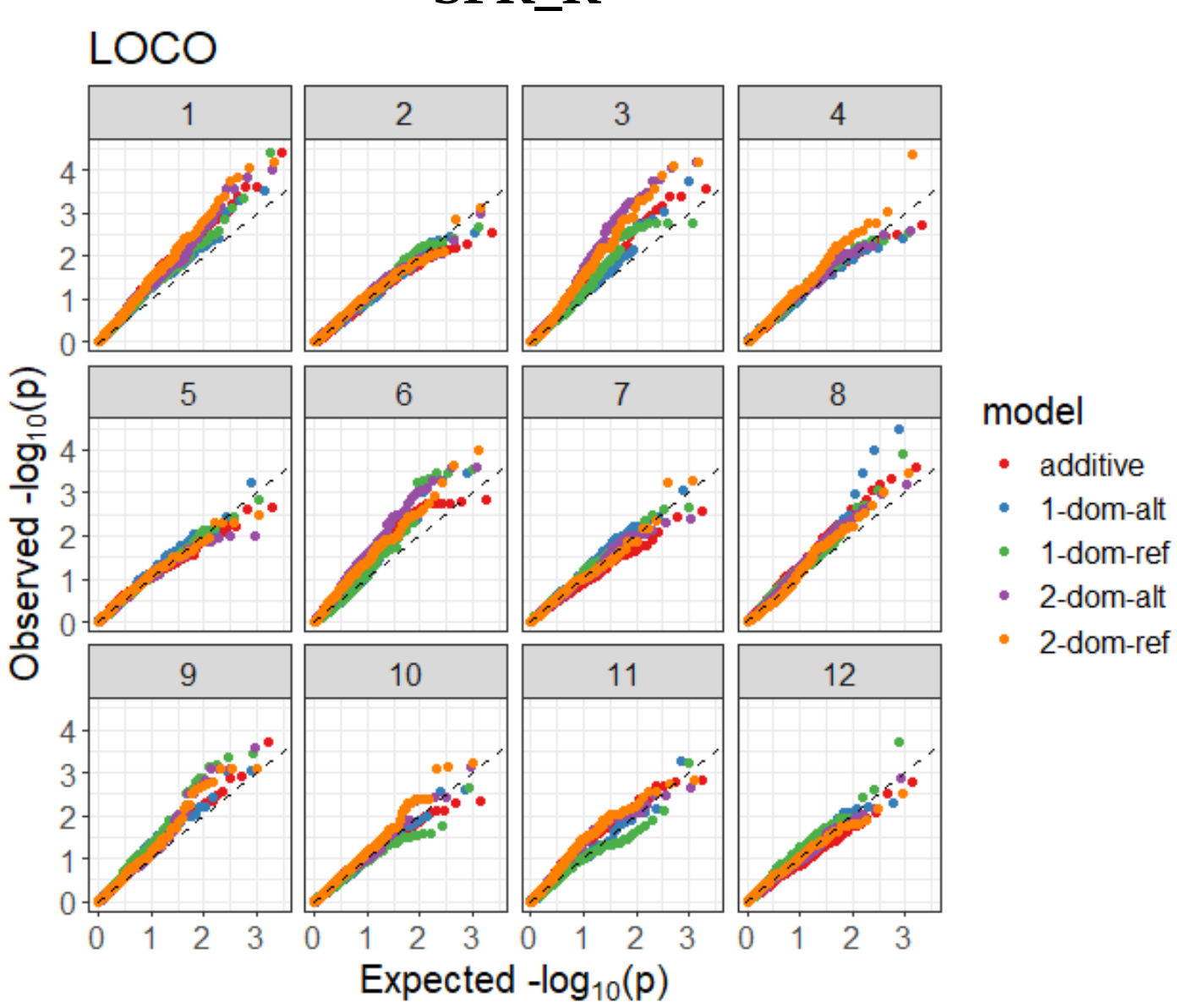

SPR_C

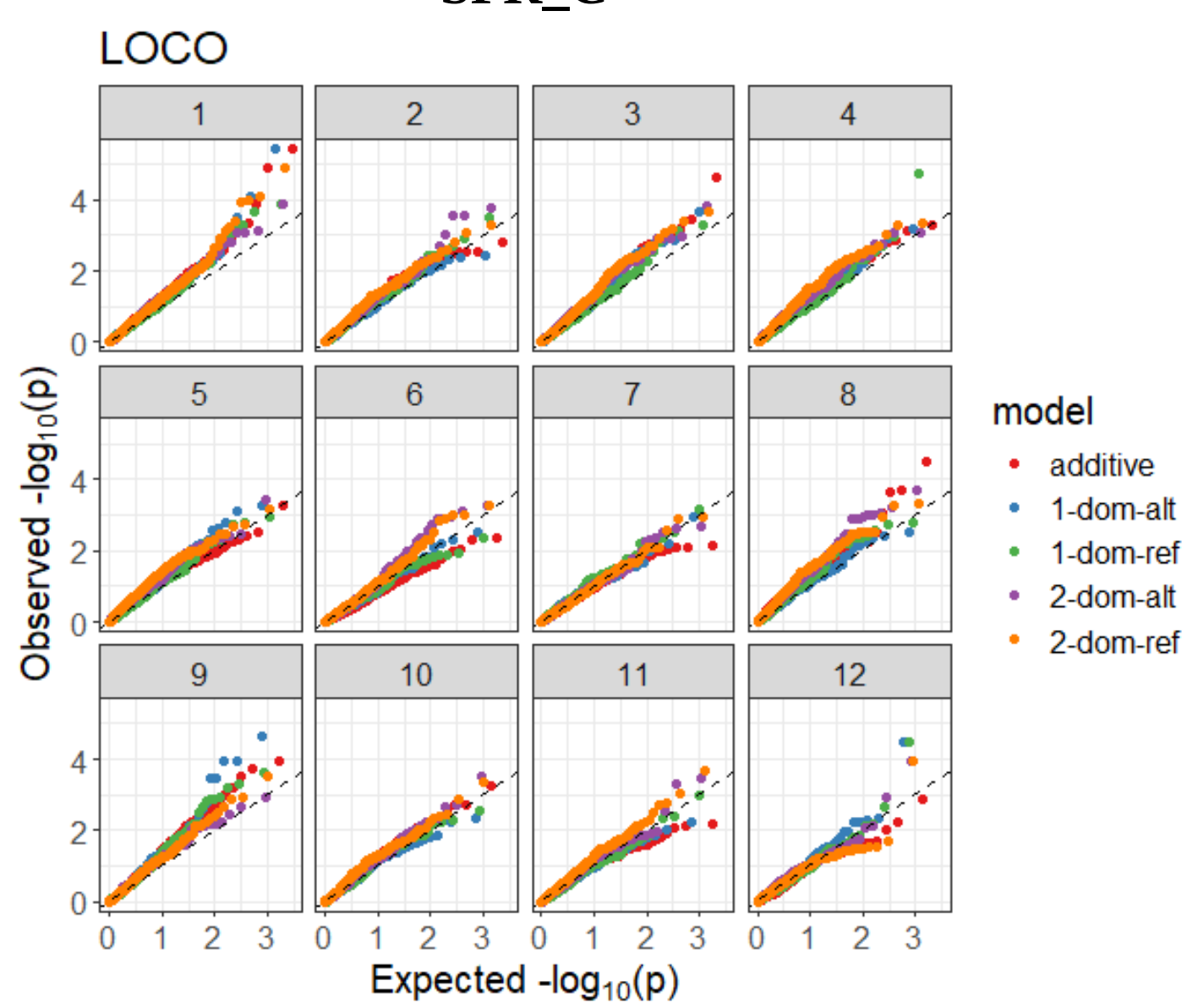

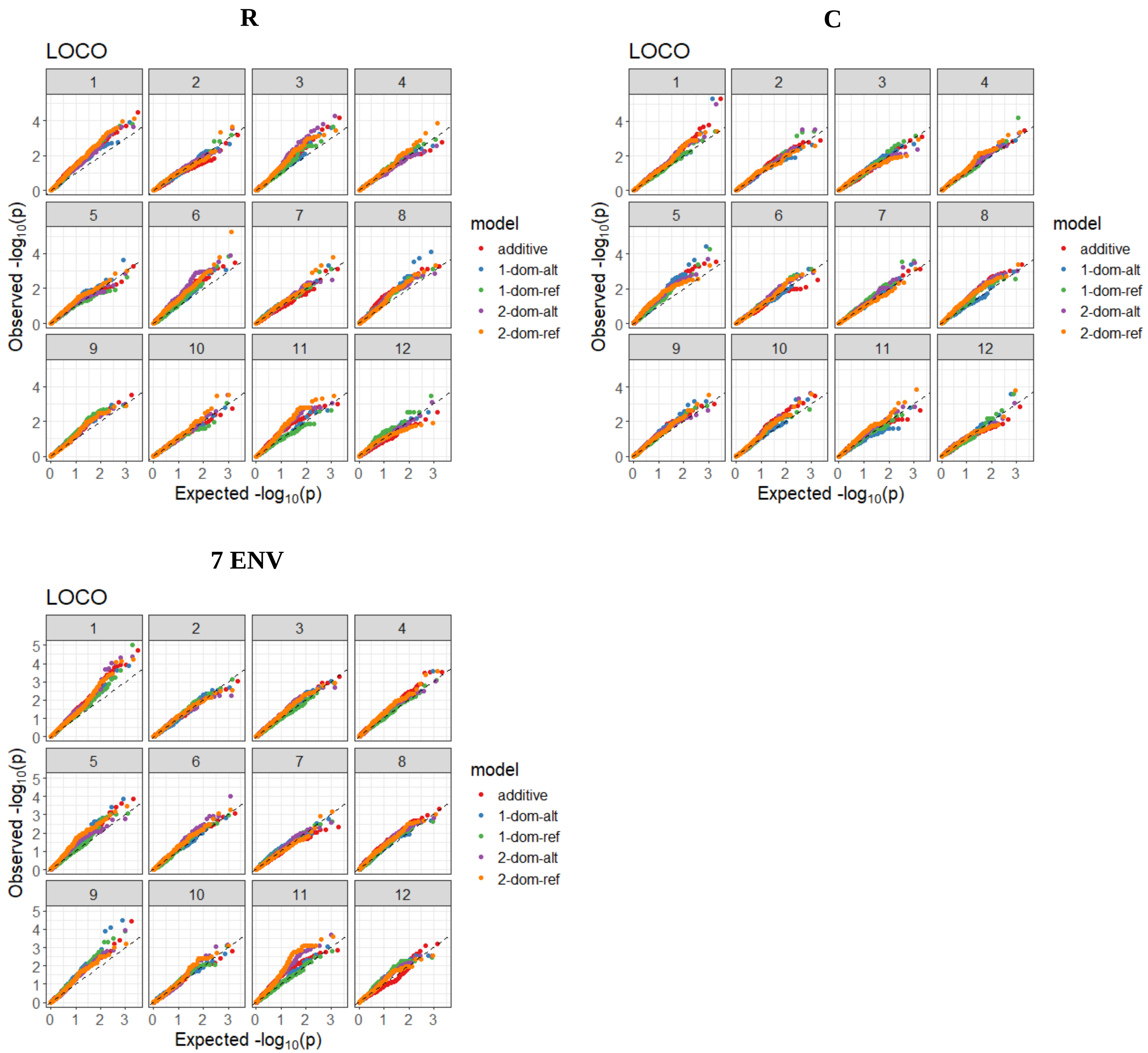

Supplementary Figure S3. QQ plots of observed vs. expected -log10(p values) for potato tuber dormancy using the additive and dominant model when multiple environments were combined for analysis. Based on 216 potato genotypes evaluated in seven environments involving two locations (Dalhart and Springlake, Texas), three years (2019 - 2021), and two storage conditions (room temperature and cold storage). DAL – Dalhart; SPR – Springlake; R – room temperature; C – cold storage; 7 ENV – all seven environments combined.

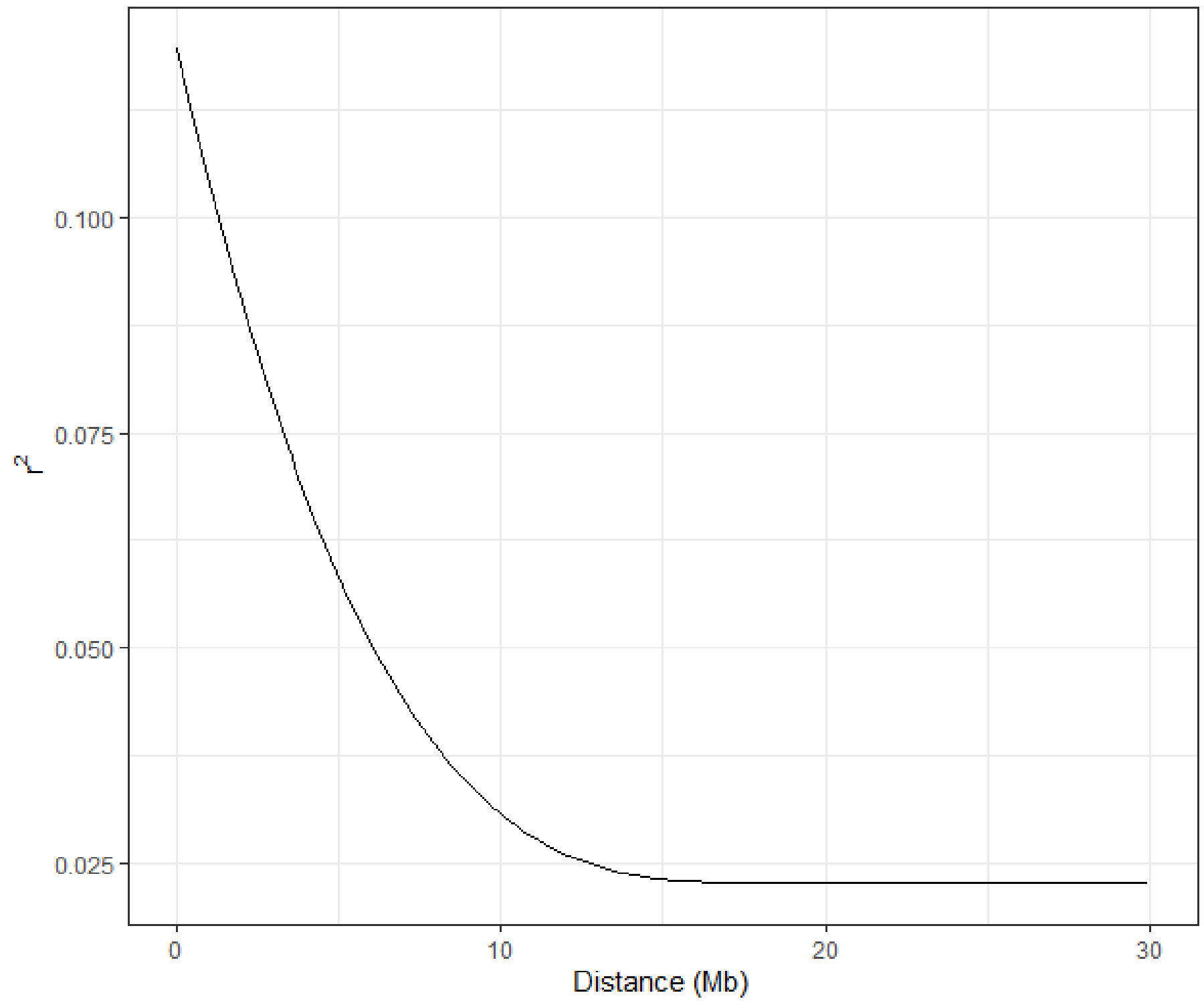

Supplementary Figure S4. Genome-wide linkage disequilibrium in 216 potato genotypes that were evaluated for tuber dormancy in seven environments involving two locations (Dalhart and Springlake, TX), three years (2019-2021), and two storage conditions (room temperature and cold storage).

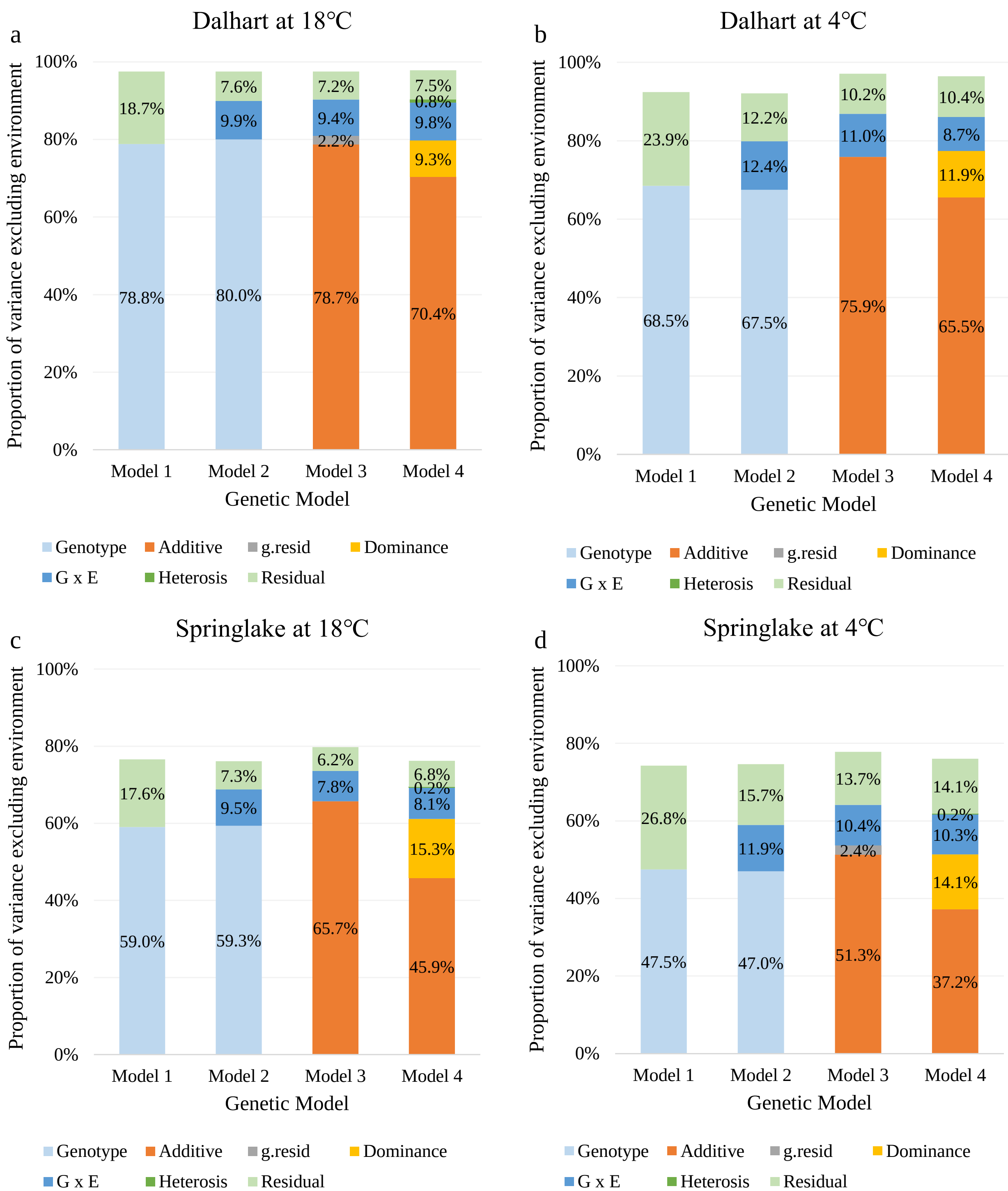

Supplementary Figure S5. Models to partition the proportion of variance excluding the environment. Based on the evaluation of 292 potato genotypes in eleven environments involving two locations (Dalhart and Springlake, TX), four years (2019-2022) and two storage conditions (room temperature and cold storage). a: from Dalhart and stored at room temperature (18 °C); b: from Dalhart and kept in cold storage (4 °C); c: from Springlake and stored at room temperature (18 °C); d: from Springlake and kept in cold storage (4 °C).
