# Supplementary material for: Genome‐wide association studies and genomics‐informed breeding for tuber dormancy in tetraploid potatoes
Source: Plant Genome. 2026 Feb 19;19(1):e70205. doi: 10.1002/tpg2.70205 (PMC12919731; doi:10.1002/tpg2.70205)
Supplement: Supplementary file 5 [file TPG2-19-e70205-s009.pdf]

Genome-wide association studies and genomics-informed breeding for tuber dormancy in tetraploid potatoes

Ao Jiao, Sanjeev Gautam, Jeewan Pandey, Douglas C. Scheuring, Jeffrey W. Koym, and M. Isabel Vales

Supplementary Table S3. Analysis of variance assessing the effect of (A) environments, (B) locations, (C-D) years, (E-F) storage conditions, (G) location - storage combinations, and (H-I) market groups on tuber dormancy length in 216 tetraploid advanced potato genotypes evaluated in Texas (Dalhart and Springlake) for three years (2019-2021) and two storage conditions (18 °C and 4 °C).

(A) Effect of environment (Env) (year-location-storage condition combination) (19DAL-R, 20DAL-R, 20DAL-C, 20SPR-R, 20SPR-C, 21SPR-R, 21SPR-C)

Source of variation	Significance
Genotype	***
Env	ns
rep(Env)	ns
Genotype x Env	***

(B) Effect of location (Loc) when tubers were stored at room temperature (20DAL-R, 20SPR-R)

Source of variation	Significance
Genotype	***
Loc	***
rep(Loc)	*
Genotype x Loc	***

(C) Effect of location when tubers were stored in cold storage (20DAL-C, 20SPR-C)

Source of variation	Significance
Genotype	***
Loc	**
rep(Loc)	ns
Genotype x Loc	***

(D) Three years, two locations and two storage conditions (19DAL-R, 20DAL-R, 20SPR-R, 21SPR-R, 20SPR-C, 21SPR-C)

Source of variation	Significance
Genotype	***
Year	ns
rep(Year)	ns
Genotype x Year	***

(E) Two storage conditions, two locations, and two years (20DAL-R, 20DAL-C, 20SPR-R, 20SPR-C, 21SPR-R, 21SPR-C)

Source of variation	Significance
Genotype	***
Storage	***
rep(Storage)	ns
Genotype x Storage	***

(F) Two locations, two storage conditions and one year (20DAL-R, 20DAL-C, 20SPR-R, 20SPR-C)

Source of variation	Significance
Genotype	***
Loc_Storage	***
rep(Loc_Storage)	ns
Genotype x Loc_Storage	***

(G) Five market groups, (19DAL-C)

Source of variation	Significance
Market Group	***
rep(Market Group)	ns

(H) Five market groups, (19DAL-R, 20DAL-R)

Source of variation	Significance
Market Group	***
Year	ns
rep(Year)	ns
Market Group x Year	ns

(I) Five market groups, (20SPR-R, 21SPR-R)

Source of variation	Significance
Market Group	***
Year	ns
rep(Year)	***
Market Group x Year	ns

(J) Five market groups, (20SPR-C, 21SPR-C)

Source of variation	Significance
Market Group	***
Year	ns
rep(Year)	ns
Market Group x Year	*

ns - not significant; * $p < 0.05$; ** $p < 0.01$; and *** $p < 0.001$
19, 20, 21 - years 2019, 2020, 2021; DAL - Dalhart; SPR - Springlake; C - cold storage - 4 °C; R - room temperature - 18 °C
