# Supplementary material for: Genome‐wide association studies and genomics‐informed breeding for tuber dormancy in tetraploid potatoes
Source: Plant Genome. 2026 Feb 19;19(1):e70205. doi: 10.1002/tpg2.70205 (PMC12919731; doi:10.1002/tpg2.70205)
Supplement: Supplementary file 6 [file TPG2-19-e70205-s001.docx]

Genome-wide association studies and genomics-informed breeding for tuber dormancy in tetraploid potatoes

Ao Jiao, Sanjeev Gautam, Jeewan Pandey, Douglas C. Scheuring, Jeffrey W. Koym, and M. Isabel Vales

**Supplementary Table S2**

**Supplementary Table S2**. Environmental details and field planting and harvesting dates of potatoes evaluated for tuber dormancy length.

| Environment code | Year | Location | Storage condition | Planting date | Vine kill date | Harvesting date |
| --- | --- | --- | --- | --- | --- | --- |
| 19DAL-R | 2019 | Dalhart | Room temperature | 13-May-19 | 29-Aug-19 | 9-Sep-19 |
| 20DAL-R | 2020 | Dalhart | Room temperature | 9-May-20 | 17-Aug-20 | 2-Sep-20 |
| 20DAL-C | 2020 | Dalhart | Cold storage | 9-May-20 | 17-Aug-20 | 2-Sep-20 |
| 20SPR-R | 2020 | Springlake | Room temperature | 26-Mar-20 | 30-Jun-20 | 6-Jul-20 |
| 20SPR-C | 2020 | Springlake | Cold storage | 26-Mar-20 | 30-Jun-20 | 6-Jul-20 |
| 21SPR-R | 2021 | Springlake | Room temperature | 30-Mar-21 | 12-Jul-21 | 19-Jul-21 |
| 21SPR-C | 2021 | Springlake | Cold storage | 30-Mar-21 | 12-Jul-21 | 19-Jul-21 |
| 22DAL-R | 2022 | Dalhart | Room temperature | 6-May-22 | 8-Aug-22 | 18-Sep-22 |
| 22DAL-C | 2022 | Dalhart | Cold storage | 6-May-22 | 8-Aug-22 | 18-Sep-22 |
| 22SPR-R | 2022 | Springlake | Room temperature | 25-Mar-22 | 2-Jul-22 | 17-Jul-22 |
| 22SPR-C | 2022 | Springlake | Cold storage | 25-Mar-22 | 2-Jul-22 | 17-Jul-22 |

Abbreviations: 19, 20, 21, 22 - years 2019, 2020, 2021, 2022; DAL - Dalhart, Texas; SPR - Springlake, Texas; R - room temperature (18 ℃); C - cold storage (4 ℃).
