# Supplementary material for: Genome‐wide association studies and genomics‐informed breeding for tuber dormancy in tetraploid potatoes
Source: Plant Genome. 2026 Feb 19;19(1):e70205. doi: 10.1002/tpg2.70205 (PMC12919731; doi:10.1002/tpg2.70205)
Supplement: Supplementary file 7 [file TPG2-19-e70205-s003.docx]

Genome-wide association studies and genomics-informed breeding for tuber dormancy in tetraploid potatoes

Ao Jiao, Sanjeev Gautam, Jeewan Pandey, Douglas C. Scheuring, Jeffrey W. Koym, and M. Isabel Vales

**Supplementary Table S8**

**Supplementary Table S8**. Akaike information criterion (AIC) of different models to partition the proportion of variance excluding the environment. Based on the evaluation of 292 potato genotypes in eleven environments involving two locations (Dalhart and Springlake, Texas), four years (2019-2022), and two storage conditions (room temperature and cold storage).

| Model | Variance components | AIC | | | |
| --- | --- | --- | --- | --- | --- |
|  |  | DAL_C | DAL_R | SPR_C | SPR_R |
| 1 | genotype, residual | 2054.133 | 3365.589 | 3277.101 | 3281.995 |
| 2 | genotype, genotype x environment, residual | 2051.605 | 3359.291 | 3277.106 | 3274.448 |
| 3 | additive, genotype x environment, residual | 1957.736 | 3169.938 | 3172.813 | 3122.199 |
| 4 | additive, dominance, genotype x environment, residual | 1949.022 | 3157.574 | 3160.104 | 3106.497 |

Abbreviations: DAL - Dalhart, Texas; SPR - Springlake, Texas; R - room temperature (18 ℃); C - cold storage (4 ℃).
