# Supplementary material for: Genome‐wide association studies and genomics‐informed breeding for tuber dormancy in tetraploid potatoes
Source: Plant Genome. 2026 Feb 19;19(1):e70205. doi: 10.1002/tpg2.70205 (PMC12919731; doi:10.1002/tpg2.70205)
Supplement: Supplementary file 8 [file TPG2-19-e70205-s007.docx]

Genome-wide association studies and genomics-informed breeding for tuber dormancy in tetraploid potatoes

Ao Jiao, Sanjeev Gautam, Jeewan Pandey, Douglas C. Scheuring, Jeffrey W. Koym, and M. Isabel Vales

**Supplementary Table S1**

**Supplementary Table S1**. Genotypes evaluated for potato tuber dormancy evaluation across all years (2019 to 2022), locations (Dalhart and Springlake, Texas), and storage conditions (18 ℃ and 4 ℃).

| **Genotype** | **Market Class** | **Market Group** |
| --- | --- | --- |
| Atlantic | Processing | Chipper |
| COTX10080-2Ru | Fresh | Russet |
| Reveille Russet | Fresh | Russet |
| Russet Burbank | Processing | Russet |
| Russet Norkotah | Fresh | Russet |
| Sierra Gold^TM^ | Fresh | Russet |
| Vanguard Russet | Fresh | Russet |
