# Supplementary material for: Genome‐wide association studies and genomics‐informed breeding for tuber dormancy in tetraploid potatoes
Source: Plant Genome. 2026 Feb 19;19(1):e70205. doi: 10.1002/tpg2.70205 (PMC12919731; doi:10.1002/tpg2.70205)
Supplement: Supplementary file 9 [file TPG2-19-e70205-s008.docx]

Genome-wide association studies and genomics-informed breeding for tuber dormancy in tetraploid potatoes

Ao Jiao, Sanjeev Gautam, Jeewan Pandey, Douglas C. Scheuring, Jeffrey W. Koym, and M. Isabel Vales

**Supplementary Table S5**

**Supplementary Table S5**. Estimates of variance components for potato tuber dormancy based on 216 genotypes evaluated for three years (2019-2021) at two locations (Dalhart and Springlake, Texas) under two storage conditions (18 ℃ and 4℃).

| Variance component | Estimate | Standard error | Wald p-value | Percent of total variance |
| --- | --- | --- | --- | --- |
| A. Seven environments: 19DAL-R, 20DAL-R, 20DAL-C, 20SPR-R, 20SPR-C, 21SPR-R, 21SPR-C | | | | |
| Genotype^a^ | 83.37 | 21.19 | <0.0001 | 4.46 |
| Location | 41.56 | 116.52 | 0.7214 | 2.23 |
| Storage | 1337.63 | 1913.04 | 0.4844 | 71.61 |
| Year[Location] | 46.45 | 51.83 | 0.3701 | 2.49 |
| Rep[Location,Year] | 0.55 | 0.62 | 0.3733 | 0.03 |
| Genotype x Location | 36.92 | 8.64 | <0.0001 | 1.98 |
| Genotype x Storage | 141.25 | 17.64 | <0.0001 | 7.56 |
| Location x Storage | 22.33 | 41.57 | 0.5911 | 1.20 |
| Genotype x Location x Storage | 26.45 | 8.49 | 0.0018 | 1.42 |
| Year x Genotype[Location] | 15.75 | 5.33 | 0.0031 | 0.84 |
| Year x Storage[Location] | 8.21 | 11.26 | 0.4656 | 0.44 |
| Year x Storage x Genotype[Location] | 26.04 | 6.59 | <0.0001 | 1.39 |
| Residual | 81.55 | 3.23 |  | 4.37 |
| Total | 1868.08 | 1917.39 |  | 100.00 |
| B. Room temperature: 19DAL-R, 20DAL-R, 20SPR-R, 21SPR-R | | | | |
| Genotype | 275.17 | 29.17 | <0.0001 | 59.66 |
| Rep[Year,Location] | 0.53 | 0.60 | 0.3719 | 0.12 |
| Year[Location] | 68.23 | 68.83 | 0.3216 | 14.79 |
| Location | -6.79 | 52.14 | 0.8964 | 0.00 |
| Genotype*Location | 15.87 | 5.62 | 0.0048 | 3.44 |
| Year*Genotype[Location] | 38.75 | 5.23 | <0.0001 | 8.40 |
| Residual | 62.72 | 3.15 |  | 13.60 |
| Total | 461.26 | 74.80 |  | 100.00 |
| C. Cold storage: 20DAL-C, 20SPR-C, 21SPR-C | | | | |
| Genotype | 155.65 | 27.97 | <0.0001 | 22.62 |
| Rep[Year,Location] | 0.13 | 0.58 | 0.8270 | 0.02 |
| Year[Location] | 47.11 | 67.45 | 0.4849 | 6.85 |
| Location | 190.57 | 325.09 | 0.5577 | 27.69 |
| Genotype*Location | 137.82 | 24.51 | <0.0001 | 20.03 |
| Year*Genotype[Location] | 46.54 | 11.62 | <0.0001 | 6.76 |
| Residual | 110.39 | 7.01 |  | 16.04 |
| Total | 688.21 | 322.78 |  | 100.00 |

^a^Significant variance components (with a p-value smaller than 0.05) are colored in red.

Abbreviations: 19, 20, 21, 22 - years 2019, 2020, 2021, 2022; DAL - Dalhart, Texas; SPR - Springlake, Texas; R - room temperature (18 ℃); C - cold storage (4 ℃).
