# Supplementary material for: Genome‐wide association studies and genomics‐informed breeding for tuber dormancy in tetraploid potatoes
Source: Plant Genome. 2026 Feb 19;19(1):e70205. doi: 10.1002/tpg2.70205 (PMC12919731; doi:10.1002/tpg2.70205)
Supplement: Supplementary file 10 [file TPG2-19-e70205-s011.docx]

**Supplementary File S1**. R script to identify candidate genes (using the potato reference genome of Solanum tuberosum group Phureja DM1-3 PGSC v4.03 from SpudDB) within a 2-Mb (Megabase) flanking genomic region of the peak of each QTL detected for the length of potato tuber dormancy.

rm(list = ls())

library(tidyverse)

\# read in the gff

PGSC_DM_V403_representative_genes <- read.delim("PGSC_DM_V403_representative_genes.gff", header=FALSE, comment.char="#")

gff<-PGSC_DM_V403_representative_genes

\# subset and clear up the gff - add names

colnames(gff) <- c("chr", "source", "feature", "start", "end", "score",

"strand", "frame", "attribute")

gff<-subset(gff, chr=="ST4.03ch01")

\# select genes only

new_gff <- gff %>% filter(feature == "gene")

\# arrange the gff

new_gff <- new_gff %>% arrange(start, end)

\# make a gene mid point variable

new_gff <- new_gff %>% mutate(mid = start + (end-start)/2)

\# plot selection scan again

#ggplot(windowStats, aes(mid/10^6, Fst_Red_RN)) + geom_point()

\# identify the highest peak of selection

#hits <- windowStats %>% arrange(desc(Fst_ChipRu_Red)) %>% top_n(3)

hits<- read.delim("chr1.txt")

#get hits from top 5% Fst intersecting with either of the gene estimates

hitsuniqmid<-hits %>% distinct(mid, .keep_all = TRUE)

hits<-hitsuniqmid

nrow(hits)

x <- hits$mid[1]

\# find hits within 2 Mb

for (i in 1:nrow(hits)) {

x <- hits$mid[i]

gene_hits <- new_gff %>% mutate(hit_dist = abs(mid - x)) %>% arrange(hit_dist)%>% filter(hit_dist < 2000000)

assign(paste0("gene_hits",i), gene_hits %>% select(chr, start,end,mid, attribute,hit_dist))

}

name<-paste("gene_hits",1:nrow(hits), sep="" ,collapse=", ")

print(name)

bind<-rbind(gene_hits1)

a<-bind %>% separate(attribute,c("ID", "Name"), ";")

df<-a %>% select(chr, start, end, mid, ID, Name, hit_dist)

write.csv(df, "Chr1_2MB.csv")
